# A U-system approach for predicting metabolic behaviors and responses based on an alleged metabolic reaction network

**DOI:** 10.1186/1752-0509-8-S5-S4

**Published:** 2014-12-12

**Authors:** Kansuporn Sriyudthsak, Yuji Sawada, Yukako Chiba, Yui Yamashita, Shigehiko Kanaya, Hitoshi Onouchi, Toru Fujiwara, Satoshi Naito, Ebernard O Voit, Fumihide Shiraishi, Masami Yokota Hirai

**Affiliations:** 1 RIKEN Center for Sustainable Resource Science, Yokohama 230-0045, Japan; 2 JST, CREST, Kawaguchi 332-0012, Japan; 3 Graduate School of Life Science, Hokkaido University, Sapporo 060-0810, Japan; 4 Faculty of Science, Hokkaido University, Sapporo 060-0810, Japan; 5 Graduate School of Information Science, NARA Institute of Science and Technology, Nara 630-0192, Japan; 6 Graduate School of Agriculture, Hokkaido University, Sapporo 060-8586, Japan; 7 Graduate School of Agricultural and Life Sciences, University of Tokyo, Tokyo 113- 8657, Japan; 8 The Wallace H. Coulter Department of Biomedical Engineering, Georgia Institute of Technology and Emory University, Atlanta, GA 30332-0535, USA; 9 Graduate School of Bioresource and Bioenvironmental Sciences, Kyushu University, Fukuoka 812-8581, Japan

**Keywords:** Arabidopsis thaliana, kinetic parameter, mathematical modeling, metabolic reaction network, metabolomics

## Abstract

**Background:**

Progress in systems biology offers sophisticated approaches toward a comprehensive understanding of biological systems. Yet, computational analyses are held back due to difficulties in determining suitable model parameter values from experimental data which naturally are subject to biological fluctuations. The data may also be corrupted by experimental uncertainties and sometimes do not contain all information regarding variables that cannot be measured for technical reasons.

**Results:**

We show here a streamlined approach for the construction of a coarse model that allows us to set up dynamic models with minimal input information. The approach uses a hybrid between a pure mass action system and a generalized mass action (GMA) system in the framework of biochemical systems theory (BST) with rate constants of 1, normal kinetic orders of 1, and -0.5 and 0.5 for inhibitory and activating effects, named Unity (U)-system. The U-system model does not necessarily fit all data well but is often sufficient for predicting metabolic behavior of metabolites which cannot be simultaneously measured, identifying inconsistencies between experimental data and the assumed underlying pathway structure, as well as predicting system responses to a modification of gene or enzyme. The U-system approach was validated with small, generic systems and implemented to model a large-scale metabolic reaction network of a higher plant, *Arabidopsis*. The dynamic behaviors obtained by predictive simulations agreed with actually available metabolomic time-series data, identified probable errors in the experimental datasets, and estimated probable behavior of unmeasurable metabolites in a qualitative manner. The model could also predict metabolic responses of *Arabidopsis *with altered network structures due to genetic modification.

**Conclusions:**

The U-system approach can effectively predict metabolic behaviors and responses based on structures of an alleged metabolic reaction network. Thus, it can be a useful first-line tool of data analysis, model diagnostics and aid the design of next-step experiments.

## Background

**Figure 1:**
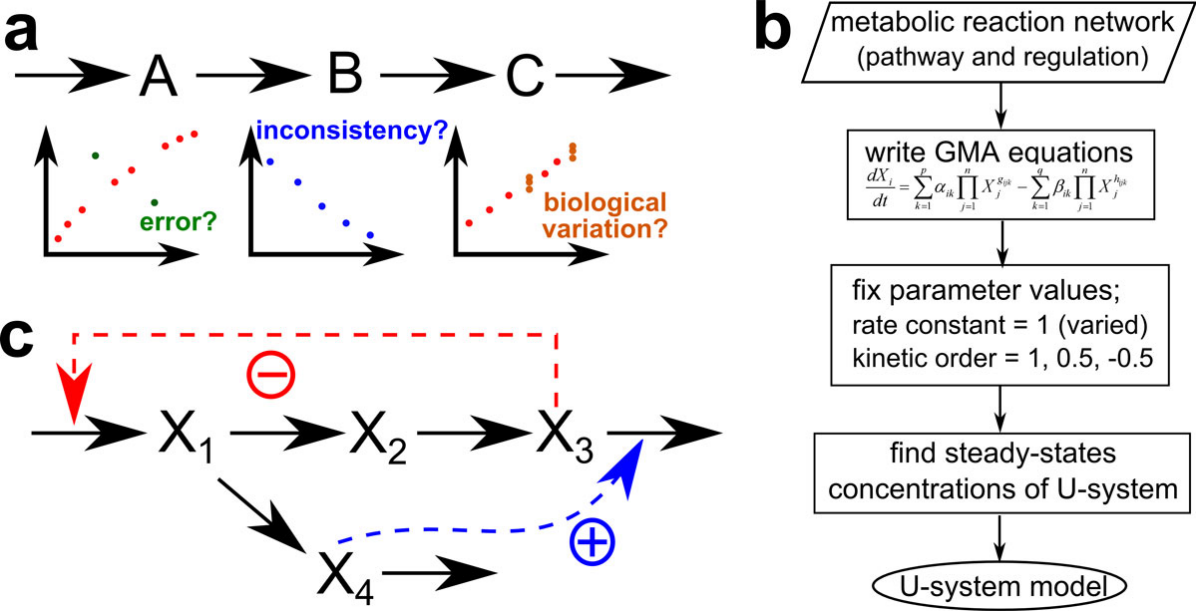
**Examples for typical metabolic pathways and U-system approach**. a Simple linear pathway. A, B, and C represent metabolites. b Diagram for constructing a mathematical model using U-system approach. c Branched pathway with inhibition (⊕) and activation (⊖). *X_i _*(*i*=1, 2, 3 and 4) represent metabolites.

Systems biology have advanced far enough that it is becoming possible to gain a comprehensive understanding of a metabolic system, and to use this knowledge for developing rational treatment options for diseases or devising strategies for increasing the productivity of foods and chemicals. While the main hallmark of systems biology is often portrayed as a means for predicting systems responses to external or internal stimuli, a similarly important aspect is its abilities to envisage all relevant information and integrate experimental data into logical and computable structures. As an example, suppose that three metabolites, A, B, and C, form a linear pathway (Figure 1a). Supposing further that only experimental data for A and C are available and the data show A and C increasing after stimulating the reaction producing A. On the basis of the network structure together with the available data, even though B may not be ably measured, it is logically predictable that B is probably increased as well. On the other hand, supposing that the data for all three metabolites, A, B, and C, are available and the data show that A and C increasing whereas B decreasing. Without any information on the structure of pathway, one can only take the data of B at face value and deem them correct. However, if there is an indication showing that A, B, and C, form a linear pathway like the previous case, it becomes immediately clear that there is some inconsistencies. According to the logic of a conceptual or mathematical model of the pathway, either some branches or regulatory signals are missing, or the data are unreliable. To be more precise, if the data behave in a reasonable trend with some biological fluctuations like those in orange dots (Figure 1a), one may assume that these data just contain intrinsic biological variations and some missing pathways or regulations make the data inconsistent. In contrast, if some data split out of the trend in an unusual way like those in green dots (Figure 1a), one may judge that these data are prone to be errors and probably unreliable. This implies that the logical concepts allow us to assess associated data and possibly identify inconsistencies.

The exploitation of logical concepts for the prediction of probable behaviors of unmeasurable data and identification of uncertainties in datasets becomes much more complicated and important if the pathway systems are large. Large-scale datasets, and in particular time series data characterizing metabolic systems, are also becoming more and more commonplace to assist the investigation. To acquire such datasets with reasonable efforts, the high-throughput analytical instruments have been developed in recent times [1-3]. Given the nature of experiments, however, it is not even always certain that all information for interested variables can be gained from experiments and not all metabolites in the large-scale pathway can be measured. It also cannot be guarantee that the replicated samples in terms of biological, technical and analytical aspects reproduce the same results, even in a qualitative sense. Two major questions thus arise. The first question is whether computational methods of systems biology which are cheap and straightforward may be able to predict or analyze such unmeasurable data with respect to system identification based on our understanding of the system. The second question is how reliable or accurate biological data are, especially if they were obtained from different cellular compartments or from different developmental stages in plants and animals.

It is therefore useful to explore to what degree it might be possible to employ the logic of the pathway to address these issues. Such an analysis seems to be feasible in principle, because the biologist executing the experiments usually has a relatively well-supported concept of the topology of the pathway system. The challenge is that even small pathways quickly become too complicated for intuitive assessments, and larger and more complicated pathway systems simply prevent us from testing assumptions or hypotheses without computational support. Such computational support of course requires mathematical models, which immediately leads to the challenge of setting up detailed, quantitatively appropriate models with a minimum of input requirements. The goal of such models is not necessarily to provide a quantitatively perfect picture of a pathway or to fit all data with great accuracy. Instead, these models should be easy to construct, robust, and merely able to identify how data within a large dataset could probably be.

Power-law models within Biochemical Systems Theory (BST; [4]) possess two great advantages that can be leveraged toward predicting the unmeasurable data as well as testing the consistency between the measured data and an alleged pathway structure and its regulation. First, symbolic BST models can be designed immediately for a pathway system of any complexity. These symbolic models do not include parameter values, but they do define a model's potential repertoire of responses, rather than addressing specific data fits. Second, the estimation of optimal parameter values for large systems is generally fraught with technical difficulties [5]. This issue is greatly ameliorated for BST models, because even relatively coarse numerical settings of their parameters are often sufficient to capture the behavior of a metabolic pathway system in a semi-quantitative fashion.

Here we capitalize on these two features of BST and propose a coarse test for the prospect of metabolic time-series data and consistency between the data and an alleged pathway diagram. The purpose is not to establish optimally fitting models but to identify how the unmeasurable data of metabolites located in a focused pathway probably behave, which time-series data within the dataset may be inconsistent with the understanding of pathway, as well as which data within a large dataset are probably reliable and which, if any, are most likely imprecise. We use a hybrid between a pure mass action system and a generalized mass action (GMA) system in the framework of BST with kinetic orders of 1, which allows for inhibitory and activating effects that are modeled with kinetic orders of -0.5 and 0.5. The rate constants are at first set to 1, but may be later subjected to order-of-magnitude adjustments, which obviously improve the model representation of the data. Once the parameter values are fixed, their effects are to some degree compensated by adjustments in metabolite concentrations according to the network structure so that we named this simplified method unity (U)-system. The diagram for U-system approach is also exhibited in Figure 1b. We begin the demonstration of the U-system approach with artificial "data" from fully known, representative models and show that coarse values for kinetic orders and rate constants retain much of the qualitative behavior of true model responses. These U-system solutions are rather coarse, but can be improved with order-of-magnitude adjustments of flux split ratios at branched points. We then demonstrate the feasibility of the method with actual data describing amino acid synthesis in *Arabidopsis*. This pathway system contains 351 metabolites and 441 fluxes, which are subject to various regulatory mechanisms. Apart from these, we have measurements from two different analytical methods, one comprising 268 metabolites, including 21 amino acids, and the other one accounting for 16 amino acids. As expected, although a large number of data can be measured, it is clear that not all data can be obtained. Also, none of the obtained data contain exactly the same values even in the replicated samples, mainly due to high biological variation among samples. In most cases, the measured time series are qualitatively the same, but in some cases they differ. We show that the U-system analysis estimates the probable behaviors of unmeasurable data and identifies at least some of the unreliable data. Besides, the U-system model is also applicable to predict metabolic responses when a metabolic reaction is altered.

## Results and discussion

### Testing of feasibility of assumptions in U-system

Obviously, a system with arbitrary parameter values of 1 is quite different from a system with diverse parameter values. The performances and characteristics of U-system approach were simply approved using a simple model of a linear pathway by comparing with the typical model constructed in Michaelis-Menten format where the enzymatic reaction velocity is usually determined by an *in vitro *experiment (Supplementary Information - additional file 1). The result showed that U-system model can produce qualitatively similar results to the original. Thus, a simple generic, yet representative model was used to test to what degree the U-system model can produce qualitatively comparable results.

Many studies in recent years have used a simple, representative model of a branched pathway with inhibition and activation (Figure 1c), for instance, to test new parameter estimation methods [6-8]. We used this same system but slightly modified it to allow a wider range of responses. Equations (1)-(4) show the differential equations together with actual parameters for the rate constants and kinetic orders of this system in GMA-system format.

(1) $$\frac{dX_{1}}{dt}=\alpha_{1}X_{3}^{g13}-\beta_{11}X_{1}^{h111}-\beta_{12}X_{1}^{h112}=12X_{3}^{-0.8}-8X_{1}^{0.5}-2X_{1}^{0.5}$$

(2) $$\frac{dX_{2}}{dt}=\alpha_{2}X_{1}^{g21}-\beta_{2}X_{2}^{h22}=8X_{1}^{0.5}-3X_{2}^{0.75}$$

(3) $$\frac{dX_{3}}{dt}=\alpha_{3}X_{2}^{g32}-\beta_{3}X_{3}^{h33}X_{4}^{h34}=3X_{2}^{0.75}-5X_{3}^{0.5}X_{4}^{0.2}$$

(4) $$\frac{dX_{4}}{dt}=\alpha_{4}X_{1}^{g41}-\beta_{4}X_{4}^{h44}=2X_{1}^{0.5}-6X_{4}^{0.8}$$

#### Effect of rate constants on metabolic behaviors

**Figure 2:**
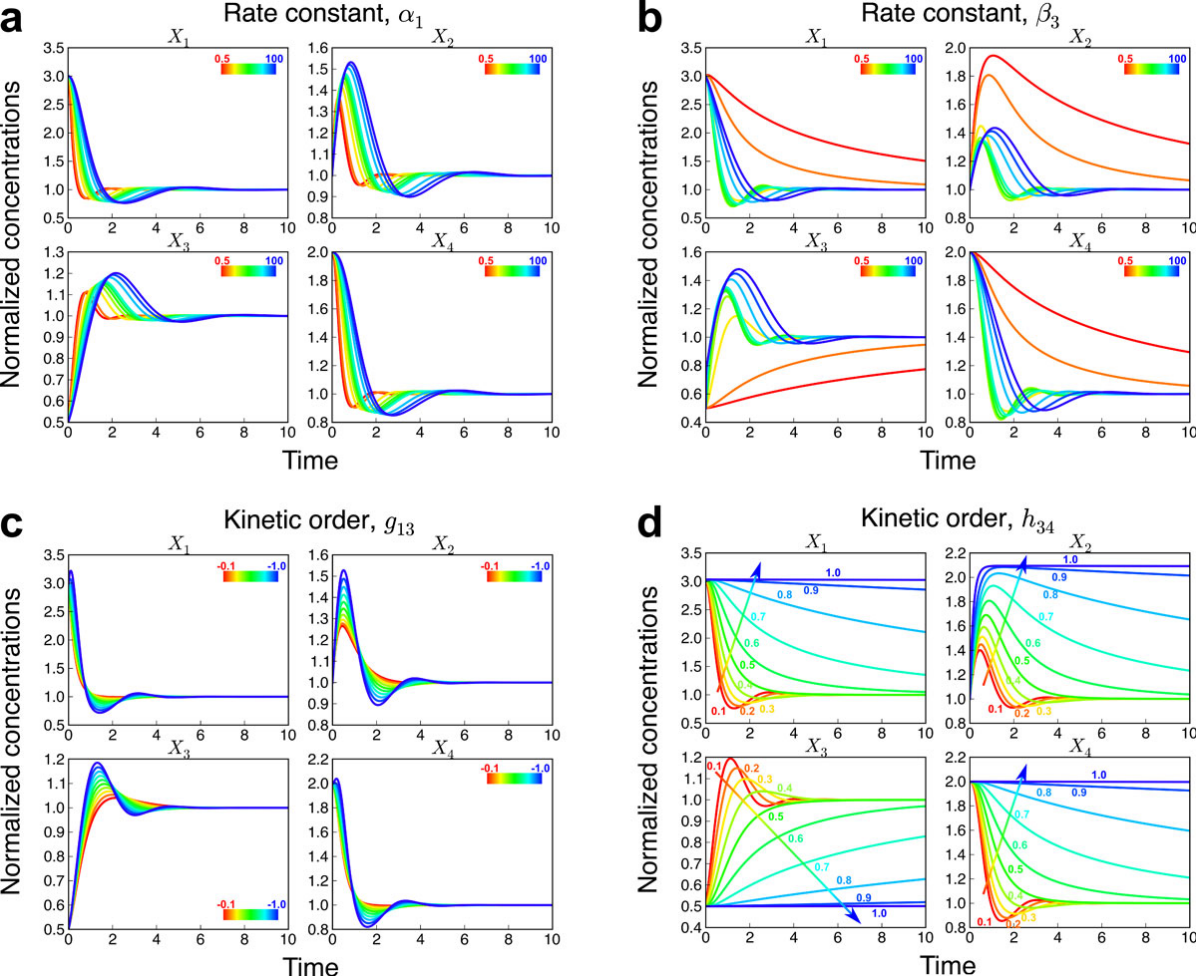
**Normalized concentrations of each metabolite in response to variations of rate constants and kinetic orders**. a Rate constant *α*_1 _was varied at 0.5, 1, 5, 10, 15, 20, 25, 50, 75 and 100 in rainbow- colored lines varying from red to blue, respectively. b Rate constant *β*_3 _was varied at 0.5, 1, 5, 10, 15, 20, 25, 50, 75 and 100 in rainbow- colored lines varying from red to blue, respectively. c Kinetic order *g*_13 _was varied at 0.5, 1, 5, 10, 15, 20, 25, 50, 75 and 100 in rainbow- colored lines varying from red to blue, respectively. d Kinetic order *h*_34_was varied at 0.5, 1, 5, 10, 15, 20, 25, 50, 75 and 100 in rainbow- colored lines varying from red to blue, respectively.

Figure 2a and 2b show patterns of normalized concentrations for each metabolite over time resulting from changes in rate constants *α*_1 _and *β*_3_, respectively. It is clear that when *α*_1 _is increased, the time-course curves of metabolite concentrations tend to stretch along the x-axis, whereas the extension along the y-axis is only slight. Although the results for *β*_3 _are clearly different from those for *α*_1_, the dynamic responses for each *X*_i _are qualitatively similar. Overall, changes in the rate constants tend to affect the dynamics in the direction of the x-axis. The individual degree of elongation depends on the specifics of the system. This result is not surprising, because multiplying the entire system with a positive constant corresponds to changing the unit of time and thereby the scale of the x-axis (see, *e.g*., [9]). Additional results are presented in Supplementary Figures S3 and S4 (additional file 1).

#### Effect of kinetic orders on metabolic behaviors

Figure 2c and 2d show patterns of normalized concentrations of each metabolite over time resulting from changes in kinetic orders *g*_13 _and *h*_34_, respectively. When *g*_13 _is changed, only the magnitudes of normalized metabolite concentrations are changed but almost no difference is observed in temporal changes in time-course patterns of metabolite concentrations. Similar results are also observed for *g*_21_, *g*_32_, *g*_41_, *h*_111_, *h*_112_, *h*_22_, *h*_33 _and *h*_44 _(Supplementary Figures S5 and S6 - additional file 1). This consistency may be due to the fact that the ranges of kinetic orders are typically between −1 and 1 [9], so that kinetic orders do not play an overly strong role in the variation of metabolite concentrations, and the corresponding fluxes do not change much. The results for *h*_34 _(Figure 2d) and *h*_44 _(Supplementary Figure S6 - additional file 1) are different, as the trajectories noticeably elongate in the direction of the x-axis with changes in kinetic orders. According to metabolic reaction network diagram in Figure 1c, one sees that these kinetic orders are involved in the degradation of X_3_, so that an increase in kinetic order strongly increases the degradation flux of X_3_. However, this situation is somewhat unusual because of the high activation of the efflux. In this case, X_4 _is produced from X_1 _which is inhibited by X_3 _so that a strong activation of the efflux of X_3 _by X_4 _quite effectively slows down changes in X_3_. Also, X_3 _has a low initial value and kinetic order compared to other metabolites. Thus, the dynamics of X_3 _is driven by other metabolites and *X*_3 _concentration does not change much unless the other parameters vary. Furthermore, the input X_1 _is strongly affected by X_3 _which does not change much. Accordingly, other metabolites hardly change throughout the period of observed time. In general, the kinetic parameters for activation are seldom higher than kinetic parameters for the metabolite itself so that this particular scenario is rare. As a consequence, it is still able to deduce that changes of kinetic orders tend to affect the trajectories in the direction of the y-axis rather than the x-axis.

#### Effect of random parameters on metabolic behaviors using Monte-Carlo simulation

**Figure 3:**
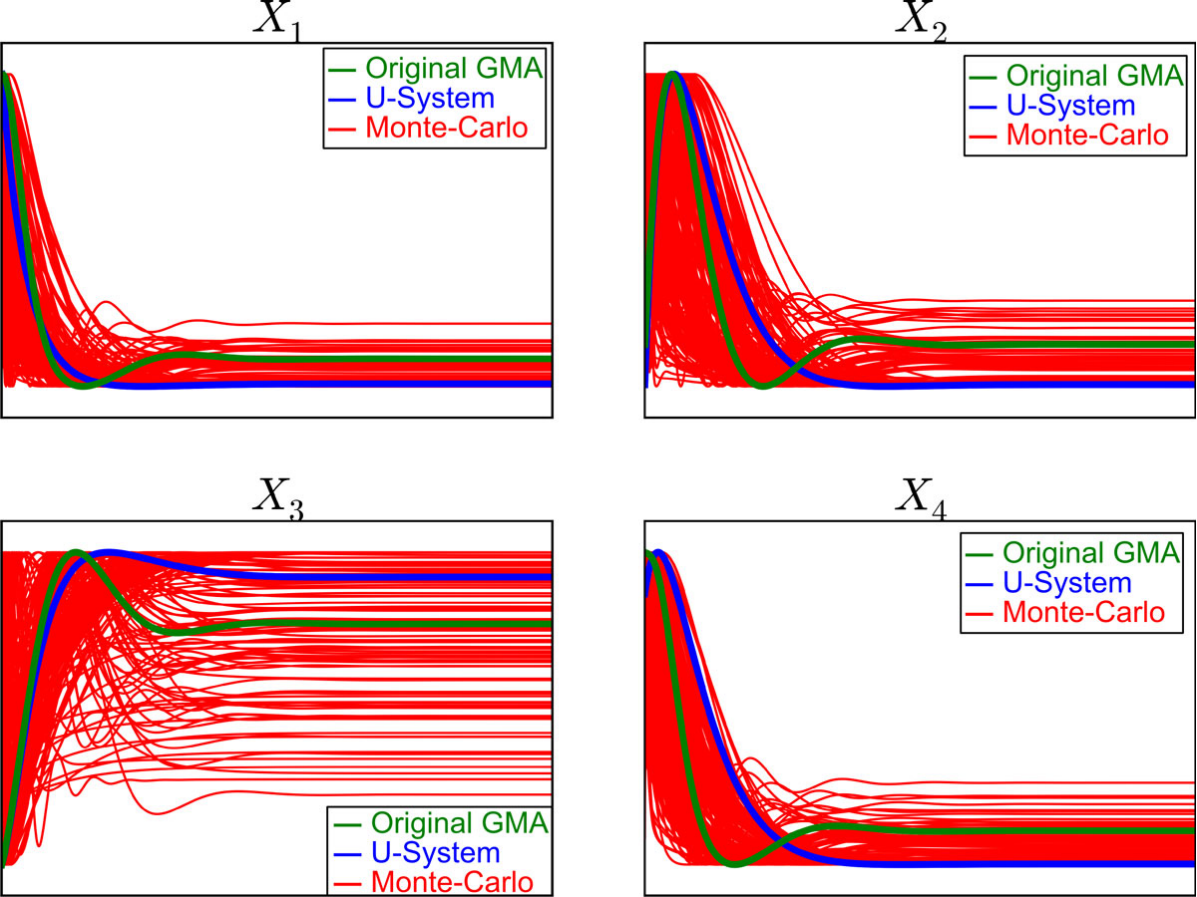
**Comparisons among Monte-Carlo simulations, U-system simulations and original GMA model simulations**. The U-system simulations and original GMA model simulations are shown in blue and green lines, respectively. The Monte-Carlo simulations in response to changes of rate constants within ranges of 0.5 and 20 and kinetic orders between 0.2(-0.2) and 0.8(-0.8) are shown in red lines. The concentrations of *X_i _*along the *y*-axis are scaled using maximum and minimum values of each simulation, while the time along the *x*-axis is scaled using maximal values of the *X_i _*concentrations before they return to their steady-states.

Figure 3 shows a selection of Monte-Carlo simulation results associated with changes in the rate constants between 0.2 and 20, and kinetic orders between 0.2 (-0.2) and 0.8 (-0.8) for the branched pathway model with inhibition and activation as well as the simulations for U-system model (blue lines) and original GMA-system model (green lines). Parameter combinations not leading to a stable system were removed. For the U-system model, all rate constants and kinetic orders of substrates and enzymes were set to 1, while the kinetic orders of parameters for inhibitions and activations were set to be -0.5 and 0.5, respectively. As a result, the observed metabolite concentrations are no longer the real concentrations, but simply indications of the shapes of their trajectories. The present study calls these concentrations as U-system concentrations (Figure 1b).

The initial values of metabolite concentrations of *X*_1_, *X*_2_, *X*_3_, and *X*_4 _were set to 3-, 1-, 0.5- and 2-fold their steady-state values. The simulated metabolite concentrations for all cases were normalized by their steady-state values for the observation and comparison. To facilitate inspection of the calculated behaviors of the metabolite concentrations, the scales of both axes were adjusted by the maximum and minimum values of each simulation. The result shows that most Monte-Carlo simulations exhibit qualitatively comparable dynamic responses regardless of the parameter values chosen. Each metabolite concentration changes throughout the period of time and returns to its steady-state value in a similar manner. This observation implies that both models provide qualitatively comparable dynamic patterns of changes in metabolite concentrations. Furthermore, the GMA and U-system responses are surrounded by the Monte-Carlo simulation results for 1000 parameter sets.

In summary of this analysis, the U-system approach indicates that metabolic behaviors mainly depend on the network structure of a metabolic system. Unlike most of modeling methods, it does not need the process of parameter estimation in model construction, although it cannot offer actual quantitative concentrations of metabolic behaviors. This insight leads to several consequences. First, the U-system approach provides a coarse time-transient behavior of the system regardless of the requirements or dependencies of experimental data. Accordingly, it will not confront difficulties in parameter fitting, especially in a large-scale system. Second, the U-system approach is simple. Plausible parameters for inhibition and activation can be easily set into the model to grasp their possible effects, which makes it possible to design additional experiments. Third, the U-system approach is suitable for practical applications because of its simplicity and flexibility. One can simply construct a mathematical model based on only experimental facts regarding the network structure and regulation. If the U-system simulations are inconsistent with experimental data, other possible systems may be simply constructed to find out unknown candidates. As a result, an appropriate experiment may be designed to validate or refute the predictions from the simulation and to characterize biological details. Lastly, the U-system approach allows us to acquire tendencies of metabolic behaviors to assess experimental data within the context of their actual network structure.

### Application to metabolic reaction network of *Arabidopsis*

**Figure 4:**
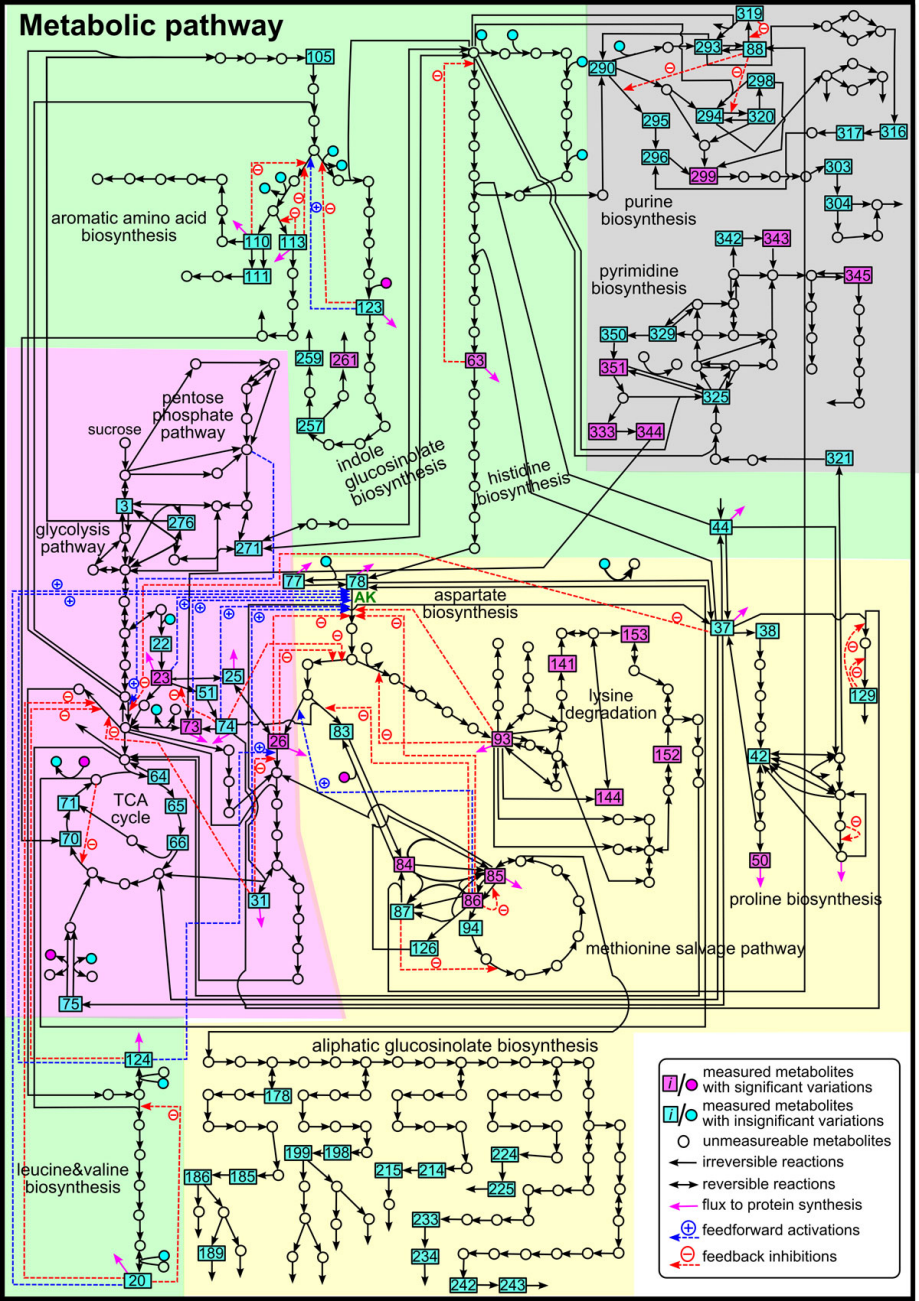
**Metabolic reaction network of *Arabidopsis *focused in this study**. Green zone, aromatic amino acid biosynthesis, indole GSL biosynthesis, histidine biosynthesis and leucine and valine biosynthesis; gray zone, purine and pyrimidine biosynthesis; pink zone, glycolysis pathway, TCA cycle and pentose phosphate pathway; yellow zone, aspartate-family amino acid biosynthesis, proline biosynthesis, lysine degradation, methionine salvage pathway, aliphatic GSL biosynthesis. The number *i *in square boxes indicate the metabolite numbers arbitrarily given for simulations (Supplementary Model - additional file 2).

Methionine, lysine and threonine are essential amino acids for non-ruminant animals including humans. The methionine derivative, *S*-adenosyl methionine (AdoMet), is a major primary methyl-group donor and also a precursor for a plant hormone ethylene and polyamines. To increase the sulfur-containing amino acids methionine and cysteine in crops, numerous researches in the field of genetics and metabolic engineering have been carried out for more than two decades, and the objectives have been partially realized. However, the results are not quite satisfactory yet, because the amino acids and related metabolites in the obtained transgenic crops are not well balanced. One of the major causes for not achieving a good balance is an incomplete understanding of the whole regulatory mechanisms of amino acid accumulation, as well as the regulatory feedback structure of the system, although it is well known that the accumulation of methionine, a member of the aspartate-family amino acids together with lysine and threonine, is tightly controlled by negative feedback by lysine and threonine [10]. Another cause is an ambiguity in the characteristics of the metabolic flow pattern, which affects productivity and metabolic balance [11]. To elucidate their correlations among metabolites, there are various researches focusing on the integrations of *in vitro *data into kinetic models for specific pathways [12,13] and the reconstructions of metabolic fluxes at the genome scale [14,15]. However, no large-scale kinetic models including regulatory mechanisms are known for the *Arabidopsis *metabolic system. In this study, therefore, we exploited the U-system approach to construct a large-scale mathematical model based on available metabolic reaction networks (Figure 4) for describing important characteristics of its metabolic system and predicting metabolic behaviors in whole. A perturbation experiment was also conducted with *Arabidopsis *callus by exogenous application of lysine and threonine to obtain metabolic time-series data for validating the U-system model.

#### Modified U-system approach with rough adjustments of rate constants

The mathematical model including nonlinear ordinary differential equations (ODEs) for 351 metabolites was constructed by U-system approach on the basis of only structures of metabolic reaction networks available in databases (Supplementary Model - additional file 2). Fick's laws of diffusion [16] was used for representing the uptake rate of lysine plus threonine from the callus culture medium to the inside of the cells from *t*=0, and the parameters for uptake rates were estimated using experimental data. For simulations, all ODEs representing changes of metabolite concentrations inside the cells were calculated together with equations for the supplementation throughout a period of 100 h.

**Figure 5:**
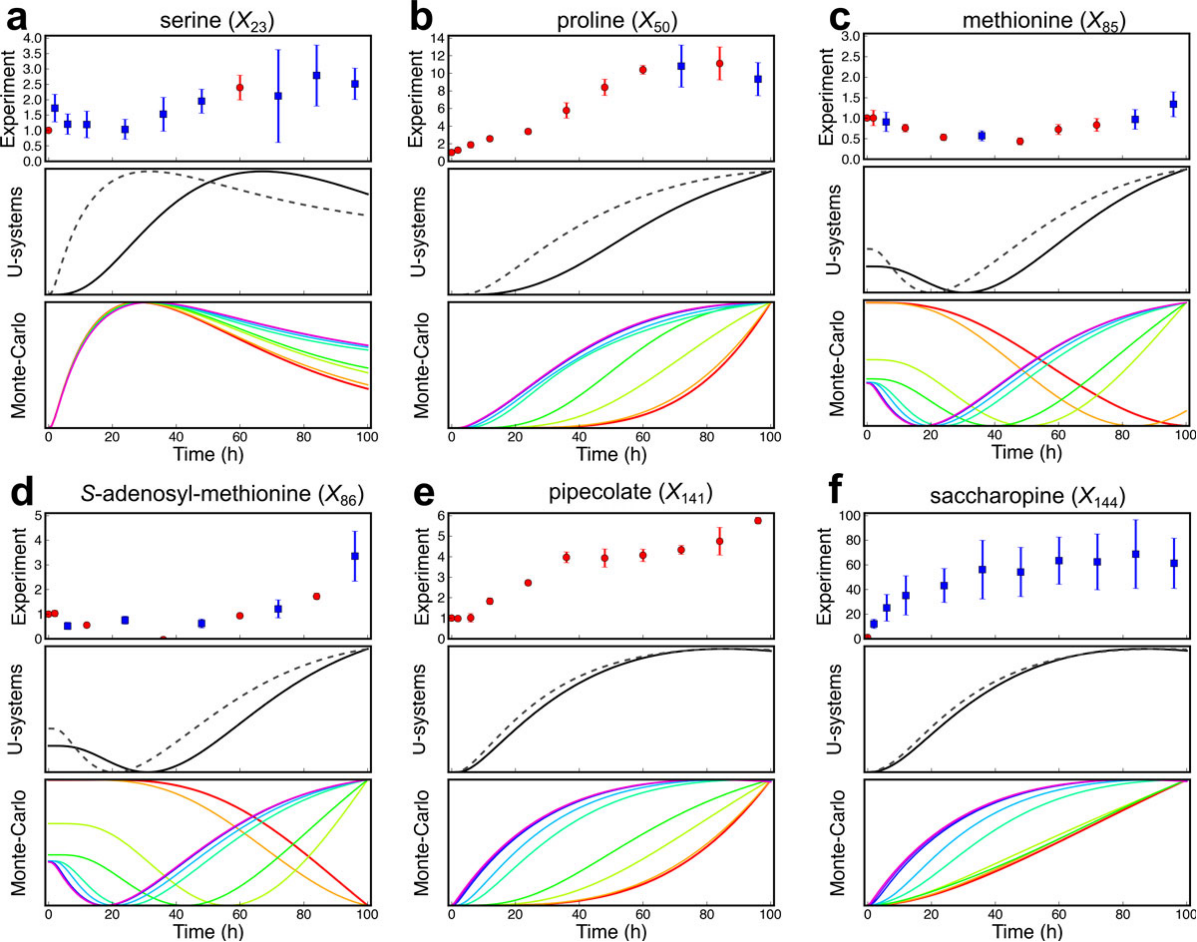
**Comparisons of metabolome data with U-system and Monte-Carlo simulations**. Experimental data from metabolome analysis with coefficient of variations less than and more than 20% are represented in red and blue dots, respectively (upper boxes). The U-system and modified U-system simulations for metabolic system of *Arabidopsis *are represented in gray dotted and black lines, respectively (middle boxes), whereas Monte-Carlo simulations with changes of rate constants between branch points around proline biosynthesis, lysine degradation, methionine salvage pathway, aliphatic GSL biosynthesis (yellow zone in Figure 5), in which parameters are 0.005, 0.01, 0.05, 0.1, 0.5, 1, 5, 10, and 50, are represented in rainbow-colored lines varying from red (0.005) to blue (50), respectively (lower boxes). a, serine (*X*_23_); b, proline (*X*_50_); c, methionine (*X*_85_); d, *S*-adenosyl-methionine (*X*_86_); e, pipecolate, (*X*_141_); f, saccharopine (*X*_144_). The results for other metabolites are shown in Supplementary Figures (additional file 3).

Figure 5 shows the time courses of metabolite concentrations from the U-system approach (gray dotted lines) compared with those from metabolome analysis covering 268 metabolites measured by liquid chromatography-mass spectrometry (LC-MS) (red dots) and those with a coefficient of variations more than 20% (blue dots). It is clear that the actual data contained some biological fluctuations and/or analytical errors. The results indicated that simulated metabolic behaviors changed in similar ways to actual metabolic behaviors but in different time-scales. This is because all rate constants were fixed to 1 so that some rate constants may be too large whereas others may be too small compared with the real ones. To prove the hypothesis that it is possible to roughly adjust rate constants, the flux-split ratios at the branched point associated with methionine salvage pathway, aliphatic glucosinolate (GSL) biosynthesis, lysine degradation and proline biosynthesis (yellow zone in Figure 4) were varied. Specifically, the rate constants from these branched points to other branched points were varied at 0.005, 0.01, 0.05, 0.1, 0.5, 1, 5, 10, and 50, respectively, on the basis of Monte-Carlo simulations. Some calculation results satisfactorily agreed with the measured data although there were some differences. The calculation results indicated that simulated concentrations of serine (*X*_23_) (Figure 5a) does not vary whereas those of proline (*X*_50_) (Figure 5b) clearly alter. This is because the rate constants of fluxes nearby serine (*X*_23_) (pink zone in Figure 4) were kept constant whereas those nearby proline (*X*_50_) (yellow zone in Figure 4) were varied. Similarly, with high rate constants (between 0.5 and 50), the simulated concentrations of methionine (*X*_85_) (Figure 5c) and AdoMet (*X*_86_) (Figure 5d) temporarily decreased and increased back, whereas those of pipecolate (*X*_141_) (Figure 5e) and saccharopine (*X*_144_) (Figure 5f) increased and remained constant during the observation period. At the same time, their metabolic responses seemed to slow down with lower values of rate constants, although they would behave in the same manner if longer period of time were observed. These findings implied that it is possible to adjust the time scale of calculated metabolite concentrations in the U-system model by adjusting the rate constants of fluxes at branched points. Thus, rate constants between branched points were roughly adjusted using some of the time course of relative metabolite concentrations for the coarse prediction of the metabolites concentration and this system was named modified U-system. The results for modified U-system (black lines in Figure 5) showed reasonable metabolic behaviors useful for further analysis.

#### Evaluation of the modified U-system approach for metabolic responses in Arabidopsis

**Figure 6:**
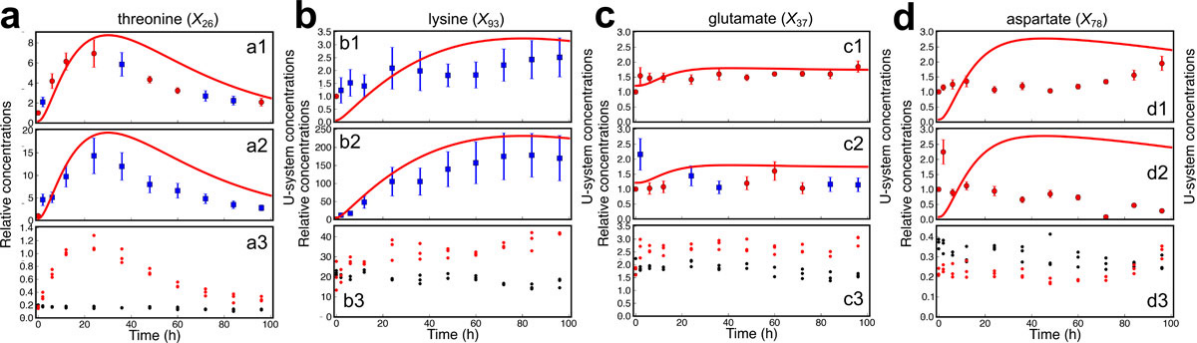
**Simulations from modified U-system approach compared with relative concentrations obtained by metabolome and amino acid analyses**. The red and blue dots represent relative concentrations (see Materials and Methods) of lysine and threonine supplementation experiment with coefficient of variations less than or more than 20%, respectively, which are obtained by metabolome (upper boxes) and amino acid (middle boxes) analyses. Red lines represent simulations from the modified U-system approach. The normalized peak intensities of lysine and threonine-supplemented samples and control samples are shown as red dots and black dots, respectively (lower boxes). a, threonine (*X*_26_); b, lysine (*X*_93_); c, glutamate (*X*_37_); d, aspartate (*X*_78_).

The modified U-system includes various approximations so that it is necessary to validate whether it is practicability and conformable to experimental data. Figure 6 (upper boxes) shows the time courses after lysine and threonine application of the relative concentrations of four amino acids measured in metabolome analysis. The threonine concentration (*X*_26_) exhibited small biological fluctuations among sample replicates, increasing from its steady-state level to a maximum at around 24 h, from where it decreased back to its steady-state level. The lysine concentrations (*X*_93_) contained more biological fluctuations among the sample replications, but it is still possible to detect a clear pattern over time. The glutamate concentration (*X*_37_) increased within 2 h and then seemed to be constant. The aspartate concentration (*X*_78_) increased slightly, then decreased, and finally tended to increase considerably.

The red lines in Figure 6 (a1 and b1) indicate that the simulated concentrations of threonine (*X*_26_) and lysine (*X*_93_) agreed with the metabolic behaviors of experimental data obtained by metabolome analysis with significant correlations (*p*-values < 0.005; detailed in Supplementary Model - additional file 2). However, the simulated concentration of aspartate (*X*_78_) was not consistent with metabolome data (Figure 6d1). It could be that, although the metabolic reaction network used for model construction is correct, the U-system approach might fail to predict the appropriate metabolic behavior. To assess this possibility, a specific measurement only for amino acids using another analytical instrument gas chromatography-mass spectrometry (GC-MS) was performed using the identical samples (Figure 6, middle boxes). The results from amino acid analysis seemed to provide better metabolite trajectories with lower fluctuations. With respect to the concentrations of threonine (X_26_), lysine (X_93_) and glutamate (X_37_), both methods yielded similar results in terms of the metabolic trajectories, although the simulations were in better agreement with the time-courses of metabolite concentrations measured by amino acid analysis. For example, lysine concentration (*X*_93_) measured by metabolome analysis (Figure 6b1) increased and maintained its level from *t*=24 to *t*=60 h before increasing again. The U-system simulation pinpointed that four data at *t*=24, 36, 48 and 60 h obtained by metabolome analysis may contain errors. Actually, the data at *t*=24 and 36 h (shown as blue dots) exhibited high standard deviations. This assumption may be indirectly supported by the results of correlation analysis. For example, the correlation between the U-system simulation and the time-series data obtained by the amino acid analysis (Figure 6b2) is 0.991, which is higher than the correlation of the value, 0.881, between the U-system simulation and the data obtained by metabolome analysis.The result indicates that if a relatively reliable metabolic reaction network diagram is available, the prediction of time-dependent changes in metabolite concentrations using the U-system is beneficial for judging the likely correctness of data measured by metabolome analysis (see, *e.g*., green dots in Figure 1a), which is designed to provide massive datasets of metabolite concentrations sometimes at the expense of absolute concentration accuracies for all metabolites. By contrast, if the predicted behaviors are significantly different in some of the metabolites (see, *e.g*., blue dots in Figure 1a), there may be an unknown metabolic pathway associated with these metabolites or an unidentified regulatory signal. Thus, one may attempt to test a possible system by modifying a pathway or regulation, which can provide a consistent result to predict an unknown candidate before performing a next experiment.

As mentioned above, the relative concentrations observed for several amino acids such as aspartate and glutamate were scattered mainly because of biological variability and possibly due to analytical limitations, and it was difficult to precisely capture the time courses of their concentrations (Figure 6c and 6d). In fact, significant fluctuations in actual metabolite concentrations were observed even in the control samples without lysine and threonine supplementation (Figure 6c3 and 6d3, black dots). However, the computational results suggested that when lysine and threonine were applied as supplements, the aspartate concentration (*X*_78_) slightly increased and then decreased to its initial concentration level or steady-state (Figure 6d1 and 2). This estimation can be supported by the previous experiment related to aspartate kinase (AK) [11]. AK is the first committed enzyme of aspartate-family amino acid biosynthesis and regulated by the feedback inhibition by lysine and threonine (Figure 4). The flux analysis in *Lemna *revealed that the deregulation of AK, *i.e*., the increase of AK activity, caused accumulation of lysine, threonine, methionine, and AdoMet and decrease in the aspartate concentration [11]. Thus, in our study, a reduction in the AK activity through the feedback inhibition by supplementation of lysine and threonine might lead to an initial increase in the aspartate concentration as predicted in modified U-system. The transient increases in glutamate and aspartate concentrations (*X*_37 _and *X*_78_, respectively) after lysine and threonine supplementation were also indirectly supported by the increase of proline (*X*_50_) observed both in simulation and metabolome analysis (Figure 5b). One could assume that if the simulated behaviors of dead-end metabolite concentrations (proline (*X*_50_) in this case) agree well with the experimental data, those of intermediate metabolites (glutamate (*X*_37_) and aspartate (*X*_78_) in this case) are likely to be correct. This consistency implies that the prediction of metabolic behavior by our approach is reliable even if the quality of the experimental data for some metabolites is not very high.

#### Prediction of dynamic behaviors of unmeasurable metabolite concentrations

The 86 out of 268 metabolite concentrations measured by metabolome analysis (purple and blue boxes in Figure 4) were included in the U-system model. The 66 out of 86 metabolite concentrations (blue boxes in Figure 4) showed unclear dynamic patterns changing throughout the period of 96 h after the supplementation of lysine and threonine (Supplementary Figures - additional file 3). One may note that these metabolite concentrations showing no significant variations do not change or change only slightly to the extent that cannot be experimentally observed due to biological fluctuations and analytical constraints. Thus, although a large number of metabolite concentrations in metabolic pathways can be simultaneously measured, the metabolic behaviors assumed from measured metabolite concentrations may sometimes not be entirely reliable. Also, the 265 out of 351 metabolites incorporated in the U-system model could not be detected by this metabolome analysis. Given a computational model, we may be able to predict the dynamic responses of metabolites that cannot be measured reliably nor detected at all. The computational results also have a potential to provide some types of theoretical validation on the accuracy of the experimental data.

**Figure 7:**
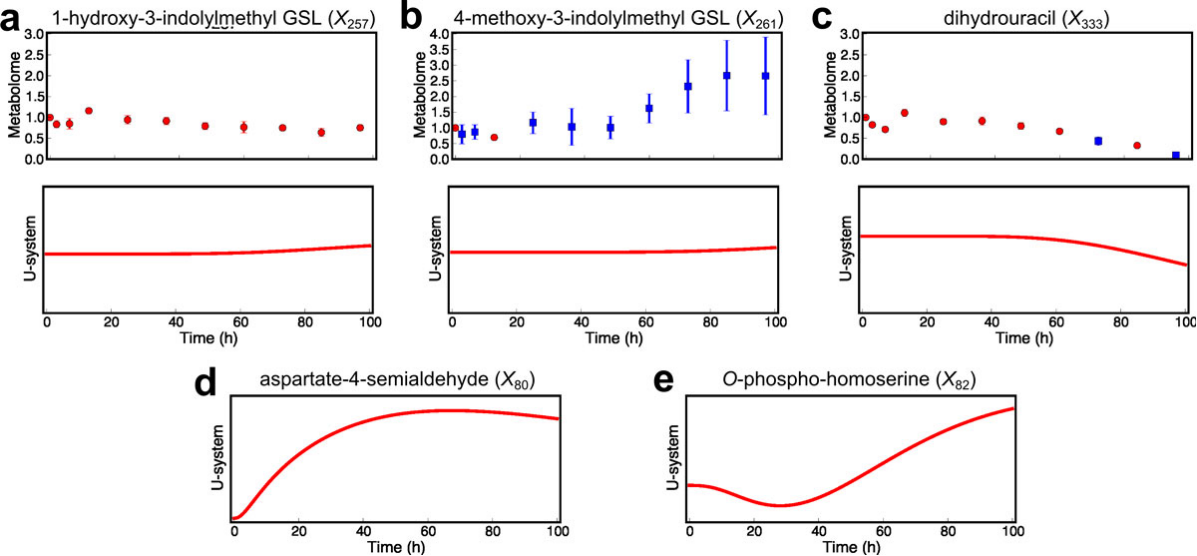
**Predictive simulations from modified U-system approach compared with relative concentrations obtained by metabolome analysis**. The red and blue dots represent relative concentrations (see Materials and Methods) of lysine and threonine supplementation experiment with coefficient of variations less than or more than 20%, respectively. Red lines represent simulations from the modified U-system approach. a Simulation compared with relative concentration of 1-hydroxy-3-indolylmethyl GSL (*X*_257_). b Simulation compared with relative concentration of 4-methoxy-3-indolylmethyl GSL (*X*_261_). c Simulation compared with relative concentration of dihydrouracil (*X*_333_). d Simulation for aspartate-4-semialdehyde (*X*_80_). e Simulation for *O*-phospho-homoserine (*X*_82_).

Figure 7 illustrates that the relative concentration of 1-hydroxy-3-indolylmethyl GSL (*X*_257_) measured by metabolome analysis (Figure 7a) did not change throughout the period of 96 h whereas that of 4-methoxy-3-indolylmethyl GSL (*X*_261_) (Figure 7b) started increasing at *t*=60 h. Interestingly, in the U-system simulations 1-hydroxy-3-indolylmethyl GSL concentration (*X*_257_) started increasing at around *t*=55 h and then 4-methoxy-3-indolylmethyl GSL concentration (*X*_261_) increased around *t*=60 h. In this case, the dynamic behaviors of 1-hydroxy-3-indolylmethyl GSL (*X*_257_) from model prediction were inconsistent with the relative concentrations obtained by metabolome analysis. In the metabolic map, the reactions between *X*_257 _and *X*_261 _are linearly connected without any regulations, *i.e*., inhibition and activation (green zone in Figure 4) so that *X*_257 _and *X*_261 _are expected to behave in the similar manner as observed in the predictive simulations. This inconsistency between actual data and computational prediction suggests two possibilities; that is, an unknown regulatory mechanism may exist or 1-hydroxy-3-indolylmethyl GSL concentration (*X*_257_) might have not be reliably analyzed.

**Figure 8:**
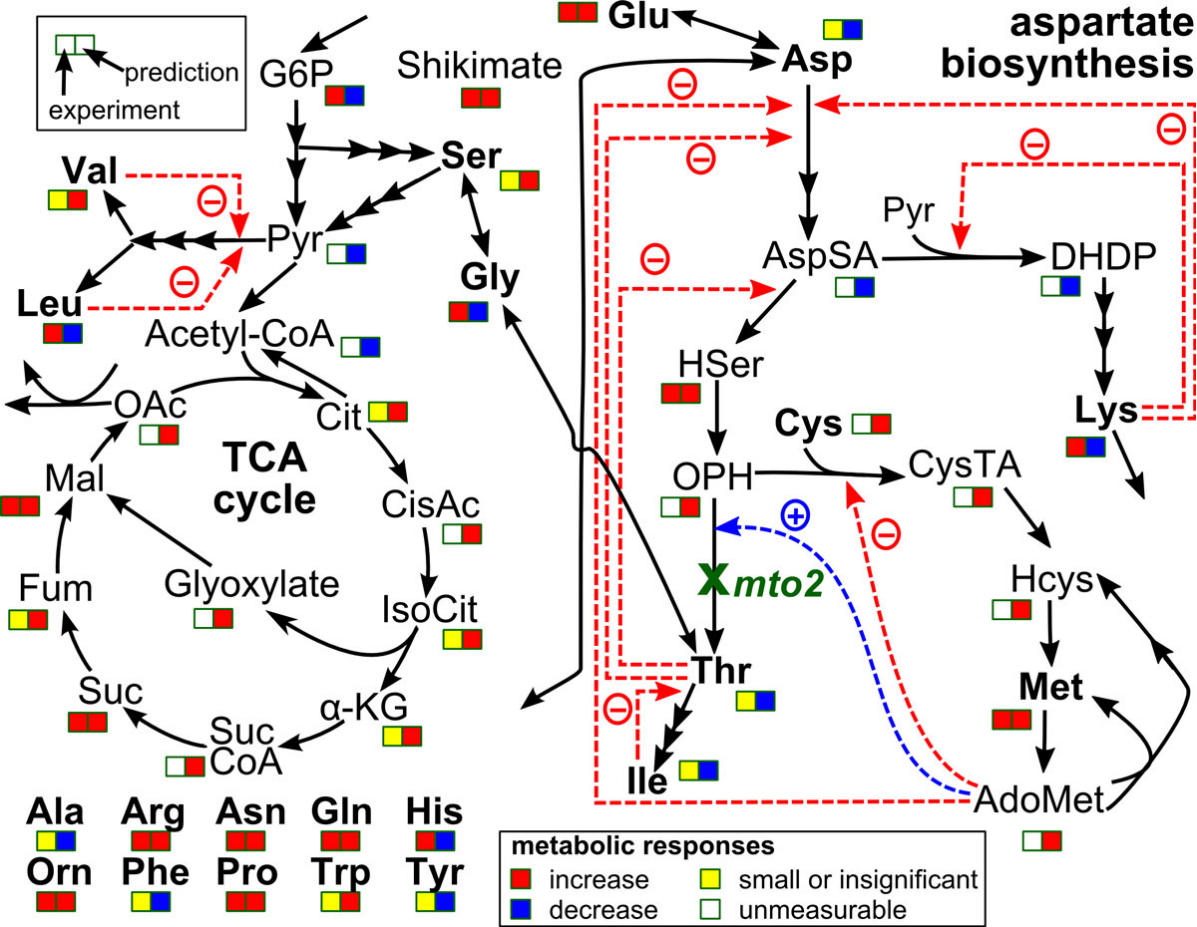
**Qualitative predictions of consequences of gene knockdown**. The simplified aspartate-family biosynthesis pathway enlarged from Figure 4. Metabolites (AdoMet, *S*-adenosyl-methionine; Ala, alanine; Arg, arginine; Asn, asparagine; Asp, aspartate; AspSA, aspartate-semialdehyde; Cit, citrate; CisAc, cis-aconitate; Cys, cysteine; CysTA, cystathionine; DHDP, dihydrodipicolinate; Fum, fumalate; Gln, glutamine; Glu, glutamate; Gly, glycine; G6P, glucose-6-phosphate; Hcys, homocysteine; HSer, homoserine; His, histidine; Ile, iso-leucine; IsoCit, iso-citrate; α-KG, α-ketoglutarate; Leu, leucine; Lys, lysine; Mal, malate; Met, methionine; OAc, oxaloacetate; OPH, *O*-phosphohomoserine; Orn, ornitine; Phe, phenylalanine; Pro, proline; Pyr, pyruvate; Ser, serine; Suc, succinate; SucCoA, succinyl CoA; Thr, threonine; Trp, tryptophan; Tyr, tyrosine; Val, valine) are represented in black letters whereas the knocked-down gene (*mto2, MTO2 *encoding threonine synthase) is represented in green letters. Two squares boxes indicate the comparisons between experimental results (the former) and *in silico *prediction (the latter).

Moreover, we were able to predict the behaviors of concentrations of aspartate-4-semialdehyde (*X*_80_) (Figure 7d) and *O*-phospho-homoserine (*X*_82_) (Figure 7e), which can hardly be detected due to technical limitations. They are not only located at important branched points in the metabolic map but also related to many regulations including both inhibitions and activations (Figure 8). Thus, the information of these metabolites could allow us to comprehend metabolic system. Again, the aspartate-family amino acid biosynthesis includes various inhibitions and activations, so that these dynamic behaviors will not be reasonably observed by normal mass action equations.

#### Prediction of "unpredictable" metabolic behaviors

The modified U-system enables us to mathematically model a relatively large-scale metabolic system, and hence simulations based on the modified U-system provide predictive dynamic behaviors of metabolite concentrations which are hardly provided by existing methods. The modified U-system model for the *Arabidopsis *metabolic system predicted that the supplementation of lysine and threonine did not affect 4-methoxy-3-indolylmethyl GSL concentration (*X*_261_) until *t*=60 h (Figure 7b), nor dihydrouracil concentration (*X*_333_) in pyrimidine biosynthesis until *t*=40 h (Figure 7c). These results suggested that the supplementation of lysine and threonine influenced *X*_261 _and *X*_333 _in a delayed fashion due to metabolic flow passing through various reactions in long and complicated pathways (see Figure 4). The U-system predictions of the time-transient behaviors of metabolite concentrations were reasonable and this kind of metabolic relationship between distant metabolites on metabolic map cannot be examined by either statistical methods or typical mathematical modeling without delay functions. Furthermore, similar predictive simulations of several metabolite located in long and complicated pathways such as xanthine (X_299_) and ureidoproprionate (X_344_) in purine and pyrimidine biosynthesis (gray zone in Figure 4), and aminoadipate (X_153_), glutarate (X_152_), and pipecolate (X_141_) in lysine degradation (yellow zone in Figure 4) reasonably agreed with the experimental results (Supplementary Figures - additional file 3). The results could be an example for a large-scale system to indirectly validate the U-system approach. They also imply that predicted behaviors of unmeasurable metabolites located between detectable metabolites are probably correct, at least, qualitatively.

#### Qualitative predictions of consequences of gene modifications

A challenge in systems biology, in addition to the prediction of metabolic behaviors, is the prediction of metabolic responses of an altered network structure, in which, for instance, gene expression or enzyme activities are modified. To assess whether the modified U-system model is applicable for this kind of prediction, an *in silico *knockdown for *MTO2 *encoding threonine synthase gene of *Arabidopsis *in the biosynthetic pathway of aspartate-family amino acids (Figure 8 enlarged from Figure 4). When the flux controlled by *MTO2 *was 90% reduced *in silico*, the threonine concentration was predicted to decrease whereas the methionine concentration was predicted to increase. In overall, the 6 out of 7 amino acids or 85% prediction agreed with the original result from biological experiment using an *Arabidopsis mto2 *mutant [17]. The prediction was further compared to the result from metabolome analysis [18]. Figure 8 showed that 73% correctness from 11 metabolites showing the significant changes comparing with the wide-type whereas the metabolome and amino acid data showed only 50% agreement among 6 amino acids. The incorrect prediction might be because of the insufficient information for model input which could be considered to be an unidentified pathway. Nevertheless, changes of all unmeasurable metabolites can also be qualitatively predicted using our model. The result suggests that the U-system approach is capable of predicting metabolic levels when the system is modified, even if specific information on complicated regulatory mechanisms is not available. This information can aid the design of next-step experiments.

## Conclusions

The U-system approach is simple, flexible and can be practically exploited to predict the coarse time-transient behavior of metabolites in a large-scale metabolic system. It is also capable of predicting metabolic behaviors when the system is modified. The U-system approach has two major characteristics: 1) the U-system model is presented in a normalized form, and 2) the U-system model constructed only using partial, currently-available metabolic pathways. Recent advances in metabolomics technology have enabled us to obtain large-scale semi-quantitative datasets for hundreds of metabolite concentrations. The first characteristic is essential to establish mathematical models for actual metabolic systems consisting of hundreds to thousands of metabolites, whose concentrations cannot be measured practically by existing methods of measurement other than metabolomics. The second characteristic is desirable for the coarse characterization of complicated systems, because metabolic responses mainly depend on the network structure, rather than parameter values, as we have shown in this study. Thus, the U-system approach is not only informatively useful for data analysis and model diagnostics but it also can aid the design of next-step experiments.

## Methods

### Background on Biochemical Systems Theory (BST)

#### 1. Structure of equations

BST uses ordinary differential equation systems of a specific format: Each process is represented with a product of power-law functions. This format is the result of Taylor approximation in a logarithmic space [4,9,19,20]. The change in each variable of a system is thus represented as a so-called Generalized Mass Action (GMA)-system as follows:

(5) $$\frac{dX_{i}}{dt}-\overset{p}{\underset{k=1}{\sum }}\alpha_{ik}\overset{n}{\underset{j=1}{\prod }}{X_{j}}^{g_{ijk}}\overset{m}{\underset{j=1}{\prod }}{Y_{j}}^{G_{ijk}}-\overset{q}{\underset{k=1}{\sum }}\beta_{ik}\overset{n}{\underset{j=1}{\prod }}{X_{j}}^{h_{ijk}}\overset{m}{\underset{j=1}{\prod }}{Y_{j}}^{H_{ijk}}\;\;\;i=1,2,...,n$$

Here, *X_i _*(*i*=1,..., *n*) are dependent variables (typically metabolite concentrations), *Y_j _*(*j*=1,..., *m*) are independent variables (typically external substrates or enzyme activities), α*_ik _*(*i*=1,..., *n; k*=1,..., *p*) and β*_ik _*(*i*=1,..., *n; k*=1,..., *q*) are the non-negative rate constants for influxes and effluxes, respectively, *g_ijk _*(*i*=1,..., *n; j*=1,..., *m; k*=1,..., *p*) and *h_ijk _*(*i*=1,..., *n; j*=1,..., *m; k*=1,..., *q*) are real-valued kinetic orders associated with dependent variables in influxes and effluxes, respectively, while *G_ijk _*(*i*=1,..., *n; j*=1,..., *m; k*=1,..., *p*) and *H_ijk _*(*i*=1,..., *n; j*=1,..., *m; k*=1,..., *q*) are those associated with independent variables. *n *and *m *are the numbers of dependent and independent variables, respectively, *p *and *q *are the maximum numbers of influxes and effluxes, respectively, and *t *is time. Details have been documented in the literature many times, *e.g*. in [6,7,9,21].

#### 2. Validity and accuracy of BST for modeling biological systems

Since the power-law format in BST appears to be quite restrictive, the accuracy of different model variants within BST has been assessed many times. As Taylor approximations, the models are mathematically guaranteed to be correct at an operating point of choice, but their ranges of validity were also tested against results from more traditional formulations like the Michaelis-Menten formalism. These comparisons demonstrated sufficient accuracy of BST models over quite large ranges in variation of the involved variables [22-25]. Applications of BST models were also demonstrated with various large-scale systems, including the TCA cycle [26], ethanol fermentation [27], purine metabolism [28], and sphingolipid metabolism [29].

### Meaning of parameters and U-system approach

Model parameters of pathway systems are usually estimated from kinetic information or from metabolic time series data. In Eq. (5), the familiar kinetic parameters involved in Michaelis-Menten kinetics (maximum reaction rate *V*_max_, Michaelis constant *K*_M_, etc.) are not explicitly visible, but they are instead distributed to the rate constants and kinetic orders. The BST parameters have their own meaning either as turn-over rates or as the specific effect that a variable has on the flux term in which it is involved. Thus, if a kinetic order is negative, the variable with which it is associated has a negative (inhibitory) effect on the flux term. This effect is given exclusively by the one appropriate kinetic order, thereby reducing the number of kinetic parameters in the system, which can be much higher in traditional rate functions. Still, the simplified BST parameters are able to capture time-transient behaviors of metabolite concentrations and other characteristics of metabolic reaction systems very well.

A consequence of the direct meaning of kinetic orders in BST is that it is possible to set up differential equations in symbolic notation, solely based on the structure of a metabolic pathway and its regulatory structure, whereas deep knowledge of their regulatory mechanisms is not needed. Furthermore, the kinetic parameters in Eq. (5) can be estimated directly from time-series data of metabolite concentrations, and subsequently convey important information about the system characteristics. In some cases, traditional kinetic parameters from the literature, such as *V*_max_, can be converted into BST parameters, but this is seldom possible on the basis of metabolome data, which are often presented as relative rather than absolute concentrations.

It was recently shown [30] that a coarse grid of kinetic orders is sufficient to fit time developments in pathway systems with surprisingly high accuracy. Expanding on this idea, we here fix all kinetic orders to 1, 0.5 or -0.5 to represent enzyme kinetics, positive or negative effects of metabolites, respectively. In other words, we use a hybrid between a pure mass action system and a GMA system, which allows for inhibitory and activating effects that are modeled with kinetic orders of -0.5 and 0.5. The rate constants are at first also set to 1, but may be later subjected to order-of-magnitude adjustments, which obviously improve the model representation of the data. Thus, at branched points, the equations are adjusted based on stoichiometry. For example, in a branched reaction of the type shown in Figure 1c the X_1 _is converted into X_2 _and X_4_, so that the equation is d*X*_1_/d*t *= V_inX1 _- V_outX1-1 _- V_outX1-2_. Since most of the parameters are set at unity in the proposed approach, we name this system as Unity-system or U-system.

Fixing the parameters values is to some degree compensated by adjustments in metabolite concentrations. In other words, if the kinetic orders are forced to be 1, +0.5, or -0.5, a data fit will lead to adjusted metabolite concentrations.

### Monte-Carlo simulations

Monte-Carlo simulations were employed to test in an unbiased manner whether the qualitative behavior of a model is consistent with the U-system representation. Most Monte-Carlo simulations were calculated for 1,000 loops. The parameter values were generated randomly from uniform distributions. The ranges for rate constants and kinetic orders were between 0.5 and 20, and ±0.2 and ±0.8, respectively.

### Calculation method

Differential equations were solved using automatic switching for stiff and non-stiff problems (LSODA) [31] using the ODE integration package from SciPy

(Scientific Tools for Python) [32]. All visualizations were created using Matplotlib [33].

### Details of the *Arabidopsis *system

#### Arabidopsis callus culture

*Arabidopsis thaliana *liquid callus culture derived from accession Col-0 was prepared as described in Murota et al. [34] with slight modifications. For callus induction, minced seedlings were incubated in RM28 medium under constant light. The medium was changed every 6 days. For a metabolic perturbation experiment, RM28 medium supplemented with 10 mM L-lysine and 1 mM L-threonine [35] was used at the third medium change. For a control experiment, RM28 without supplementation was used. Sucrose in RM28 medium was a sole carbon source for callus culture. The experiments were carried out in triplicate.

For both metabolome and amino acid analyses, calli were collected prior to lysine and threonine treatment (0 h), and 2, 6, 12, 24, 36, 48, 60, 72, 84 and 96 h after the treatment. The calli were immediately frozen in liquid nitrogen and stored at -80°C. Prior to analyses, the frozen samples were lyophilized using a freeze dryer (FDU-2100, EYELA) in a vacuum.

#### Metabolome analysis

Metabolites were extracted by homogenizing lyophilized callus in 500 uL 80% methanol in 0.1% formic acid per 2 mg dry weight callus with 5 mm zirconia beads (no.5-4060-13, AS ONE Co. Ltd.) in 2.0 mL sampling tubes (no.132-620C, WATSON Co., LTD) for 5 min using shake master NEO (Bio Medical Science, Tokyo, Japan). After centrifugation using a high speed refrigerated micro centrifuge (TOMY MX-300) at 14,000 r.p.m. at 4°C, 250 uL supernatant was dried up in 96 well plate and the residue was dissolved in 120 uL ultrapure water (no. 210-01303, Wako Pure Chemical Industries, Ltd.). One uL of the solution was subjected to widely targeted metabolome analysis [36] by LC-MS using UPLC-TQD system (Waters, Milford, MA, USA). The peak intensities were normalized by those of internal standards (10-campher sulfonate and lidocaine). For mathematical modeling, the normalized peak intensities of treated samples were subtracted from those of control samples to eliminate effects of cell growth and obtain relative metabolite concentrations.

#### Amino acid analysis

Metabolites were extracted by homogenizing lyophilized callus in 500 uL of the extraction solution (methanol: milliQ water = 4:1) per 2 mg dry weight in 2.0 mL sampling tubes using Mixer Mill MM300. After centrifugation at 15,000 r.p.m. at 25°C for 10 min, the 400 uL supernatant was used for the analysis performed according to the protocol based on the EZ:faast amino acid derivatization technique for GC-MS (Phenomenex, Torrance, CA) with slight modification. The reagent 1 (internal standard solution) was diluted to 10-fold while the reagent 6 (re-dissolution solvent) was added only 50uL to concentrate amino acid concentrations in samples. Then, 1 uL of solution was subjected to amino acid analysis by GC-MS using GCMS-QP2010 Plus (Shimadzu, Kyoto, Japan).

#### Statistical evaluation

The correlation coefficients, testing for the significance of the correlation coefficients, and student's *t *continuous random variable based on a survival function were calculated for comparing model simulations with experimental data.

#### Arabiodopsis callus model

Information for metabolic pathways including their inhibition and activation was assembled from KEGG (http://www.genome.jp/kegg/) [37,38] and AraCyc (http://arabidopsis.org/tools/aracyc/) [39] databases. A mathematical model was constructed based on the metabolic reaction network including 351 metabolites and 441 fluxes, which cover a wide variety of metabolic pathways related to amino acid biosynthesis, glycolysis pathway, TCA cycle, pentose phosphate pathway, GSL biosynthesis, and purine and pyrimidine biosynthesis (Figure 4; Supplementary Model - additional file 2). The relative concentrations of 86 metabolites among 268 analyzed in metabolome analysis were included in the model. Twenty (square purple boxes in Figure 4) out of the 86 metabolite concentrations included in the model (23.2%) showed considerable changes.

The present study employs a GMA-system model to which the U-system approach was applied for characterizing metabolic behaviors in a large-scale metabolic reaction network related to aspartate-derived amino acids, namely, aspartate, lysine, threonine, and methionine. The effects of feedback inhibition and activation were also included based on information available from the literature and pertinent databases [12,40]. Kinetic orders of metabolites were set to 1, while kinetic orders for inhibition and activation signals were set to -0.5 and 0.5, respectively. In the experiments to obtain relative metabolite concentrations, lysine and threonine was supplemented to the culture medium and transported into the callus cells. Then Fick's law of diffusion [16] was included in the equations for the supplemented metabolites lysine and threonine. Also, their parameters were appropriately fitted with available data of Lys and Thr obtained by metabolome analysis. Metabolite concentrations at the steady state were calculated by simultaneously solving the ordinary differential equations for all 351 metabolites (Figure 4). Since these concentrations were optimized according to the mass balance of the system, the magnitude of the concentration values become smaller as the location of metabolites on the metabolic map were farther away from glucose, which was the main initial metabolite in the present study. It is noted that time-series metabolome data from the experiment were given in relative rather than absolute concentrations. Thus, it is reasonable to take into account the magnitudes of metabolite concentrations while considering predicted behaviors of the metabolite concentrations.

## Competing interests

The authors declare that they have no competing interests.

## Authors' contributions

SN and MYH conceived the experimental concepts and designed callus culture experiment. KS, EOV, FS and MYH conceived the theoretical concepts and designed modeling approach. YC, YY and HO planned and performed callus culture experiments. YS conducted metabolome analysis by LC-MS. KS conducted amino acid analysis by GC-MS, and performed modeling and simulations. KS, TF, SN, EOV, FS and MYH performed data analyses. KS, SK, HO, TF, SN, EOV, FS and MYH performed data interpretation. KS, YS, SN, EOV, FS, MYH wrote and edited the manuscript. All authors read and approved the final manuscript.

## Supplementary Material

Additional file 1 Usystem_SupplementaryInformation.pdf. Additional figures for the branched pathway model. Figure S1. Linear pathway with inhibition (⊖). *X*_1 _and *X*_2 _represent metabolites whereas *Y*_1 _and *Y*_2 _represent enzyme activities for the *X*_1 _influx and *X*_2 _efflux, respectively. Figure S2. Comparisons of time courses of *X*_1 _and *X*_2 _obtained by the Michaelis-Menten model and those by the simplified U-system approach of the corresponding GMA model. The x-axis represents time, while the y-axis represents the concentrations of *X_i_*. The red lines pertain to the actual system in left and bottom axes whereas the blue dotted lines are for U-system in right and top axes. a The *X*_1 _U-system concentration compared with real concentration when the values of *X*_1 _was increased two-fold at *t*=0. b The *X*_2 _U-system concentration compared with real concentration when the values of *X*_1 _was increased two-fold at *t*=0. c The *X*_1 _U-system concentration compared with real concentration when the values of *X*_2 _was increased two-fold at *t*=0. d The *X*_2 _U-system concentration compared with real concentration when the values of *X*_2 _was increased two-fold at *t*=0. Figure S3. Normalized concentrations of *X_i _*with variations of parameters *α_i _*which value from 0.5 (red), 1, 5, 10, 15, 20, 25, 50, 75 to 100 (blue). Figure S4. Normalized concentrations of *X_i _*with variations of parameters *β_i _*which value from 0.5 (red), 1, 5, 10, 15, 20, 25, 50, 75 to 100 (blue). Figure S5. Normalized concentrations of *X_i _*with variations of parameters *g_ij _*which range from 0.1 (or -0.1; red) to 1.0 (or -1.0; blue). Figure S6. Normalized concentrations of *X_i _*with variations of parameters *h_ij _*which range from 0.1 (red) to 1.0 (blue). Click here for file

Additional file 2 Usystem_SupplementaryModel.pdf. The details for *Arabidopsis *model. Click here for file

Additional file 3 Usystem_SupplementaryFigures.pdf. The U-system simulations comparing with experimental data from metabolome and amino acids analysis. Click here for file

## Declarations

Publication charges for this article were funded by KAKENHI grant number 25660084.

This article has been published as part of *BMC systems Biology *Volume 8 Supplement 5, 2014: Proceedings of the 25th International Conference on Genome Informatics (GIW/ISCB-Asia): Systems Biology. The full contents of the supplement are available online at http://www.biomedcentral.com/bmcsystbiol/supplements/8/S5.
