# Supplementary material for: A U-system approach for predicting metabolic behaviors and responses based on an alleged metabolic reaction network
Source: BMC Syst Biol. 2014 Dec 12;8(Suppl 5):S4. doi: 10.1186/1752-0509-8-S5-S4 (PMC4305983; doi:10.1186/1752-0509-8-S5-S4)
Supplement: Additional file 1 — Usystem_SupplementaryInformation.pdf. Additional figures for the branched pathway model. Figure S1. Linear pathway with inhibition (⊖). X1 and X2 represent metabolites whereas Y1 and Y2 represent enzyme activities for the X1 influx and X2 efflux, respectively. Figure S2. Comparisons of time courses of X1 and X2 obtained by the Michaelis-Menten model and those by the simplified U-system approach of the corresponding GMA model. The x-axis represents time, while the y-axis represents the concentrations of Xi. The red lines pertain to the actual system in left and bottom axes whereas the blue dotted lines are for U-system in right and top axes. a The X1 U-system concentration compared with real concentration when the values of X1 was increased two-fold at t=0. b The X2 U-system concentration compared with real concentration when the values of X1 was increased two-fold at t=0. c The X1 U-system concentration compared with real concentration when the values of X2 was increased two-fold at t=0. d The X2 U-system concentration compared with real concentration when the values of X2 was increased two-fold at t=0. Figure S3. Normalized concentrations of Xi with variations of parameters αi which value from 0.5 (red), 1, 5, 10, 15, 20, 25, 50, 75 to 100 (blue). Figure S4. Normalized concentrations of Xi with variations of parameters βi which value from 0.5 (red), 1, 5, 10, 15, 20, 25, 50, 75 to 100 (blue). Figure S5. Normalized concentrations of Xi with variations of parameters gij which range from 0.1 (or -0.1; red) to 1.0 (or -1.0; blue). Figure S6. Normalized concentrations of Xi with variations of parameters hij which range from 0.1 (red) to 1.0 (blue). [file 1752-0509-8-S5-S4-S1.pdf]

# Supplementary Information

Title: A U–system approach for predicting metabolic behaviors and responses based on an alleged metabolic reaction network

Authors: Kansuporn Sriyudthsak, Yuji Sawada, Yukako Chiba, Yui Yamashita, Shigehiko Kanaya, Hitoshi Onouchi, Toru Fujiwara, Satoshi Naito, Ebernard O. Voit, Fumihide Shiraishi and Masami Yokota Hirai

## Simple Michaelis-Menten system

A simple model of a linear pathway with inhibition is sketched in Figure S1.

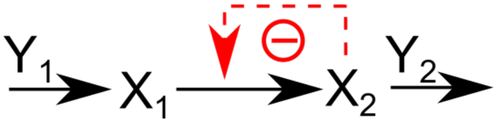


**Figure S1. Linear pathway with inhibition (⊖).** $X_1$ and $X_2$ represent metabolites whereas $Y_1$ and $Y_2$ represent enzyme activities for the $X_1$ influx and $X_2$ efflux, respectively.

Flux expressions and parameters for this pathway were constructed in the typical Michaelis-Menten format where the enzymatic reaction velocity is usually determined by an *in vitro* experiment [6]. The usual Michaelis-Menten model with inhibition is presented in equations (S1) and (S2),

$$\frac{dX_1}{dt} = Y_1 - \frac{V_{\max} X_1}{X_1 + K_m\left(1 + X_2 / K_i\right)} \tag{S1}$$

$$\frac{dX_2}{dt} = \frac{V_{\max} X_1}{X_1 + K_m\left(1 + X_2 / K_i\right)} - Y_2 X_2 \tag{S2}$$

where $V_{\max}$=100.0, $K_m$=10.0, $K_i$=5.0, $Y_1$=5.0, $Y_2$=2.0, and the steady state is $X_1^* = 0.789$, $X_2^* = 2.50$.

The corresponding GMA model with U–system simplifications is presented in equations (S3) and (S4),

$$\frac{dX_1}{dt} = Y_1 - X_1 X_2^{-0.5} \tag{S3}$$

$$\frac{dX_2}{dt} = X_1 X_2^{-0.5} - X_2 \tag{S4}$$

where $Y_1 = 5.0$, $X_1^* = 11.180$, and $X_2^* = 5.0$

The dynamics of the time-varying metabolite concentrations was calculated for the Michaelis-Menten equations and the U–system approach of the GMA model. Specifically, the initial value of either $X_1$ or $X_2$ at $t$=0 was increased to twice the steady-state concentrations and the following trajectories were computed. Figure S2 shows the comparison of calculations from both models. It is clear that the results from both models are numerically quite different but qualitatively comparable. For both scenarios, when $X_1$ was perturbed, $X_2$ increased until it reached its maximum and decreased back to its steady-state, whereas $X_1$ continuously decreased. The results indicate that the qualitative shapes of the trajectories of metabolite concentrations strongly depend on the network structure, but less so on specific parameter values, as it was observed elsewhere [7]. Nonetheless, the metabolite concentrations at each time point and the time-scale of metabolite concentrations are quite different.

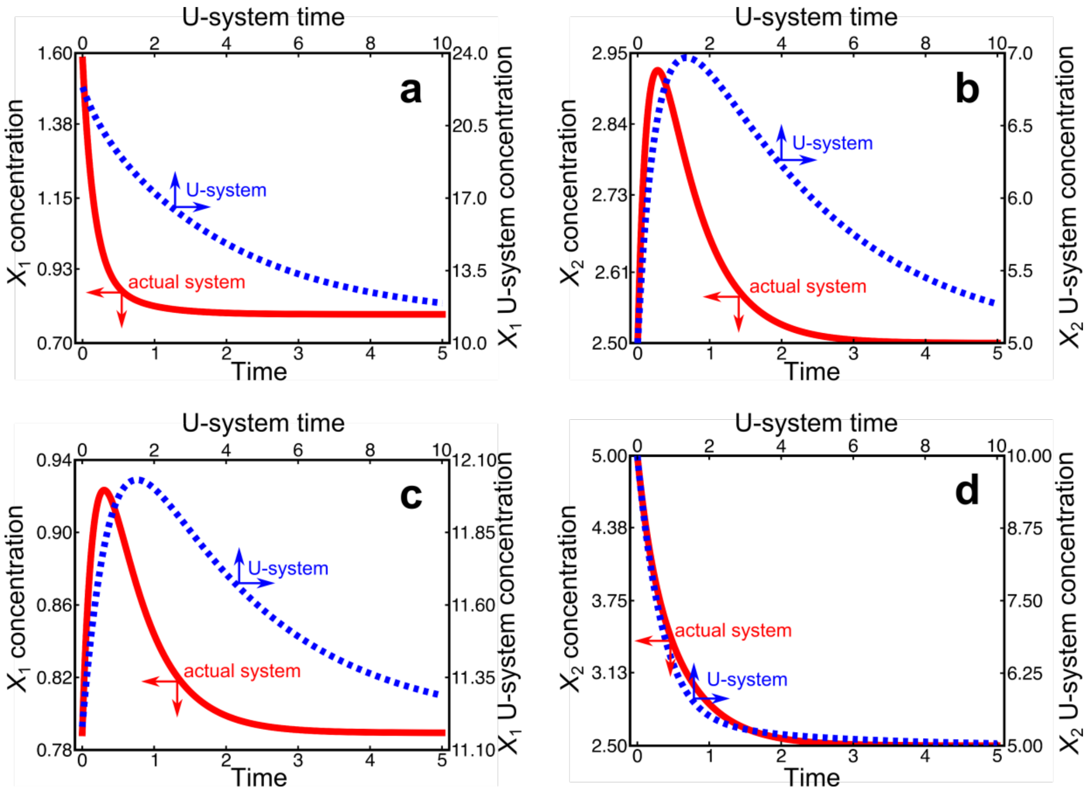


**Figure S2**. **Comparisons of time courses of $X_1$ and $X_2$ obtained by the Michaelis-Menten model and those by the simplified U–system approach of the corresponding GMA model.** The x-axis represents time, while the y-axis represents the concentrations of $X_i$. The red lines pertain to the actual system in left and bottom axes whereas the blue dotted lines are for U–system in right and top axes.

a The $X_1$ U–system concentration compared with real concentration when the values of $X_1$ was increased two-fold at $t$=0.

b The $X_2$ U–system concentration compared with real concentration when the values of $X_1$ was increased two-fold at $t$=0

c The $X_1$ U–system concentration compared with real concentration when the values of $X_2$ was increased two-fold at $t$=0.

d The $X_2$ U–system concentration compared with real concentration when the values of $X_2$ was increased two-fold at $t$=0.

## Branched pathway

These supplements contain additional figures for the branched pathway model while the details for *Arabidopsis* model are provided in Usystem_SupplementaryModel.pdf with the U-system simulations comparing with experimental data from metabolome and amino acids analysis in Usystem_SupplementaryFigures.pdf, as mentioned in the body of the text.

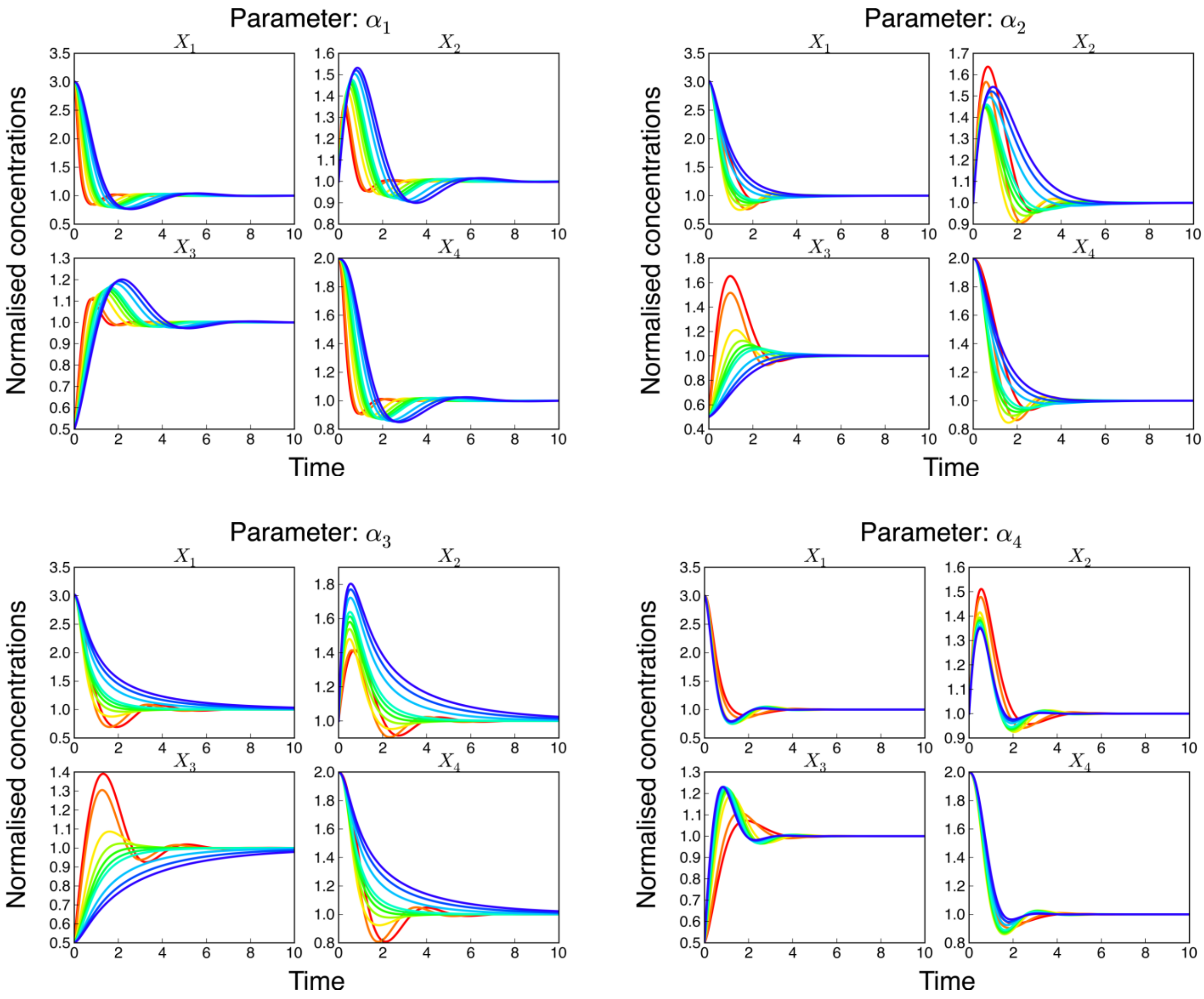


**Figure S3. Normalized concentrations of $X_i$ with variations of parameters $\alpha_i$ which value from 0.5 (red), 1, 5, 10, 15, 20, 25, 50, 75 to 100 (blue)**

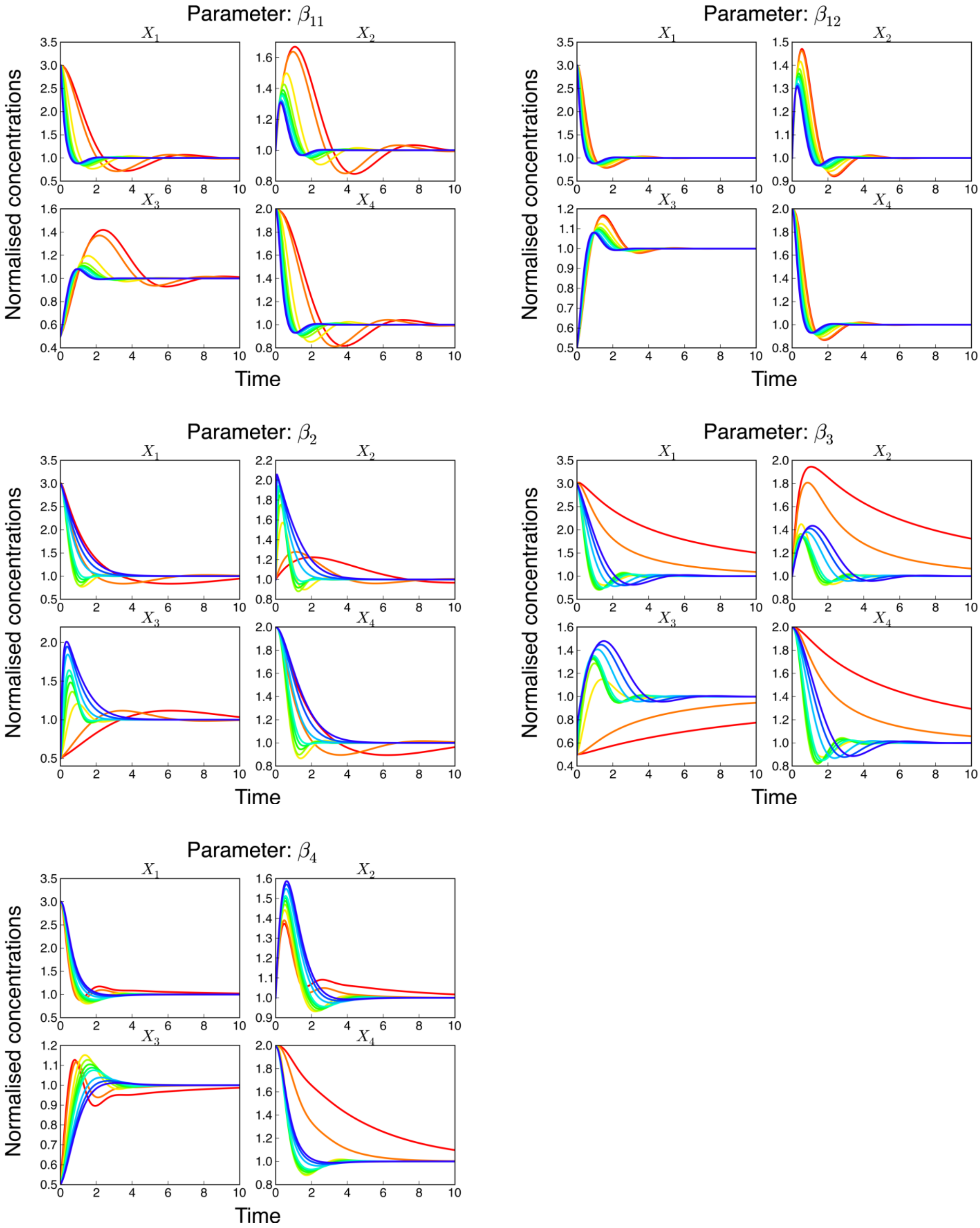


**Figure S4. Normalized concentrations of $X_i$ with variations of parameters $\beta_i$ which value from 0.5 (red), 1, 5, 10, 15, 20, 25, 50, 75 to 100 (blue)**

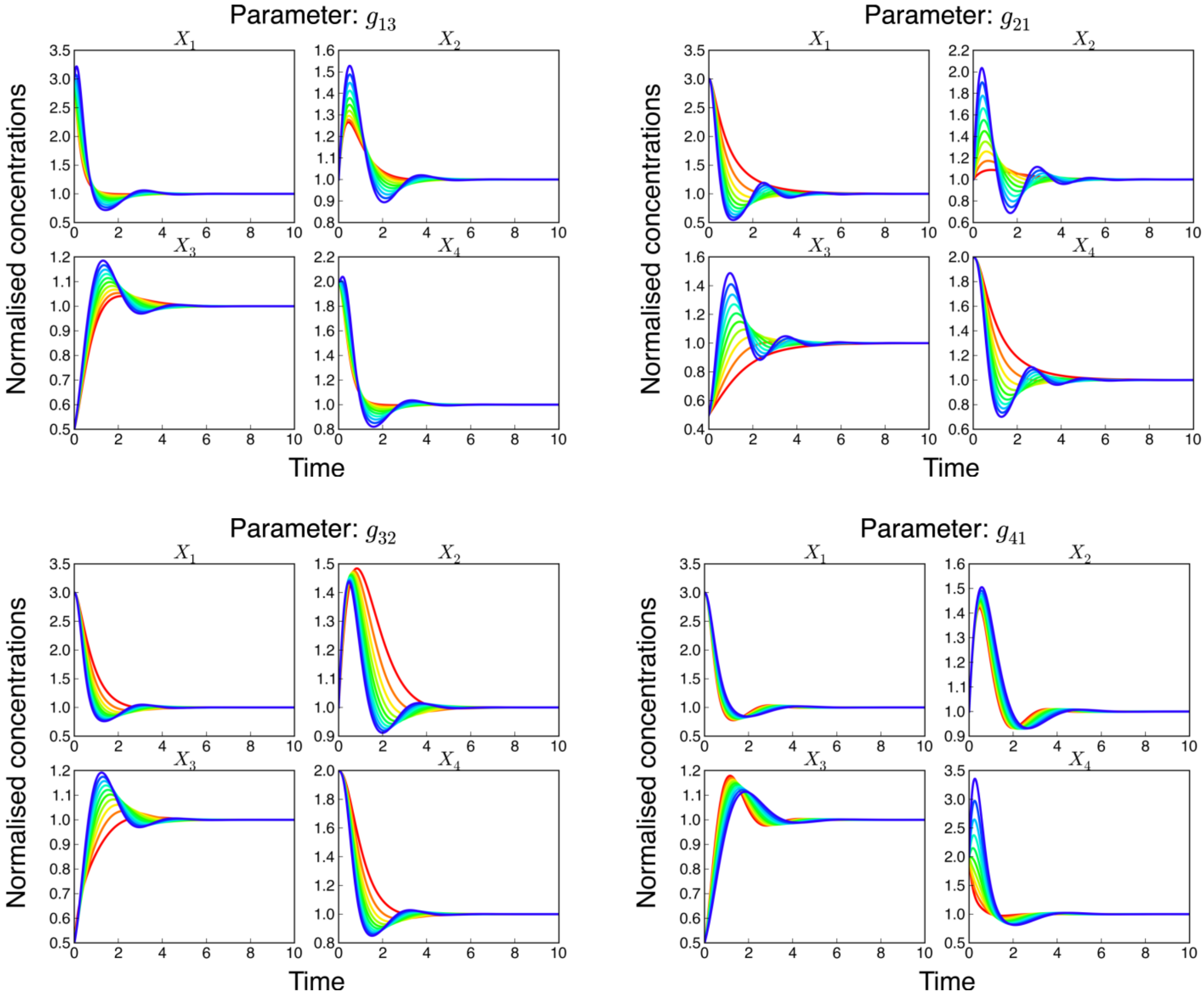


**Figure S5. Normalized concentrations of $X_i$ with variations of parameters $g_{ij}$ which range from 0.1 (or –0.1; red) to 1.0 (or –1.0; blue)**

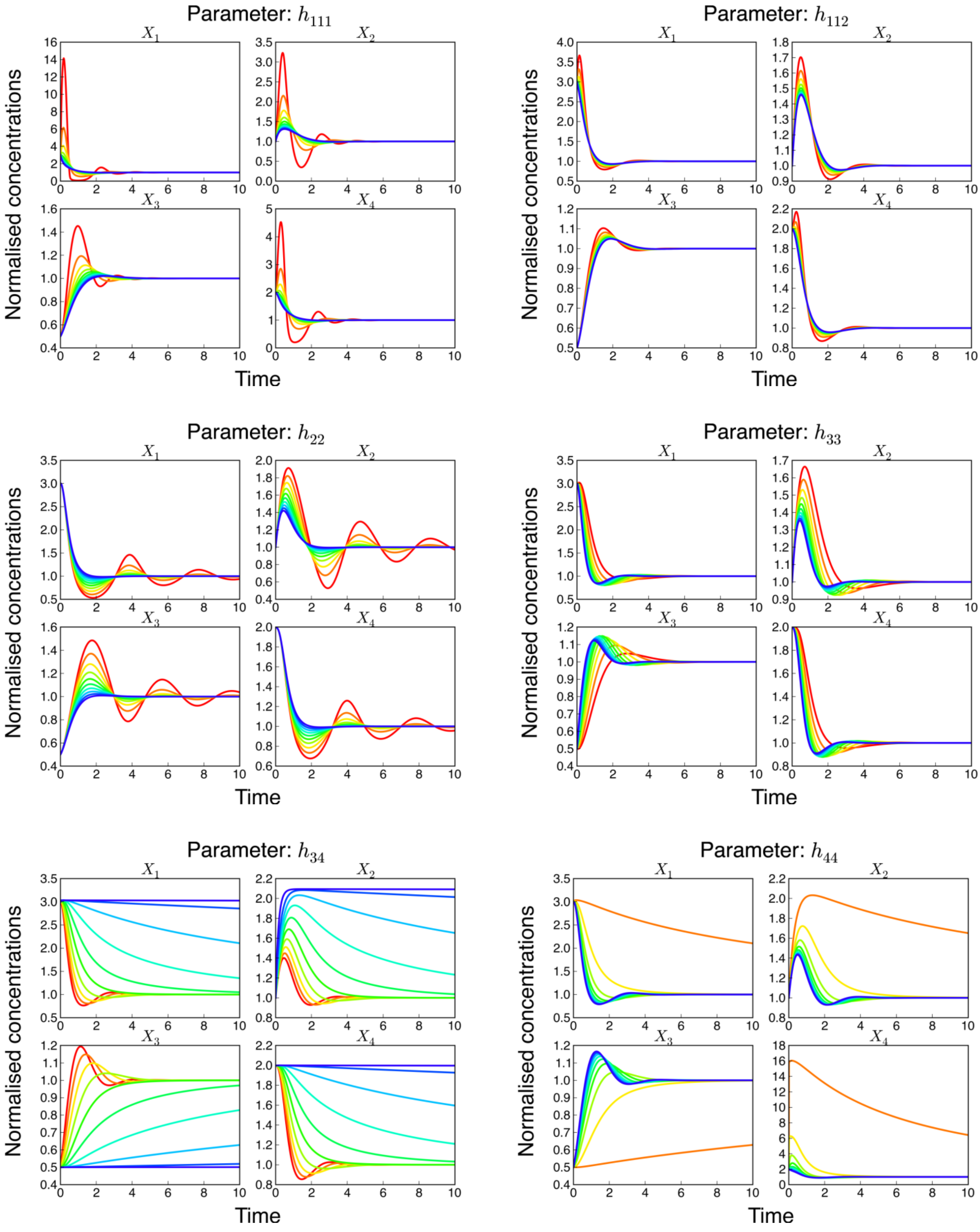


**Figure S6. Normalized concentrations of $X_i$ with variations of parameters $h_{ij}$ which range from 0.1 (red) to 1.0 (blue)**
