# Supplementary material for: A U-system approach for predicting metabolic behaviors and responses based on an alleged metabolic reaction network
Source: BMC Syst Biol. 2014 Dec 12;8(Suppl 5):S4. doi: 10.1186/1752-0509-8-S5-S4 (PMC4305983; doi:10.1186/1752-0509-8-S5-S4)
Supplement: Additional file 2 — Usystem_SupplementaryModel.pdf. The details for Arabidopsis model. [file 1752-0509-8-S5-S4-S2.pdf]

Worksheet	Description
MetaboliteNames	Numbers for metabolites in U-system model arbitrarily given. X_i represent metabolites.
Fluxes	Fluxes for U-system model. V_j represent fluxes.
ODE	Ordinary differential equations (ODE) for the modified U-system model. F_i represent ODE of metabolite *i*. For the original model, a0, a1, a2, a3 are 1.0. For the Monte-Carlo simulations, the value of a3 was varied while other parameters a in the original model were kept to be 1.0. For the modified model, a0=0.05, a1=0.01, a2=0.1 and a3=1.0.
CorrelationAnalysis	Correlation coefficient and p-values for the comparisons of experiemental data and U-system simulation.
	CorrMetabolomeSimulation and pValMetabolomeSimulation refer to correlation coefficients and p-values between relative concentrations from metabolome analysis and U-system concentrations. CorrAminoAcidSimulation and pValAminoAcidSimulation refer to correlation coefficients and p-values between relative concentrations from amino acid analysis and U-system concentrations. CorrMetabolomeAminoAcid and pValMetabolomeAminoAcid refer to correlation coefficients and p-values between relative concentrations from metabolome analysis and those from amino acid analysis. Columns for Simulation, AminoAcidAnalysis and MetabolomeAnalysis represents metabolic names for each analysis system.

The correlation coefficients, testing for the significance of the correlation coefficients, and student's t continuous random variable based on a survival function were calculated for comparing model simulations with experimental data.

$$r = \sqrt{\frac{\left(\sum xy - n\overline{x}\,\overline{y}\right)^2}{\left(\sum x^2 - n\overline{x}^2\right)\left(\sum y^2 - n\overline{y}^2\right)}}$$

$$t = \frac{r}{\sqrt{\frac{\left(1-r^2\right)}{N-2}}}$$

$$F(t) = 1 - S(t)$$

$$p = P(T \geq t) = 1 - F(t)$$

where r represents correlation coefficient while x and y represent experimental and simulated data; t is testing for the significance of the correlation coefficients while p is p-value which is calculated using survival function,

X_1	=	sucrose
X_2	=	glucose-6P
X_3	=	fructose-6P
X_4	=	fructose-1,6-bis-P
X_5	=	glyceraldehyde-3-P
X_6	=	dihydroxyacetone-P
X_7	=	1,3-diPglycerate
X_8	=	3-phosphoglycerate
X_9	=	2-phosphoglycerate
X_{10}	=	phosphoenolpyruvate
X_{11}	=	pyruvate
X_{12}	=	2-acetolactate
X_{13}	=	2,3-dihydroxyisovalerate
X_{14}	=	2-ketoisovalerate
X_{15}	=	2-isopropylmalate
X_{16}	=	isopropylmaleate
X_{17}	=	3-isopropylmalate
X_{18}	=	2-isopropyl-3-oxosuccinate
X_{19}	=	2-ketoisocaproate
X_{20}	=	leucine
X_{21}	=	3-phosphohydroxypyruvate
X_{22}	=	3-phosphoserine
X_{23}	=	L-serine
X_{24}	=	D-serine
X_{25}	=	glycine
X_{26}	=	threonine
X_{27}	=	2-oxobutanoate
X_{28}	=	2-aceto-2-hydroxybutyrate
X_{29}	=	2,3-dihydroxy-3-methylvalerate
X_{30}	=	2-keto-3-methylvalerate
X_{31}	=	isoleucine
X_{32}	=	S-2-methylbutyryl-CoA
X_{33}	=	tigloylCoA
X_{34}	=	2-methyl-3-hydroxybutyryl-CoA
X_{35}	=	2-methylacetoacetyl-CoA
X_{36}	=	acetylCoA
X_{37}	=	glutamate
X_{38}	=	N-acetyl-L-glutamate
X_{39}	=	N-acetylglutamyl-P
X_{40}	=	N-acetyl-L-glutamate-5-semialdehyde
X_{41}	=	N-acetyl-L-ornithine
X_{42}	=	ornithine
X_{43}	=	carbamoyl-P
X_{44}	=	glutamine
X_{45}	=	citrulline
X_{46}	=	argininosuccinate
X_{47}	=	arginine
X_{48}	=	glutamate-gamma-semialdehyde
X_{49}	=	S-1-pyrroline-5-carboxylate

X_{50}	=	proline
X_{51}	=	O-acetyl-L-serine
X_{52}	=	5-phosphoribosyl-1-pyrophosphate
X_{53}	=	phosphoribosylATP
X_{54}	=	phosphoribosylAMP
X_{55}	=	phosphoribosylforniminoAICARP
X_{56}	=	phosphoribulosylformiminoAICARP
X_{57}	=	AICAR
X_{58}	=	D-erythroimidazoleglycerol-P
X_{59}	=	imidazoleacetol-P
X_{60}	=	histidinolP
X_{61}	=	histidinol
X_{62}	=	histidinal
X_{63}	=	histidine
X_{64}	=	citrate
X_{65}	=	cisaconitate
X_{66}	=	isocitrate
X_{67}	=	ketoglutarate
X_{68}	=	succinyl-CoA
X_{69}	=	succinate
X_{70}	=	fumarate
X_{71}	=	malate
X_{72}	=	oxaloacetate
X_{73}	=	alanine
X_{74}	=	cysteine
X_{75}	=	4-aminobutyrate
X_{76}	=	succinatesemialdehyde
X_{77}	=	asparagine
X_{78}	=	aspartate
X_{79}	=	aspartate-4P
X_{80}	=	aspartatesemialdehyde
X_{81}	=	homoserine
X_{82}	=	O-phospho-L-homoserine
X_{83}	=	cystathionine
X_{84}	=	homocysteine
X_{85}	=	methionine
X_{86}	=	S-adenosyl-L-methionine
X_{87}	=	S-adenosyl-L-homocysteine
X_{88}	=	adenosine
X_{89}	=	L-2,3-dihydrodipicolinate
X_{90}	=	tetrahydrodipicolinate
X_{91}	=	L,L-diamionopimelate
X_{92}	=	mesodiaminopimelate
X_{93}	=	lysine
X_{94}	=	S-methyl-5-thioadenosine
X_{95}	=	5-methythioribose
X_{96}	=	5-methylthioribose-1P
X_{97}	=	5-methioribulose-1P
X_{98}	=	5-methylthio-2,3-dioxopentyl-P

X_{99} = 2-hydroxy-3-keto-5-methylthio-1-phosphopentene
X_{100} = 1,2-dihydroxy-3-keto-5-methylthiopentene
X_{101} = 2-oxo-4-methylthiobutanoate
X_{102} = 3-deoxyDarabinoheptulosonate-7P
X_{103} = 3-dehydroquinate
X_{104} = 3-dehydroshikimate
X_{105} = shikimate
X_{106} = shikimate-3P
X_{107} = 5-enolpyruvylshikimate-3P
X_{108} = prephenate
X_{109} = arogenate
X_{110} = phenylalanine
X_{111} = phenylpyruvate
X_{112} = phenylacetaldehyde
X_{113} = tyrosine
X_{114} = phydroxyphenylpyruvate
X_{115} = homogentisate
X_{116} = maleylacetoacetate
X_{117} = 4-fumarylacetoacetate
X_{118} = anthranilate
X_{119} = N-5-phosphoribosylanthranilate
X_{120} = 1-O-carboxyphenylamino-1-deoxyribulose-5P
X_{121} = indole3glycerolP
X_{122} = indole
X_{123} = tryptophan
X_{124} = valine
X_{125} = glyoxylate
X_{126} = SmethylLmethionine
X_{127} = chorismate
X_{128} = glutamylcysteine
X_{129} = glutathione
X_{130} = 2-methylmalate
X_{131} = methylmaleate
X_{132} = 3-methylmalate
X_{133} = hydroxylysine
X_{134} = acetylhydroxylysine
X_{135} = D-lysine
X_{136} = diaminohexanoate
X_{137} = N-acetyllysine
X_{138} = acetamidooxohexanoate
X_{139} = aminooxohexanoate
X_{140} = piperideine-2-carboxylate
X_{141} = pipecolate
X_{142} = piperideine-6-carboxylate
X_{143} = aminoadipatesemialdehyde
X_{144} = saccharopine
X_{145} = cadaverine
X_{146} = piperideine
X_{147} = aminopentanamide

X_{148} = aminopentanoate
X_{149} = acetyllysine
X_{150} = acetamidopentanoate
X_{151} = glutaratesemialdehyde
X_{152} = glutarate
X_{153} = aminoadipate
X_{154} = oxoadipate
X_{155} = glutaryldihydrolipoamide
X_{156} = glutaryl-CoA
X_{157} = crotonoyl-CoA
X_{158} = hydroxybutanoyl-CoA
X_{159} = acetoacetyl-CoA
X_{160} = 2,2-methylthioethylmalate
X_{161} = 3,2-methylthioethylmalate
X_{162} = 2-oxo-5-methylthiopentanoicacid
X_{163} = 2,3-methylthiopropylmalate
X_{164} = 3,3-methylthiopropylmalte
X_{165} = 2-oxo-6-methylthiohexanoate
X_{166} = 2,4-methylthiobutylmalate
X_{167} = 3,4-methylthiobutylmalate
X_{168} = 2-oxo-7-methylthioheptanoate
X_{169} = 2,5-methylthiopentylmalate
X_{170} = 3,5-methylthiobutylmalate
X_{171} = 2-oxo-8-methyloctanoate
X_{172} = 2,6-methylthiohexylmalate
X_{173} = 3,6-methylthiohexylmalate
X_{174} = 2-oxo-9-methylononanoate
X_{175} = 2,7-methylthioheptylmalate
X_{176} = 3,7-methylthioheptylmalate
X_{177} = 2-oxo-1,0-methylthiodecanoate
X_{178} = homomethionine
X_{179} = methylthiobutanaldoxime
X_{180} = methylthiobutanonitrileoxide
X_{181} = methylthiobutylhydroximoylglutathione
X_{182} = methylthiobutylhydroximoylcysteinylglycine
X_{183} = methylthiobutylhydroximate
X_{184} = methylthiopropyldesulfoglucosinolate
X_{185} = methylthiopropylglucosinolate
X_{186} = methylsulfinylpropylglucosinolate
X_{187} = propernylglucosinolate
X_{188} = hydroxypropylglucosinolate
X_{189} = benzoyloxypropylglucosinolate
X_{190} = sinapoyloxypropylglucosinolate
X_{191} = dihomomethionine
X_{192} = methylthiopentanaldoxime
X_{193} = methylthiopentanonitrileoxide
X_{194} = methylthiopentylhydroximoylglutathione
X_{195} = methylthiopentylhydroximoylcysteinylglycine
X_{196} = methylthiopentylhydroximate

X_{197} = methylthiobutyldesulfoglucosinolate
X_{198} = methylthiobutylglucosinolate
X_{199} = methylsulfinylbutylglucosinolate
X_{200} = butenylglucosinolate
X_{201} = hydroxy-3-butenylglucosinolate
X_{202} = benzoyloxy-3-butenylglucosinolate
X_{203} = sinapoyloxy-3-butenylglucosinolate
X_{204} = hydroxybutylglucosinolate
X_{205} = benzoyloxybutylglucosinolate
X_{206} = sinapoyloxybutylglucosinolate
X_{207} = trihomomethionine
X_{208} = methylthiohexanaldoxime
X_{209} = methylthiohexanonitrildoxide
X_{210} = methylthiohexylhydroximoylglutathione
X_{211} = methylthiohexylhydroximoylcysteinylglycine
X_{212} = methylthiohexylhydroximate
X_{213} = methylthiopentyldesulfoglucosinolate
X_{214} = methylthiopentylglusinolate
X_{215} = methylsulfinylpentylglucosinolate
X_{216} = pentenylglucosinolate
X_{217} = tetrahomomethionine
X_{218} = methylthioheptanaldoxime
X_{219} = methylthioheptanonitrileoxide
X_{220} = methylthioheptylhydroximoylglutathione
X_{221} = methylthioheptylhydroximoylcysteinylglycine
X_{222} = methylthioheptylhydroximate
X_{223} = methylthiohexyldesulfoglucosinolate
X_{224} = methylthiohexylglucosinolate
X_{225} = methylsulfinylhexylglucosinolate
X_{226} = pentahomomethionine
X_{227} = methylthiooctanaldoxime
X_{228} = methylthiooctanonitrileoxide
X_{229} = methylthiooctylhydroximoylglutathione
X_{230} = methylthiooctylhydroximoylcysteinylglycine
X_{231} = methylthiooctylhydroximate
X_{232} = methylthioheptyldesulfoglucosinolate
X_{233} = methylthioheptylglucosinolate
X_{234} = methylsulfinylheptylglucosinolate
X_{235} = hexahomomethionine
X_{236} = methylthiononanaldoxime
X_{237} = methylthiononanonitrileoxide
X_{238} = methylthiononylhydroximoylglutathionine
X_{239} = methylthiononylhydroximoylcysteinylglycine
X_{240} = methylthiononylhydroximate
X_{241} = methylthiooctyldesulfoglucosinolate
X_{242} = methylthiooctylglucosinolate
X_{243} = methylsulfinyloctylglucosinolate
X_{244} = phenylacetaldoxime
X_{245} = phenylacetonitrileoxide

X_{246} = phenylacetohydroximoylglutathione
X_{247} = phenylacetohydroximoylcysteinylglycine
X_{248} = phenylacetothiohydroximate
X_{249} = benzyldesulfoglucosinolate
X_{250} = benzylglucosinolate
X_{251} = indole-3-acetaldoxime
X_{252} = indole-3-acetonitrileoxide
X_{253} = indole-3-acetohydroximoylglutathione
X_{254} = indole-3-acetohydroximoylcysteinylglycine
X_{255} = indolylmethylthiohydroximate
X_{256} = indolylmethyldesulfoglucosinolate
X_{257} = indol-3-ylmethylglucosinolate
X_{258} = 1-hydroxy-3-indolylmethylglucosinolate
X_{259} = 1-methoxy-3-indolylmethylglucosinolate
X_{260} = 4-hydroxy-3-indolylmethylglucosinolate
X_{261} = 4-methoxy-3-indolylmethylglucosinolate
X_{262} = histamine
X_{263} = imidazoleacetaldehyde
X_{264} = imidazole4acetate
X_{265} = imidazoloneacetate
X_{266} = NformiminoLaspartate
X_{267} = NformylLaspartate
X_{268} = D-glucono-1,5-lactone-6P
X_{269} = 6-phospho-D-gluconate
X_{270} = D-ribulose-5P
X_{271} = D-ribose-5P
X_{272} = D-ribose
X_{273} = D-ribose-1P
X_{274} = D-ribose-1,5P
X_{275} = D-xylulose-5P
X_{276} = D-erythrose-4P
X_{277} = D-sedoheptulose-7P
X_{278} = D-gluconate
X_{279} = D-glucono-1,5-lactone
X_{280} = 2-dehydro-D-gluconate
X_{281} = 2-dehydrogluconate-6P
X_{282} = 5-phospho-B-D-ribosylamine
X_{283} = 5-phosphoribosylglycineamine
X_{284} = 5-phosphoribosyl-N-formylglycineamide
X_{285} = 5-phosphoribosyl-N-formylglycineamidine
X_{286} = 5-aminoimidazoleribonucleotide
X_{287} = 4-carboxyaminoimidazoleribonucleotide
X_{288} = 5-phosphoribosyl-4-N-succinocarboxamide-5-aminoimidazole
X_{289} = phosphoribosylformamidocarboxamide
X_{290} = inosine-5-phosphate
X_{291} = adenylosuccinate
X_{292} = xanthosine-5-phosphate
X_{293} = AMP
X_{294} = GMP

X_{295} = inosine
X_{296} = hypoxanthine
X_{297} = xanthosine
X_{298} = guanine
X_{299} = xanthine
X_{300} = urate
X_{301} = 5-hydroxyisourate
X_{302} = 5-hydroxy-2-oxo-4-ureido-2,5-dihydro-1H-imidazole-5-carboxylate
X_{303} = S-allantoin
X_{304} = allantoate
X_{305} = ureidoglycine
X_{306} = ureidoglycolate
X_{307} = urea
X_{308} = GDP
X_{309} = GTP
X_{310} = dGDP
X_{311} = dGTP
X_{312} = ADP
X_{313} = dADP
X_{314} = ATP
X_{315} = dATP
X_{316} = dAMP
X_{317} = deoxyadenosine
X_{318} = deoxyinosine
X_{319} = adenine
X_{320} = guanosine
X_{321} = NcarbamoylLaspartate
X_{322} = dihydroorotate
X_{323} = orotate
X_{324} = orotidine-5P
X_{325} = UMP
X_{326} = UDP
X_{327} = UTP
X_{328} = CTP
X_{329} = CMP
X_{330} = CDP
X_{331} = dCDP
X_{332} = dCTP
X_{333} = dihydrouracil
X_{334} = dUDP
X_{335} = uracil
X_{336} = dUTP
X_{337} = dUMP
X_{338} = dTMP
X_{339} = dTDP
X_{340} = dTTP
X_{341} = dCMP
X_{342} = deoxycytidine
X_{343} = deoxyuridine

X_{344}	=	3-ureidopropionate
X_{345}	=	thymidine
X_{346}	=	thymine
X_{347}	=	dihydrothymine
X_{348}	=	3-ureidoisobutyrate
X_{349}	=	3-aminoisobutanoate
X_{350}	=	cytidine
X_{351}	=	uridine
X_{352}	=	Supplementation of threonine in medium
X_{353}	=	Supplementation of lysine in medium
X_{354}	=	Transporation of threonine to cells
X_{355}	=	Transporation of lysine to cells

V_{1} = sucrose*2
V_{2} = glucose6P − fructose6P
V_{3} = fructose6P
V_{4} = fructose16bisP
V_{5} = fructose16bisP − glyceraldehyde3P
V_{6} = fructose16bisP − dihydroxyacetoneP
V_{7} = dihydroxyacetoneP − glyceraldehyde3P
V_{8} = glyceraldehyde3P − 13diPglycerate
V_{9} = 13diPglycerate − 3phosphoglycerate
V_{10} = 3phosphoglycerate − 2phosphoglycerate
V_{11} = 2phosphoglycerate − phosphoenolpyruvate
V_{12} = phosphoenolpyruvate * 6phosphoDgluconate**0.5 / glutamate**0.5
V_{13} = pyruvate
V_{14} = 3phosphoglycerate − 3phosphohydroxypyruvate
V_{15} = 3phosphohydroxypyruvate**0.5*glutamate**0.5
V_{16} = 3phosphoserine
V_{17} = Lserine
V_{18} = Dserine
V_{19} = Lserine − glycine
V_{20} = glyoxylate**0.5*glutamate**0.5
V_{21} = glyoxylate**0.5*Lserine**0.5
V_{22} = threonine − glycine
V_{23} = threonine*valine**0.5/isoleucine**0.5
V_{24} = 2oxobutanoate*pyruvate
V_{25} = 2aceto2hydroxybutyrate
V_{26} = 23dihydroxy3methylvalerate
V_{27} = 2keto3methylvalerate**0.5*glutamate**0.5 − isoleucine**0.5*ketoglutarate**0.5
V_{28} = 2keto3methylvalerate
V_{29} = S2methylbutyrylCoA
V_{30} = tigloylCoA
V_{31} = 2methyl3hydroxybutyrylCoA
V_{32} = 2methylacetoacetylCoA
V_{33} = acetylCoA
V_{34} = pyruvate / isoleucine**0.5 / leucine**0.5 / valine**0.5
V_{35} = 2acetolactate
V_{36} = 23dihydroxyisovalerate
V_{37} = 2ketoisovalerate**0.5*glutamate**0.5 − valine**0.5*ketoglutarate**0.5
V_{38} = 2ketoisovalerate**0.5*acetylCoA**0.5/leucine**0.5
V_{39} = 2isopropylmalate
V_{40} = isopropylmaleate
V_{41} = 3isopropylmalate
V_{42} = 2isopropyl3oxosuccinate
V_{43} = 2ketoisocaproate**0.5*glutamate**0.5 − leucine**0.5*ketoglutarate**0.5
V_{44} = glutamate
V_{45} = NacetylLglutamate
V_{46} = NacetylglutamylP
V_{47} = NacetylLglutamate5semialdehyde
V_{48} = NacetylLornithine
V_{49} = ornithine − citrulline
V_{50} = carbamoylP − citrulline
V_{51} = ornithine − carbamoylP
V_{52} = citrulline
V_{53} = argininosuccinate/argininosuccinate**0.5
V_{54} = glutamine
V_{55} = arginine
V_{56} = ornithine − glutamateGsemialdehyde
V_{57} = glutamateGsemialdehyde
V_{58} = S1pyrroline5carboxylate
V_{59} = S1pyrroline5carboxylate
V_{60} = arginine
V_{61} = citrulline
V_{62} = citrulline − ornithine**0.5*carbamoylP**0.5
V_{63} = glutamate
V_{64} = succinatesemialdehyde
V_{65} = aspartate**0.5 * ketoglutarate**0.5
V_{66} = Lserine / cysteine**0.5
V_{67} = OacetylLserine
V_{68} = 5phosphoribosyl1pyrophosphate/histidine**0.5
V_{69} = phosphoribosylATP
V_{70} = phosphoribosylAMP
V_{71} = phosphoribosylforniminoAICARP
V_{72} = phosphoribulosylformiminoAICARP**0.5*glutamine**0.5
V_{73} = DerythroimidazoleglycerolP
V_{74} = imidazoleacetolP**0.5*glutamate**0.5
V_{75} = histidinolP
V_{76} = histidinol
V_{77} = histidinal

V_{78} = cysteine
V_{79} = pyruvate**0.5*glutamate**0.5 − alanine**0.5*ketoglutarate**0.5
V_{80} = glutamine
V_{81} = glutamate
V_{82} = glutamate
V_{83} = 4aminobutyrate**0.5*pyruvate**0.5
V_{84} = 4aminobutyrate**0.5*ketoglutarate**0.5
V_{85} = pyruvate
V_{86} = citrate
V_{87} = acetylCoA**0.5 * oxaloacetate**0.5
V_{88} = citrate
V_{89} = cisaconitate
V_{90} = isocitrate
V_{91} = isocitrate
V_{92} = ketoglutarate
V_{93} = glyoxylate
V_{94} = succinylCoA
V_{95} = succinate/oxaloacetate**0.5
V_{96} = fumarate
V_{97} = malate
V_{98} = oxaloacetate
V_{99} = oxaloacetate**0.5*glutamate**0.5
V_{100} = asparagine
V_{101} = aspartate**0.5*glutamate**0.5
V_{102} = aspartate * alanine**0.5 * cysteine**0.5 * isoleucine**0.5 * valine**0.5 * leucine**0.5 * Lserine**0.5 / lysine**0.5 / threonine**0.5 / SadenosylLmethionine**0.5
V_{103} = aspartate4P
V_{104} = aspartatesemialdehyde / cysteine**0.5 / threonine**0.5
V_{105} = homoserine
V_{106} = OphosphoLhomoserine * SadenosylLmethionine**0.5
V_{107} = OphosphoLhomoserine**0.5 * cysteine**0.5 / SadenosylLmethionine**0.5
V_{108} = cystathionine
V_{109} = aspartatesemialdehyde**0.5 * pyruvate**0.5 / lysine**0.5
V_{110} = L23dihydrodipicolinate
V_{111} = tetrahydrodipicolinate**0.5 * glutamate**0.5
V_{112} = LLdiamionopimelate
V_{113} = mesodiaminopimelate
V_{114} = SmethylLmethionine**0.5 * homocysteine**0.5
V_{115} = methionine**0.5 * SadenosylLmethionine**0.5
V_{116} = methionine / SadenosylLmethionine**0.5
V_{117} = SadenosylLmethionine
V_{118} = SadenosylLhomocysteine
V_{119} = homocysteine
V_{120} = methionine
V_{121} = SadenosylLmethionine
V_{122} = Smethyl5thioadenosine
V_{123} = 5methythioribose / SadenosylLhomocysteine**0.5
V_{124} = 5methylthioribose1P
V_{125} = 5methioribulose1P
V_{126} = 5methylthio23dioxopentylP
V_{127} = 2hydroxy3keto5methylthio1phosphopentene
V_{128} = 12dihydroxy3keto5methylthiopentene
V_{129} = 2oxo4methylthiobutanoate − methionine
V_{130} = Derythrose4P**0.5 * phosphoenolpyruvate**0.5
V_{131} = 3deoxyDarabinoheptulosonate7P
V_{132} = 3dehydroquinate
V_{133} = 3dehydroshikimate
V_{134} = shikimate
V_{135} = shikimate3P**0.5 * phosphoenolpyruvate**0.5
V_{136} = 5enolpyruvylshikimate3P
V_{137} = chorismate * tryptophan**0.5 / phenylalanine**0.5 / tyrosine**0.5
V_{138} = prephenate**0.5 * glutamate**0.5
V_{139} = arogenate
V_{140} = arogenate / tyrosine**0.5
V_{141} = phenylalanine**0.5 * ketoglutarate**0.5
V_{142} = phenylalanine**0.5 * pyruvate**0.5
V_{143} = phenylpyruvate
V_{144} = phenylacetaldehyde
V_{145} = tyrosine**0.5 * ketoglutarate**0.5
V_{146} = phydroxyphenylpyruvate
V_{147} = homogentisate
V_{148} = maleylacetoacetate
V_{149} = 4fumarylacetoacetate
V_{150} = 4fumarylacetoacetate
V_{151} = chorismate**0.5 * glutamine**0.5 / tryptophan**0.5
V_{152} = anthranilate**0.5 * 5phosphoribosyl1pyrophosphate**0.5
V_{153} = N5phosphoribosylanthranilate
V_{154} = 1Ocarboxyphenylamino1deoxyribulose5P

V_{155} = indole3glycerolP
V_{156} = indole**0.5 * Lserine**0.5
V_{157} = glutamate**0.5 * cysteine**0.5 / glutathione**0.5
V_{158} = glutamylcysteine / glutathione**0.5
V_{159} = pyruvate
V_{160} = 2methylmalate
V_{161} = methylmaleate
V_{162} = 3methylmalate
V_{163} = lysine − Dlysine
V_{164} = Dlysine
V_{165} = diaminohexanoate
V_{166} = lysine
V_{167} = hydroxylysine
V_{168} = acetylhydroxylysine
V_{169} = lysine − acetyllysine
V_{170} = lysine
V_{171} = cadaverine
V_{172} = piperideine
V_{173} = lysine
V_{174} = aminopentanamide − aminopentanoate
V_{175} = acetamidopentanoate − aminopentanoate
V_{176} = acetamidooxohexanoate − acetamidopentanoate
V_{177} = acetyllysine − acetamidooxohexanoate
V_{178} = aminopentanoate − glutaratesemialdehyde
V_{179} = glutaratesemialdehyde − glutarate
V_{180} = glutarate
V_{181} = lysine
V_{182} = Nacetyllysine
V_{183} = lysine
V_{184} = Nacetyllysine − acetamidooxohexanoate
V_{185} = acetamidooxohexanoate
V_{186} = Dlysine
V_{187} = aminooxohexanoate
V_{188} = piperideine2carboxylate
V_{189} = pipecolate
V_{190} = lysine**0.5 * ketoglutarate**0.5
V_{191} = saccharopine − aminoadipatesemialdehyde
V_{192} = piperideine6carboxylate − aminoadipatesemialdehyde
V_{193} = aminoadipatesemialdehyde
V_{194} = aminoadipate − oxoadipate
V_{195} = oxoadipate
V_{196} = glutaryldihydrolipoamide − glutarylCoA
V_{197} = glutarylCoA
V_{198} = crotonoylCoA
V_{199} = hydroxybutanoylCoA
V_{200} = acetoacetylCoA
V_{201} = 2oxo4methylthiobutanoate
V_{202} = 22methylthioethylmalate
V_{203} = 32methylthioethylmalate
V_{204} = 2oxo5methylthiopentanoicacid
V_{205} = 23methylthiopropylmalate
V_{206} = 33methylthiopropylmalte
V_{207} = 2oxo6methylthiohexanoate
V_{208} = 24methylthiobutylmalate
V_{209} = 34methylthiobutylmalate
V_{210} = 2oxo7methylthioheptanoate
V_{211} = 25methylthiopentylmalate
V_{212} = 35methylthiobutylmalate
V_{213} = 2oxo8methyloctanoate
V_{214} = 26methylthiohexylmalate
V_{215} = 36methylthiohexylmalate
V_{216} = 2oxo9methylononanoate
V_{217} = 27methylthioheptylmalate
V_{218} = 37methylthioheptylmalate
V_{219} = 2oxo5methylthiopentanoicacid − homomethionine
V_{220} = homomethionine
V_{221} = methylthiobutanaldoxime
V_{222} = methylthiobutanonitrileoxide
V_{223} = methylthiobutylhydroximoylglutathione
V_{224} = methylthiobutylhydroximoylcysteinylglycine
V_{225} = methylthiobutylhydroximate
V_{226} = methylthiopropyldesulfoglucosinolate
V_{227} = methylthiopropylglucosinolate
V_{228} = methylsulfinylpropylglucosinolate
V_{229} = methylsulfinylpropylglucosinolate
V_{230} = hydroxypropylglucosinolate
V_{231} = hydroxypropylglucosinolate

V_{232} = 2oxo6methylthiohexanoate − dihomomethionine
V_{233} = dihomomethionine
V_{234} = methylthiopentanaldoxime
V_{235} = methylthiopentanonitrileoxide
V_{236} = methylthiopentylhydroximoylglutathione
V_{237} = methylthiopentylhydroximoylcysteinylglycine
V_{238} = methylthiopentylhydroximate
V_{239} = methylthiobutyldesulfoglucosinolate
V_{240} = methylthiobutylglucosinolate
V_{241} = methylsulfinylbutylglucosinolate
V_{242} = butenylglucosinolate
V_{243} = hydroxy3butenylglucosinolate
V_{244} = hydroxy3butenylglucosinolate
V_{245} = methylsulfinylbutylglucosinolate
V_{246} = hydroxybutylglucosinolate
V_{247} = hydroxybutylglucosinolate
V_{248} = 2oxo7methylthioheptanoate − trihomomethionine
V_{249} = trihomomethionine
V_{250} = methylthiohexanaldoxime
V_{251} = methylthiohexanonitrildoxide
V_{252} = methylthiohexylhydroximoylglutathione
V_{253} = methylthiohexylhydroximoylcysteinylglycine
V_{254} = methylthiohexylhydroximate
V_{255} = methylthiopentyldesulfoglucosinolate
V_{256} = methylthiopentylglusinolate
V_{257} = methylsulfinylpentylglucosinolate
V_{258} = 2oxo8methyloctanoate − tetrahomomethionine
V_{259} = tetrahomomethionine
V_{260} = methylthioheptanaldoxime
V_{261} = methylthioheptanonitrileoxide
V_{262} = methylthioheptylhydroximoylglutathione
V_{263} = methylthioheptylhydroximoylcysteinylglycine
V_{264} = methylthioheptylhydroximate
V_{265} = methylthiohexyldesulfoglucosinolate
V_{266} = methylthiohexylglucosinolate
V_{267} = 2oxo9methylononanoate − pentahomomethionine
V_{268} = pentahomomethionine
V_{269} = methylthiooctanaldoxime
V_{270} = methylthiooctanonitrileoxide
V_{271} = methylthiooctylhydroximoylglutathione
V_{272} = methylthiooctylhydroximoylcysteinylglycine
V_{273} = methylthiooctylhydroximate
V_{274} = methylthioheptyldesulfoglucosinolate
V_{275} = methylthioheptylglucosinolate
V_{276} = 2oxo10methylthiodecanoate − hexahomomethionine
V_{277} = hexahomomethionine
V_{278} = methylthiononanaldoxime
V_{279} = methylthiononanonitrileoxide
V_{280} = methylthiononylhydroximoylglutathionine
V_{281} = methylthiononylhydroximoylcysteinylglycine
V_{282} = methylthiononylhydroximate
V_{283} = methylthiooctyldesulfoglucosinolate
V_{284} = methylthiooctylglucosinolate
V_{285} = phenylalanine
V_{286} = phenylacetaldoxime
V_{287} = phenylacetonitrileoxide
V_{288} = phenylacetohydroximoylglutathione
V_{289} = phenylacetohydroximoylcysteinylglycine
V_{290} = phenylacetothiohydroximate
V_{291} = benzyldesulfoglucosinolate
V_{292} = tryptophan
V_{293} = indole3acetaldoxime
V_{294} = indole3acetonitrileoxide
V_{295} = indole3acetohydroximoylglutathione
V_{296} = indole3acetohydroximoylcysteinylglycine
V_{297} = indolylmethylthiohydroximate
V_{298} = indolylmethyldesulfoglucosinolate
V_{299} = indol3ylmethylglucosinolate
V_{300} = 1hydroxy3indolylmethylglucosinolate
V_{301} = indol3ylmethylglucosinolate
V_{302} = 4hydroxy3indolylmethylglucosinolate
V_{303} = histidine
V_{304} = histamine
V_{305} = imidazoleacetaldehyde
V_{306} = imidazole4acetate
V_{307} = imidazoloneacetate
V_{308} = NformiminoLaspartate

V_{309} = NformylLaspartate
V_{310} = glucose6P
V_{311} = Dglucono15lactone6P
V_{312} = glucose6P
V_{313} = 6phosphoDgluconate
V_{314} = Dribose5P − Dribulose5P
V_{315} = Dribose − Dribose5P
V_{316} = Dribose5P − Dribose1P
V_{317} = Dribose1P
V_{318} = Dribose15P
V_{319} = Dribose5P − x5phosphoribosyl1pyrophosphate
V_{320} = Dxylulose5P**0.5 * Dribose5P**0.5 − Dsedoheptulose7P**0.5 * glyceraldehyde3P**0.5
V_{321} = Dsedoheptulose7P**0.5 * glyceraldehyde3P**0.5 − fructose6P**0.5 * Derythrose4P**0.5
V_{322} = Dgluconate
V_{323} = 2dehydroDgluconate − Dgluconate
V_{324} = Dgluconate
V_{325} = 2dehydroDgluconate
V_{326} = 2dehydrogluconate6P
V_{327} = Dglucono15lactone
V_{328} = glucose6P
V_{329} = Dribulose5P
V_{330} = Dxylulose5P**0.5 * Derythrose4P**0.5 − fructose6P**0.5 * glyceraldehyde3P**0.5
V_{331} = cystathionine
V_{332} = homocysteine**0.5 * Lserine**0.5
V_{333} = homocysteine**0.5 * SadenosylLmethionine**0.5
V_{334} = 5phosphoribosyl1pyrophosphate**0.5 * glutamine**0.5
V_{335} = 5phosphoBDribosylamine**0.5 * glycine**0.5
V_{336} = 5phosphoribosylglycineamine
V_{337} = 5phosphoribosylNformylglycineamide**0.5 * glutamine**0.5
V_{338} = 5phosphoribosylNformylglycineamidine
V_{339} = 5aminoimidazoleribonucleotide
V_{340} = 4carboxyaminoimidazoleribonucleotide**0.5 * aspartate**0.5
V_{341} = 5phosphoribosyl4Nsuccinocarboxamide5aminoimidazole
V_{342} = AICAR
V_{343} = phosphoribosylformamidocarboxamide
V_{344} = inosine5phosphate**0.5 * aspartate**0.5
V_{345} = inosine5phosphate
V_{346} = adenylosuccinate
V_{347} = xanthosine5phosphate**0.5 * glutamine**0.5
V_{348} = AMP
V_{349} = inosine5phosphate / adenosine**0.5
V_{350} = inosine
V_{351} = hypoxanthine
V_{352} = inosine5phosphate
V_{353} = xanthosine5phosphate
V_{354} = xanthosine
V_{355} = GMP / adenosine**0.5
V_{356} = guanosine
V_{357} = guanosine
V_{358} = guanine
V_{359} = xanthine
V_{360} = urate
V_{361} = 5hydroxyisourate
V_{362} = 5hydroxy2oxo4ureido25dihydro1Himidazole5carboxylate
V_{363} = Sallantoin
V_{364} = allantoate
V_{365} = ureidoglycine
V_{366} = ureidoglycolate
V_{367} = allantoate
V_{368} = urea
V_{369} = guanosine
V_{370} = guanine**0.5 * 5phosphoribosyl1pyrophosphate**0.5
V_{371} = adenine**0.5 * 5phosphoribosyl1pyrophosphate**0.5
V_{372} = adenosine / adenine**0.5
V_{373} = adenosine
V_{374} = AMP
V_{375} = GMP
V_{376} = GDP
V_{377} = GDP
V_{378} = GTP
V_{379} = dGDP
V_{380} = AMP
V_{381} = ADP
V_{382} = ADP
V_{383} = dADP
V_{384} = ATP
V_{385} = dATP

V_{386} = dAMP
V_{387} = deoxyadenosine
V_{388} = deoxyinosine
V_{389} = dGTP
V_{390} = glutamine
V_{391} = carbamoylP
V_{392} = NcarbamoylLaspartate
V_{393} = dihydroorotate
V_{394} = orotate**0.5 * 5phosphoribosyl1pyrophosphate**0.5
V_{395} = orotidine5P
V_{396} = UMP
V_{397} = UDP
V_{398} = UDP
V_{399} = UTP
V_{400} = UTP
V_{401} = UTP
V_{402} = CTP
V_{403} = CTP
V_{404} = CDP
V_{405} = CMP
V_{406} = CDP
V_{407} = dCDP − dCTP
V_{408} = CTP
V_{409} = UDP
V_{410} = dCTP
V_{411} = UTP
V_{412} = dUDP
V_{413} = dUTP
V_{414} = dUMP
V_{415} = dTMP
V_{416} = dTDP
V_{417} = dTTP
V_{418} = dCMP
V_{419} = dCDP
V_{420} = dCMP
V_{421} = dCMP
V_{422} = deoxycytidine
V_{423} = deoxyuridine
V_{424} = dUMP
V_{425} = dTMP
V_{426} = thymidine
V_{427} = thymidine
V_{428} = thymine
V_{429} = dihydrothymine
V_{430} = 3ureidoisobutyrate
V_{431} = 3aminoisobutanoate
V_{432} = CMP
V_{433} = cytidine
V_{434} = uridine
V_{435} = uracil**0.5 * 5phosphoribosyl1pyrophosphate**0.5
V_{436} = UMP
V_{437} = uridine**0.5 * GTP**0.5
V_{438} = uracil
V_{439} = dihydrouracil
V_{440} = 3ureidopropionate
V_{441} = methionine

$f_{1} = a2*(\ 0\ )$

$f_{2} = a2*(\ V_{1} - V_{2} - V_{310} - V_{312} - V_{328}\ )$

$f_{3} = a2*(\ V_{2} + V_{4} - V_{3} + V_{330} + V_{321}\ )$

$f_{4} = a2*(\ V_{3} - V_{4} - V_{5} - V_{6}\ )$

$f_{5} = a2*(\ V_{5} + V_{7} - V_{8} + V_{320} - V_{321} + V_{330}\ )$

$f_{6} = a2*(\ V_{6} - V_{7}\ )$

$f_{7} = a2*(\ V_{8} - V_{9}\ )$

$f_{8} = a2*(\ V_{9} - V_{10} - V_{14}\ )$

$f_{9} = a2*(\ V_{10} - V_{11}\ )$

$f_{10} = a2*(\ V_{11} + V_{13} - V_{12}\ )$

$f_{11} = a2*(\ V_{12} - V_{13} - V_{34} + V_{18} - V_{79} - V_{85} - V_{159} - \text{pyruvate}\ )$

$f_{12} = a2*(\ V_{34} - V_{35}\ )$

$f_{13} = a2*(\ V_{35} - V_{36}\ )$

$f_{14} = a2*(\ V_{36} - V_{37} - V_{38}\ )$

$f_{15} = a2*(\ V_{38} - V_{39}\ )$

$f_{16} = a2*(\ V_{39} - V_{40}\ )$

$f_{17} = a2*(\ V_{40} - V_{41}\ )$

$f_{18} = a2*(\ V_{41} - V_{42}\ )$

$f_{19} = a2*(\ V_{42} - V_{43}\ )$

$f_{20} = a2*(\ V_{43} - \text{leucine}\ )$

$f_{21} = a2*(\ V_{14} - V_{15}\ )$

$f_{22} = a2*(\ V_{15} - V_{16}\ )$

$f_{23} = a1*(\ V_{16} - V_{17} - V_{66} - V_{19} - \text{Lserine}\ )$

$f_{24} = a1*(\ V_{17} - V_{18}\ )$

$f_{25} = a2*(\ V_{19} + V_{20} + V_{21} + V_{22} - \text{glycine}\ )$

$f_{26} = a2*(\ V_{106} - V_{22} - V_{23} - \text{threonine}\ ) + 0.04*X353$

$f_{27} = a2*(\ V_{23} - V_{24} + V_{162} + V_{441}\ )$

$f_{28} = a2*(\ V_{24} - V_{25}\ )$

$f_{29} = a2*(\ V_{25} - V_{26}\ )$

$f_{30} = a2*(\ V_{26} - V_{27} - V_{28}\ )$

$f_{31} = a2*(\ V_{27} - \text{isoleucine}\ )$

$f_{32} = a2*(\ V_{28} - V_{29}\ )$

$f_{33} = a2*(\ V_{29} - V_{30}\ )$

$f_{34} = a2*(\ V_{30} - V_{31}\ )$

$f_{35} = a2*(\ V_{31} - V_{32}\ )$

$f_{36} = a2*(\ V_{32} - V_{33} + V_{85} + V_{86} + V_{200}\ )$

$f_{37} = a2*(\ V_{80} - V_{44} - V_{81} - V_{82} - V_{63} + V_{58} + V_{65}/2 - V_{157}/2 - V_{74}/2 - \text{glutamate}\ )$

$f_{38} = a2*(\ V_{44} - V_{45}\ )$

$f_{39} = a2*(\ V_{45} - V_{46}\ )$

$f_{40} = a2*(\ V_{46} - V_{47}\ )$

$f_{41} = a2*(\ V_{47} - V_{48}\ )$

$f_{42} = a3*(\ V_{48} - V_{49} - V_{51} + V_{55} - V_{56} + V_{61} + V_{62}\ )$

$f_{43} = a2*(\ V_{51} + V_{54} - V_{50} - V_{391}\ )$

$f_{44} = a2*(\ V_{63} - V_{54} - V_{80} + \text{constant} - \text{glutamine} - V_{72}/2\ )$

$f_{45} = a2*(\ V_{49} + V_{50} - V_{52} + V_{60} - V_{61} - V_{62}\ )$

$f_{46} = a2*(\ V_{52} - V_{53}\ )$

$f_{47} = a2*(\ V_{53} - V_{55} - V_{60} - \text{arginine}\ )$

$f_{48} = a3*(\ V_{56} - V_{57}\ )$

$f_{49} = a3*(\ V_{57} - V_{58} - V_{59}\ )$

$f_{50} = a3*(\ V_{59} - \text{proline}\ )$

$f_{51} = a2*(\ V_{66} - V_{67}\ )$

$f_{52} = a2*(\ V_{319} + V_{318} - V_{68} - V_{334} - V_{370}/2 - V_{371}/2 - V_{394}/2\ )$

$f_{53} = a2*(\ V_{68} - V_{69}\ )$

$f_{54} = a2*(\ V_{69} - V_{70}\ )$

$f_{55} = a2*(\ V_{70} - V_{71}\ )$

$f_{56} = a2*(\ V_{71} - V_{72}/2\ )$

$f_{57} = a2*(\ V_{341} - V_{342} + V_{72}/2\ )$

$f_{58} = a2*(\ V_{72}/2 - V_{73}\ )$

$f_{59} = a2*(\ V_{73} - V_{74}/2\ )$

$f_{60} = a2*(\ V_{74} - V_{75}\ )$

$f_{61} = a2*(\ V_{75} - V_{76}\ )$

$f_{62} = a2*(\ V_{76} - V_{77}\ )$

$f_{63} = a2*(V_{77} - \text{histidine} - V_{303})$

$f_{64} = a2*(V_{87} - V_{88} - V_{86})$

$f_{65} = a2*(V_{88} - V_{89})$

$f_{66} = a2*(V_{89} - V_{90} - V_{91})$

$f_{67} = a2*(V_{90} - V_{92} + V_{81} - V_{65}/2 - V_{190}/2)$

$f_{68} = a2*(V_{92} - V_{94})$

$f_{69} = a2*(V_{94} - V_{95} + V_{64})$

$f_{70} = a2*(V_{95} - V_{96} + V_{150})$

$f_{71} = a2*(V_{96} + V_{93} - V_{97})$

$f_{72} = a2*(V_{97} - V_{87} + V_{65}/2)$

$f_{73} = a0*(V_{79} + V_{78} + V_{440} - \text{alanine})$

$f_{74} = a2*(V_{67} - V_{78} - V_{157}/2 - \text{cysteine})$

$f_{75} = a2*(V_{82} - V_{83} - V_{84})$

$f_{76} = a2*(V_{83} + V_{84} - V_{64})$

$f_{77} = a2*(V_{101} - V_{100} - \text{asparagine})$

$f_{78} = a2*(V_{100} - V_{101} - V_{102} - V_{65}/2 + V_{309} - \text{aspartate})$

$f_{79} = a2*(V_{102} - V_{103})$

$f_{80} = a2*(V_{103} - V_{104} - V_{109})$

$f_{81} = a2*(V_{104} - V_{105})$

$f_{82} = a2*(V_{105} - V_{106} - V_{107})$

$f_{83} = a3*(V_{107} - V_{108} + V_{332})$

$f_{84} = a3*(V_{118}/2 - V_{119} + V_{108} - V_{114}/2 - V_{332} - V_{333}/2)$

$f_{85} = a3*(V_{129} - V_{120} + V_{119} - V_{116} + V_{114} - V_{115}/2 - \text{methionine} + V_{333}/2 + V_{441})$

$f_{86} = a3*(V_{120} - V_{121} + V_{116} - V_{117} - V_{115}/2 - V_{333}/2)$

$f_{87} = a3*(V_{117} - V_{118} + V_{115}/2 + V_{333}/2)$

$f_{88} = a3*(V_{374} - V_{373} - V_{372} + V_{118}/2)$

$f_{89} = a2*(V_{109} - V_{110})$

$f_{90} = a2*(V_{110} - V_{111})$

$f_{91} = a2*(V_{111} - V_{112})$

$f_{92} = a2*(V_{112} - V_{113})$

$f_{93} = a2*(V_{113} - \text{lysine} + V_{182} - V_{181} - V_{163} - V_{166} - V_{169} - V_{173} - V_{170} - V_{190}/2 - V_{183}) + 0.011*X355$

$f_{94} = a2*(V_{121} - V_{122})$

$f_{95} = a2*(V_{122} - V_{123})$

$f_{96} = a2*(V_{123} - V_{124})$

$f_{97} = a2*(V_{124} - V_{125})$

$f_{98} = a2*(V_{125} - V_{126})$

$f_{99} = a2*(V_{126} - V_{127})$

$f_{100} = a2*(V_{127} - V_{128})$

$f_{101} = a2*(V_{128} - V_{129} - V_{201})$

$f_{102} = a2*(V_{130} - V_{131})$

$f_{103} = a2*(V_{131} - V_{132})$

$f_{104} = a2*(V_{132} - V_{133})$

$f_{105} = a2*(V_{133} - V_{134})$

$f_{106} = a2*(V_{134} - V_{135})$

$f_{107} = a2*(V_{135} - V_{136})$

$f_{108} = a2*(V_{137} - V_{138})$

$f_{109} = a2*(V_{138} - V_{139} - V_{140})$

$f_{110} = a2*(V_{139} - V_{141} - V_{142} - V_{285} - \text{phenylalanine})$

$f_{111} = a2*(V_{141} + V_{142} - V_{143})$

$f_{112} = a2*(V_{143} - V_{144})$

$f_{113} = a2*(V_{140} - V_{145} - \text{tyrosine})$

$f_{114} = a2*(V_{145} - V_{146})$

$f_{115} = a2*(V_{146} - V_{147})$

$f_{116} = a2*(V_{147} - V_{148})$

$f_{117} = a2*(V_{148} - V_{149} - V_{150})$

$f_{118} = a2*(V_{151} - V_{152})$

$f_{119} = a2*(V_{152} - V_{153})$

$f_{120} = a2*(V_{153} - V_{154})$

$f_{121} = a2*(V_{154} - V_{155})$

$f_{122} = a2*(V_{155} - V_{156})$

$f_{123} = a2*(V_{156} - V_{292} - \text{tryptophan})$

$f_{124} = a2*(V_{37} - \text{valine})$

f_{125}	=	a2*($V_{91} - V_{93} - V_{20} - V_{21}$	)
f_{126}	=	a3*($V_{115}/2 - V_{114}/2$	)
f_{127}	=	a2*($V_{136} - V_{137} - V_{151}$	)
f_{128}	=	a2*($V_{157} - V_{158}$	)
f_{129}	=	a2*(V_{158} − glutathione	)
f_{130}	=	a2*($V_{159} - V_{160}$	)
f_{131}	=	a2*($V_{160} - V_{161}$	)
f_{132}	=	a2*($V_{161} - V_{162}$	)
f_{133}	=	a3*($V_{166} - V_{167}$	)
f_{134}	=	a3*($V_{167} - V_{168}$	)
f_{135}	=	a3*($V_{163} - V_{164} - V_{186}$	)
f_{136}	=	a3*($V_{164} - V_{165}$	)
f_{137}	=	a3*($V_{181} - V_{182} - V_{184}$	)
f_{138}	=	a3*($V_{184} - V_{185} + V_{177} - V_{176}$	)
f_{139}	=	a3*($V_{183} + V_{185} + V_{186} - V_{187}$	)
f_{140}	=	a3*($V_{187} - V_{188}$	)
f_{141}	=	a3*($V_{188} - V_{189}$	)
f_{142}	=	a3*($V_{189} - V_{192}$	)
f_{143}	=	a3*($V_{192} + V_{191} - V_{193}$	)
f_{144}	=	a3*($V_{190} - V_{191}$	)
f_{145}	=	a3*($V_{170} - V_{171}$	)
f_{146}	=	a3*($V_{171} - V_{172}$	)
f_{147}	=	a3*($V_{173} - V_{174}$	)
f_{148}	=	a3*($V_{172} + V_{174} + V_{175} - V_{178}$	)
f_{149}	=	a3*($V_{169} - V_{177}$	)
f_{150}	=	a3*($V_{176} - V_{175}$	)
f_{151}	=	a3*($V_{178} - V_{179}$	)
f_{152}	=	a3*($V_{179} - V_{180}$	)
f_{153}	=	a3*($V_{193} - V_{194}$	)
f_{154}	=	a3*($V_{194} - V_{195}$	)
f_{155}	=	a3*($V_{195} - V_{196}$	)
f_{156}	=	a3*($V_{196} + V_{180} - V_{197}$	)
f_{157}	=	a3*($V_{197} - V_{198}$	)
f_{158}	=	a3*($V_{198} - V_{199}$	)
f_{159}	=	a3*($V_{199} - V_{200}$	)
f_{160}	=	a2*($V_{201} - V_{202}$	)
f_{161}	=	a2*($V_{202} - V_{203}$	)
f_{162}	=	a2*($V_{203} - V_{204} - V_{219}$	)
f_{163}	=	a2*($V_{204} - V_{205}$	)
f_{164}	=	a2*($V_{205} - V_{206}$	)
f_{165}	=	a2*($V_{206} - V_{207} - V_{232}$	)
f_{166}	=	a2*($V_{207} - V_{208}$	)
f_{167}	=	a2*($V_{208} - V_{209}$	)
f_{168}	=	a2*($V_{209} - V_{210} - V_{248}$	)
f_{169}	=	a2*($V_{210} - V_{211}$	)
f_{170}	=	a2*($V_{211} - V_{212}$	)
f_{171}	=	a2*($V_{212} - V_{213} - V_{258}$	)
f_{172}	=	a2*($V_{213} - V_{214}$	)
f_{173}	=	a2*($V_{214} - V_{215}$	)
f_{174}	=	a2*($V_{215} - V_{216} - V_{267}$	)
f_{175}	=	a2*($V_{216} - V_{217}$	)
f_{176}	=	a2*($V_{217} - V_{218}$	)
f_{177}	=	a2*($V_{218} - V_{276}$	)
f_{178}	=	a2*($V_{219} - V_{220}$	)
f_{179}	=	a2*($V_{220} - V_{221}$	)
f_{180}	=	a2*($V_{221} - V_{222}$	)
f_{181}	=	a2*($V_{222} - V_{223}$	)
f_{182}	=	a2*($V_{223} - V_{224}$	)
f_{183}	=	a2*($V_{224} - V_{225}$	)
f_{184}	=	a2*($V_{225} - V_{226}$	)
f_{185}	=	a2*($V_{226} - V_{227}$	)
f_{186}	=	a2*($V_{227} - V_{228} - V_{229}$	)

$f_{187} = a2*(V_{228} - X_{187})$

$f_{188} = a2*(V_{229} - V_{230} - V_{231})$

$f_{189} = a2*(V_{230} - X_{189})$

$f_{190} = a2*(V_{231} - X_{190})$

$f_{191} = a2*(V_{232} - V_{233})$

$f_{192} = a2*(V_{233} - V_{234})$

$f_{193} = a2*(V_{234} - V_{235})$

$f_{194} = a2*(V_{235} - V_{236})$

$f_{195} = a2*(V_{236} - V_{237})$

$f_{196} = a2*(V_{237} - V_{238})$

$f_{197} = a2*(V_{238} - V_{239})$

$f_{198} = a2*(V_{239} - V_{240})$

$f_{199} = a2*(V_{240} - V_{241} - V_{245})$

$f_{200} = a2*(V_{241} - V_{242})$

$f_{201} = a2*(V_{242} - V_{243} - V_{244})$

$f_{202} = a2*(V_{243} - X_{202})$

$f_{203} = a2*(V_{244} - X_{203})$

$f_{204} = a2*(V_{245} - V_{246} - V_{247})$

$f_{205} = a2*(V_{246} - X_{205})$

$f_{206} = a2*(V_{247} - X_{206})$

$f_{207} = a2*(V_{248} - V_{249})$

$f_{208} = a2*(V_{249} - V_{250})$

$f_{209} = a2*(V_{250} - V_{251})$

$f_{210} = a2*(V_{251} - V_{252})$

$f_{211} = a2*(V_{252} - V_{253})$

$f_{212} = a2*(V_{253} - V_{254})$

$f_{213} = a2*(V_{254} - V_{255})$

$f_{214} = a2*(V_{255} - V_{256})$

$f_{215} = a2*(V_{256} - V_{257})$

$f_{216} = a2*(V_{257} - X_{216})$

$f_{217} = a2*(V_{258} - V_{259})$

$f_{218} = a2*(V_{259} - V_{260})$

$f_{219} = a2*(V_{260} - V_{261})$

$f_{220} = a2*(V_{261} - V_{262})$

$f_{221} = a2*(V_{262} - V_{263})$

$f_{222} = a2*(V_{263} - V_{264})$

$f_{223} = a2*(V_{264} - V_{265})$

$f_{224} = a2*(V_{265} - V_{266})$

$f_{225} = a2*(V_{266} - X_{225})$

$f_{226} = a2*(V_{267} - V_{268})$

$f_{227} = a2*(V_{268} - V_{269})$

$f_{228} = a2*(V_{269} - V_{270})$

$f_{229} = a2*(V_{270} - V_{271})$

$f_{230} = a2*(V_{271} - V_{272})$

$f_{231} = a2*(V_{272} - V_{273})$

$f_{232} = a2*(V_{273} - V_{274})$

$f_{233} = a2*(V_{274} - V_{275})$

$f_{234} = a2*(V_{275} - X_{234})$

$f_{235} = a2*(V_{276} - V_{277})$

$f_{236} = a2*(V_{277} - V_{278})$

$f_{237} = a2*(V_{278} - V_{279})$

$f_{238} = a2*(V_{279} - V_{280})$

$f_{239} = a2*(V_{280} - V_{281})$

$f_{240} = a2*(V_{281} - V_{282})$

$f_{241} = a2*(V_{282} - V_{283})$

$f_{242} = a2*(V_{283} - V_{284})$

$f_{243} = a2*(V_{284} - X_{243})$

$f_{244} = a2*(V_{285} - V_{286})$

$f_{245} = a2*(V_{286} - V_{287})$

$f_{246} = a2*(V_{287} - V_{288})$

$f_{247} = a2*(V_{288} - V_{289})$

$f_{248} = a2*(V_{289} - V_{290})$

f_{249} = a2∗($V_{290} - V_{291}$)
f_{250} = a2∗($V_{291} - X_{250}$)
f_{251} = a2∗($V_{292} - V_{293}$)
f_{252} = a2∗($V_{293} - V_{294}$)
f_{253} = a2∗($V_{294} - V_{295}$)
f_{254} = a2∗($V_{295} - V_{296}$)
f_{255} = a2∗($V_{296} - V_{297}$)
f_{256} = a2∗($V_{297} - V_{298}$)
f_{257} = a2∗($V_{298} - V_{299} - V_{301}$)
f_{258} = a2∗($V_{299} - V_{300}$)
f_{259} = a2∗($V_{300} - X_{259}$)
f_{260} = a2∗($V_{301} - V_{302}$)
f_{261} = a2∗($V_{302} - X_{261}$)
f_{262} = a2∗($V_{303} - V_{304}$)
f_{263} = a2∗($V_{304} - V_{305}$)
f_{264} = a2∗($V_{305} - V_{306}$)
f_{265} = a2∗($V_{306} - V_{307}$)
f_{266} = a2∗($V_{307} - V_{308}$)
f_{267} = a2∗($V_{308} - V_{309}$)
f_{268} = a2∗($V_{310} - V_{311}$)
f_{269} = a2∗($V_{311} + V_{312} + V_{322} + V_{326} - V_{313}$)
f_{270} = a2∗($V_{313} + V_{314} - V_{329}$)
f_{271} = a2∗ ($- V_{314} - V_{316} - V_{319} - V_{320}$)
f_{272} = a2∗(0)
f_{273} = a2∗($V_{316} - V_{317}$)
f_{274} = a2∗($V_{317} - V_{318}$)
f_{275} = a2∗($V_{329} - V_{330} - V_{320}$)
f_{276} = a2∗($V_{321} - V_{330} - V_{130}$)
f_{277} = a2∗($V_{320} - V_{321}$)
f_{278} = a2∗($V_{327} - V_{324} + V_{323} - V_{322}$)
f_{279} = a2∗($V_{328} - V_{327}$)
f_{280} = a2∗($V_{324} - V_{323} - V_{325}$)
f_{281} = a2∗($V_{325} - V_{326}$)
f_{282} = a2∗($V_{334} - V_{335}$)
f_{283} = a2∗($V_{335} - V_{336}$)
f_{284} = a2∗($V_{336} - V_{337}$)
f_{285} = a2∗($V_{337} - V_{338}$)
f_{286} = a2∗($V_{338} - V_{339}$)
f_{287} = a2∗($V_{339} - V_{340}$)
f_{288} = a2∗($V_{340} - V_{341}$)
f_{289} = a2∗($V_{342} - V_{343}$)
f_{290} = a2∗($V_{343} - V_{344} - V_{345} + V_{348} - V_{349} - V_{352}$)
f_{291} = a2∗($V_{344} - V_{346}$)
f_{292} = a2∗($V_{345} - V_{347} + V_{352} - V_{353}$)
f_{293} = a2∗($V_{346} - V_{374} + V_{373} + V_{371} - V_{348} - V_{380}$)
f_{294} = a2∗($V_{347} - V_{355} + V_{369} + V_{370} - V_{375}$)
f_{295} = a2∗($V_{349} - V_{350}$)
f_{296} = a2∗($V_{350} - V_{351} + V_{388}$)
f_{297} = a2∗($V_{353} + V_{356} - V_{354}$)
f_{298} = a2∗($V_{357} - V_{358} - V_{370}/2$)
f_{299} = a2∗($V_{351} + V_{354} + V_{358} - V_{359}$)
f_{300} = a2∗($V_{359} - V_{360}$)
f_{301} = a2∗($V_{360} - V_{361}$)
f_{302} = a2∗($V_{361} - V_{362}$)
f_{303} = a2∗($V_{362} - V_{363}$)
f_{304} = a2∗($V_{363} - V_{364} - V_{367}$)
f_{305} = a2∗($V_{364} - V_{365}$)
f_{306} = a2∗($V_{365} - V_{366}$)
f_{307} = a2∗($V_{366} + V_{367} - V_{368}$)
f_{308} = a2∗($V_{375} - V_{376} - V_{377}$)
f_{309} = a2∗($V_{377} - V_{378}$)
f_{310} = a2∗($V_{376} - V_{379}$)

$f_{311} = a2*(V_{378} + V_{379} - V_{389})$

$f_{312} = a2*(V_{380} - V_{381} - V_{382})$

$f_{313} = a2*(V_{381} - V_{383})$

$f_{314} = a2*(V_{382} - V_{384})$

$f_{315} = a2*(V_{383} + V_{384} - V_{385})$

$f_{316} = a2*(V_{385} - V_{386})$

$f_{317} = a2*(V_{386} - V_{387})$

$f_{318} = a2*(V_{387} - V_{388})$

$f_{319} = a2*(V_{372} - V_{371}/2)$

$f_{320} = a2*(V_{355} - V_{357} - V_{369} - V_{356})$

$f_{321} = a2*(V_{391} - V_{392})$

$f_{322} = a2*(V_{392} - V_{393})$

$f_{323} = a2*(V_{393} - V_{394}/2)$

$f_{324} = a2*(V_{394} - V_{395})$

$f_{325} = a2*(V_{395} + V_{397} + V_{400} - V_{396} + V_{437} - V_{436} + V_{435})$

$f_{326} = a2*(V_{396} + V_{399} - V_{398} - V_{397} - V_{409})$

$f_{327} = a2*(V_{398} - V_{399} - V_{400} - V_{401} - V_{411})$

$f_{328} = a2*(V_{401} - V_{402} - V_{403} - V_{408})$

$f_{329} = a2*(V_{402} + V_{404} - V_{405} - V_{432})$

$f_{330} = a2*(V_{403} - V_{404} + V_{405} - V_{406})$

$f_{331} = a2*(V_{406} - V_{407} + V_{418} - V_{419})$

$f_{332} = a2*(V_{407} + V_{408} - V_{410})$

$f_{333} = a2*(V_{438} - V_{439})$

$f_{334} = a2*(V_{409} - V_{412})$

$f_{335} = a2*(V_{434} - V_{435} - V_{438})$

$f_{336} = a2*(V_{410} + V_{411} + V_{412} - V_{413})$

$f_{337} = a2*(V_{413} - V_{414} + V_{420} + V_{423})$

$f_{338} = a2*(V_{414} - V_{415} - V_{425} + V_{426})$

$f_{339} = a2*(V_{415} - V_{416})$

$f_{340} = a2*(V_{416} - V_{417})$

$f_{341} = a2*(V_{419} - V_{418} - V_{421} - V_{420})$

$f_{342} = a2*(V_{421} - V_{422})$

$f_{343} = a2*(V_{422} - V_{423})$

$f_{344} = a2*(V_{439} - V_{440})$

$f_{345} = a2*(V_{425} - V_{426} - V_{427})$

$f_{346} = a2*(V_{427} - V_{428})$

$f_{347} = a2*(V_{428} - V_{429})$

$f_{348} = a2*(V_{429} - V_{430})$

$f_{349} = a2*(V_{430} - V_{431})$

$f_{350} = a2*(V_{432} - V_{433})$

$f_{351} = a2*(V_{433} + V_{436} - V_{437} - V_{434})$

#Supplementation

$f_{352} = -0.04*X_{352}$

$f_{353} = 0.04*X_{352} - 0.04*X_{353}$

$f_{354} = -0.011*X_{354}$

$f_{355} = 0.011*X_{354}$ $.011*X_{355}$

No.	Simulation	AminoAcidAnalysis	MetabolomeAnalysis	CorrMetabolomeSimulation	CorrAminoAcidSimulation	CorrMetabolomeAminoAcid	pValMetabolomeSimulation	pValAminoAcidSimulation	pValMetabolomeAminoAcid
X_{1}	sucrose			0.000	0.000	0.000	0.000E+00	0.000E+00	0.000E+00
X_{2}	glucose-6P			0.000	0.000	0.000	0.000E+00	0.000E+00	0.000E+00
X_{3}	fructose-6P		4i_D-Fructose-6-phosphate disodium sa	0.755	0.000	0.000	5.417E-03	0.000E+00	0.000E+00
X_{4}	fructose-1,6-bis-P			0.000	0.000	0.000	0.000E+00	0.000E+00	0.000E+00
X_{5}	glyceraldehyde-3-P			0.000	0.000	0.000	0.000E+00	0.000E+00	0.000E+00
X_{6}	dihydroxyacetone-P			0.000	0.000	0.000	0.000E+00	0.000E+00	0.000E+00
X_{7}	1,3-diPglycerate			0.000	0.000	0.000	0.000E+00	0.000E+00	0.000E+00
X_{8}	3-phosphoglycerate			0.000	0.000	0.000	0.000E+00	0.000E+00	0.000E+00
X_{9}	2-phosphoglycerate			0.000	0.000	0.000	0.000E+00	0.000E+00	0.000E+00
X_{10}	phosphoenolpyruvate			0.000	0.000	0.000	0.000E+00	0.000E+00	0.000E+00
X_{11}	pyruvate			0.000	0.000	0.000	0.000E+00	0.000E+00	0.000E+00
X_{12}	2-acetolactate			0.000	0.000	0.000	0.000E+00	0.000E+00	0.000E+00
X_{13}	2,3-dihydroxyisovalerate			0.000	0.000	0.000	0.000E+00	0.000E+00	0.000E+00
X_{14}	2-ketoisovalerate			0.000	0.000	0.000	0.000E+00	0.000E+00	0.000E+00
X_{15}	2-isopropylmalate			0.000	0.000	0.000	0.000E+00	0.000E+00	0.000E+00
X_{16}	isopropylmaleate			0.000	0.000	0.000	0.000E+00	0.000E+00	0.000E+00
X_{17}	3-isopropylmalate			0.000	0.000	0.000	0.000E+00	0.000E+00	0.000E+00
X_{18}	2-isopropyl-3-oxosuccinate			0.000	0.000	0.000	0.000E+00	0.000E+00	0.000E+00
X_{19}	2-ketoisocaproate			0.000	0.000	0.000	0.000E+00	0.000E+00	0.000E+00
X_{20}	leucine	Leu	L-Leucine- (Cell Culture Reagent- Crys	-0.678	-0.751	0.654	1.982E+00	1.989E+00	1.982E+00
X_{21}	3-phosphohydroxypyruvate			0.000	0.000	0.000	0.000E+00	0.000E+00	0.000E+00
X_{22}	3-phosphoserine		o-Phospho-L-serine	-0.079	0.000	0.000	1.184E+00	0.000E+00	0.000E+00
X_{23}	Lserine	Ser	L-Serine	0.816	0.807	0.887	1.396E-03	1.624E-03	1.396E-03
X_{24}	Dserine			0.000	0.000	0.000	0.000E+00	0.000E+00	0.000E+00
X_{25}	glycine	Gly	Glycine	0.118	0.739	-0.073	7.275E-01	6.090E-01	7.275E-01
X_{26}	threonine	Thr	L-allo-threonine	0.811	0.871	0.955	1.573E-03	4.364E-04	1.573E-03
X_{27}	2-oxobutanoate			0.000	0.000	0.000	0.000E+00	0.000E+00	0.000E+00
X_{28}	2-aceto-2-hydroxybutyrate			0.000	0.000	0.000	0.000E+00	0.000E+00	0.000E+00
X_{29}	2,3-dihydroxy-3-methylvalerate			0.000	0.000	0.000	0.000E+00	0.000E+00	0.000E+00
X_{30}	2-keto-3-methylvalerate			0.000	0.000	0.000	0.000E+00	0.000E+00	0.000E+00
X_{31}	isoleucine	Ile	L-Isoleucine	-0.811	-0.889	0.784	1.998E+00	2.000E+00	1.998E+00
X_{32}	S-2-methylbutyryl-CoA			0.000	0.000	0.000	0.000E+00	0.000E+00	0.000E+00
X_{33}	tigloylCoA			0.000	0.000	0.000	0.000E+00	0.000E+00	0.000E+00
X_{34}	2-methyl-3-hydroxybutyryl-CoA			0.000	0.000	0.000	0.000E+00	0.000E+00	0.000E+00
X_{35}	2-methylacetoacetyl-CoA			0.000	0.000	0.000	0.000E+00	0.000E+00	0.000E+00
X_{36}	acetylCoA			0.000	0.000	0.000	0.000E+00	0.000E+00	0.000E+00
X_{37}	glutamate	Glu	L-Glutamic acid	0.594	-0.226	0.148	4.855E-02	9.432E-02	4.855E-02
X_{38}	N-acetyl-L-glutamate		N-Acetyl-DL-glutamic acid	0.317	0.000	0.000	3.368E-01	0.000E+00	0.000E+00
X_{39}	N-acetylglutamyl-P			0.000	0.000	0.000	0.000E+00	0.000E+00	0.000E+00
X_{40}	N-acetyl-L-glutamate-5-semialdehyde			0.000	0.000	0.000	0.000E+00	0.000E+00	0.000E+00
X_{41}	N-acetyl-L-ornithine			0.000	0.000	0.000	0.000E+00	0.000E+00	0.000E+00
X_{42}	ornithine		L-Ornithine monohydrochloride	0.550	0.000	0.000	7.409E-02	0.000E+00	0.000E+00
X_{43}	carbamoyl-P			0.000	0.000	0.000	0.000E+00	0.000E+00	0.000E+00
X_{44}	glutamine	Gln	L-Glutamine	0.736	0.427	0.464	7.632E-03	3.279E-02	7.632E-03
X_{45}	citrulline			0.000	0.000	0.000	0.000E+00	0.000E+00	0.000E+00
X_{46}	argininosuccinate			0.000	0.000	0.000	0.000E+00	0.000E+00	0.000E+00
X_{47}	arginine			0.000	0.000	0.000	0.000E+00	0.000E+00	0.000E+00
X_{48}	glutamate-gamma-semialdehyde			0.000	0.000	0.000	0.000E+00	0.000E+00	0.000E+00
X_{49}	S-1-pyrroline-5-carboxylate			0.000	0.000	0.000	0.000E+00	0.000E+00	0.000E+00
X_{50}	proline	Pro	L-Proline	0.921	0.869	0.988	2.005E-05	1.631E-04	2.005E-05
X_{51}	O-acetyl-L-serine		O-acetyl-L-serine hydrochloride	-0.735	0.000	0.000	1.992E+00	0.000E+00	0.000E+00
X_{52}	5-phosphoribosyl-1-pyrophosphate			0.000	0.000	0.000	0.000E+00	0.000E+00	0.000E+00
X_{53}	phosphoribosylATP			0.000	0.000	0.000	0.000E+00	0.000E+00	0.000E+00
X_{54}	phosphoribosylAMP			0.000	0.000	0.000	0.000E+00	0.000E+00	0.000E+00
X_{55}	phosphoribosylforniminoAICARP			0.000	0.000	0.000	0.000E+00	0.000E+00	0.000E+00
X_{56}	phosphoribulosylformiminoAICARP			0.000	0.000	0.000	0.000E+00	0.000E+00	0.000E+00
X_{57}	AICAR			0.000	0.000	0.000	0.000E+00	0.000E+00	0.000E+00
X_{58}	D-erythroimidazoleglycerol-P			0.000	0.000	0.000	0.000E+00	0.000E+00	0.000E+00
X_{59}	imidazoleacetol-P			0.000	0.000	0.000	0.000E+00	0.000E+00	0.000E+00
X_{60}	histidinolP			0.000	0.000	0.000	0.000E+00	0.000E+00	0.000E+00

X_{61}	histidinol			0.000	0.000	0.000	0.000E+00	0.000E+00	0.000E+00
X_{62}	histidinal			0.000	0.000	0.000	0.000E+00	0.000E+00	0.000E+00
X_{63}	histidine		L-Histidine	0.854	0.000	0.000	4.545E-04	0.000E+00	0.000E+00
X_{64}	citrate		Citric acid-Anhydrous	0.199	0.000	0.000	5.546E-01	0.000E+00	0.000E+00
X_{65}	cisaconitate		cis-Aconitic Acid	-0.367	0.000	0.000	1.739E+00	0.000E+00	0.000E+00
X_{66}	isocitrate		DL-Isocitric acid trisodium salt	0.095	0.000	0.000	7.810E-01	0.000E+00	0.000E+00
X_{67}	ketoglutarate			0.000	0.000	0.000	0.000E+00	0.000E+00	0.000E+00
X_{68}	succinyl-CoA			0.000	0.000	0.000	0.000E+00	0.000E+00	0.000E+00
X_{69}	succinate			0.000	0.000	0.000	0.000E+00	0.000E+00	0.000E+00
X_{70}	fumarate		Fumaric acid	-0.253	0.000	0.000	1.551E+00	0.000E+00	0.000E+00
X_{71}	malate		DL-Malic acid	0.810	0.000	0.000	1.650E-03	0.000E+00	0.000E+00
X_{72}	oxaloacetate			0.000	0.000	0.000	0.000E+00	0.000E+00	0.000E+00
X_{73}	alanine	Ala	2i_L-Alanine_Sarcosine	0.908	0.704	0.911	4.314E-05	2.768E-03	4.314E-05
X_{74}	cysteine		DL-Cysteine	0.772	0.000	0.000	3.842E-03	0.000E+00	0.000E+00
X_{75}	4-aminobutyrate		3i_L-2-Aminobutyric acid_N-N-Dimethy	-0.469	0.000	0.000	1.860E+00	0.000E+00	0.000E+00
X_{76}	succinatesemialdehyde			0.000	0.000	0.000	0.000E+00	0.000E+00	0.000E+00
X_{77}	asparagine	Asn	L-Asparagine	0.390	0.412	0.021	2.302E-01	2.256E-01	2.302E-01
X_{78}	aspartate	Asp	L-Aspartic acid	0.287	-0.690	-0.474	3.886E-01	2.601E-01	3.886E-01
X_{79}	aspartate-4P			0.000	0.000	0.000	0.000E+00	0.000E+00	0.000E+00
X_{80}	aspartatesemialdehyde			0.000	0.000	0.000	0.000E+00	0.000E+00	0.000E+00
X_{81}	homoserine			0.000	0.000	0.000	0.000E+00	0.000E+00	0.000E+00
X_{82}	O-phospho-L-homoserine			0.000	0.000	0.000	0.000E+00	0.000E+00	0.000E+00
X_{83}	cystathionine		Cystathionine	0.732	0.000	0.000	8.053E-03	0.000E+00	0.000E+00
X_{84}	homocysteine		DL-homocysteine	0.919	0.000	0.000	2.288E-05	0.000E+00	0.000E+00
X_{85}	methionine		L-Methionine	0.702	0.000	0.000	1.310E-02	0.000E+00	0.000E+00
X_{86}	S-adenosyl-L-methionine		S-(5'-Adenosyl)-L-methionine chloride	0.884	0.000	0.000	1.432E-04	0.000E+00	0.000E+00
X_{87}	S-adenosyl-L-homocysteine		S-Adenosyl-L-homocysteine	0.484	0.000	0.000	1.250E-01	0.000E+00	0.000E+00
X_{88}	adenosine		Adenosine	0.284	0.000	0.000	3.934E-01	0.000E+00	0.000E+00
X_{89}	L-2,3-dihydrodipicolinate			0.000	0.000	0.000	0.000E+00	0.000E+00	0.000E+00
X_{90}	tetrahydrodipicolinate			0.000	0.000	0.000	0.000E+00	0.000E+00	0.000E+00
X_{91}	L,L-diamionopimelate			0.000	0.000	0.000	0.000E+00	0.000E+00	0.000E+00
X_{92}	mesodiaminopimelate			0.000	0.000	0.000	0.000E+00	0.000E+00	0.000E+00
X_{93}	lysine	Lys	L-Lysine	0.881	0.991	0.902	1.633E-04	6.690E-10	1.633E-04
X_{94}	S-methyl-5-thioadenosine		5'-Deoxy-5'-Methylthioadenosine	0.704	0.000	0.000	1.271E-02	0.000E+00	0.000E+00
X_{95}	5-methythioribose			0.000	0.000	0.000	0.000E+00	0.000E+00	0.000E+00
X_{96}	5-methylthioribose-1P			0.000	0.000	0.000	0.000E+00	0.000E+00	0.000E+00
X_{97}	5-methioribulose-1P			0.000	0.000	0.000	0.000E+00	0.000E+00	0.000E+00
X_{98}	5-methylthio-2,3-dioxopentyl-P			0.000	0.000	0.000	0.000E+00	0.000E+00	0.000E+00
X_{99}	2-hydroxy-3-keto-5-methylthio-1-phosphopentene			0.000	0.000	0.000	0.000E+00	0.000E+00	0.000E+00
X_{100}	1,2-dihydroxy-3-keto-5-methylthiopentene			0.000	0.000	0.000	0.000E+00	0.000E+00	0.000E+00
X_{101}	2-oxo-4-methylthiobutanoate			0.000	0.000	0.000	0.000E+00	0.000E+00	0.000E+00
X_{102}	3-deoxyDarabinoheptulosonate-7P			0.000	0.000	0.000	0.000E+00	0.000E+00	0.000E+00
X_{103}	3-dehydroquinate			0.000	0.000	0.000	0.000E+00	0.000E+00	0.000E+00
X_{104}	3-dehydroshikimate			0.000	0.000	0.000	0.000E+00	0.000E+00	0.000E+00
X_{105}	shikimate		(-)Shikimic acid	0.955	0.000	0.000	1.069E-06	0.000E+00	0.000E+00
X_{106}	shikimate-3P			0.000	0.000	0.000	0.000E+00	0.000E+00	0.000E+00
X_{107}	5-enolpyruvylshikimate-3P			0.000	0.000	0.000	0.000E+00	0.000E+00	0.000E+00
X_{108}	prephenate			0.000	0.000	0.000	0.000E+00	0.000E+00	0.000E+00
X_{109}	arogenate			0.000	0.000	0.000	0.000E+00	0.000E+00	0.000E+00
X_{110}	phenylalanine		L-(-)-Phenylalanine	-0.810	0.000	0.000	1.998E+00	0.000E+00	0.000E+00
X_{111}	phenylpyruvate		Sodium phenylpyruvate	-0.792	0.000	0.000	1.998E+00	0.000E+00	0.000E+00
X_{112}	phenylacetaldehyde			0.000	0.000	0.000	0.000E+00	0.000E+00	0.000E+00
X_{113}	tyrosine	Tyr	L-Tyrosine	-0.175	-0.608	0.102	1.395E+00	1.477E+00	1.395E+00
X_{114}	phydroxyphenylpyruvate			0.000	0.000	0.000	0.000E+00	0.000E+00	0.000E+00
X_{115}	homogentisate			0.000	0.000	0.000	0.000E+00	0.000E+00	0.000E+00
X_{116}	maleylacetoacetate			0.000	0.000	0.000	0.000E+00	0.000E+00	0.000E+00
X_{117}	4-fumarylacetoacetate			0.000	0.000	0.000	0.000E+00	0.000E+00	0.000E+00
X_{118}	anthranilate			0.000	0.000	0.000	0.000E+00	0.000E+00	0.000E+00
X_{119}	N-5-phosphoribosylanthranilate			0.000	0.000	0.000	0.000E+00	0.000E+00	0.000E+00
X_{120}	1-O-carboxyphenylamino-1-deoxyribulose-5P			0.000	0.000	0.000	0.000E+00	0.000E+00	0.000E+00
X_{121}	indole3glycerolP			0.000	0.000	0.000	0.000E+00	0.000E+00	0.000E+00

X_{122}	indole			0.000	0.000	0.000	0.000E+00	0.000E+00	0.000E+00
X_{123}	tryptophan	Tryp	L−Tryptophane	−0.619	0.326	−0.084	1.963E+00	1.925E+00	1.963E+00
X_{124}	valine	Val	2i_L−Norvaline_L−Valine	−0.055	0.430	0.066	1.128E+00	1.141E+00	1.128E+00
X_{125}	glyoxylate			0.000	0.000	0.000	0.000E+00	0.000E+00	0.000E+00
X_{126}	SmethylLmethionine		S−Methylmethionine	0.663	0.000	0.000	2.236E−02	0.000E+00	0.000E+00
X_{127}	chorismate			0.000	0.000	0.000	0.000E+00	0.000E+00	0.000E+00
X_{128}	glutamylcysteine			0.000	0.000	0.000	0.000E+00	0.000E+00	0.000E+00
X_{129}	glutathione	GSH	Glutathione (oxidized form)	0.668	−0.193	0.055	2.081E−02	6.571E−02	2.081E−02
X_{130}	2−methylmalate			0.000	0.000	0.000	0.000E+00	0.000E+00	0.000E+00
X_{131}	methylmaleate			0.000	0.000	0.000	0.000E+00	0.000E+00	0.000E+00
X_{132}	3−methylmalate			0.000	0.000	0.000	0.000E+00	0.000E+00	0.000E+00
X_{133}	hydroxylysine			0.000	0.000	0.000	0.000E+00	0.000E+00	0.000E+00
X_{134}	acetylhydroxylysine			0.000	0.000	0.000	0.000E+00	0.000E+00	0.000E+00
X_{135}	D−lysine			0.000	0.000	0.000	0.000E+00	0.000E+00	0.000E+00
X_{136}	diaminohexanoate			0.000	0.000	0.000	0.000E+00	0.000E+00	0.000E+00
X_{137}	N−acetyllysine			0.000	0.000	0.000	0.000E+00	0.000E+00	0.000E+00
X_{138}	acetamidooxohexanoate			0.000	0.000	0.000	0.000E+00	0.000E+00	0.000E+00
X_{139}	aminooxohexanoate			0.000	0.000	0.000	0.000E+00	0.000E+00	0.000E+00
X_{140}	piperideine−2−carboxylate			0.000	0.000	0.000	0.000E+00	0.000E+00	0.000E+00
X_{141}	pipecolate		DL−Pipecolinic acid	0.971	0.000	0.000	9.817E−08	0.000E+00	0.000E+00
X_{142}	piperideine−6−carboxylate			0.000	0.000	0.000	0.000E+00	0.000E+00	0.000E+00
X_{143}	aminoadipatesemialdehyde			0.000	0.000	0.000	0.000E+00	0.000E+00	0.000E+00
X_{144}	saccharopine		L−saccharopine	0.939	0.000	0.000	5.139E−06	0.000E+00	0.000E+00
X_{145}	cadaverine			0.000	0.000	0.000	0.000E+00	0.000E+00	0.000E+00
X_{146}	piperideine			0.000	0.000	0.000	0.000E+00	0.000E+00	0.000E+00
X_{147}	aminopentanamide			0.000	0.000	0.000	0.000E+00	0.000E+00	0.000E+00
X_{148}	aminopentanoate			0.000	0.000	0.000	0.000E+00	0.000E+00	0.000E+00
X_{149}	acetyllysine			0.000	0.000	0.000	0.000E+00	0.000E+00	0.000E+00
X_{150}	acetamidopentanoate			0.000	0.000	0.000	0.000E+00	0.000E+00	0.000E+00
X_{151}	glutaratesemialdehyde			0.000	0.000	0.000	0.000E+00	0.000E+00	0.000E+00
X_{152}	glutarate		3i_Ethylmalonic acid _Glutaric acid_Meth	0.979	0.000	0.000	1.819E−08	0.000E+00	0.000E+00
X_{153}	aminoadipate		DL−2−Aminoadipic Acid	0.945	0.000	0.000	3.105E−06	0.000E+00	0.000E+00
X_{154}	oxoadipate			0.000	0.000	0.000	0.000E+00	0.000E+00	0.000E+00
X_{155}	glutaryldihydrolipoamide			0.000	0.000	0.000	0.000E+00	0.000E+00	0.000E+00
X_{156}	glutaryl−CoA			0.000	0.000	0.000	0.000E+00	0.000E+00	0.000E+00
X_{157}	crotonoyl−CoA			0.000	0.000	0.000	0.000E+00	0.000E+00	0.000E+00
X_{158}	hydroxybutanoyl−CoA			0.000	0.000	0.000	0.000E+00	0.000E+00	0.000E+00
X_{159}	acetoacetyl−CoA			0.000	0.000	0.000	0.000E+00	0.000E+00	0.000E+00
X_{160}	2,2−methylthioethylmalate			0.000	0.000	0.000	0.000E+00	0.000E+00	0.000E+00
X_{161}	3,2−methylthioethylmalate			0.000	0.000	0.000	0.000E+00	0.000E+00	0.000E+00
X_{162}	2−oxo−5−methylthiopentanoicacid			0.000	0.000	0.000	0.000E+00	0.000E+00	0.000E+00
X_{163}	2,3−methylthiopropylmalate			0.000	0.000	0.000	0.000E+00	0.000E+00	0.000E+00
X_{164}	3,3−methylthiopropylmalte			0.000	0.000	0.000	0.000E+00	0.000E+00	0.000E+00
X_{165}	2−oxo−6−methylthiohexanoate			0.000	0.000	0.000	0.000E+00	0.000E+00	0.000E+00
X_{166}	2,4−methylthiobutylmalate			0.000	0.000	0.000	0.000E+00	0.000E+00	0.000E+00
X_{167}	3,4−methylthiobutylmalate			0.000	0.000	0.000	0.000E+00	0.000E+00	0.000E+00
X_{168}	2−oxo−7−methylthioheptanoate			0.000	0.000	0.000	0.000E+00	0.000E+00	0.000E+00
X_{169}	2,5−methylthiopentylmalate			0.000	0.000	0.000	0.000E+00	0.000E+00	0.000E+00
X_{170}	3,5−methylthiobutylmalate			0.000	0.000	0.000	0.000E+00	0.000E+00	0.000E+00
X_{171}	2−oxo−8−methyloctanoate			0.000	0.000	0.000	0.000E+00	0.000E+00	0.000E+00
X_{172}	2,6−methylthiohexylmalate			0.000	0.000	0.000	0.000E+00	0.000E+00	0.000E+00
X_{173}	3,6−methylthiohexylmalate			0.000	0.000	0.000	0.000E+00	0.000E+00	0.000E+00
X_{174}	2−oxo−9−methylononanoate			0.000	0.000	0.000	0.000E+00	0.000E+00	0.000E+00
X_{175}	2,7−methylthioheptylmalate			0.000	0.000	0.000	0.000E+00	0.000E+00	0.000E+00
X_{176}	3,7−methylthioheptylmalate			0.000	0.000	0.000	0.000E+00	0.000E+00	0.000E+00
X_{177}	2−oxo−1,0−methylthiodecanoate			0.000	0.000	0.000	0.000E+00	0.000E+00	0.000E+00
X_{178}	homomethionine		L−beta−homomethionine−HCl	−0.180	0.000	0.000	1.407E+00	0.000E+00	0.000E+00
X_{179}	methylthiobutanaldoxime			0.000	0.000	0.000	0.000E+00	0.000E+00	0.000E+00
X_{180}	methylthiobutanonitrileoxide			0.000	0.000	0.000	0.000E+00	0.000E+00	0.000E+00
X_{181}	methylthiobutylhydroximoylglutathione			0.000	0.000	0.000	0.000E+00	0.000E+00	0.000E+00
X_{182}	methylthiobutylhydroximoylcysteinylglycine			0.000	0.000	0.000	0.000E+00	0.000E+00	0.000E+00

X_{183}	methylthiobutylhydroximate			0.000	0.000	0.000	0.000E+00	0.000E+00	0.000E+00
X_{184}	methylthiopropyldesulfoglucosinolate			0.000	0.000	0.000	0.000E+00	0.000E+00	0.000E+00
X_{185}	methylthiopropylglucosinolate		3-Methylthio-n-propyl-glucosinolate	0.093	0.000	0.000	7.853E-01	0.000E+00	0.000E+00
X_{186}	methylsulfinylpropylglucosinolate		3-Methylsulfinyl-n-propyl-glucosinolate	0.196	0.000	0.000	5.618E-01	0.000E+00	0.000E+00
X_{187}	propernylglucosinolate			0.000	0.000	0.000	0.000E+00	0.000E+00	0.000E+00
X_{188}	hydroxypropylglucosinolate			0.000	0.000	0.000	0.000E+00	0.000E+00	0.000E+00
X_{189}	benzoyloxypropylglucosinolate		3-Benzoyloxy-n-propyl-glucosinolate	0.008	0.000	0.000	9.806E-01	0.000E+00	0.000E+00
X_{190}	sinapoyloxypropylglucosinolate			0.000	0.000	0.000	0.000E+00	0.000E+00	0.000E+00
X_{191}	dihomomethionine			0.000	0.000	0.000	0.000E+00	0.000E+00	0.000E+00
X_{192}	methylthiopentanaldoxime			0.000	0.000	0.000	0.000E+00	0.000E+00	0.000E+00
X_{193}	methylthiopentanonitrileoxide			0.000	0.000	0.000	0.000E+00	0.000E+00	0.000E+00
X_{194}	methylthiopentylhydroximoylglutathione			0.000	0.000	0.000	0.000E+00	0.000E+00	0.000E+00
X_{195}	methylthiopentylhydroximoylcysteinylglycine			0.000	0.000	0.000	0.000E+00	0.000E+00	0.000E+00
X_{196}	methylthiopentylhydroximate			0.000	0.000	0.000	0.000E+00	0.000E+00	0.000E+00
X_{197}	methylthiobutyldesulfoglucosinolate			0.000	0.000	0.000	0.000E+00	0.000E+00	0.000E+00
X_{198}	methylthiobutylglucosinolate		4-Methylthio-n-butyl-glucosinolate	0.021	0.000	0.000	9.502E-01	0.000E+00	0.000E+00
X_{199}	methylsulfinylbutylglucosinolate		4-Methylsulfinyl-n-butyl-glucosinolate	-0.035	0.000	0.000	1.081E+00	0.000E+00	0.000E+00
X_{200}	butenylglucosinolate			0.000	0.000	0.000	0.000E+00	0.000E+00	0.000E+00
X_{201}	hydroxy-3-butenylglucosinolate			0.000	0.000	0.000	0.000E+00	0.000E+00	0.000E+00
X_{202}	benzoyloxy-3-butenylglucosinolate			0.000	0.000	0.000	0.000E+00	0.000E+00	0.000E+00
X_{203}	sinapoyloxy-3-butenylglucosinolate			0.000	0.000	0.000	0.000E+00	0.000E+00	0.000E+00
X_{204}	hydroxybutylglucosinolate			0.000	0.000	0.000	0.000E+00	0.000E+00	0.000E+00
X_{205}	benzoyloxybutylglucosinolate			0.000	0.000	0.000	0.000E+00	0.000E+00	0.000E+00
X_{206}	sinapoyloxybutylglucosinolate			0.000	0.000	0.000	0.000E+00	0.000E+00	0.000E+00
X_{207}	trihomomethionine			0.000	0.000	0.000	0.000E+00	0.000E+00	0.000E+00
X_{208}	methylthiohexanaldoxime			0.000	0.000	0.000	0.000E+00	0.000E+00	0.000E+00
X_{209}	methylthiohexanonitrildoxide			0.000	0.000	0.000	0.000E+00	0.000E+00	0.000E+00
X_{210}	methylthiohexylhydroximoylglutathione			0.000	0.000	0.000	0.000E+00	0.000E+00	0.000E+00
X_{211}	methylthiohexylhydroximoylcysteinylglycine			0.000	0.000	0.000	0.000E+00	0.000E+00	0.000E+00
X_{212}	methylthiohexylhydroximate			0.000	0.000	0.000	0.000E+00	0.000E+00	0.000E+00
X_{213}	methylthiopentyldesulfoglucosinolate			0.000	0.000	0.000	0.000E+00	0.000E+00	0.000E+00
X_{214}	methylthiopentylglusinolate		5-Methylthio-n-pentyl-glucosinolate	-0.060	0.000	0.000	1.139E+00	0.000E+00	0.000E+00
X_{215}	methylsulfinylpentylglucosinolate		5-Methylsulfinyl-n-pentyl-glucosinolate	0.125	0.000	0.000	7.136E-01	0.000E+00	0.000E+00
X_{216}	pentenylglucosinolate			0.000	0.000	0.000	0.000E+00	0.000E+00	0.000E+00
X_{217}	tetrahomomethionine			0.000	0.000	0.000	0.000E+00	0.000E+00	0.000E+00
X_{218}	methylthioheptanaldoxime			0.000	0.000	0.000	0.000E+00	0.000E+00	0.000E+00
X_{219}	methylthioheptanonitrileoxide			0.000	0.000	0.000	0.000E+00	0.000E+00	0.000E+00
X_{220}	methylthioheptylhydroximoylglutathione			0.000	0.000	0.000	0.000E+00	0.000E+00	0.000E+00
X_{221}	methylthioheptylhydroximoylcysteinylglycine			0.000	0.000	0.000	0.000E+00	0.000E+00	0.000E+00
X_{222}	methylthioheptylhydroximate			0.000	0.000	0.000	0.000E+00	0.000E+00	0.000E+00
X_{223}	methylthiohexyldesulfoglucosinolate			0.000	0.000	0.000	0.000E+00	0.000E+00	0.000E+00
X_{224}	methylthiohexylglucosinolate		6-Methylthio-n-hexyl-glucosinolate	-0.058	0.000	0.000	1.136E+00	0.000E+00	0.000E+00
X_{225}	methylsulfinylhexylglucosinolate		6-Methylsulfinyl-n-hexyl-glucosinolate	0.001	0.000	0.000	9.972E-01	0.000E+00	0.000E+00
X_{226}	pentahomomethionine			0.000	0.000	0.000	0.000E+00	0.000E+00	0.000E+00
X_{227}	methylthiooctanaldoxime			0.000	0.000	0.000	0.000E+00	0.000E+00	0.000E+00
X_{228}	methylthiooctanonitrileoxide			0.000	0.000	0.000	0.000E+00	0.000E+00	0.000E+00
X_{229}	methylthiooctylhydroximoylglutathione			0.000	0.000	0.000	0.000E+00	0.000E+00	0.000E+00
X_{230}	methylthiooctylhydroximoylcysteinylglycine			0.000	0.000	0.000	0.000E+00	0.000E+00	0.000E+00
X_{231}	methylthiooctylhydroximate			0.000	0.000	0.000	0.000E+00	0.000E+00	0.000E+00
X_{232}	methylthioheptyldesulfoglucosinolate			0.000	0.000	0.000	0.000E+00	0.000E+00	0.000E+00
X_{233}	methylthioheptylglucosinolate		7-Methylthio-n-heptyl-glucosinolate	-0.051	0.000	0.000	1.119E+00	0.000E+00	0.000E+00
X_{234}	methylsulfinylheptylglucosinolate		7-Methylsulfinyl-n-heptyl-glucosinolate	0.306	0.000	0.000	3.553E-01	0.000E+00	0.000E+00
X_{235}	hexahomomethionine			0.000	0.000	0.000	0.000E+00	0.000E+00	0.000E+00
X_{236}	methylthiononanaldoxime			0.000	0.000	0.000	0.000E+00	0.000E+00	0.000E+00
X_{237}	methylthiononanonitrileoxide			0.000	0.000	0.000	0.000E+00	0.000E+00	0.000E+00
X_{238}	methylthiononylhydroximoylglutathionine			0.000	0.000	0.000	0.000E+00	0.000E+00	0.000E+00
X_{239}	methylthiononylhydroximoylcysteinylglycine			0.000	0.000	0.000	0.000E+00	0.000E+00	0.000E+00
X_{240}	methylthiononylhydroximate			0.000	0.000	0.000	0.000E+00	0.000E+00	0.000E+00
X_{241}	methylthiooctyldesulfoglucosinolate			0.000	0.000	0.000	0.000E+00	0.000E+00	0.000E+00
X_{242}	methylthiooctylglucosinolate		8-Methylthio-n-octyl-glucosinolate	-0.061	0.000	0.000	1.143E+00	0.000E+00	0.000E+00
X_{243}	methylsulfinyloctylglucosinolate		8-Methylsulfinyl-n-octyl-glucosinolate	0.409	0.000	0.000	2.058E-01	0.000E+00	0.000E+00

X_{244}	phenylacetaldoxime			0.000	0.000	0.000	0.000E+00	0.000E+00	0.000E+00
X_{245}	phenylacetonitrileoxide			0.000	0.000	0.000	0.000E+00	0.000E+00	0.000E+00
X_{246}	phenylacetohydroximoylglutathione			0.000	0.000	0.000	0.000E+00	0.000E+00	0.000E+00
X_{247}	phenylacetohydroximoylcysteinylglycine			0.000	0.000	0.000	0.000E+00	0.000E+00	0.000E+00
X_{248}	phenylacetothiohydroximate			0.000	0.000	0.000	0.000E+00	0.000E+00	0.000E+00
X_{249}	benzyldesulfoglucosinolate			0.000	0.000	0.000	0.000E+00	0.000E+00	0.000E+00
X_{250}	benzylglucosinolate			0.000	0.000	0.000	0.000E+00	0.000E+00	0.000E+00
X_{251}	indole-3-acetaldoxime			0.000	0.000	0.000	0.000E+00	0.000E+00	0.000E+00
X_{252}	indole-3-acetonitrileoxide			0.000	0.000	0.000	0.000E+00	0.000E+00	0.000E+00
X_{253}	indole-3-acetohydroximoylglutathione			0.000	0.000	0.000	0.000E+00	0.000E+00	0.000E+00
X_{254}	indole-3-acetohydroximoylcysteinylglycine			0.000	0.000	0.000	0.000E+00	0.000E+00	0.000E+00
X_{255}	indolylmethylthiohydroximate			0.000	0.000	0.000	0.000E+00	0.000E+00	0.000E+00
X_{256}	indolylmethyldesulfoglucosinolate			0.000	0.000	0.000	0.000E+00	0.000E+00	0.000E+00
X_{257}	indol-3-ylmethylglucosinolate		Indol-3-ylmethyl-glucosinolate	-0.615	0.000	0.000	1.961E+00	0.000E+00	0.000E+00
X_{258}	1-hydroxy-3-indolylmethylglucosinolate			0.000	0.000	0.000	0.000E+00	0.000E+00	0.000E+00
X_{259}	1-methoxy-3-indolylmethylglucosinolate		1-Methoxyindole-glucosinolate	-0.112	0.000	0.000	1.259E+00	0.000E+00	0.000E+00
X_{260}	4-hydroxy-3-indolylmethylglucosinolate			0.000	0.000	0.000	0.000E+00	0.000E+00	0.000E+00
X_{261}	4-methoxy-3-indolylmethylglucosinolate		4-Methoxyindole-glucosinolate	0.852	0.000	0.000	4.842E-04	0.000E+00	0.000E+00
X_{262}	histamine			0.000	0.000	0.000	0.000E+00	0.000E+00	0.000E+00
X_{263}	imidazoleacetaldehyde			0.000	0.000	0.000	0.000E+00	0.000E+00	0.000E+00
X_{264}	imidazole4acetate			0.000	0.000	0.000	0.000E+00	0.000E+00	0.000E+00
X_{265}	imidazoloneacetate			0.000	0.000	0.000	0.000E+00	0.000E+00	0.000E+00
X_{266}	NformiminoLaspartate			0.000	0.000	0.000	0.000E+00	0.000E+00	0.000E+00
X_{267}	NformylLaspartate			0.000	0.000	0.000	0.000E+00	0.000E+00	0.000E+00
X_{268}	D-glucono-1,5-lactone-6P			0.000	0.000	0.000	0.000E+00	0.000E+00	0.000E+00
X_{269}	6-phospho-D-gluconate			0.000	0.000	0.000	0.000E+00	0.000E+00	0.000E+00
X_{270}	D-ribulose-5P			0.000	0.000	0.000	0.000E+00	0.000E+00	0.000E+00
X_{271}	D-ribose-5P		D-Ribose-5-phosphate disodium salt hy	0.341	0.000	0.000	2.994E-01	0.000E+00	0.000E+00
X_{272}	D-ribose			0.000	0.000	0.000	0.000E+00	0.000E+00	0.000E+00
X_{273}	D-ribose-1P			0.000	0.000	0.000	0.000E+00	0.000E+00	0.000E+00
X_{274}	D-ribose-1,5P			0.000	0.000	0.000	0.000E+00	0.000E+00	0.000E+00
X_{275}	D-xylulose-5P			0.000	0.000	0.000	0.000E+00	0.000E+00	0.000E+00
X_{276}	D-erythrose-4P		D-Erythrose-4-phosphate sodium salt	0.717	0.000	0.000	1.035E-02	0.000E+00	0.000E+00
X_{277}	D-sedoheptulose-7P			0.000	0.000	0.000	0.000E+00	0.000E+00	0.000E+00
X_{278}	D-gluconate			0.000	0.000	0.000	0.000E+00	0.000E+00	0.000E+00
X_{279}	D-glucono-1,5-lactone			0.000	0.000	0.000	0.000E+00	0.000E+00	0.000E+00
X_{280}	2-dehydro-D-gluconate			0.000	0.000	0.000	0.000E+00	0.000E+00	0.000E+00
X_{281}	2-dehydrogluconate-6P			0.000	0.000	0.000	0.000E+00	0.000E+00	0.000E+00
X_{282}	5-phospho-B-D-ribosylamine			0.000	0.000	0.000	0.000E+00	0.000E+00	0.000E+00
X_{283}	5-phosphoribosylglycineamine			0.000	0.000	0.000	0.000E+00	0.000E+00	0.000E+00
X_{284}	5-phosphoribosyl-N-formylglycineamide			0.000	0.000	0.000	0.000E+00	0.000E+00	0.000E+00
X_{285}	5-phosphoribosyl-N-formylglycineamidine			0.000	0.000	0.000	0.000E+00	0.000E+00	0.000E+00
X_{286}	5-aminoimidazoleribonucleotide			0.000	0.000	0.000	0.000E+00	0.000E+00	0.000E+00
X_{287}	4-carboxyaminoimidazoleribonucleotide			0.000	0.000	0.000	0.000E+00	0.000E+00	0.000E+00
X_{288}	5-phosphoribosyl-4-N-succinocarboxamide-5-aminoimidazole			0.000	0.000	0.000	0.000E+00	0.000E+00	0.000E+00
X_{289}	phosphoribosylformamidocarboxamide			0.000	0.000	0.000	0.000E+00	0.000E+00	0.000E+00
X_{290}	inosine-5-phosphate		Inosine-5'-monophosphate	0.517	0.000	0.000	9.751E-02	0.000E+00	0.000E+00
X_{291}	adenylosuccinate			0.000	0.000	0.000	0.000E+00	0.000E+00	0.000E+00
X_{292}	xanthosine-5-phosphate			0.000	0.000	0.000	0.000E+00	0.000E+00	0.000E+00
X_{293}	AMP		Adenosine-5'-monophosphate sodium s	0.596	0.000	0.000	4.763E-02	0.000E+00	0.000E+00
X_{294}	GMP		Guanosine-3'-5'-cyclic monophosphate	-0.259	0.000	0.000	1.561E+00	0.000E+00	0.000E+00
X_{295}	inosine		Inosine	0.242	0.000	0.000	4.706E-01	0.000E+00	0.000E+00
X_{296}	hypoxanthine		Hypoxanthine	0.048	0.000	0.000	8.882E-01	0.000E+00	0.000E+00
X_{297}	xanthosine			0.000	0.000	0.000	0.000E+00	0.000E+00	0.000E+00
X_{298}	guanine		Guanine	0.797	0.000	0.000	2.242E-03	0.000E+00	0.000E+00
X_{299}	xanthine		Xanthine	0.895	0.000	0.000	8.825E-05	0.000E+00	0.000E+00
X_{300}	urate			0.000	0.000	0.000	0.000E+00	0.000E+00	0.000E+00
X_{301}	5-hydroxyisourate			0.000	0.000	0.000	0.000E+00	0.000E+00	0.000E+00
X_{302}	5-hydroxy-2-oxo-4-ureido-2,5-dihydro-1H-imidazole-5-carboxylate			0.000	0.000	0.000	0.000E+00	0.000E+00	0.000E+00
X_{303}	S-allantoin		Allantoin	0.737	0.000	0.000	7.461E-03	0.000E+00	0.000E+00
X_{304}	allantoate		Allantoic acid	0.060	0.000	0.000	8.611E-01	0.000E+00	0.000E+00

X_{305}	ureidoglycine			0.000	0.000	0.000	0.000E+00	0.000E+00	0.000E+00
X_{306}	ureidoglycolate			0.000	0.000	0.000	0.000E+00	0.000E+00	0.000E+00
X_{307}	urea			0.000	0.000	0.000	0.000E+00	0.000E+00	0.000E+00
X_{308}	GDP			0.000	0.000	0.000	0.000E+00	0.000E+00	0.000E+00
X_{309}	GTP			0.000	0.000	0.000	0.000E+00	0.000E+00	0.000E+00
X_{310}	dGDP			0.000	0.000	0.000	0.000E+00	0.000E+00	0.000E+00
X_{311}	dGTP			0.000	0.000	0.000	0.000E+00	0.000E+00	0.000E+00
X_{312}	ADP			0.000	0.000	0.000	0.000E+00	0.000E+00	0.000E+00
X_{313}	dADP			0.000	0.000	0.000	0.000E+00	0.000E+00	0.000E+00
X_{314}	ATP			0.000	0.000	0.000	0.000E+00	0.000E+00	0.000E+00
X_{315}	dATP			0.000	0.000	0.000	0.000E+00	0.000E+00	0.000E+00
X_{316}	dAMP		2'-Deoxyadenosine-5'-monophosphate	0.089	0.000	0.000	7.943E-01	0.000E+00	0.000E+00
X_{317}	deoxyadenosine		2'-Deoxyadenosine monohydrate	0.250	0.000	0.000	4.548E-01	0.000E+00	0.000E+00
X_{318}	deoxyinosine			0.000	0.000	0.000	0.000E+00	0.000E+00	0.000E+00
X_{319}	adenine		Adenine	0.183	0.000	0.000	5.879E-01	0.000E+00	0.000E+00
X_{320}	guanosine		Guanosine	-0.126	0.000	0.000	1.290E+00	0.000E+00	0.000E+00
X_{321}	NcarbamoylLaspartate		Carbamoyl-DL-aspartic acid	-0.583	0.000	0.000	1.946E+00	0.000E+00	0.000E+00
X_{322}	dihydroorotate			0.000	0.000	0.000	0.000E+00	0.000E+00	0.000E+00
X_{323}	orotate			0.000	0.000	0.000	0.000E+00	0.000E+00	0.000E+00
X_{324}	orotidine-5P			0.000	0.000	0.000	0.000E+00	0.000E+00	0.000E+00
X_{325}	UMP		Uridine-5'-monophosphate	0.218	0.000	0.000	5.166E-01	0.000E+00	0.000E+00
X_{326}	UDP			0.000	0.000	0.000	0.000E+00	0.000E+00	0.000E+00
X_{327}	UTP			0.000	0.000	0.000	0.000E+00	0.000E+00	0.000E+00
X_{328}	CTP			0.000	0.000	0.000	0.000E+00	0.000E+00	0.000E+00
X_{329}	CMP		Cytidine-3'-monophosphate	0.346	0.000	0.000	2.926E-01	0.000E+00	0.000E+00
X_{330}	CDP			0.000	0.000	0.000	0.000E+00	0.000E+00	0.000E+00
X_{331}	dCDP			0.000	0.000	0.000	0.000E+00	0.000E+00	0.000E+00
X_{332}	dCTP			0.000	0.000	0.000	0.000E+00	0.000E+00	0.000E+00
X_{333}	dihydrouracil		5-6-Dihydrouracil	0.924	0.000	0.000	1.676E-05	0.000E+00	0.000E+00
X_{334}	dUDP			0.000	0.000	0.000	0.000E+00	0.000E+00	0.000E+00
X_{335}	uracil			0.000	0.000	0.000	0.000E+00	0.000E+00	0.000E+00
X_{336}	dUTP			0.000	0.000	0.000	0.000E+00	0.000E+00	0.000E+00
X_{337}	dUMP			0.000	0.000	0.000	0.000E+00	0.000E+00	0.000E+00
X_{338}	dTMP			0.000	0.000	0.000	0.000E+00	0.000E+00	0.000E+00
X_{339}	dTDP			0.000	0.000	0.000	0.000E+00	0.000E+00	0.000E+00
X_{340}	dTTP			0.000	0.000	0.000	0.000E+00	0.000E+00	0.000E+00
X_{341}	dCMP			0.000	0.000	0.000	0.000E+00	0.000E+00	0.000E+00
X_{342}	deoxycytidine		2'-Deoxycytidine	0.215	0.000	0.000	5.234E-01	0.000E+00	0.000E+00
X_{343}	deoxyuridine		(+)-2'-Deoxyuridine	0.698	0.000	0.000	1.374E-02	0.000E+00	0.000E+00
X_{344}	3-ureidopropionate		3-ureidopropionic acid	0.824	0.000	0.000	1.140E-03	0.000E+00	0.000E+00
X_{345}	thymidine		Thymidine	0.815	0.000	0.000	1.447E-03	0.000E+00	0.000E+00
X_{346}	thymine			0.000	0.000	0.000	0.000E+00	0.000E+00	0.000E+00
X_{347}	dihydrothymine			0.000	0.000	0.000	0.000E+00	0.000E+00	0.000E+00
X_{348}	3-ureidoisobutyrate			0.000	0.000	0.000	0.000E+00	0.000E+00	0.000E+00
X_{349}	3-aminoisobutanoate			0.000	0.000	0.000	0.000E+00	0.000E+00	0.000E+00
X_{350}	cytidine		Cytidine-cell culture tested	-0.232	0.000	0.000	1.512E+00	0.000E+00	0.000E+00
X_{351}	uridine		Uridine	-0.715	0.000	0.000	1.989E+00	0.000E+00	0.000E+00
X_{352}			(-)-Norepinephrine	0.000	0.000	0.000	0.000E+00	0.000E+00	0.000E+00
X_{353}			(-)-Riboflavin	0.000	0.000	0.000	0.000E+00	0.000E+00	0.000E+00
X_{354}			(+-)-Jasmonic acid	0.000	0.000	0.000	0.000E+00	0.000E+00	0.000E+00
X_{355}			(2R)-2-Hydroxy-2-phenethylglucosinola	0.000	0.000	0.000	0.000E+00	0.000E+00	0.000E+00
X_{356}			10-camphorsulfonic acid	0.000	0.000	0.000	0.000E+00	0.000E+00	0.000E+00
X_{357}			1-1-Dimethylbiguanide hydrochloride	0.000	0.000	0.000	0.000E+00	0.000E+00	0.000E+00
X_{358}			11i_D-(-)-Fructose_D-(+)-Galactose_D-	0.000	0.000	0.000	0.000E+00	0.000E+00	0.000E+00
X_{359}			1-Amino-1-cyclopentanecarboxylic acid	0.000	0.000	0.000	0.000E+00	0.000E+00	0.000E+00
X_{360}			1-Aminocyclopropane-1-carboxylic acid	0.000	0.000	0.000	0.000E+00	0.000E+00	0.000E+00
X_{361}			1-Isothiocyanato-6-(methylsulfinyl)-hex	0.000	0.000	0.000	0.000E+00	0.000E+00	0.000E+00
X_{362}			1-Isothiocyanato-6-(methylsulfonyl)-he	0.000	0.000	0.000	0.000E+00	0.000E+00	0.000E+00
X_{363}			1-Methylguanidine hydrochloride	0.000	0.000	0.000	0.000E+00	0.000E+00	0.000E+00
X_{364}			1-Methylhistamine dihydrochloride	0.000	0.000	0.000	0.000E+00	0.000E+00	0.000E+00
X_{365}			1-O-b-D-glucopyranosyl sinapate	0.000	0.000	0.000	0.000E+00	0.000E+00	0.000E+00

X_{366}			2-Aminoethylphosphonic acid	0.000	0.000	0.000	0.000E+00	0.000E+00	0.000E+00
X_{367}			2'-Deoxyguanosine monohydrate	0.000	0.000	0.000	0.000E+00	0.000E+00	0.000E+00
X_{368}			2-Deoxyribose-5-phosphate sodium sal	0.000	0.000	0.000	0.000E+00	0.000E+00	0.000E+00
X_{369}			2i_1-3-Diaminopropane dihydrochloride_	0.000	0.000	0.000	0.000E+00	0.000E+00	0.000E+00
X_{370}			2i_Adipic acid_2-Methylglutaric Acid	0.000	0.000	0.000	0.000E+00	0.000E+00	0.000E+00
X_{371}			2i_alpha-Ketoglutaric acid disodium salt_	0.000	0.000	0.000	0.000E+00	0.000E+00	0.000E+00
X_{372}			2i_-alpha-Lipoamide_DL-Thioctamide	0.000	0.000	0.000	0.000E+00	0.000E+00	0.000E+00
X_{373}			2i_cyanidin-3-5-di-O-glucoside chloride	0.000	0.000	0.000	0.000E+00	0.000E+00	0.000E+00
X_{374}			2i_D-(-)-Ribose_D-Xylulose	0.000	0.000	0.000	0.000E+00	0.000E+00	0.000E+00
X_{375}			2i_D-(+)-Cellobiose_Lactulose	0.000	0.000	0.000	0.000E+00	0.000E+00	0.000E+00
X_{376}			2i_D-(+)-Cellobiose_Lactulose	0.000	0.000	0.000	0.000E+00	0.000E+00	0.000E+00
X_{377}			2i_D-(+)-Melezitose monohydrate_1-Kes	0.000	0.000	0.000	0.000E+00	0.000E+00	0.000E+00
X_{378}			2i_D-Mannose-6-phosphate barium salt	0.000	0.000	0.000	0.000E+00	0.000E+00	0.000E+00
X_{379}			2i_eriodictyol-7-O-glucoside_Marein	0.000	0.000	0.000	0.000E+00	0.000E+00	0.000E+00
X_{380}			2i_Ideain chloride_cyanidin-3-glucoside c	0.000	0.000	0.000	0.000E+00	0.000E+00	0.000E+00
X_{381}			2i_Indole-3-carboxyaldehyde_Indole-3-a	0.000	0.000	0.000	0.000E+00	0.000E+00	0.000E+00
X_{382}			2i_Kaempferol-3-Glucoside-2-Rhamnos	0.000	0.000	0.000	0.000E+00	0.000E+00	0.000E+00
X_{383}			2i_L-Norleucine_D-Alloisoleucine	0.000	0.000	0.000	0.000E+00	0.000E+00	0.000E+00
X_{384}			2i_Maritimein_luteolin-7-O-glucoside	0.000	0.000	0.000	0.000E+00	0.000E+00	0.000E+00
X_{385}			2i_Nicotinic Acid_Isonicotinic acid	0.000	0.000	0.000	0.000E+00	0.000E+00	0.000E+00
X_{386}			2i_Quercitrin_Quercetin-7-O-rhamnosid	0.000	0.000	0.000	0.000E+00	0.000E+00	0.000E+00
X_{387}			2i_Rutin _Quercetin-3-O-b-glucopyrano	0.000	0.000	0.000	0.000E+00	0.000E+00	0.000E+00
X_{388}			2i_Sarsapogenine_Smilagenin	0.000	0.000	0.000	0.000E+00	0.000E+00	0.000E+00
X_{389}			2i_Theophylline-anhydrous_1-7-Dimethy	0.000	0.000	0.000	0.000E+00	0.000E+00	0.000E+00
X_{390}			2i_Uridine-5'-diphospho-N-acetylgalact	0.000	0.000	0.000	0.000E+00	0.000E+00	0.000E+00
X_{391}			2-Isopropylmalic acid	0.000	0.000	0.000	0.000E+00	0.000E+00	0.000E+00
X_{392}			3-5-Dimethoxycinnamic acid (predomina	0.000	0.000	0.000	0.000E+00	0.000E+00	0.000E+00
X_{393}			3-Chloro-L-tyrosine hydrochloride	0.000	0.000	0.000	0.000E+00	0.000E+00	0.000E+00
X_{394}			3-cyanopyridine	0.000	0.000	0.000	0.000E+00	0.000E+00	0.000E+00
X_{395}			3'-Dephosphocoenzyme A	0.000	0.000	0.000	0.000E+00	0.000E+00	0.000E+00
X_{396}			3-hydroxy-3-methylbutanoic acid	0.000	0.000	0.000	0.000E+00	0.000E+00	0.000E+00
X_{397}			3-Hydroxy-3-methylglutarate	0.000	0.000	0.000	0.000E+00	0.000E+00	0.000E+00
X_{398}			3i_Glycolaldehyde dimer-mixture of ster	0.000	0.000	0.000	0.000E+00	0.000E+00	0.000E+00
X_{399}			3i_Isorhamnetin-3-Glucoside-6-Rhamno	0.000	0.000	0.000	0.000E+00	0.000E+00	0.000E+00
X_{400}			3i_L-Iditol_D-Sorbitol_D-(-)-Mannitol	0.000	0.000	0.000	0.000E+00	0.000E+00	0.000E+00
X_{401}			3i_L-Threonine_-Methyl-DL-serine_L-H	0.000	0.000	0.000	0.000E+00	0.000E+00	0.000E+00
X_{402}			3i_Sodium pantothenate_D-Pantothenic	0.000	0.000	0.000	0.000E+00	0.000E+00	0.000E+00
X_{403}			3i_Uridine-5'-diphosphogalactose disodiu	0.000	0.000	0.000	0.000E+00	0.000E+00	0.000E+00
X_{404}			3-Methyl-L-histidine	0.000	0.000	0.000	0.000E+00	0.000E+00	0.000E+00
X_{405}			4-(Methylsulfinyl)but-3-enylglucosinolat	0.000	0.000	0.000	0.000E+00	0.000E+00	0.000E+00
X_{406}			4-Hydroxybenzoate	0.000	0.000	0.000	0.000E+00	0.000E+00	0.000E+00
X_{407}			4i_Kaempferol-3-Rhamnoside-7-Rhamn	0.000	0.000	0.000	0.000E+00	0.000E+00	0.000E+00
X_{408}			4i_L(+)-ArginineJHCl_Nalpha-Acetyl-L-	0.000	0.000	0.000	0.000E+00	0.000E+00	0.000E+00
X_{409}			4-Methyl-5-thiazoleethanol	0.000	0.000	0.000	0.000E+00	0.000E+00	0.000E+00
X_{410}			4-Nitrophenol	0.000	0.000	0.000	0.000E+00	0.000E+00	0.000E+00
X_{411}			4-Pyridoxate	0.000	0.000	0.000	0.000E+00	0.000E+00	0.000E+00
X_{412}			5-Aminovaleric acid	0.000	0.000	0.000	0.000E+00	0.000E+00	0.000E+00
X_{413}			5-Hydroxyindole-3-acetate (5-HIAA)	0.000	0.000	0.000	0.000E+00	0.000E+00	0.000E+00
X_{414}			5-Methylcytosine hydrochloride	0.000	0.000	0.000	0.000E+00	0.000E+00	0.000E+00
X_{415}			6-Aminohexanoic acid	0.000	0.000	0.000	0.000E+00	0.000E+00	0.000E+00
X_{416}			6i_Melibiose hydrate_D-(+)-Turanose_Isc	0.000	0.000	0.000	0.000E+00	0.000E+00	0.000E+00
X_{417}			7i_kaempferol-3-O-rutinoside	0.000	0.000	0.000	0.000E+00	0.000E+00	0.000E+00
X_{418}			Acetaminophen	0.000	0.000	0.000	0.000E+00	0.000E+00	0.000E+00
X_{419}			Acetylcholine chloride	0.000	0.000	0.000	0.000E+00	0.000E+00	0.000E+00
X_{420}			Adenosine 3'-monophosphate From Yea	0.000	0.000	0.000	0.000E+00	0.000E+00	0.000E+00
X_{421}			Adenosine-5'-phosphosulfate sodium sa	0.000	0.000	0.000	0.000E+00	0.000E+00	0.000E+00
X_{422}			Agmatine sulfate salt	0.000	0.000	0.000	0.000E+00	0.000E+00	0.000E+00
X_{423}			alpha-Lactose monohydrate	0.000	0.000	0.000	0.000E+00	0.000E+00	0.000E+00
X_{424}			alpha-Methyl-DL-histidine dihydrochlori	0.000	0.000	0.000	0.000E+00	0.000E+00	0.000E+00
X_{425}			alpha-Tocotrienol	0.000	0.000	0.000	0.000E+00	0.000E+00	0.000E+00
X_{426}			Amantadine hydrochloride	0.000	0.000	0.000	0.000E+00	0.000E+00	0.000E+00

X_{427}			Benzylglucosinolate	0.000	0.000	0.000	0.000E+00	0.000E+00	0.000E+00
X_{428}			Betaine	0.000	0.000	0.000	0.000E+00	0.000E+00	0.000E+00
X_{429}			beta-Nicotinamide adenine dinucleotide	0.000	0.000	0.000	0.000E+00	0.000E+00	0.000E+00
X_{430}			beta-Nicotinamide mononucleotide	0.000	0.000	0.000	0.000E+00	0.000E+00	0.000E+00
X_{431}			Callistephin Chloride	0.000	0.000	0.000	0.000E+00	0.000E+00	0.000E+00
X_{432}			Choline chloride	0.000	0.000	0.000	0.000E+00	0.000E+00	0.000E+00
X_{433}			cis or trans-Cinnamyl alcohol	0.000	0.000	0.000	0.000E+00	0.000E+00	0.000E+00
X_{434}			Citramalic acid	0.000	0.000	0.000	0.000E+00	0.000E+00	0.000E+00
X_{435}			Creatinine-anhydrous	0.000	0.000	0.000	0.000E+00	0.000E+00	0.000E+00
X_{436}			Cyanidin-3-O-(2-O-beta-xylopyranosyl	0.000	0.000	0.000	0.000E+00	0.000E+00	0.000E+00
X_{437}			Cyanidin-3-O-(2-O-beta-xylopyranosyl	0.000	0.000	0.000	0.000E+00	0.000E+00	0.000E+00
X_{438}			cyanidin-3-O-rhamnoside chloride	0.000	0.000	0.000	0.000E+00	0.000E+00	0.000E+00
X_{439}			cysteinylglycine	0.000	0.000	0.000	0.000E+00	0.000E+00	0.000E+00
X_{440}			Cytidine-5'-diphosphocholine sodium sa	0.000	0.000	0.000	0.000E+00	0.000E+00	0.000E+00
X_{441}			Cytidine-5'-monophosphate disodium sa	0.000	0.000	0.000	0.000E+00	0.000E+00	0.000E+00
X_{442}			D-(-)-Quinic acid	0.000	0.000	0.000	0.000E+00	0.000E+00	0.000E+00
X_{443}			D-(+)-Raffinose pentahydrate	0.000	0.000	0.000	0.000E+00	0.000E+00	0.000E+00
X_{444}			D-Ala-D-ala	0.000	0.000	0.000	0.000E+00	0.000E+00	0.000E+00
X_{445}			Daphnetin	0.000	0.000	0.000	0.000E+00	0.000E+00	0.000E+00
X_{446}			Delphinidin-3-O-(6-O-alpha-rhamnopyr	0.000	0.000	0.000	0.000E+00	0.000E+00	0.000E+00
X_{447}			D-Glucoheptose	0.000	0.000	0.000	0.000E+00	0.000E+00	0.000E+00
X_{448}			Diethanolamine	0.000	0.000	0.000	0.000E+00	0.000E+00	0.000E+00
X_{449}			DL-2-3-Diaminopropionic acid monohyd	0.000	0.000	0.000	0.000E+00	0.000E+00	0.000E+00
X_{450}			DL-beta-Hydroxybutyric acid	0.000	0.000	0.000	0.000E+00	0.000E+00	0.000E+00
X_{451}			DL-Glyceric Acid calcium Salt	0.000	0.000	0.000	0.000E+00	0.000E+00	0.000E+00
X_{452}			DL-homocystine	0.000	0.000	0.000	0.000E+00	0.000E+00	0.000E+00
X_{453}			DL-threo-beta-Methylaspartic acid	0.000	0.000	0.000	0.000E+00	0.000E+00	0.000E+00
X_{454}			D-Panose	0.000	0.000	0.000	0.000E+00	0.000E+00	0.000E+00
X_{455}			dUDP	0.000	0.000	0.000	0.000E+00	0.000E+00	0.000E+00
X_{456}			gamma-Amino-n-butyric acid	0.000	0.000	0.000	0.000E+00	0.000E+00	0.000E+00
X_{457}			Gluconasturtiin	0.000	0.000	0.000	0.000E+00	0.000E+00	0.000E+00
X_{458}			Glycocyamine	0.000	0.000	0.000	0.000E+00	0.000E+00	0.000E+00
X_{459}			Glycyl-L-proline	0.000	0.000	0.000	0.000E+00	0.000E+00	0.000E+00
X_{460}			Guanosine-5'-diphospho-beta-L-fucose	0.000	0.000	0.000	0.000E+00	0.000E+00	0.000E+00
X_{461}			Guanosine-5'-diphosphoglucose sodium	0.000	0.000	0.000	0.000E+00	0.000E+00	0.000E+00
X_{462}			Guanosine-5'-monophosphate Jdisodiur	0.000	0.000	0.000	0.000E+00	0.000E+00	0.000E+00
X_{463}			Hesperetin	0.000	0.000	0.000	0.000E+00	0.000E+00	0.000E+00
X_{464}			Hesperidin	0.000	0.000	0.000	0.000E+00	0.000E+00	0.000E+00
X_{465}			Isoguvacine hydrochloride	0.000	0.000	0.000	0.000E+00	0.000E+00	0.000E+00
X_{466}			isorhamnetin-3-O-glucoside	0.000	0.000	0.000	0.000E+00	0.000E+00	0.000E+00
X_{467}			Itaconic acid	0.000	0.000	0.000	0.000E+00	0.000E+00	0.000E+00
X_{468}			Kaempferol-3-Galactoside-6-Rhamnosi	0.000	0.000	0.000	0.000E+00	0.000E+00	0.000E+00
X_{469}			Kaempferol-7-O-alpha-L-rhamnoside	0.000	0.000	0.000	0.000E+00	0.000E+00	0.000E+00
X_{470}			Keracyanin Chloride	0.000	0.000	0.000	0.000E+00	0.000E+00	0.000E+00
X_{471}			L-Anserine nitrate salt	0.000	0.000	0.000	0.000E+00	0.000E+00	0.000E+00
X_{472}			L-beta-homoglutamine-HCl	0.000	0.000	0.000	0.000E+00	0.000E+00	0.000E+00
X_{473}			L-beta-homoleucine-HCl	0.000	0.000	0.000	0.000E+00	0.000E+00	0.000E+00
X_{474}			L-beta-homolysine-2HCl	0.000	0.000	0.000	0.000E+00	0.000E+00	0.000E+00
X_{475}			L-beta-homoproline-HCl	0.000	0.000	0.000	0.000E+00	0.000E+00	0.000E+00
X_{476}			L-beta-homothreonine	0.000	0.000	0.000	0.000E+00	0.000E+00	0.000E+00
X_{477}			L-Carnosine	0.000	0.000	0.000	0.000E+00	0.000E+00	0.000E+00
X_{478}			L-Cystine	0.000	0.000	0.000	0.000E+00	0.000E+00	0.000E+00
X_{479}			L-Glutathione (reduced form)	0.000	0.000	0.000	0.000E+00	0.000E+00	0.000E+00
X_{480}			Lidocain	0.000	0.000	0.000	0.000E+00	0.000E+00	0.000E+00
X_{481}			Lignoceric Acid	0.000	0.000	0.000	0.000E+00	0.000E+00	0.000E+00
X_{482}			L-Kynurenine	0.000	0.000	0.000	0.000E+00	0.000E+00	0.000E+00
X_{483}			L-Pyroglutamic acid	0.000	0.000	0.000	0.000E+00	0.000E+00	0.000E+00
X_{484}			L-Threonic acid hemicalcium salt	0.000	0.000	0.000	0.000E+00	0.000E+00	0.000E+00
X_{485}			luteolin-4'-O-glucoside	0.000	0.000	0.000	0.000E+00	0.000E+00	0.000E+00
X_{486}			Maleic acid	0.000	0.000	0.000	0.000E+00	0.000E+00	0.000E+00
X_{487}			Maltitol	0.000	0.000	0.000	0.000E+00	0.000E+00	0.000E+00

X_{488}			Methionine sulfoxide	0.000	0.000	0.000	0.000E+00	0.000E+00	0.000E+00
X_{489}			Methylmalonic acid	0.000	0.000	0.000	0.000E+00	0.000E+00	0.000E+00
X_{490}			m-Hydroxycinnamic acid	0.000	0.000	0.000	0.000E+00	0.000E+00	0.000E+00
X_{491}			mucic acid	0.000	0.000	0.000	0.000E+00	0.000E+00	0.000E+00
X_{492}			N-6-(delta-2-Isopentenyl)adenosinehen	0.000	0.000	0.000	0.000E+00	0.000E+00	0.000E+00
X_{493}			N-acetyl putrescine hydrochloride	0.000	0.000	0.000	0.000E+00	0.000E+00	0.000E+00
X_{494}			N-Acetyl-DL-aspartic acid	0.000	0.000	0.000	0.000E+00	0.000E+00	0.000E+00
X_{495}			N-acetyl-DL-serine	0.000	0.000	0.000	0.000E+00	0.000E+00	0.000E+00
X_{496}			N-acetyl-D-mannosamine	0.000	0.000	0.000	0.000E+00	0.000E+00	0.000E+00
X_{497}			N-Acetylglycine	0.000	0.000	0.000	0.000E+00	0.000E+00	0.000E+00
X_{498}			N-acetylneuraminic acid-Type IV-S-Sy	0.000	0.000	0.000	0.000E+00	0.000E+00	0.000E+00
X_{499}			naringenin-7-O-glucoside	0.000	0.000	0.000	0.000E+00	0.000E+00	0.000E+00
X_{500}			Neoeriocitrin	0.000	0.000	0.000	0.000E+00	0.000E+00	0.000E+00
X_{501}			Neohesperidin	0.000	0.000	0.000	0.000E+00	0.000E+00	0.000E+00
X_{502}			Nicotinic acid mono nucleotide	0.000	0.000	0.000	0.000E+00	0.000E+00	0.000E+00
X_{503}			Nystose Trihydrate	0.000	0.000	0.000	0.000E+00	0.000E+00	0.000E+00
X_{504}			O-Acetyl-L-homoserine hydrochloride	0.000	0.000	0.000	0.000E+00	0.000E+00	0.000E+00
X_{505}			o-Anisic Acid	0.000	0.000	0.000	0.000E+00	0.000E+00	0.000E+00
X_{506}			O-Phosphocholine	0.000	0.000	0.000	0.000E+00	0.000E+00	0.000E+00
X_{507}			O-Succinyl-L-homoserine	0.000	0.000	0.000	0.000E+00	0.000E+00	0.000E+00
X_{508}			Pimelic acid	0.000	0.000	0.000	0.000E+00	0.000E+00	0.000E+00
X_{509}			Puerarin	0.000	0.000	0.000	0.000E+00	0.000E+00	0.000E+00
X_{510}			pyridoxal hydrochrolide	0.000	0.000	0.000	0.000E+00	0.000E+00	0.000E+00
X_{511}			pyridoxal-5'-phosphate hydrate	0.000	0.000	0.000	0.000E+00	0.000E+00	0.000E+00
X_{512}			Pyridoxamine dihydrochloride	0.000	0.000	0.000	0.000E+00	0.000E+00	0.000E+00
X_{513}			Pyridoxine	0.000	0.000	0.000	0.000E+00	0.000E+00	0.000E+00
X_{514}			Quercetin-3-7-O-alpha-L-dirhamnopyr	0.000	0.000	0.000	0.000E+00	0.000E+00	0.000E+00
X_{515}			Quercetin-3-O-alpha-L-rhamnopyranos	0.000	0.000	0.000	0.000E+00	0.000E+00	0.000E+00
X_{516}			Robinin	0.000	0.000	0.000	0.000E+00	0.000E+00	0.000E+00
X_{517}			Safranine	0.000	0.000	0.000	0.000E+00	0.000E+00	0.000E+00
X_{518}			Salicylic Acid	0.000	0.000	0.000	0.000E+00	0.000E+00	0.000E+00
X_{519}			Sinapic acid	0.000	0.000	0.000	0.000E+00	0.000E+00	0.000E+00
X_{520}			Sinapine	0.000	0.000	0.000	0.000E+00	0.000E+00	0.000E+00
X_{521}			Sinapoyl malate	0.000	0.000	0.000	0.000E+00	0.000E+00	0.000E+00
X_{522}			S-Methyl-L-cysteine	0.000	0.000	0.000	0.000E+00	0.000E+00	0.000E+00
X_{523}			sn-Glycero-3-phosphocholine 1:1 cadm	0.000	0.000	0.000	0.000E+00	0.000E+00	0.000E+00
X_{524}			Suberic acid	0.000	0.000	0.000	0.000E+00	0.000E+00	0.000E+00
X_{525}			Sucrose	0.000	0.000	0.000	0.000E+00	0.000E+00	0.000E+00
X_{526}			Thiamine hydrochloride	0.000	0.000	0.000	0.000E+00	0.000E+00	0.000E+00
X_{527}			Thioglycolic acid solution	0.000	0.000	0.000	0.000E+00	0.000E+00	0.000E+00
X_{528}			trans-Zeatin-riboside	0.000	0.000	0.000	0.000E+00	0.000E+00	0.000E+00
X_{529}			Trimethylamine N-oxide dihydrate	0.000	0.000	0.000	0.000E+00	0.000E+00	0.000E+00
X_{530}			UDP-beta-L-rhamnose	0.000	0.000	0.000	0.000E+00	0.000E+00	0.000E+00
X_{531}			UDP-xylose	0.000	0.000	0.000	0.000E+00	0.000E+00	0.000E+00
X_{532}			Vanillin	0.000	0.000	0.000	0.000E+00	0.000E+00	0.000E+00
X_{533}			Zeatin-9-glucoside	0.000	0.000	0.000	0.000E+00	0.000E+00	0.000E+00
