# Supplementary material for: A U-system approach for predicting metabolic behaviors and responses based on an alleged metabolic reaction network
Source: BMC Syst Biol. 2014 Dec 12;8(Suppl 5):S4. doi: 10.1186/1752-0509-8-S5-S4 (PMC4305983; doi:10.1186/1752-0509-8-S5-S4)
Supplement: Additional file 3 — Usystem_SupplementaryFigures.pdf. The U-system simulations comparing with experimental data from metabolome and amino acids analysis. [file 1752-0509-8-S5-S4-S3.pdf]

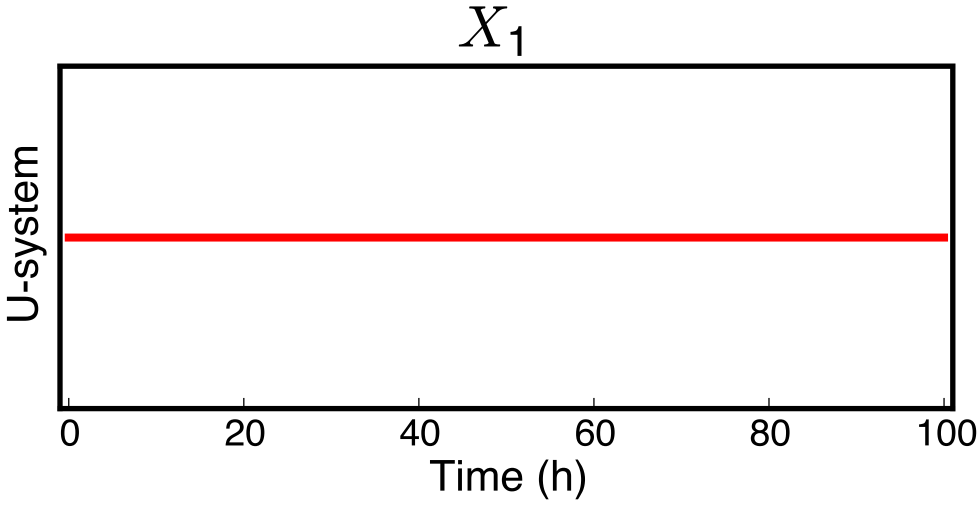

X_1
U-system
0
20
40
60
80
100
Time (h)

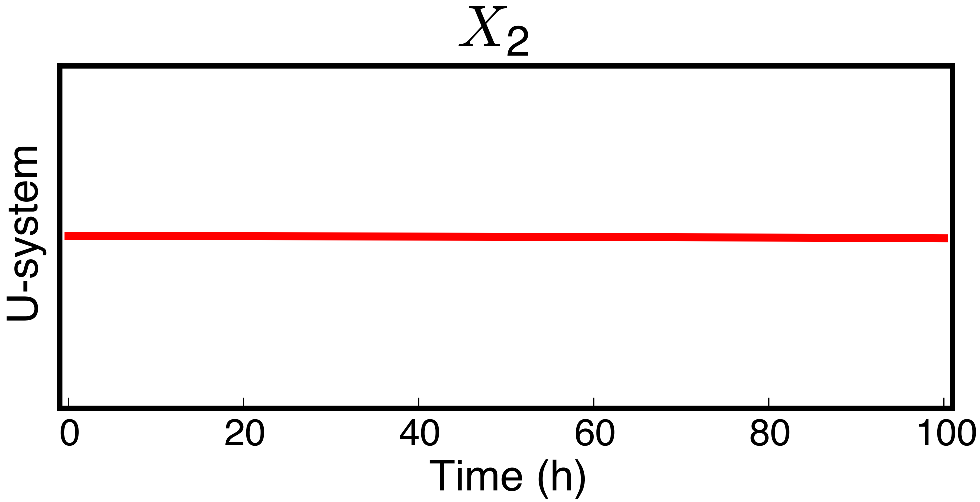
X_2
U-system
0
20
40
60
80
100
Time (h)

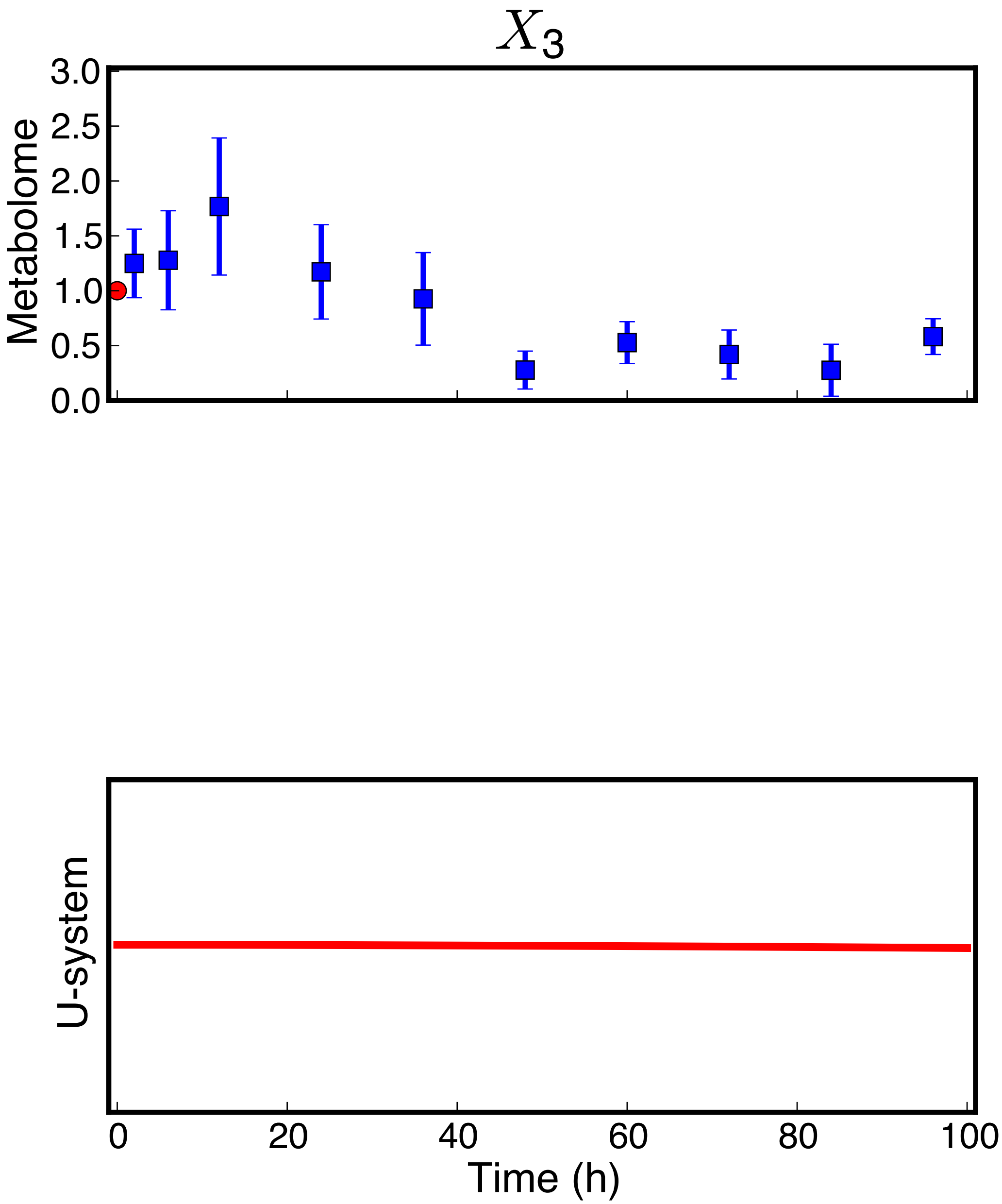
X_3
3.0
2.5
2.0
1.5
1.0
0.5
0.0
Metabolome
U-system
0
20
40
60
80
100
Time (h)

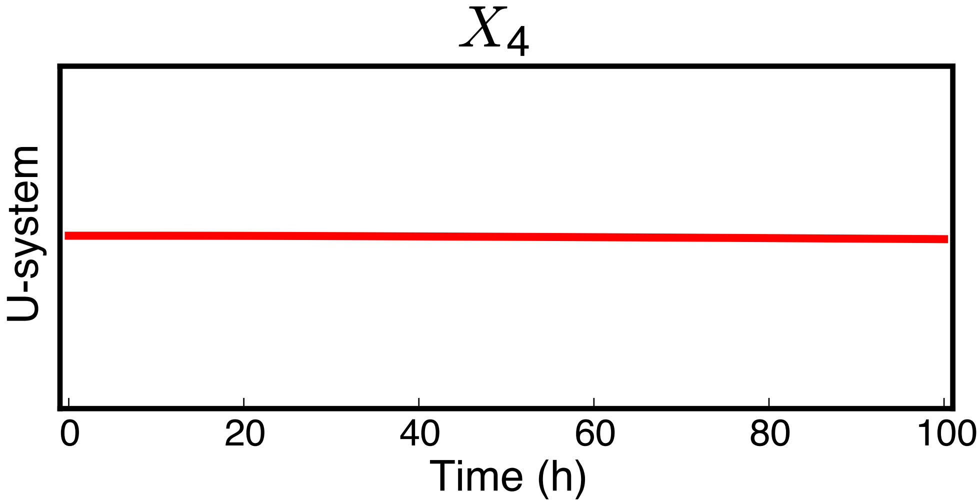

X_4
U-system
0
20
40
60
80
100
Time (h)

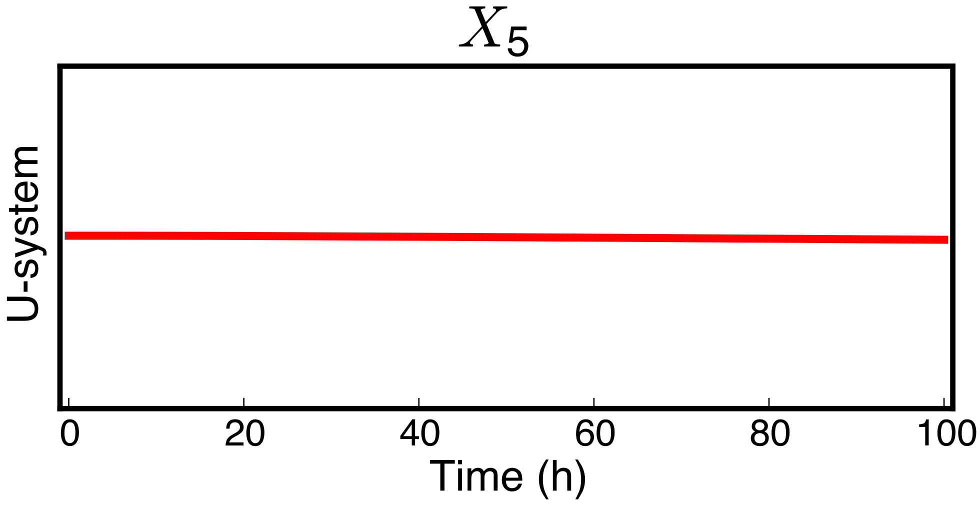

X_5
U-system
0
20
40
60
80
100
Time (h)

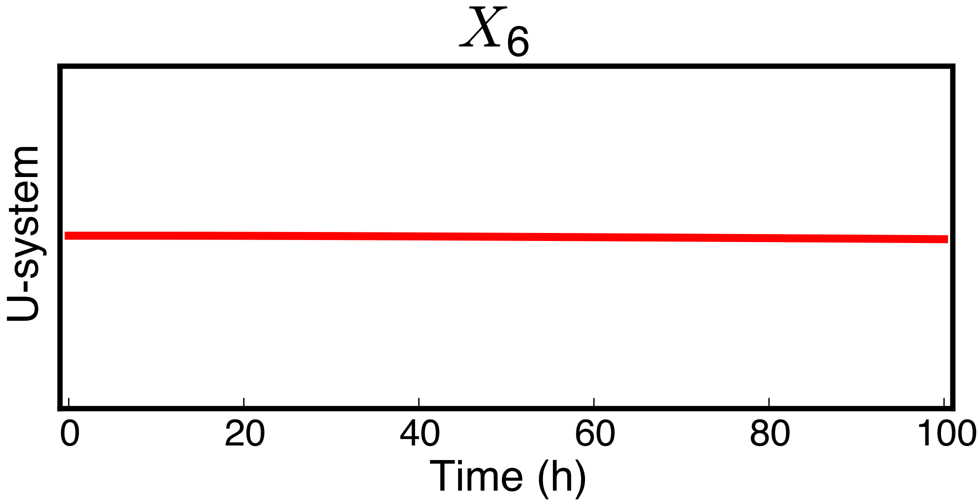

X_6
U-system
0
20
40
60
80
100
Time (h)

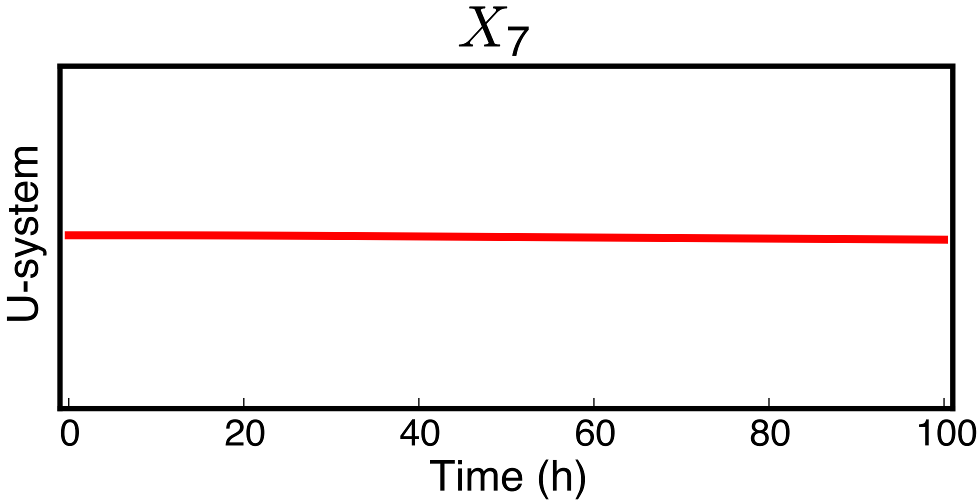
X_7
U-system
0
20
40
60
80
100
Time (h)

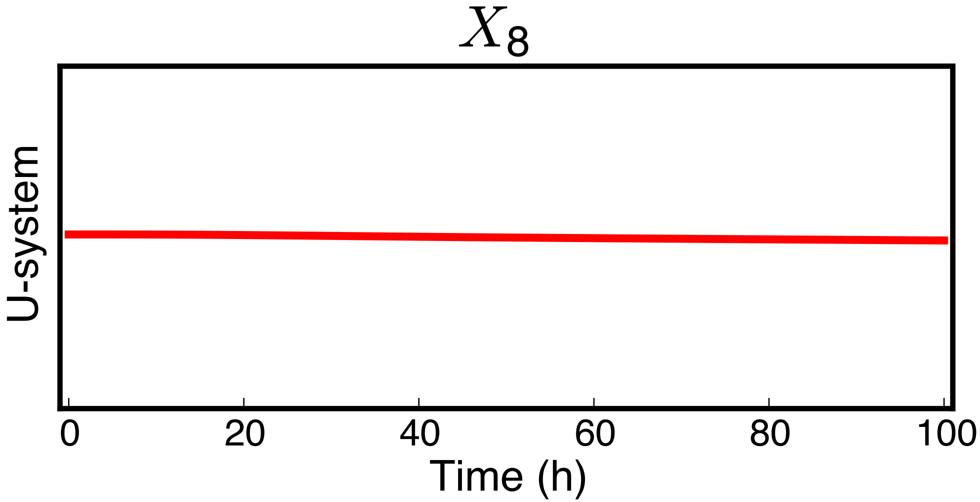

X_8
U-system
0
20
40
60
80
100
Time (h)

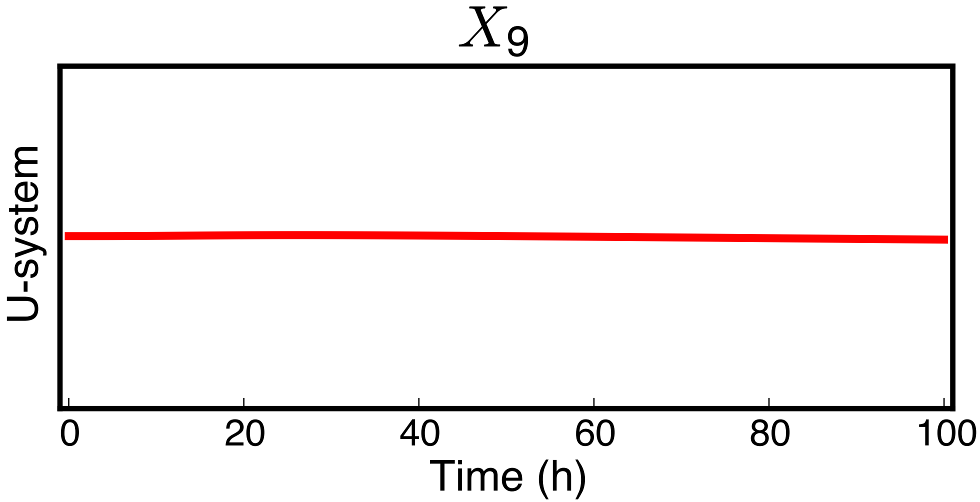

X_9
U-system
0
20
40
60
80
100
Time (h)

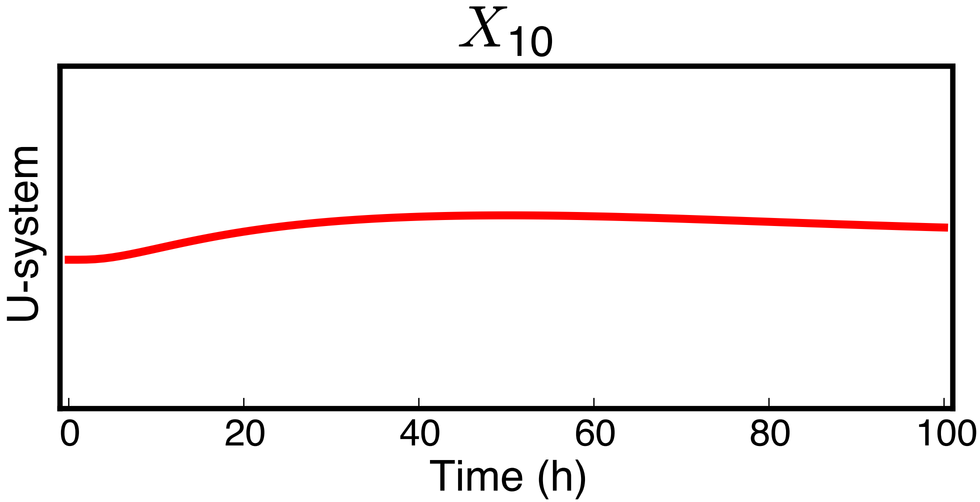

X_{10}
U-system
0
20
40
60
80
100
Time (h)

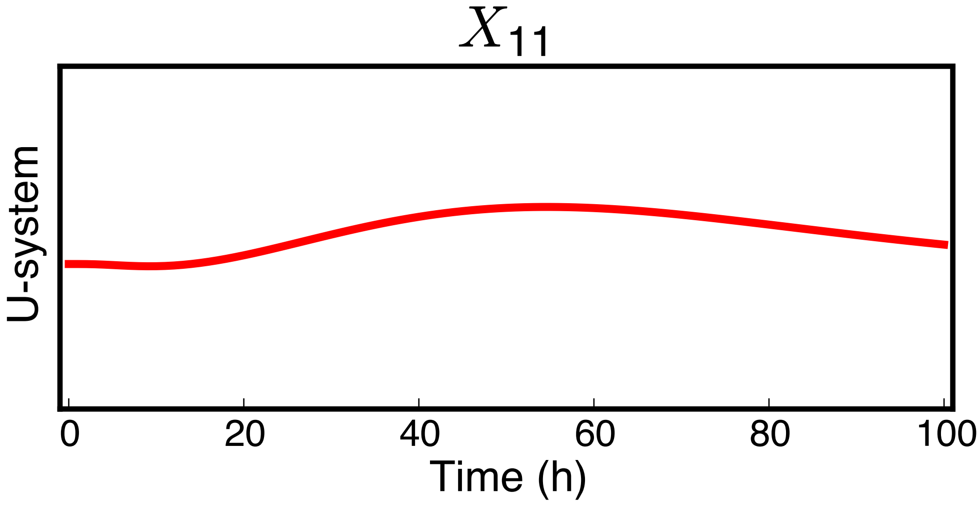
X_{11}
U-system
0
20
40
60
80
100
Time (h)

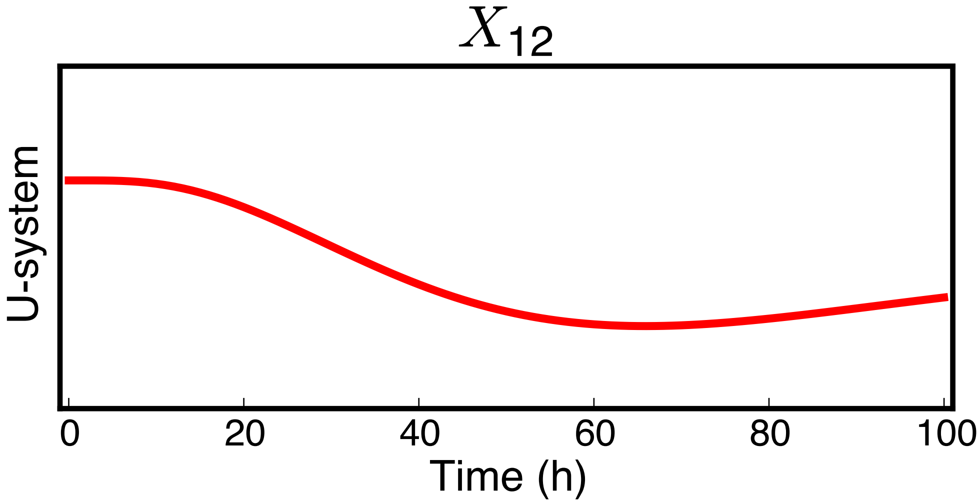

X_{12}
U-system
0
20
40
60
80
100
Time (h)

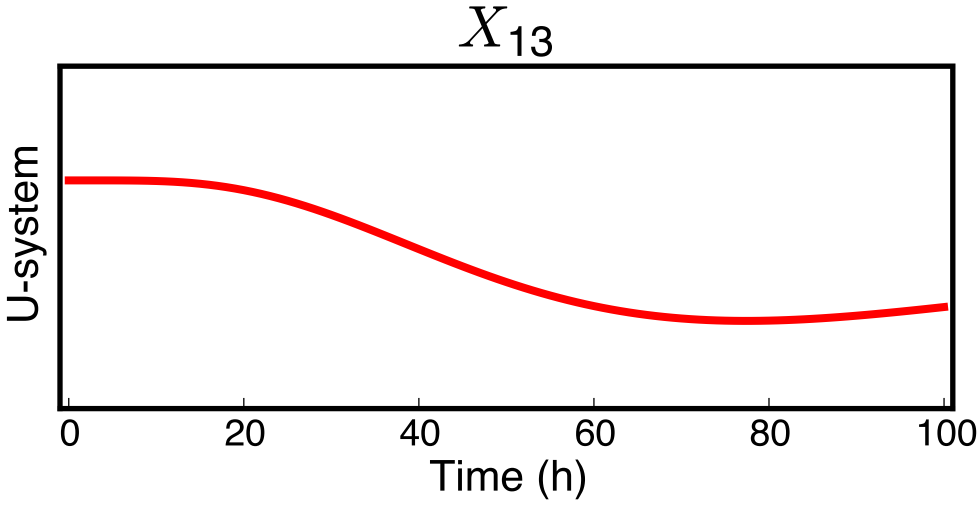

X_{13}
U-system
0
20
40
60
80
100
Time (h)

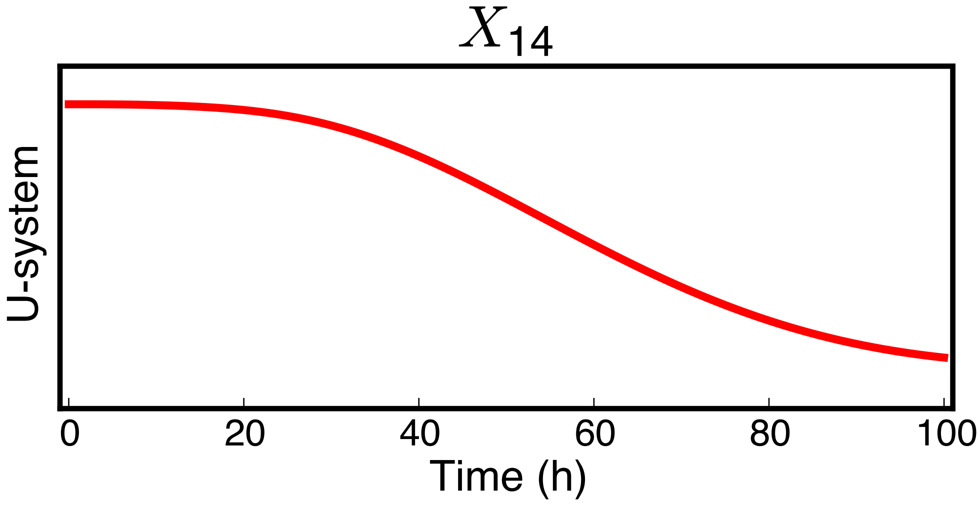

X_{14}
U-system
0
20
40
60
80
100
Time (h)

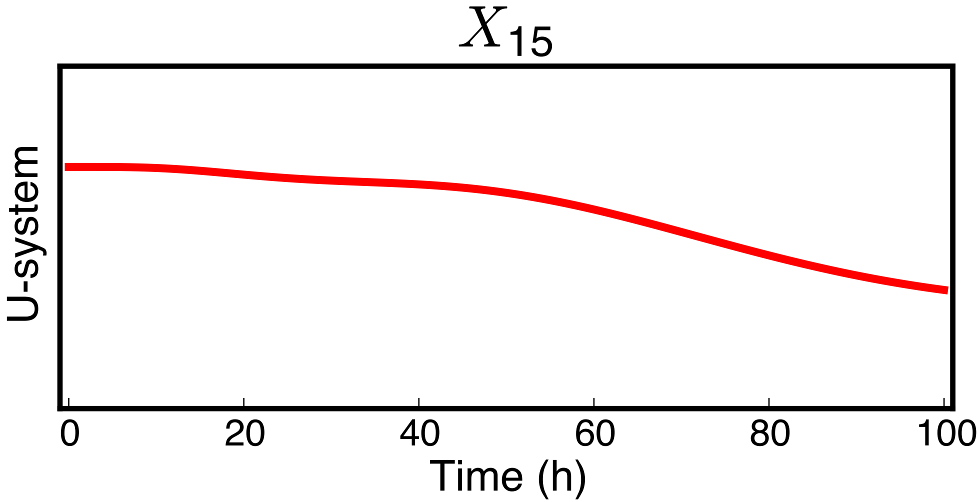
X_{15}
U-system
0
20
40
60
80
100
Time (h)

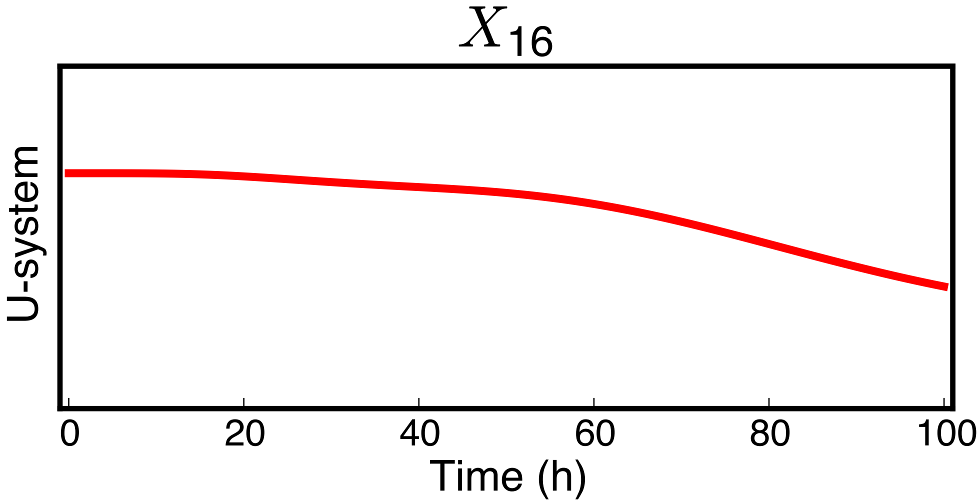

X_{16}
U-system
0
20
40
60
80
100
Time (h)

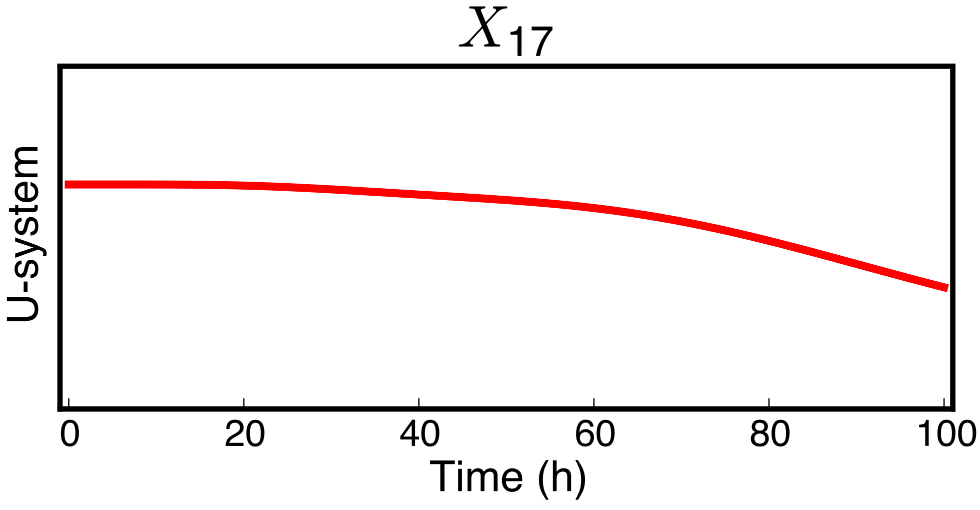
X_{17}
U-system
0
20
40
60
80
100
Time (h)

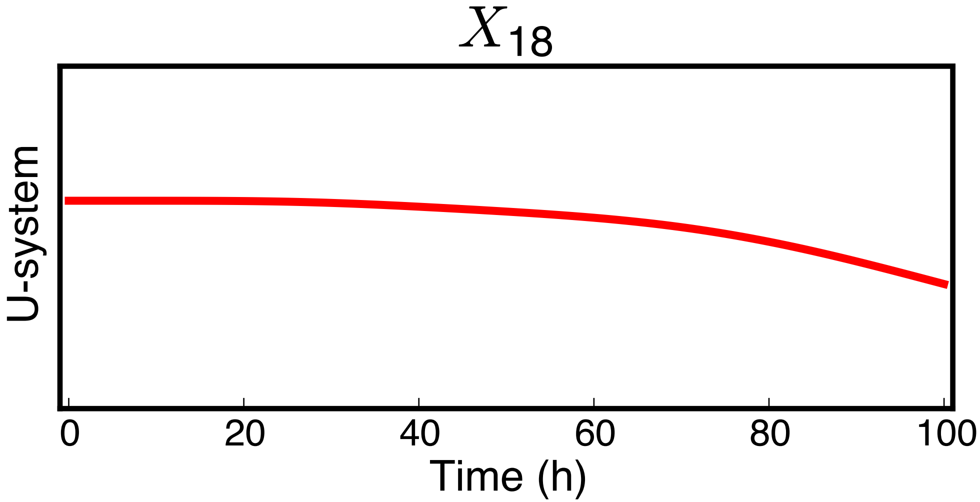

X_{18}
U-system
0
20
40
60
80
100
Time (h)

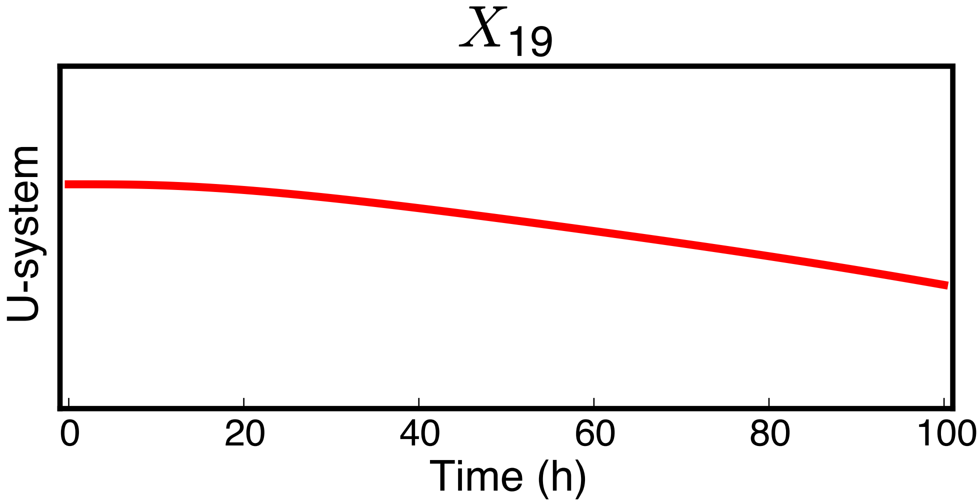

X_{19}
U-system
0
20
40
60
80
100
Time (h)

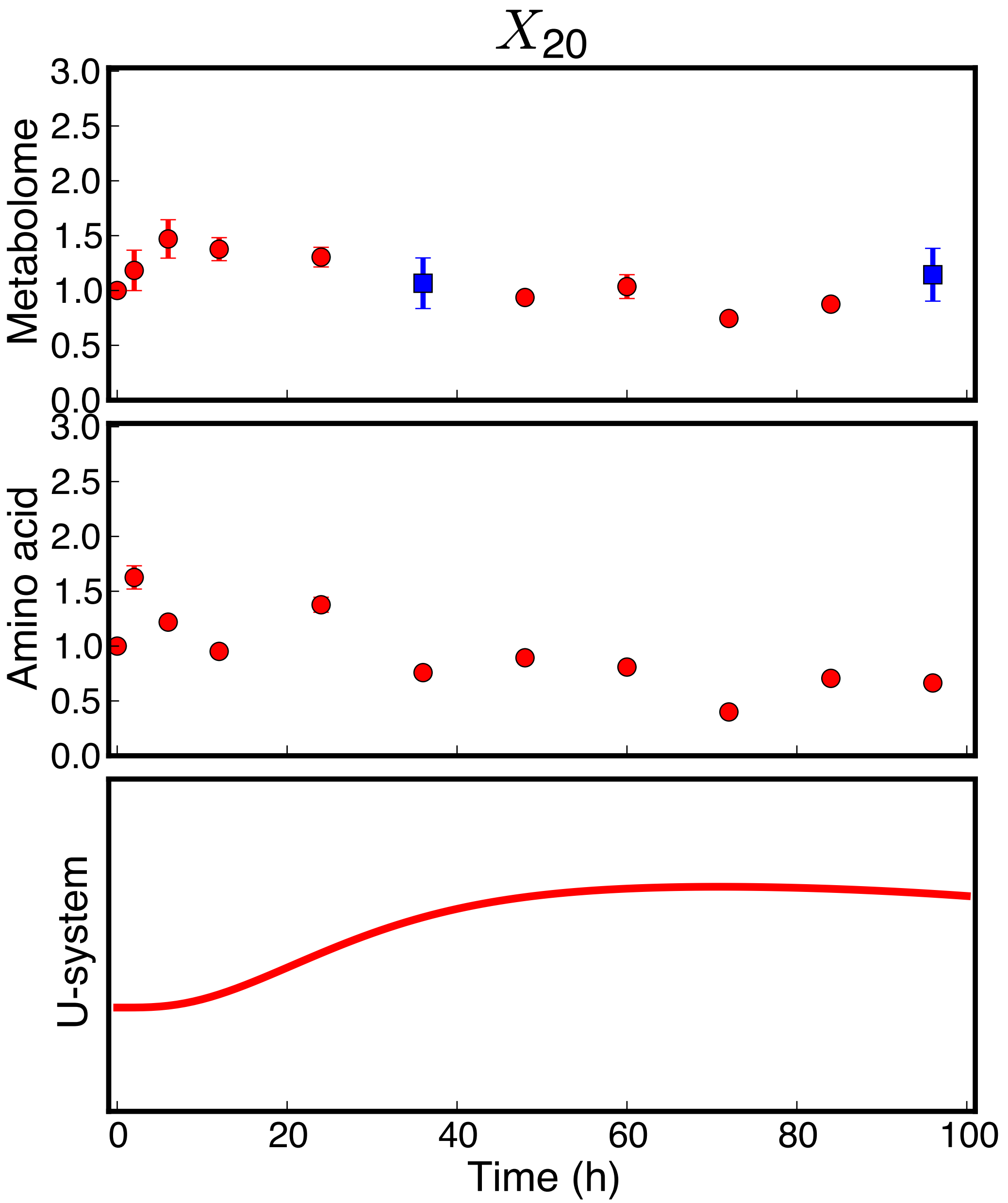
X_{20}
Metabolome
Amino acid
U-system
3.0
2.5
2.0
1.5
1.0
0.5
0.0
0
20
40
60
80
100
Time (h)

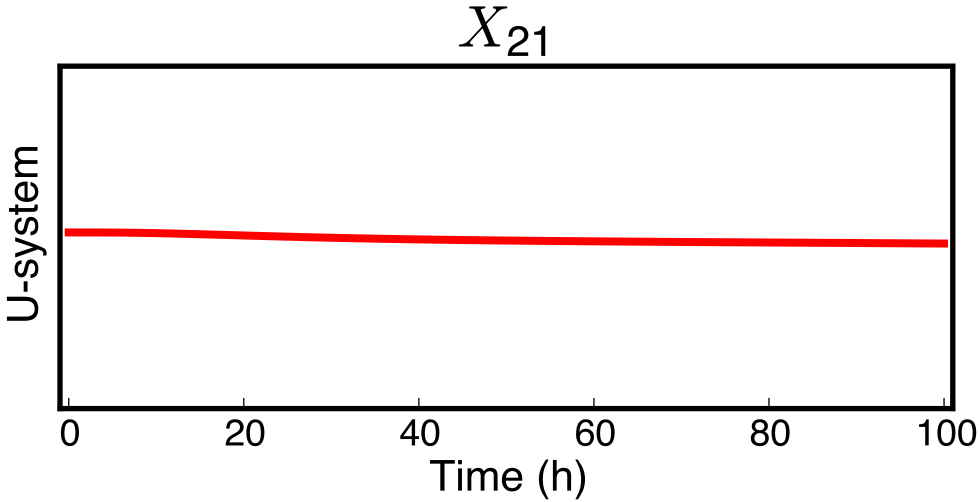
X_{21}
U-system
0
20
40
60
80
100
Time (h)

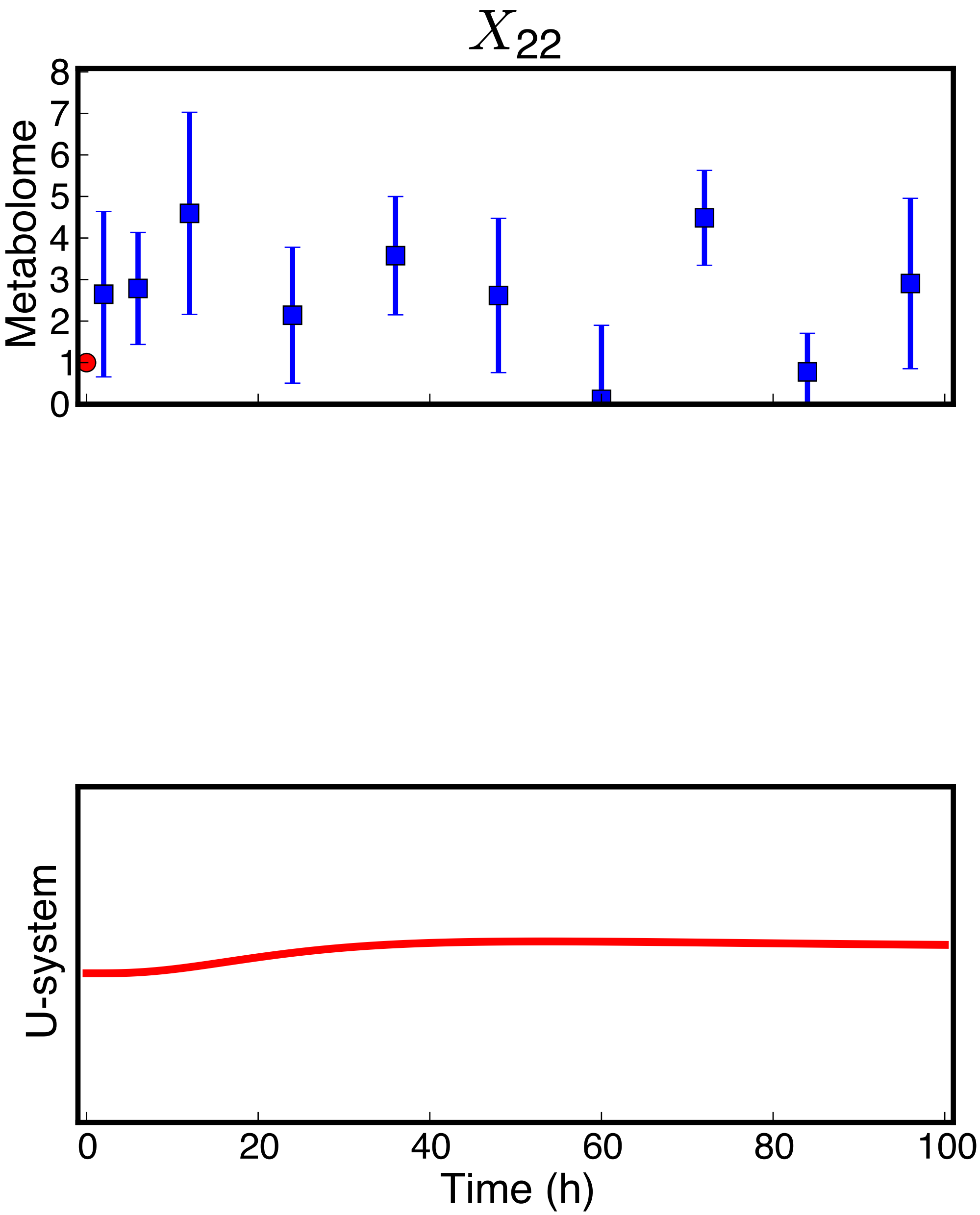

X_{22}
Metabolome
8
7
6
5
4
3
2
1
0
U-system
0
20
40
60
80
100
Time (h)

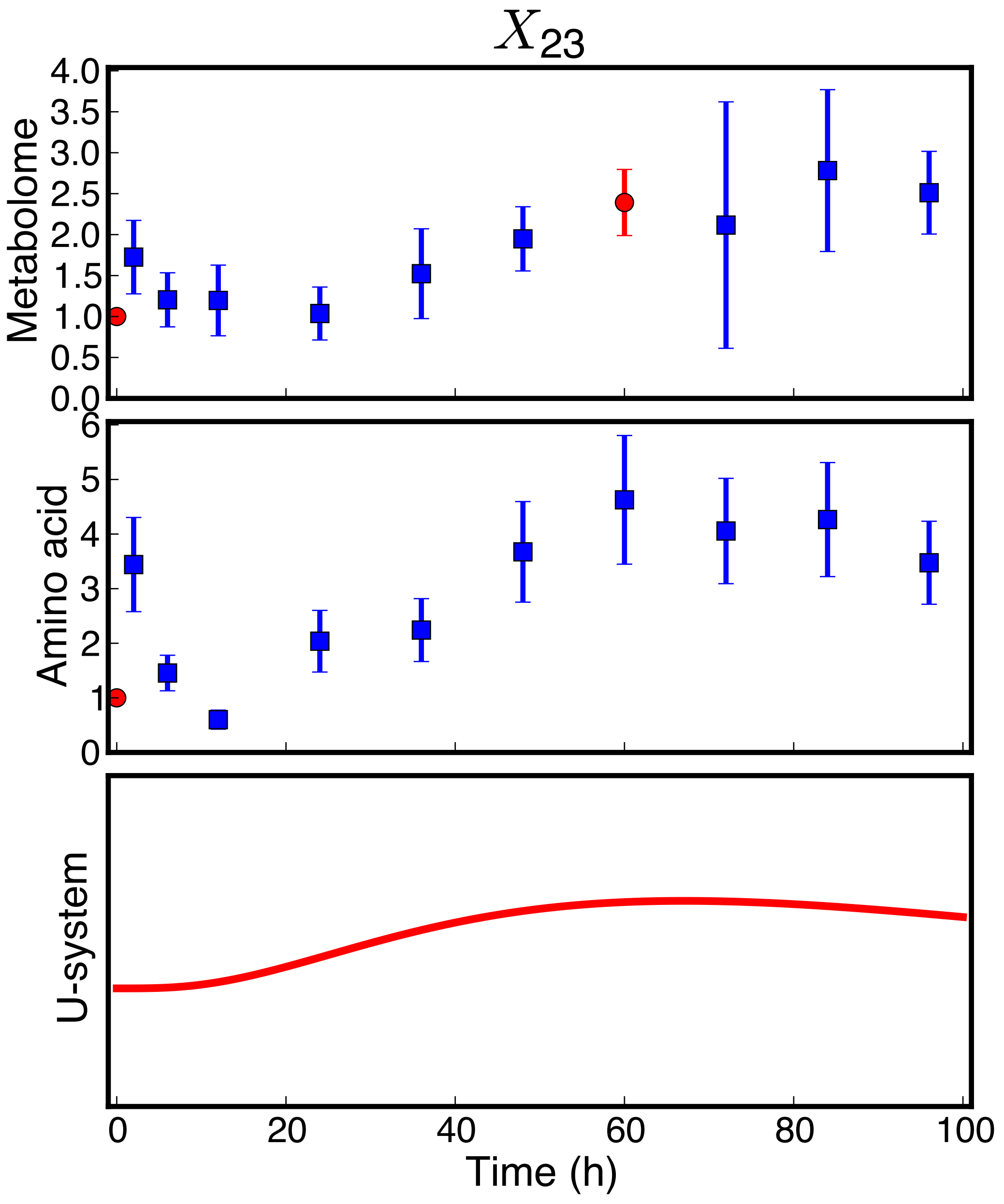

X_{23}
Metabolome
4.0
3.5
3.0
2.5
2.0
1.5
1.0
0.5
0.0
Amino acid
6
5
4
3
2
1
0
U-system
0
20
40
60
80
100
Time (h)

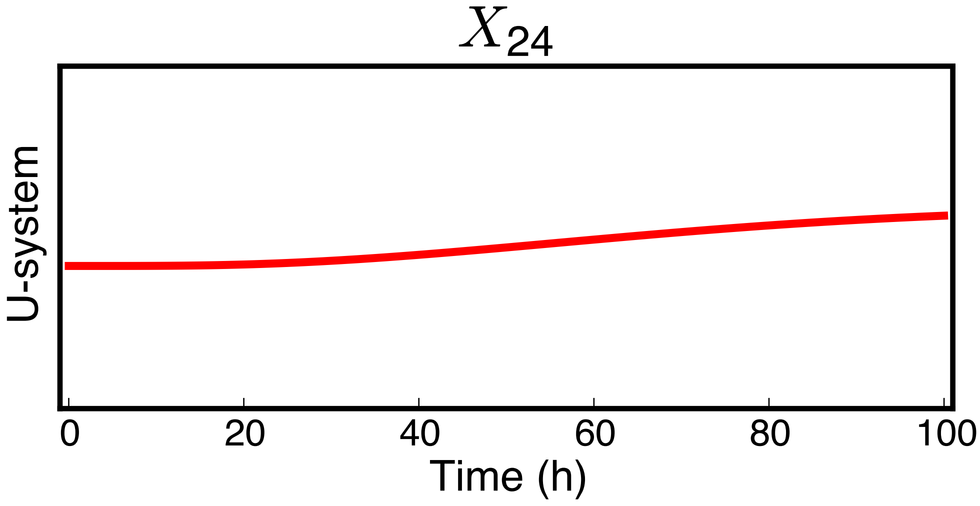

X_{24}
U-system
0
20
40
60
80
100
Time (h)

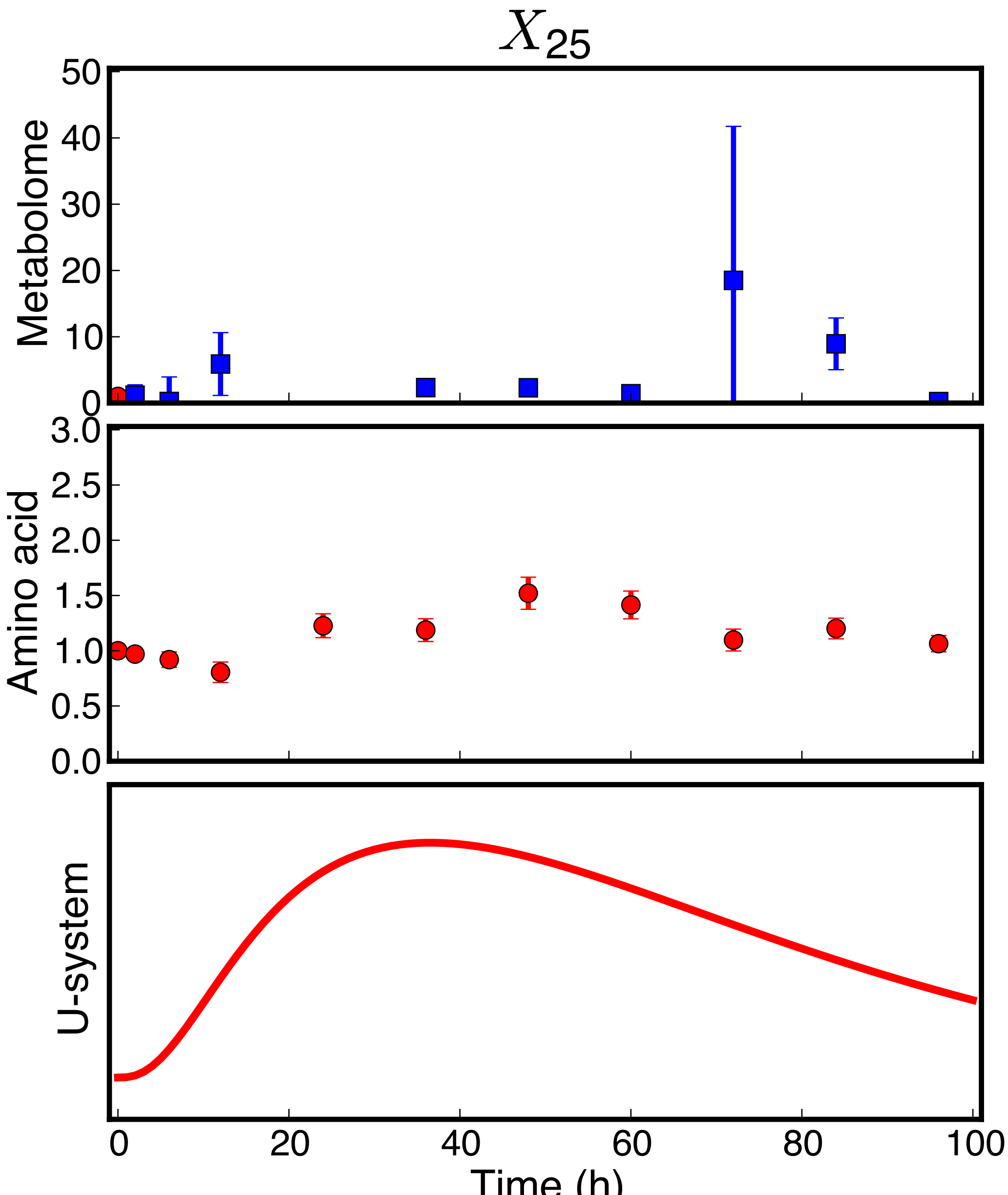

X_{25}
Metabolome
0
10
20
30
40
50
Amino acid
0.0
0.5
1.0
1.5
2.0
2.5
3.0
U-system
0
20
40
60
80
100
Time (h)

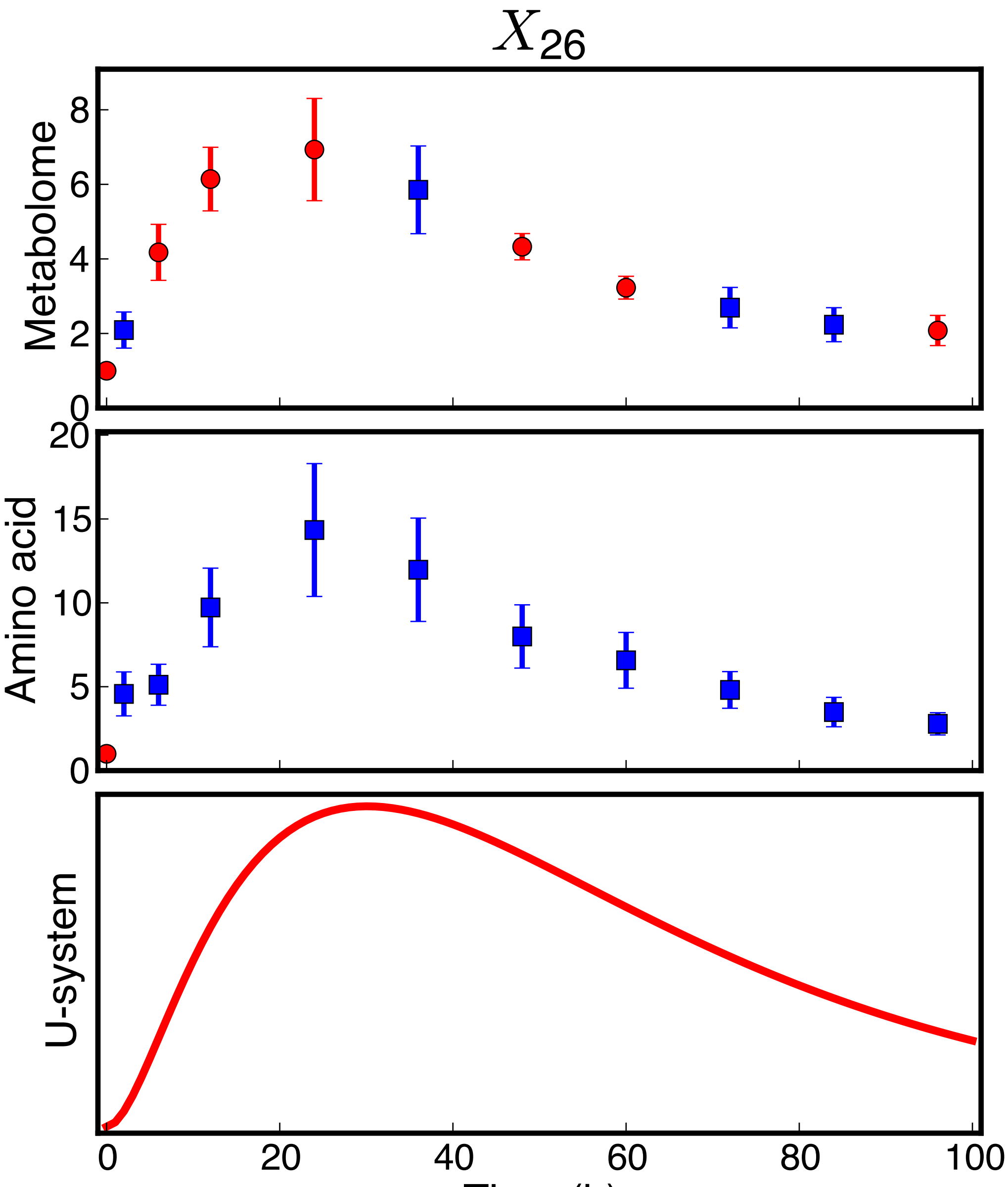

X_{26}
Metabolome
0
2
4
6
8
Amino acid
0
5
10
15
20
U-system
0
20
40
60
80
100
Time (h)

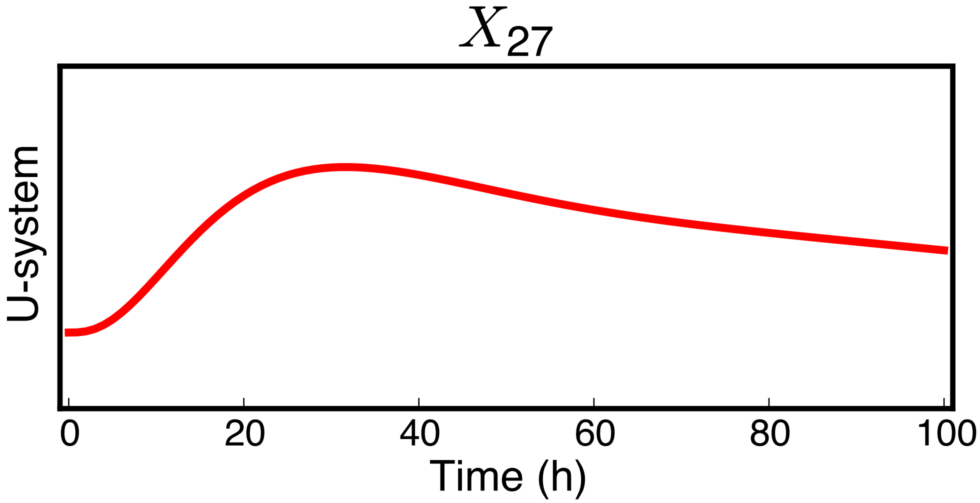

X_{27}
U-system
0
20
40
60
80
100
Time (h)

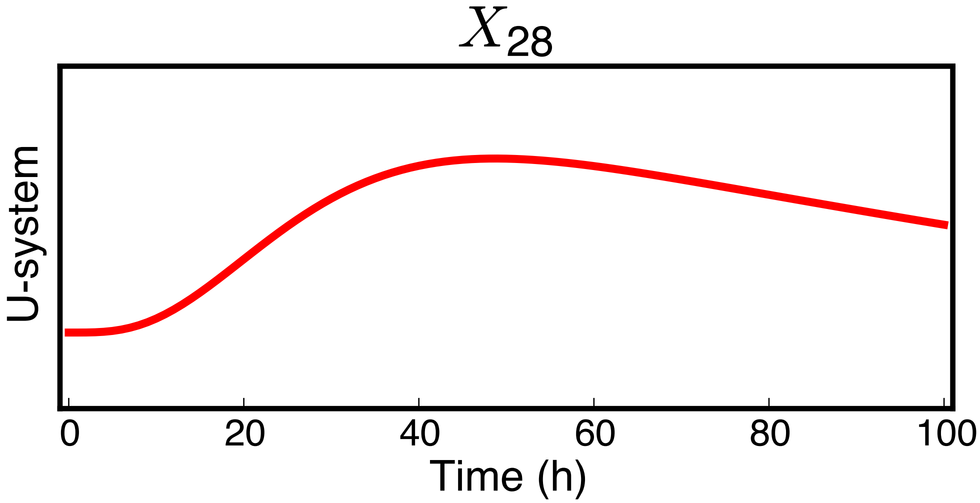
X_{28}
U-system
0
20
40
60
80
100
Time (h)

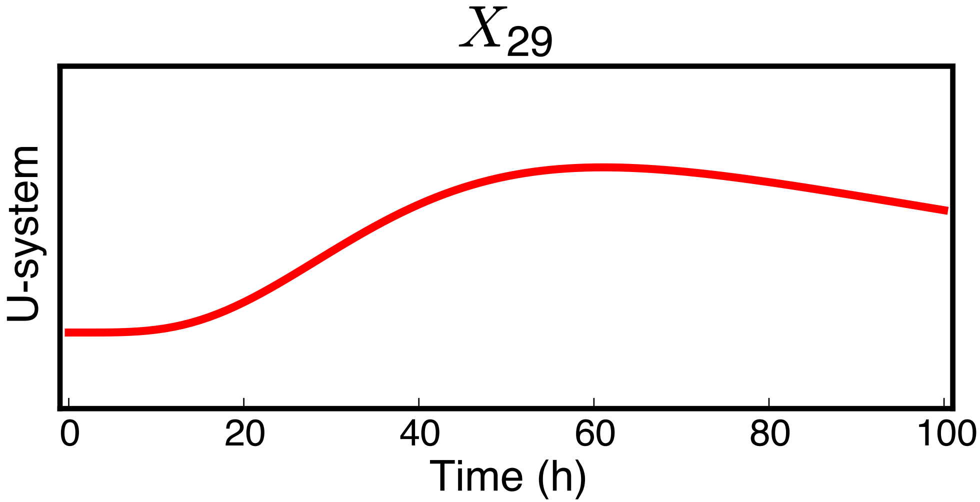

X_{29}
U-system
0
20
40
60
80
100
Time (h)

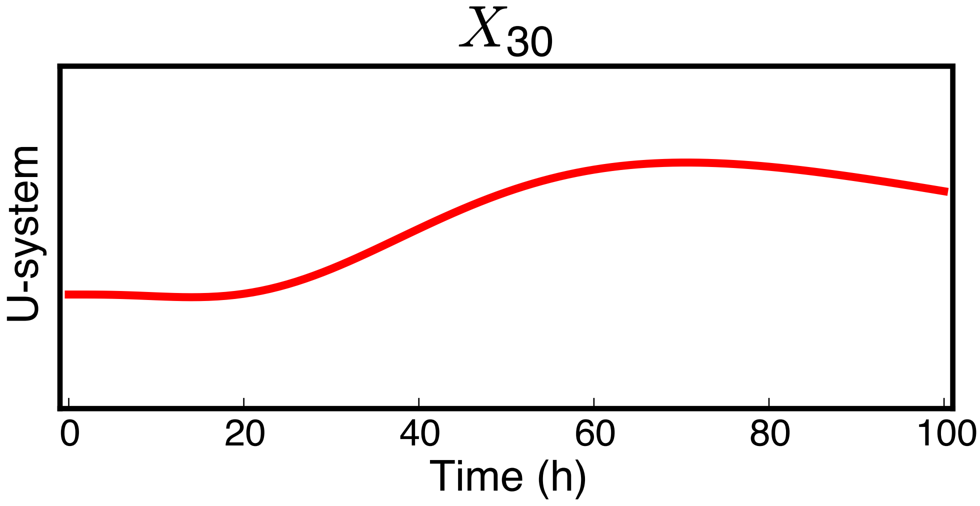

X_{30}
U-system
0
20
40
60
80
100
Time (h)

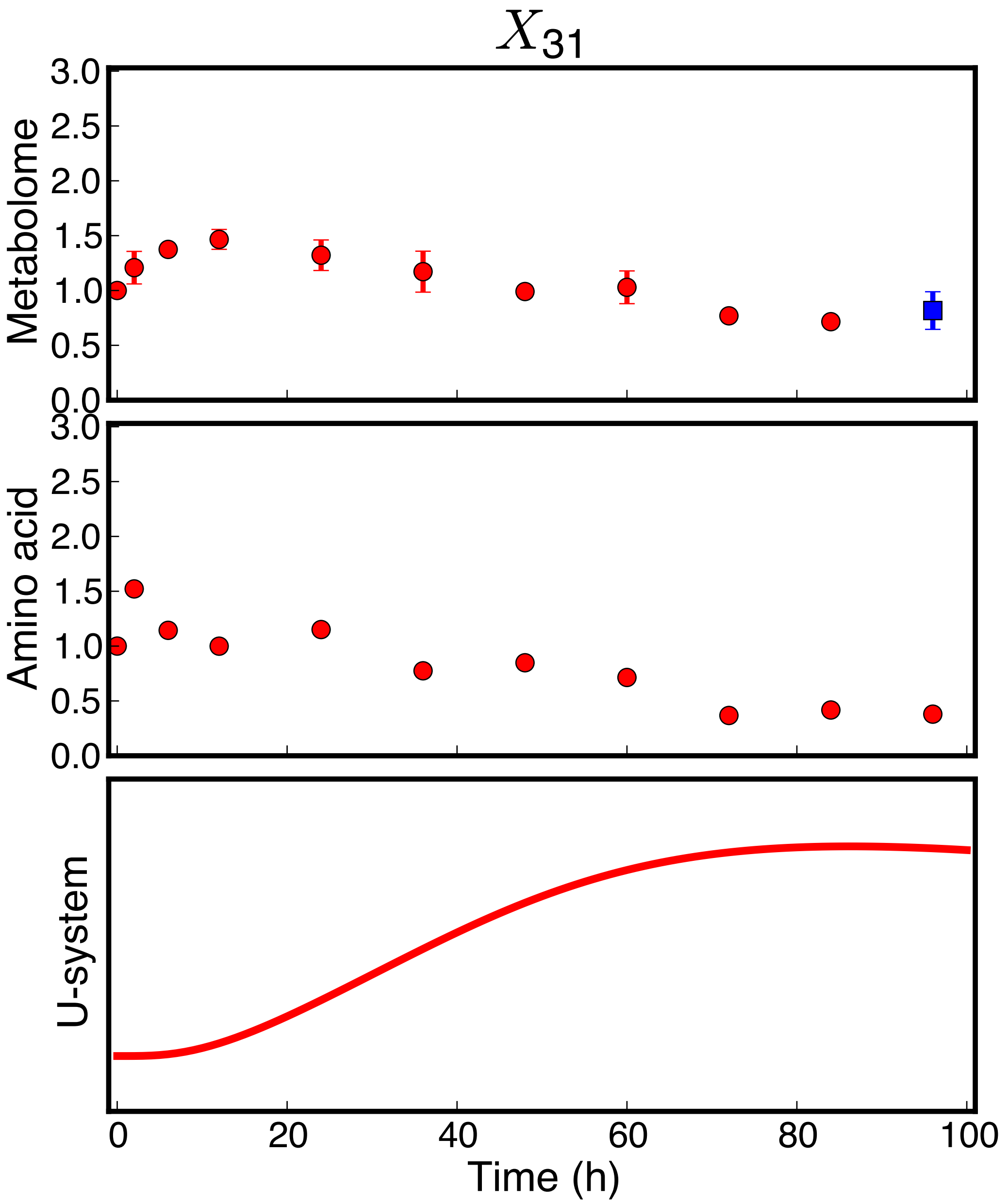

X_{31}
Metabolome
Amino acid
U-system
Time (h)
0.0
0.5
1.0
1.5
2.0
2.5
3.0
0
20
40
60
80
100

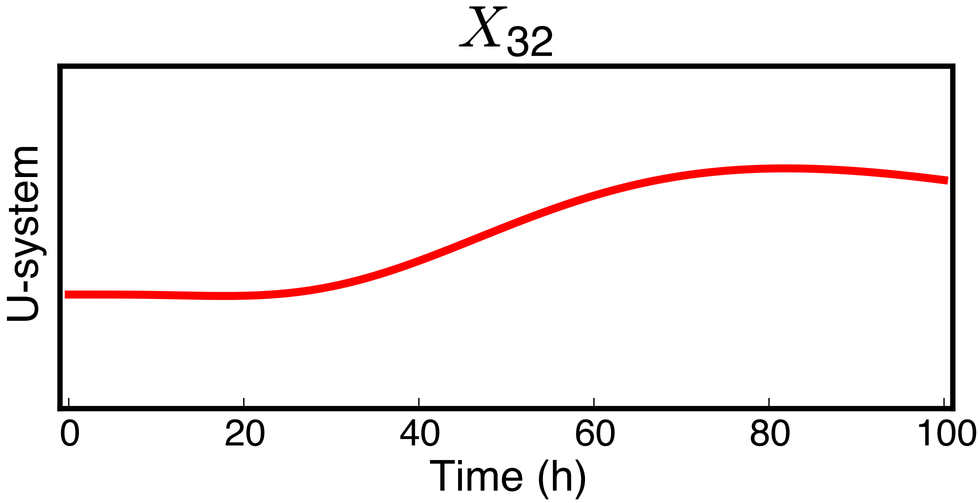
X_{32}
U-system
0
20
40
60
80
100
Time (h)

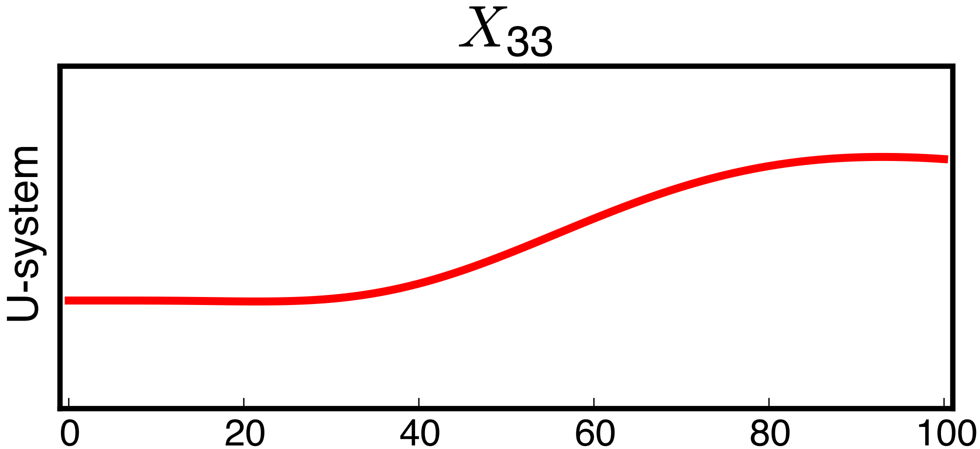

X_{33}
U-system
0
20
40
60
80
100
Time (h)

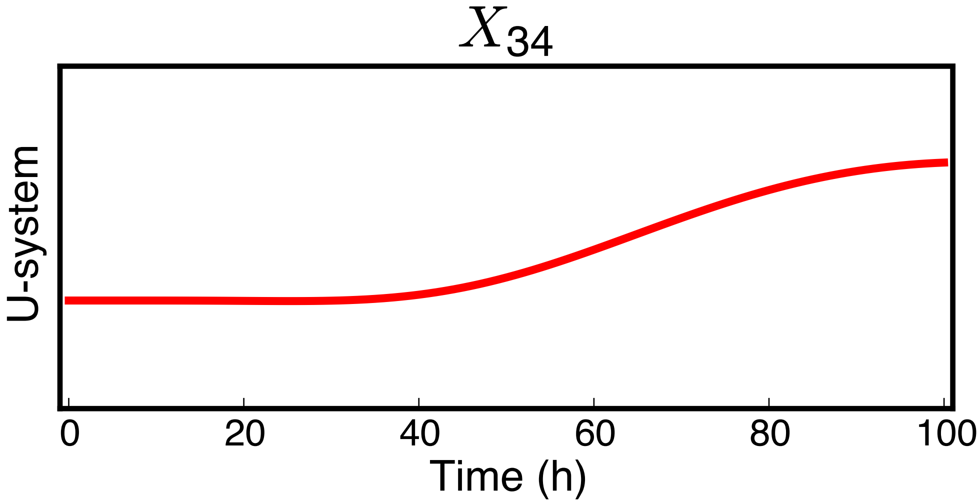

X_{34}
U-system
0
20
40
60
80
100
Time (h)

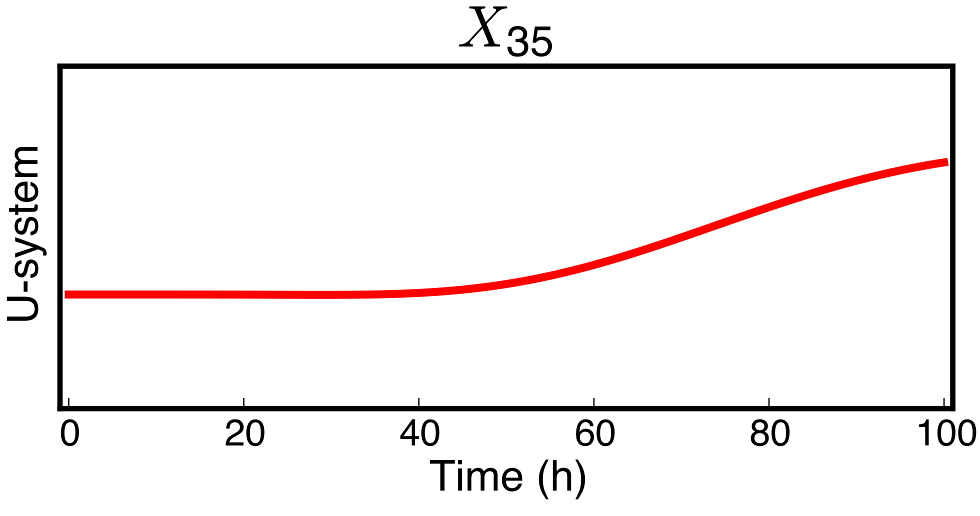

X_{35}
U-system
0
20
40
60
80
100
Time (h)

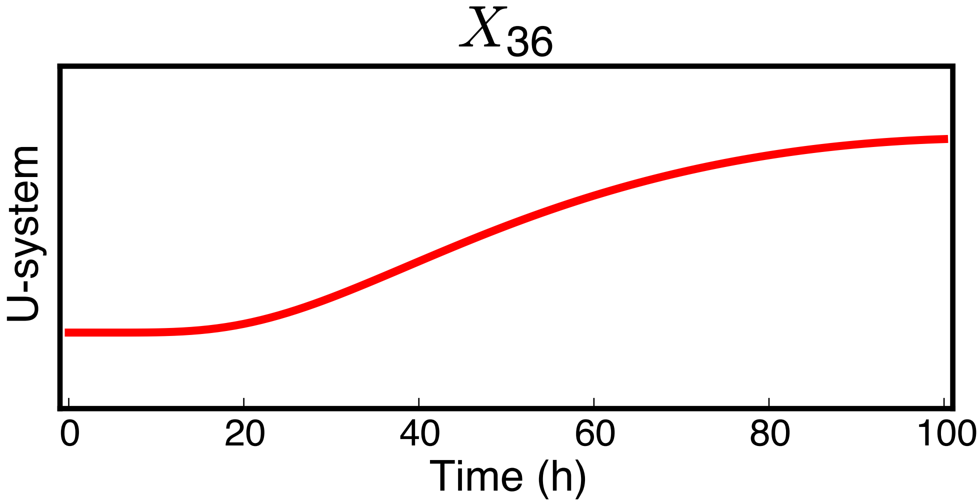

X_{36}
U-system
0
20
40
60
80
100
Time (h)

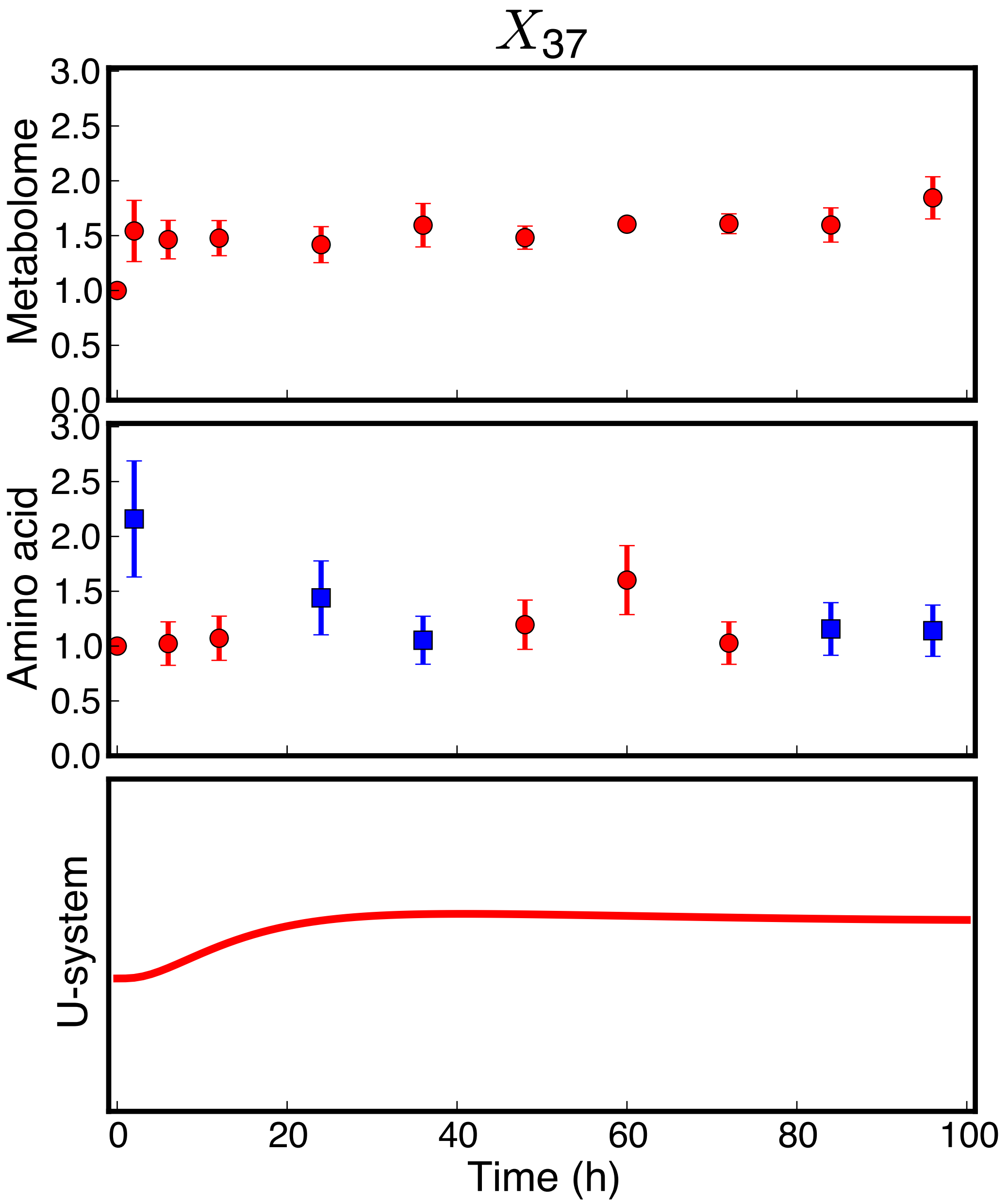
X_{37}
Metabolome
Amino acid
U-system
3.0
2.5
2.0
1.5
1.0
0.5
0.0
0
20
40
60
80
100
Time (h)

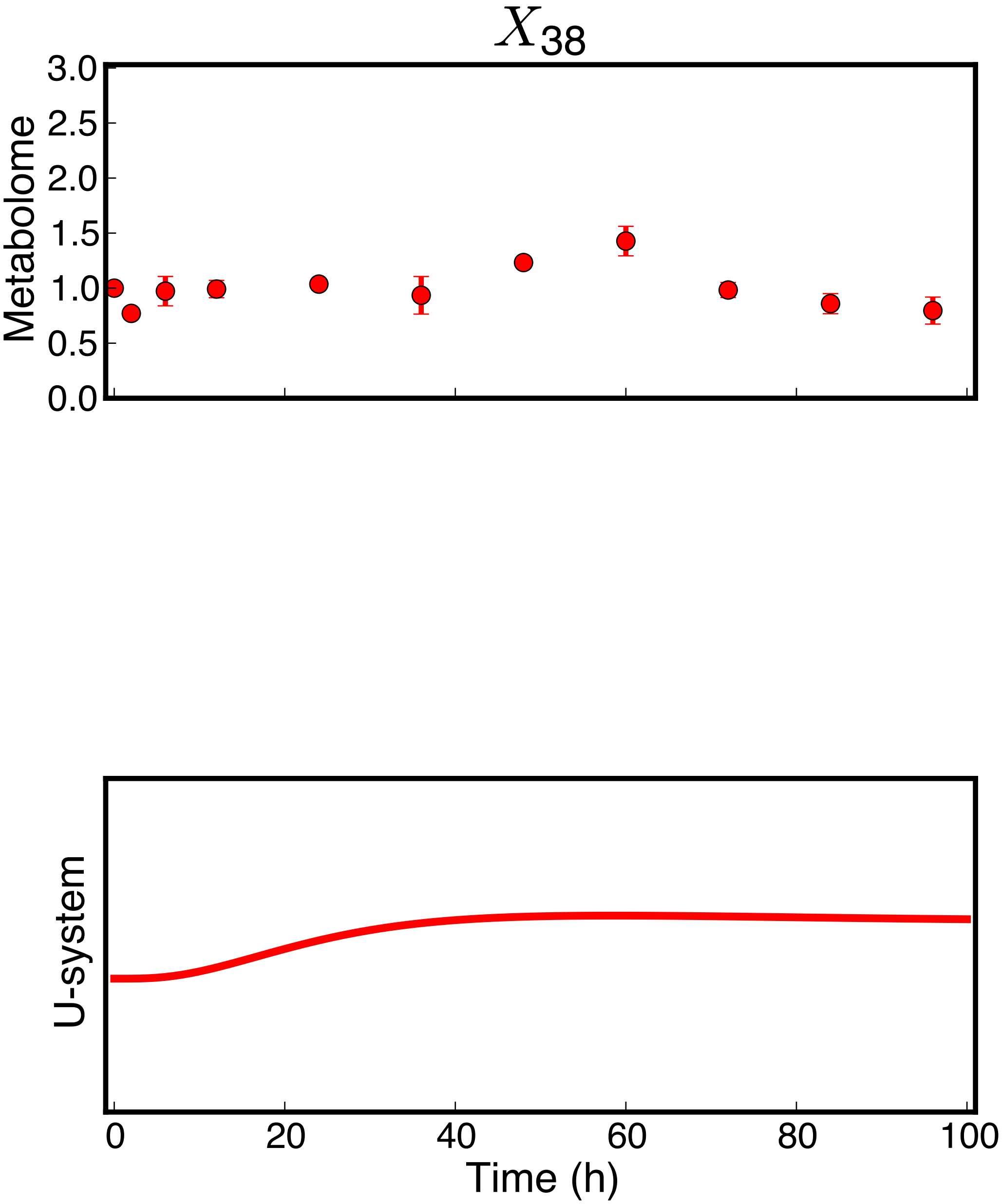
X_{38}
Metabolome
3.0
2.5
2.0
1.5
1.0
0.5
0.0
U-system
0
20
40
60
80
100
Time (h)

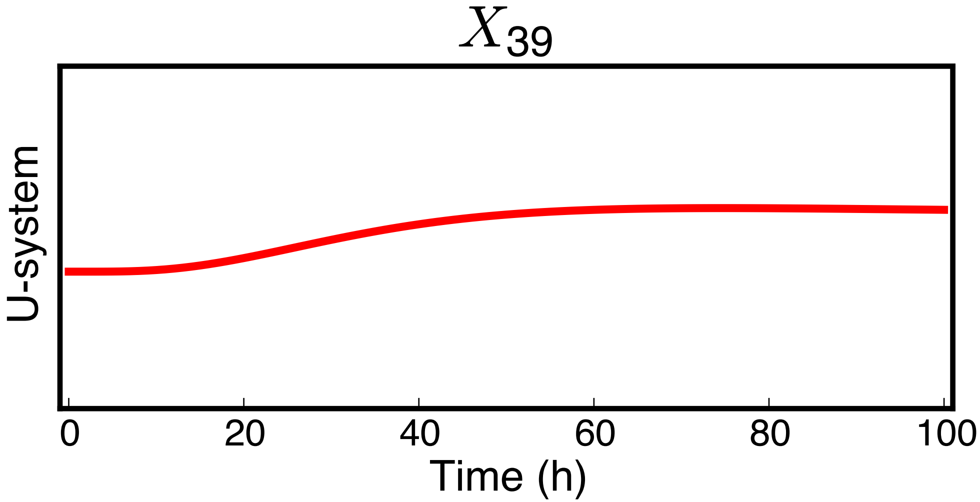

X_{39}
U-system
0
20
40
60
80
100
Time (h)

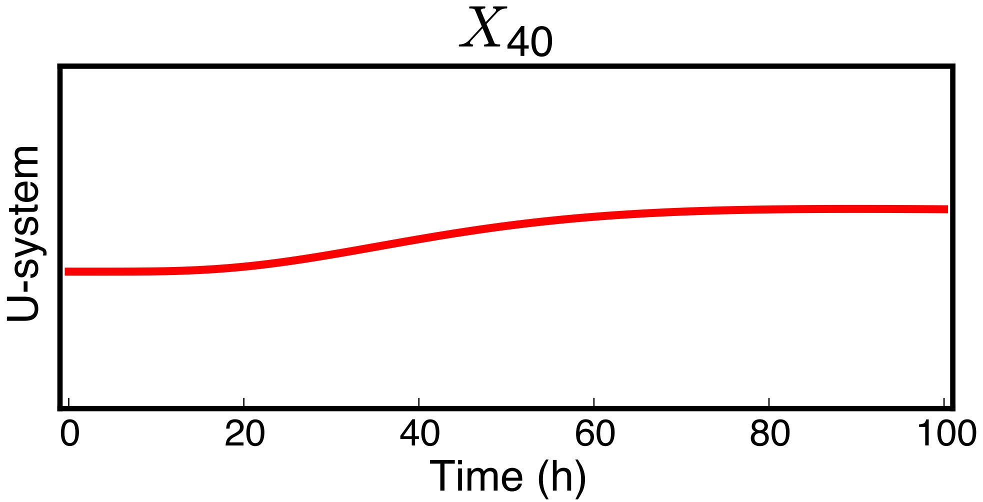
X_{40}
U-system
0
20
40
60
80
100
Time (h)

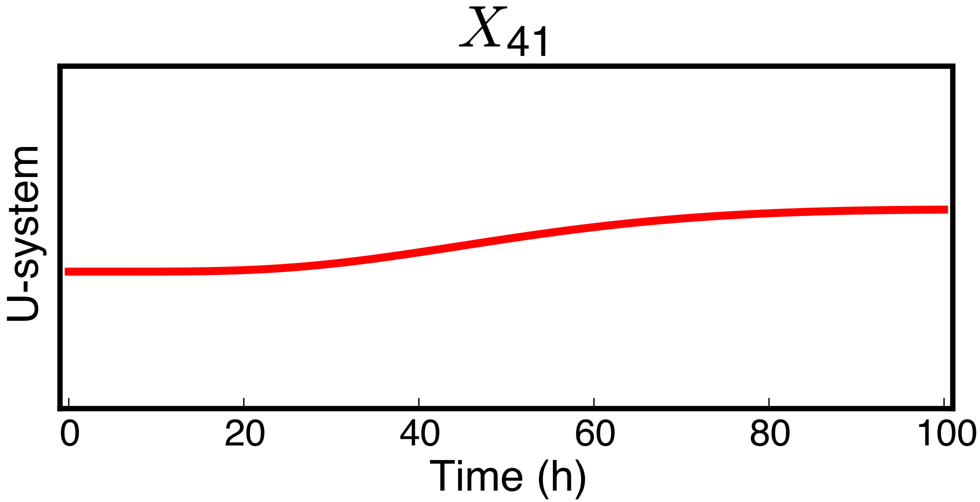

X_{41}
U-system
0
20
40
60
80
100
Time (h)

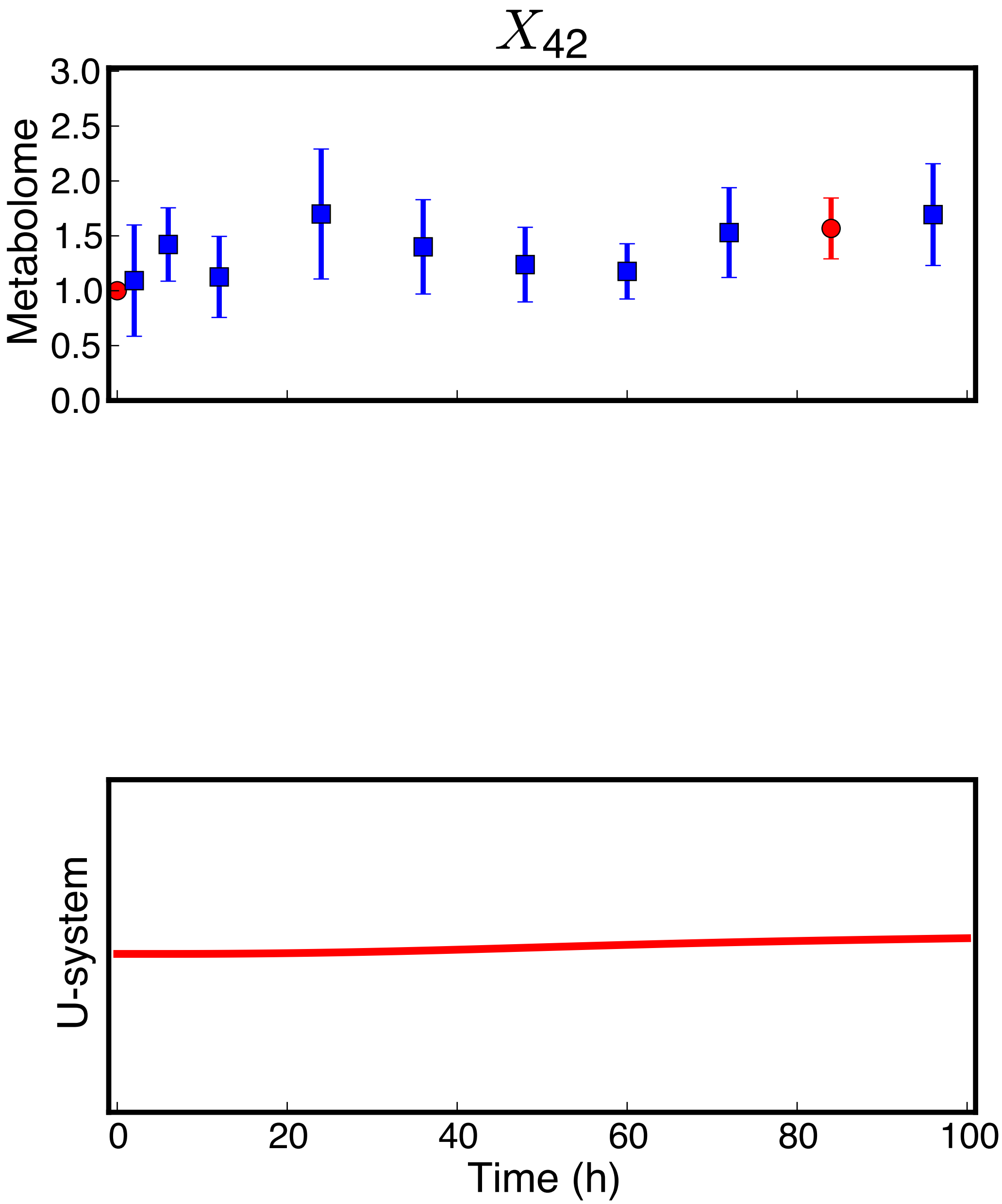

X_{42}
Metabolome
3.0
2.5
2.0
1.5
1.0
0.5
0.0
U-system
0
20
40
60
80
100
Time (h)

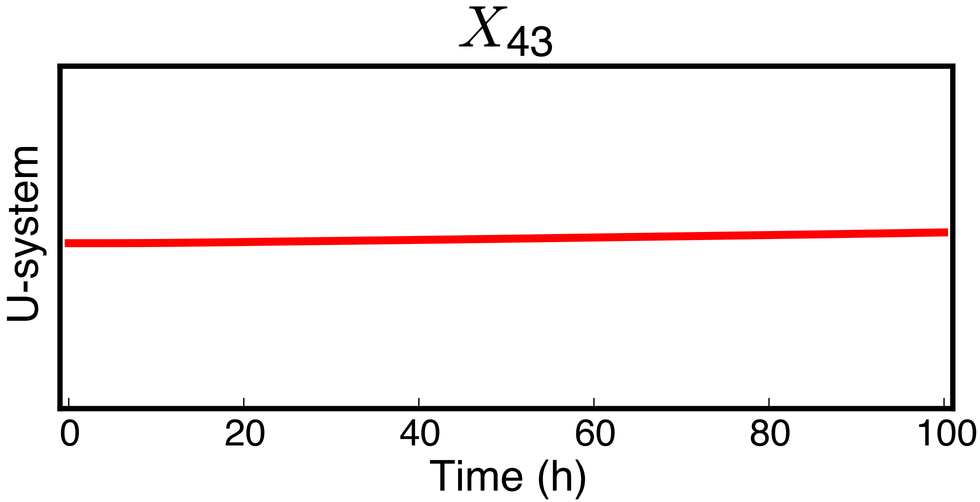

X_{43}
U-system
0
20
40
60
80
100
Time (h)

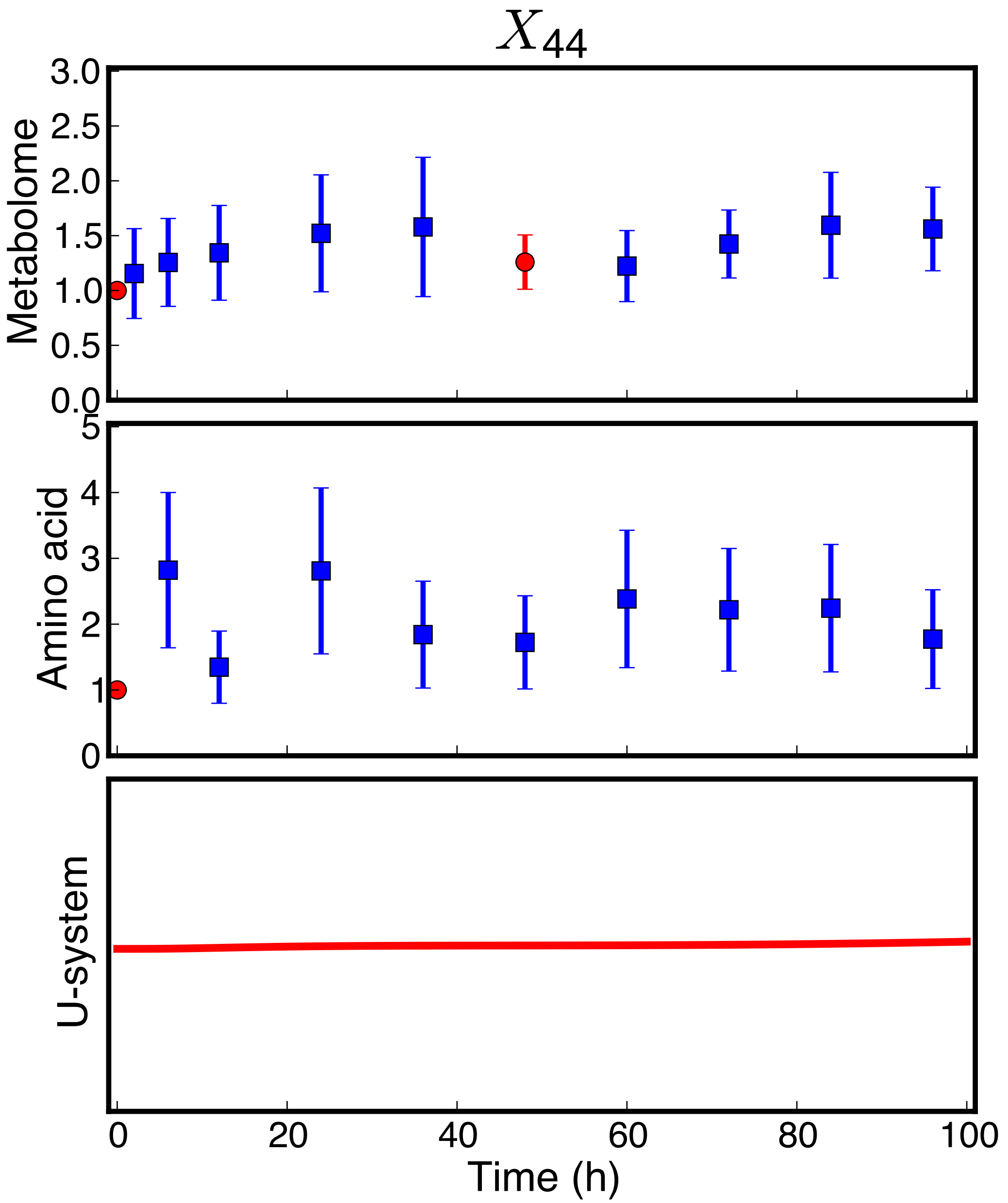

X_{44}
Metabolome
3.0
2.5
2.0
1.5
1.0
0.5
0.0
Amino acid
5
4
3
2
1
0
U-system
0
20
40
60
80
100
Time (h)

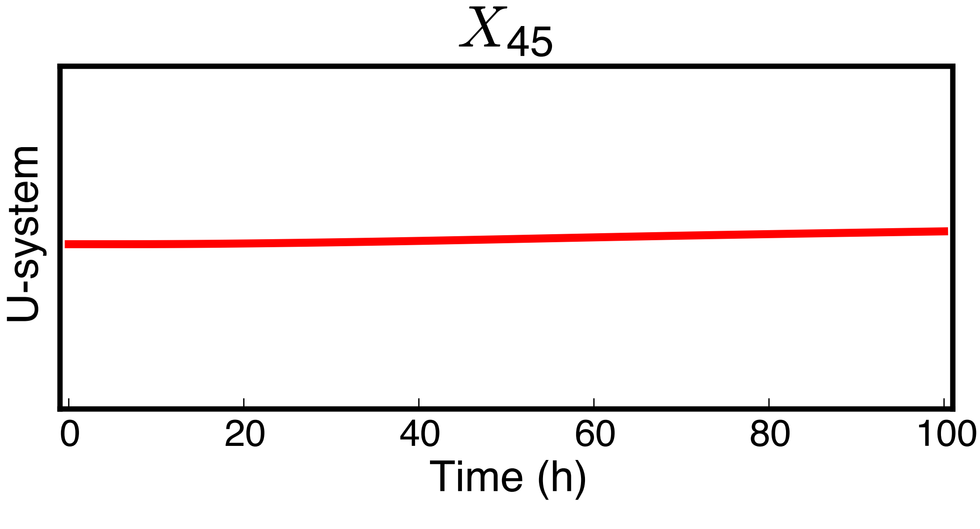

X_{45}
U-system
0
20
40
60
80
100
Time (h)

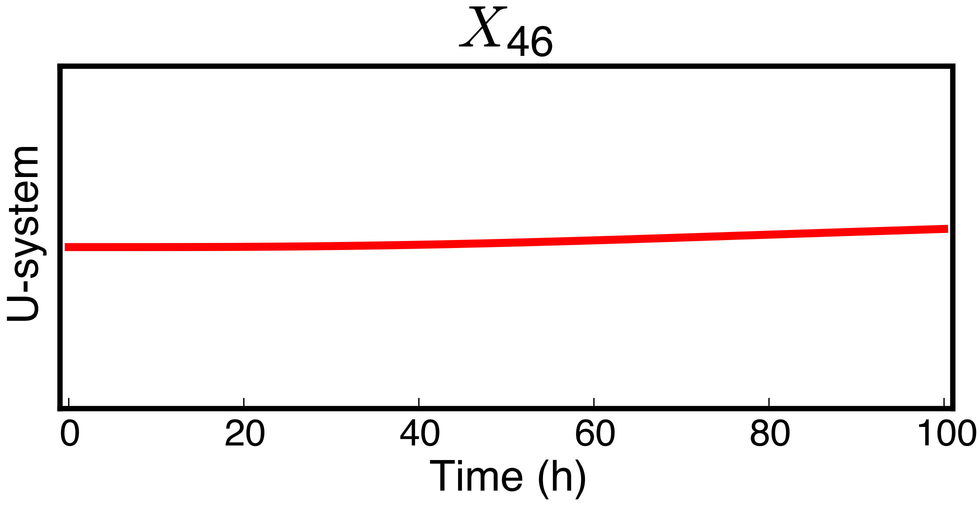
X_{46}
U-system
0
20
40
60
80
100
Time (h)

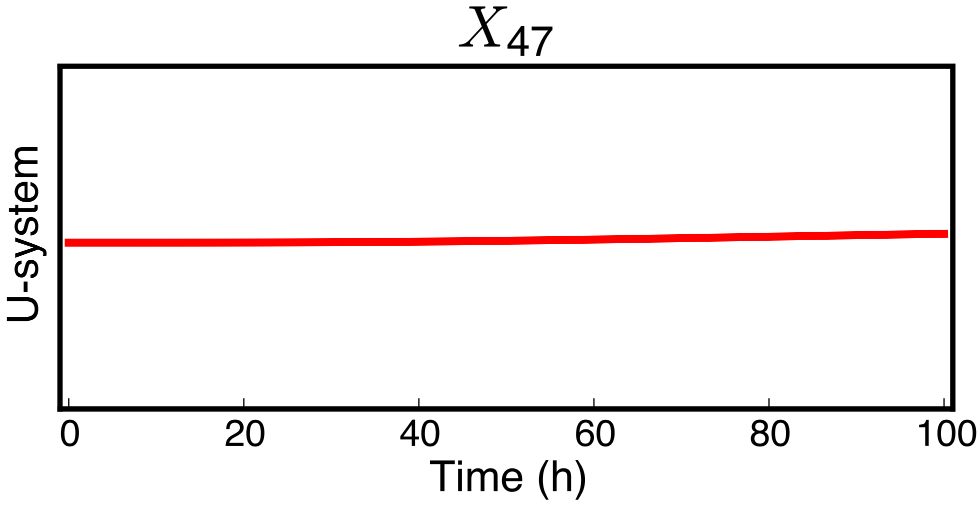

X_{47}
U-system
0
20
40
60
80
100
Time (h)

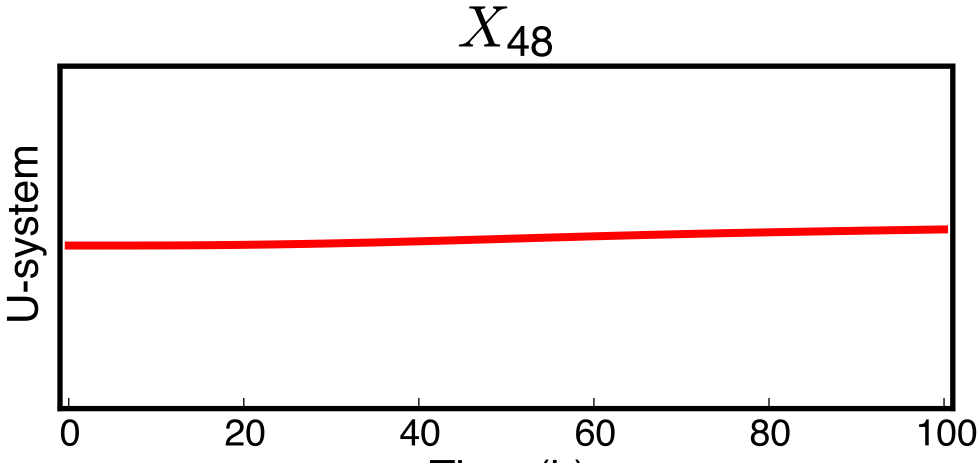
X_{48}
U-system
0
20
40
60
80
100
Time (h)

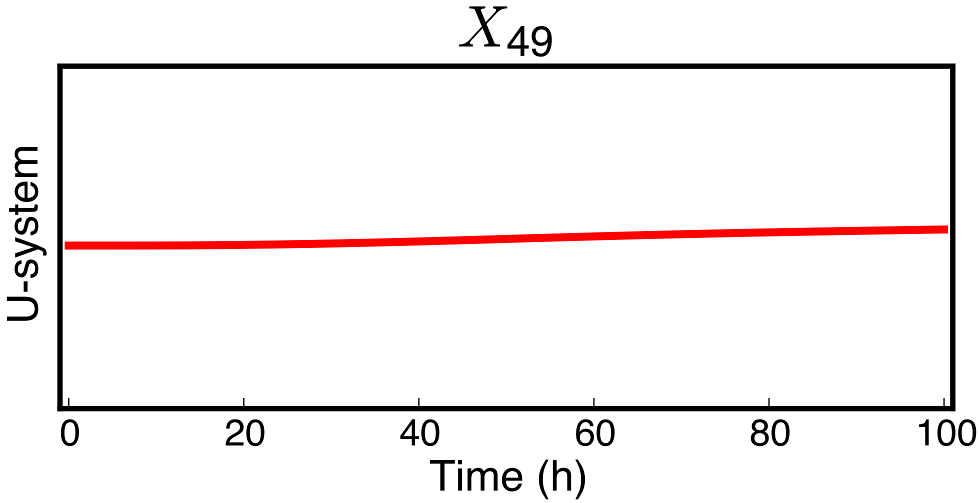
X_{49}
U-system
0
20
40
60
80
100
Time (h)

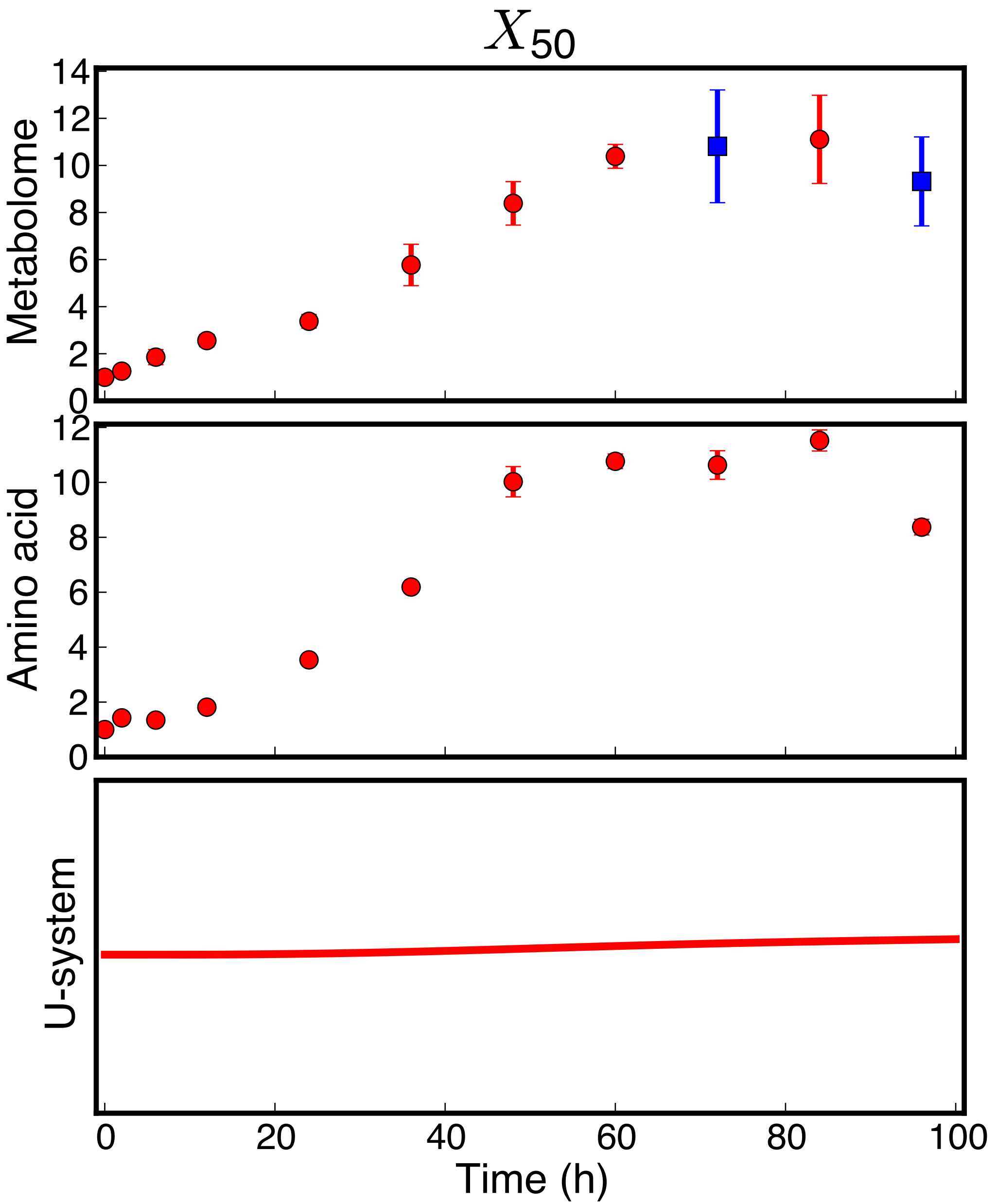

X_{50}
Metabolome
0
2
4
6
8
10
12
14
Amino acid
0
2
4
6
8
10
12
U-system
0
20
40
60
80
100
Time (h)

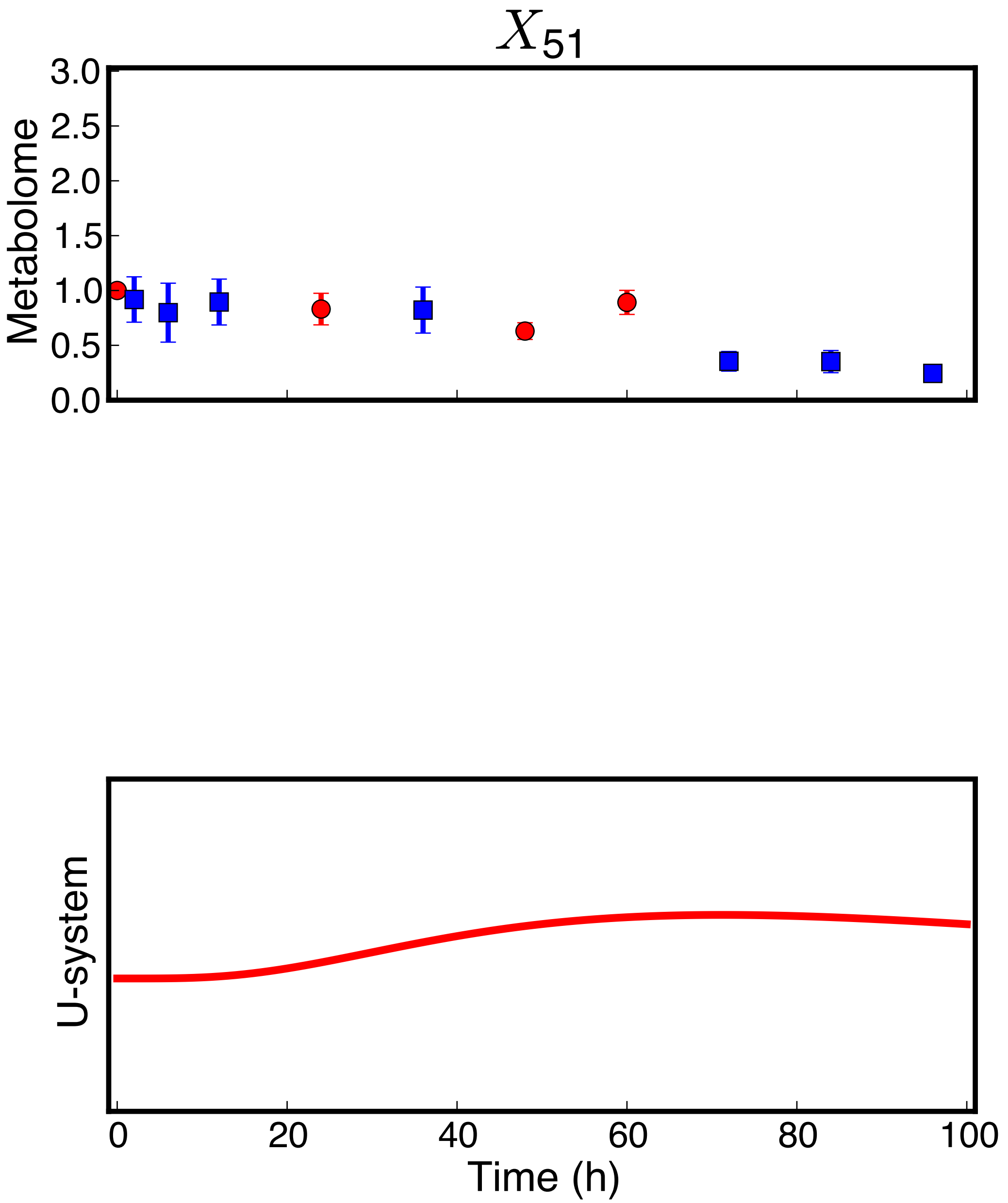
X_{51}
Metabolome
3.0
2.5
2.0
1.5
1.0
0.5
0.0
U-system
0
20
40
60
80
100
Time (h)

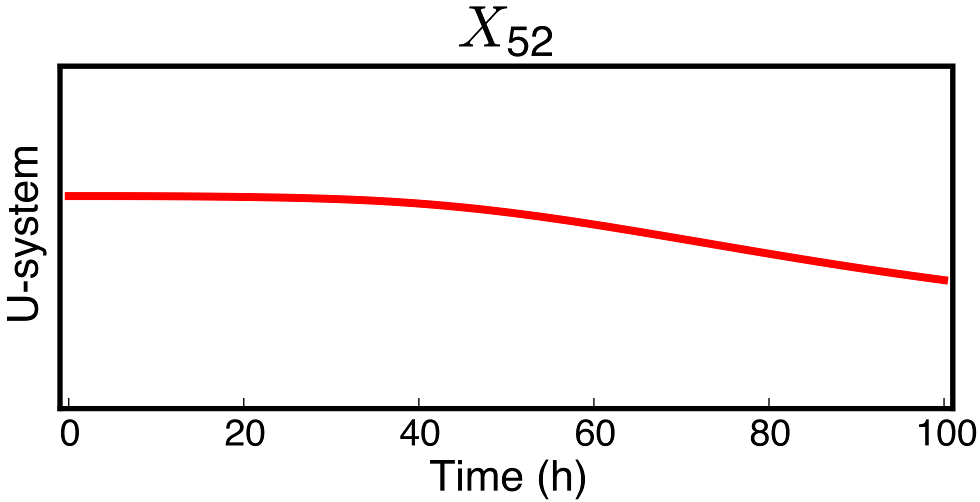

X_{52}
U-system
0
20
40
60
80
100
Time (h)

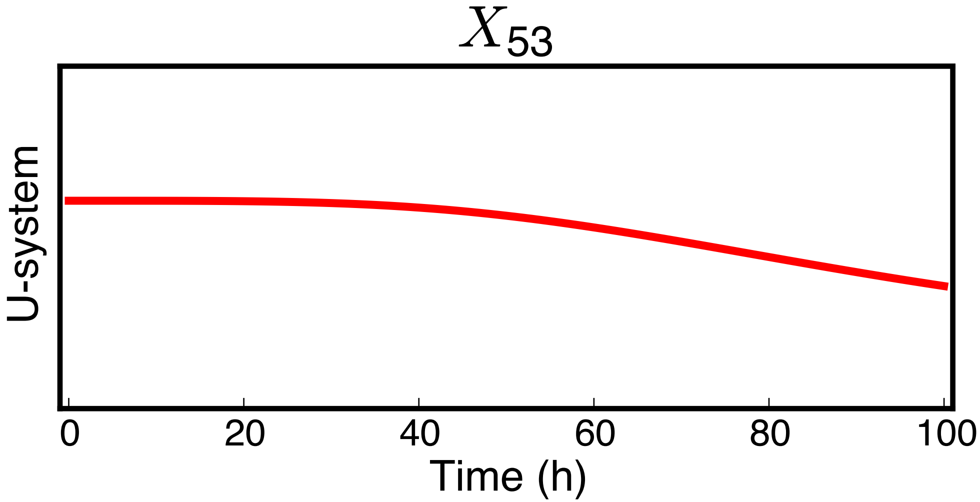
X_{53}
U-system
0
20
40
60
80
100
Time (h)

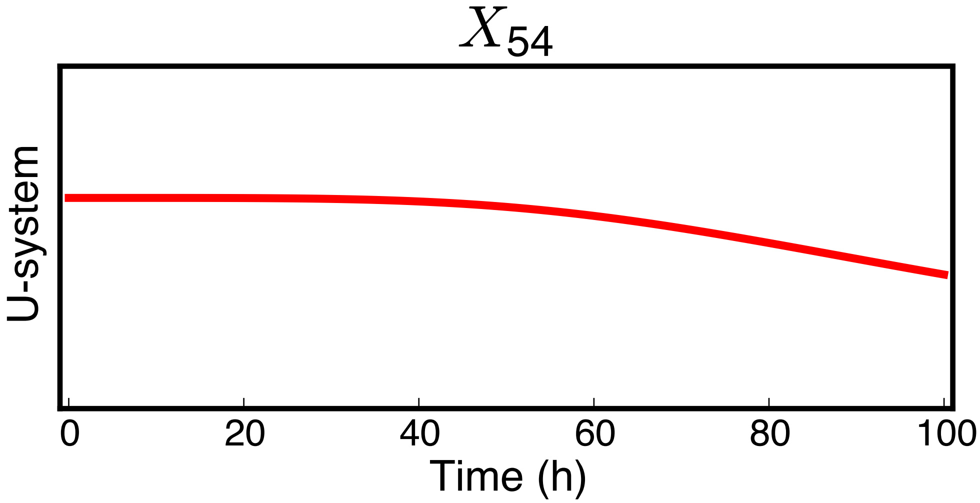

X_{54}
U-system
0
20
40
60
80
100
Time (h)

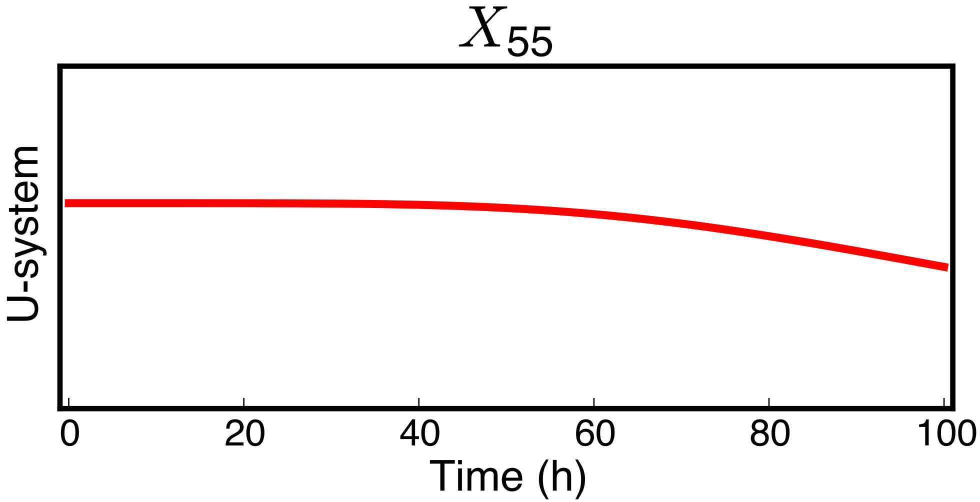
X_{55}
U-system
0
20
40
60
80
100
Time (h)

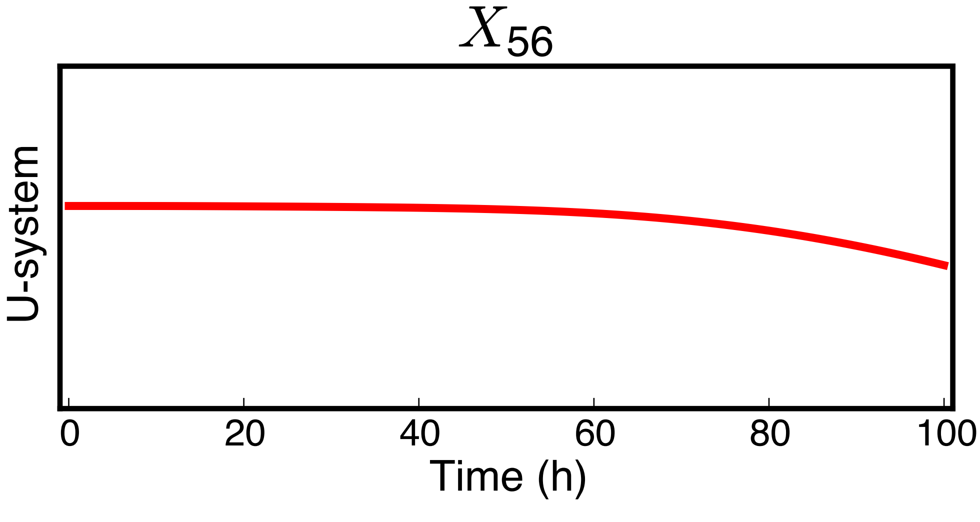

X_{56}
U-system
0
20
40
60
80
100
Time (h)

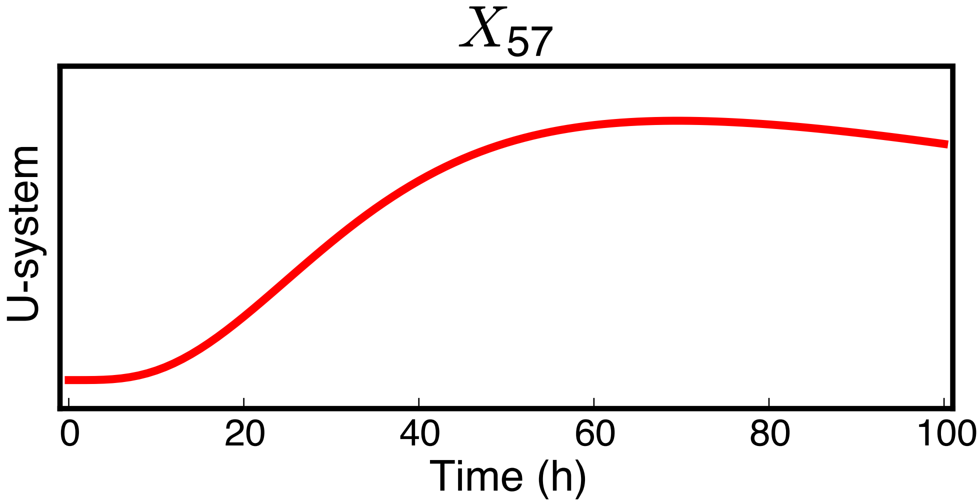

X_{57}
U-system
0
20
40
60
80
100
Time (h)

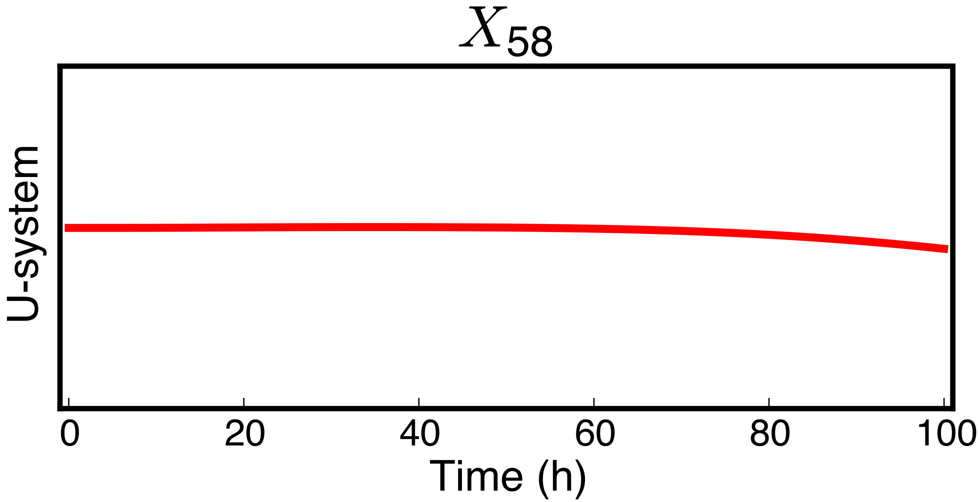

X_{58}
U-system
0
20
40
60
80
100
Time (h)

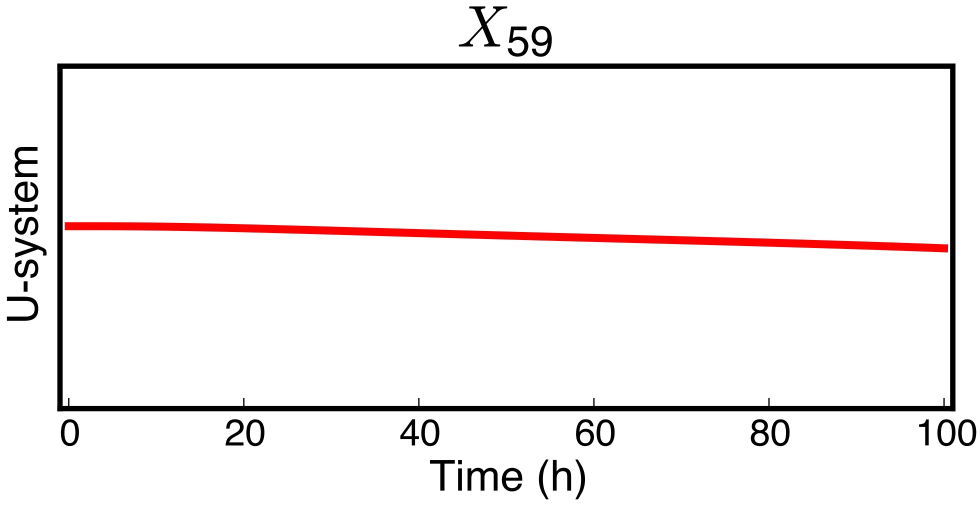

X_{59}
U-system
0
20
40
60
80
100
Time (h)

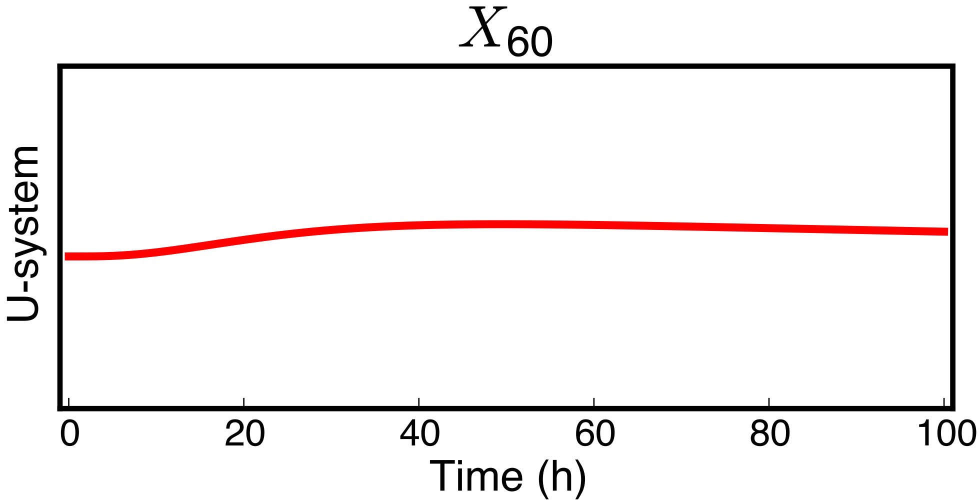
X_{60}
U-system
0
20
40
60
80
100
Time (h)

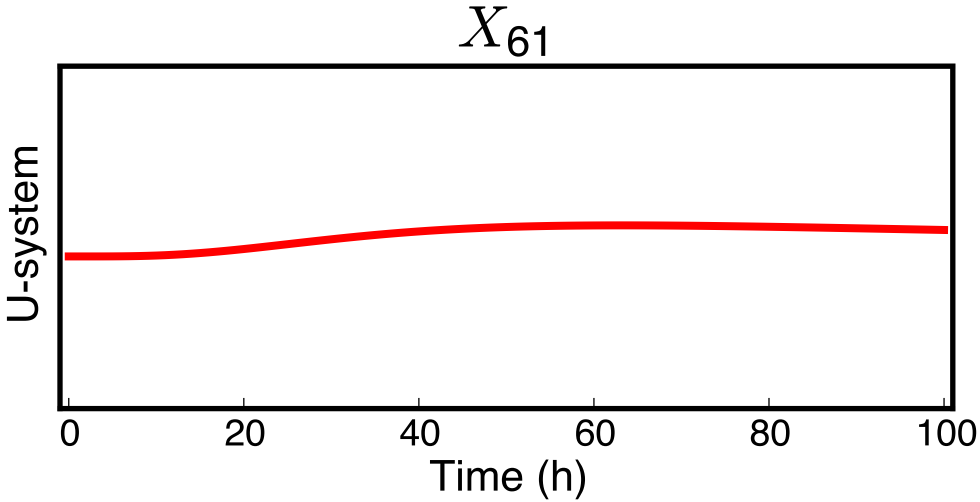

X_{61}
U-system
0
20
40
60
80
100
Time (h)

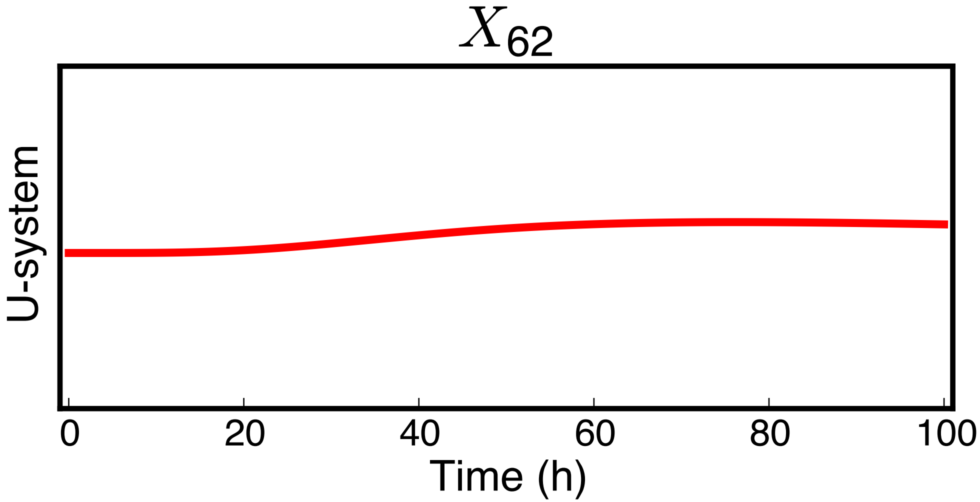

X_{62}
U-system
0
20
40
60
80
100
Time (h)

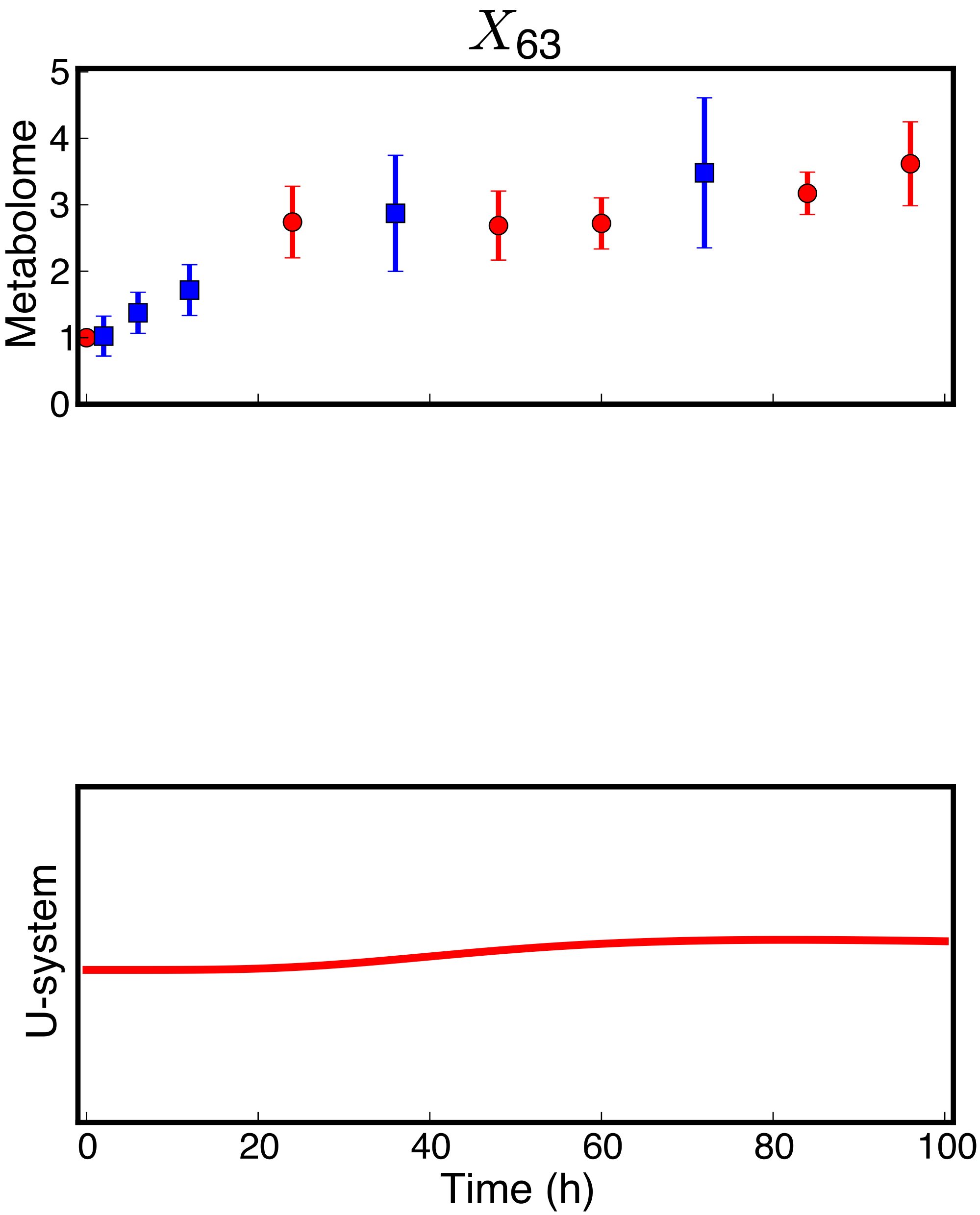

X_{63}
Metabolome
0
1
2
3
4
5
U-system
0
20
40
60
80
100
Time (h)

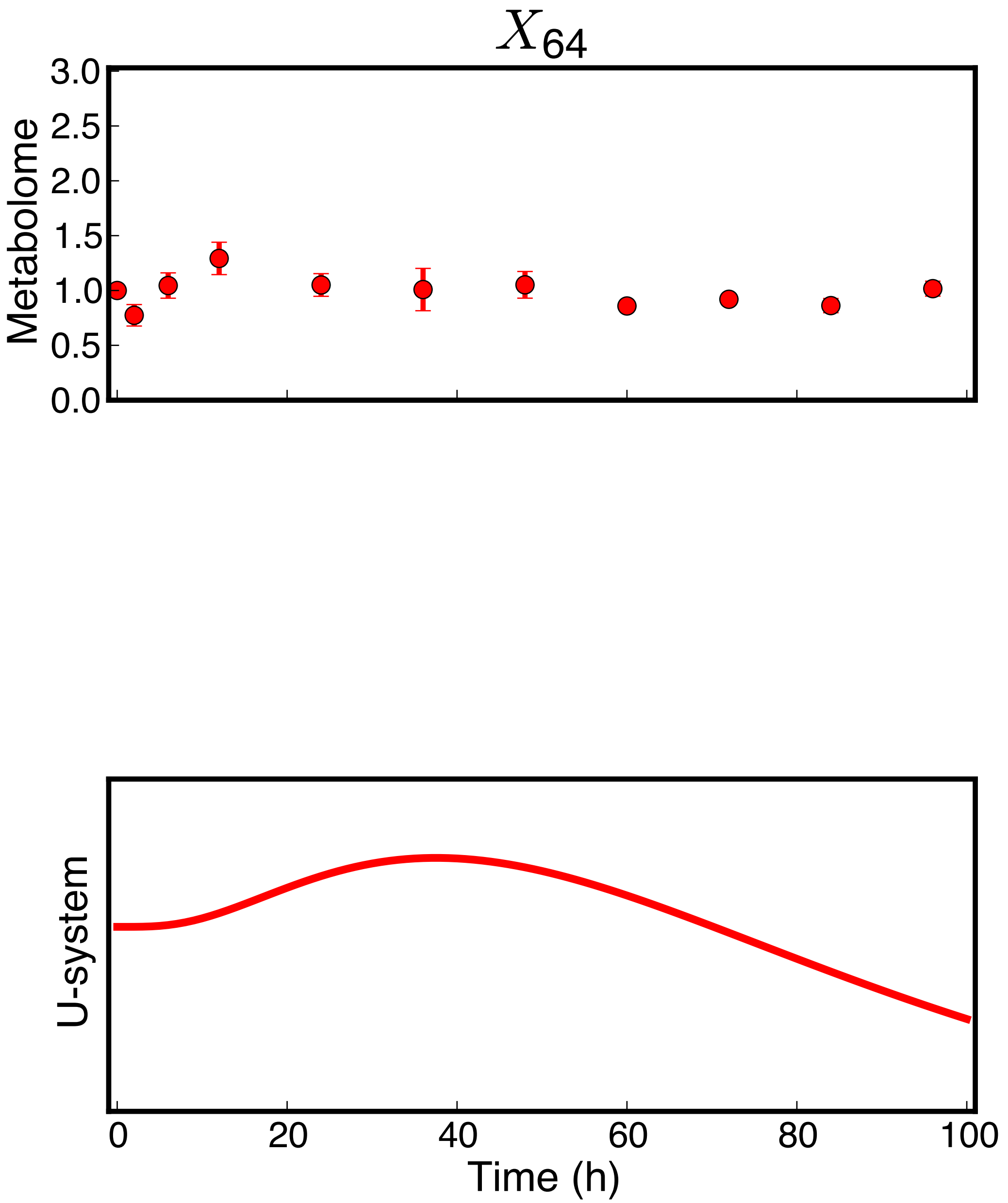
X_{64}
Metabolome
3.0
2.5
2.0
1.5
1.0
0.5
0.0
U-system
0
20
40
60
80
100
Time (h)

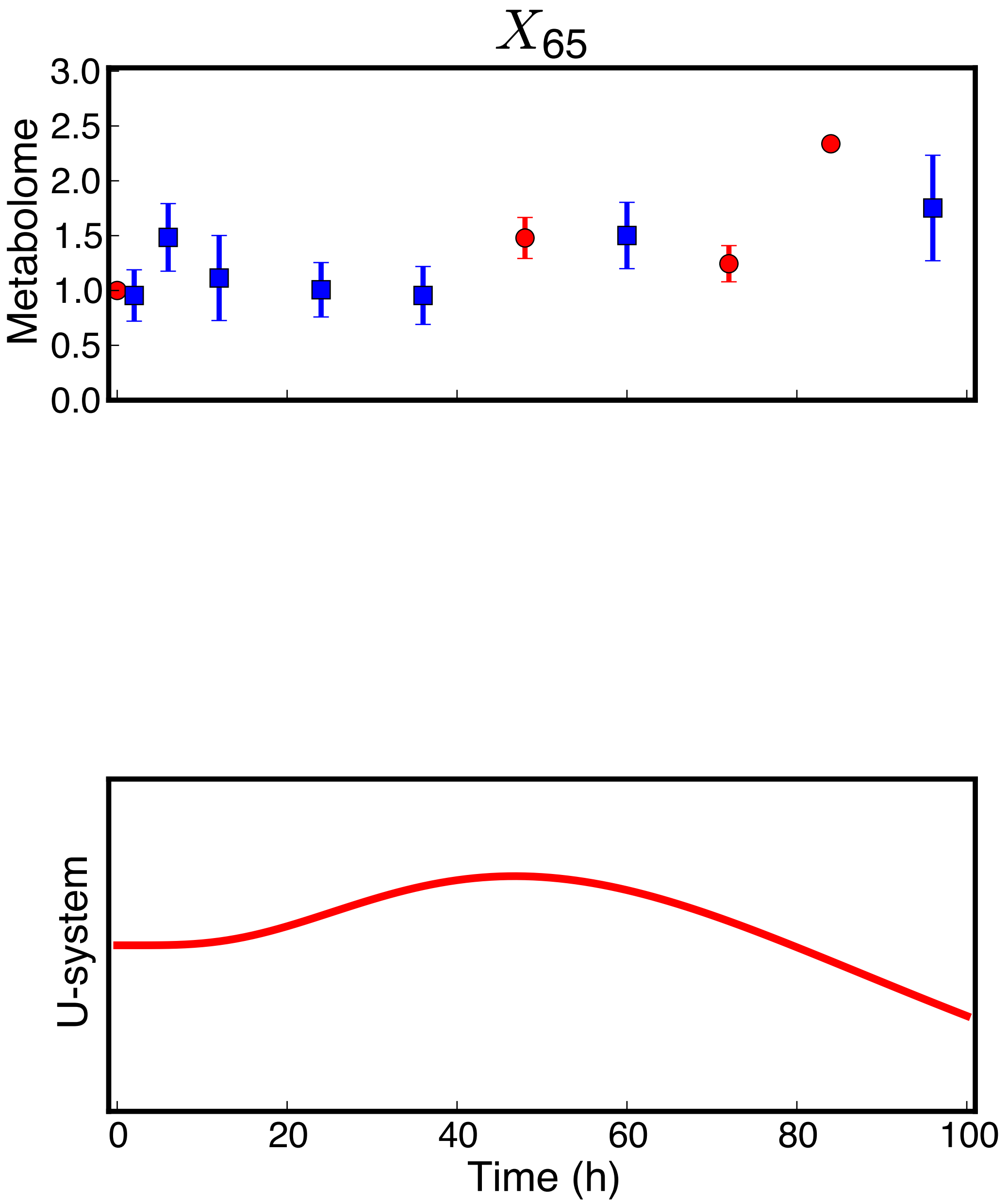

X_{65}
Metabolome
3.0
2.5
2.0
1.5
1.0
0.5
0.0
U-system
0
20
40
60
80
100
Time (h)

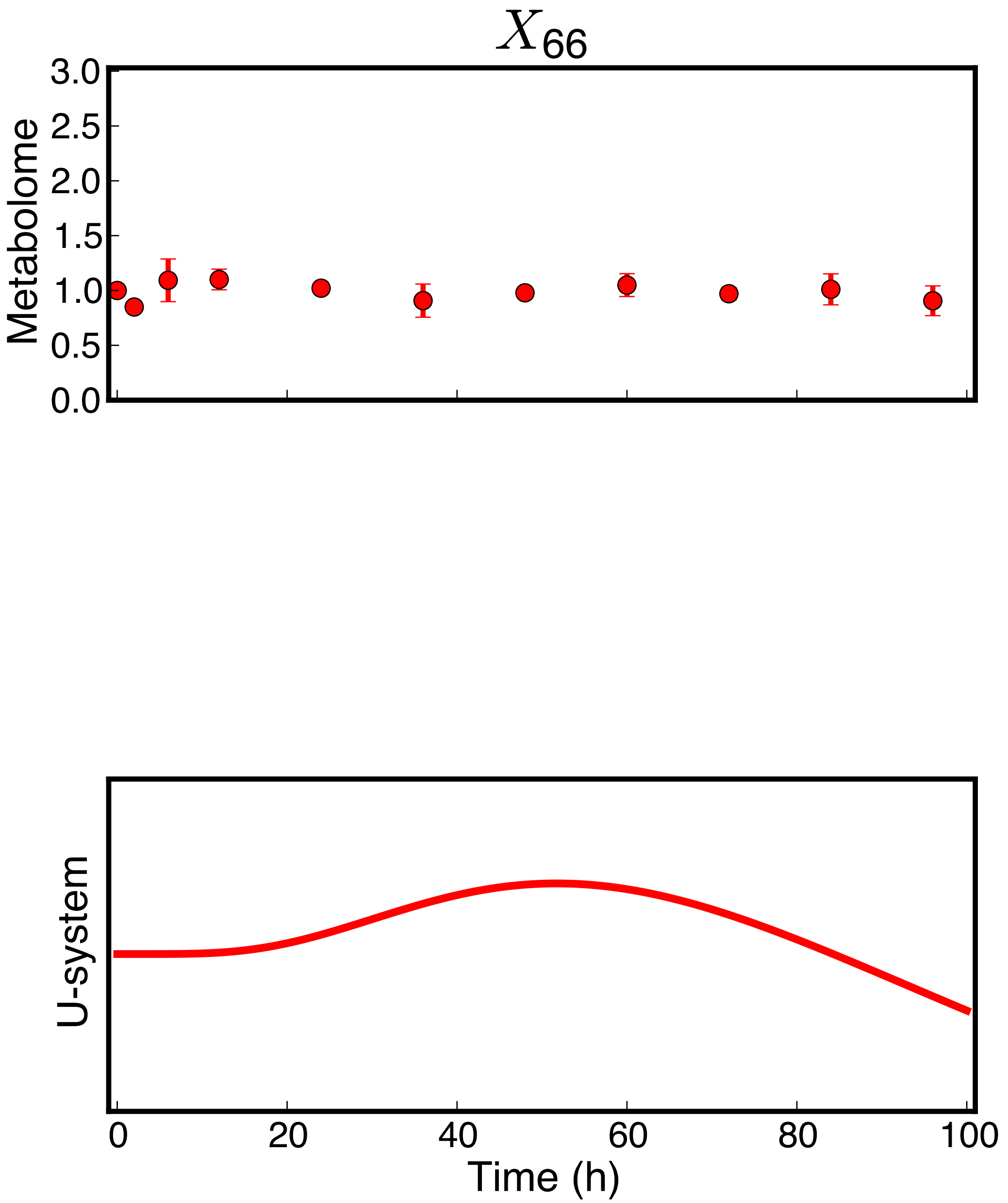

X_{66}
Metabolome
3.0
2.5
2.0
1.5
1.0
0.5
0.0
U-system
0
20
40
60
80
100
Time (h)

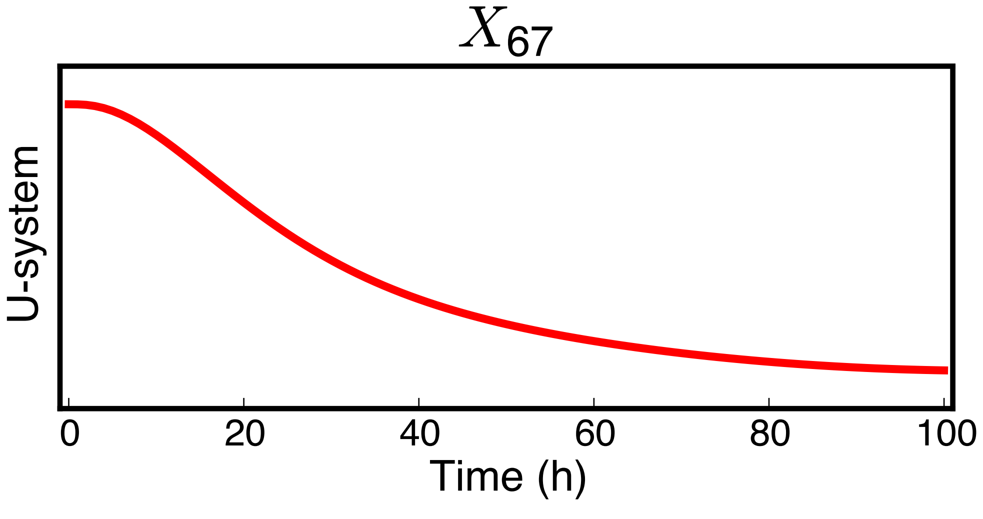

X_{67}
U-system
0
20
40
60
80
100
Time (h)

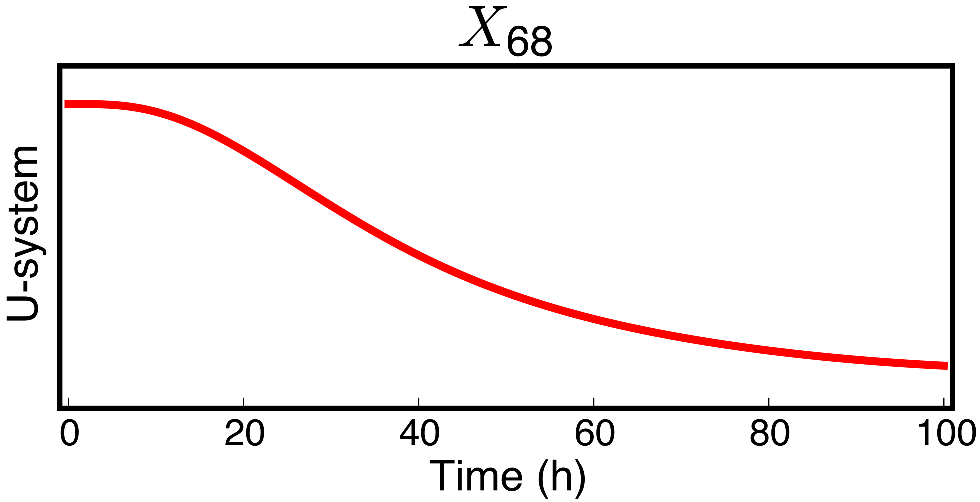

X_{68}
U-system
0
20
40
60
80
100
Time (h)

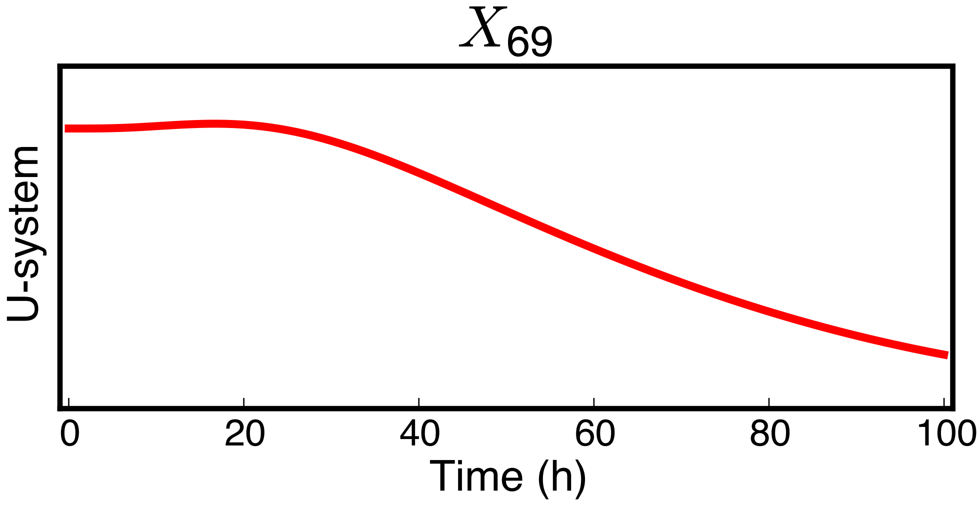

X_{69}
U-system
0
20
40
60
80
100
Time (h)

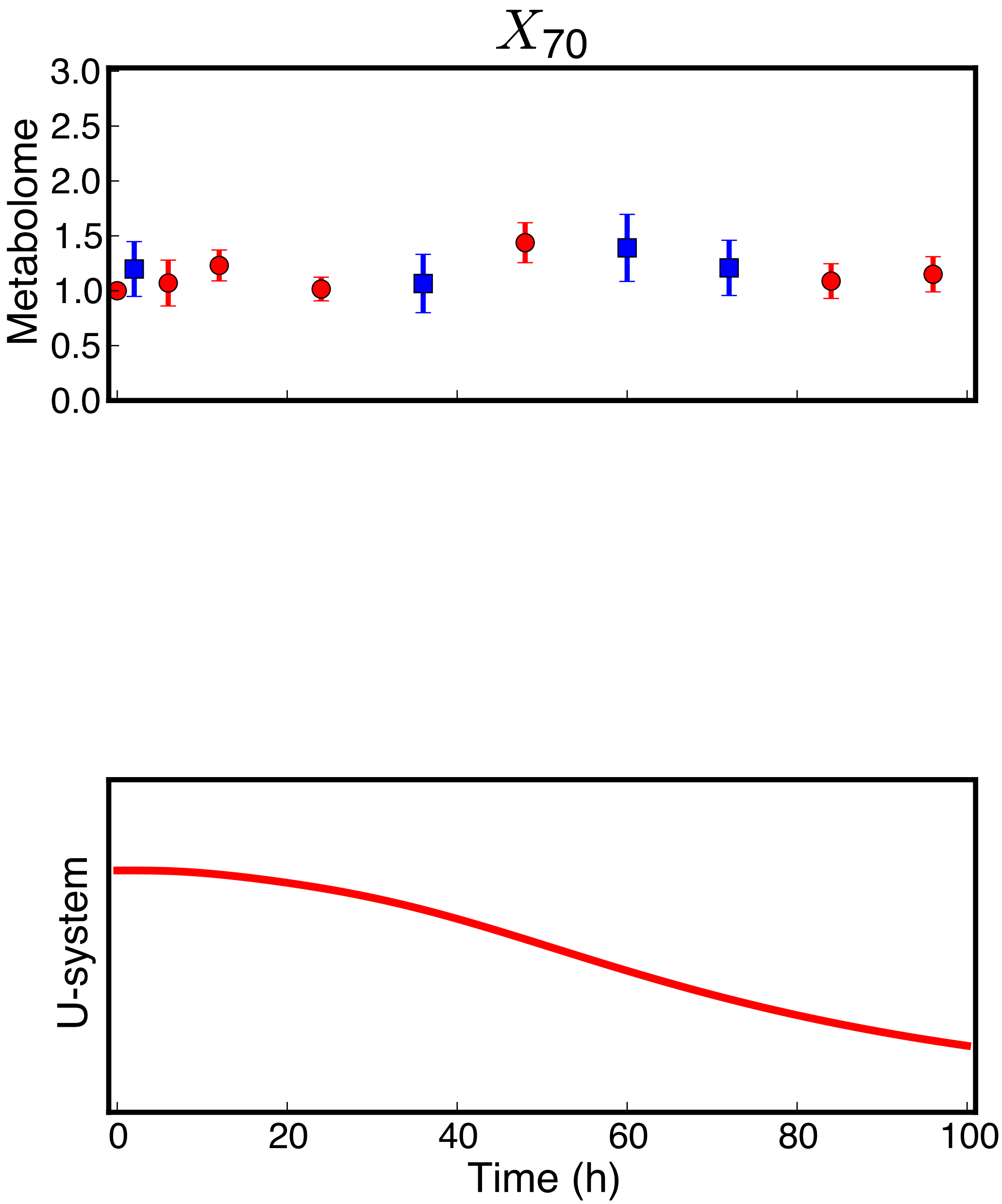
X_{70}
Metabolome
3.0
2.5
2.0
1.5
1.0
0.5
0.0
U-system
0
20
40
60
80
100
Time (h)

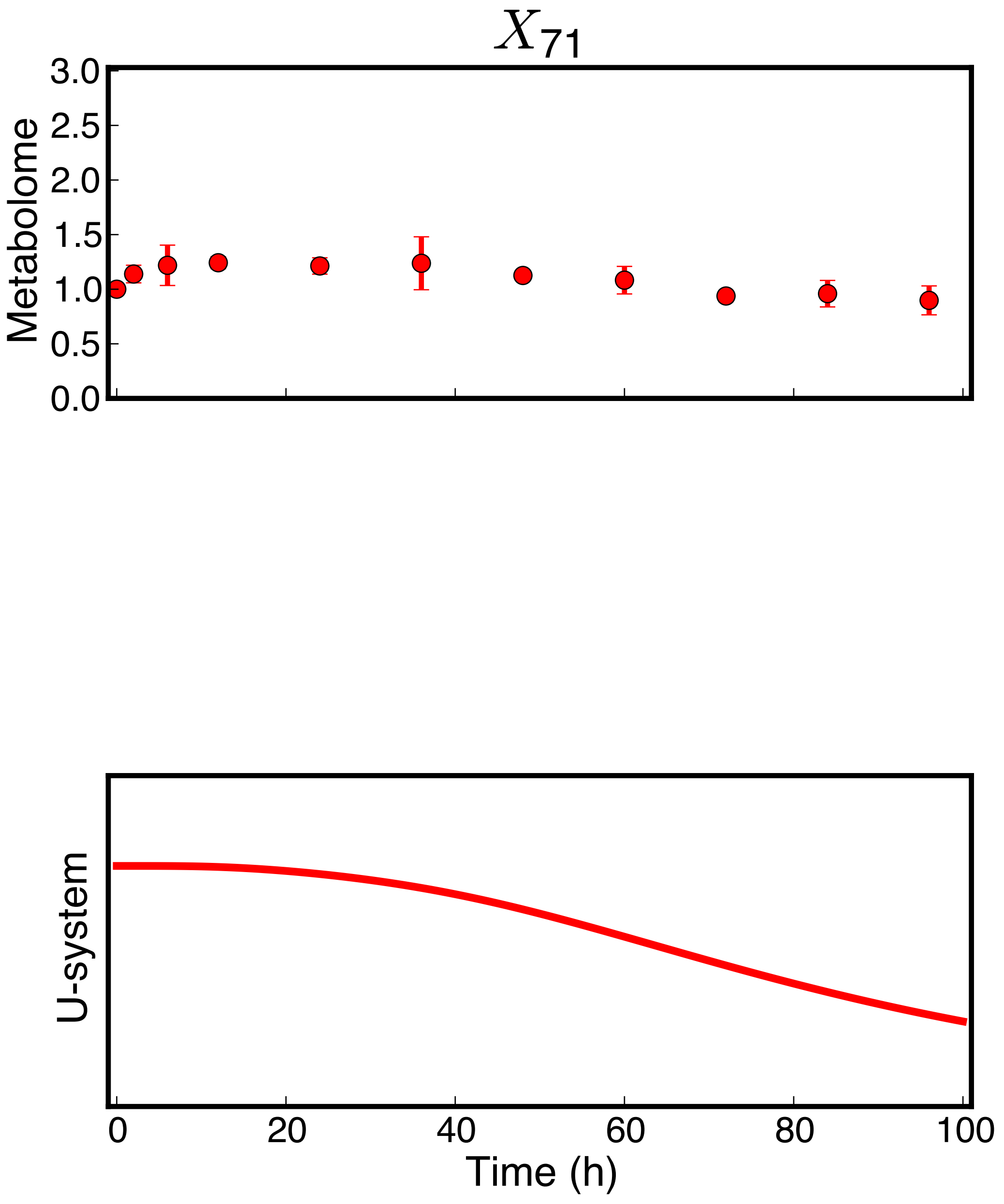

X_{71}
Metabolome
3.0
2.5
2.0
1.5
1.0
0.5
0.0
U-system
0
20
40
60
80
100
Time (h)

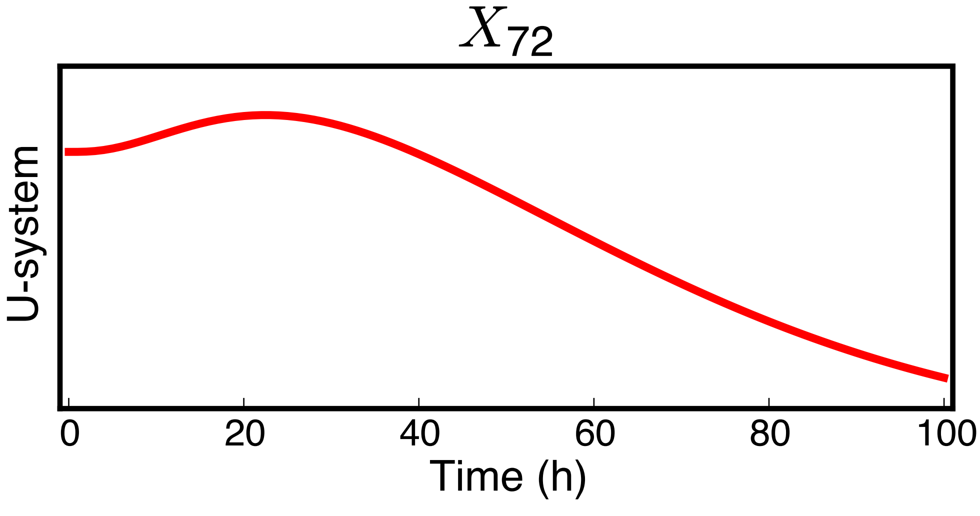
X_{72}
U-system
0
20
40
60
80
100
Time (h)

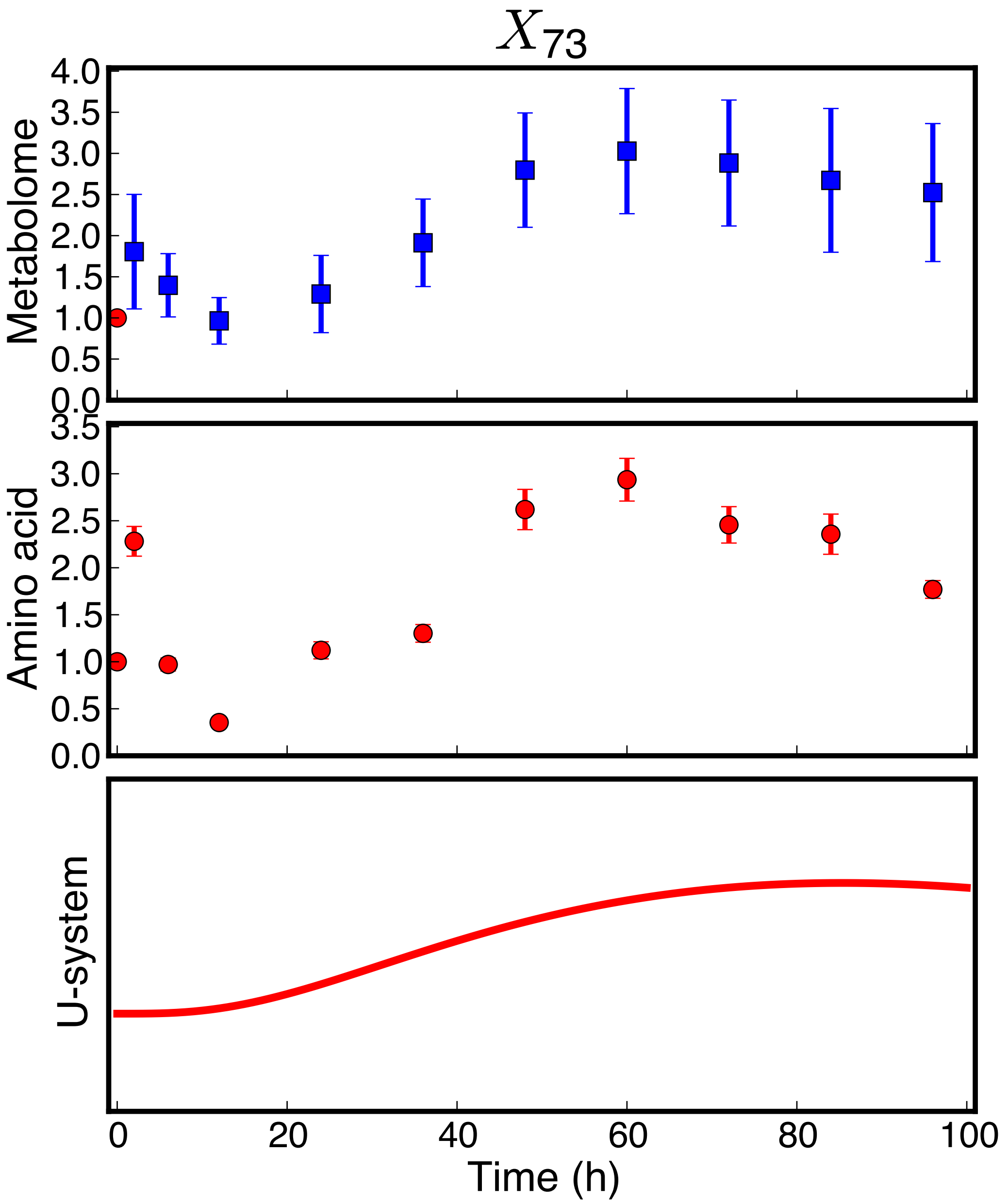
X_{73}
Metabolome
Amino acid
U-system
0.0
0.5
1.0
1.5
2.0
2.5
3.0
3.5
4.0
0
20
40
60
80
100
Time (h)

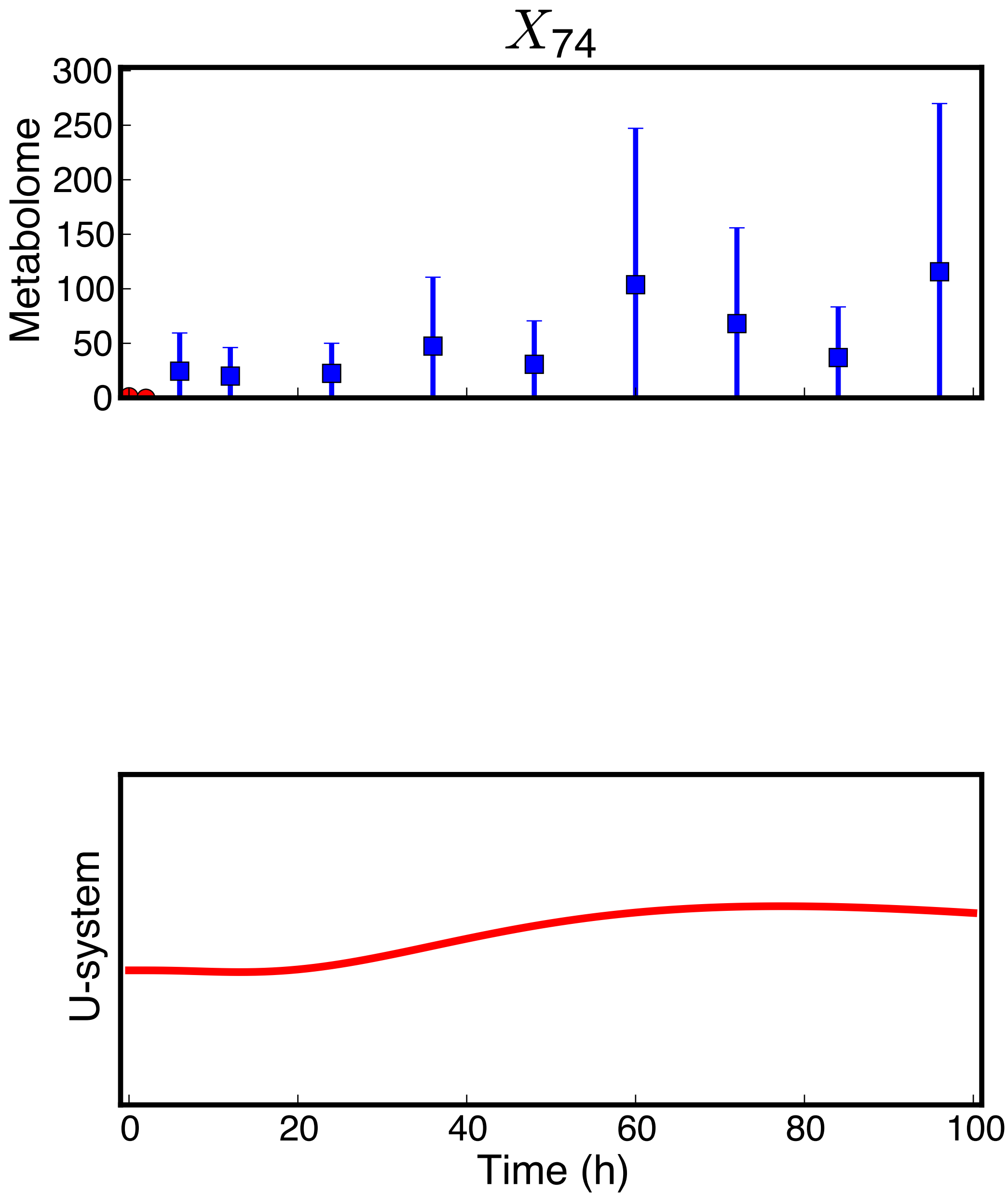

X_{74}
Metabolome
300
250
200
150
100
50
0
U-system
0
20
40
60
80
100
Time (h)

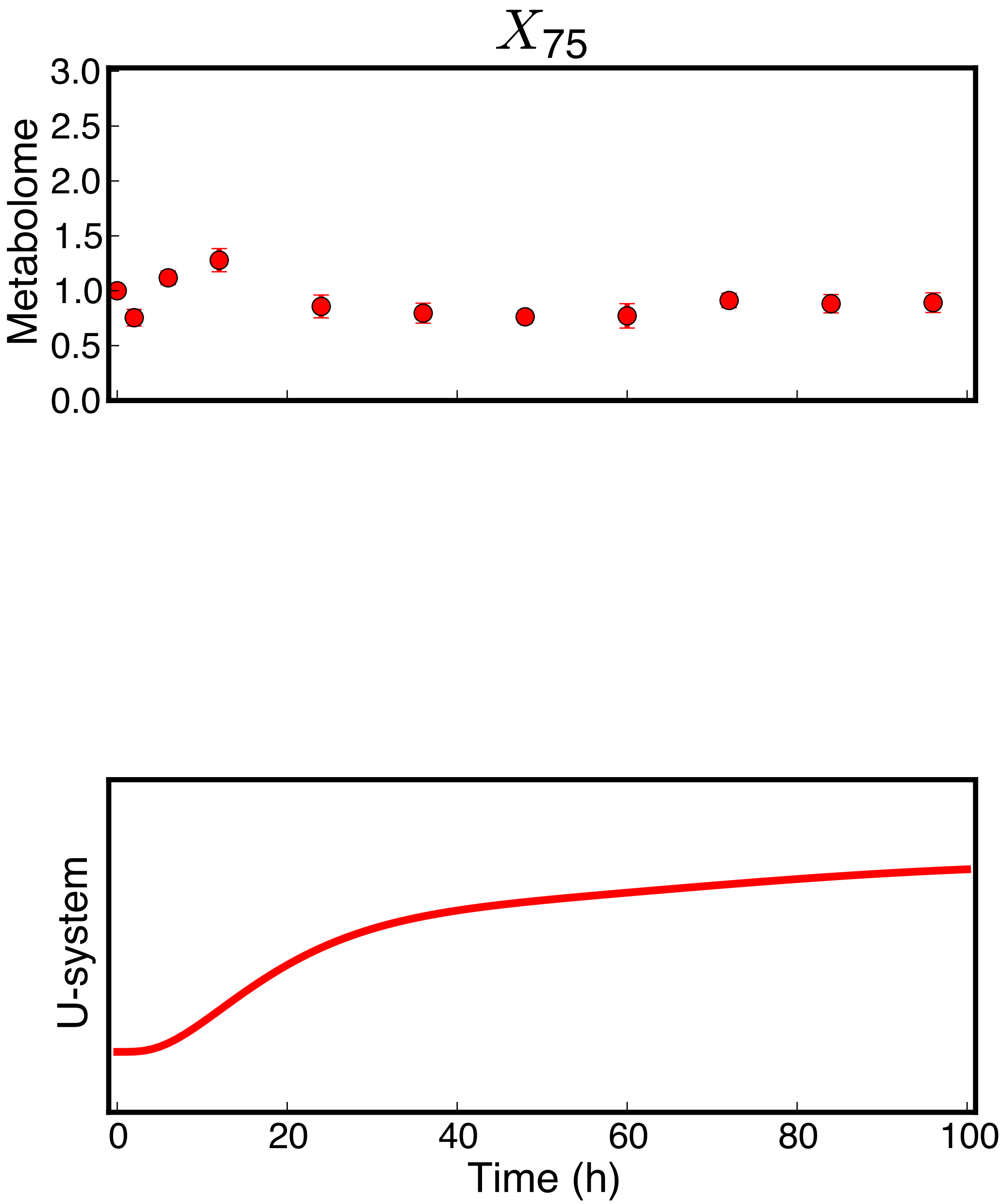

X_{75}
Metabolome
3.0
2.5
2.0
1.5
1.0
0.5
0.0
U-system
0
20
40
60
80
100
Time (h)

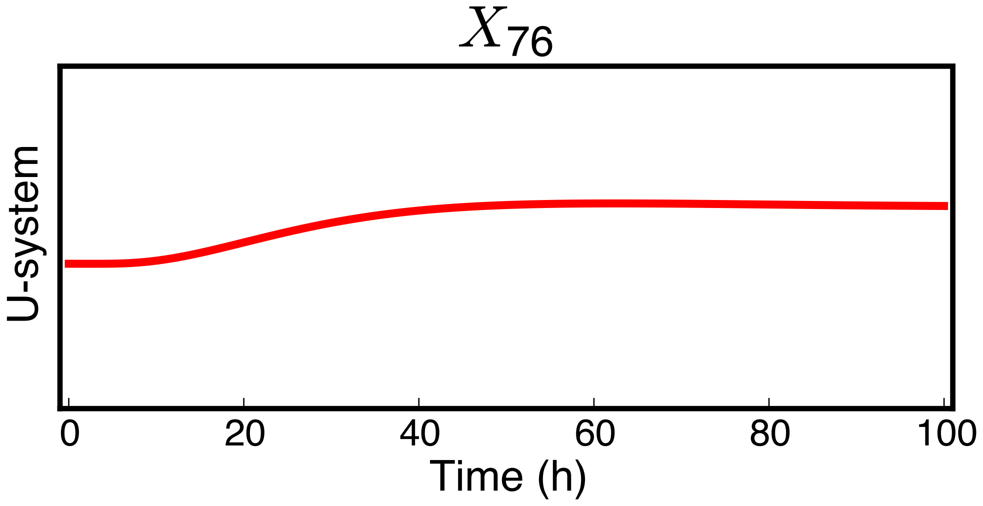

X_{76}
U-system
0
20
40
60
80
100
Time (h)

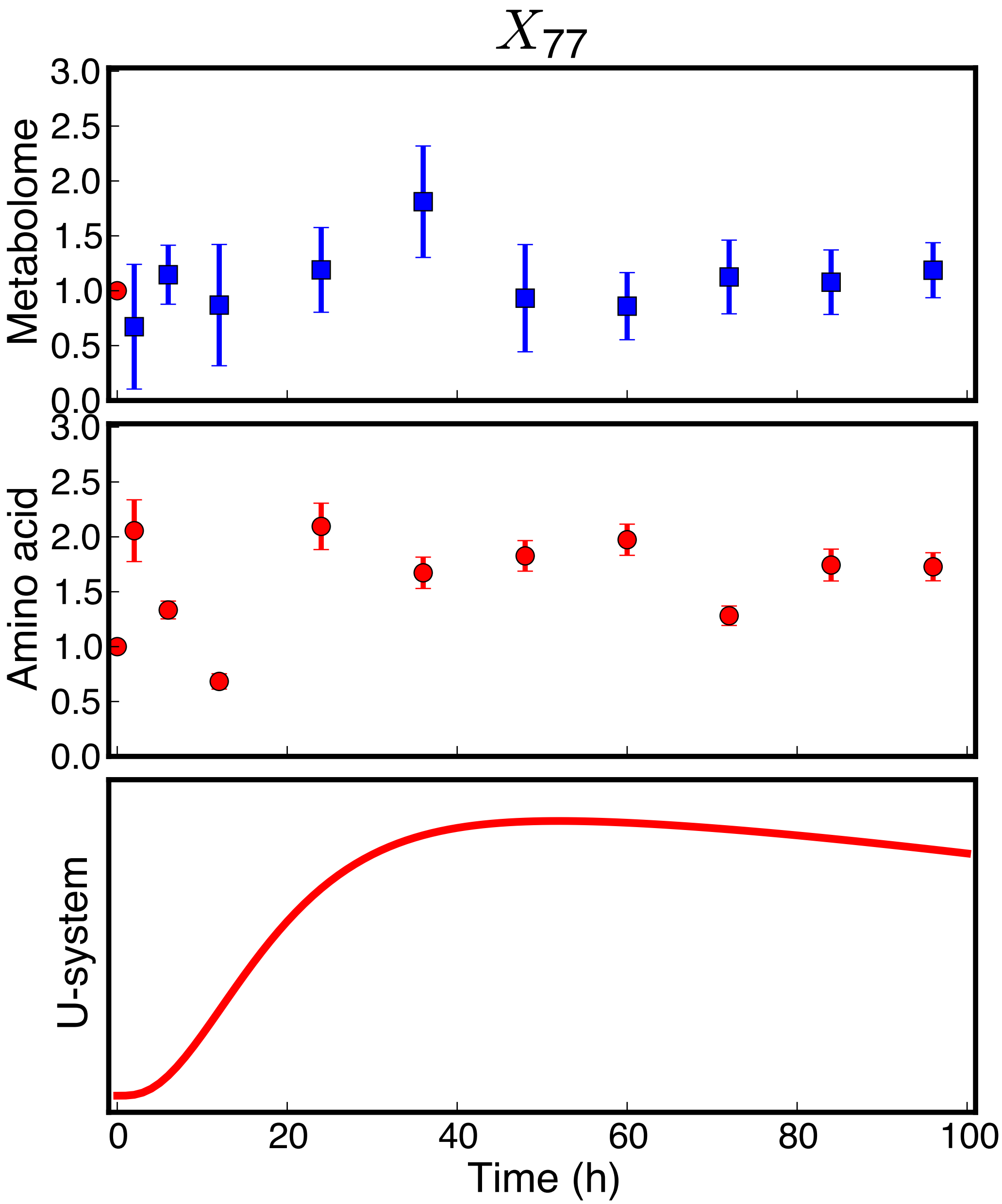

X_{77}
Metabolome
Amino acid
U-system
3.0
2.5
2.0
1.5
1.0
0.5
0.0
0
20
40
60
80
100
Time (h)

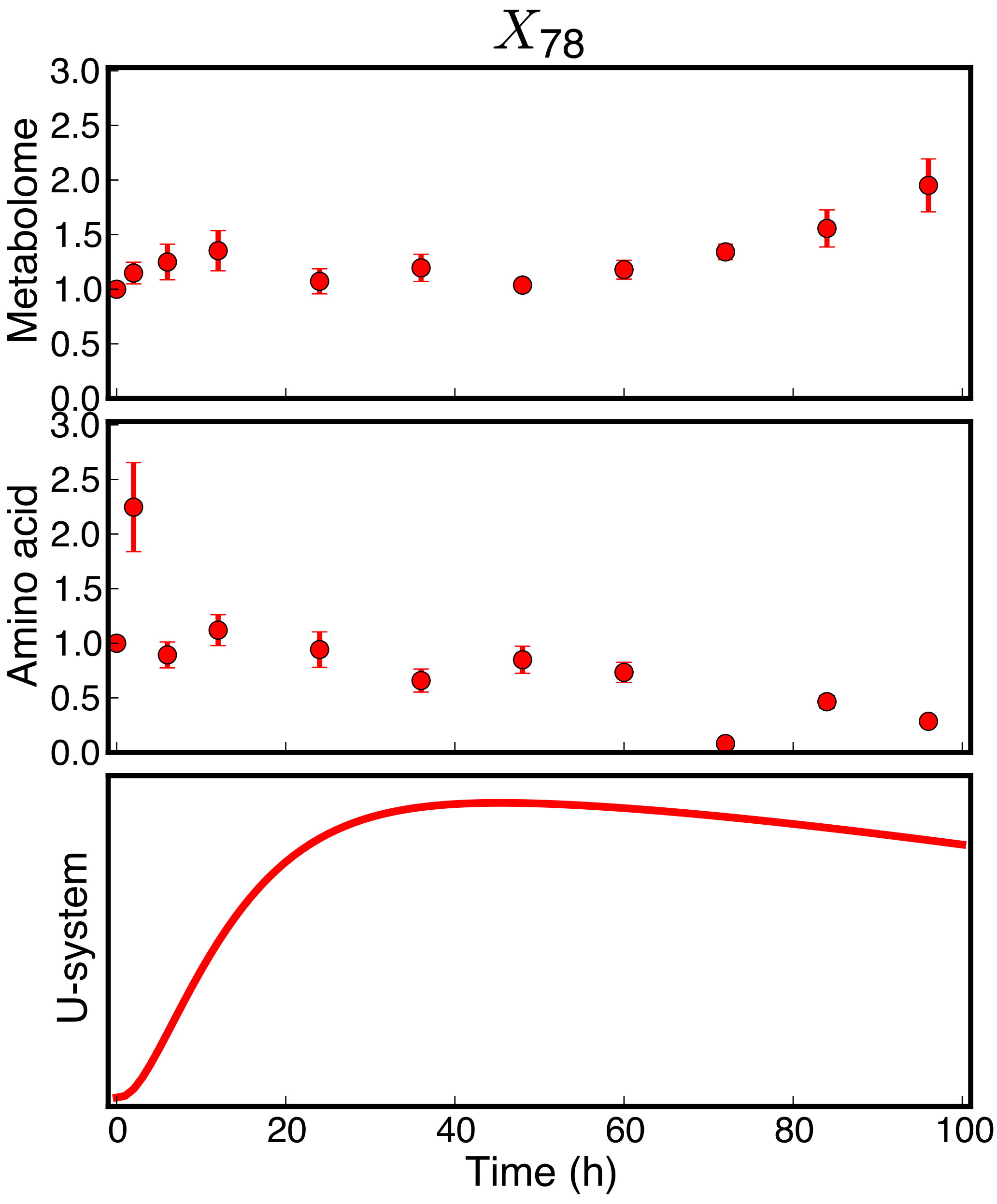

X_{78}
Metabolome
3.0
2.5
2.0
1.5
1.0
0.5
0.0
Amino acid
3.0
2.5
2.0
1.5
1.0
0.5
0.0
U-system
0
20
40
60
80
100
Time (h)

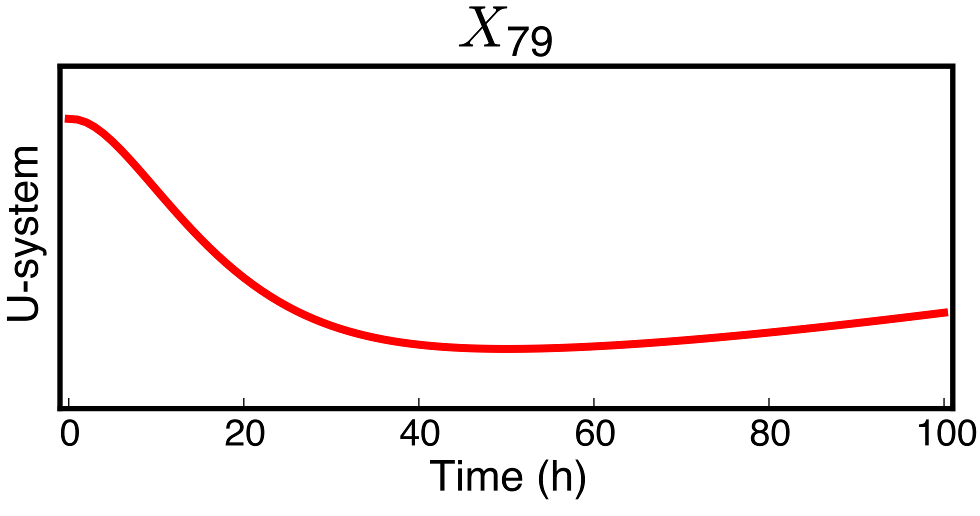
X_{79}
U-system
0
20
40
60
80
100
Time (h)

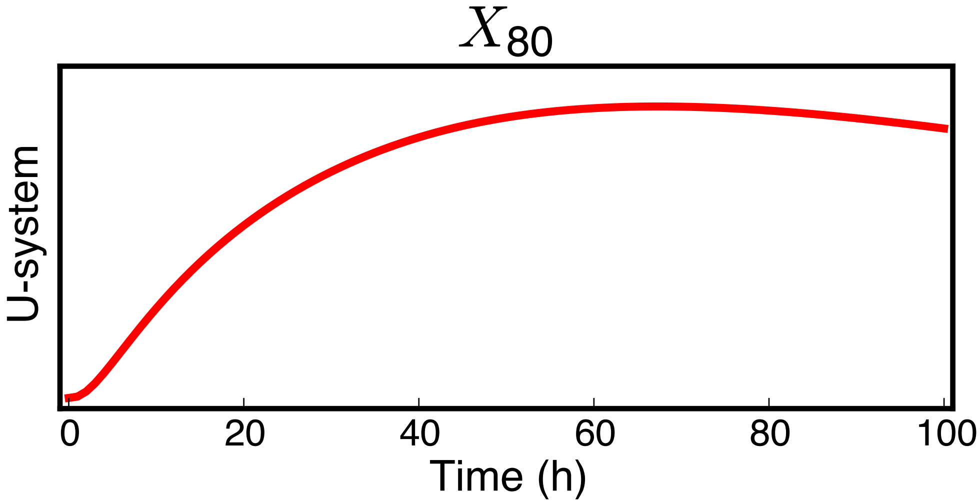

X_{80}
U-system
0
20
40
60
80
100
Time (h)

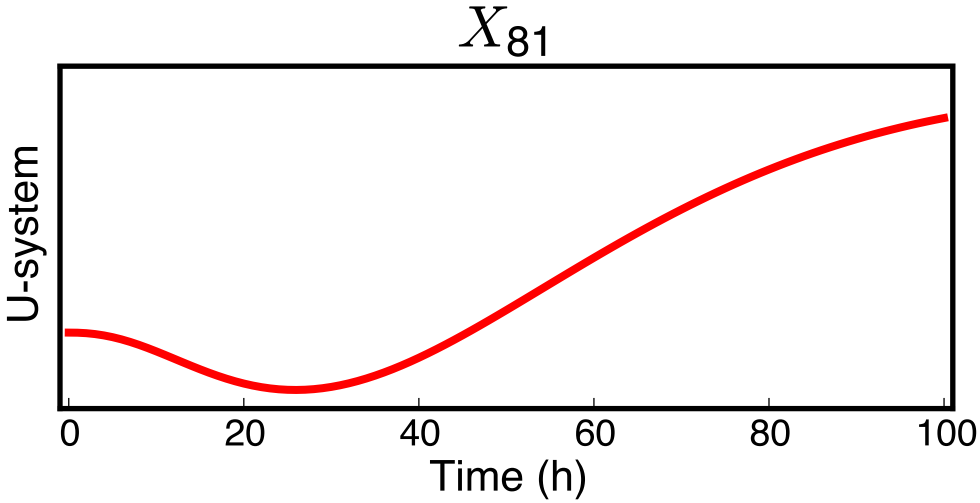
X_{81}
U-system
0
20
40
60
80
100
Time (h)

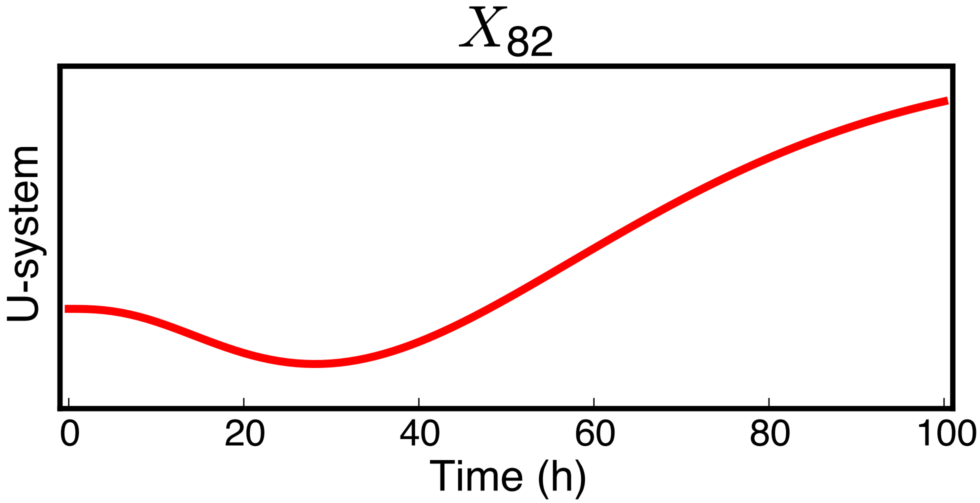

X_{82}
U-system
0
20
40
60
80
100
Time (h)

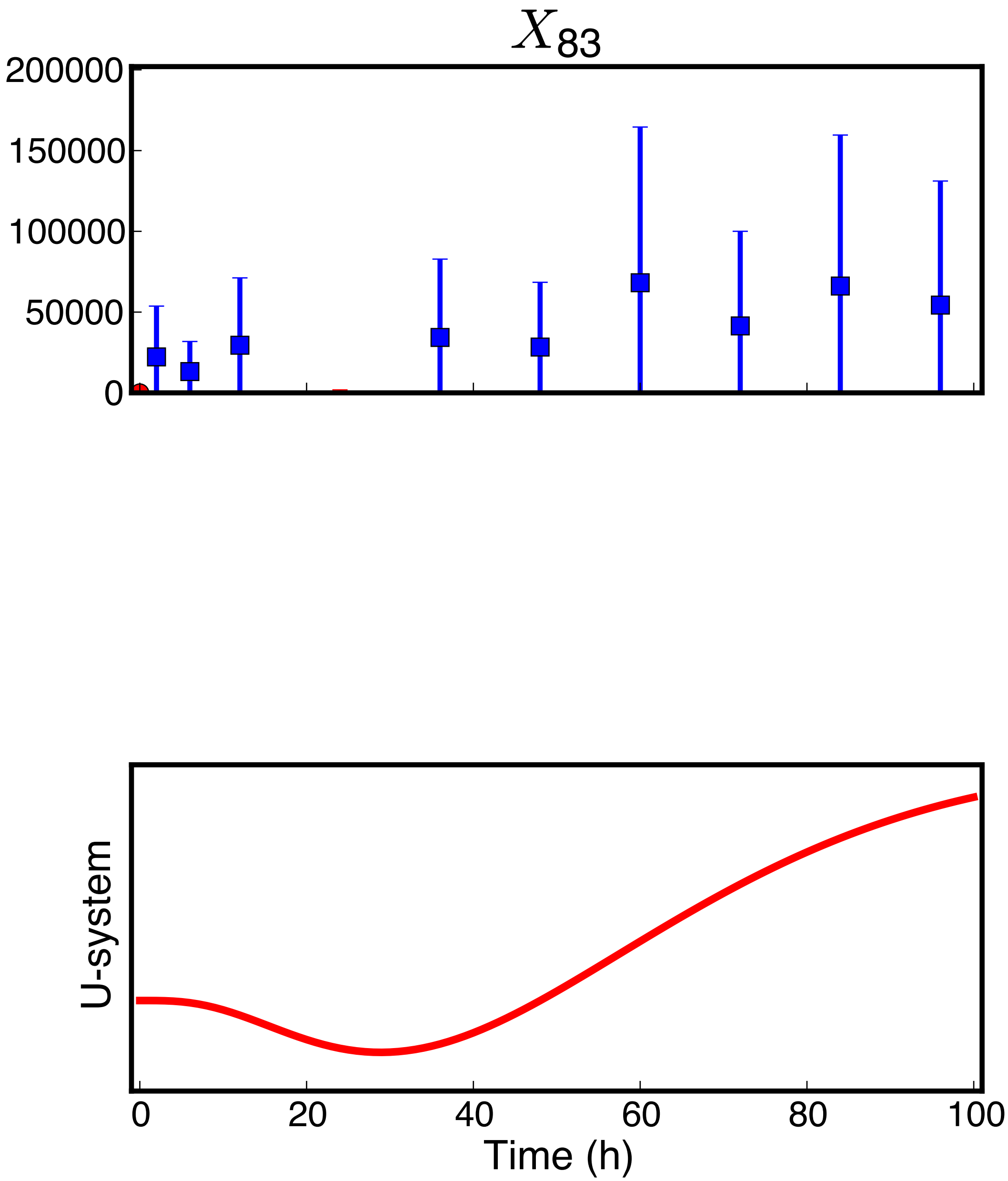

X_{83}
200000
150000
100000
50000
0
U-system
0
20
40
60
80
100
Time (h)

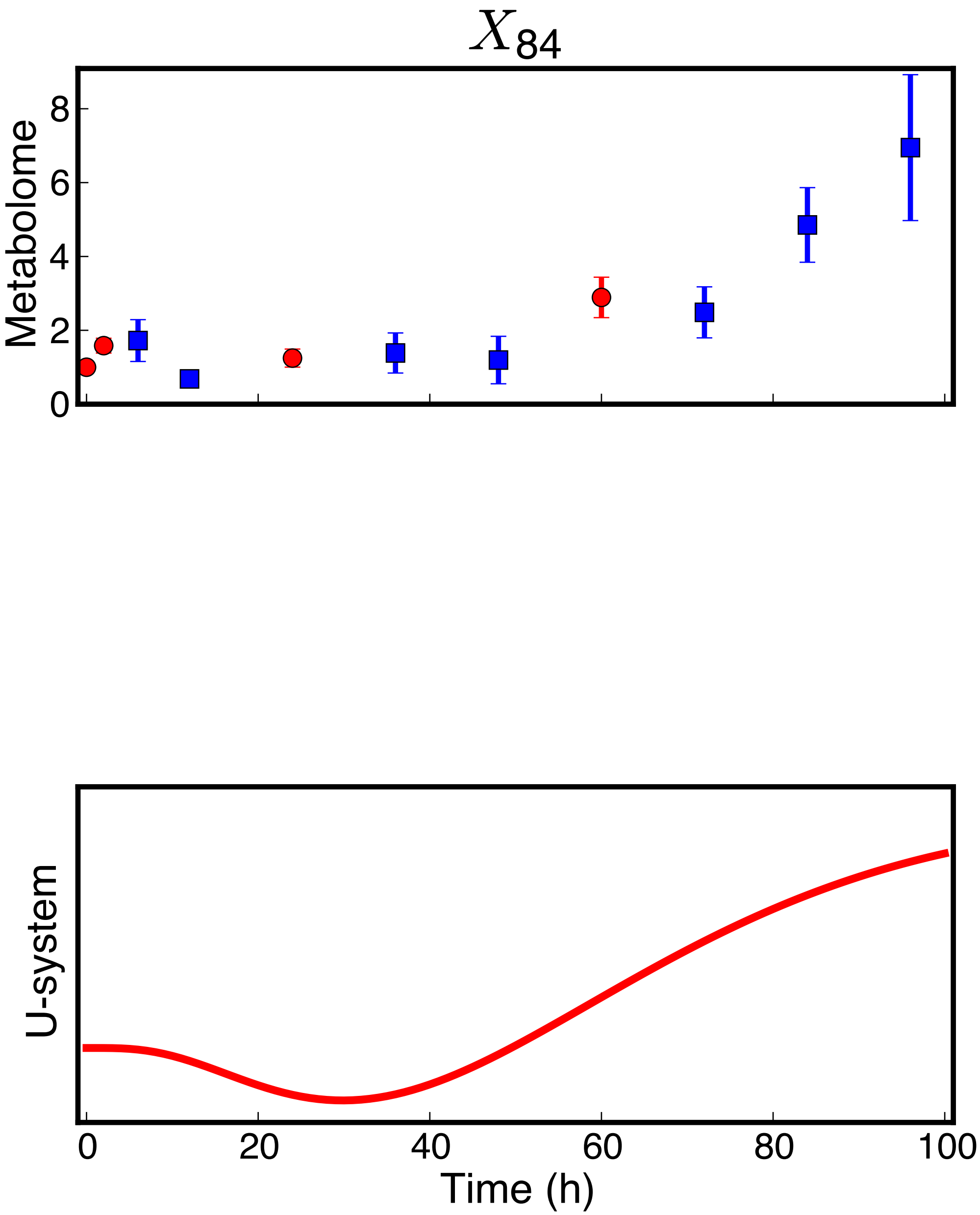

X_{84}
Metabolome
0
2
4
6
8
U-system
0
20
40
60
80
100
Time (h)

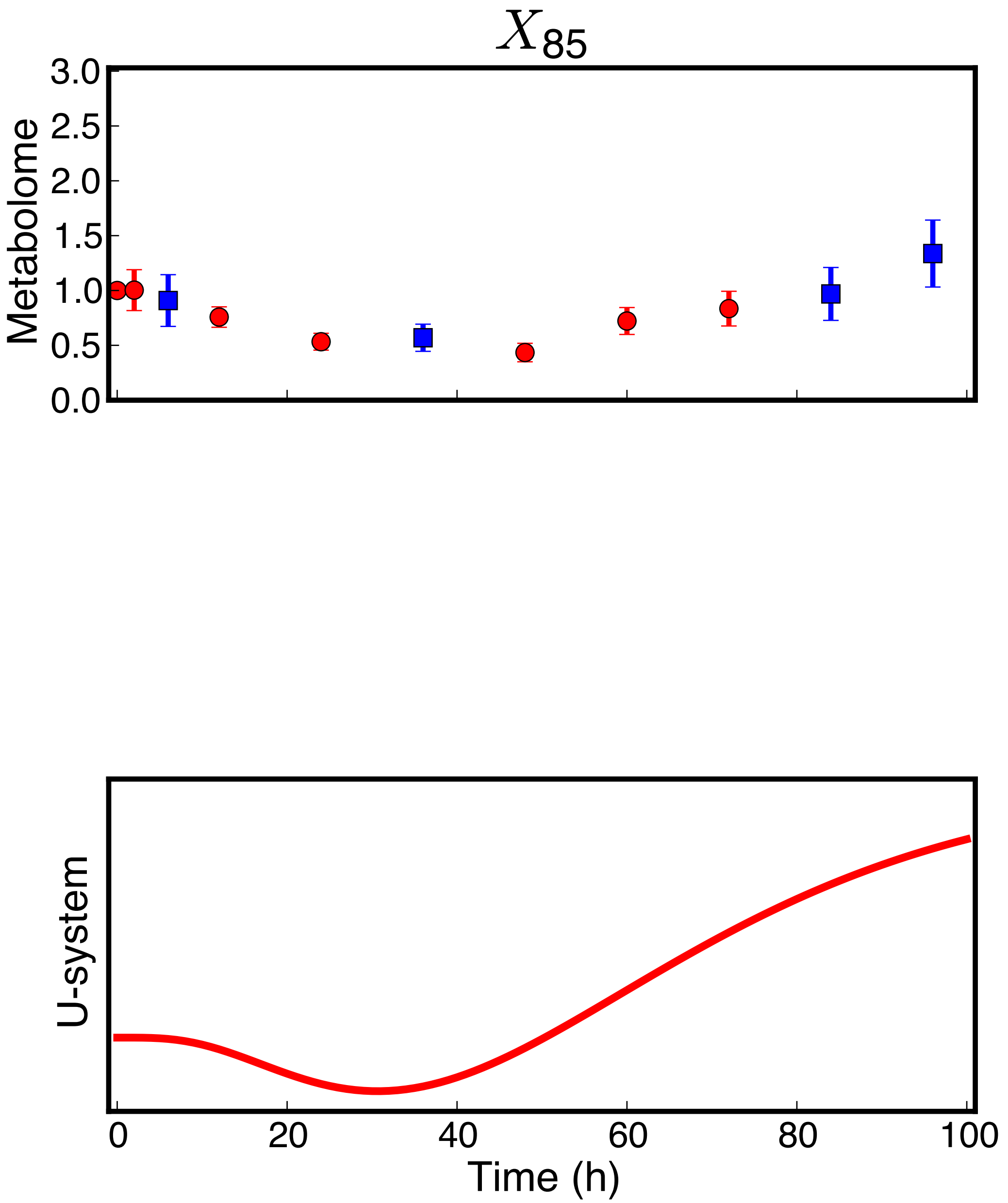

X_{85}
Metabolome
3.0
2.5
2.0
1.5
1.0
0.5
0.0
U-system
0
20
40
60
80
100
Time (h)

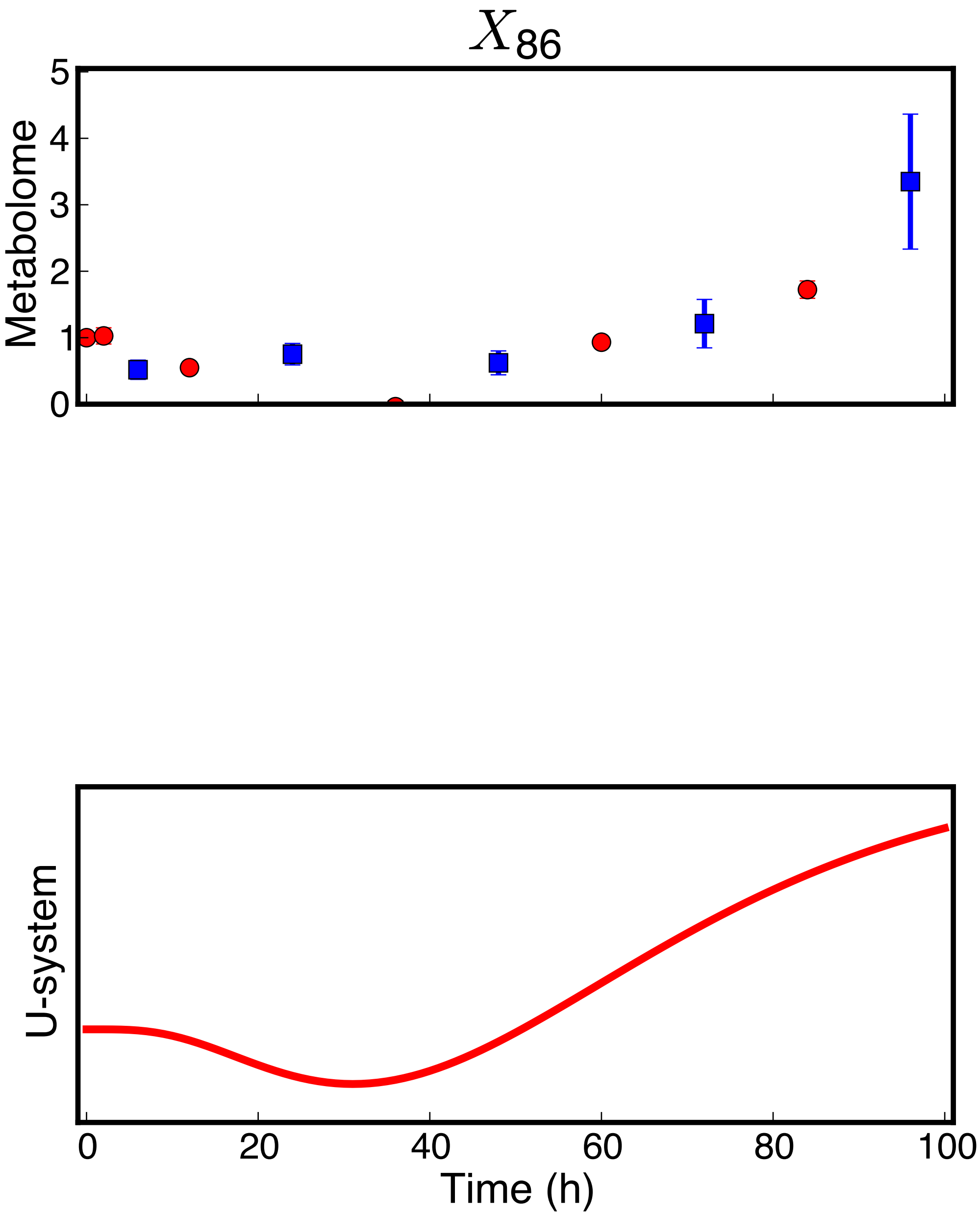
X_{86}
Metabolome
0
1
2
3
4
5
U-system
0
20
40
60
80
100
Time (h)

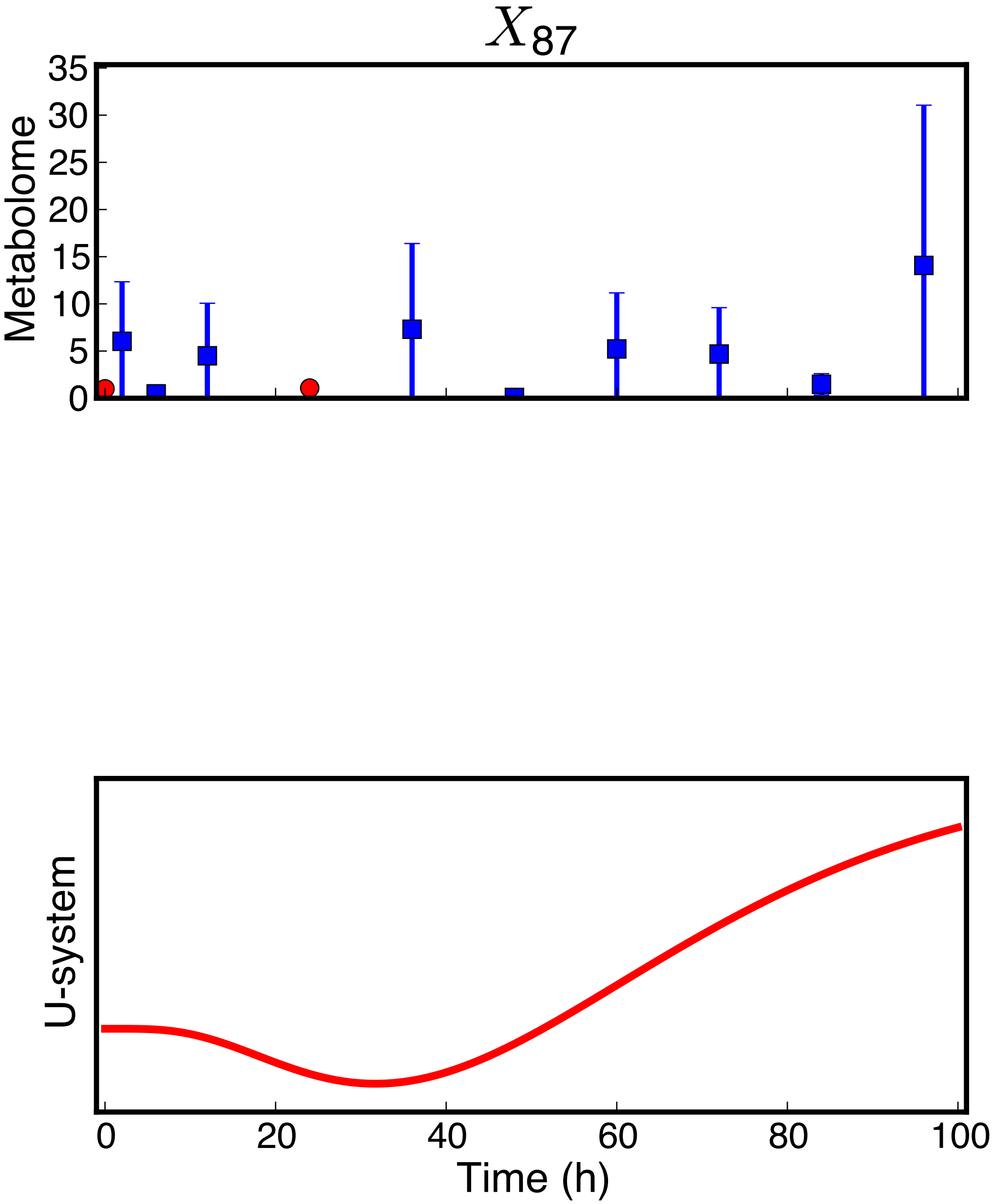

X_{87}
Metabolome
35
30
25
20
15
10
5
0
U-system
0
20
40
60
80
100
Time (h)

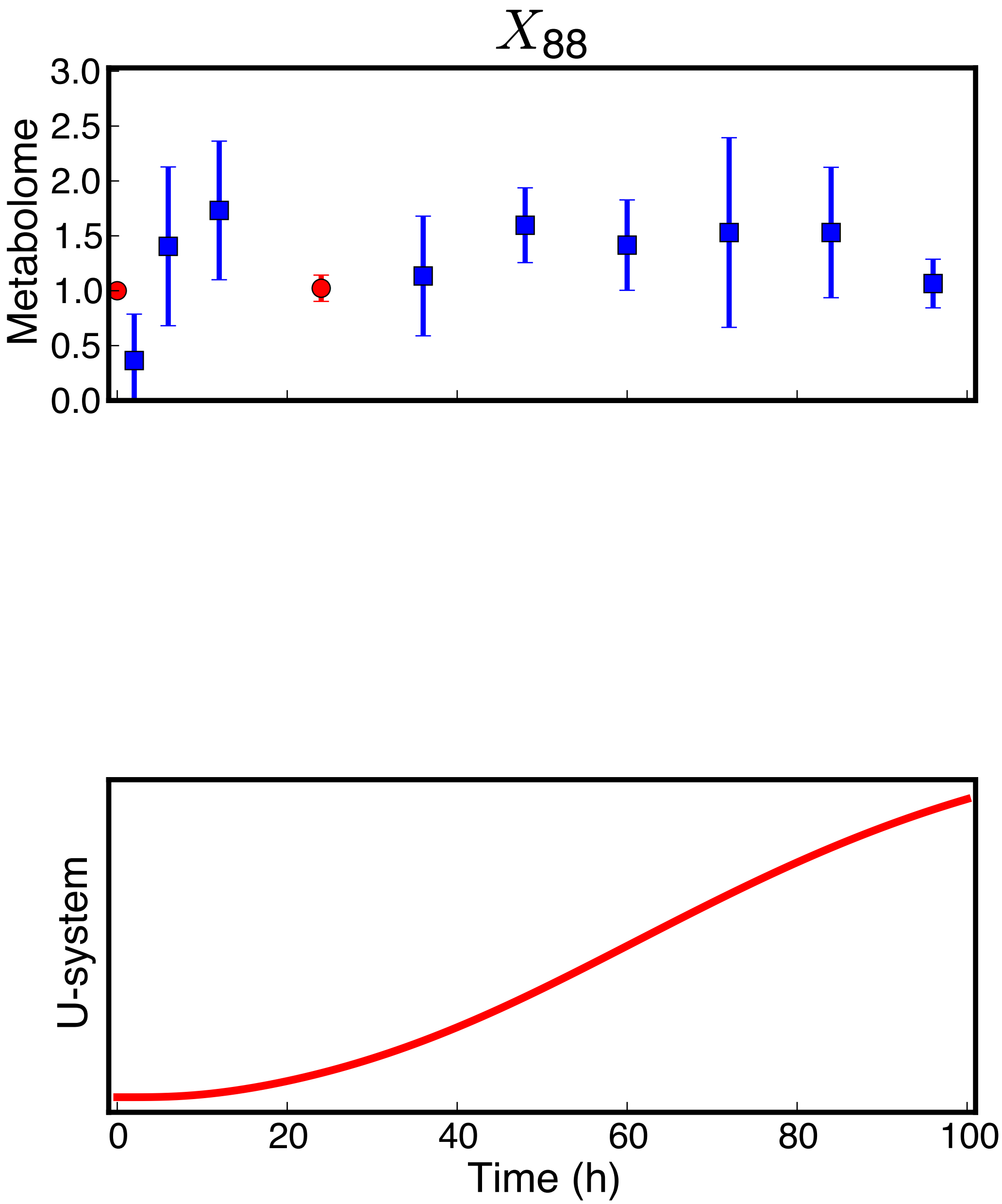

X_{88}
Metabolome
3.0
2.5
2.0
1.5
1.0
0.5
0.0
U-system
0
20
40
60
80
100
Time (h)

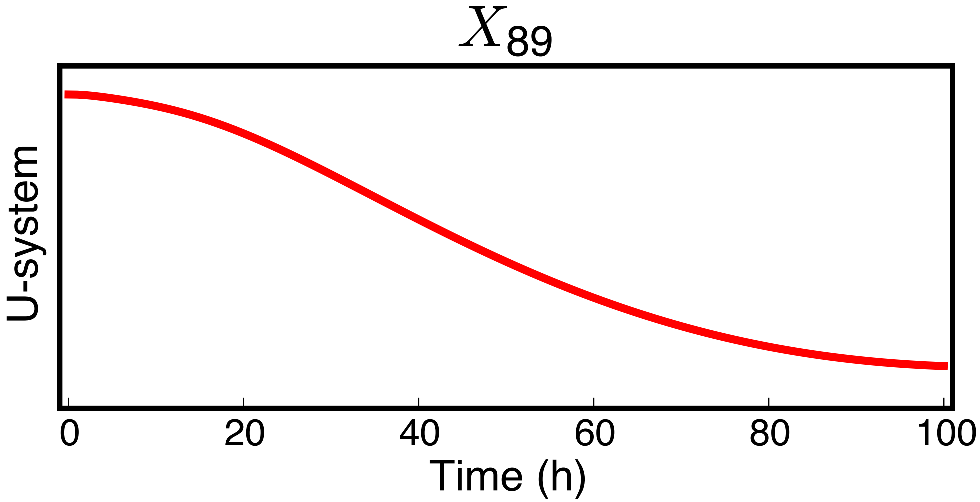

X_{89}
U-system
0
20
40
60
80
100
Time (h)

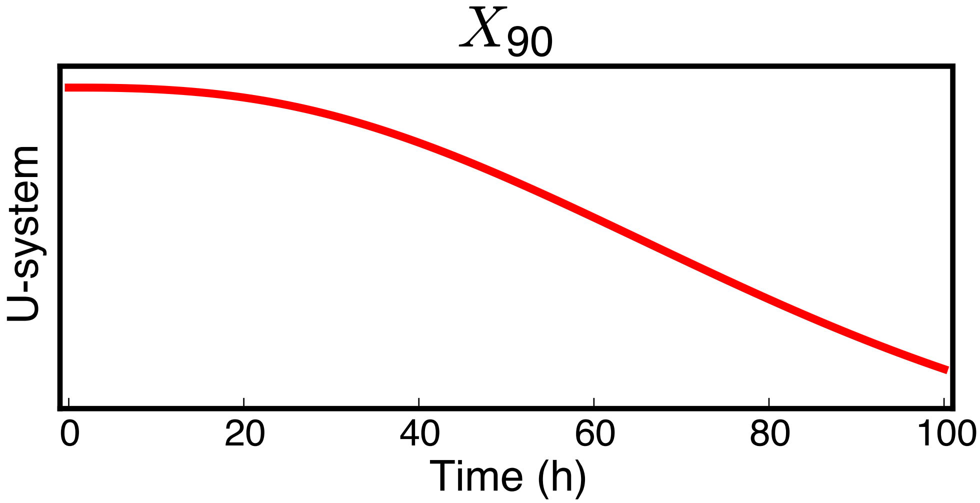

X_{90}
U-system
0
20
40
60
80
100
Time (h)

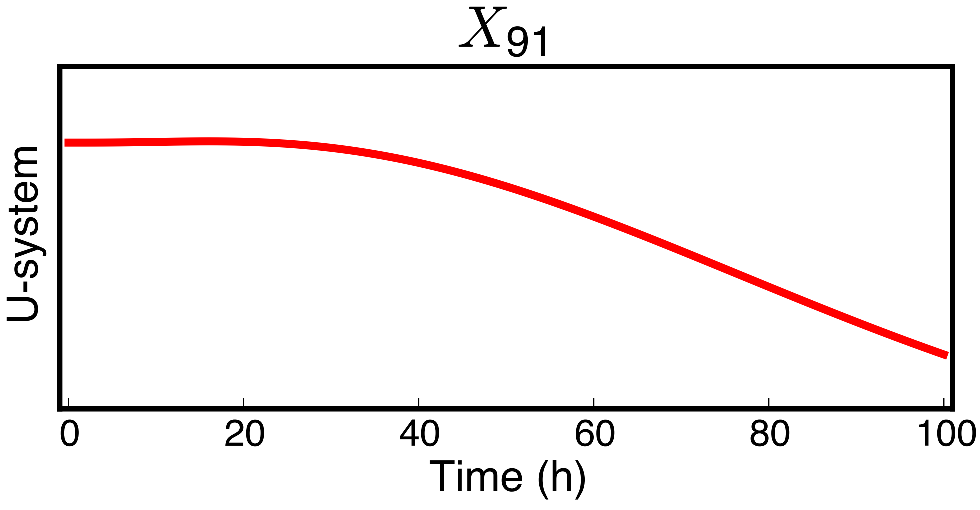

X_{91}
U-system
0
20
40
60
80
100
Time (h)

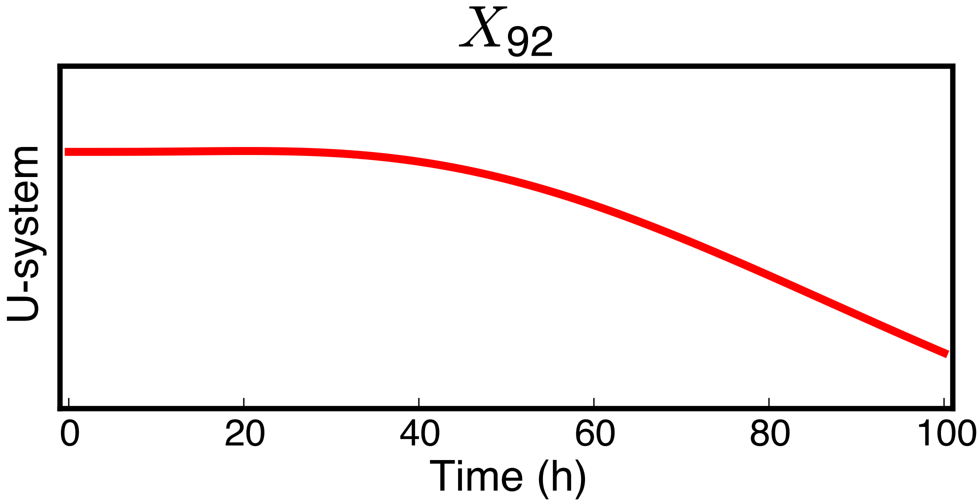

X_{92}
U-system
0
20
40
60
80
100
Time (h)

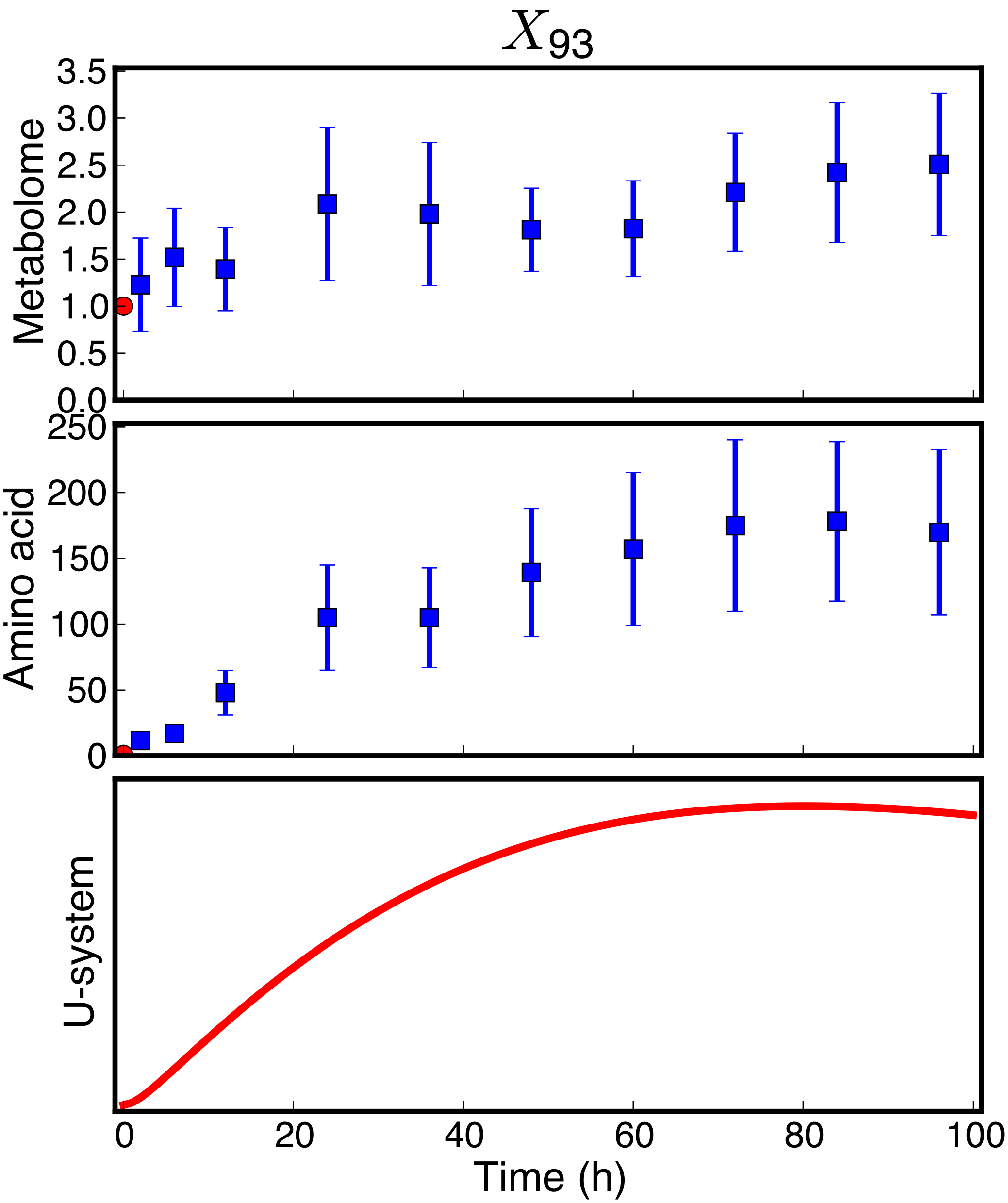

X_{93}
Metabolome
3.5
3.0
2.5
2.0
1.5
1.0
0.5
0.0
Amino acid
250
200
150
100
50
0
U-system
0
20
40
60
80
100
Time (h)

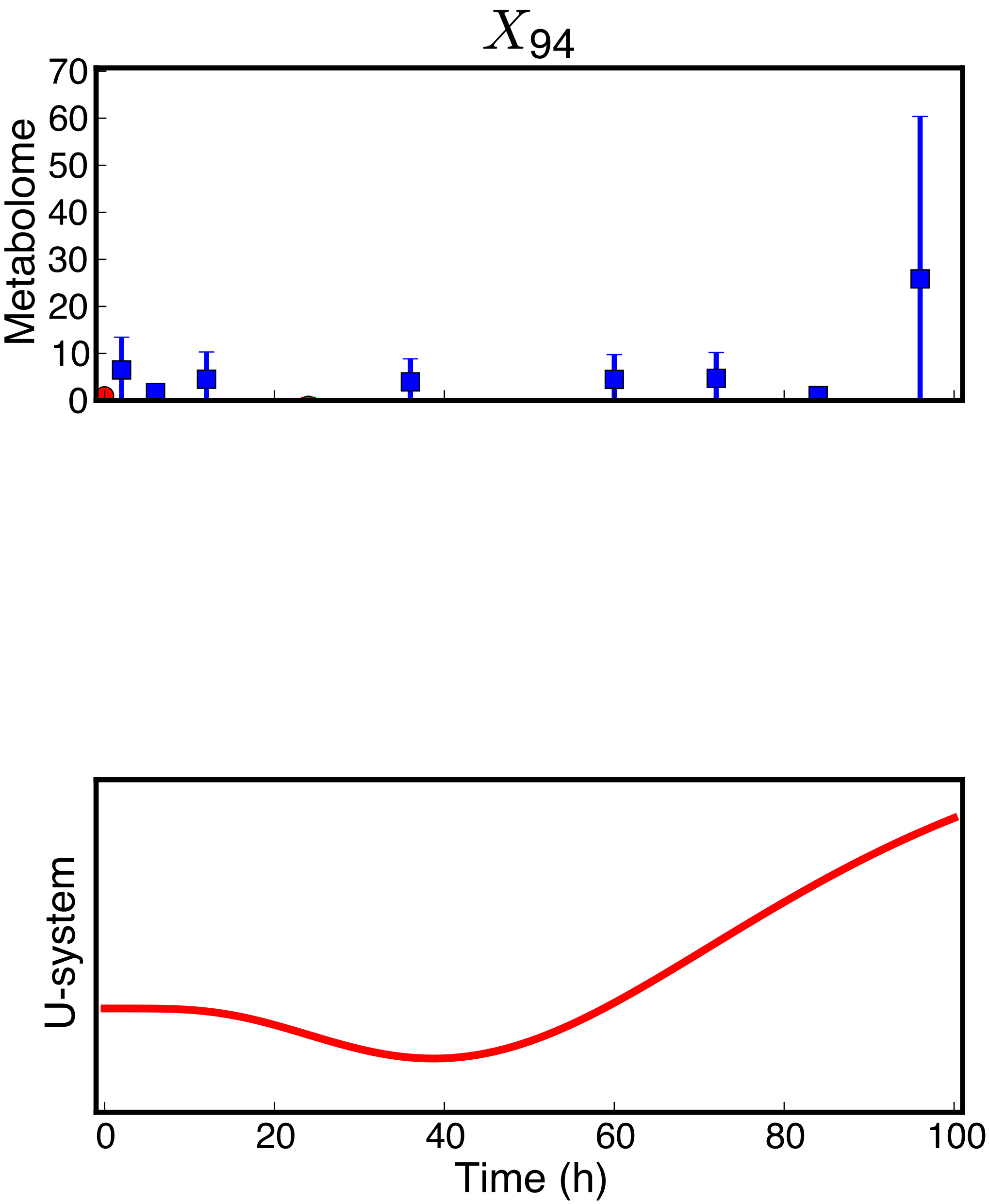

X_{94}
Metabolome
70
60
50
40
30
20
10
0
U-system
0
20
40
60
80
100
Time (h)

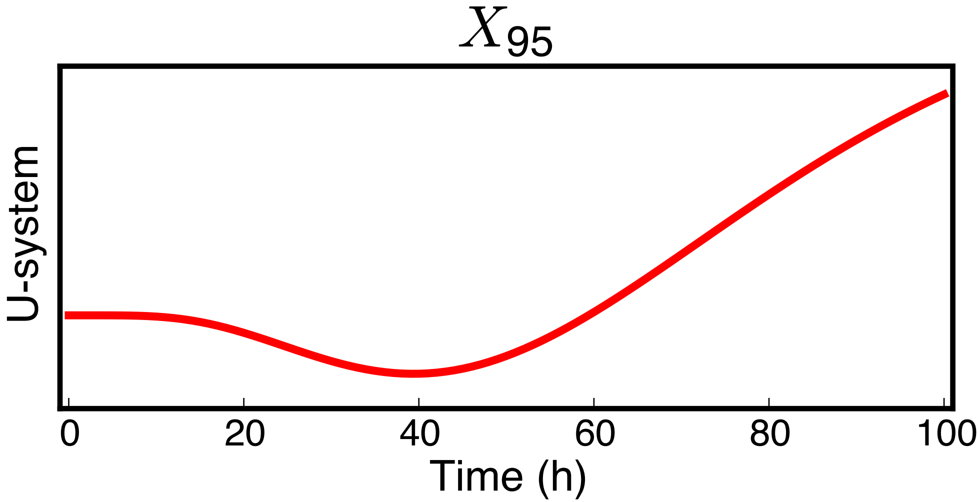
X_{95}
U-system
0
20
40
60
80
100
Time (h)

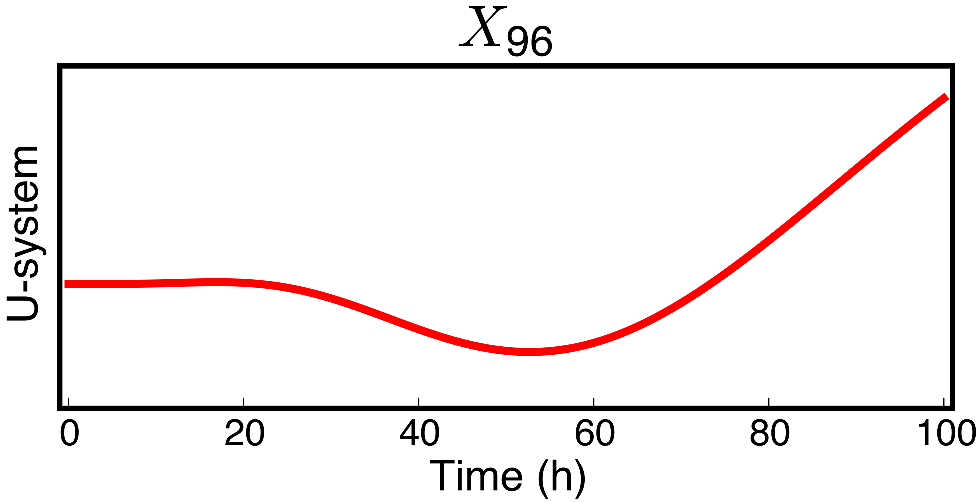

X_{96}
U-system
0
20
40
60
80
100
Time (h)

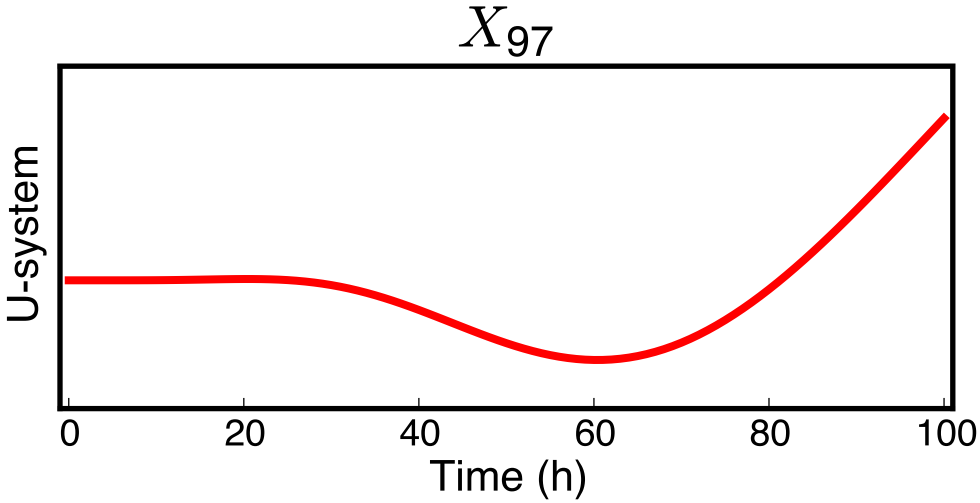
X_{97}
U-system
0
20
40
60
80
100
Time (h)

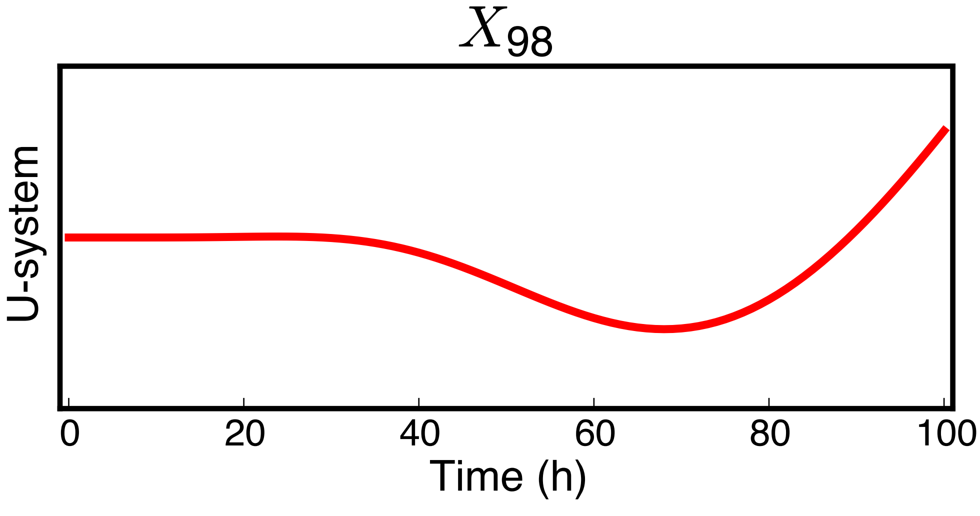

X_{98}
U-system
0
20
40
60
80
100
Time (h)

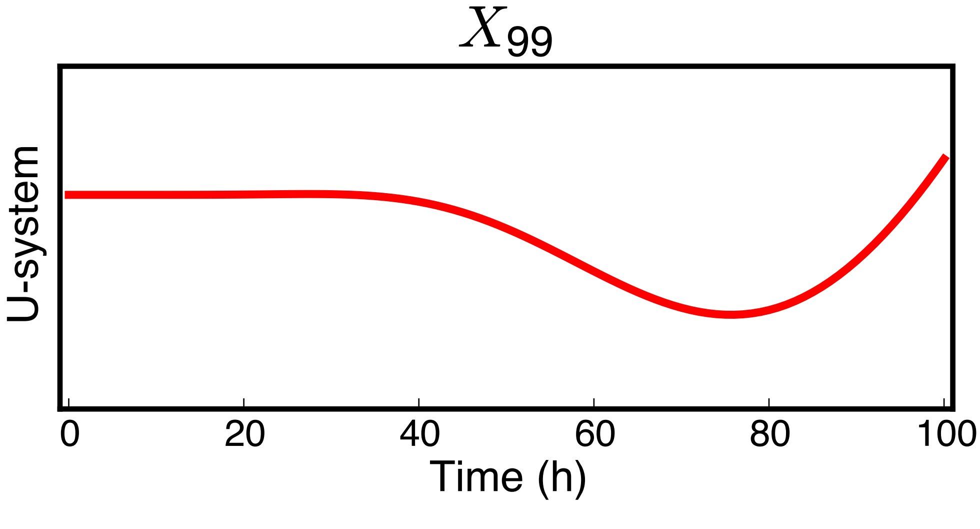

X_{99}
U-system
0
20
40
60
80
100
Time (h)

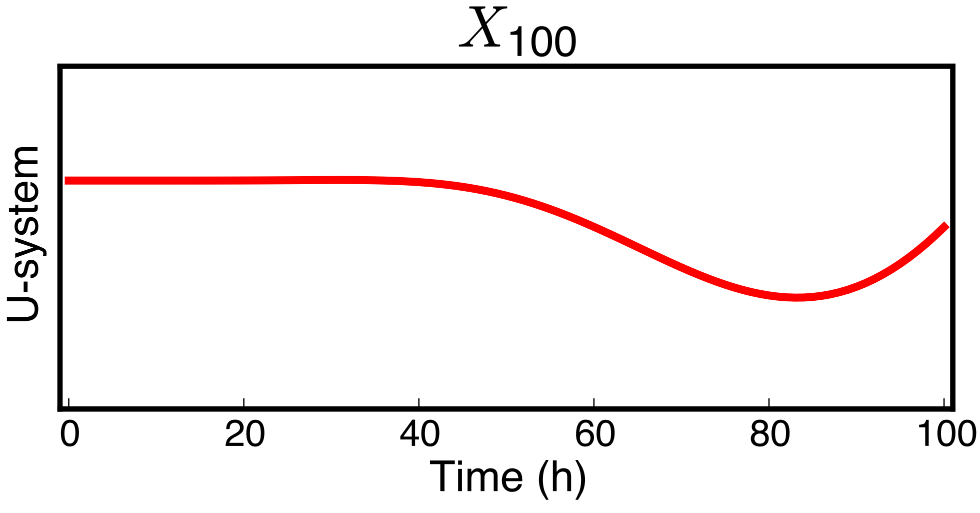

X_{100}
U-system
0
20
40
60
80
100
Time (h)

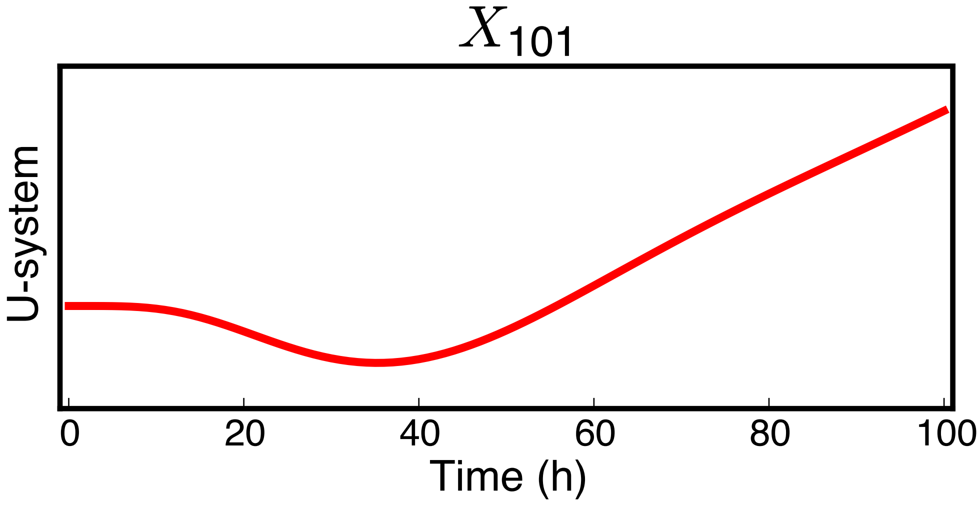

X_{101}
U-system
0
20
40
60
80
100
Time (h)

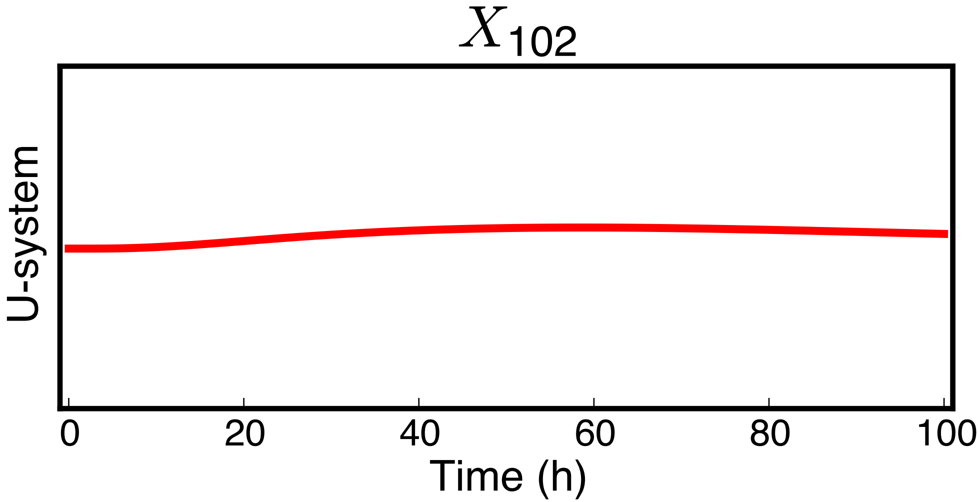

X_{102}
U-system
0
20
40
60
80
100
Time (h)

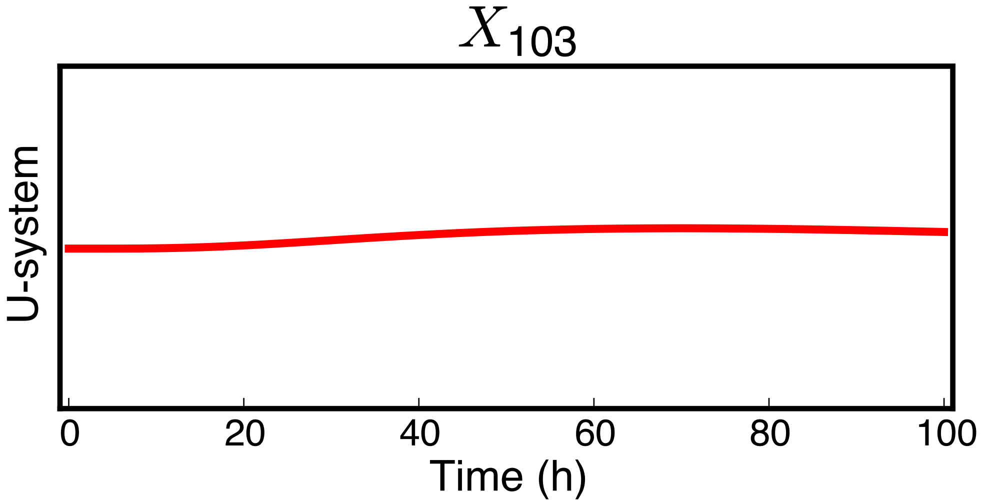

X_{103}
U-system
0
20
40
60
80
100
Time (h)

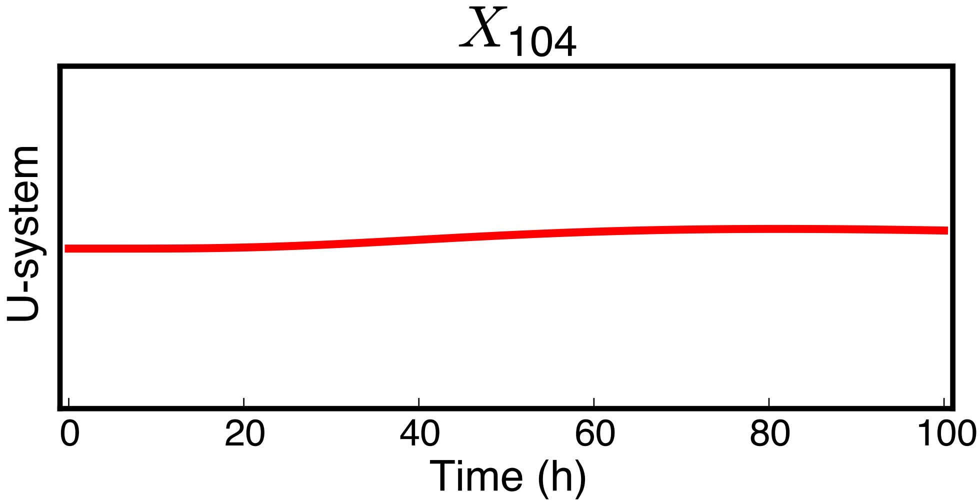

X_{104}
U-system
0
20
40
60
80
100
Time (h)

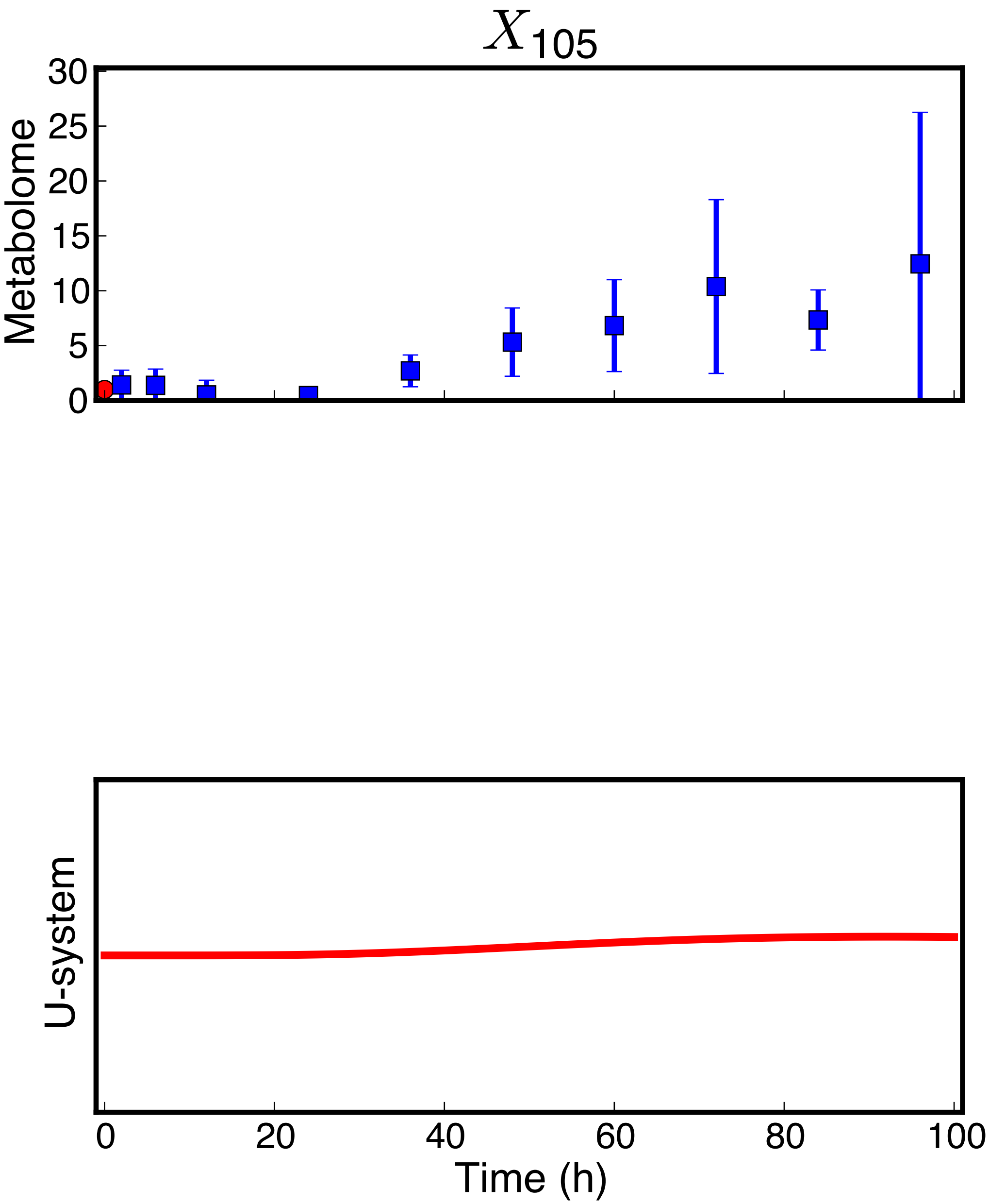

X_{105}
Metabolome
0
5
10
15
20
25
30
U-system
0
20
40
60
80
100
Time (h)

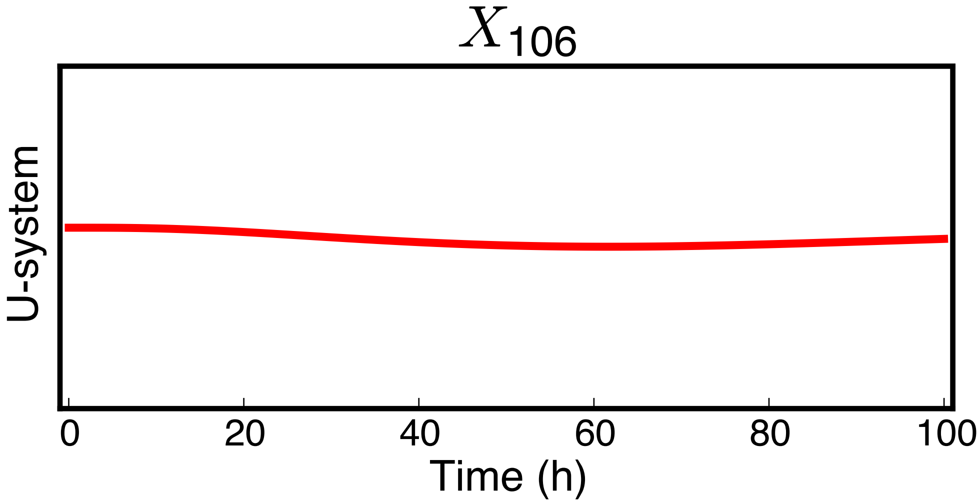

X_{106}
U-system
0
20
40
60
80
100
Time (h)

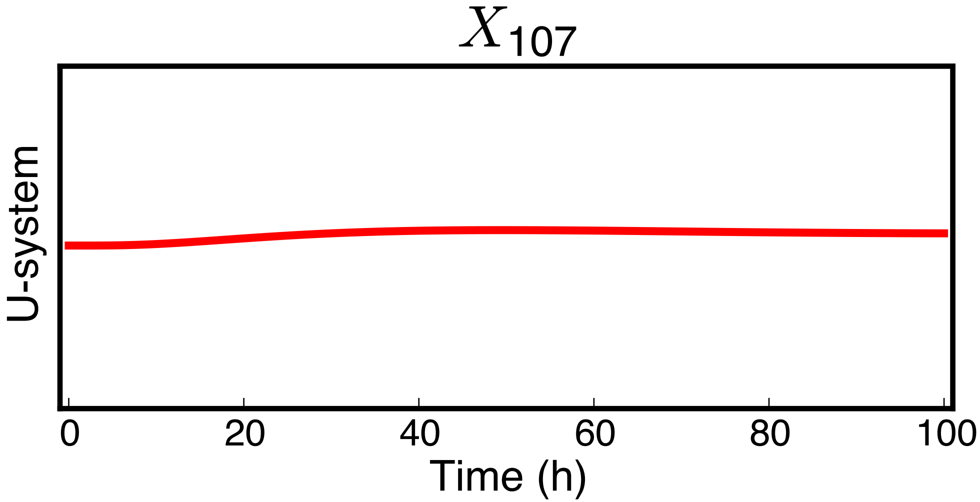

X_{107}
U-system
0
20
40
60
80
100
Time (h)

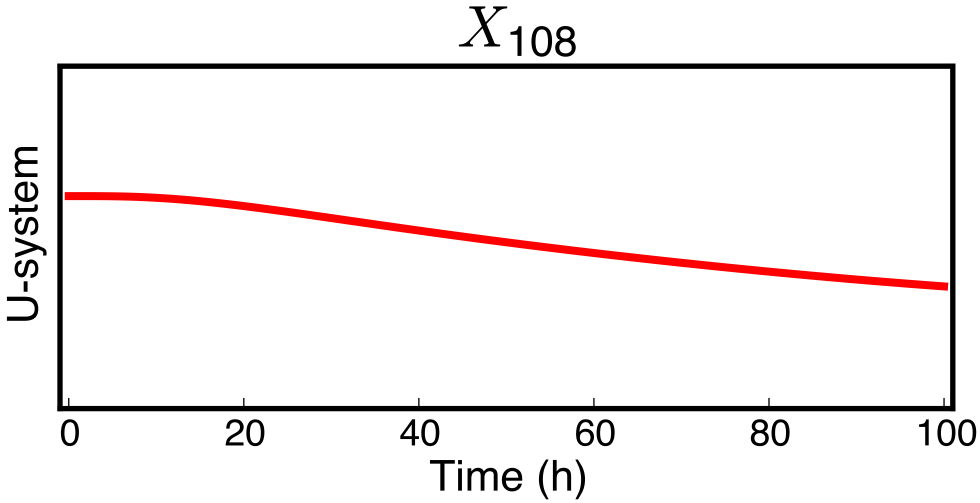

X_{108}
U-system
0
20
40
60
80
100
Time (h)

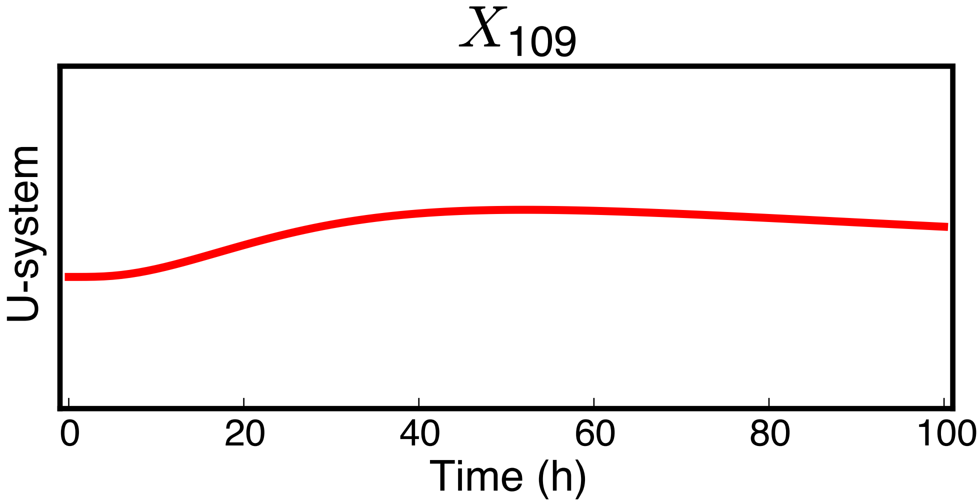
X_{109}
U-system
0
20
40
60
80
100
Time (h)

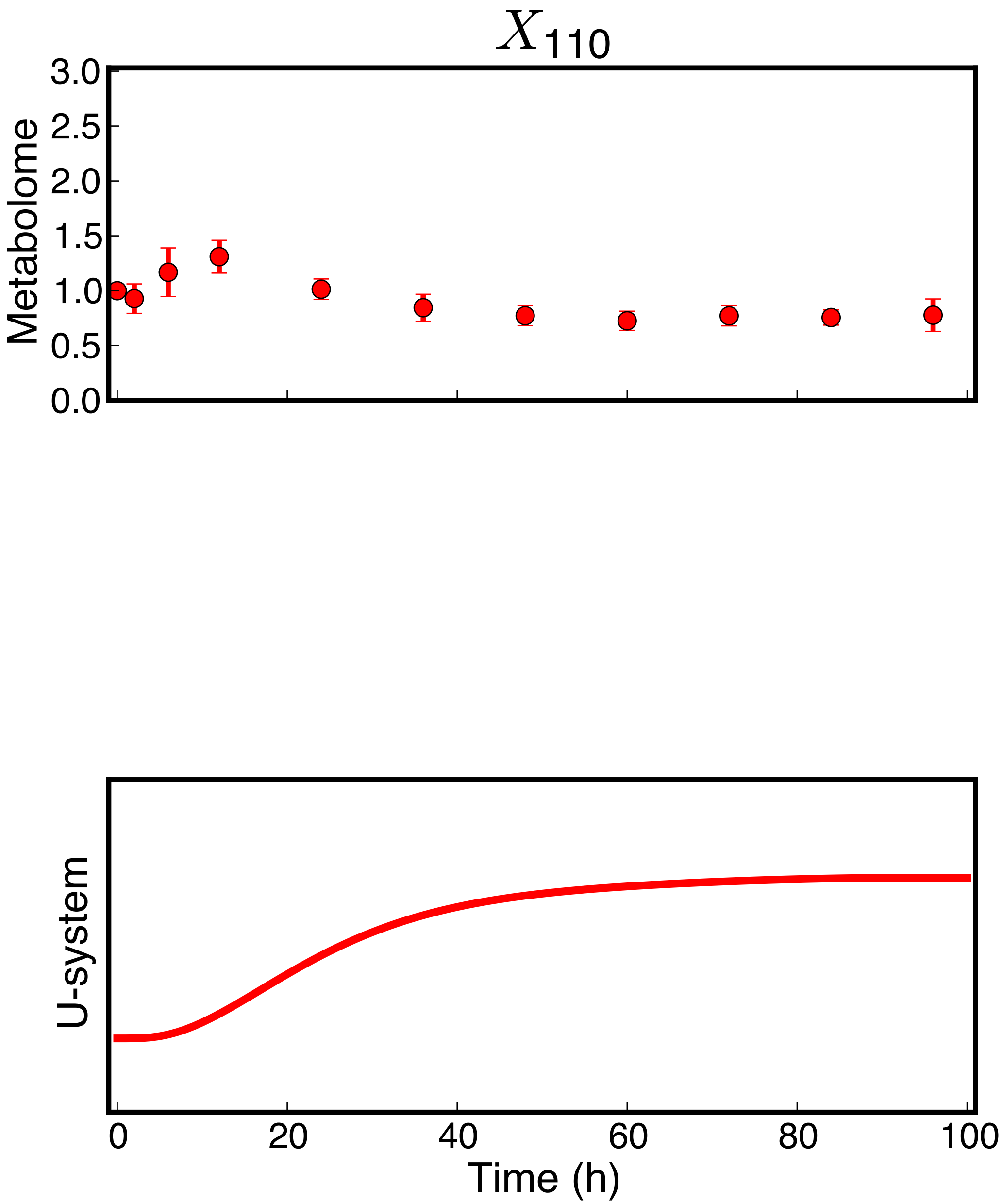

X_{110}
Metabolome
3.0
2.5
2.0
1.5
1.0
0.5
0.0
U-system
0
20
40
60
80
100
Time (h)

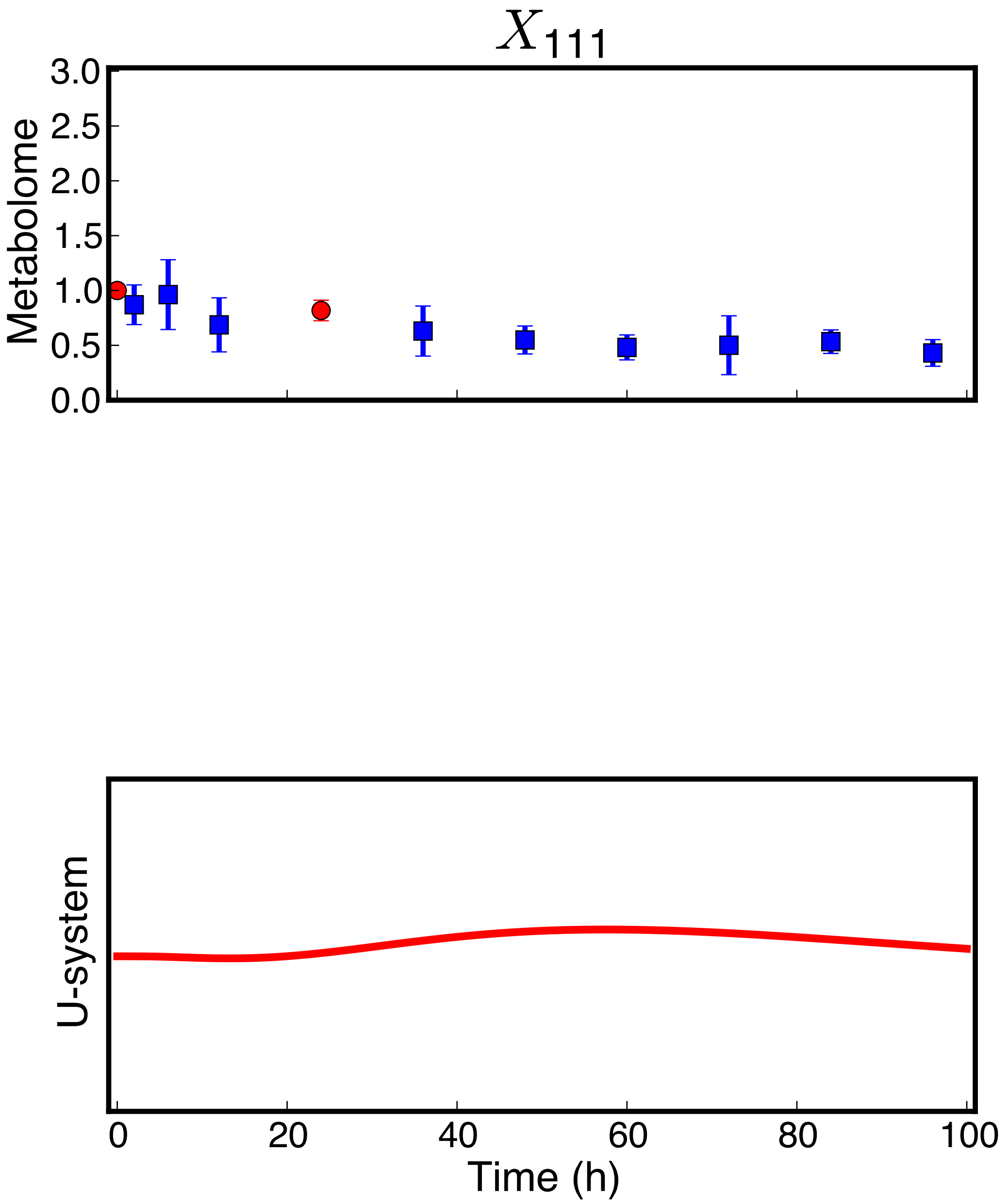

X_{111}
Metabolome
3.0
2.5
2.0
1.5
1.0
0.5
0.0
U-system
0
20
40
60
80
100
Time (h)

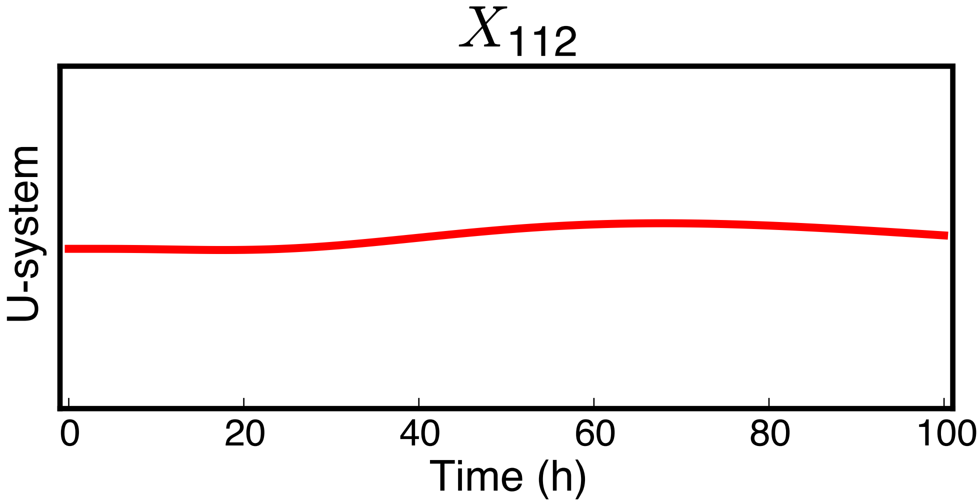
X_{112}
U-system
0
20
40
60
80
100
Time (h)

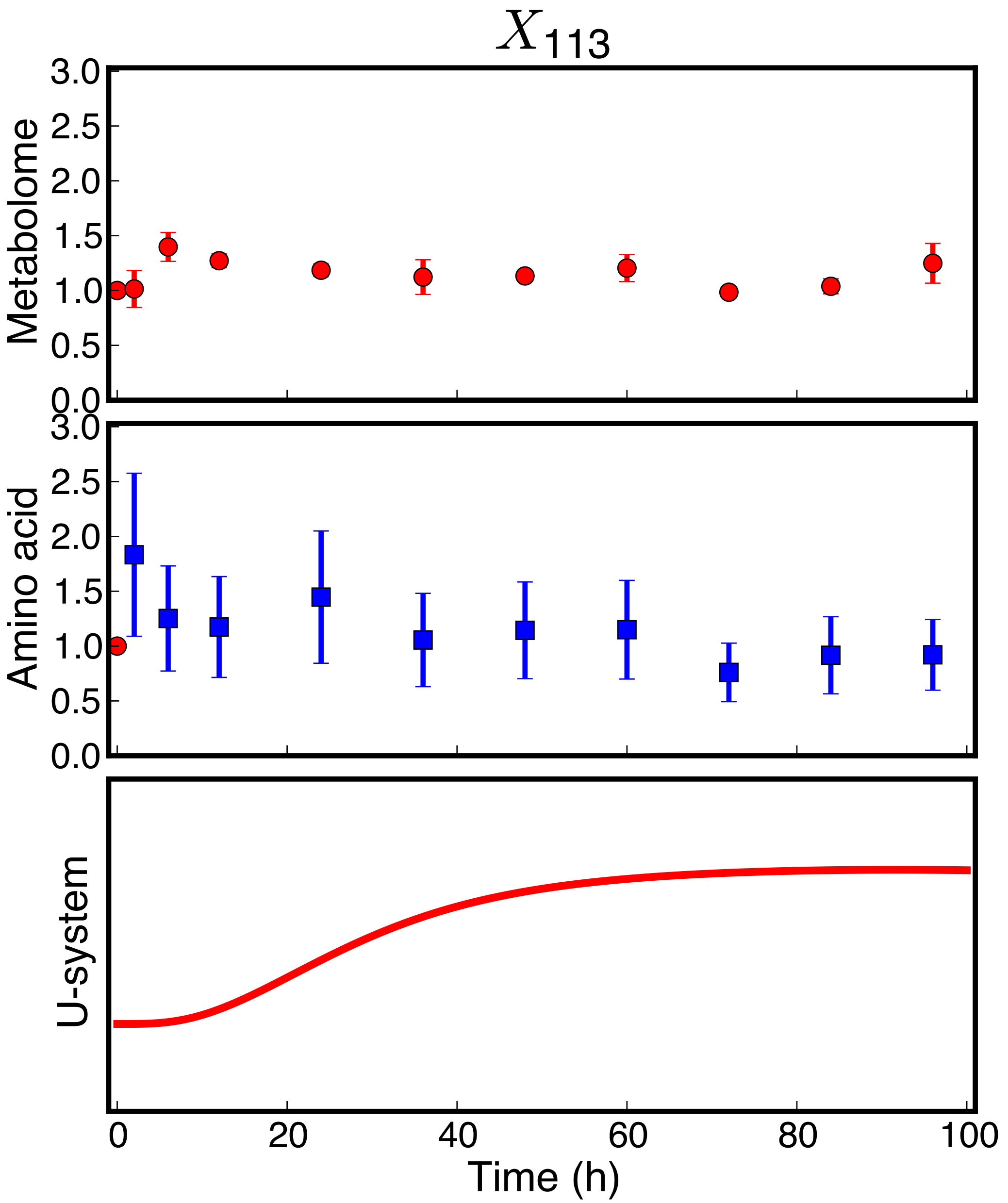
X_{113}
Metabolome
3.0
2.5
2.0
1.5
1.0
0.5
0.0
Amino acid
3.0
2.5
2.0
1.5
1.0
0.5
0.0
U-system
0
20
40
60
80
100
Time (h)

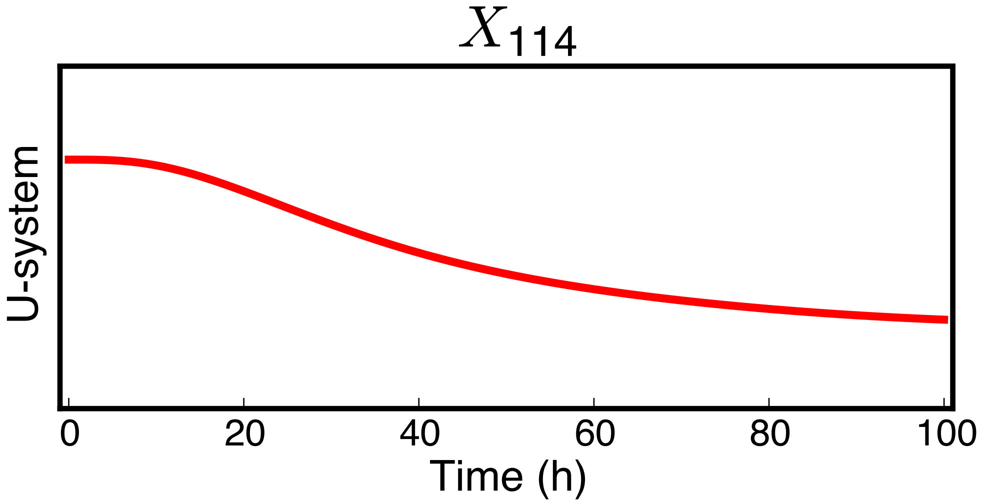

X_{114}
U-system
0
20
40
60
80
100
Time (h)

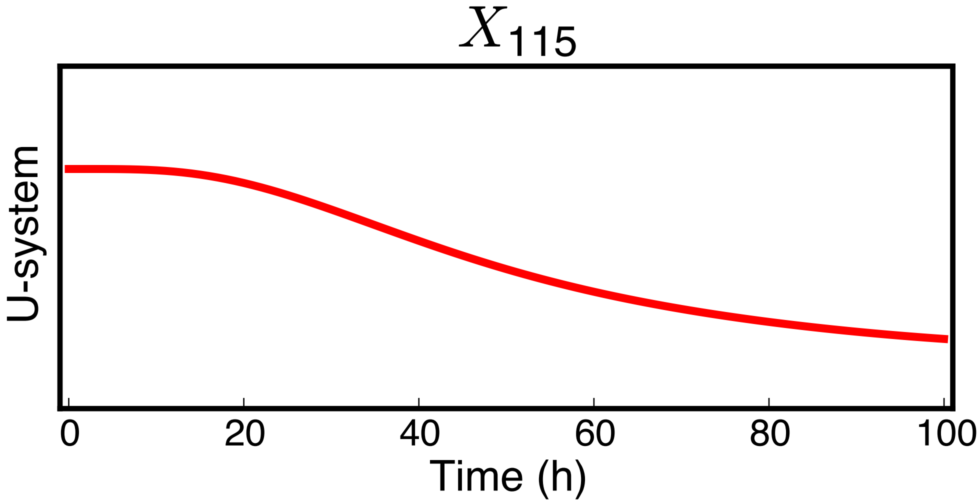
X_{115}
U-system
0
20
40
60
80
100
Time (h)

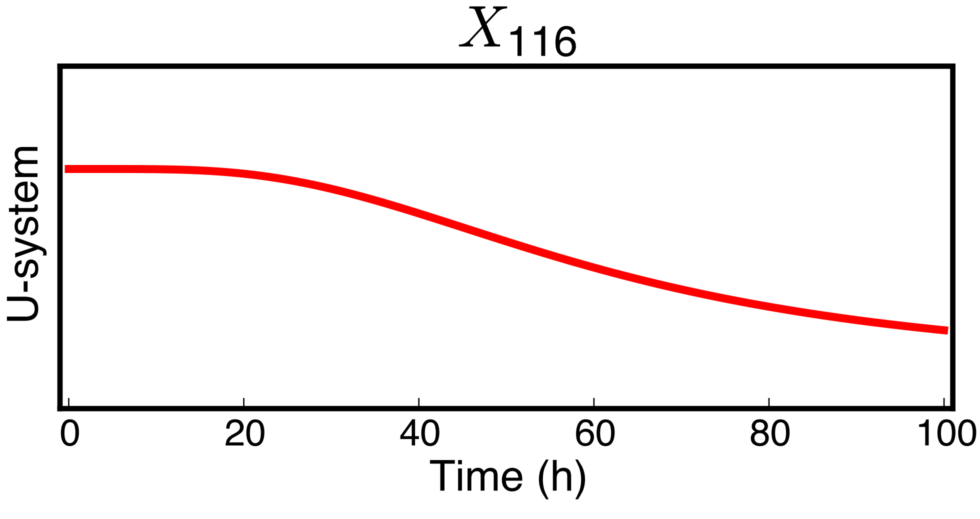

X_{116}
U-system
0
20
40
60
80
100
Time (h)

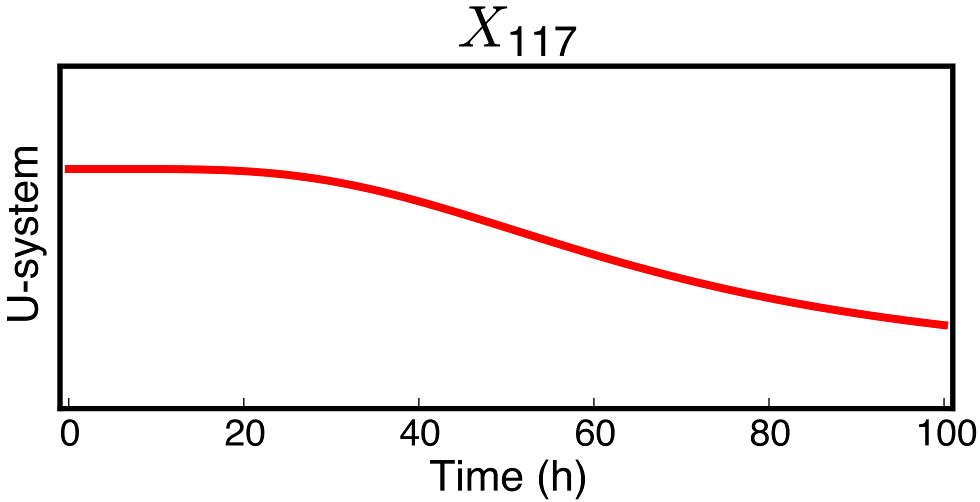

X_{117}
U-system
0
20
40
60
80
100
Time (h)

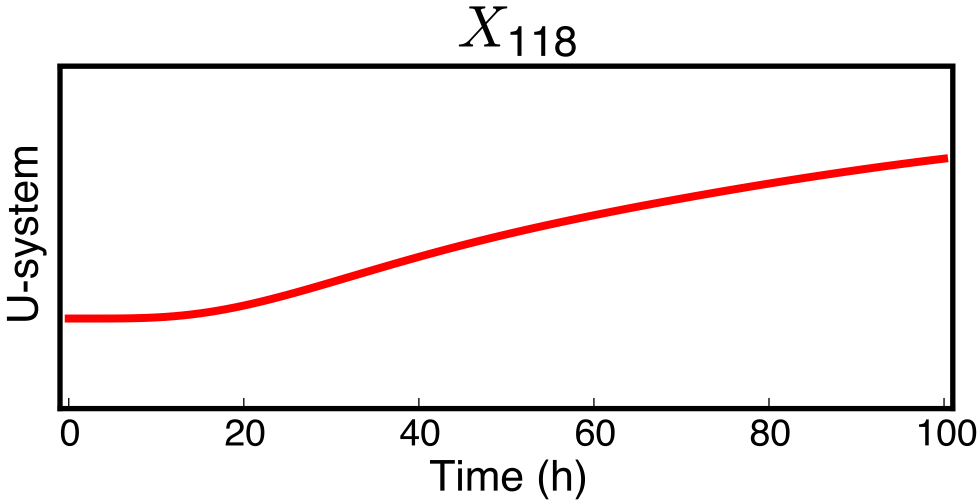
X_{118}
U-system
0
20
40
60
80
100
Time (h)

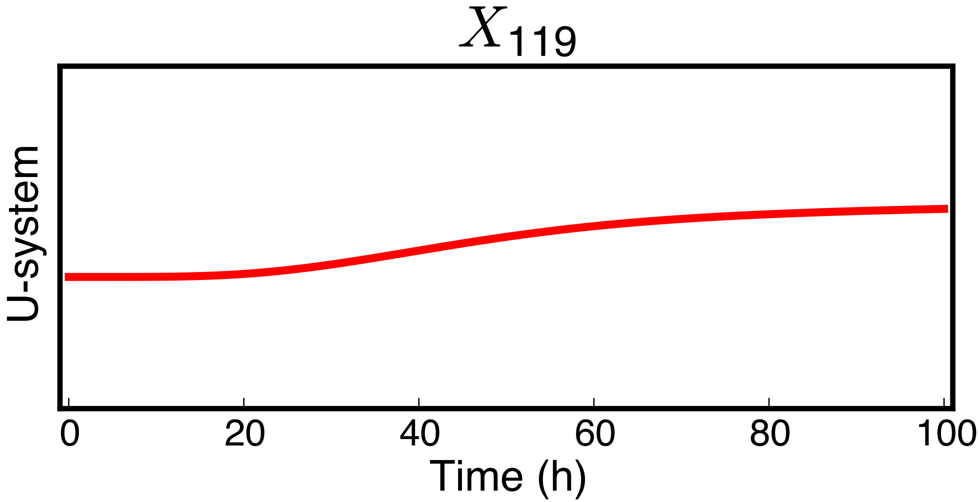
X_{119}
U-system
0
20
40
60
80
100
Time (h)

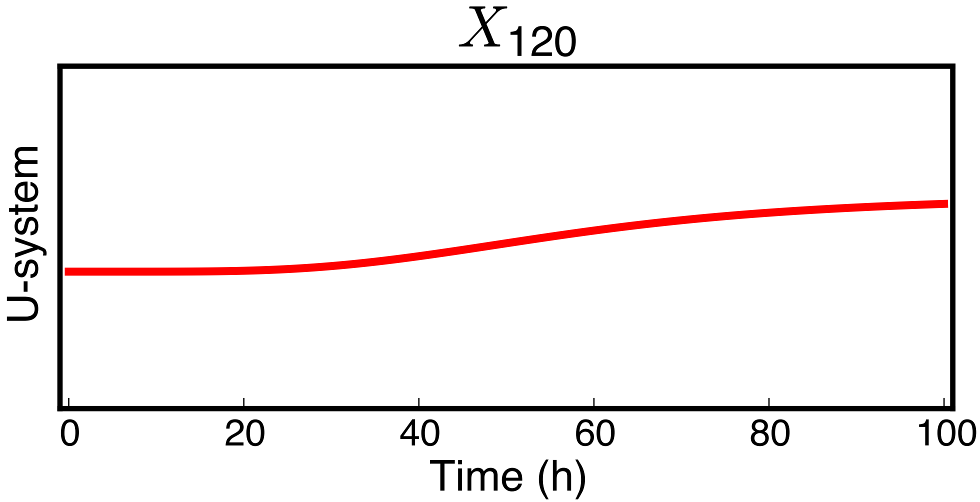

X_{120}
U-system
0
20
40
60
80
100
Time (h)

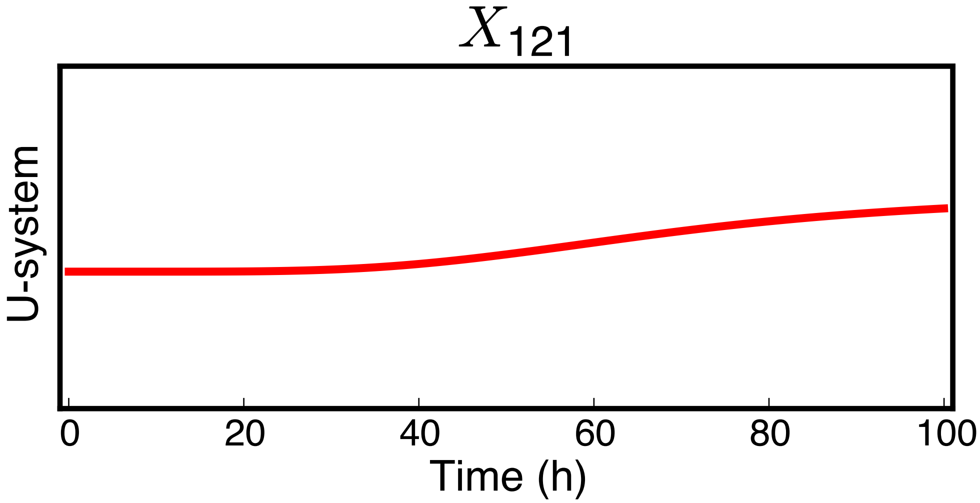
X_{121}
U-system
0
20
40
60
80
100
Time (h)

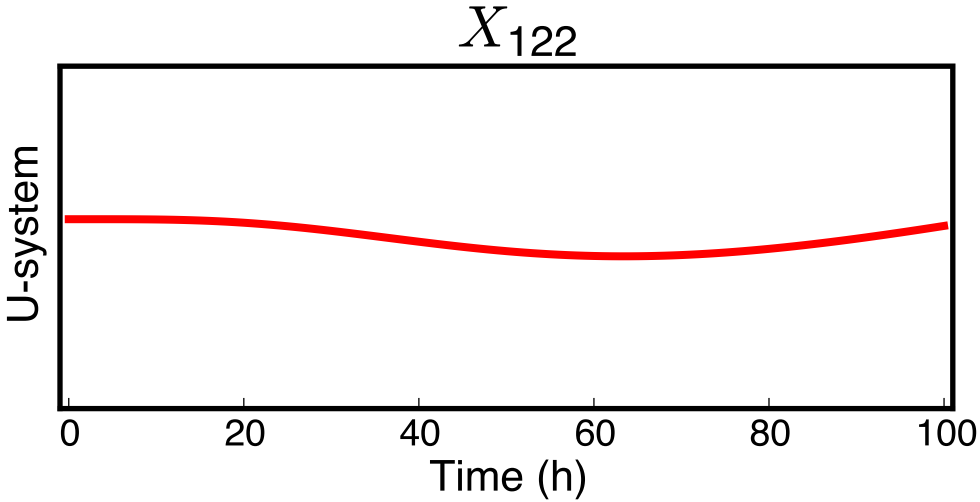

X_{122}
U-system
0
20
40
60
80
100
Time (h)

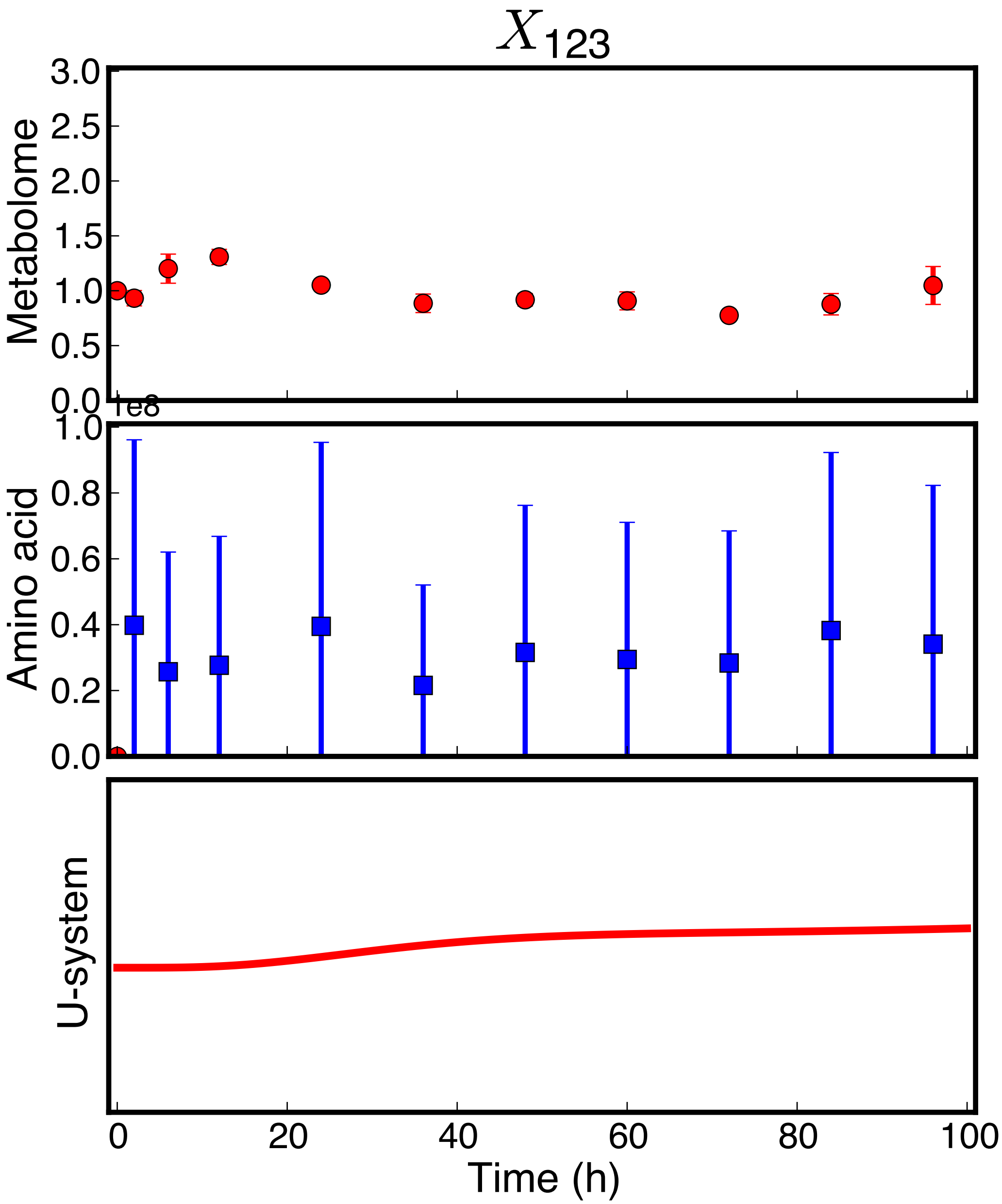

X_{123}
Metabolome
3.0
2.5
2.0
1.5
1.0
0.5
0.0
1e8
Amino acid
1.0
0.8
0.6
0.4
0.2
0.0
U-system
0
20
40
60
80
100
Time (h)

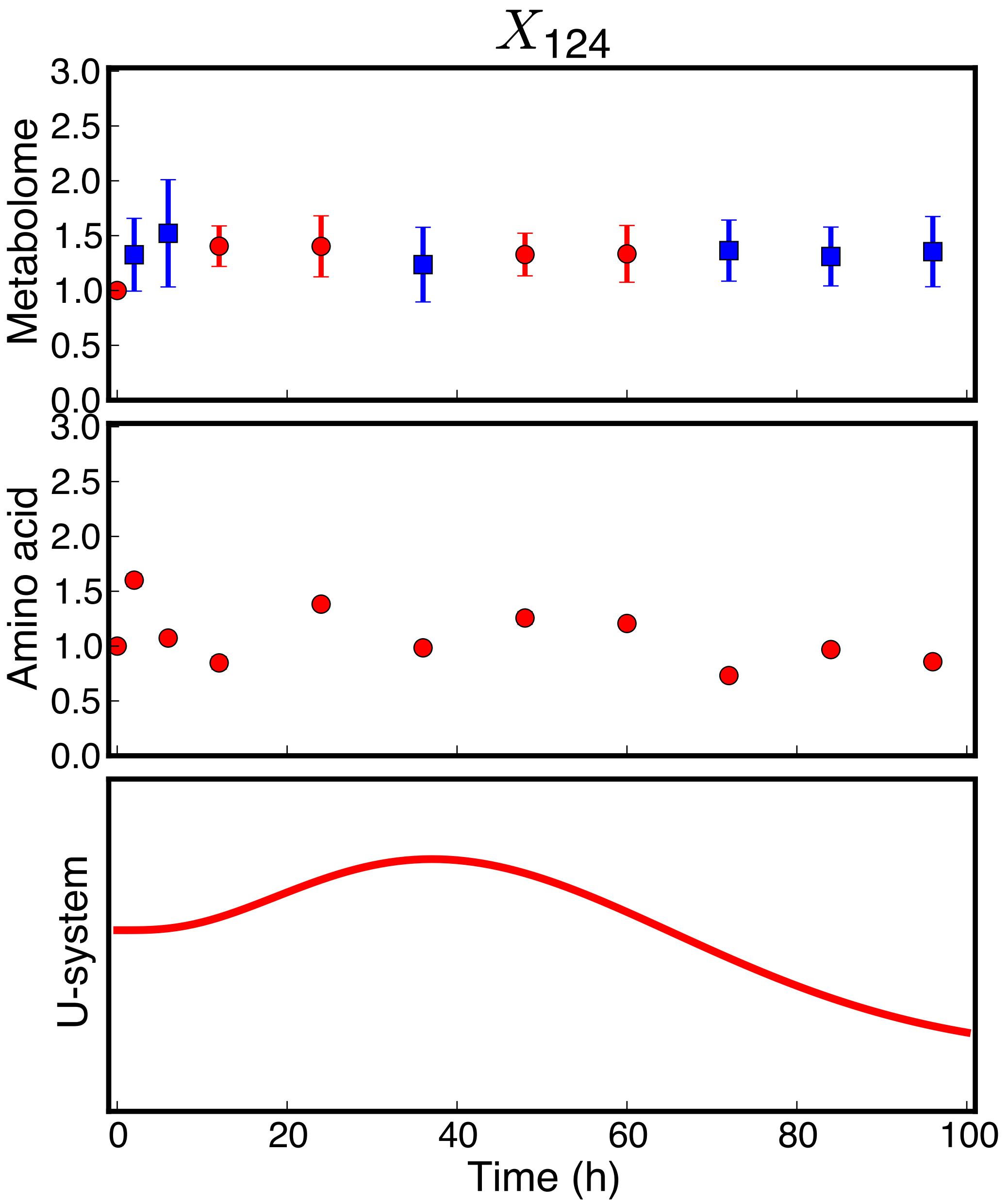

X_{124}
Metabolome
Amino acid
U-system
3.0
2.5
2.0
1.5
1.0
0.5
0.0
0
20
40
60
80
100
Time (h)

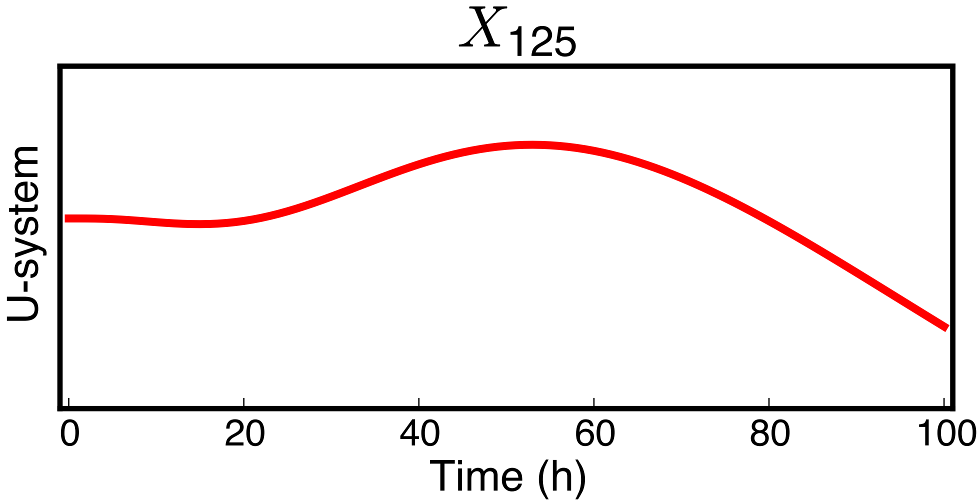

X_{125}
U-system
0
20
40
60
80
100
Time (h)

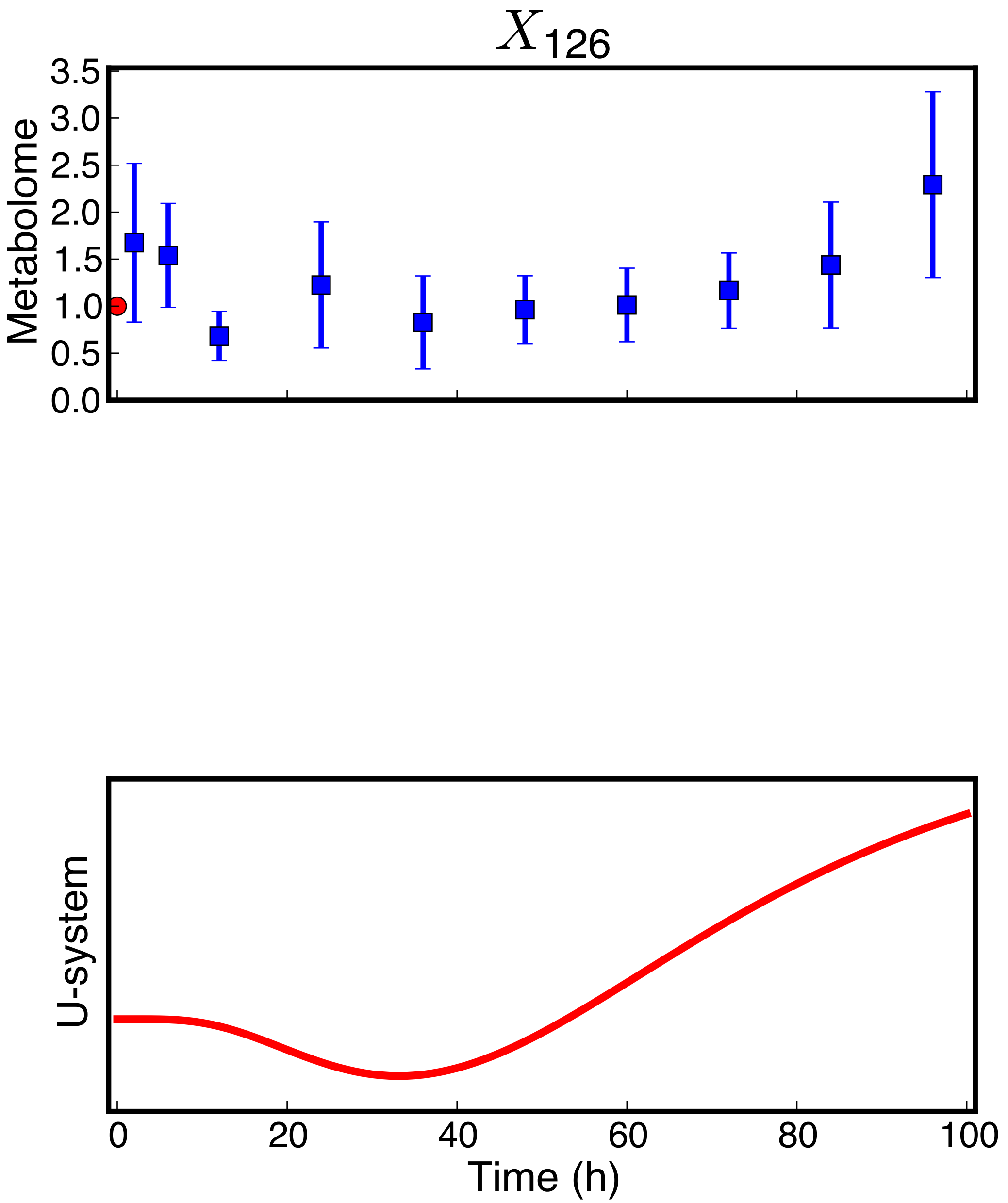

X_{126}
Metabolome
3.5
3.0
2.5
2.0
1.5
1.0
0.5
0.0
U-system
0
20
40
60
80
100
Time (h)

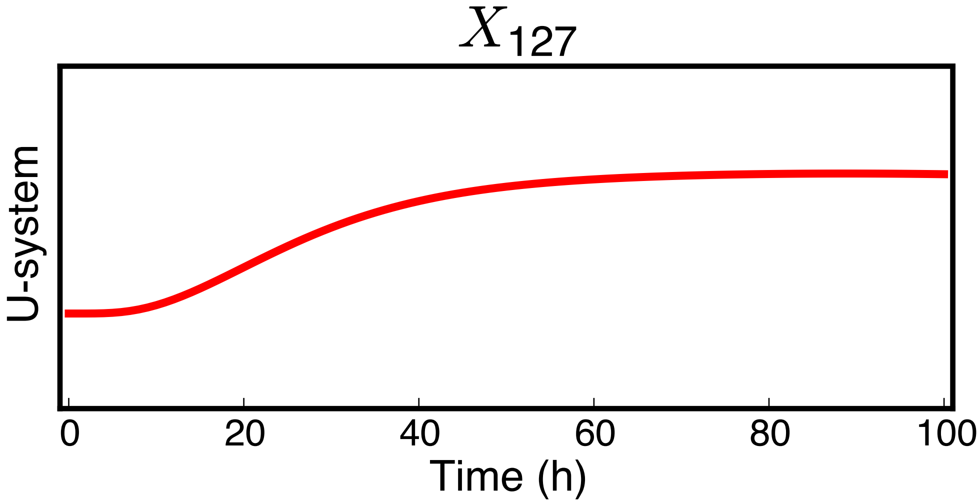

X_{127}
U-system
0
20
40
60
80
100
Time (h)

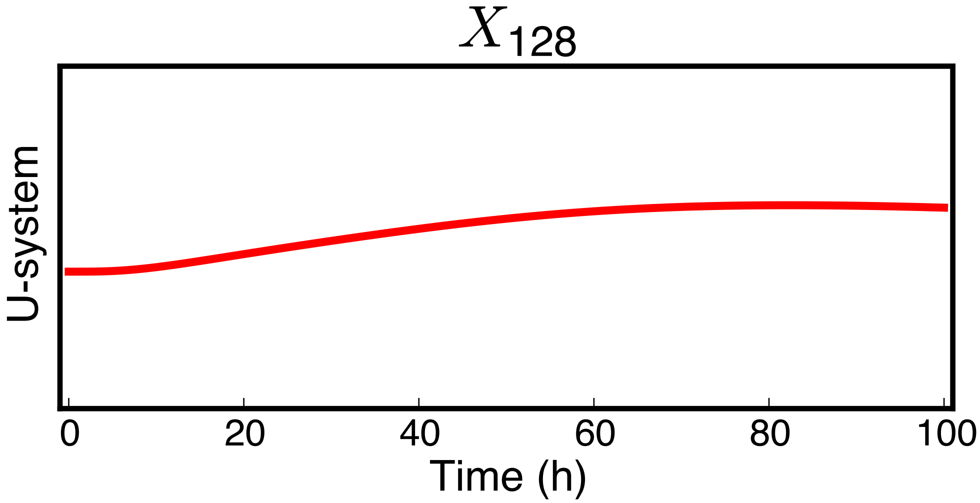

X128
U-system
0
20
40
60
80
100
Time (h)

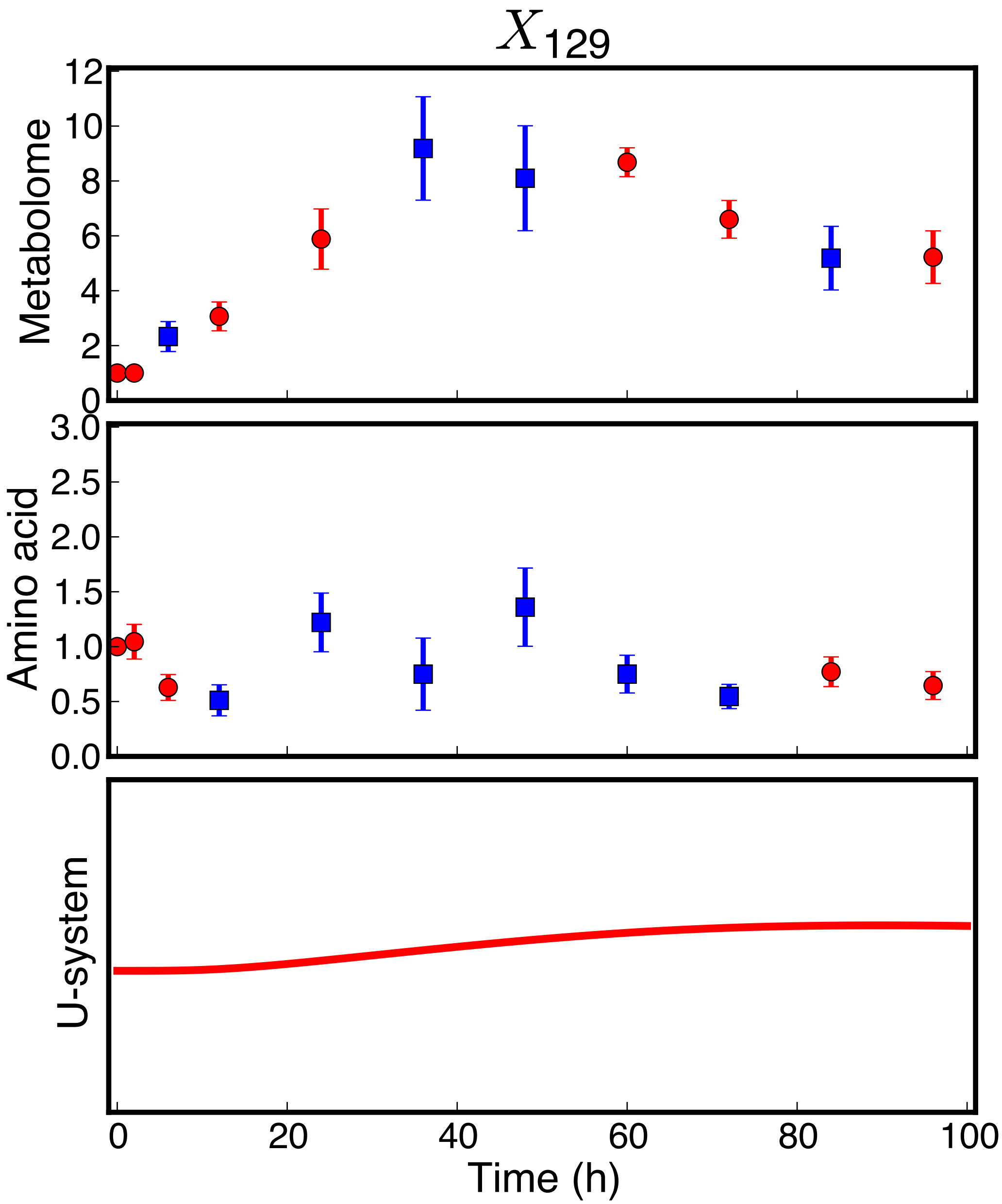

X_{129}
Metabolome
0
2
4
6
8
10
12
Amino acid
0.0
0.5
1.0
1.5
2.0
2.5
3.0
U-system
0
20
40
60
80
100
Time (h)

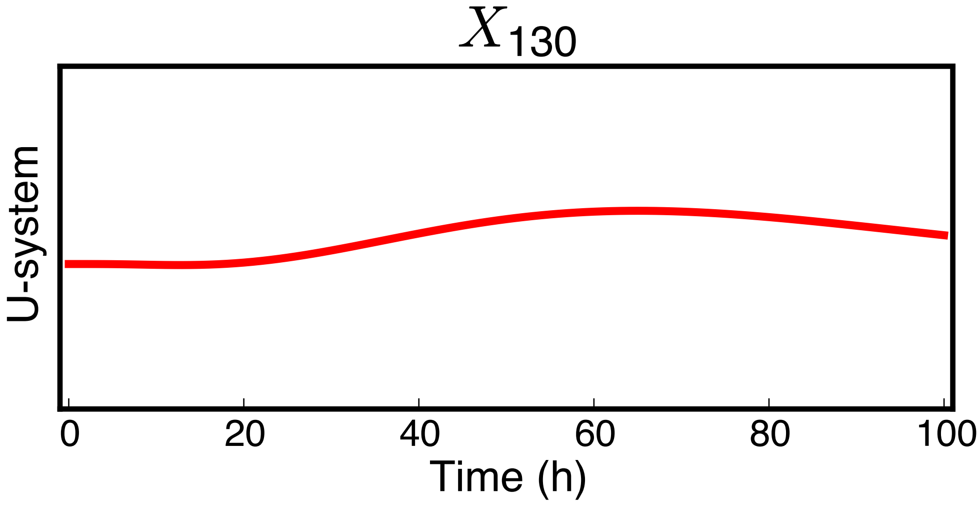
X_{130}
U-system
0
20
40
60
80
100
Time (h)

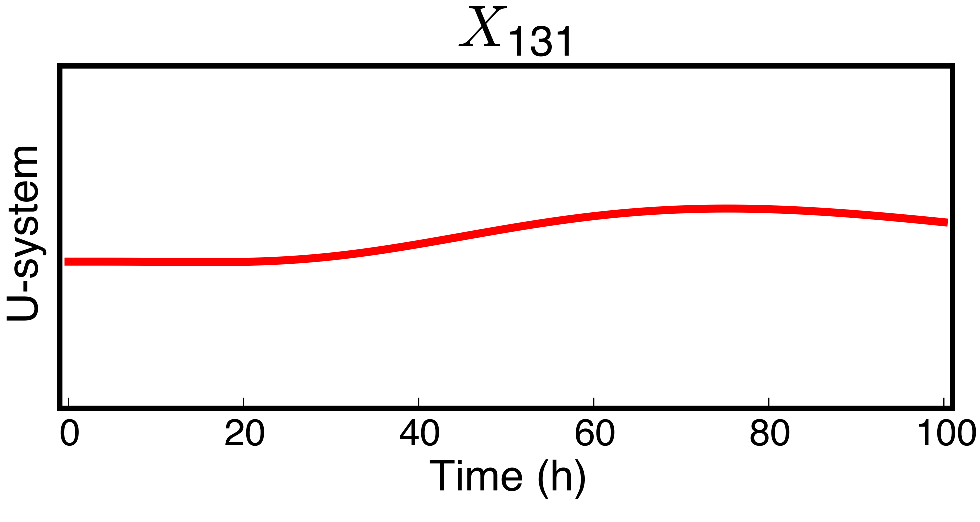
X_{131}
U-system
0
20
40
60
80
100
Time (h)

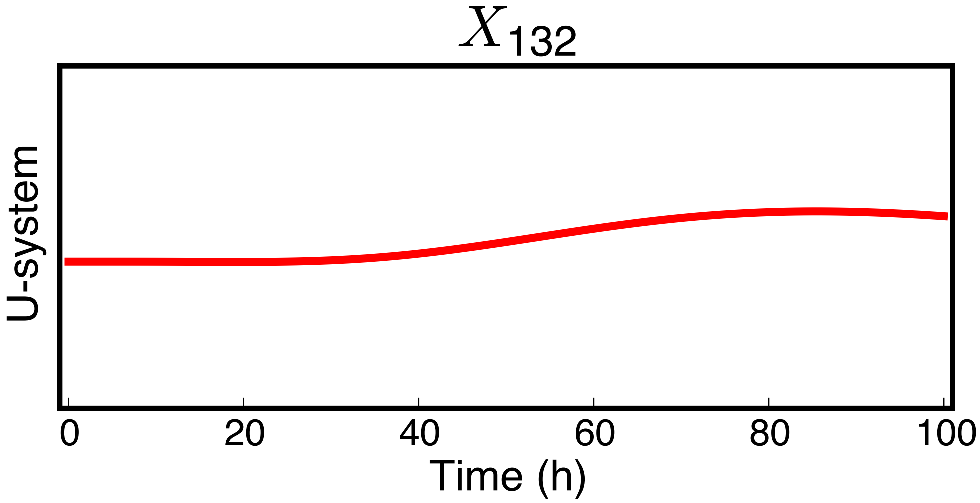

X_{132}
U-system
0
20
40
60
80
100
Time (h)

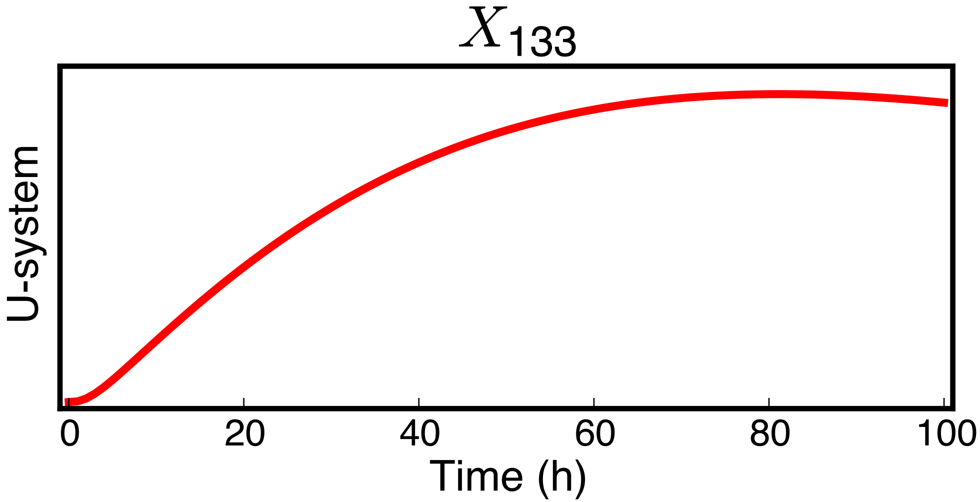

X_{133}
U-system
0
20
40
60
80
100
Time (h)

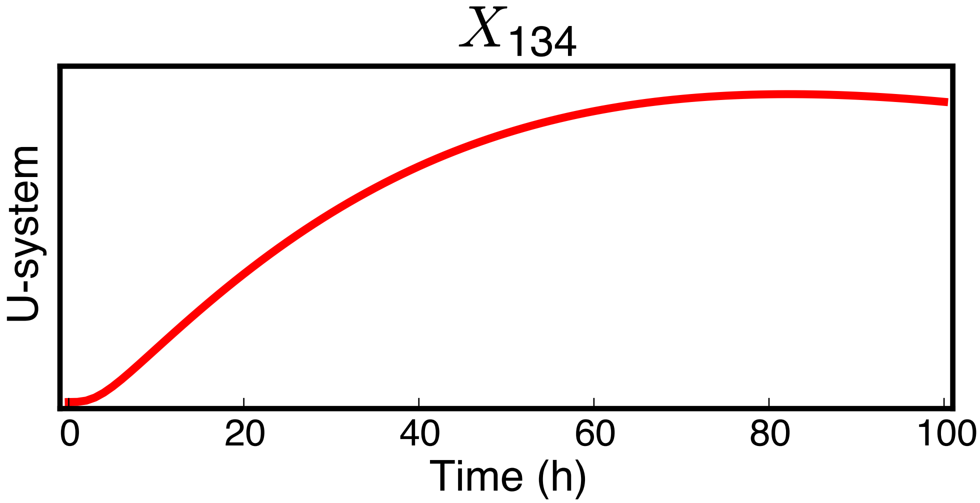

X_{134}
U-system
0
20
40
60
80
100
Time (h)

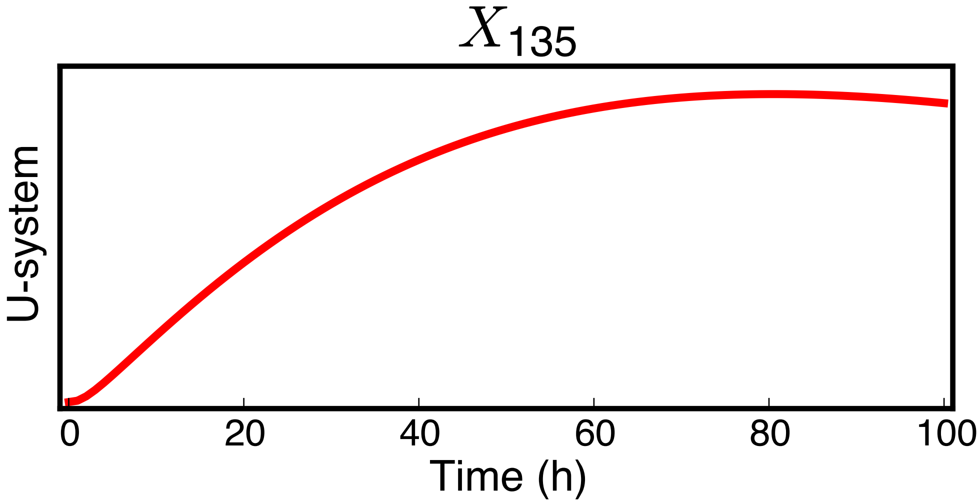

X_{135}
U-system
0
20
40
60
80
100
Time (h)

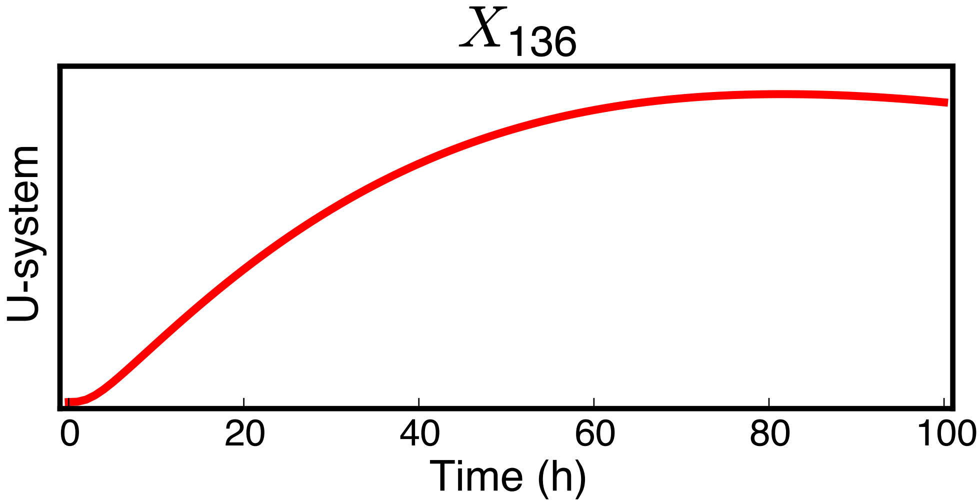

X_{136}
U-system
0
20
40
60
80
100
Time (h)

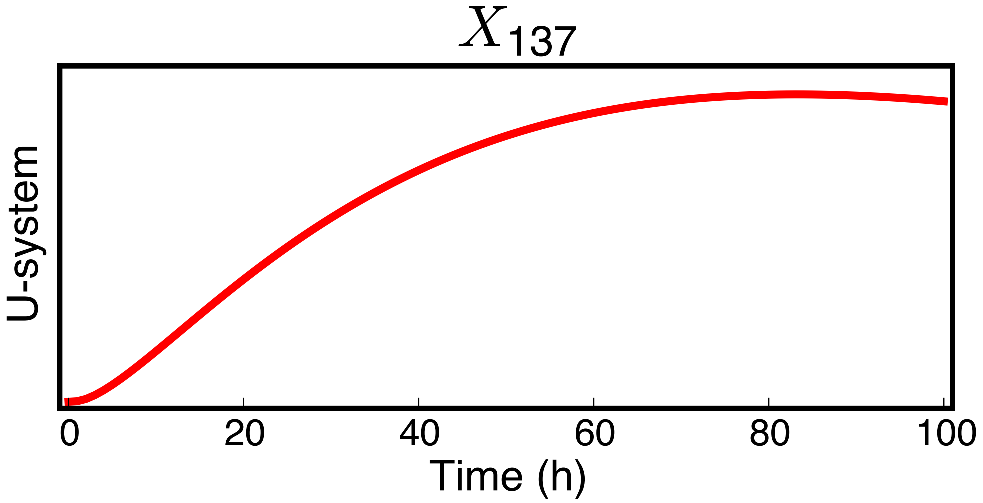

X_{137}
U-system
0
20
40
60
80
100
Time (h)

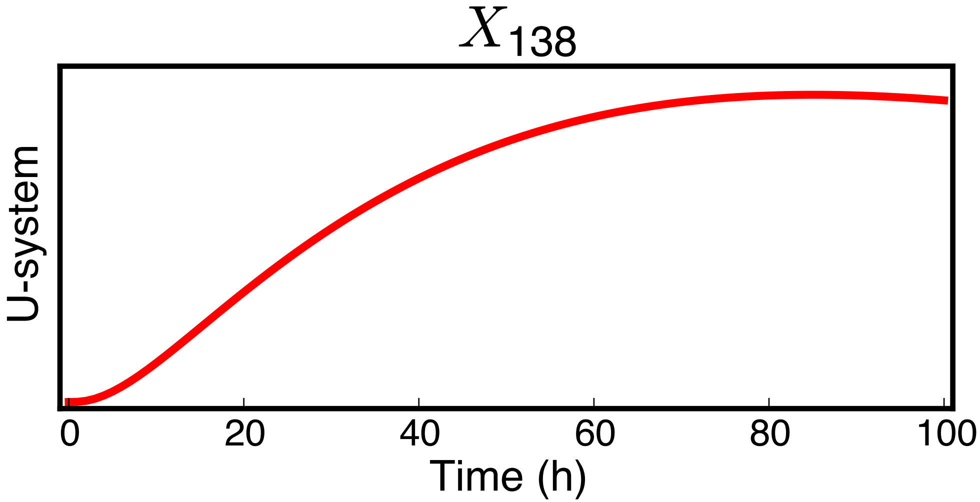

X_{138}
U-system
0
20
40
60
80
100
Time (h)

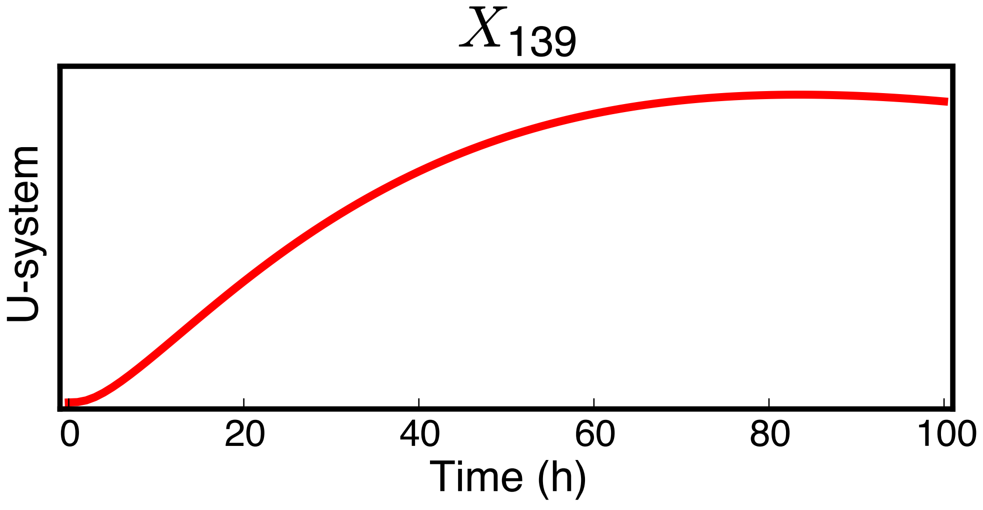

X_{139}
U-system
0
20
40
60
80
100
Time (h)

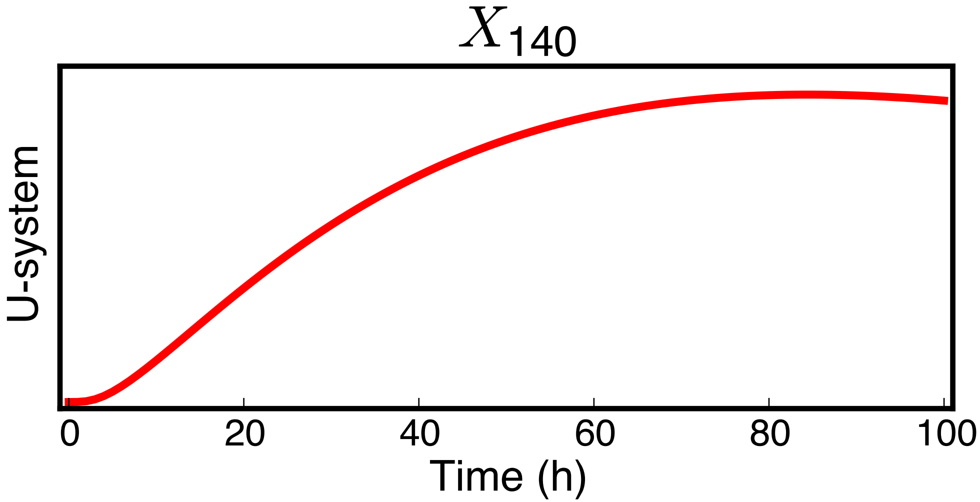

X_{140}
U-system
0
20
40
60
80
100
Time (h)

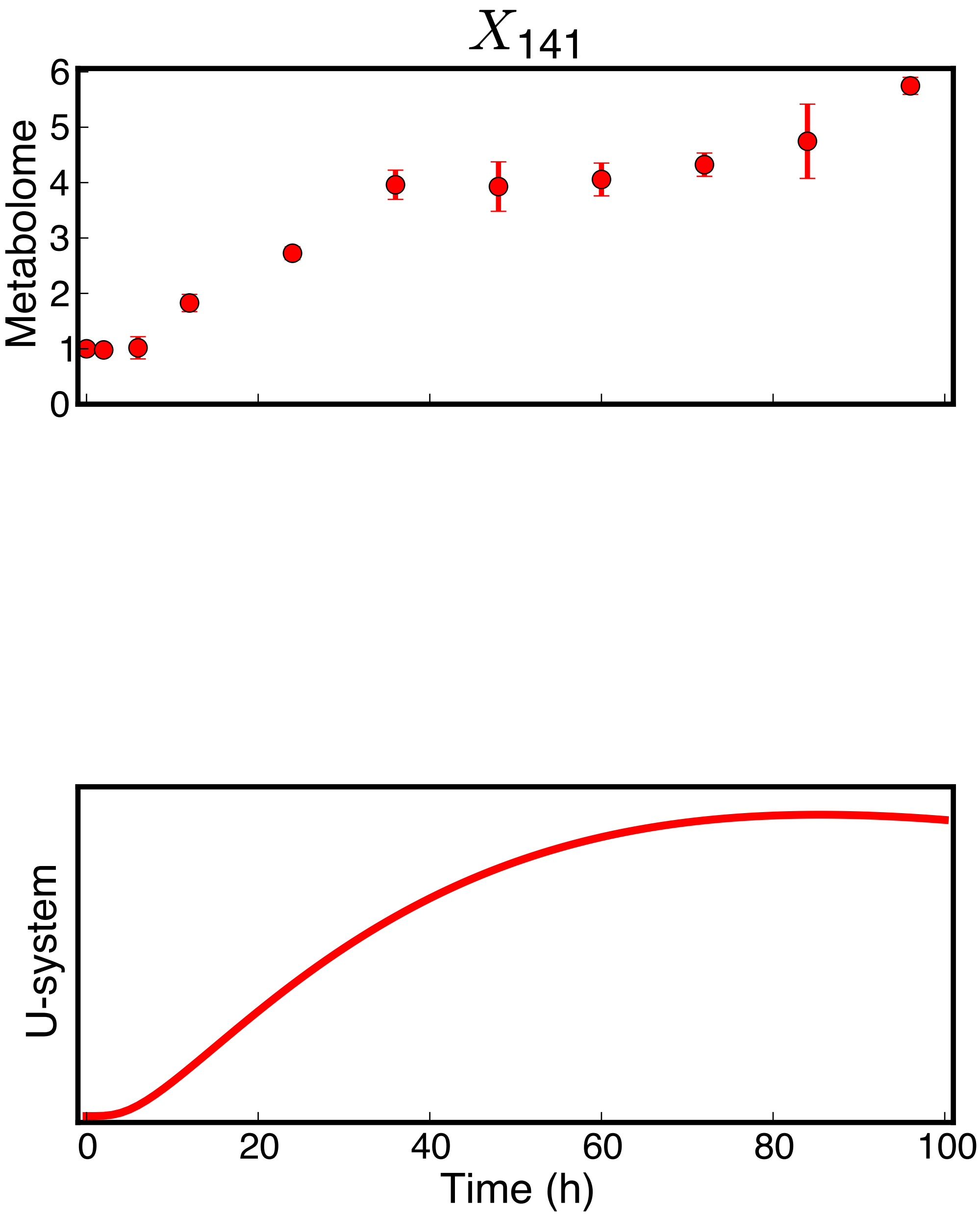

X_{141}
Metabolome
0
1
2
3
4
5
6
U-system
0
20
40
60
80
100
Time (h)

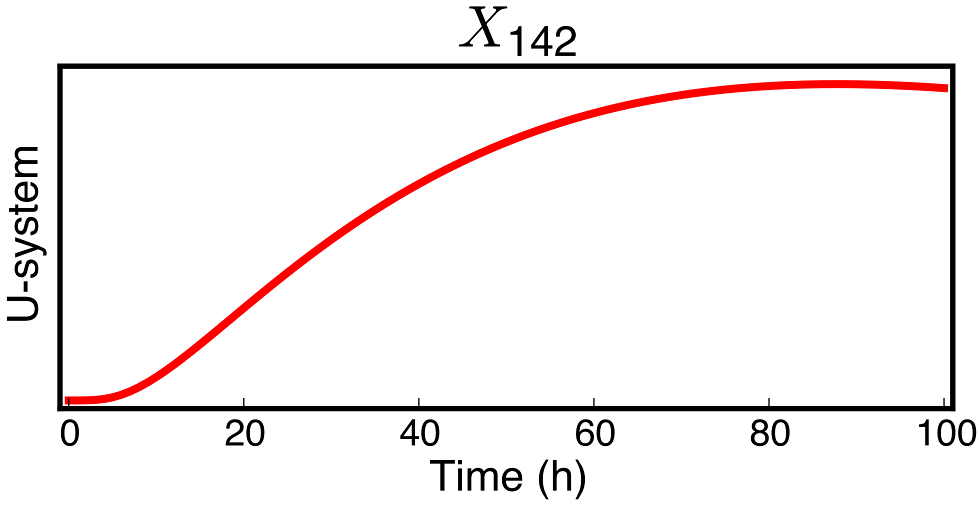

X_{142}
U-system
0
20
40
60
80
100
Time (h)

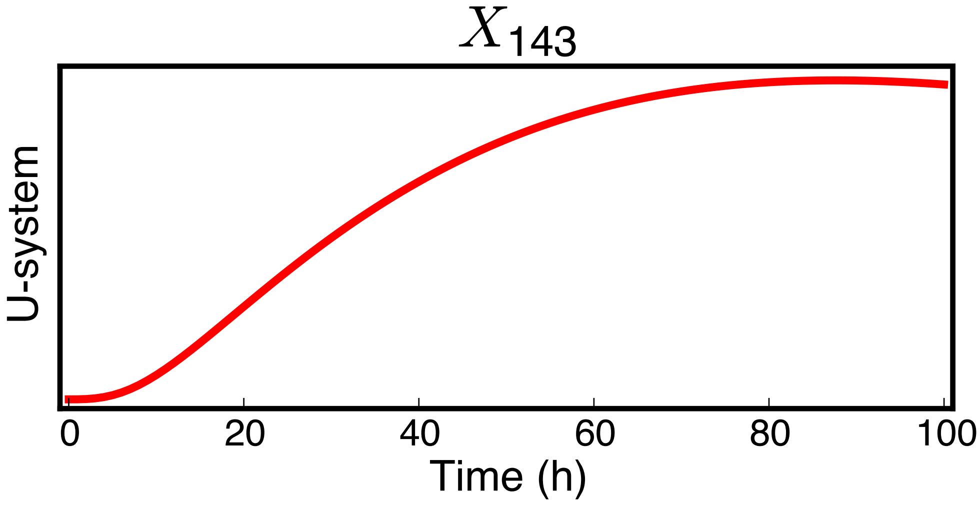

X_{143}
U-system
0
20
40
60
80
100
Time (h)

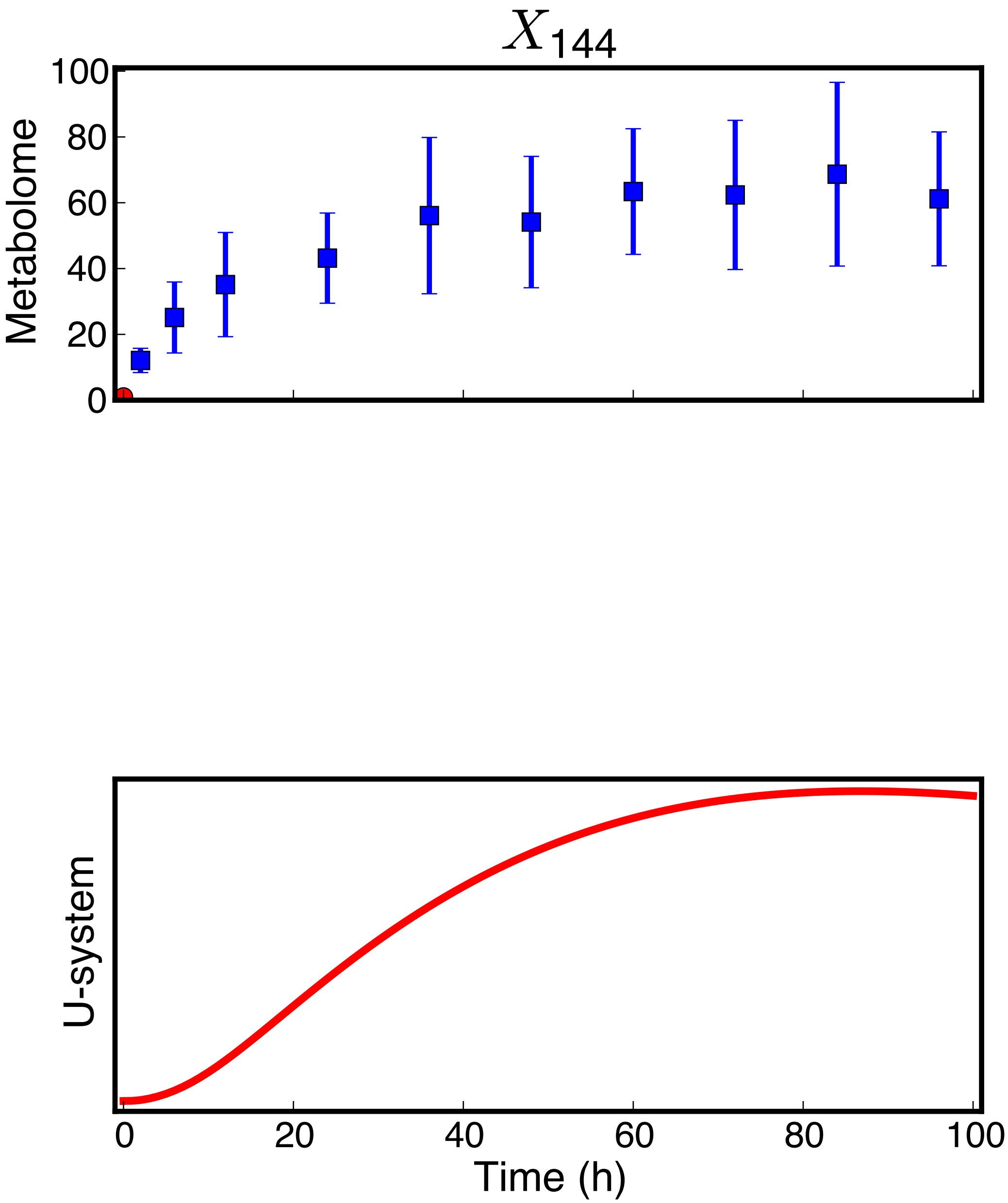

X_{144}
Metabolome
100
80
60
40
20
0
U-system
0
20
40
60
80
100
Time (h)

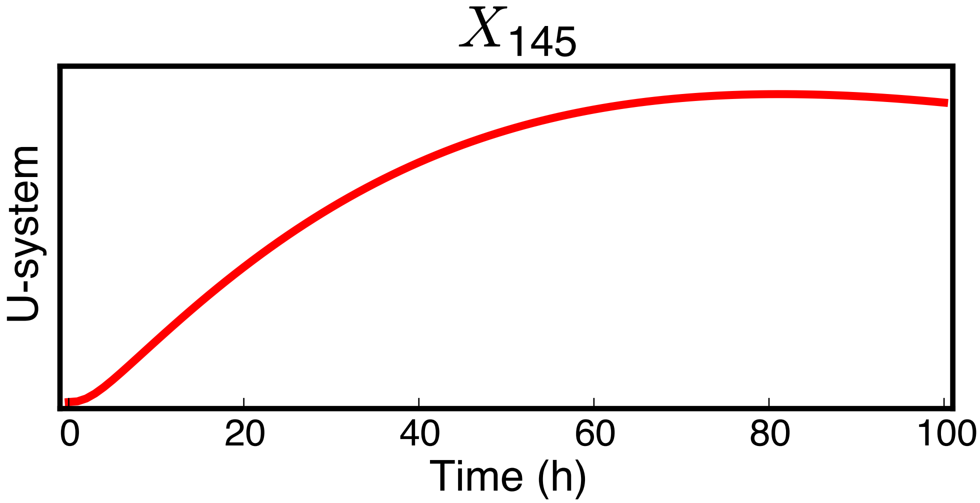
X_{145}
U-system
0
20
40
60
80
100
Time (h)

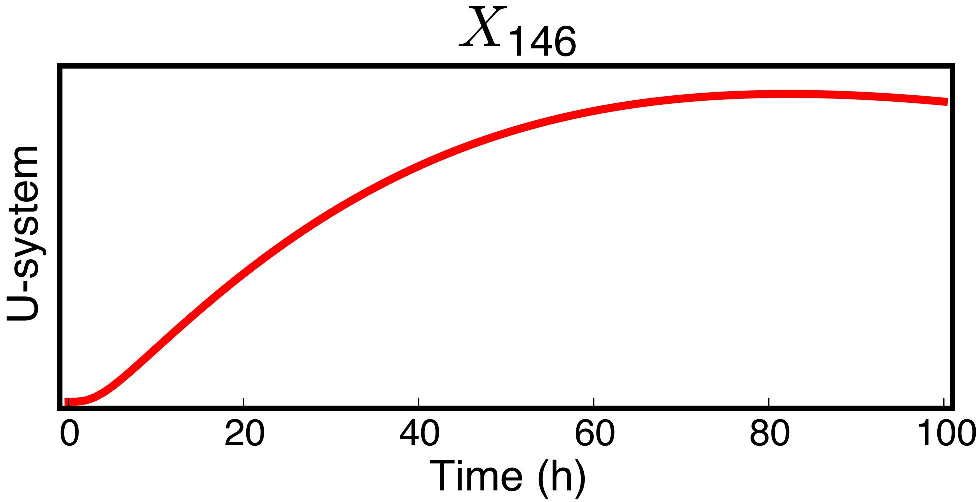

X_{146}
U-system
0
20
40
60
80
100
Time (h)

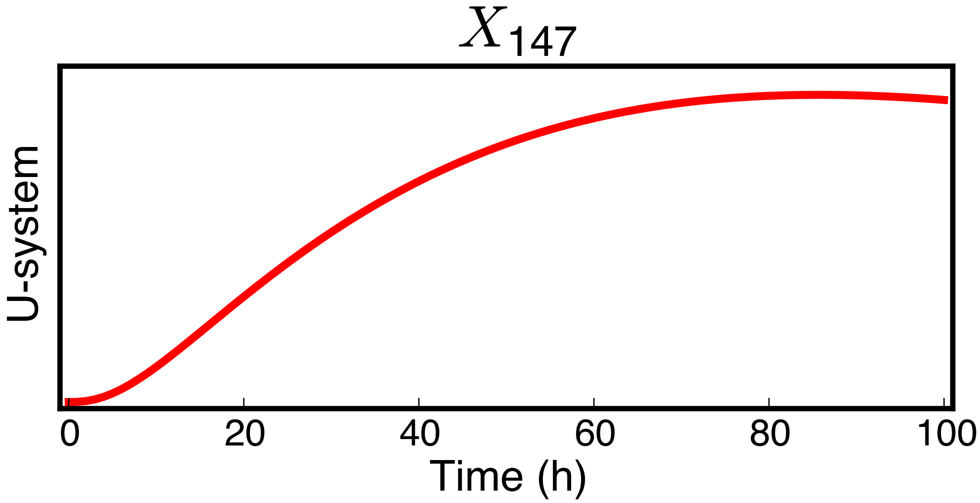

X_{147}
U-system
0
20
40
60
80
100
Time (h)

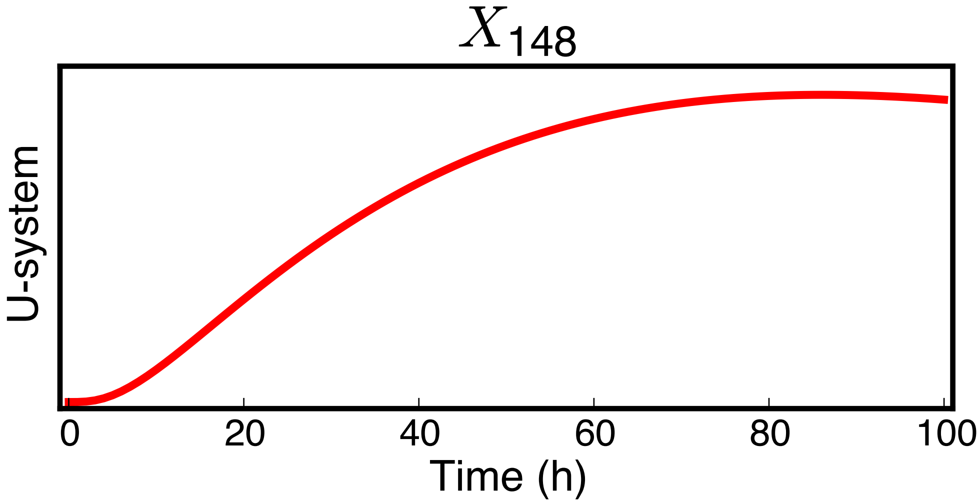

X_{148}
U-system
0
20
40
60
80
100
Time (h)

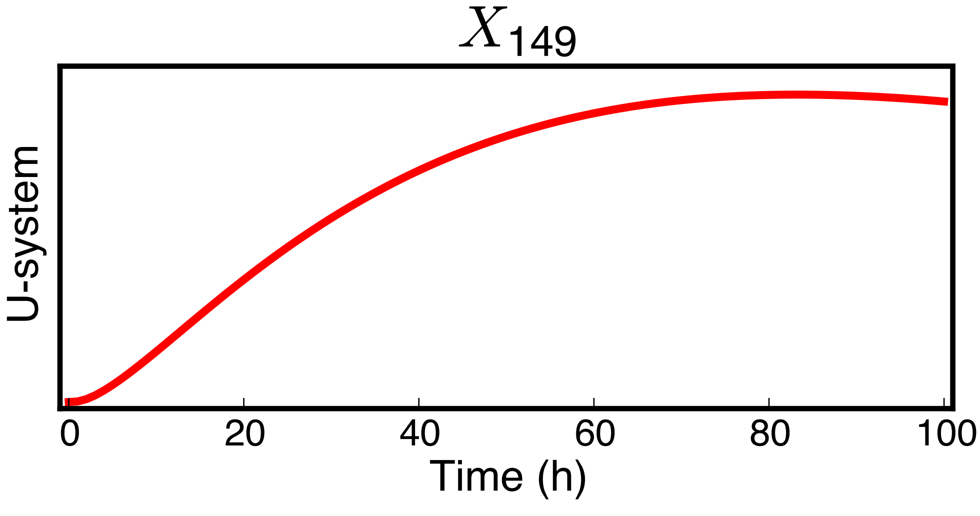

X_{149}
U-system
0
20
40
60
80
100
Time (h)

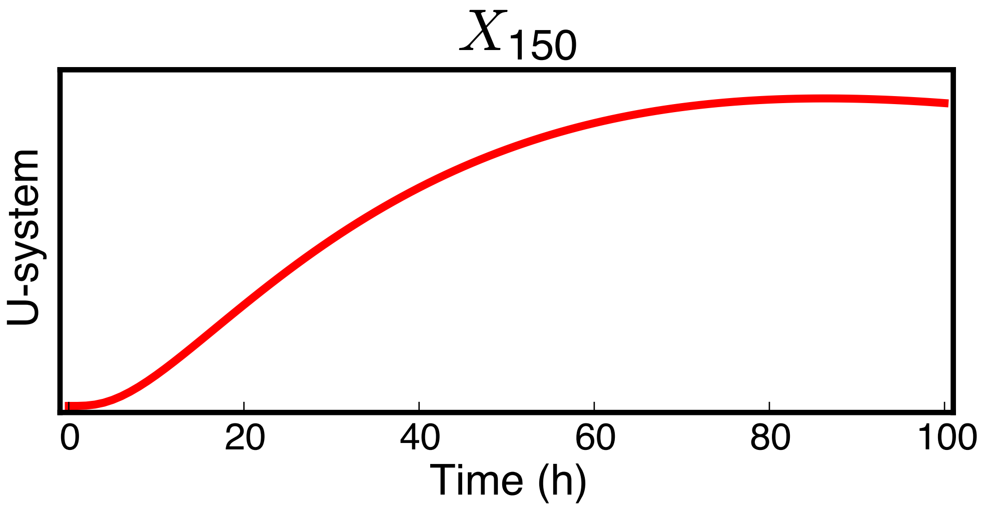

X_{150}
U-system
0
20
40
60
80
100
Time (h)

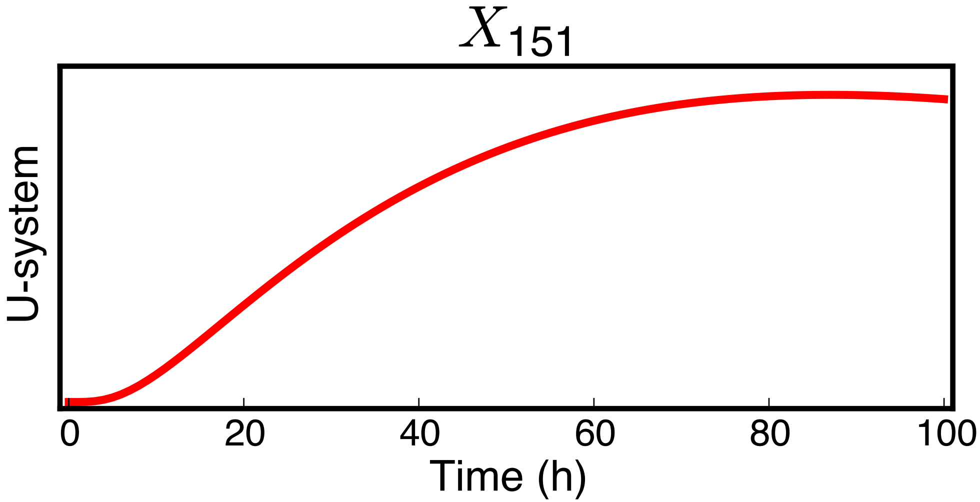

X_{151}
U-system
0
20
40
60
80
100
Time (h)

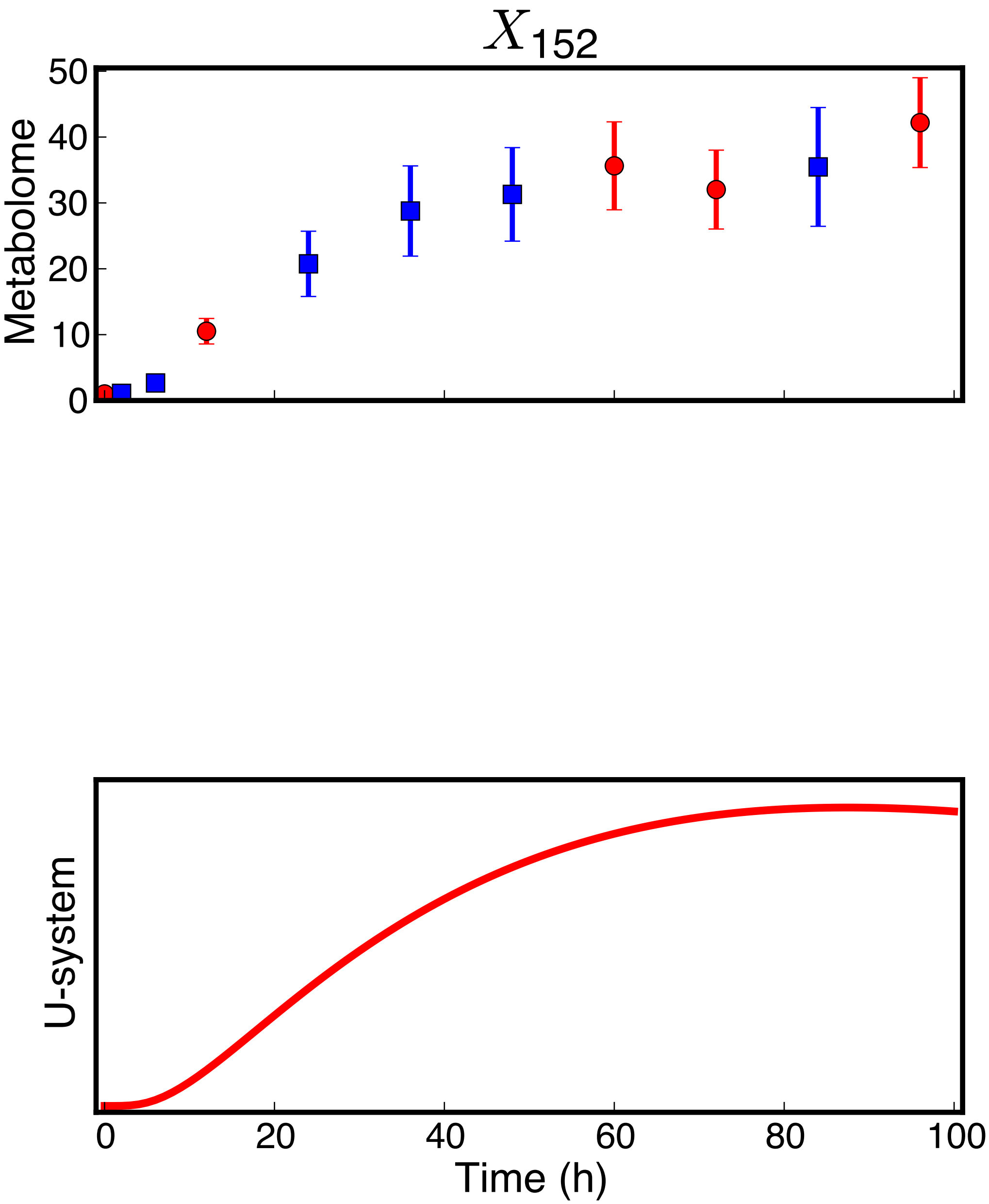

X_{152}
Metabolome
50
40
30
20
10
0
U-system
0
20
40
60
80
100
Time (h)

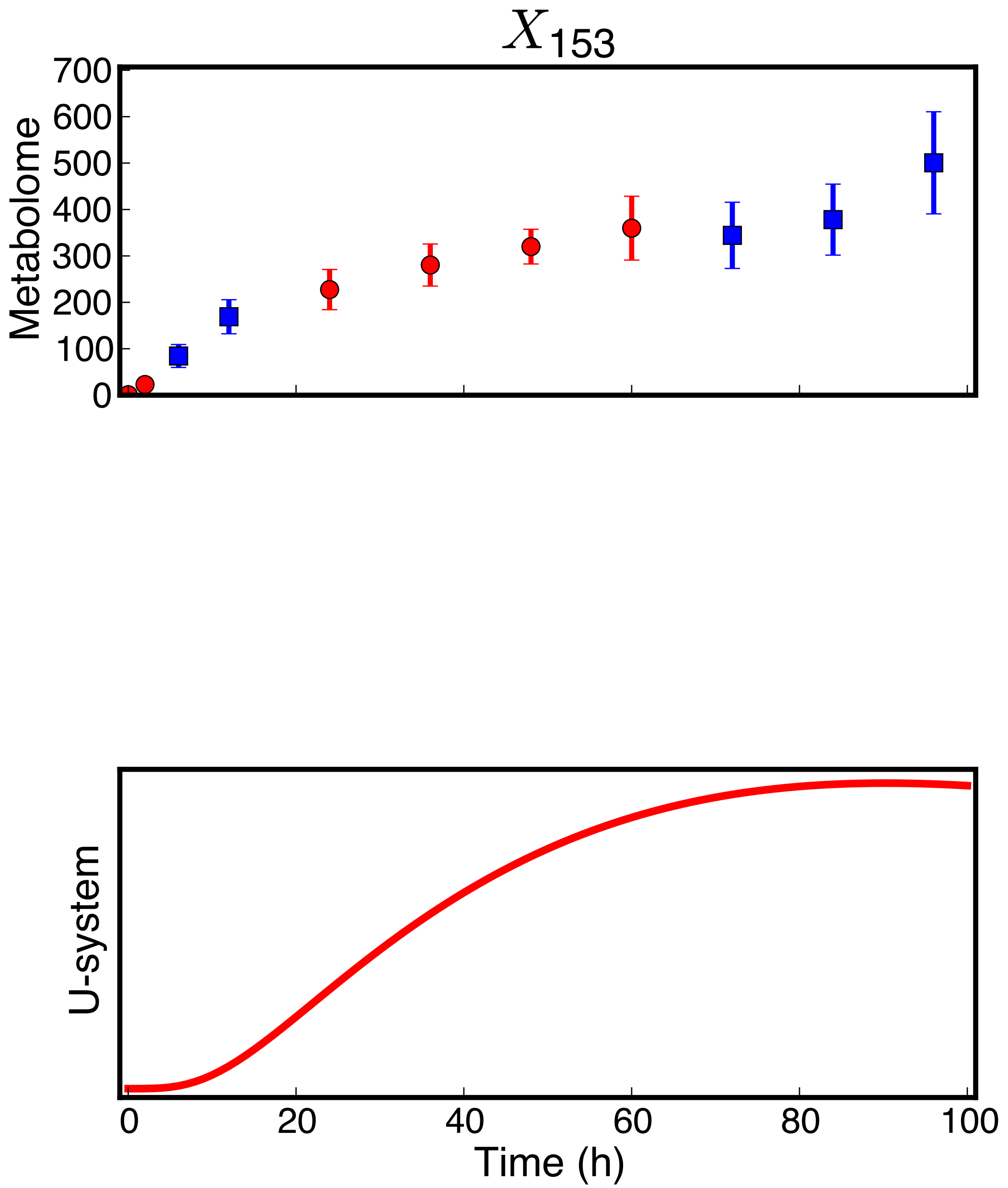

X_{153}
Metabolome
700
600
500
400
300
200
100
0
U-system
0
20
40
60
80
100
Time (h)

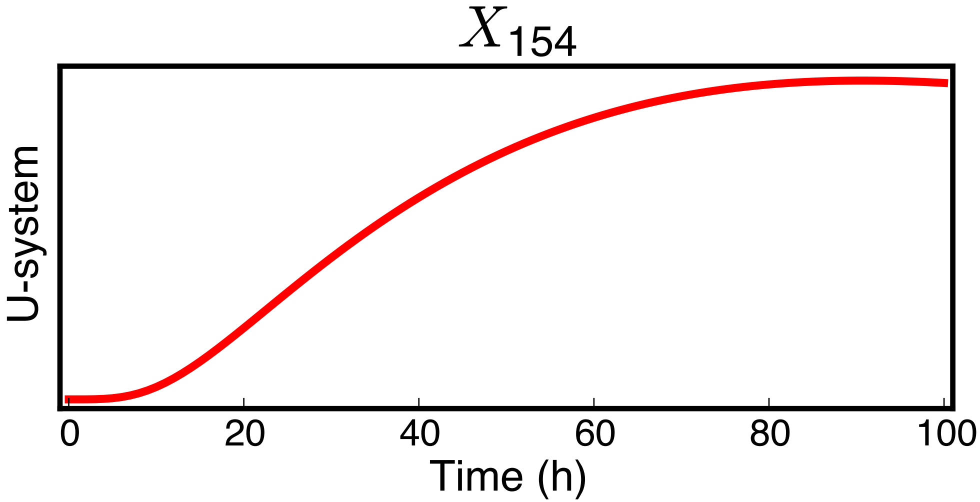

X_{154}
U-system
0
20
40
60
80
100
Time (h)

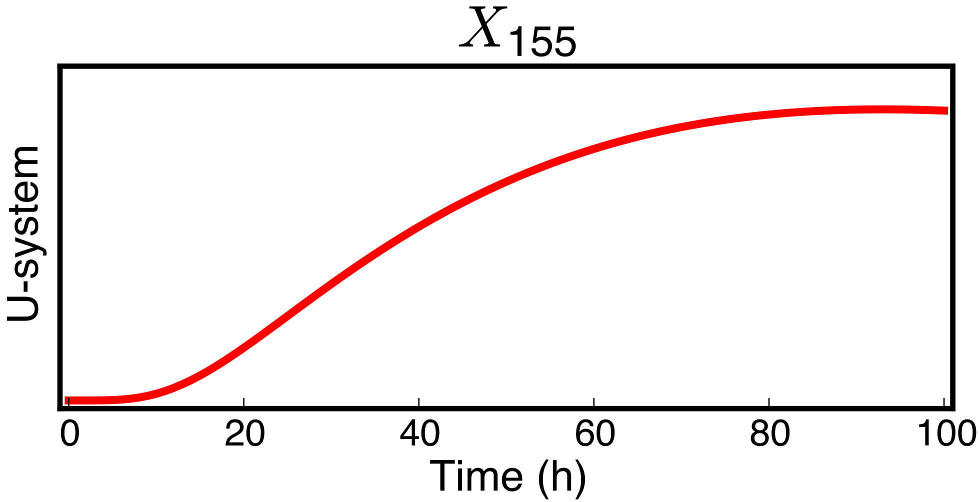
X_{155}
U-system
0
20
40
60
80
100
Time (h)

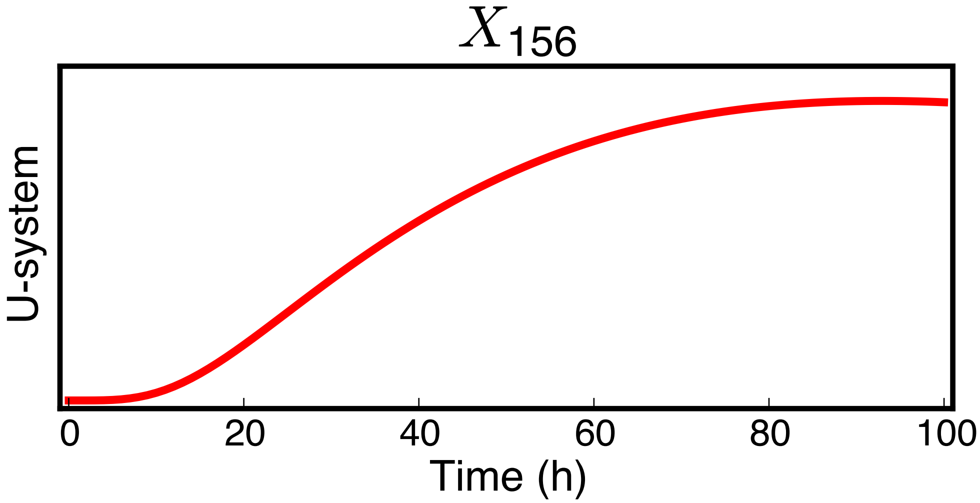
X_{156}
U-system
0
20
40
60
80
100
Time (h)

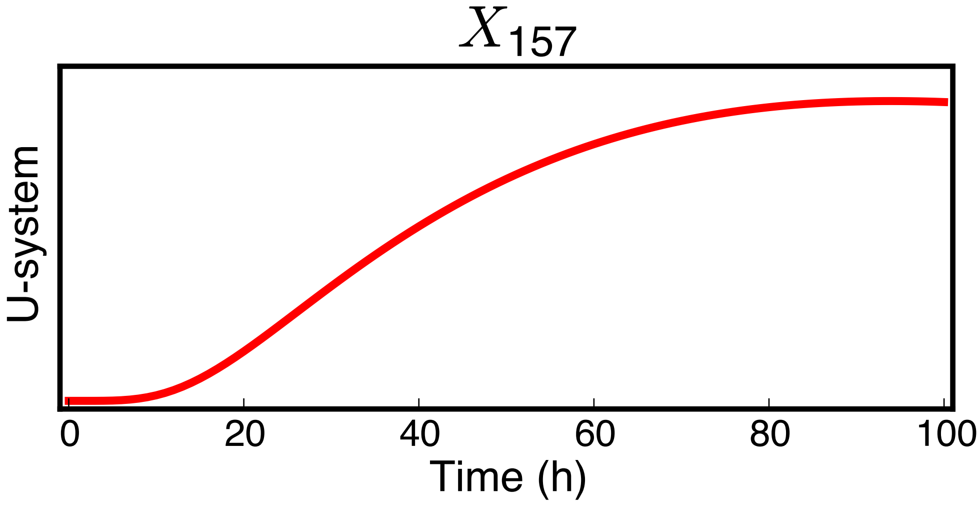

X_{157}
U-system
0
20
40
60
80
100
Time (h)

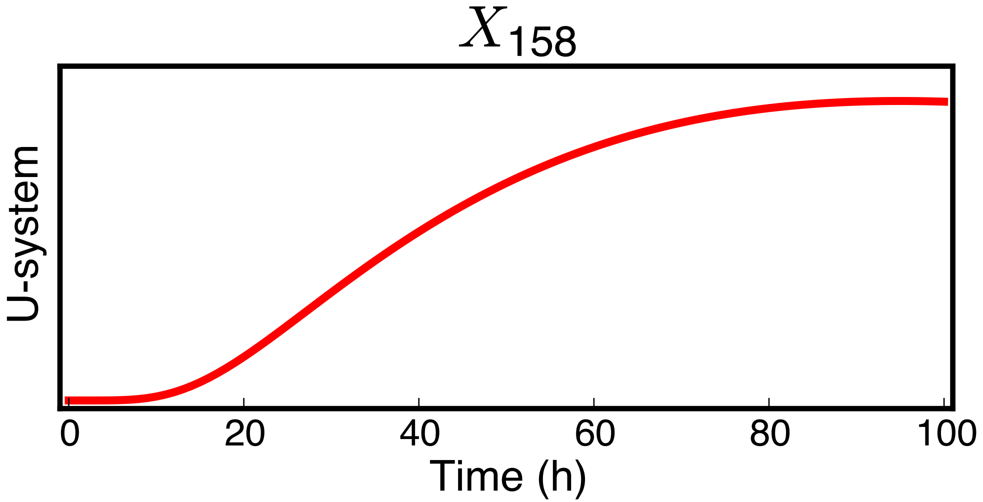

X_{158}
U-system
0
20
40
60
80
100
Time (h)

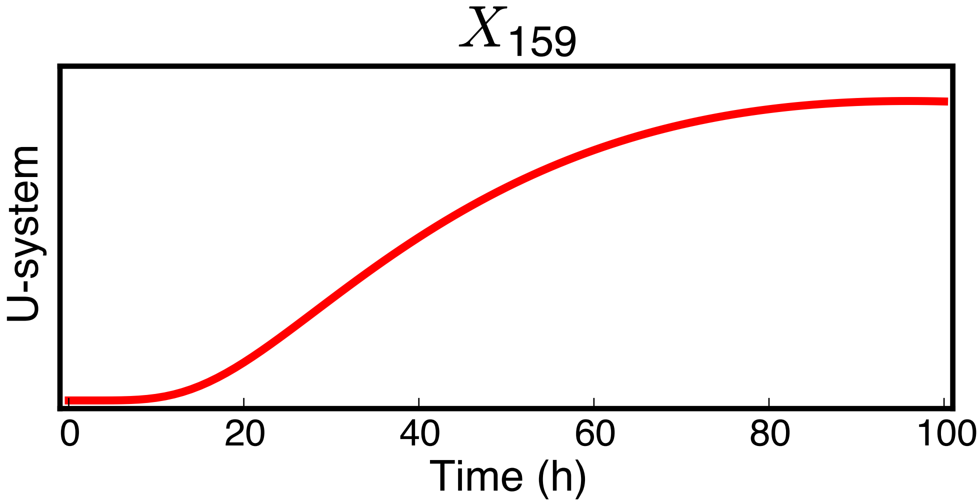

X_{159}
U-system
0
20
40
60
80
100
Time (h)

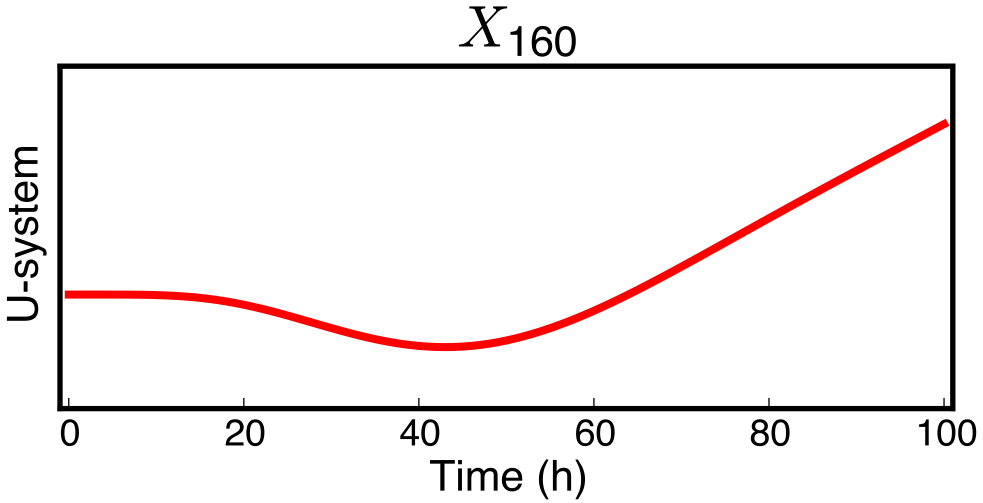
X_{160}
U-system
0
20
40
60
80
100
Time (h)

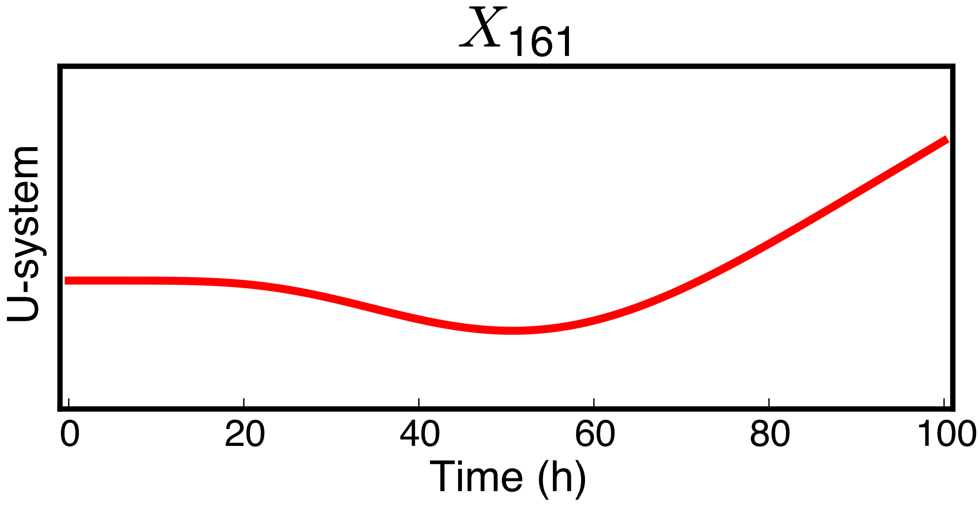

X_{161}
U-system
0
20
40
60
80
100
Time (h)

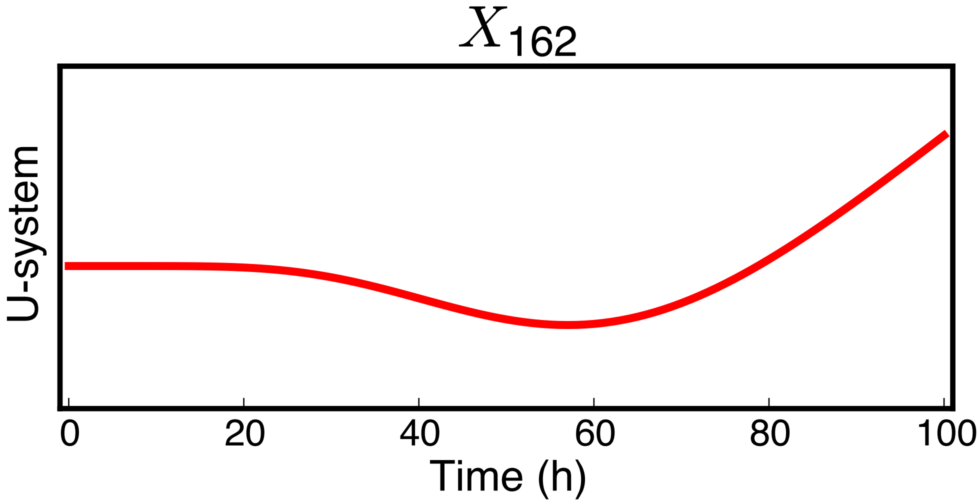
X_{162}
U-system
0
20
40
60
80
100
Time (h)

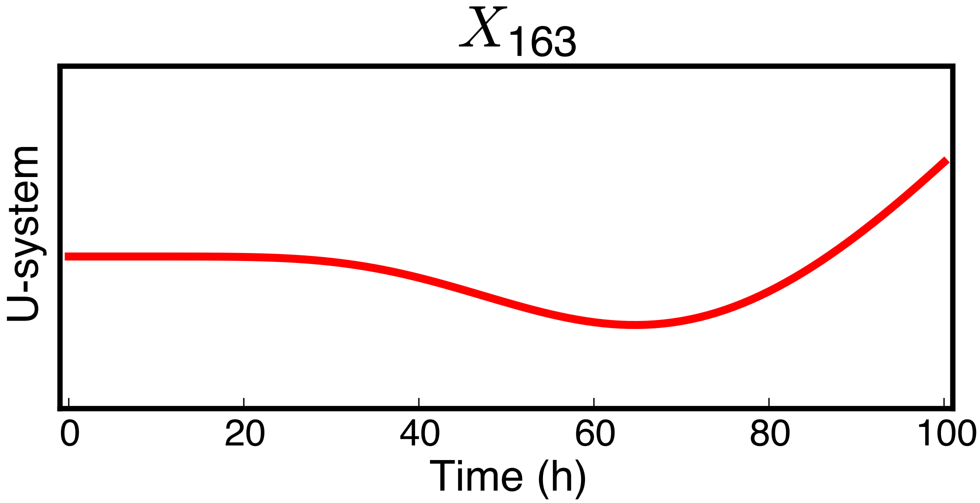

X_{163}
U-system
0
20
40
60
80
100
Time (h)

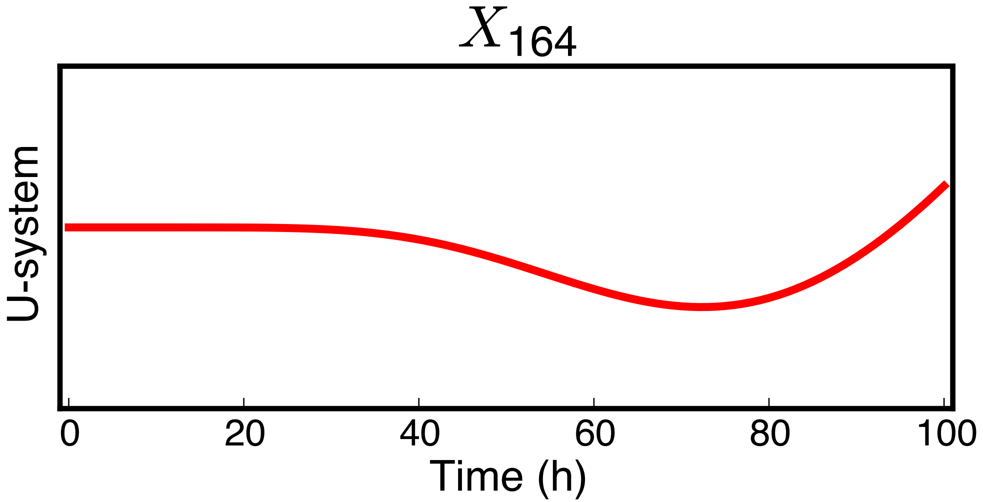

X_{164}
U-system
0
20
40
60
80
100
Time (h)

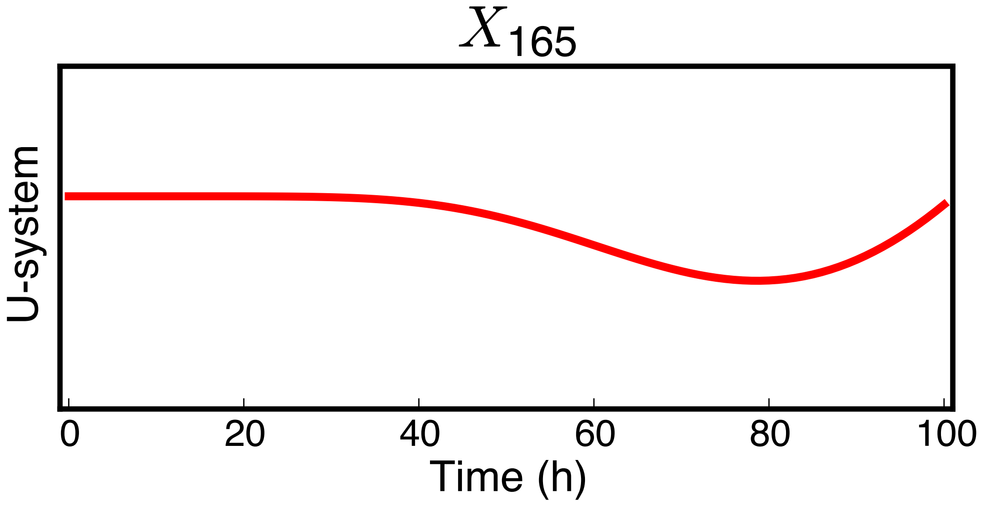

X_{165}
U-system
0
20
40
60
80
100
Time (h)

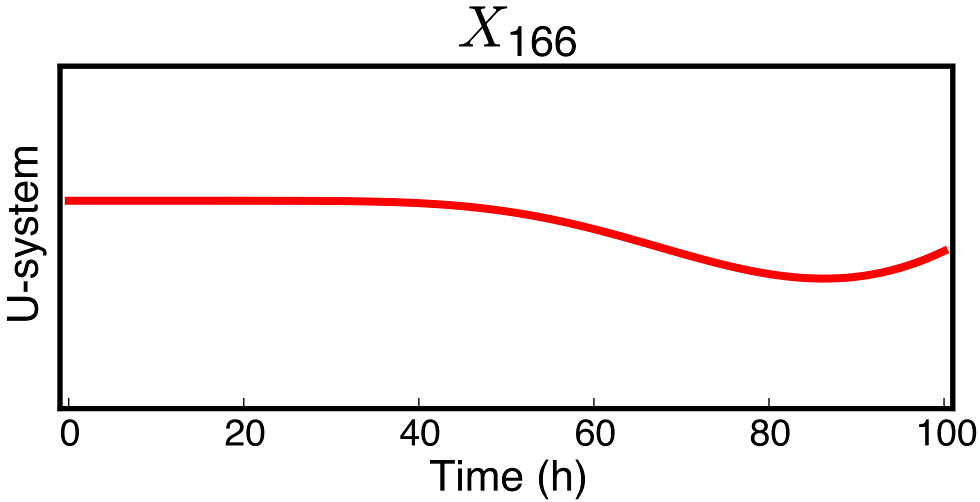
X_{166}
U-system
0
20
40
60
80
100
Time (h)

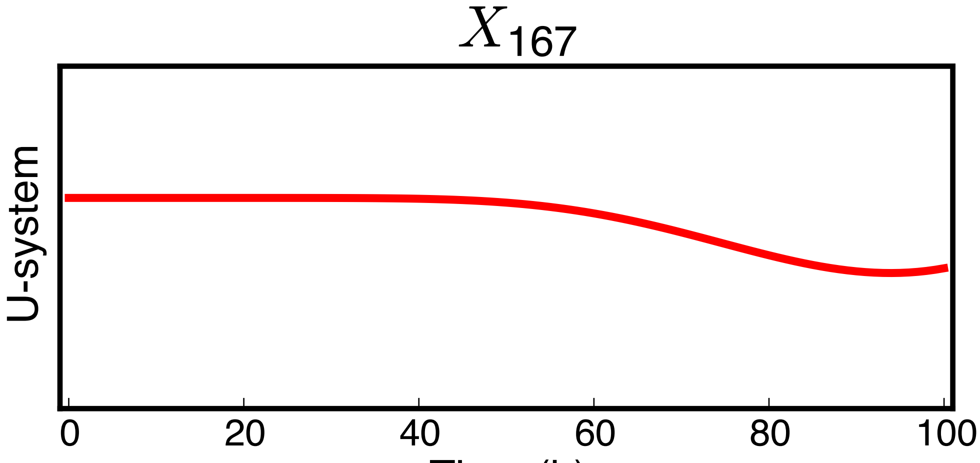

X_{167}
U-system
0
20
40
60
80
100
Time (h)

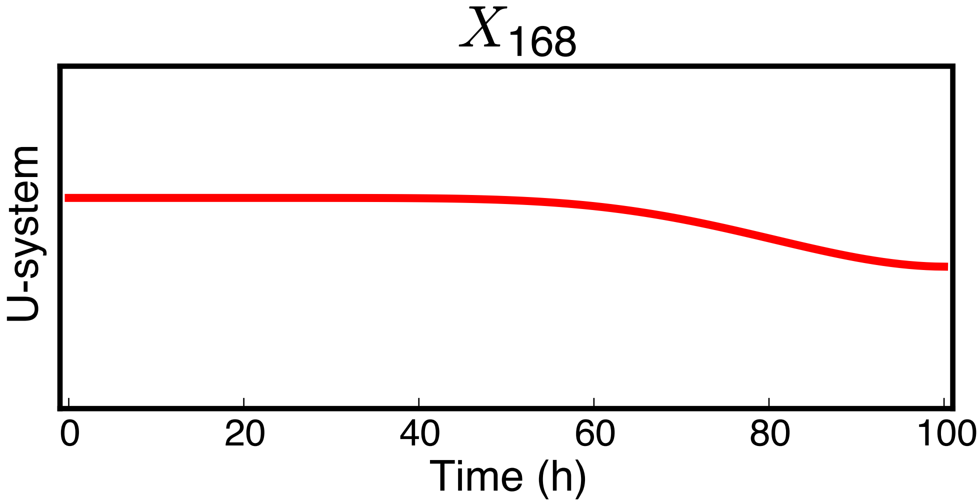

X_{168}
U-system
0
20
40
60
80
100
Time (h)

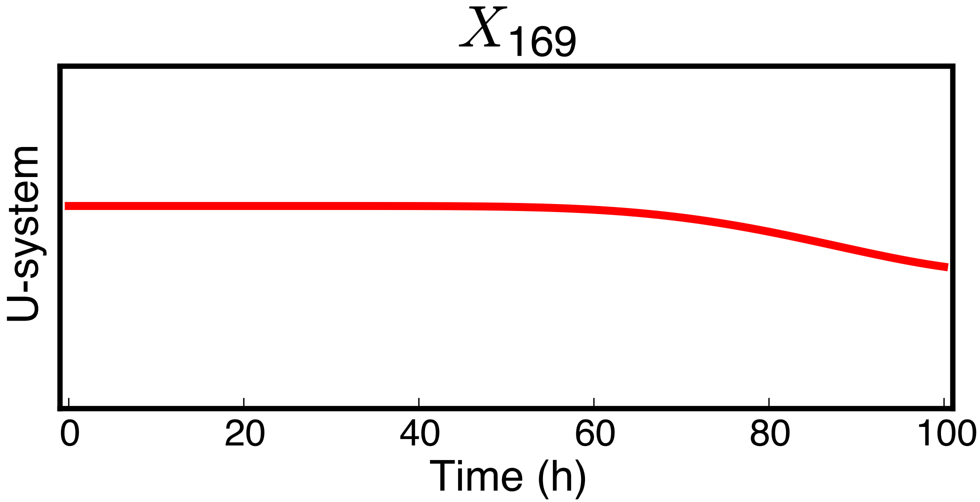
X_{169}
U-system
0
20
40
60
80
100
Time (h)

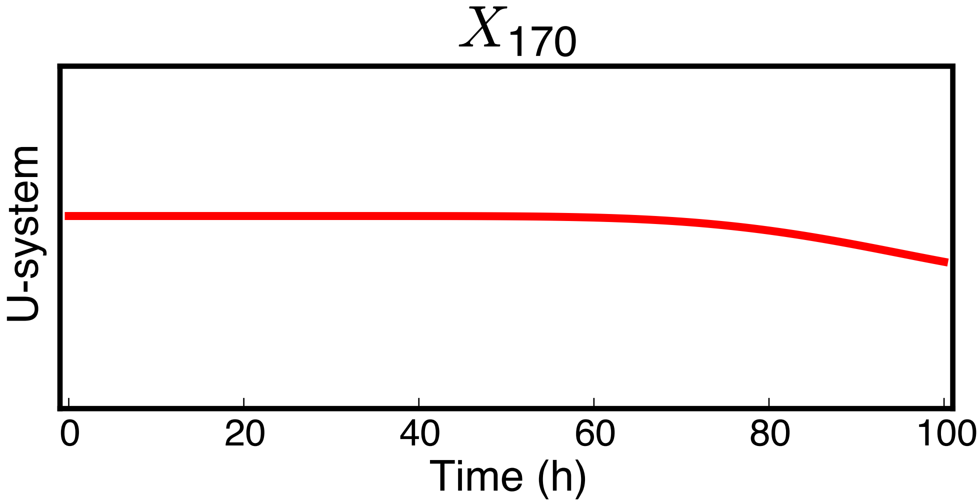

X_{170}
U-system
0
20
40
60
80
100
Time (h)

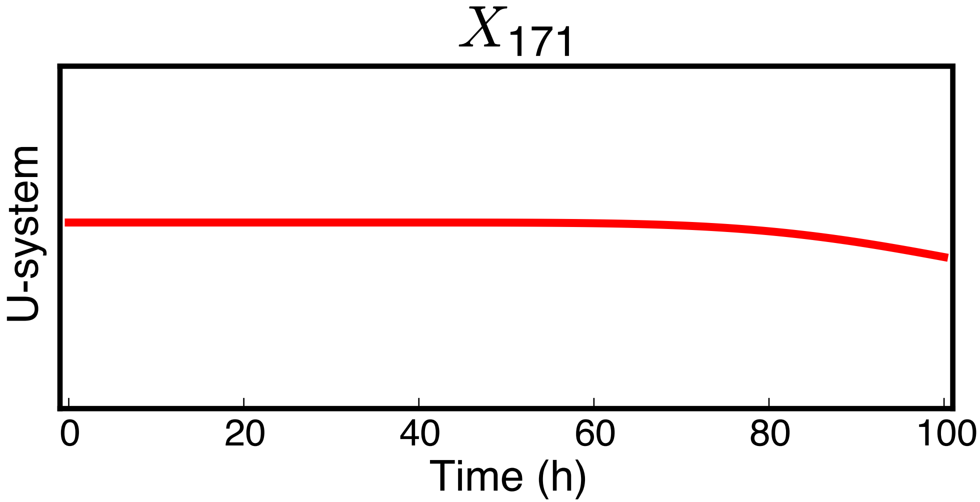
X_{171}
U-system
0
20
40
60
80
100
Time (h)

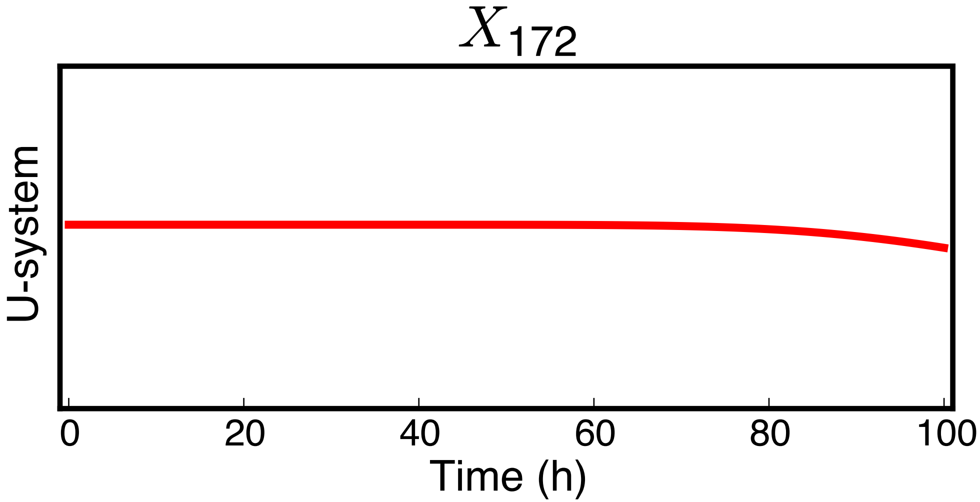
X_{172}
U-system
0
20
40
60
80
100
Time (h)

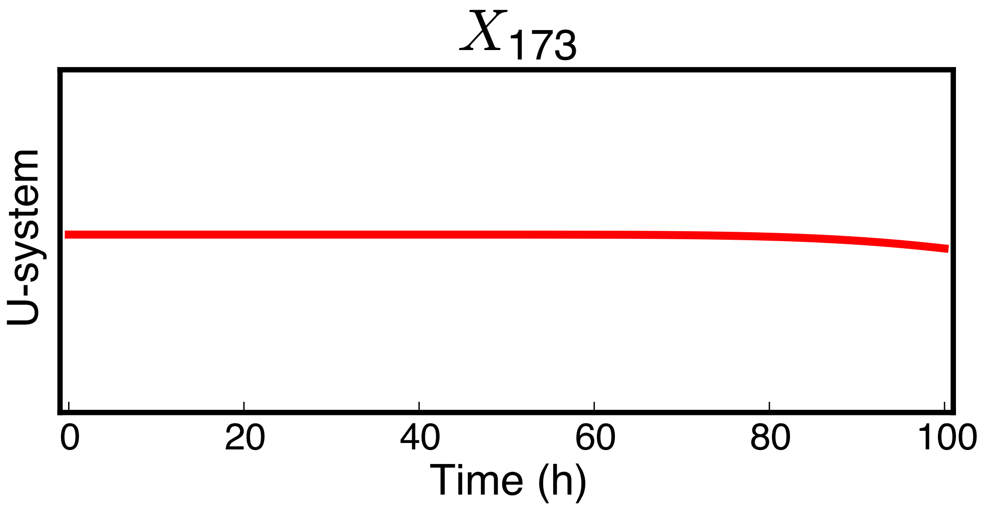

X_{173}
U-system
0
20
40
60
80
100
Time (h)

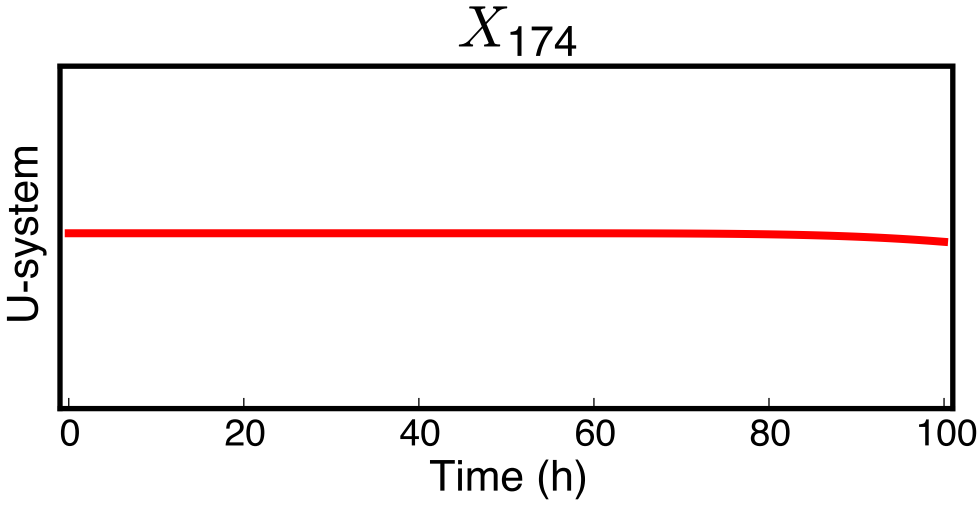

X_{174}
U-system
0
20
40
60
80
100
Time (h)

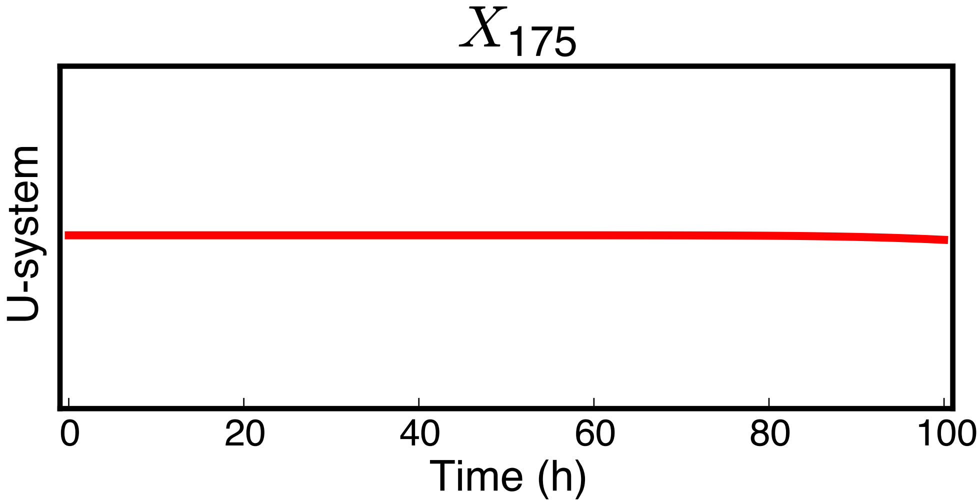

X_{175}
U-system
0
20
40
60
80
100
Time (h)

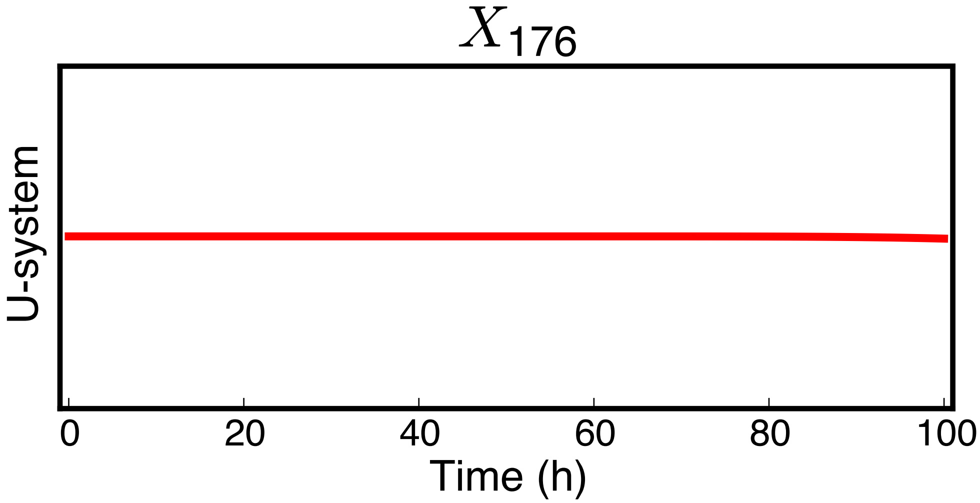
X_{176}
U-system
0
20
40
60
80
100
Time (h)

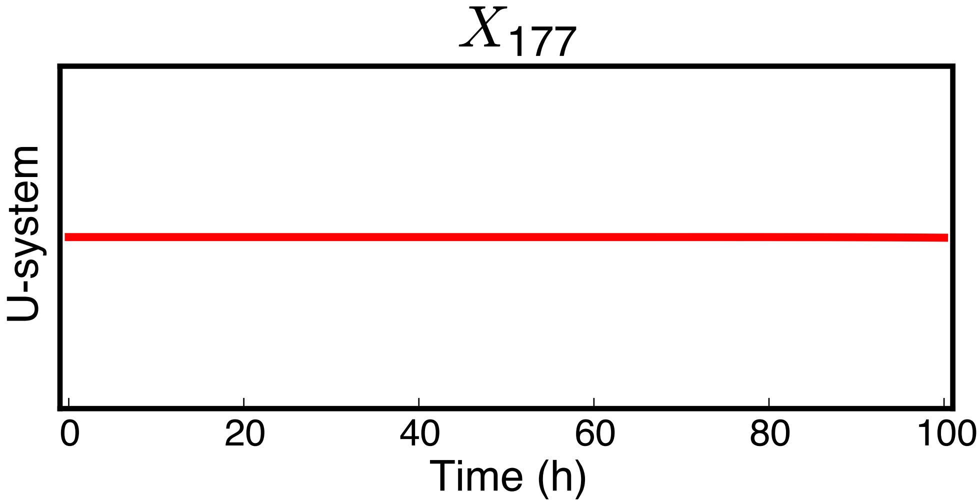
X_{177}
U-system
0
20
40
60
80
100
Time (h)

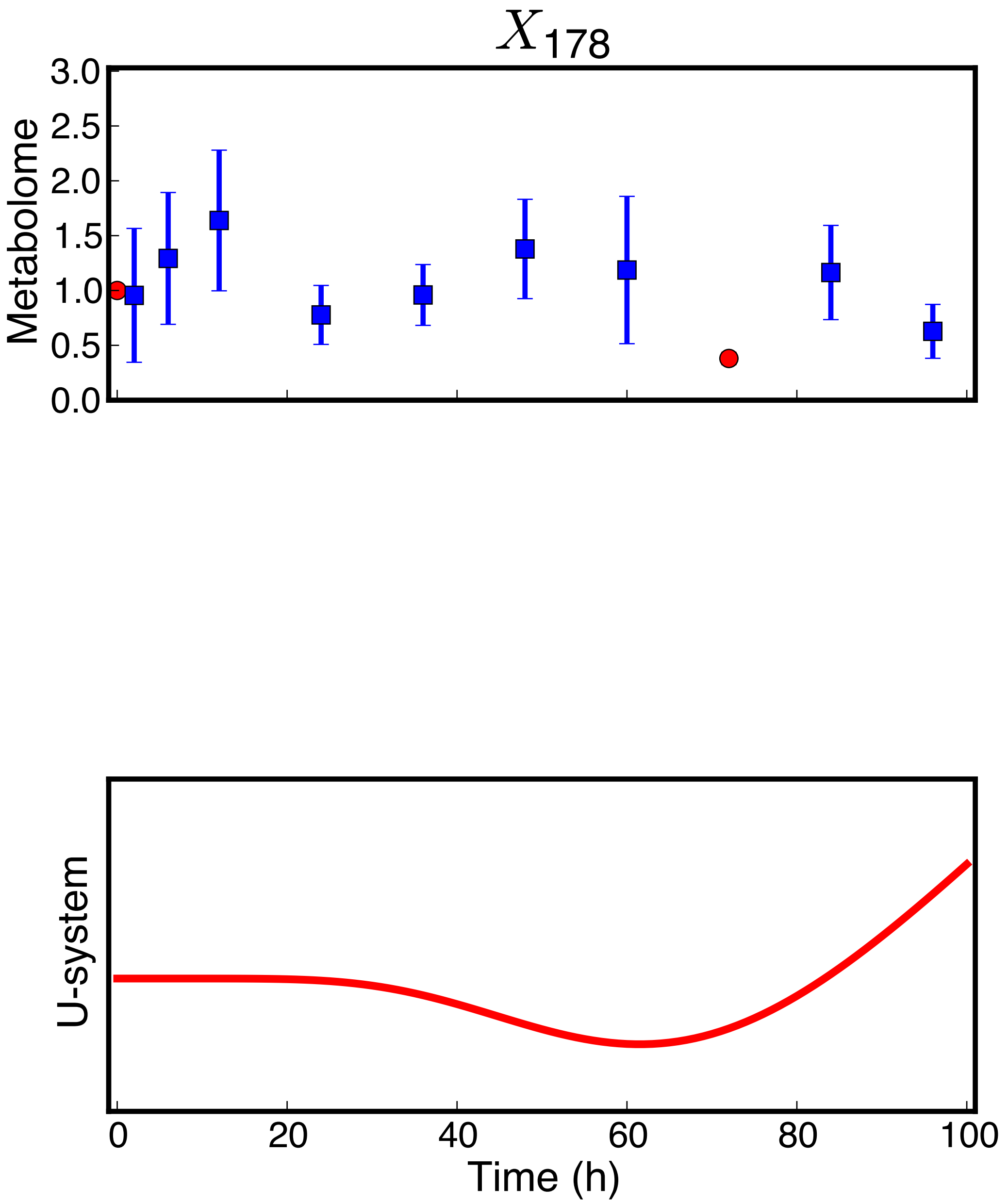

X_{178}
Metabolome
3.0
2.5
2.0
1.5
1.0
0.5
0.0
U-system
0
20
40
60
80
100
Time (h)

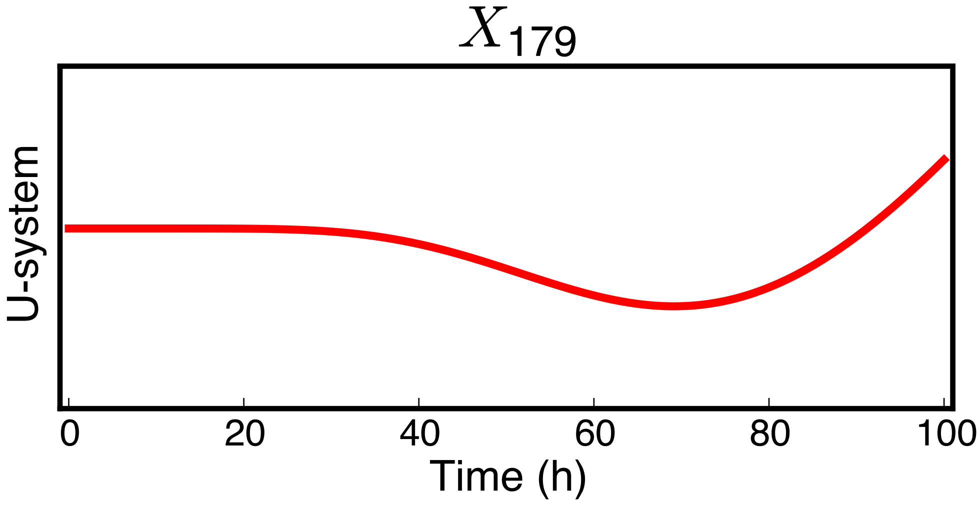

X_{179}
U-system
0
20
40
60
80
100
Time (h)

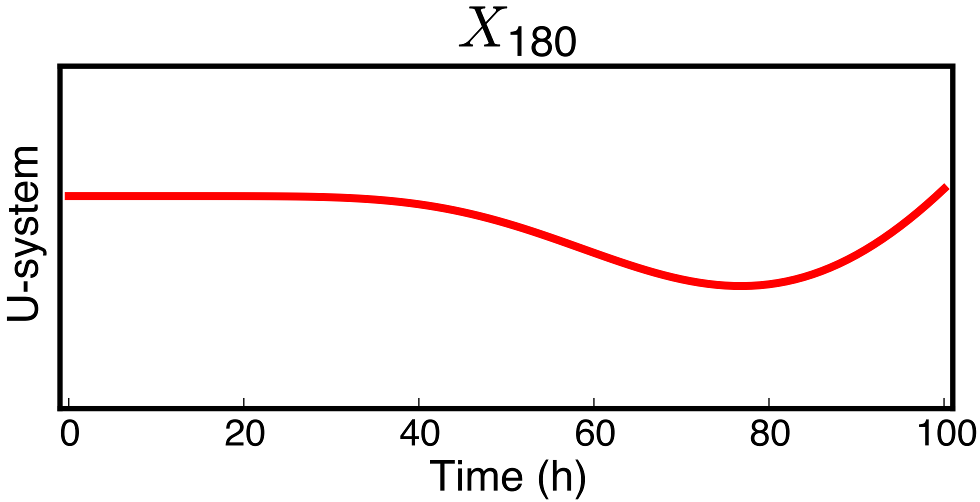

X_{180}
U-system
0
20
40
60
80
100
Time (h)

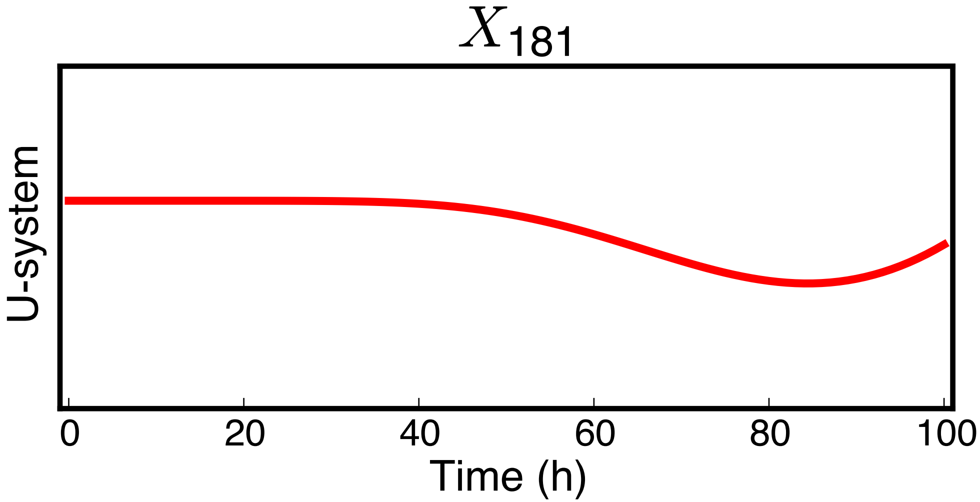
X_{181}
U-system
0
20
40
60
80
100
Time (h)

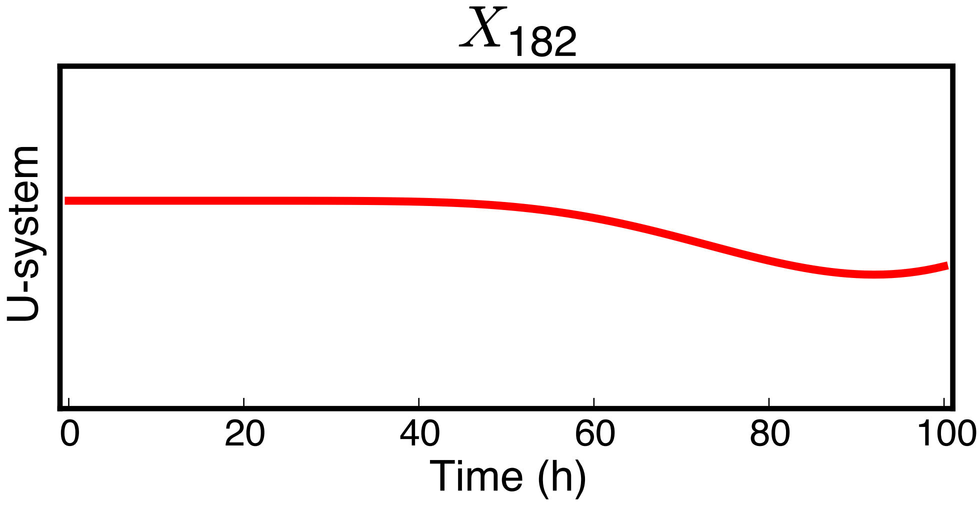
X_{182}
U-system
0
20
40
60
80
100
Time (h)

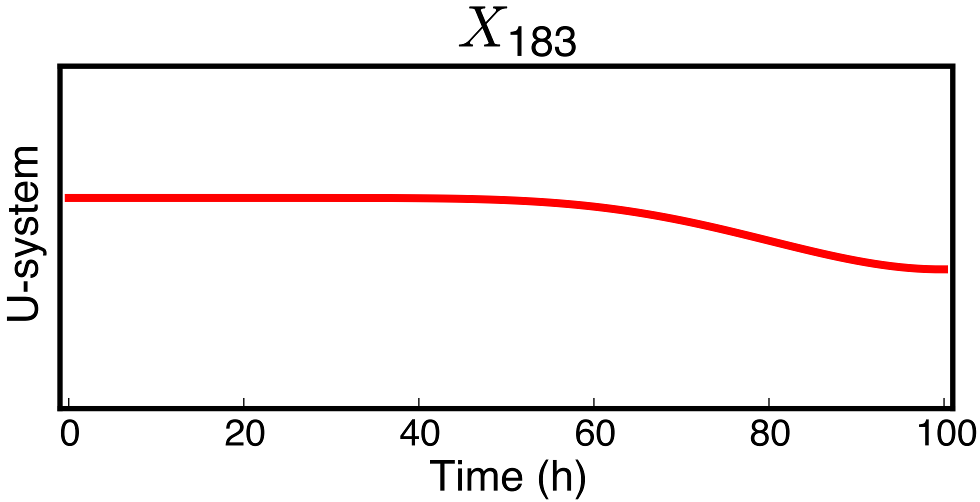

X_{183}
U-system
0
20
40
60
80
100
Time (h)

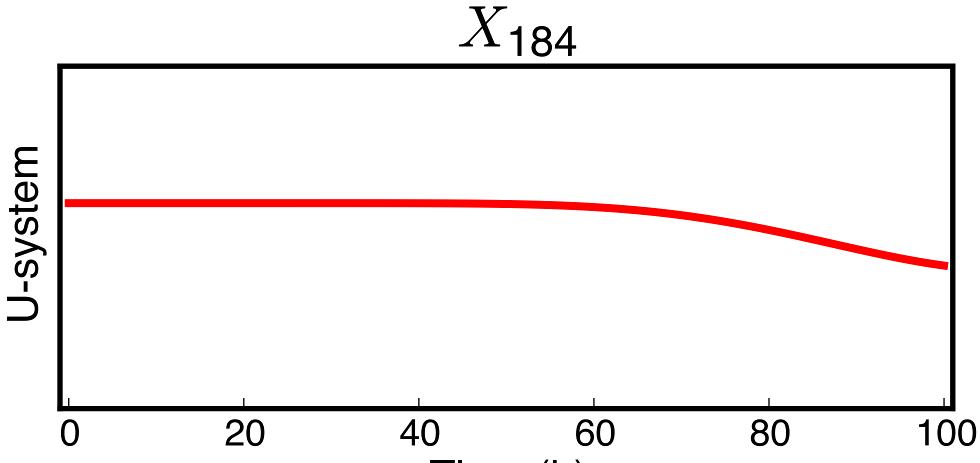
X_{184}
U-system
0
20
40
60
80
100
Time (h)

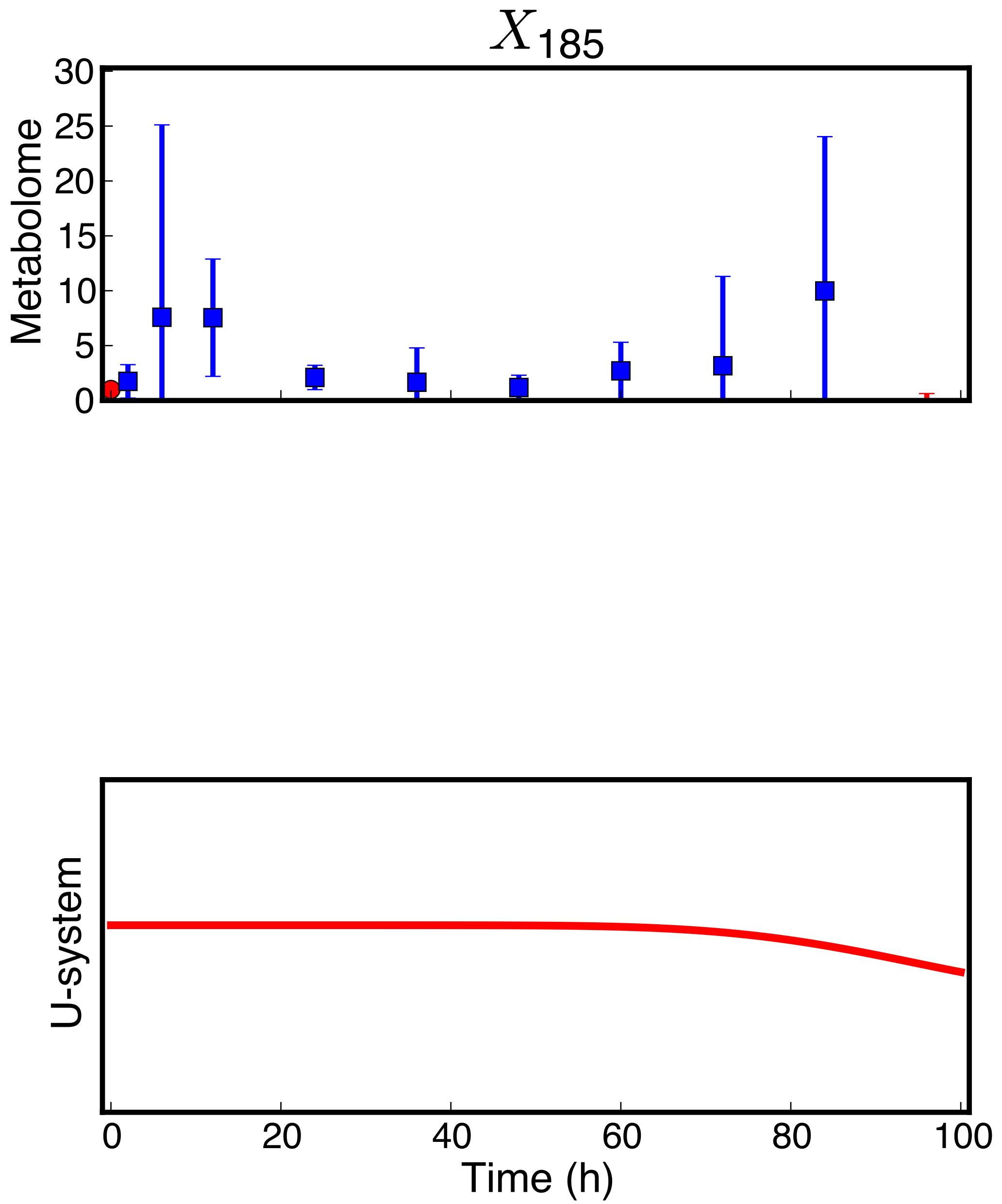

X_{185}
Metabolome
0
5
10
15
20
25
30
U-system
0
20
40
60
80
100
Time (h)

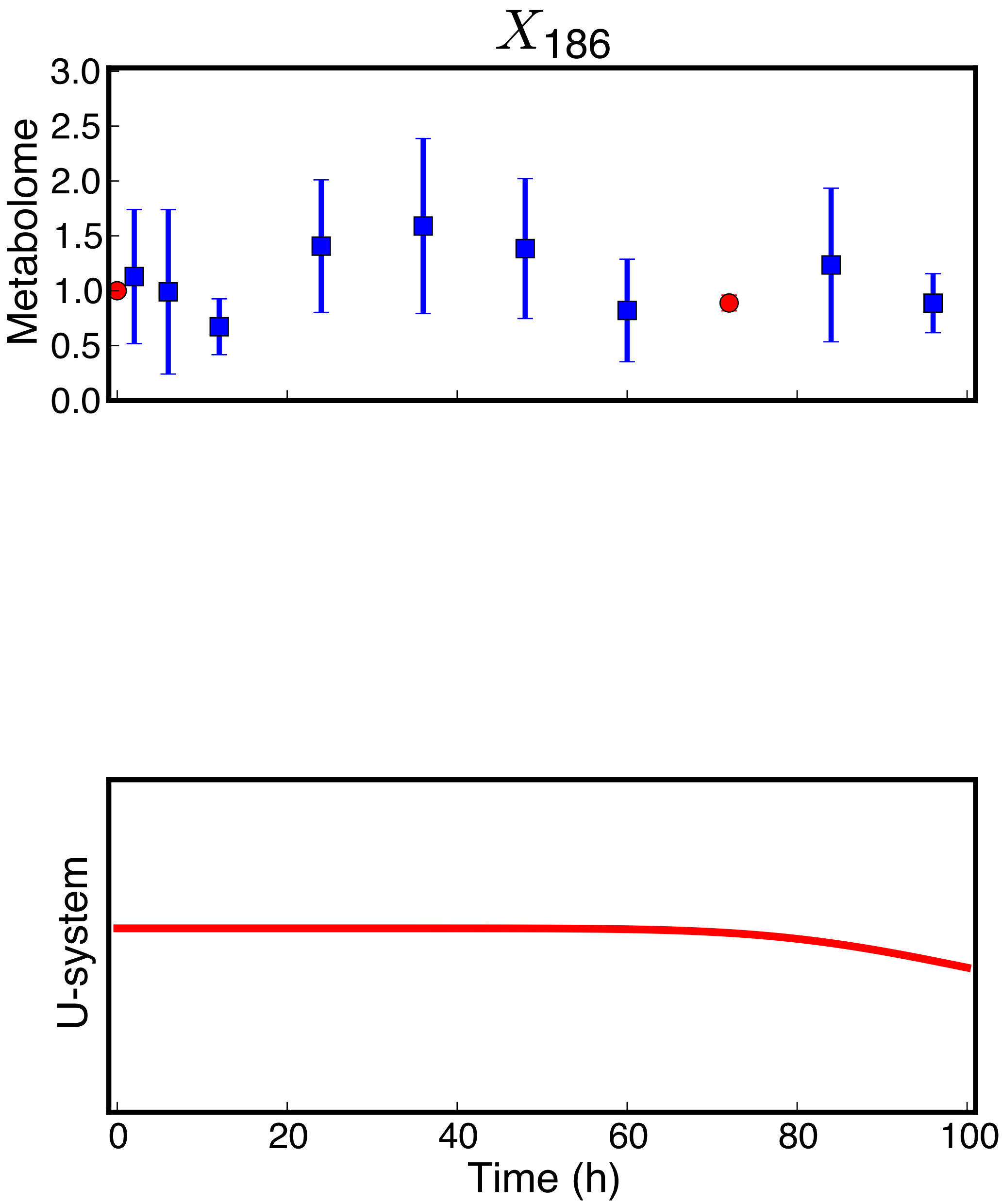

X_{186}
Metabolome
3.0
2.5
2.0
1.5
1.0
0.5
0.0
U-system
0
20
40
60
80
100
Time (h)

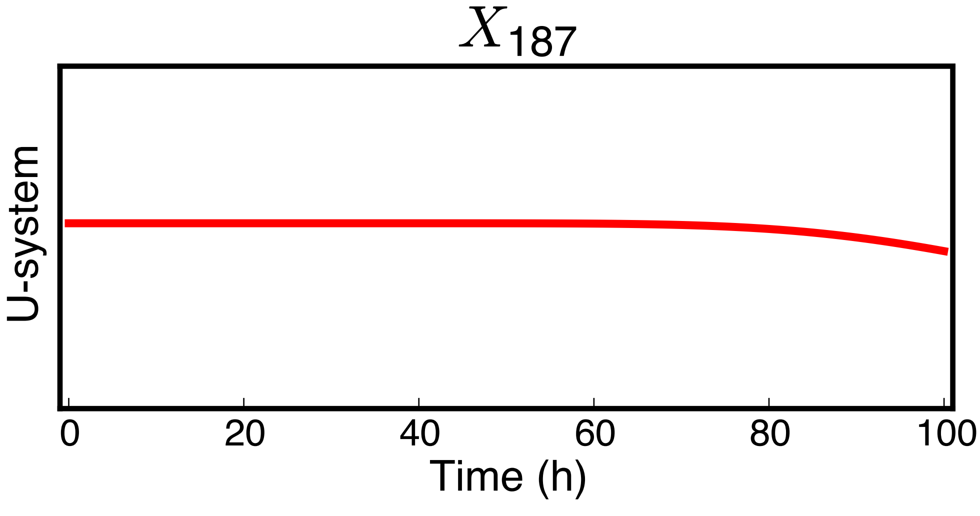

X_{187}
U-system
0
20
40
60
80
100
Time (h)

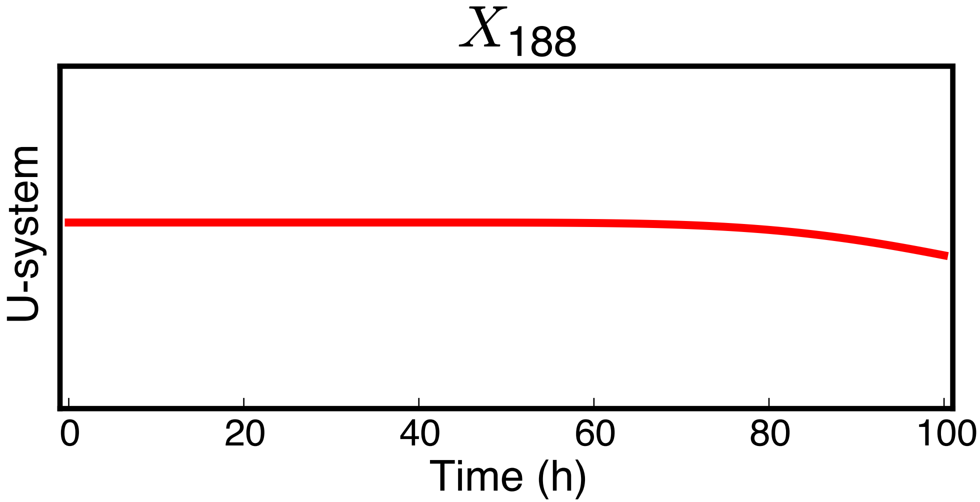

X_{188}
U-system
0
20
40
60
80
100
Time (h)

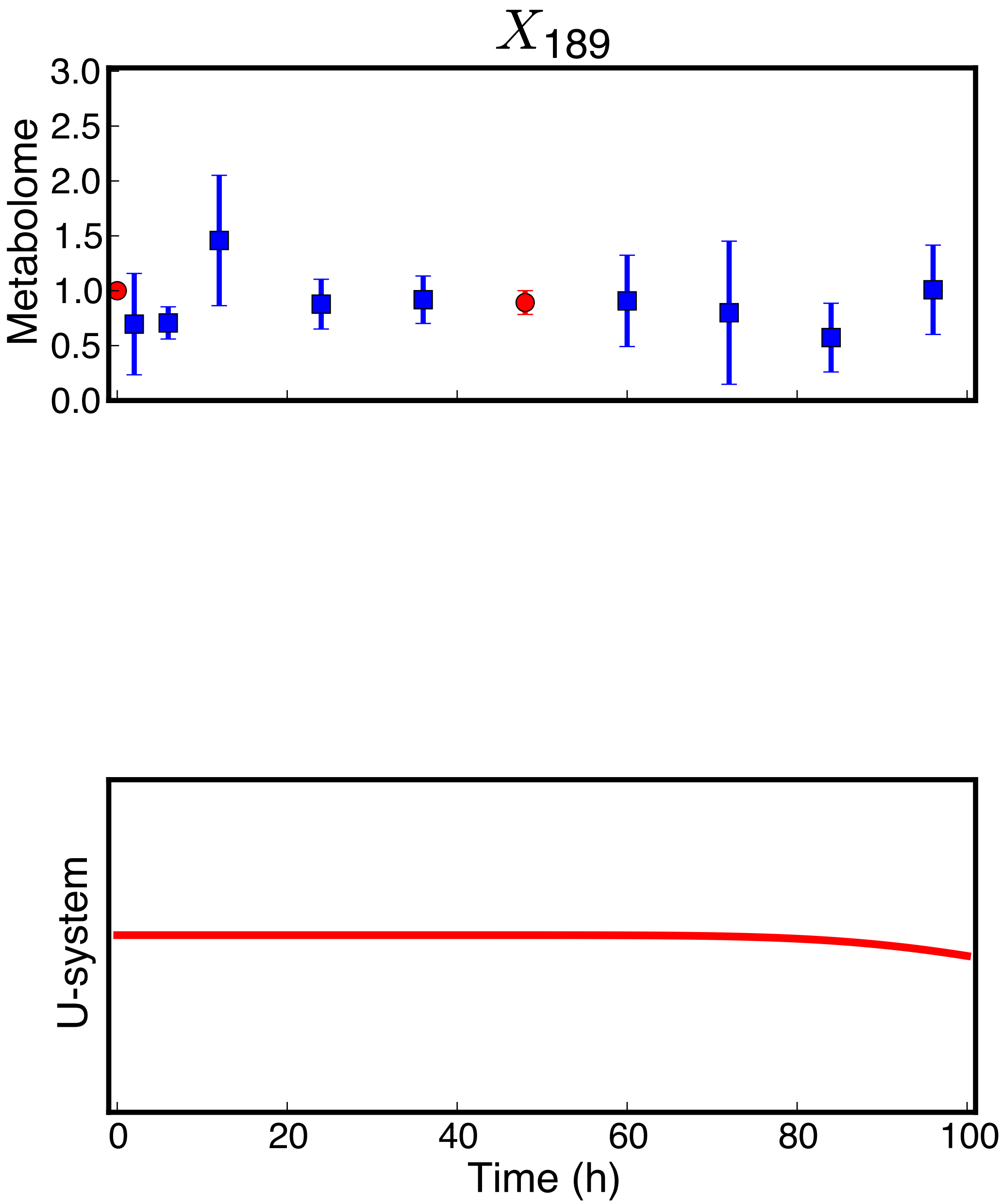

X_{189}
Metabolome
3.0
2.5
2.0
1.5
1.0
0.5
0.0
U-system
0
20
40
60
80
100
Time (h)

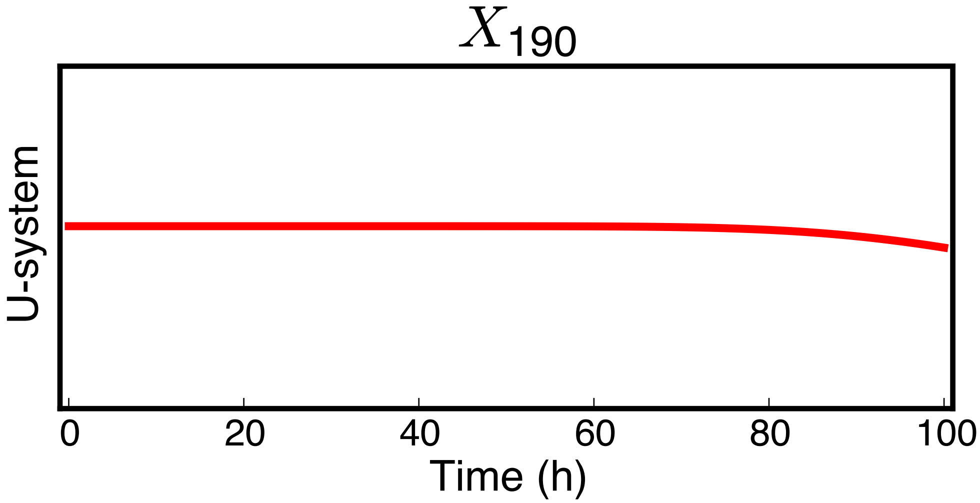

X_{190}
U-system
0
20
40
60
80
100
Time (h)

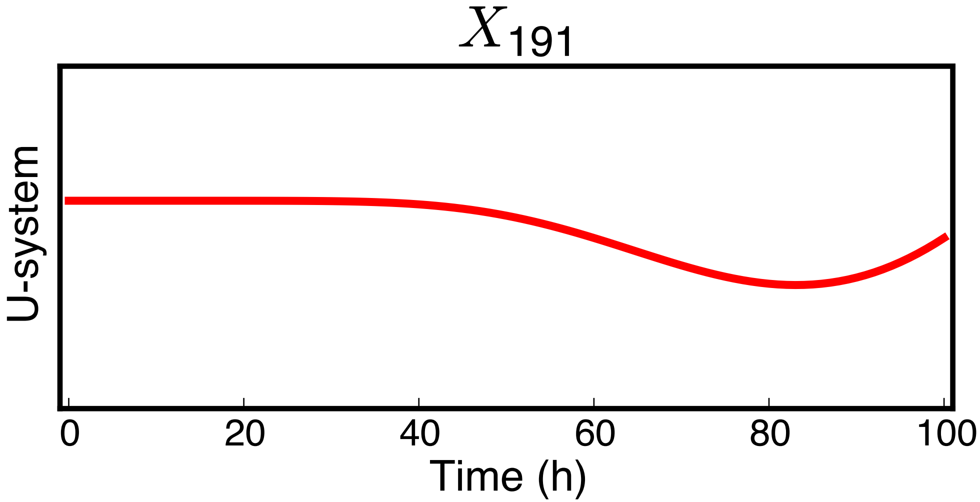

X_{191}
U-system
0
20
40
60
80
100
Time (h)

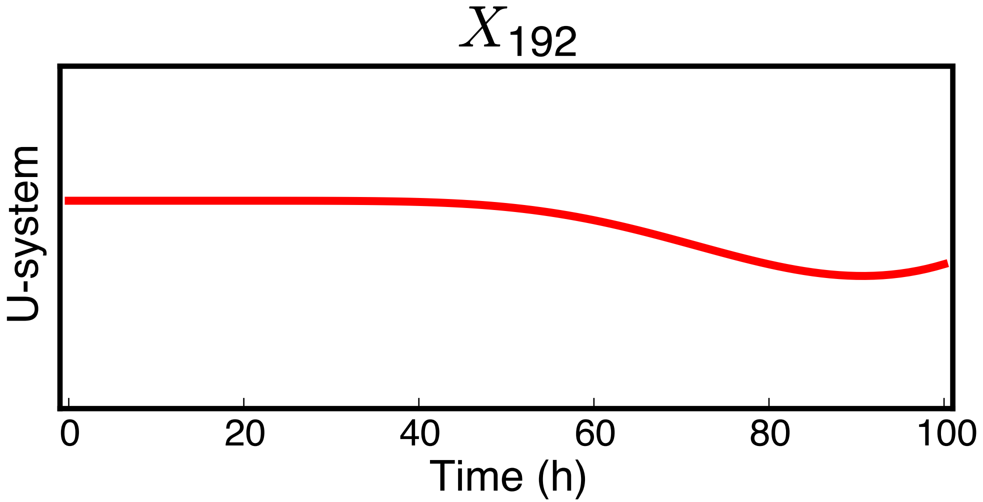

X_{192}
U-system
0
20
40
60
80
100
Time (h)

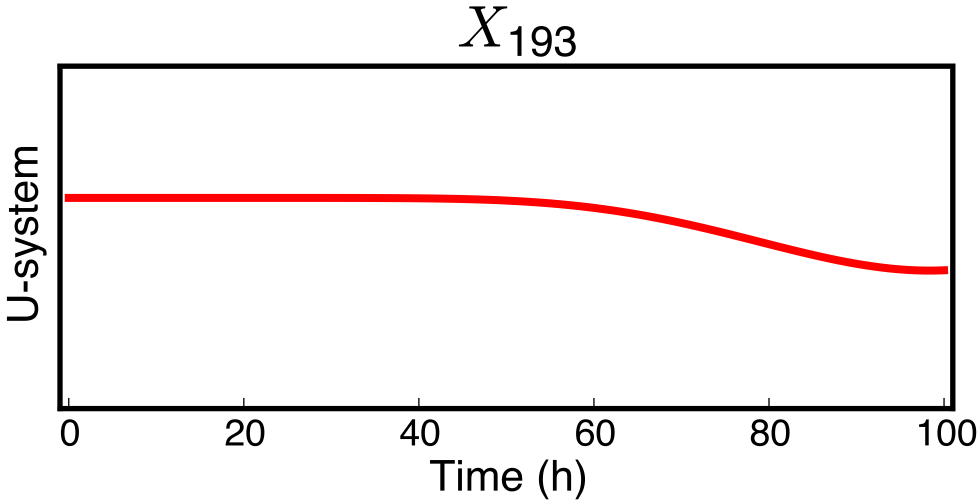

X_{193}
U-system
0
20
40
60
80
100
Time (h)

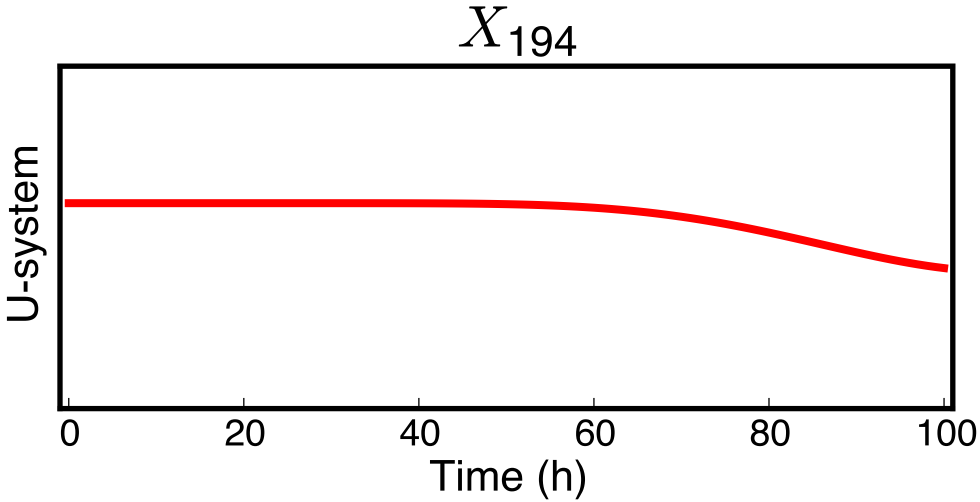
X_{194}
U-system
0
20
40
60
80
100
Time (h)

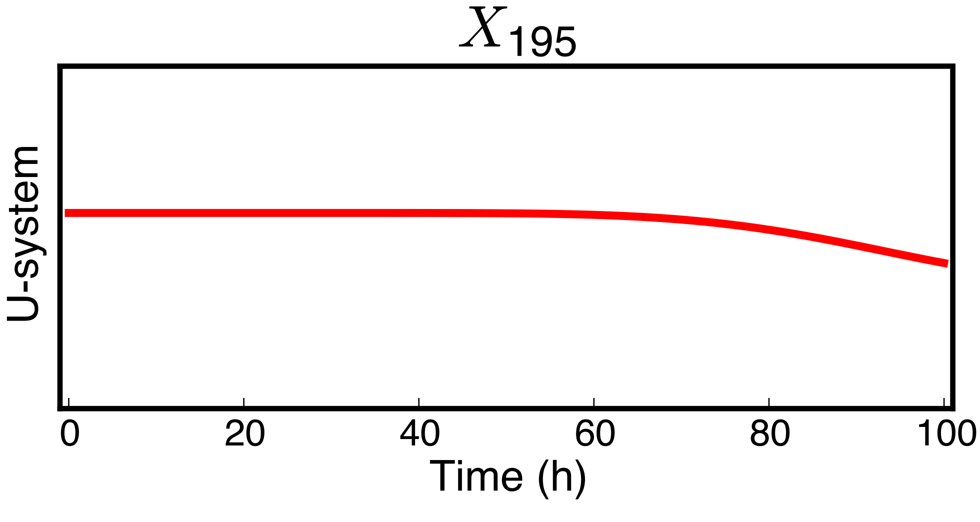

X_{195}
U-system
0
20
40
60
80
100
Time (h)

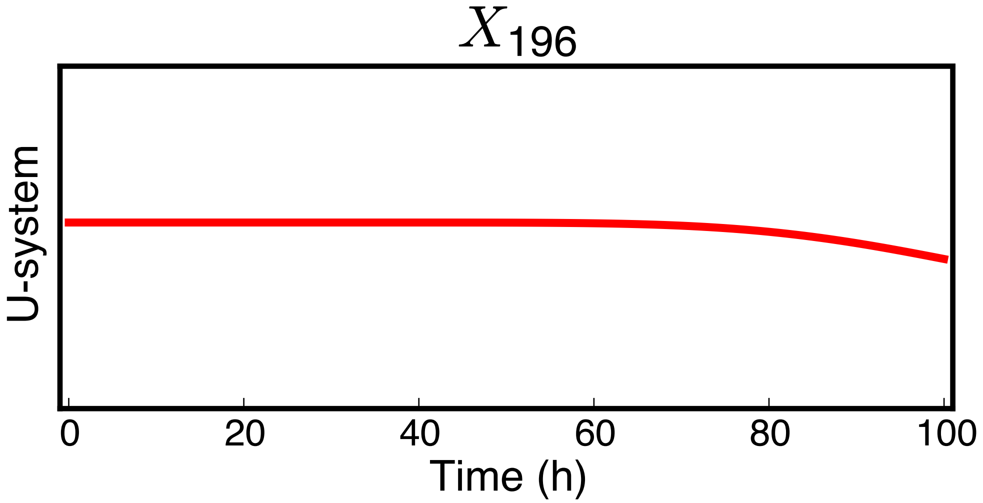

X_{196}
U-system
0
20
40
60
80
100
Time (h)

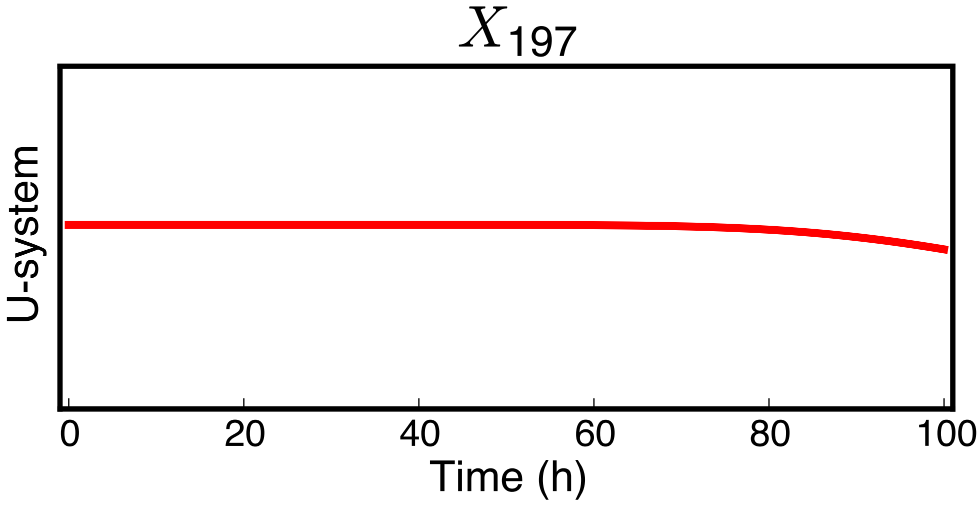
X_{197}
U-system
0
20
40
60
80
100
Time (h)

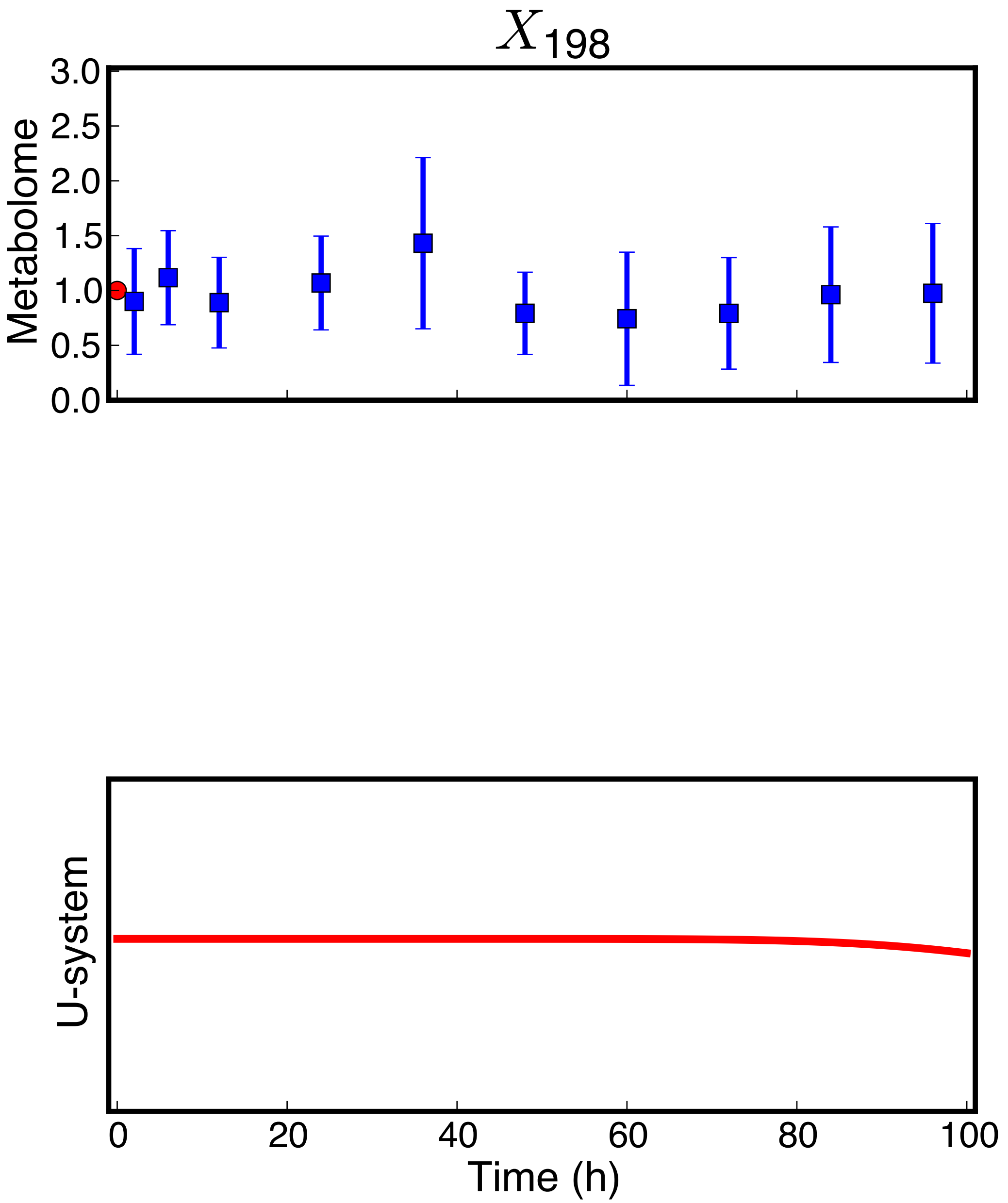

X_{198}
3.0
2.5
2.0
1.5
1.0
0.5
0.0
Metabolome
U-system
0
20
40
60
80
100
Time (h)

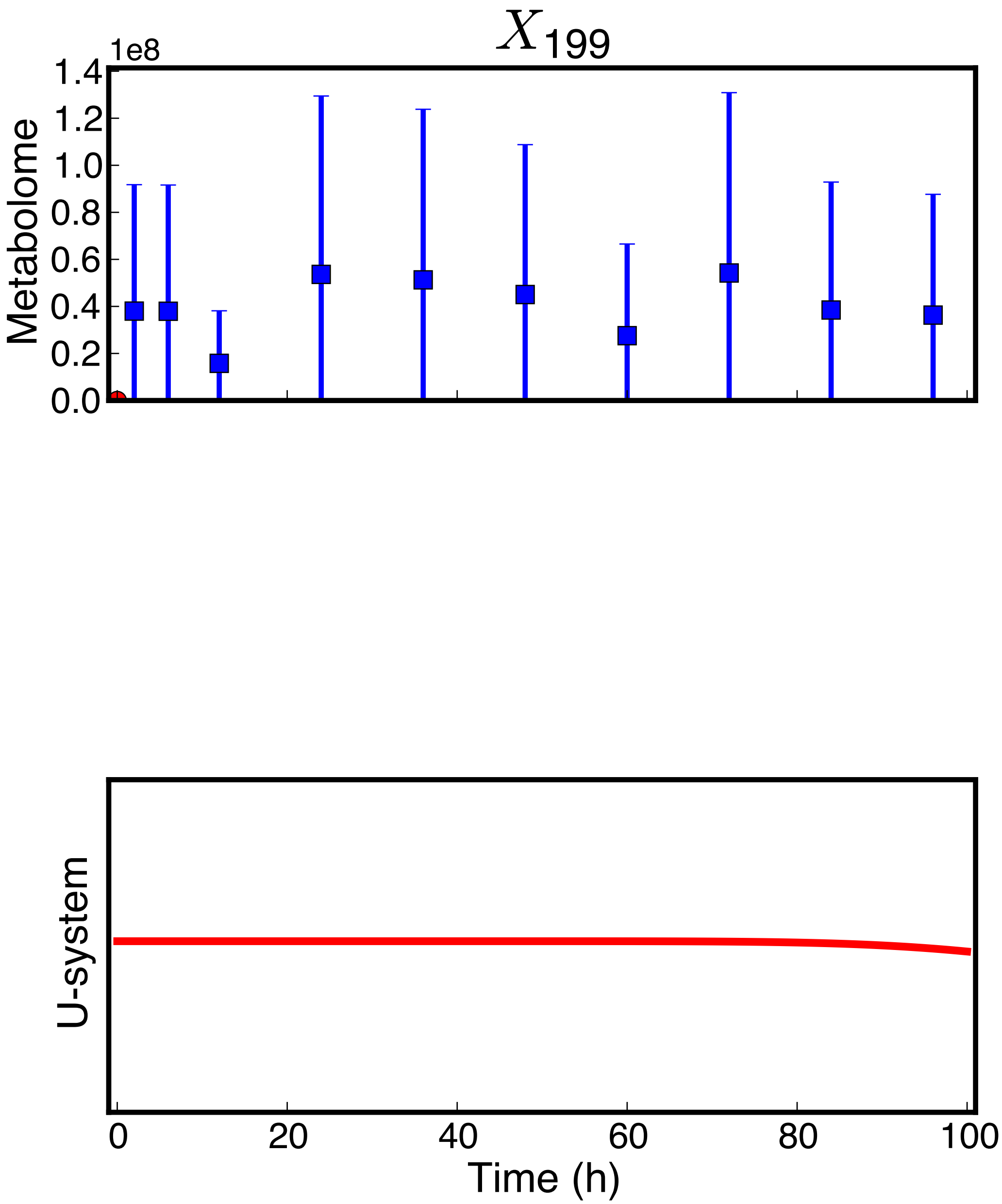

X_{199}
1e8
1.4
1.2
1.0
0.8
0.6
0.4
0.2
0.0
Metabolome
U-system
0
20
40
60
80
100
Time (h)

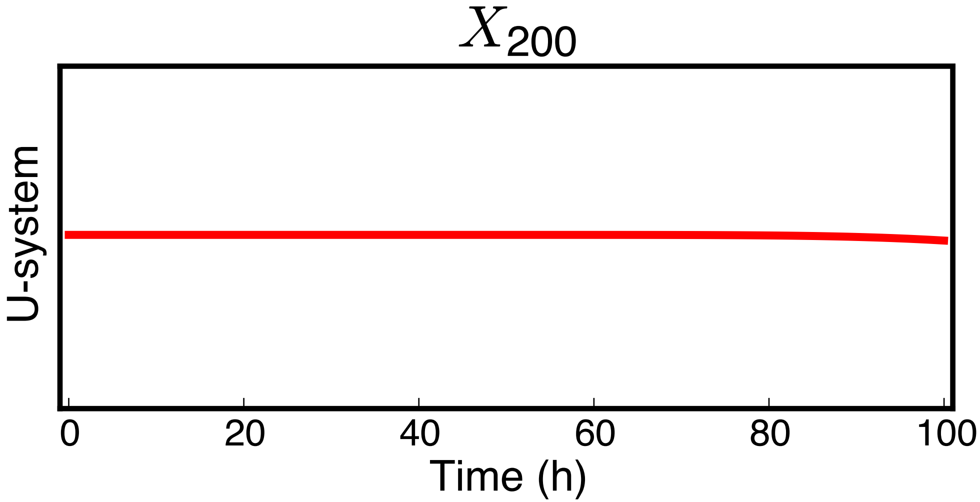

X_{200}
U-system
0
20
40
60
80
100
Time (h)

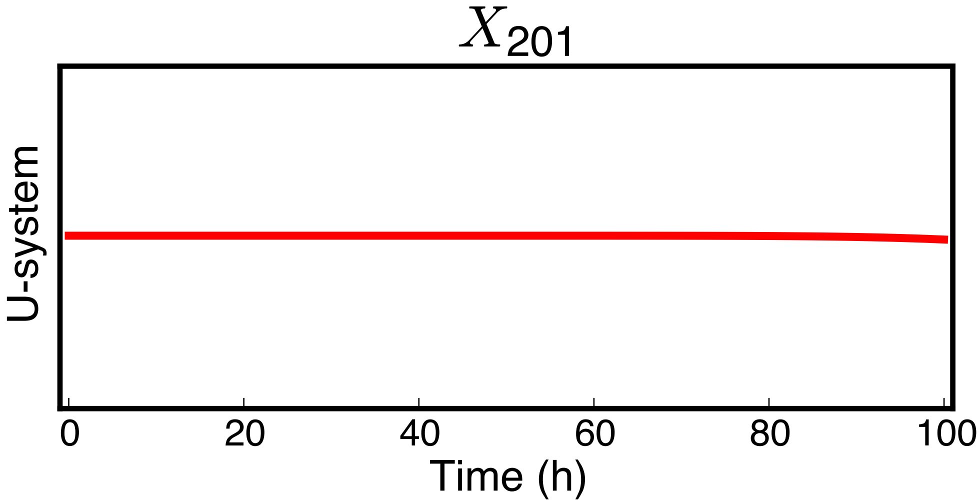
X_{201}
U-system
0
20
40
60
80
100
Time (h)

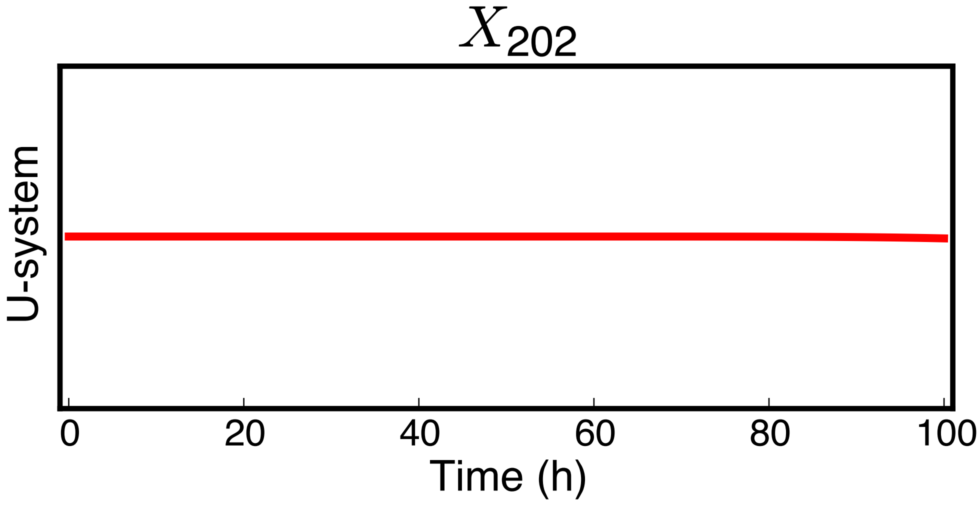

X_{202}
U-system
0
20
40
60
80
100
Time (h)

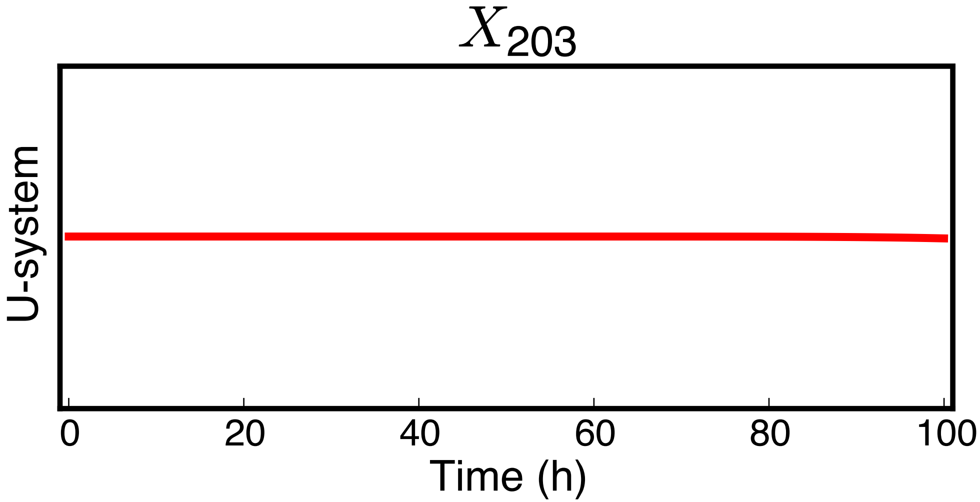

X_{203}
U-system
0
20
40
60
80
100
Time (h)

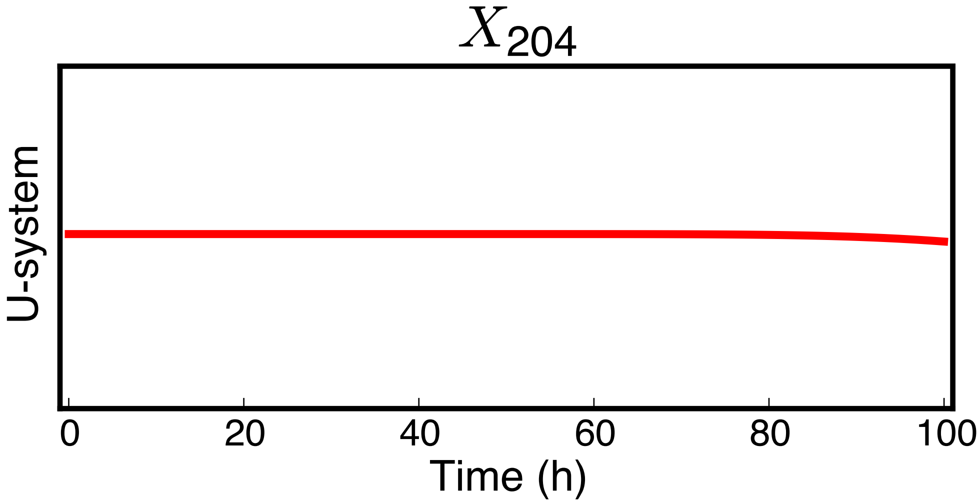

X_{204}
U-system
0
20
40
60
80
100
Time (h)

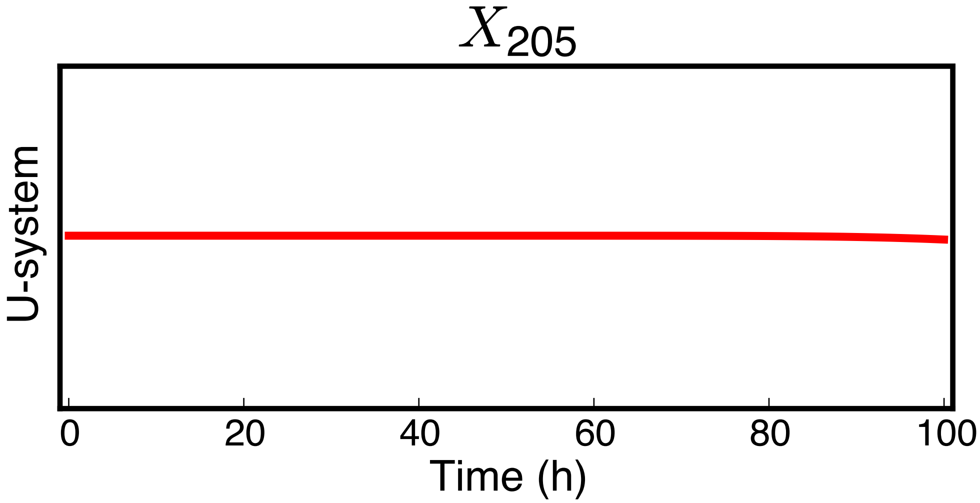
X_{205}
U-system
0
20
40
60
80
100
Time (h)

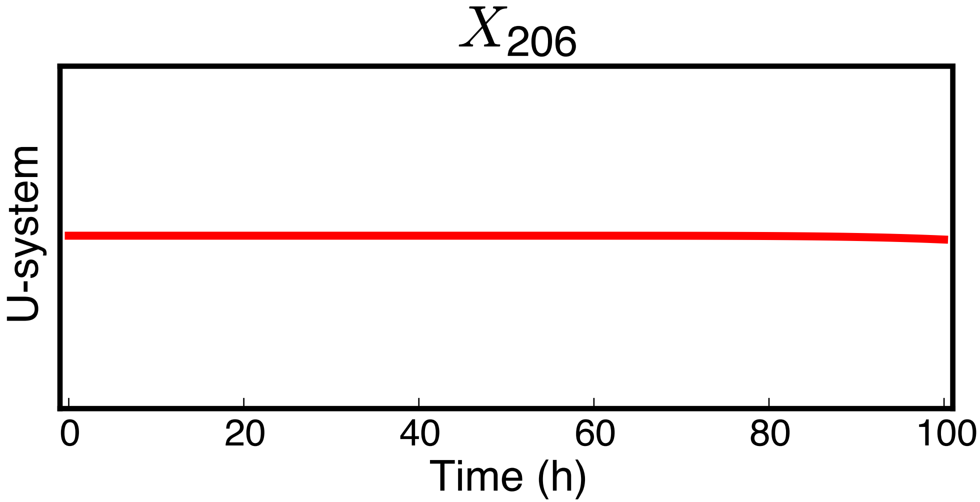
X_{206}
U-system
0
20
40
60
80
100
Time (h)

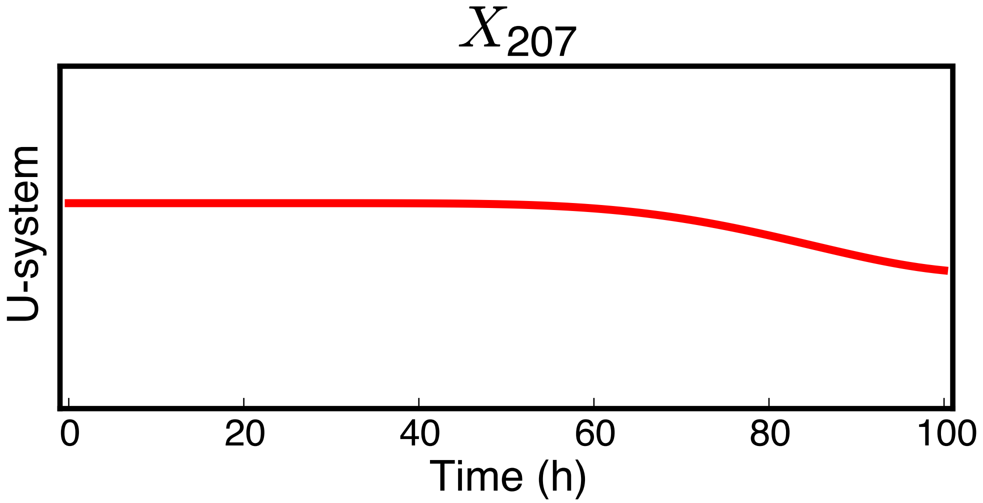
X_{207}
U-system
0
20
40
60
80
100
Time (h)

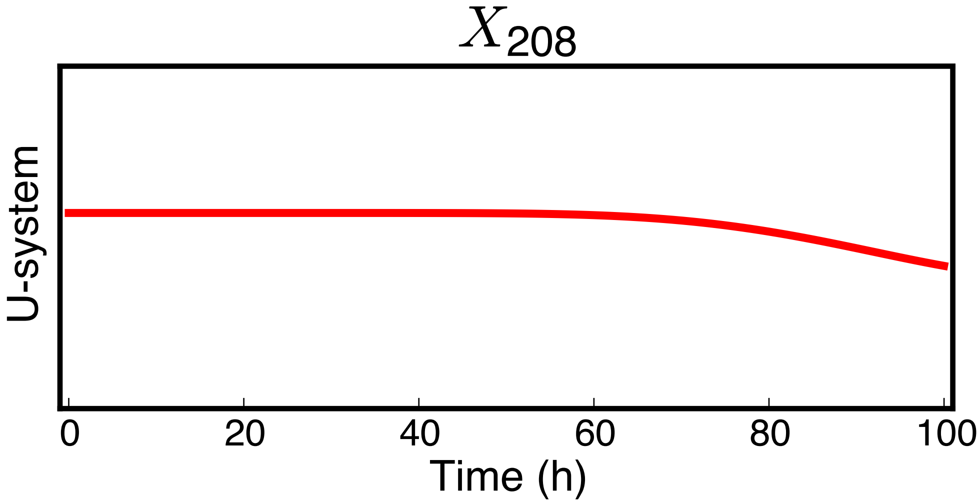

X_{208}
U-system
0
20
40
60
80
100
Time (h)

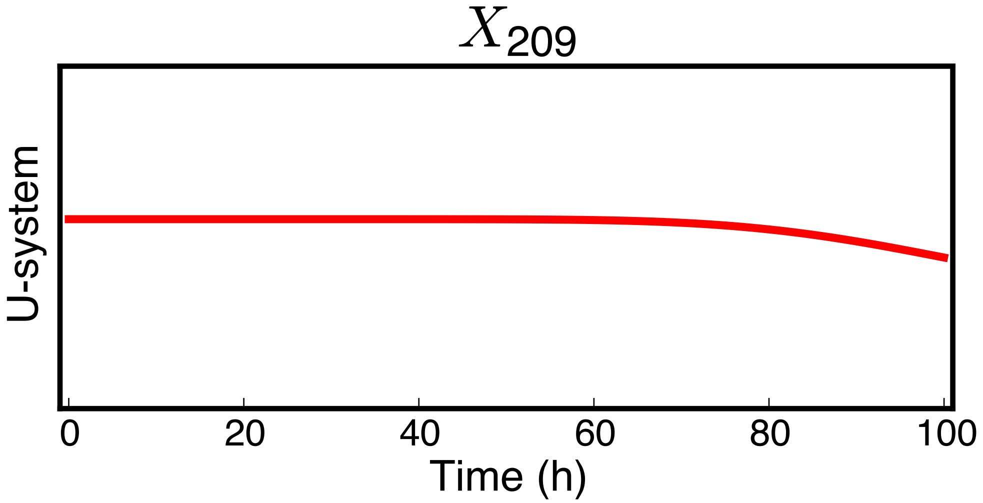

X_{209}
U-system
0
20
40
60
80
100
Time (h)

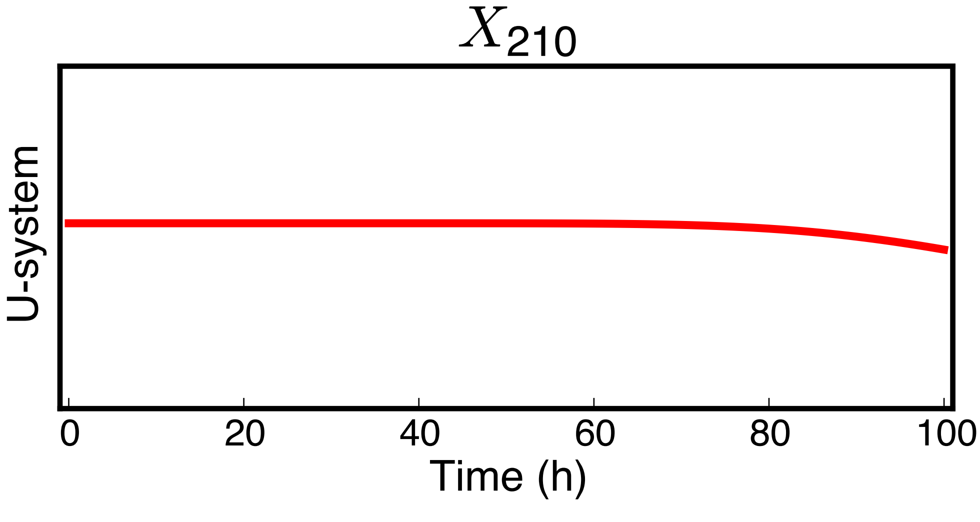
X_{210}
U-system
0
20
40
60
80
100
Time (h)

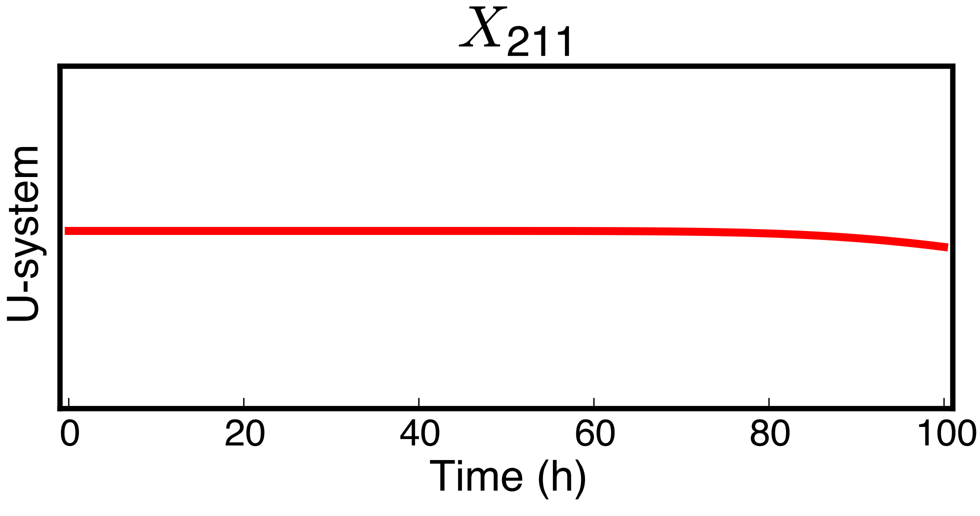

X_{211}
U-system
0
20
40
60
80
100
Time (h)

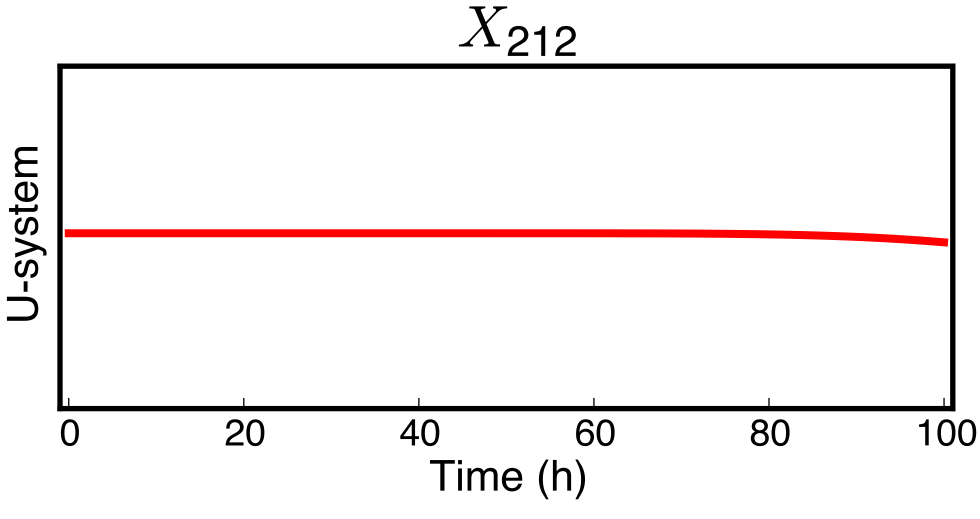
X_{212}
U-system
0
20
40
60
80
100
Time (h)

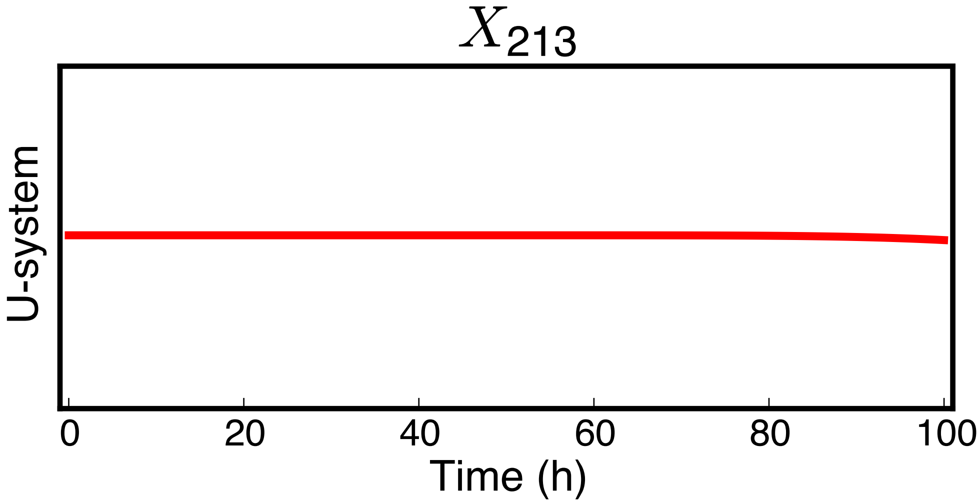

X_{213}
U-system
0
20
40
60
80
100
Time (h)

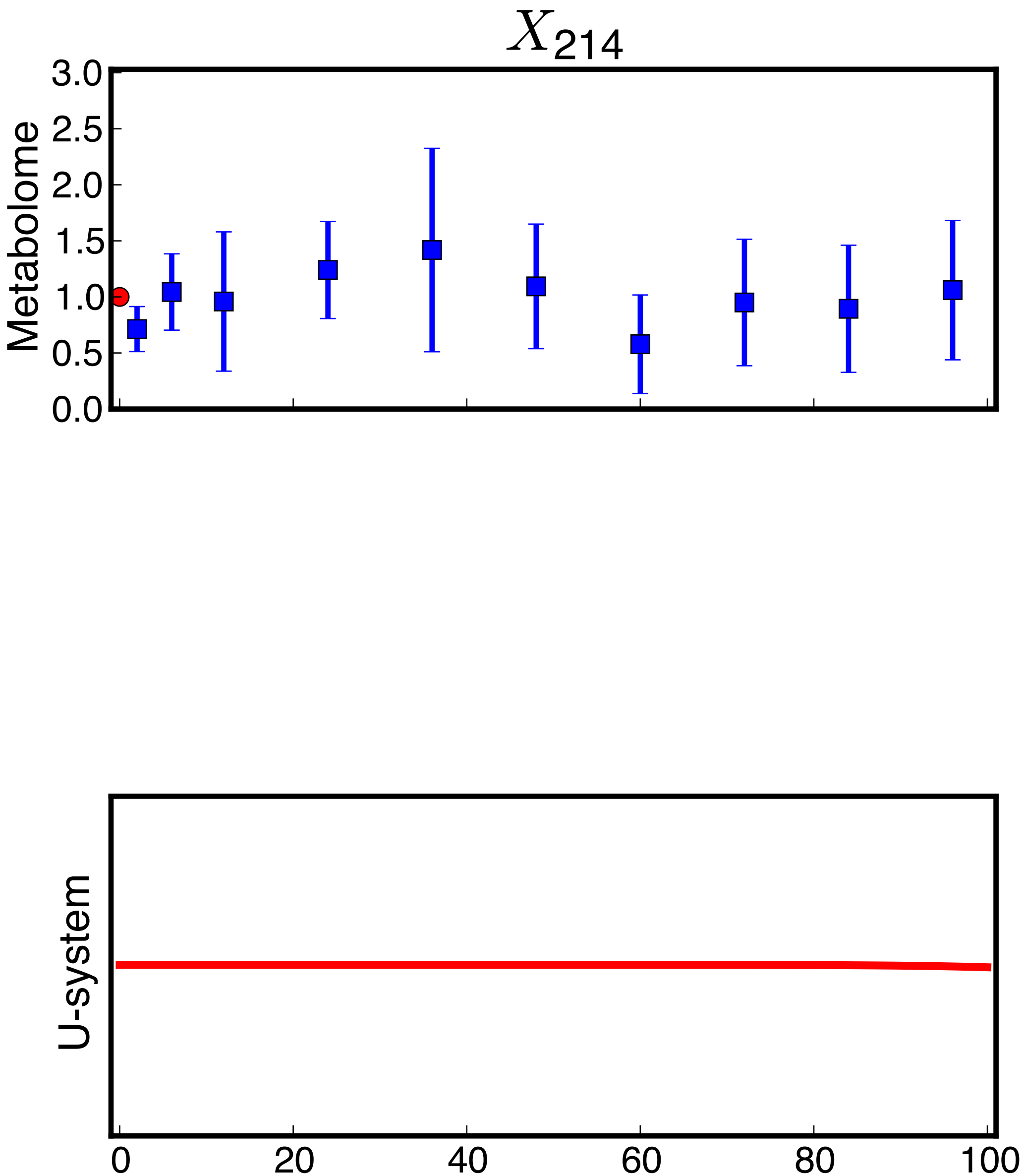
X_{214}
Metabolome
3.0
2.5
2.0
1.5
1.0
0.5
0.0
U-system
0
20
40
60
80
100
Time (h)

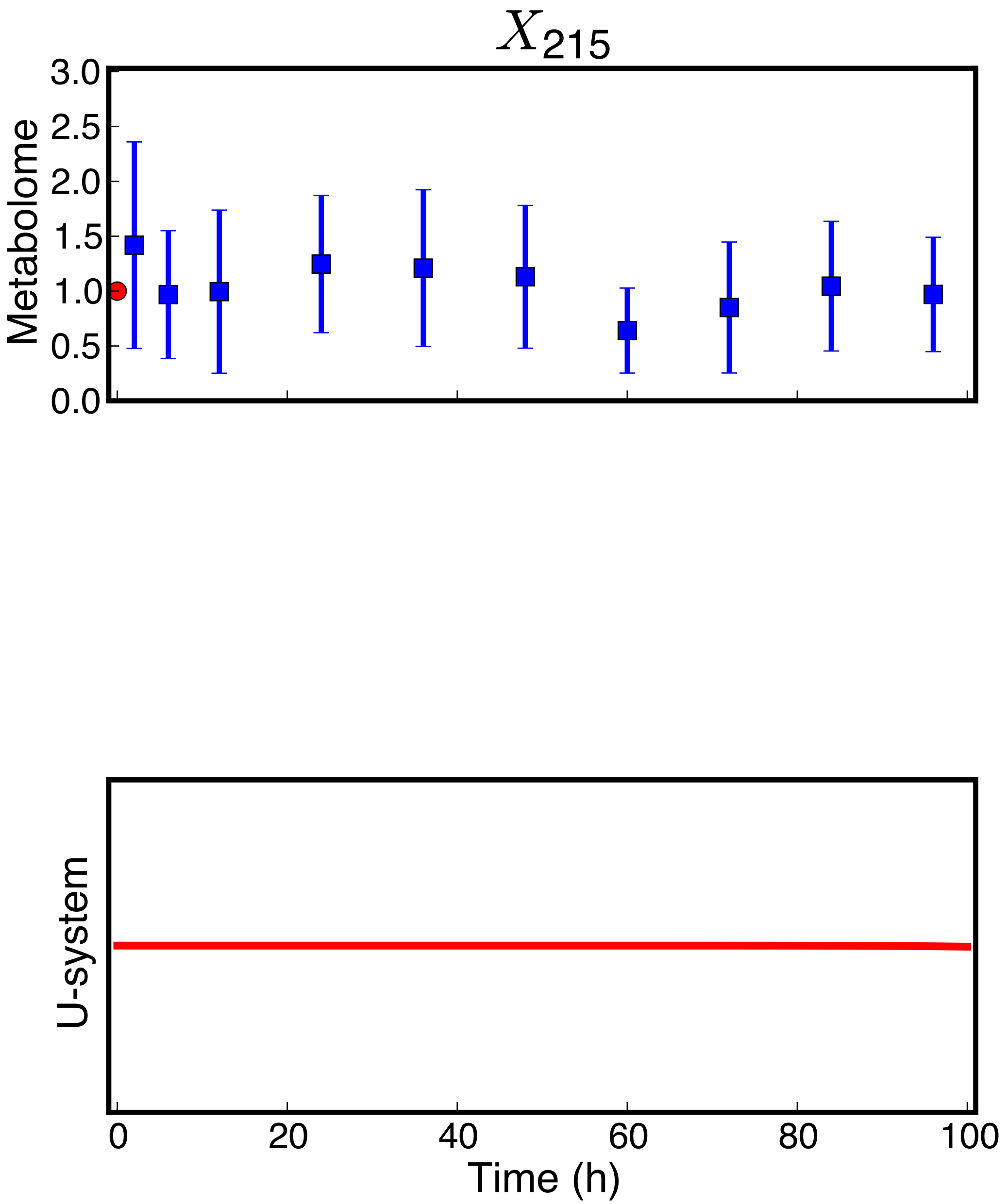
X_{215}
Metabolome
3.0
2.5
2.0
1.5
1.0
0.5
0.0
U-system
0
20
40
60
80
100
Time (h)

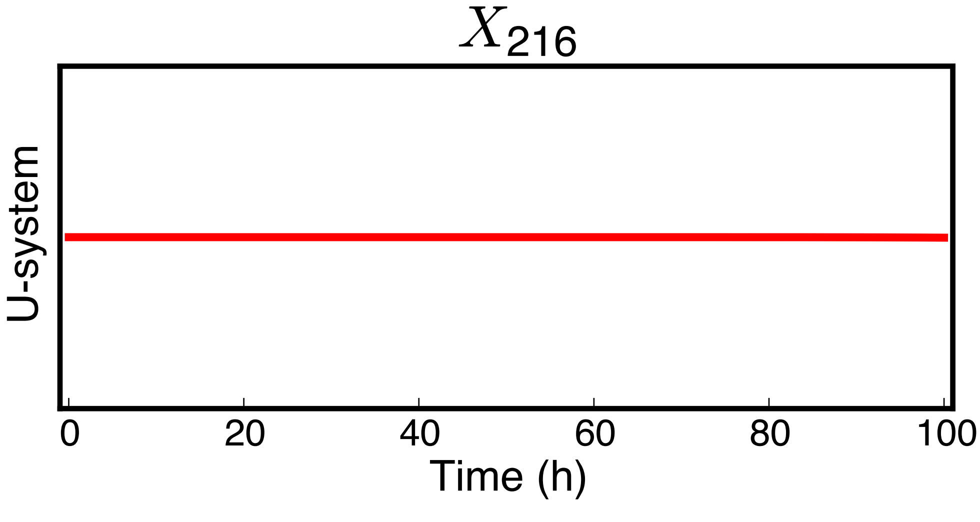

X_{216}
U-system
0
20
40
60
80
100
Time (h)

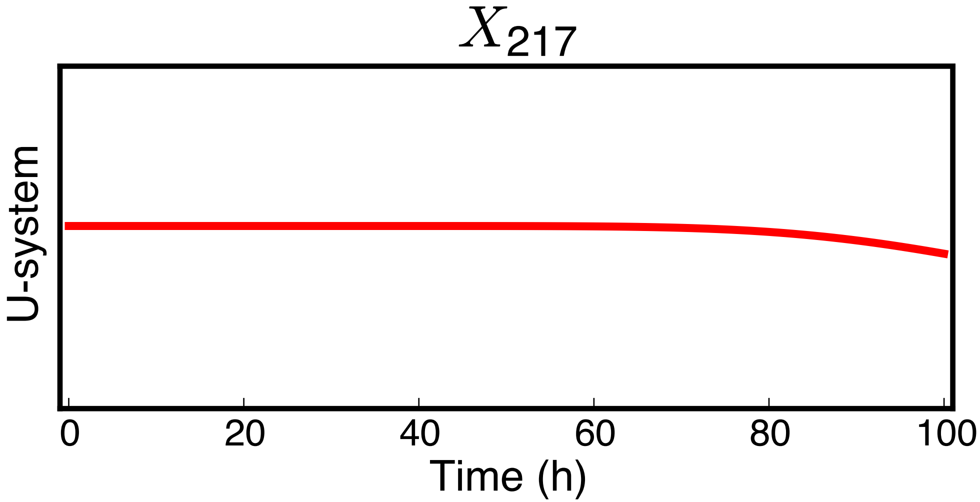

X_{217}
U-system
0
20
40
60
80
100
Time (h)

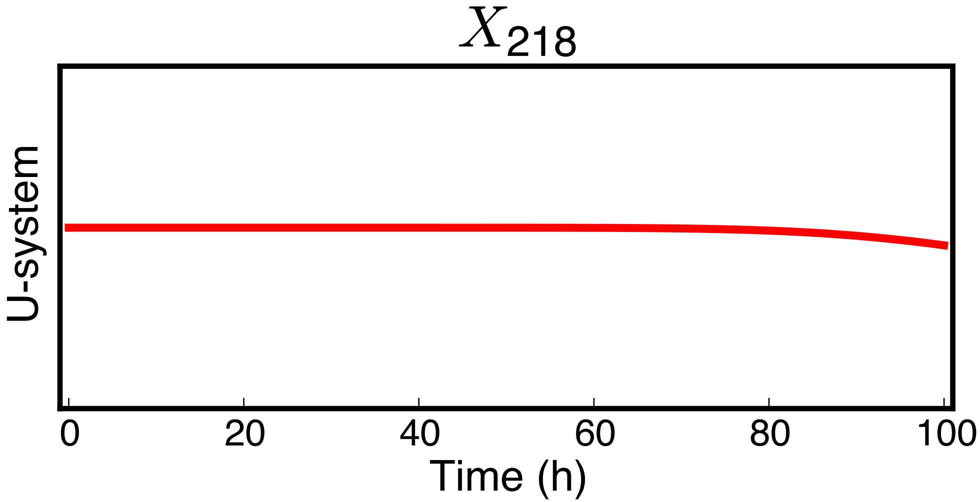

X_{218}
U-system
0
20
40
60
80
100
Time (h)

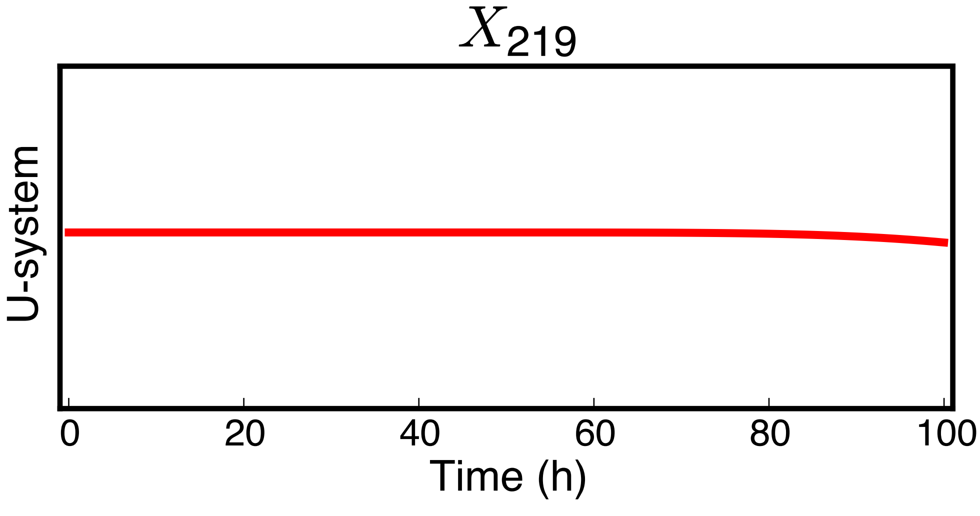
X_{219}
U-system
0
20
40
60
80
100
Time (h)

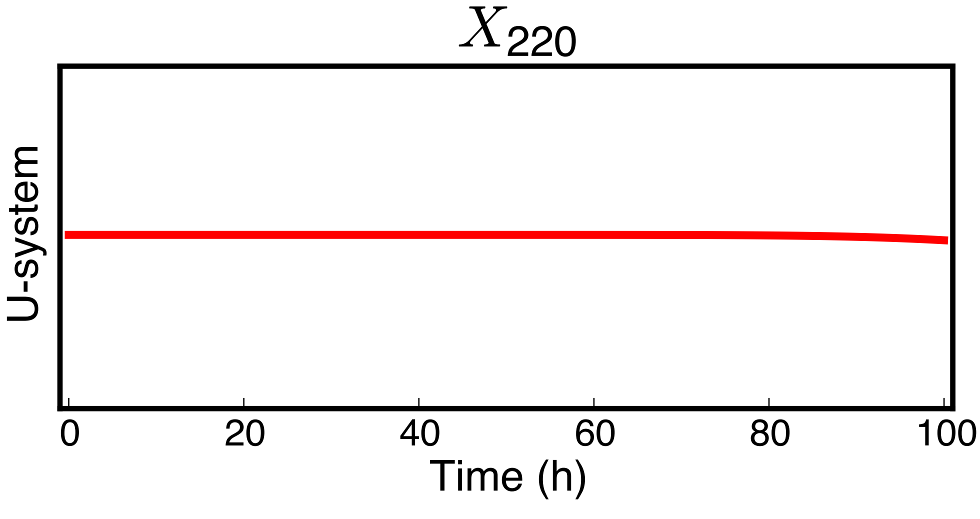

X_{220}
U-system
0
20
40
60
80
100
Time (h)

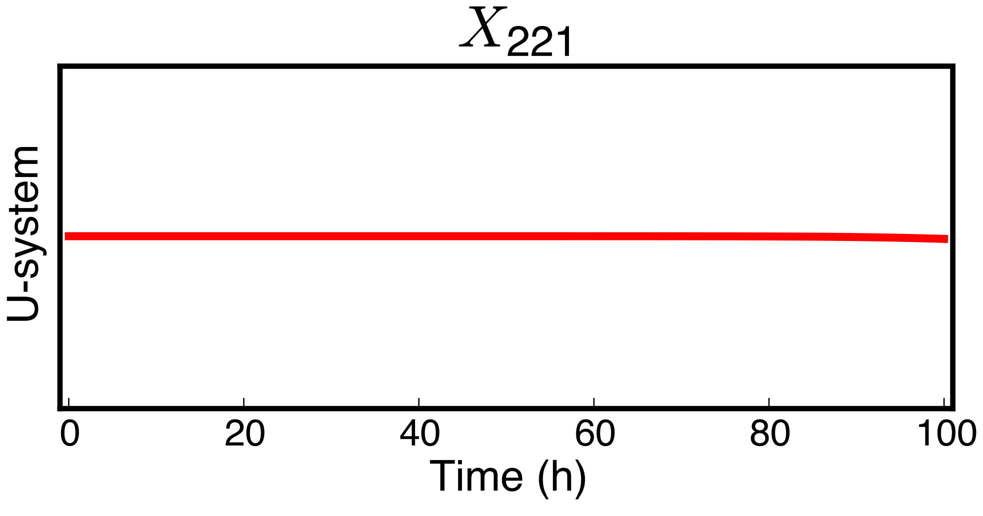

X_{221}
U-system
0
20
40
60
80
100
Time (h)

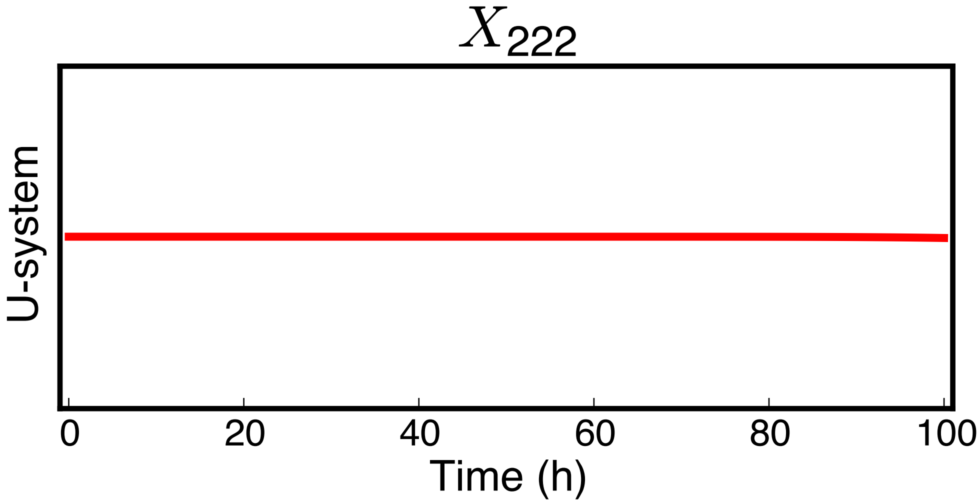

X_{222}
U-system
0
20
40
60
80
100
Time (h)

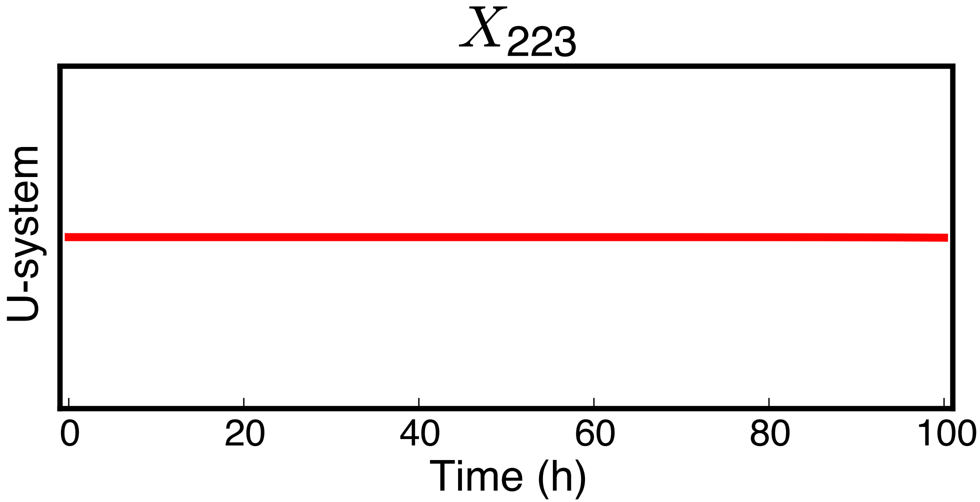
X_{223}
U-system
0
20
40
60
80
100
Time (h)

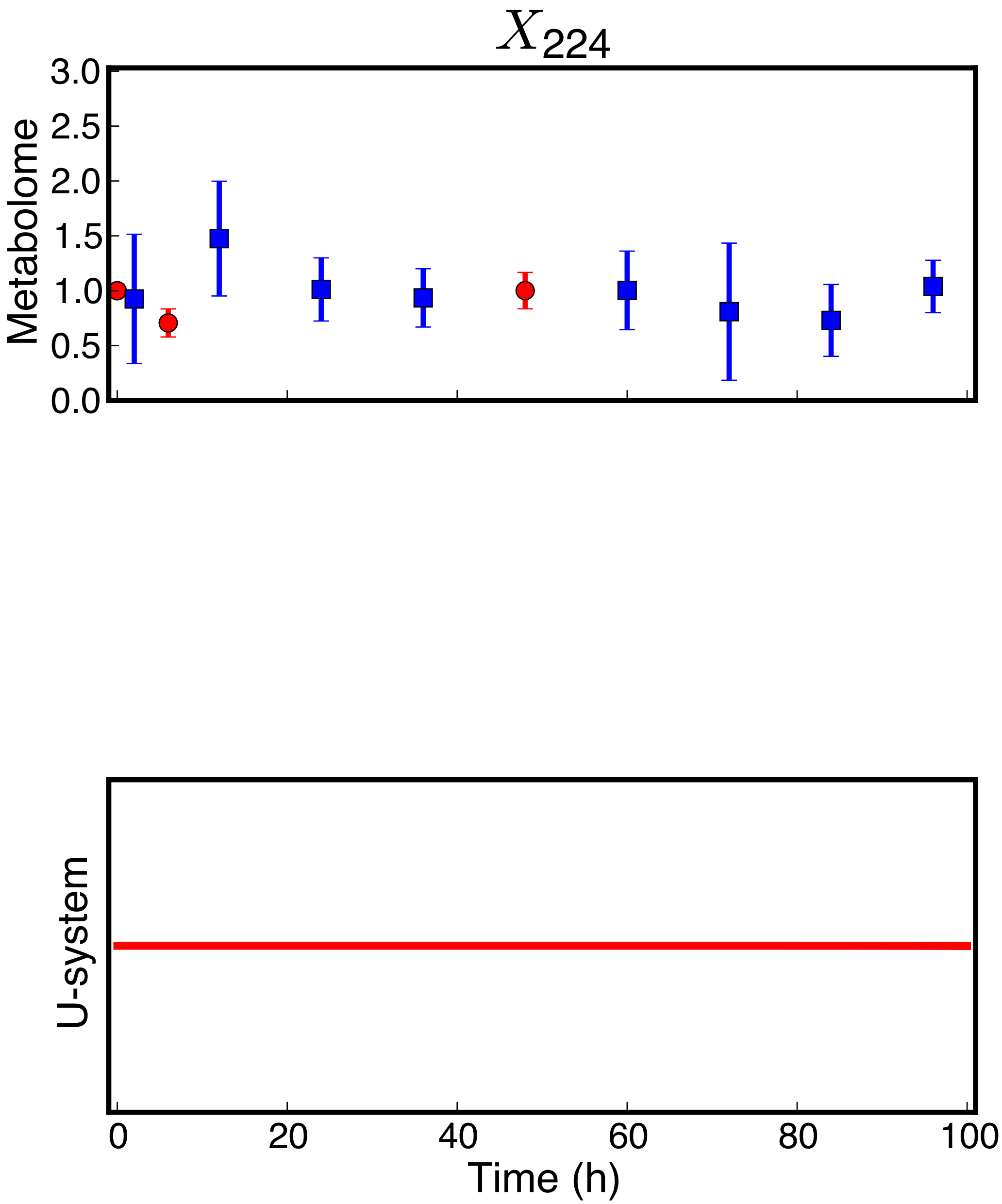

X_{224}
Metabolome
3.0
2.5
2.0
1.5
1.0
0.5
0.0
U-system
0
20
40
60
80
100
Time (h)

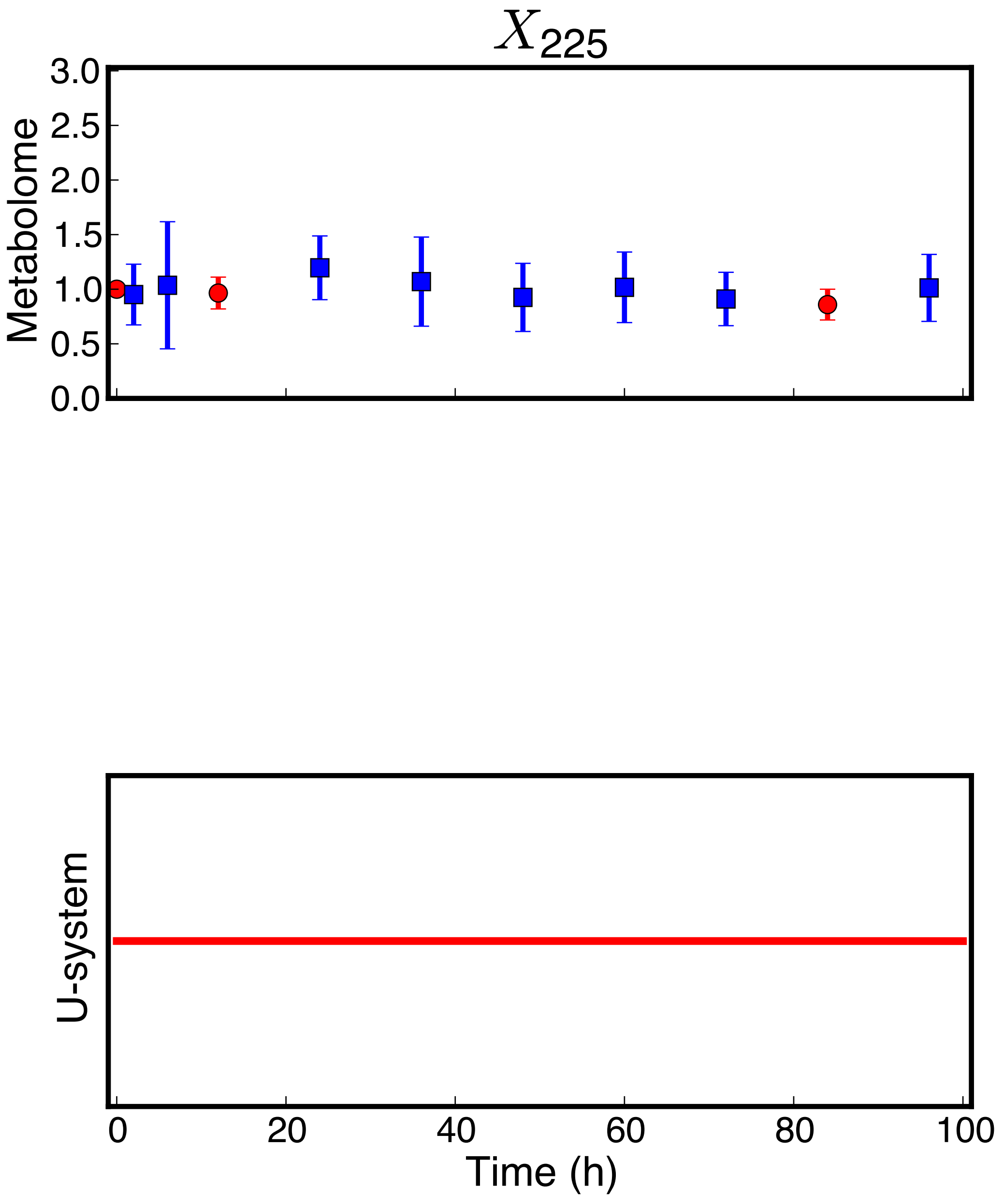

X_{225}
Metabolome
3.0
2.5
2.0
1.5
1.0
0.5
0.0
U-system
0
20
40
60
80
100
Time (h)

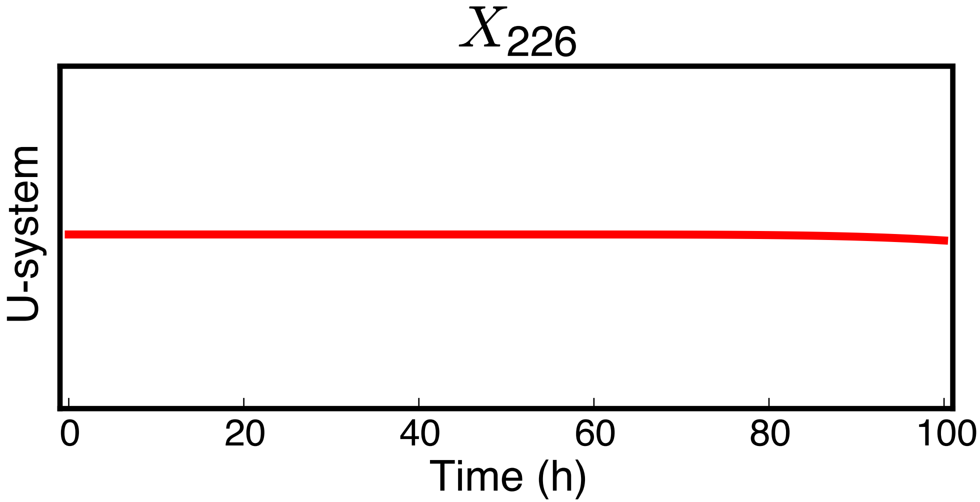
X_{226}
U-system
0
20
40
60
80
100
Time (h)

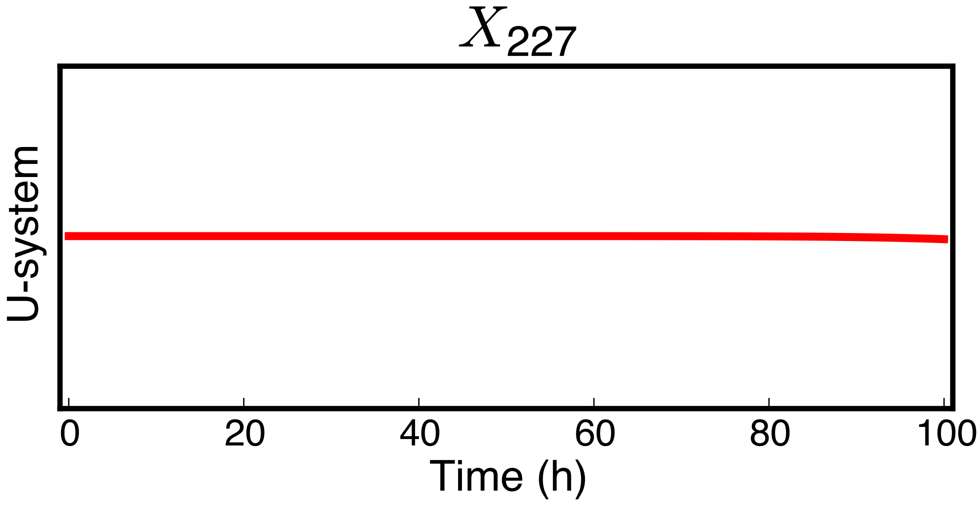
X_{227}
U-system
0
20
40
60
80
100
Time (h)

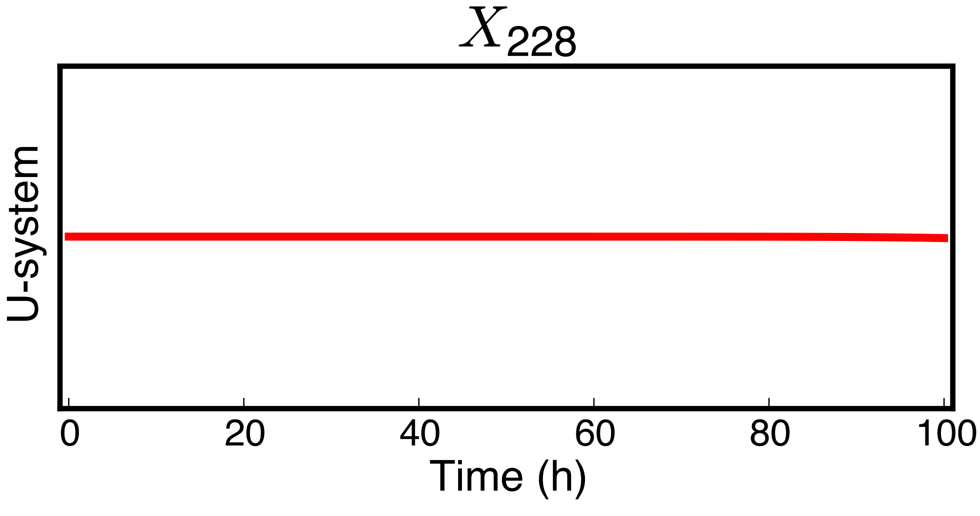
X_{228}
U-system
0
20
40
60
80
100
Time (h)

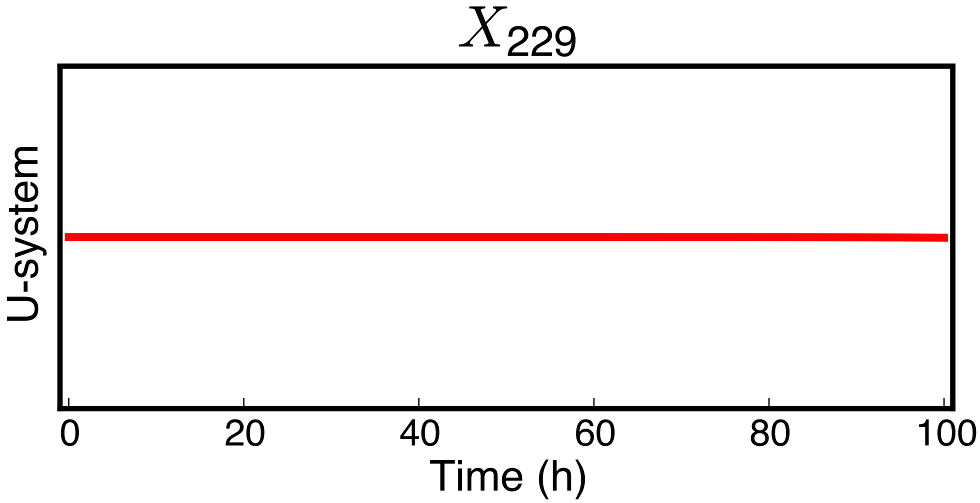

X_{229}
U-system
0
20
40
60
80
100
Time (h)

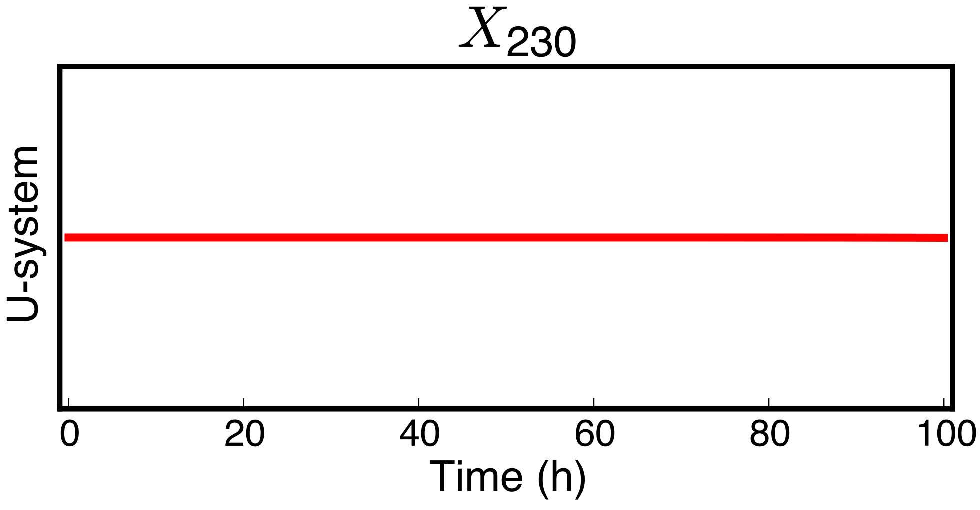

X_{230}
U-system
0
20
40
60
80
100
Time (h)

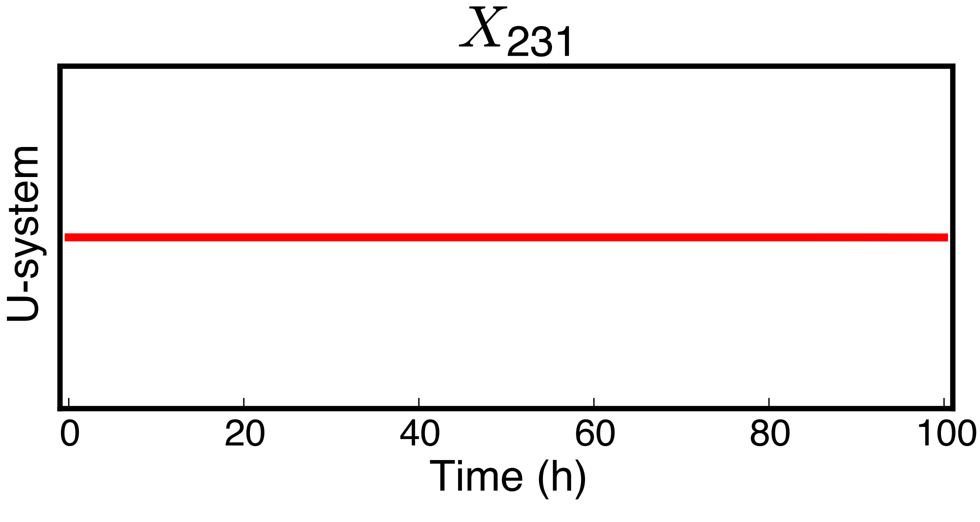

X_{231}
U-system
0
20
40
60
80
100
Time (h)

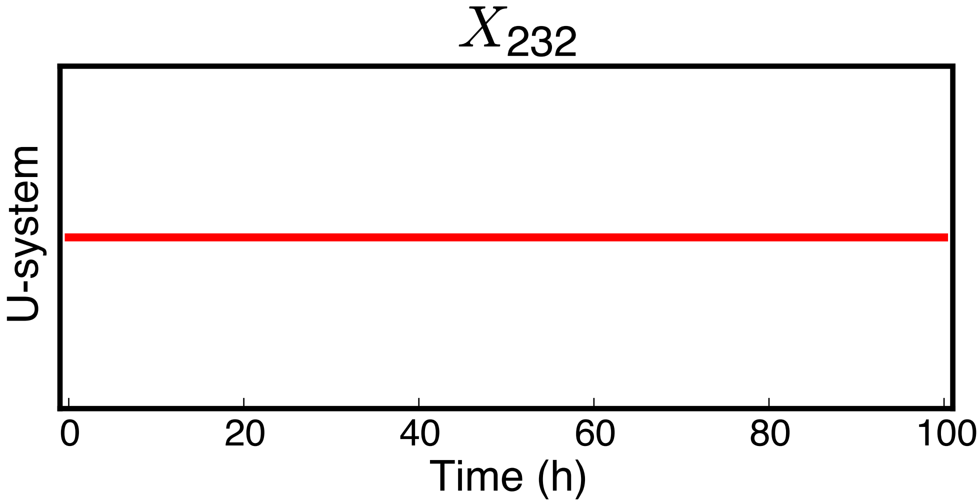
X_{232}
U-system
0
20
40
60
80
100
Time (h)

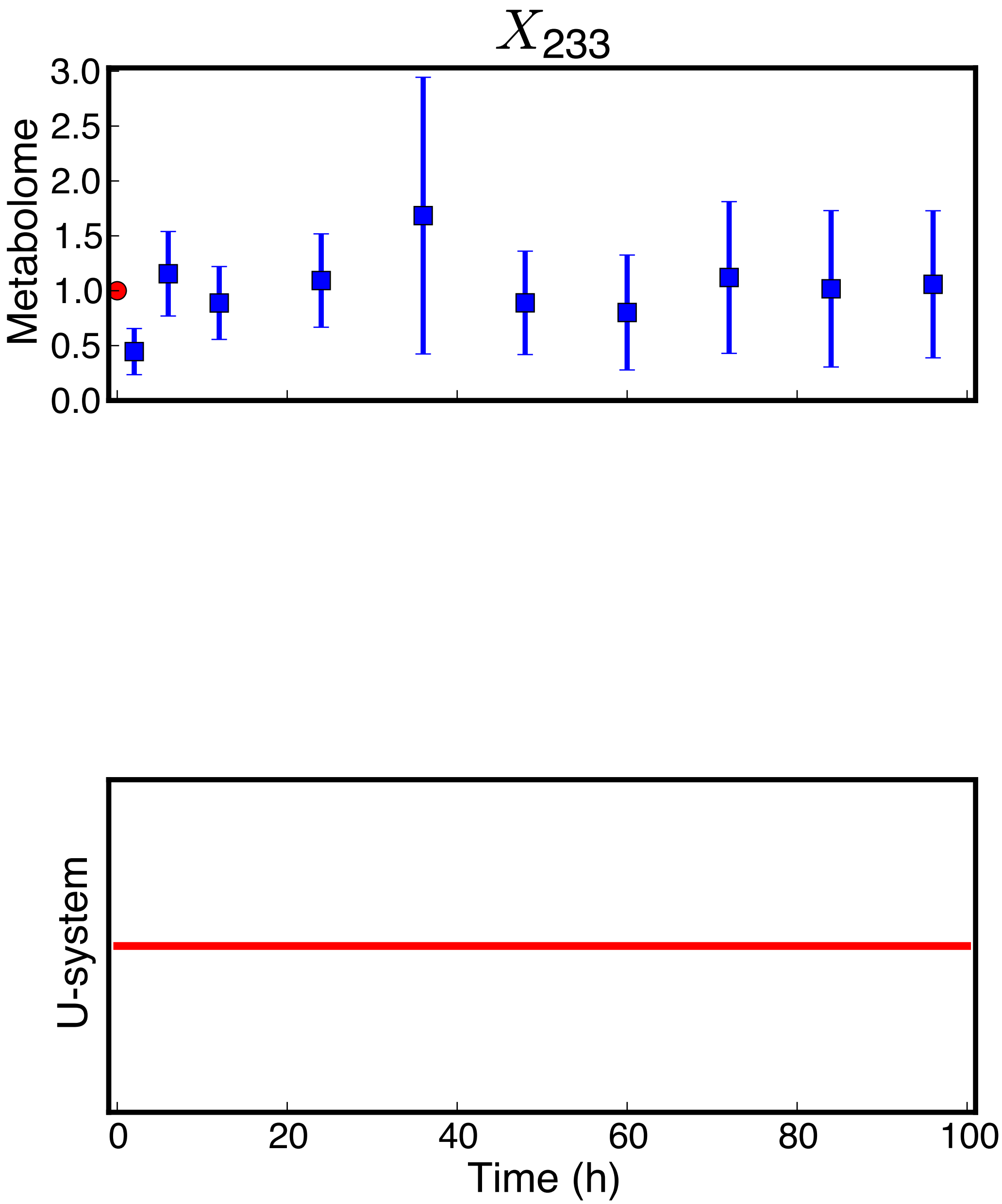

X_{233}
Metabolome
3.0
2.5
2.0
1.5
1.0
0.5
0.0
U-system
0
20
40
60
80
100
Time (h)

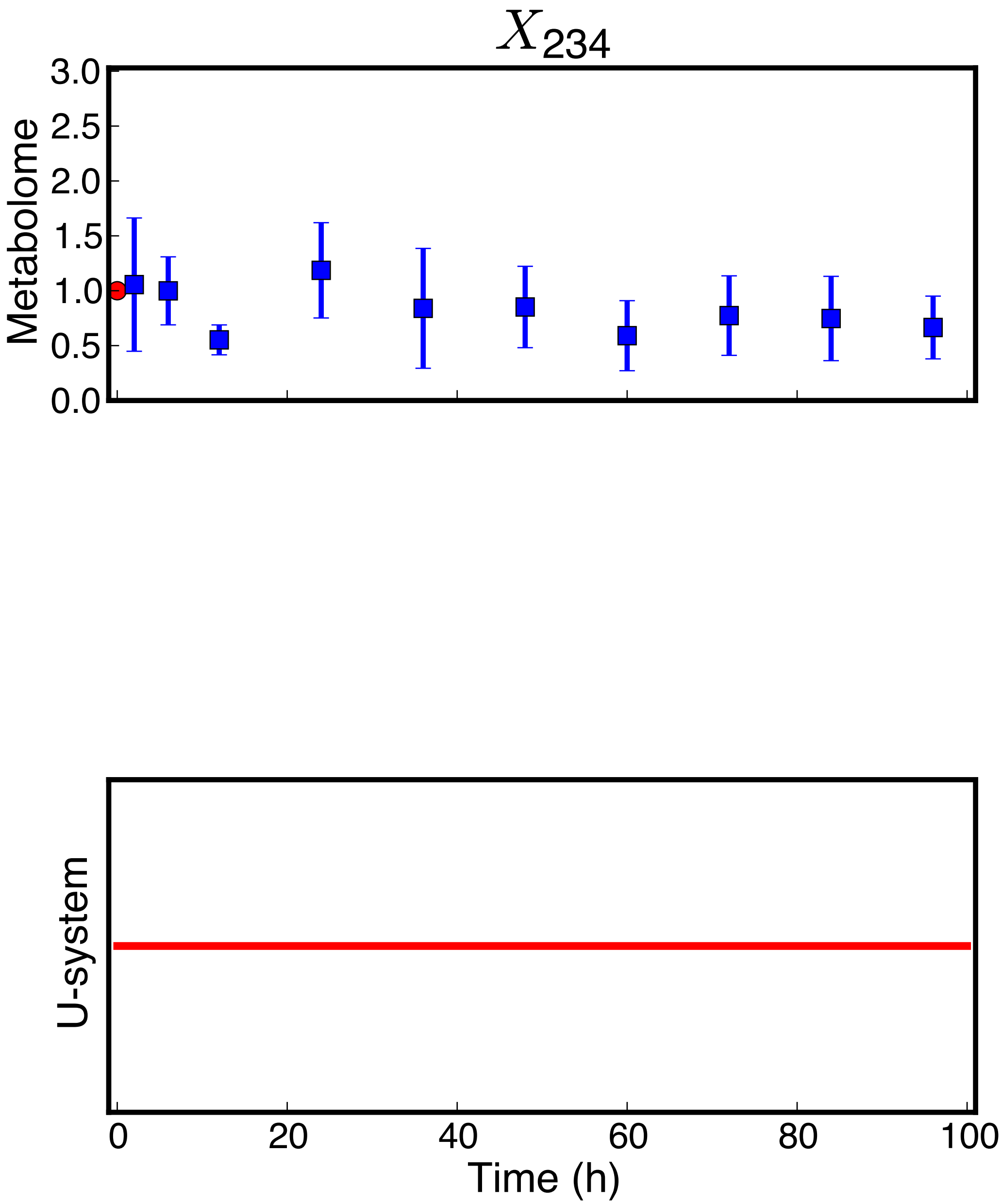

X_{234}
Metabolome
3.0
2.5
2.0
1.5
1.0
0.5
0.0
U-system
0
20
40
60
80
100
Time (h)

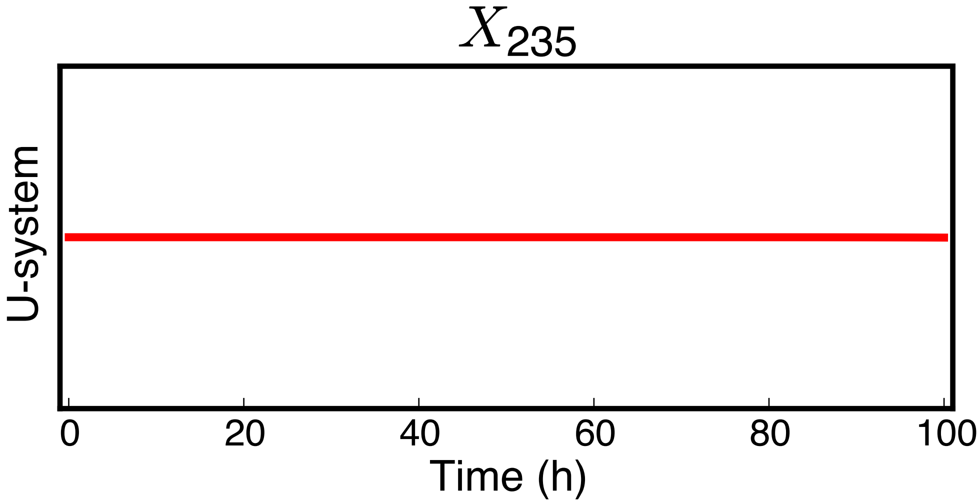
X_{235}
U-system
0
20
40
60
80
100
Time (h)

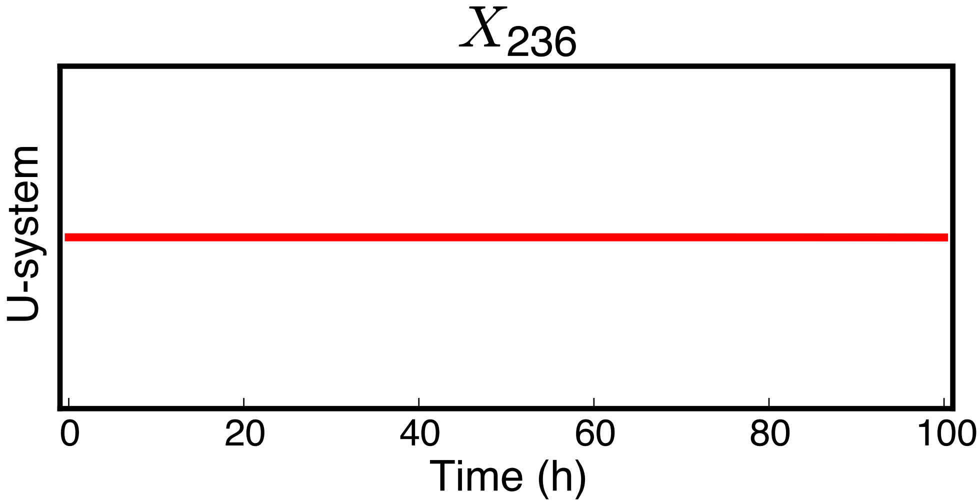

X_{236}
U-system
0
20
40
60
80
100
Time (h)

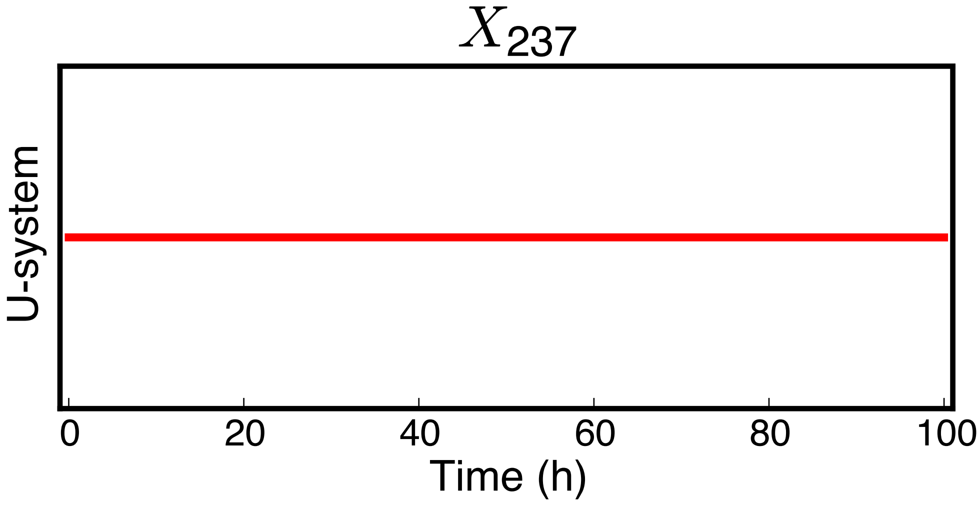
X_{237}
U-system
0
20
40
60
80
100
Time (h)

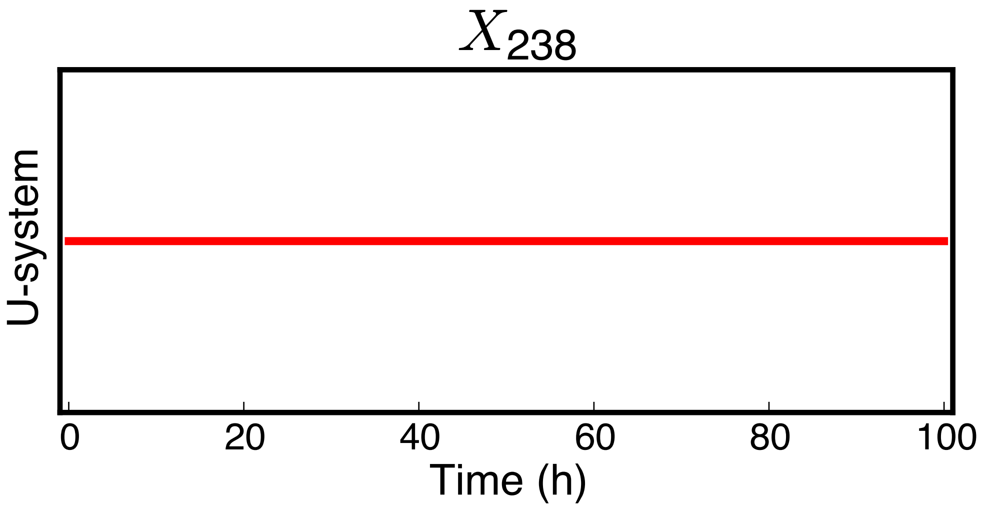

X_{238}
U-system
0
20
40
60
80
100
Time (h)

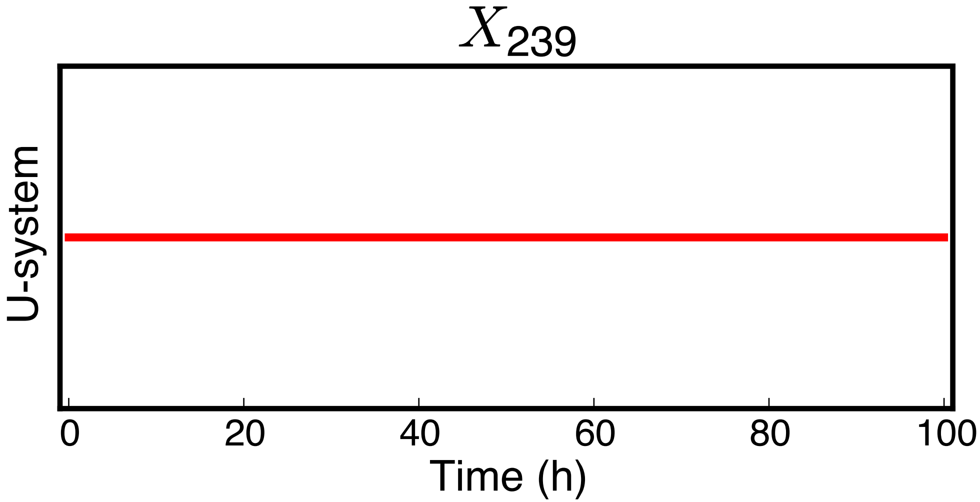
X_{239}
U-system
0
20
40
60
80
100
Time (h)

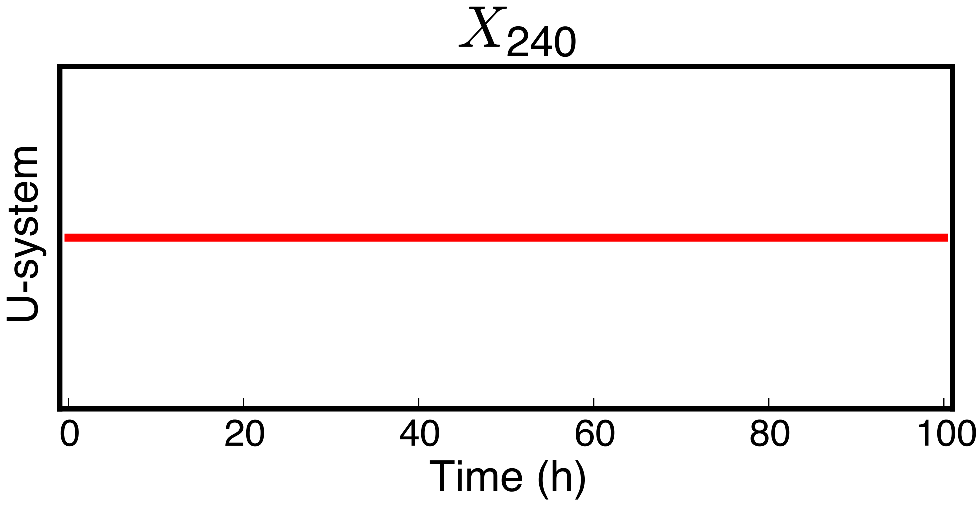

X_{240}
U-system
0
20
40
60
80
100
Time (h)

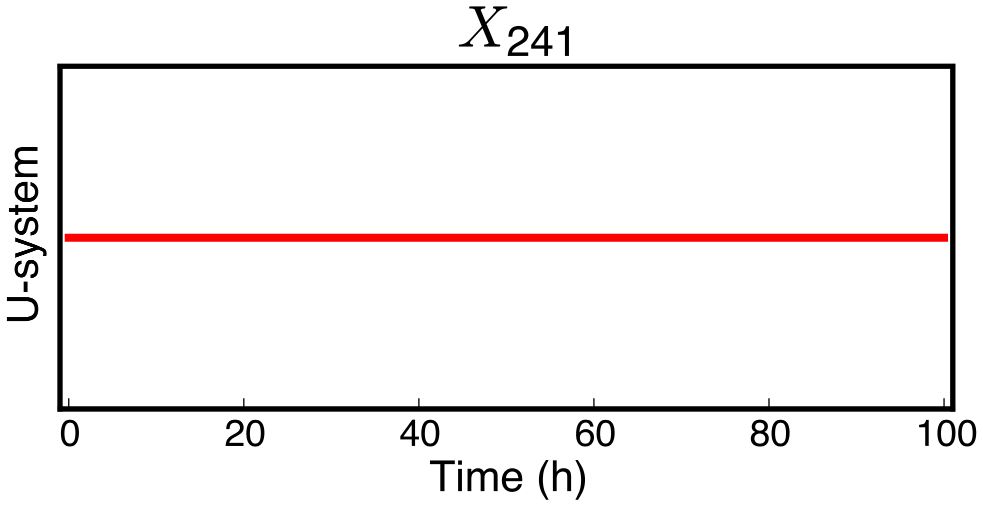

X_{241}
U-system
0
20
40
60
80
100
Time (h)

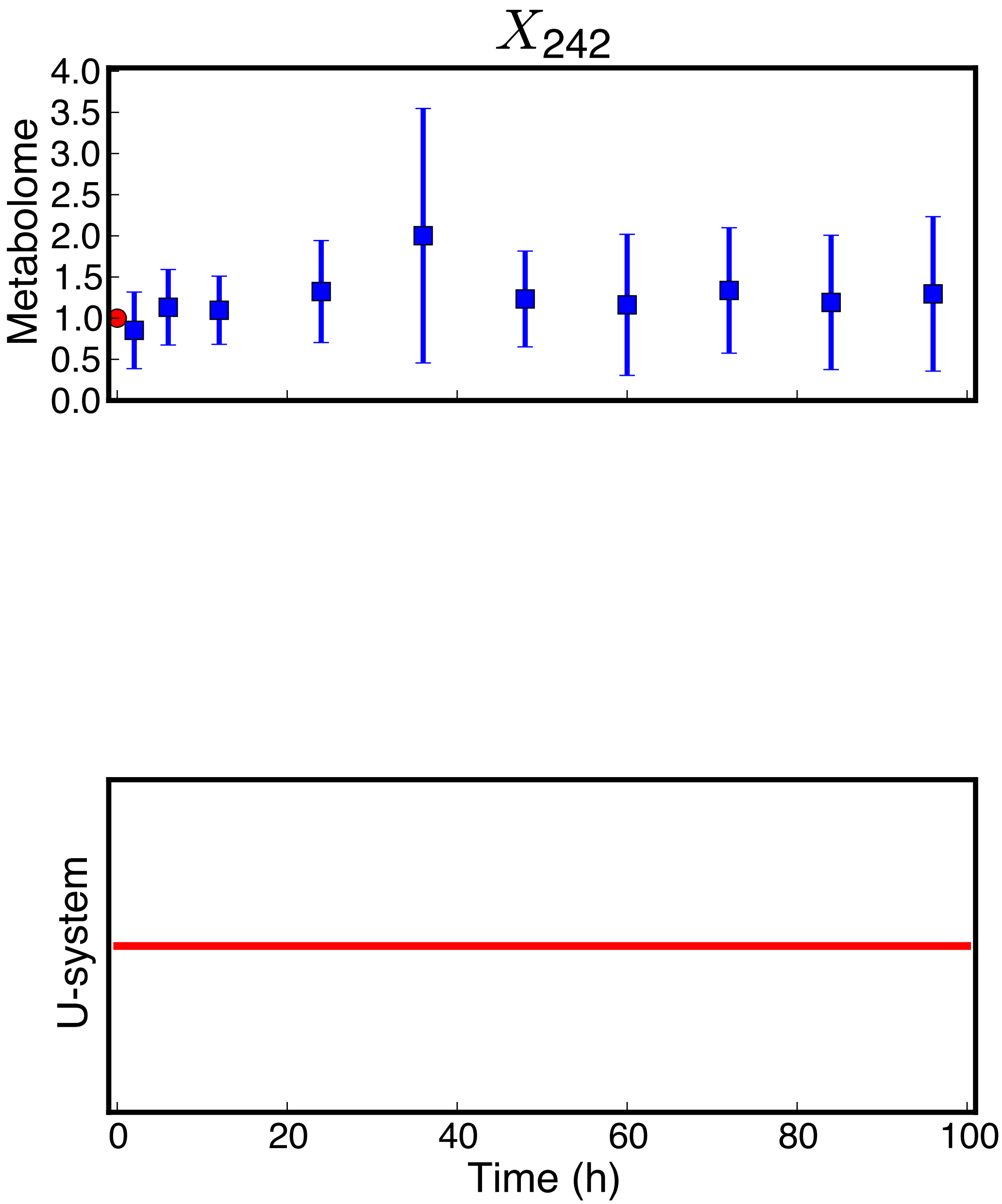

X_{242}
Metabolome
4.0
3.5
3.0
2.5
2.0
1.5
1.0
0.5
0.0
U-system
0
20
40
60
80
100
Time (h)

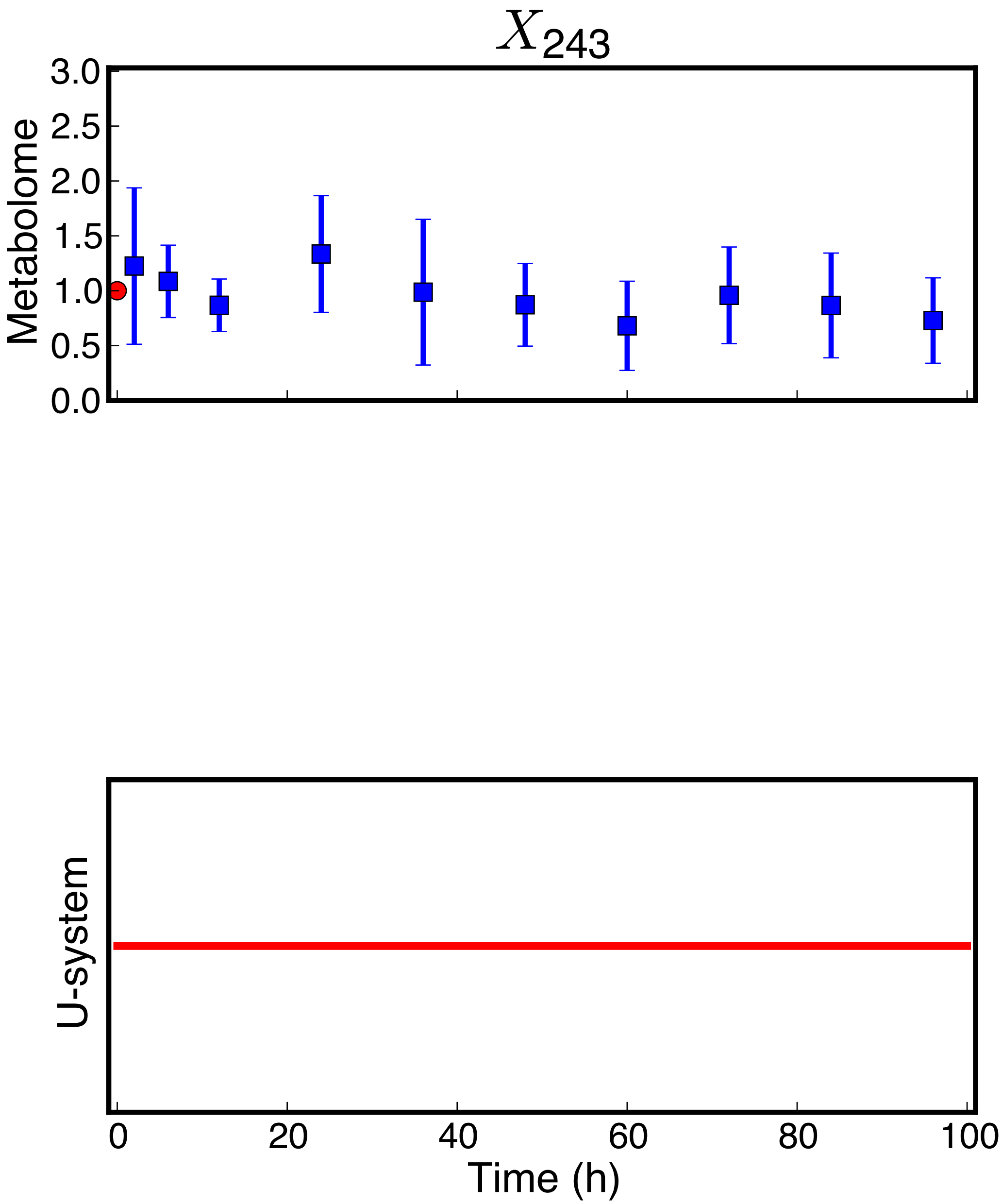

X_{243}
Metabolome
3.0
2.5
2.0
1.5
1.0
0.5
0.0
U-system
0
20
40
60
80
100
Time (h)

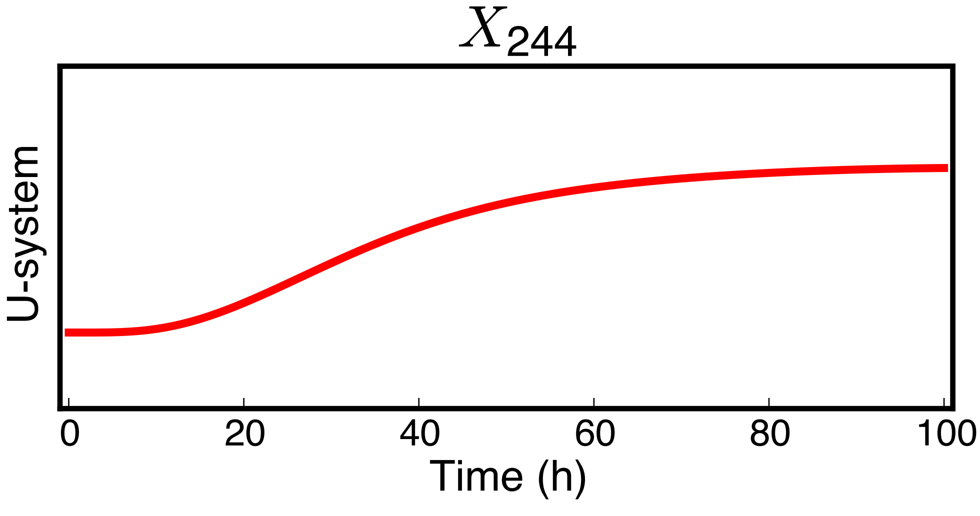
X_{244}
U-system
0
20
40
60
80
100
Time (h)

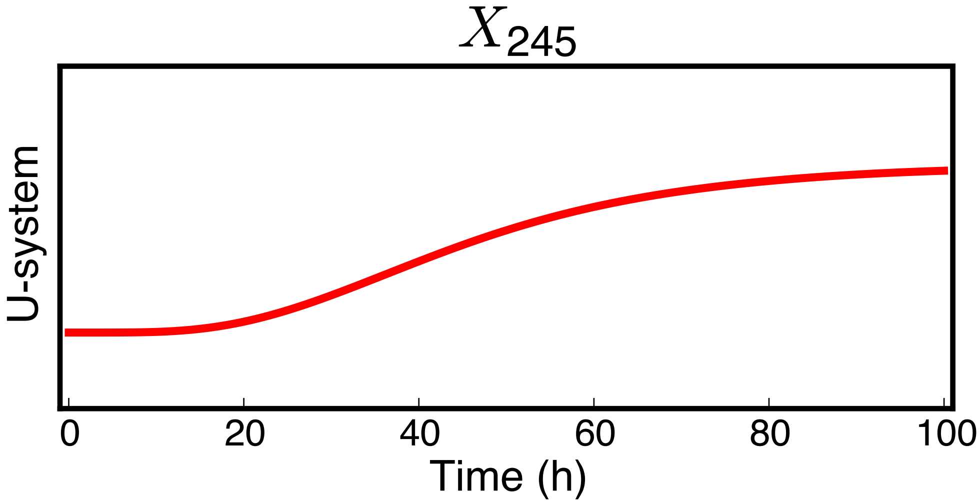
X_{245}
U-system
0
20
40
60
80
100
Time (h)

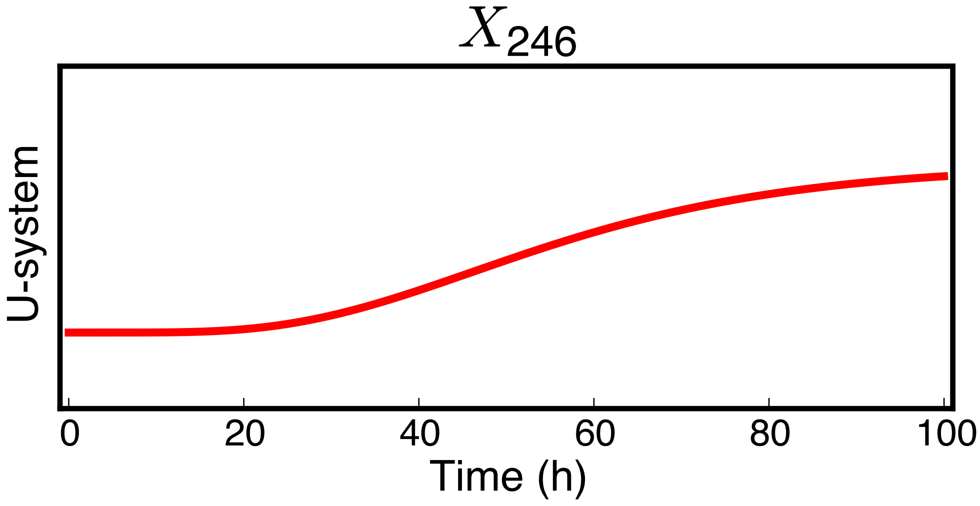

X_{246}
U-system
0
20
40
60
80
100
Time (h)

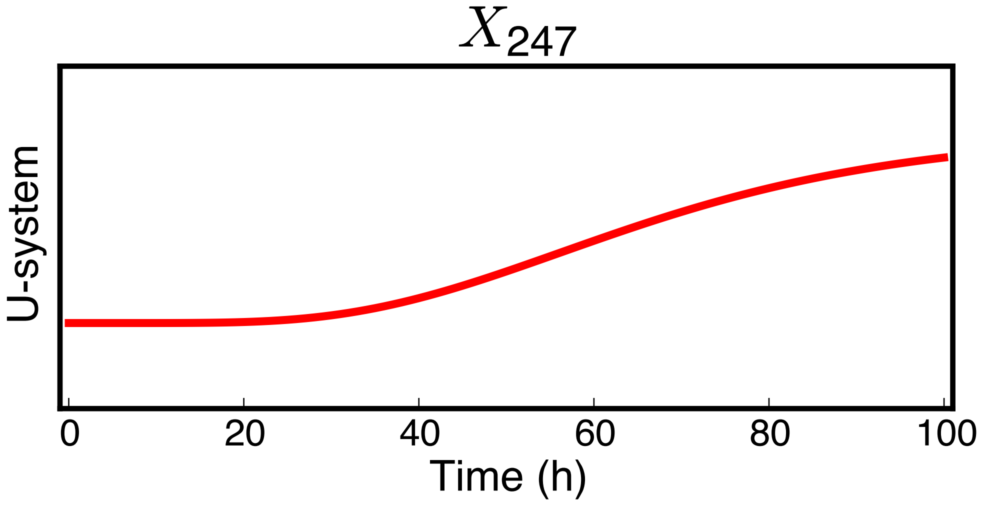

X_{247}
U-system
0
20
40
60
80
100
Time (h)

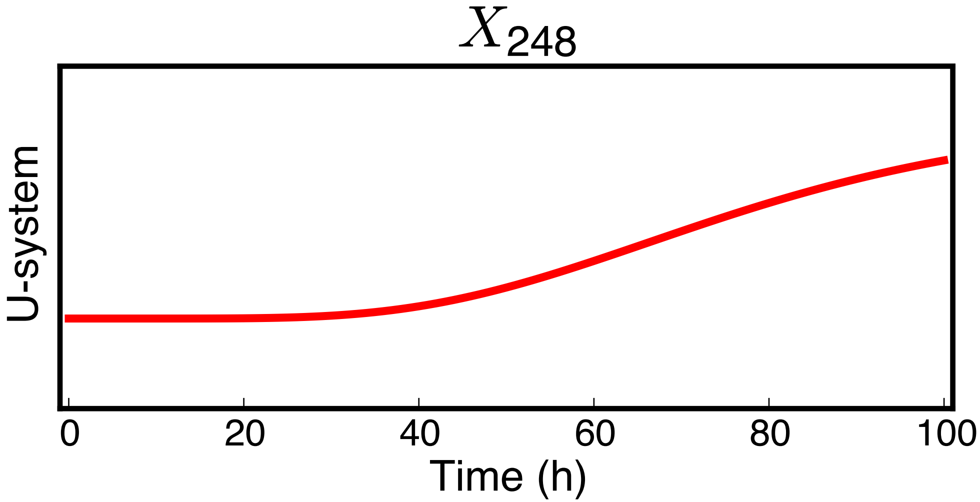
X_{248}
U-system
0
20
40
60
80
100
Time (h)

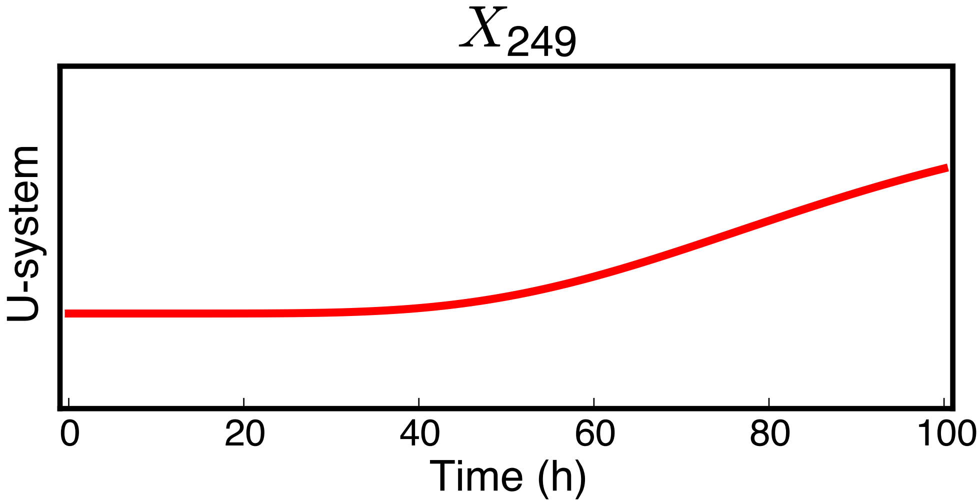
X_{249}
U-system
0
20
40
60
80
100
Time (h)

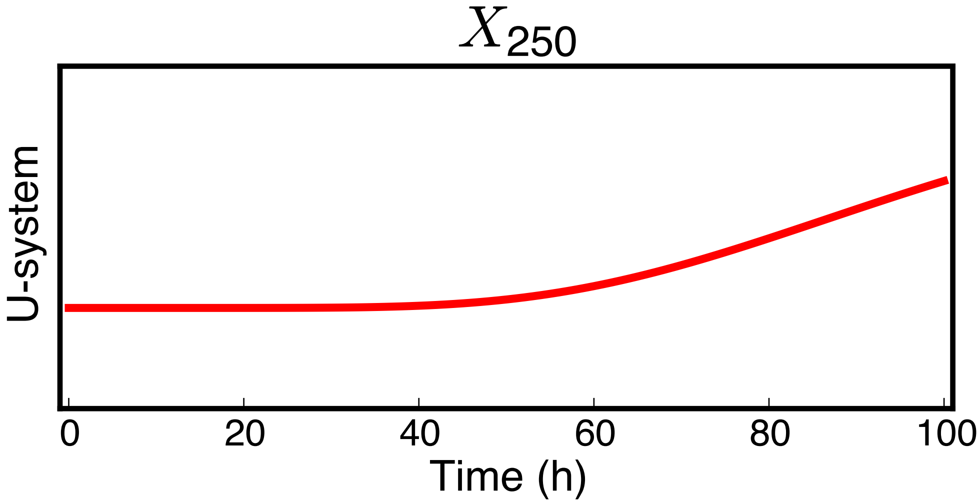

X_{250}
U-system
0
20
40
60
80
100
Time (h)

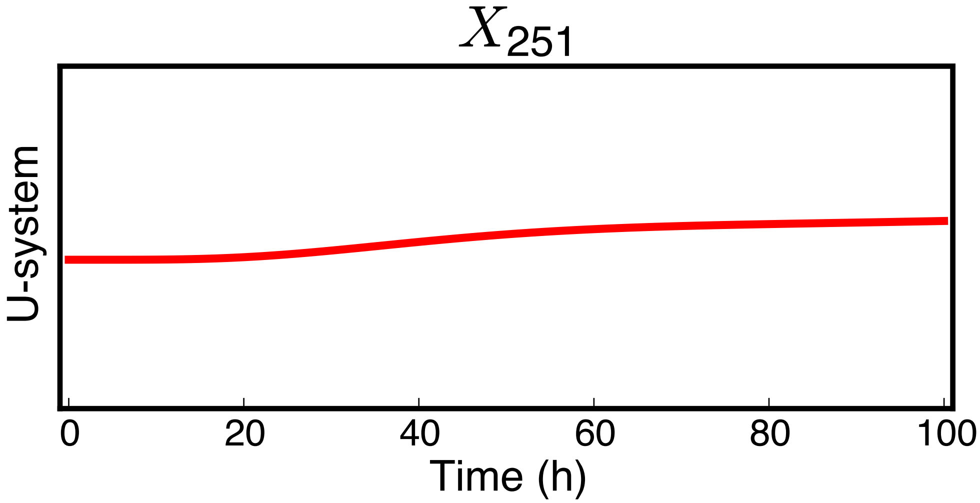

X_{251}
U-system
0
20
40
60
80
100
Time (h)

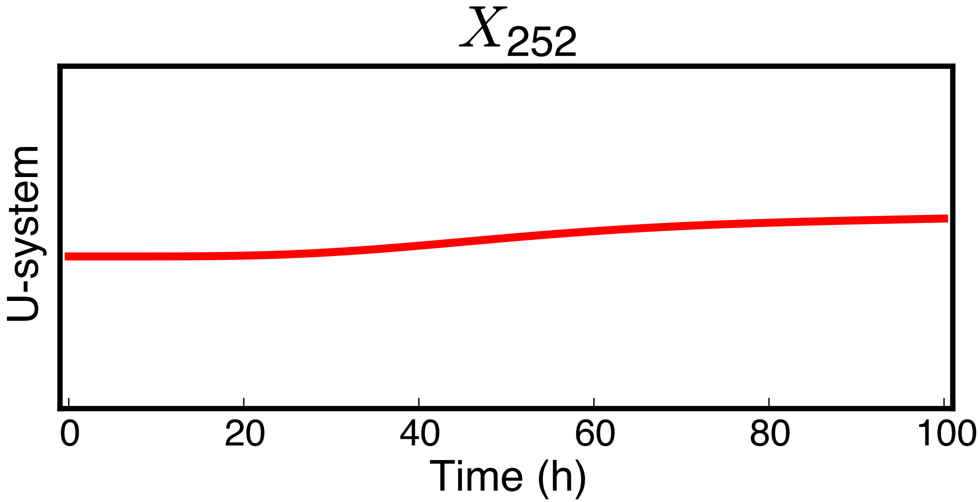
X_{252}
U-system
0
20
40
60
80
100
Time (h)

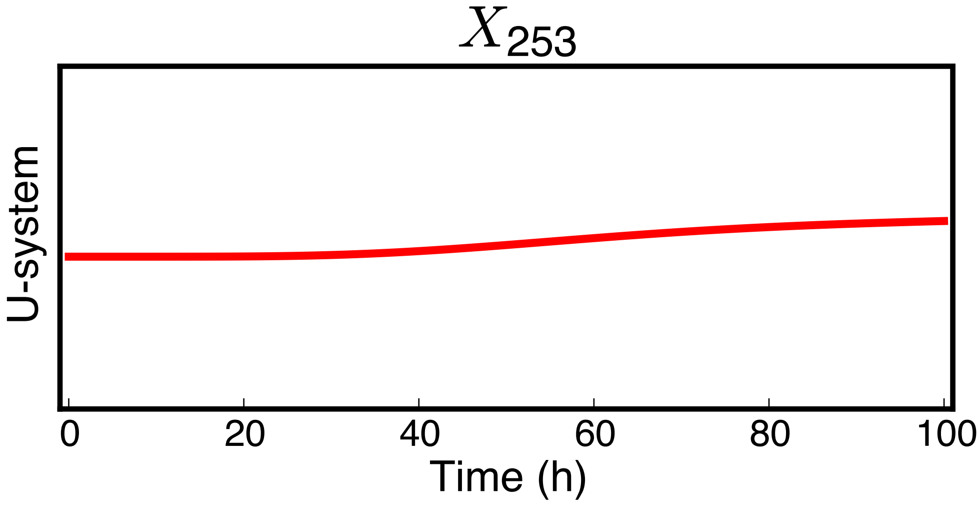

X_{253}
U-system
0
20
40
60
80
100
Time (h)

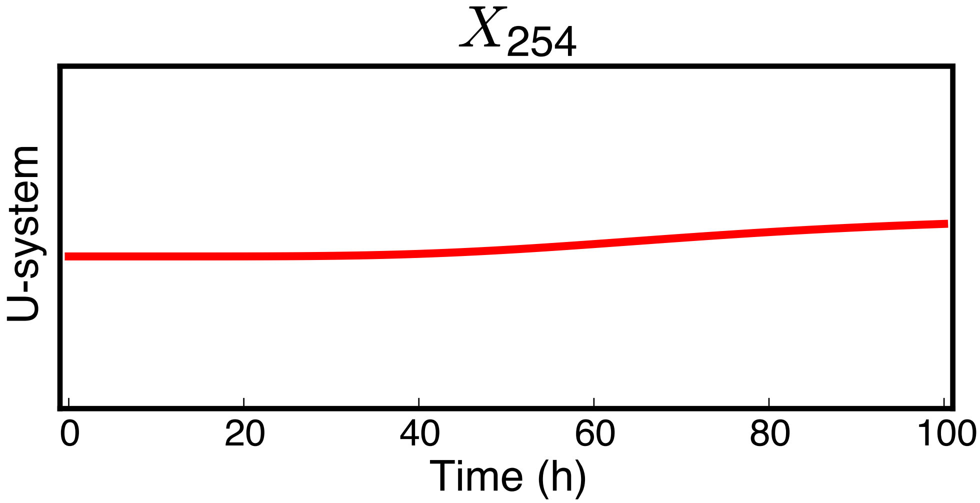

X_{254}
U-system
0
20
40
60
80
100
Time (h)

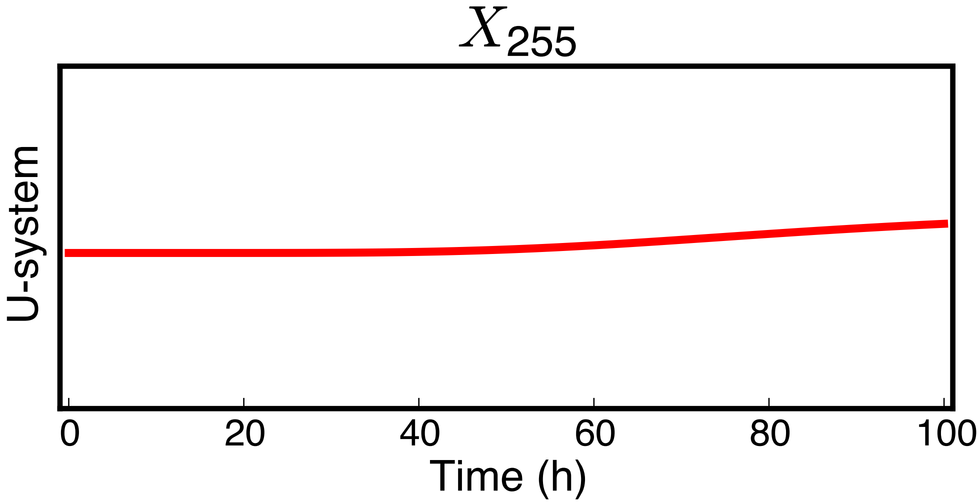

X_{255}
U-system
0
20
40
60
80
100
Time (h)

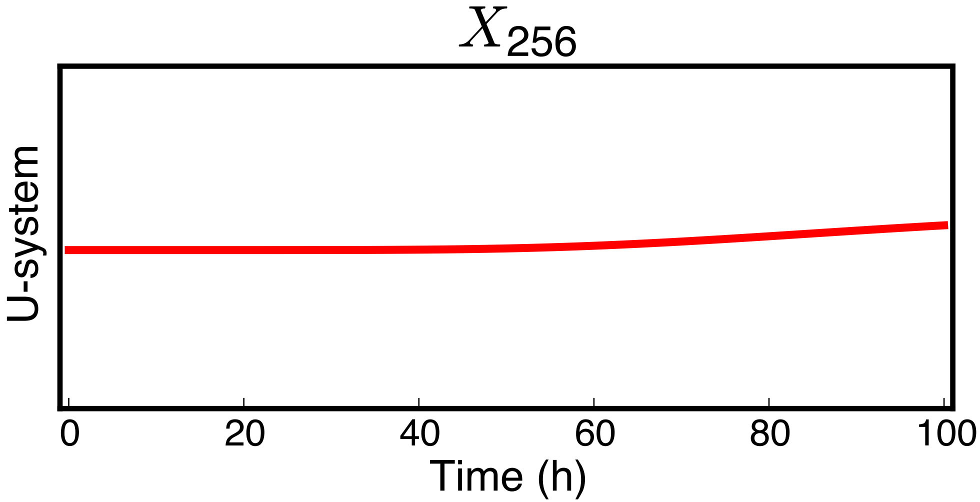

X_{256}
U-system
0
20
40
60
80
100
Time (h)

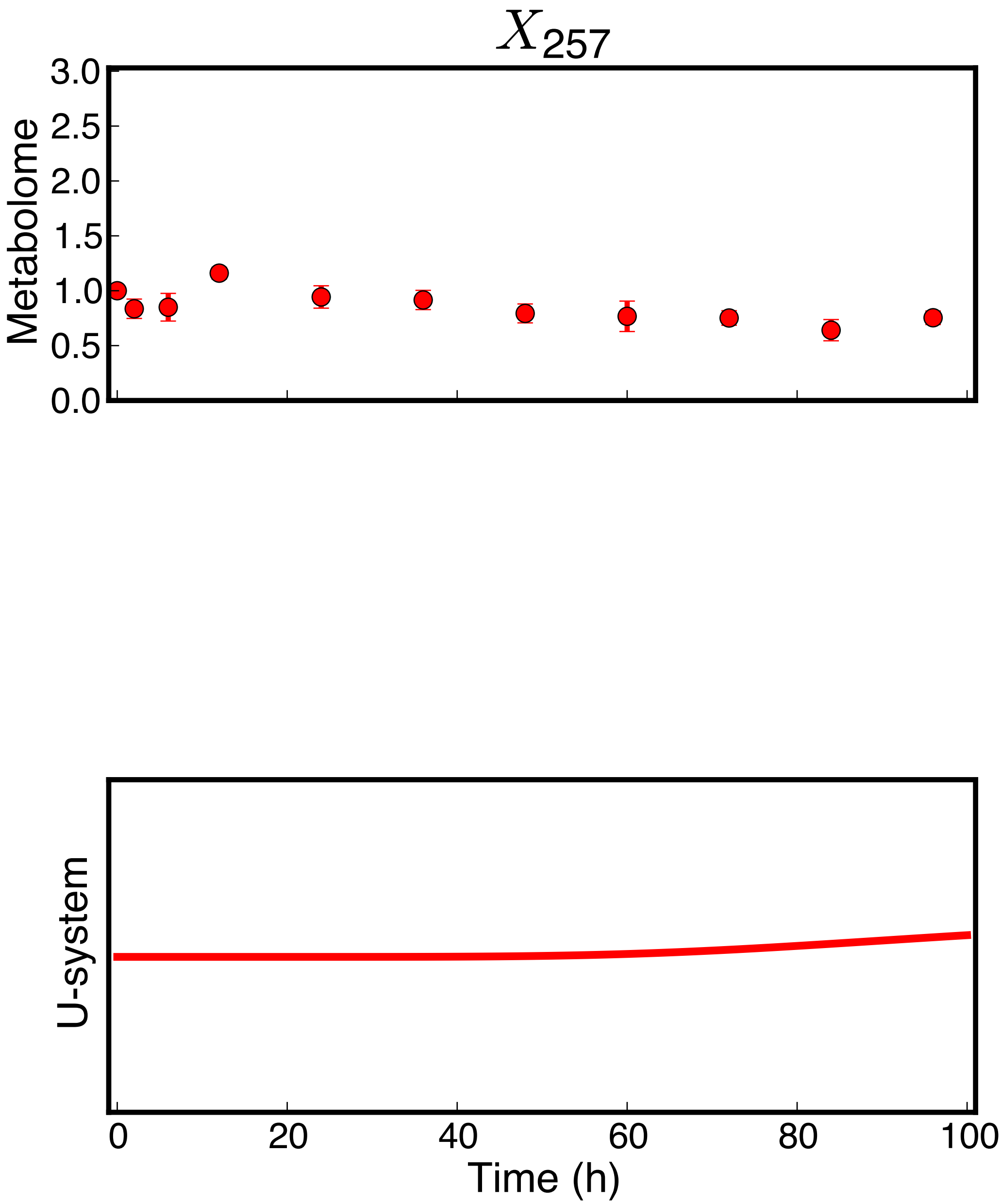
X_{257}
Metabolome
3.0
2.5
2.0
1.5
1.0
0.5
0.0
U-system
0
20
40
60
80
100
Time (h)

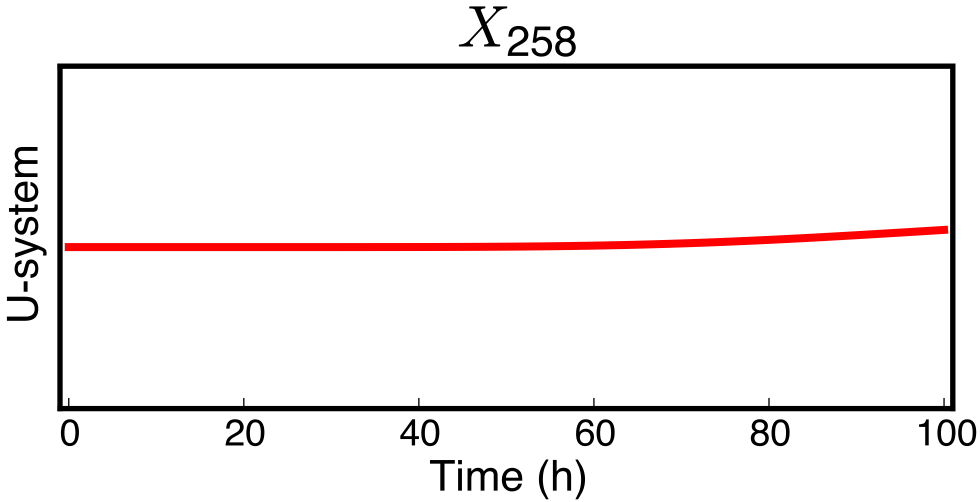
X_{258}
U-system
0
20
40
60
80
100
Time (h)

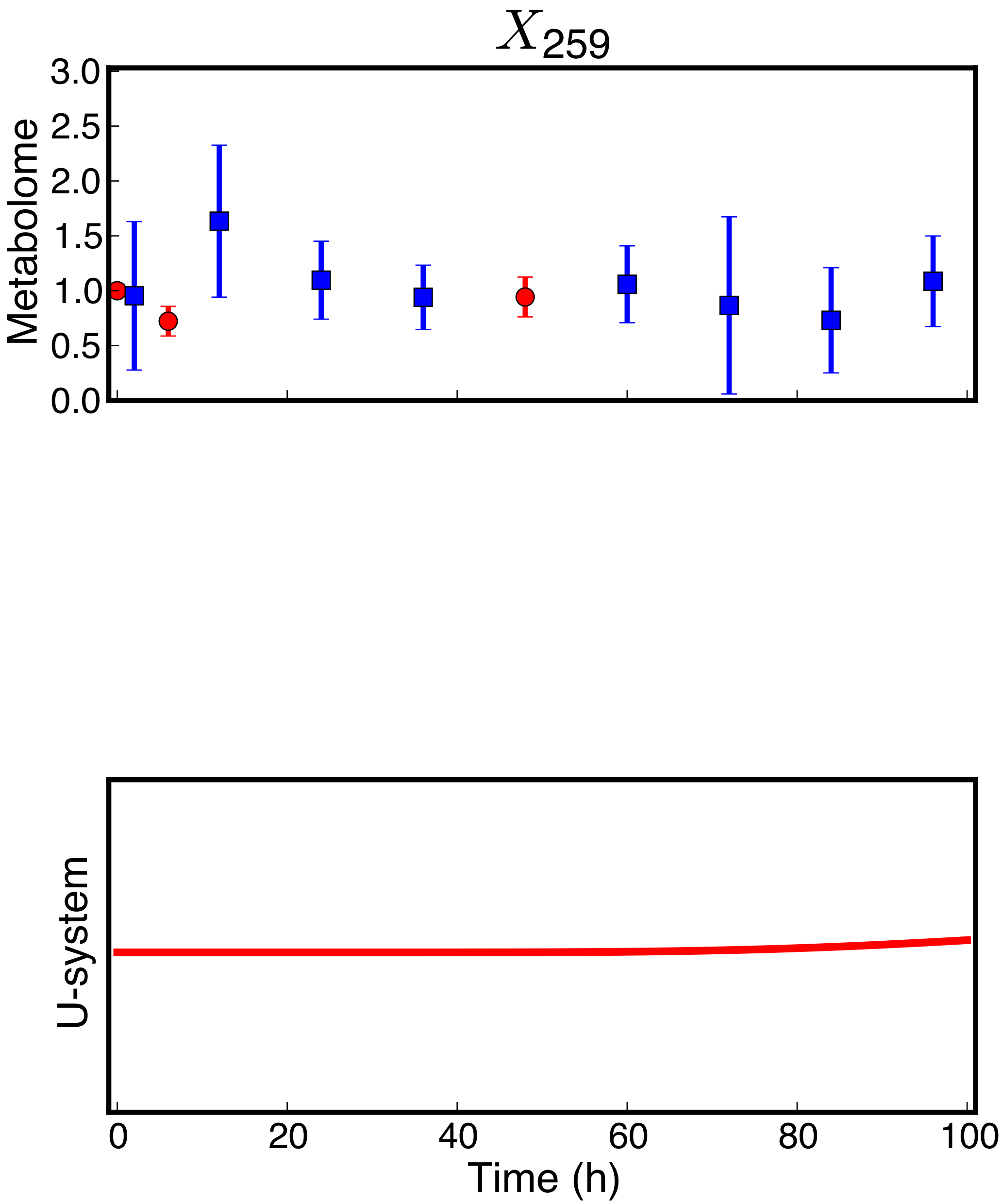

X_{259}
Metabolome
3.0
2.5
2.0
1.5
1.0
0.5
0.0
U-system
0
20
40
60
80
100
Time (h)

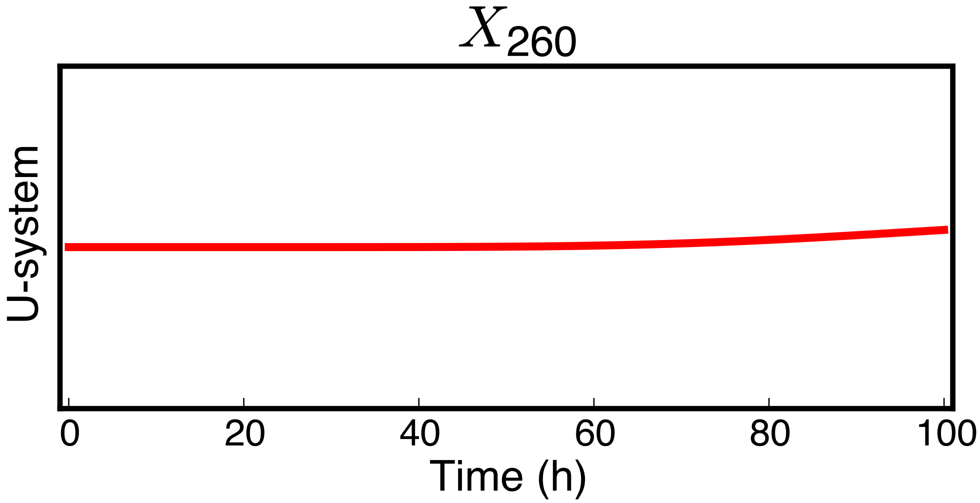
X_{260}
U-system
0
20
40
60
80
100
Time (h)

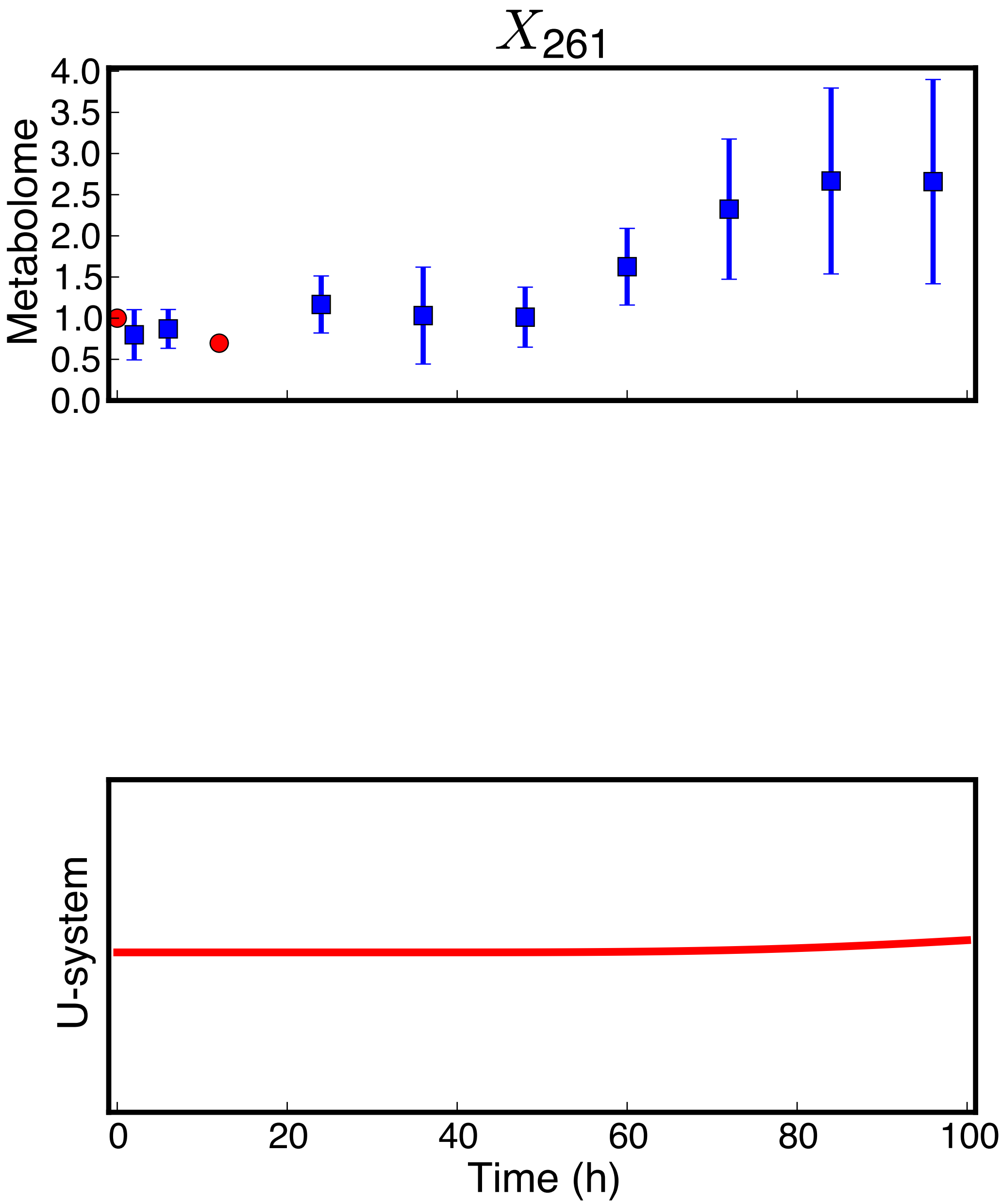

X_{261}
Metabolome
4.0
3.5
3.0
2.5
2.0
1.5
1.0
0.5
0.0
U-system
0
20
40
60
80
100
Time (h)

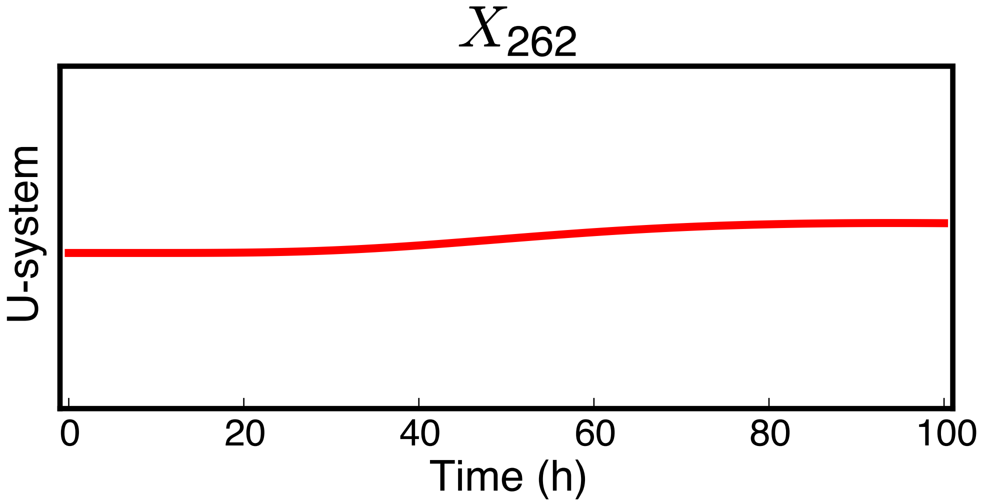

X_{262}
U-system
0
20
40
60
80
100
Time (h)

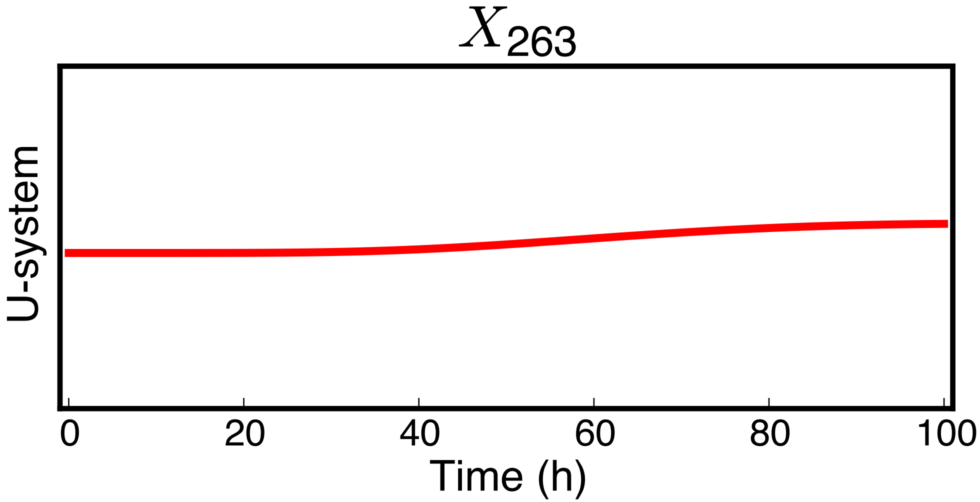

X_{263}
U-system
0
20
40
60
80
100
Time (h)

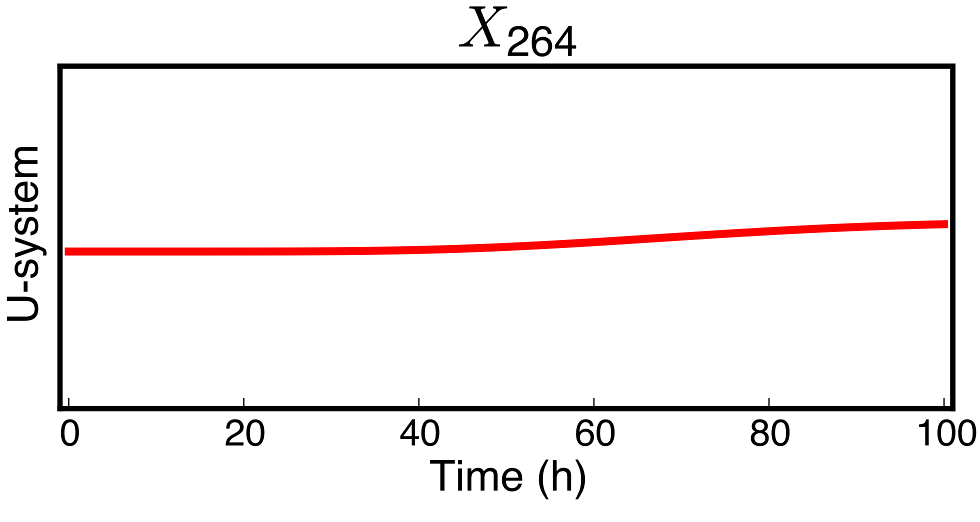
X_{264}
U-system
0
20
40
60
80
100
Time (h)

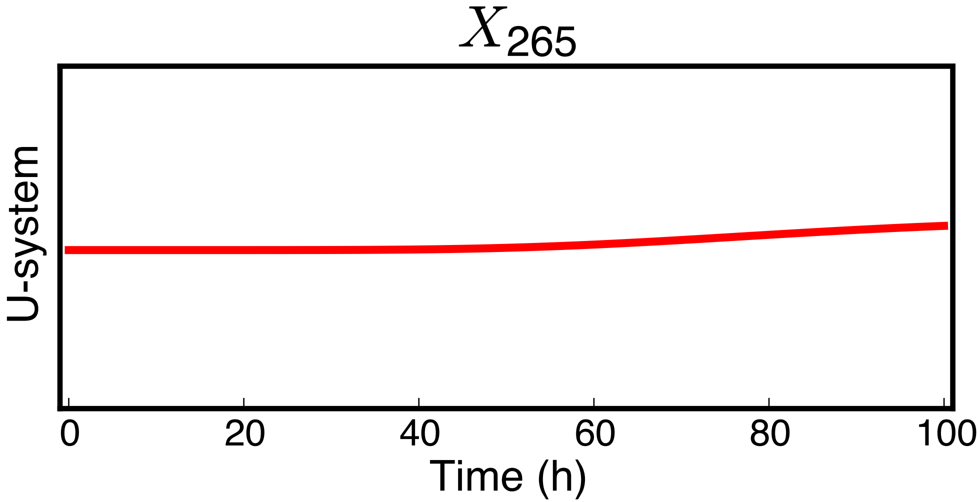
X_{265}
U-system
0
20
40
60
80
100
Time (h)

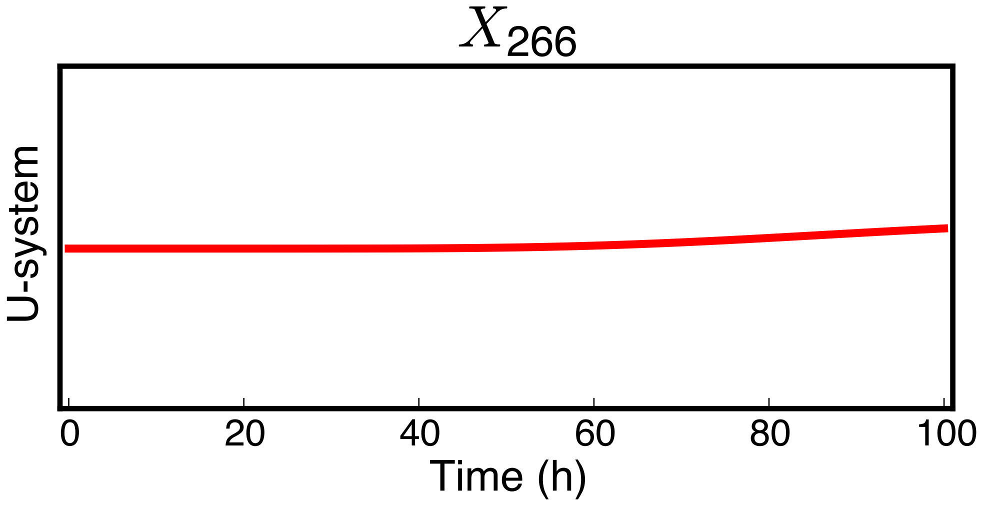

X_{266}
U-system
0
20
40
60
80
100
Time (h)

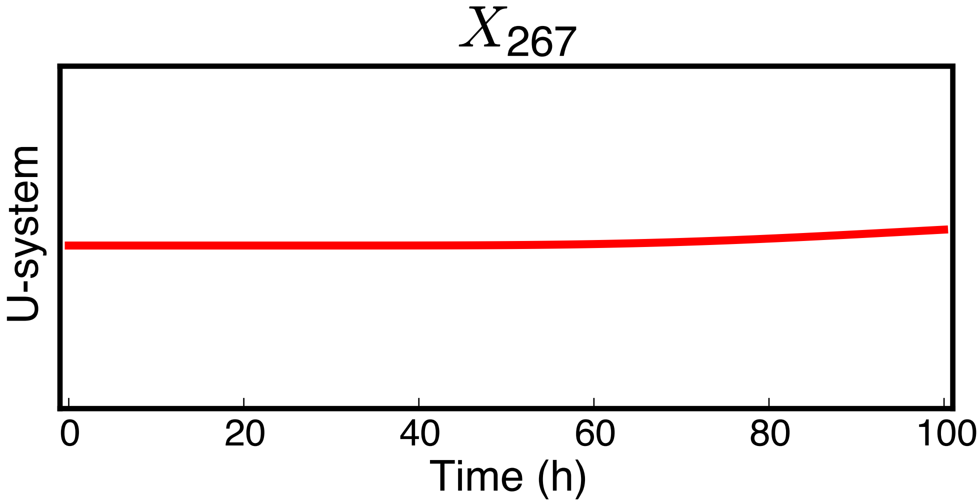

X_{267}
U-system
0
20
40
60
80
100
Time (h)

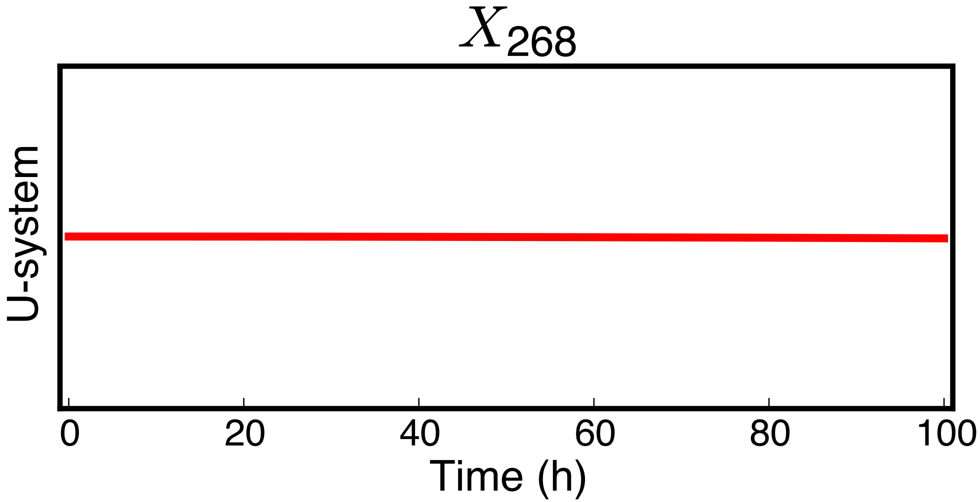
X_{268}
U-system
0
20
40
60
80
100
Time (h)

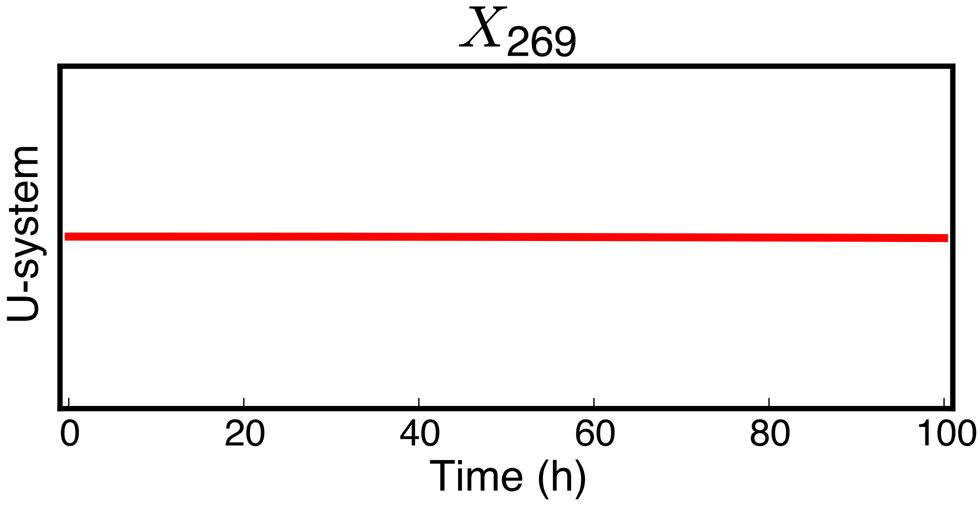

X_{269}
U-system
0
20
40
60
80
100
Time (h)

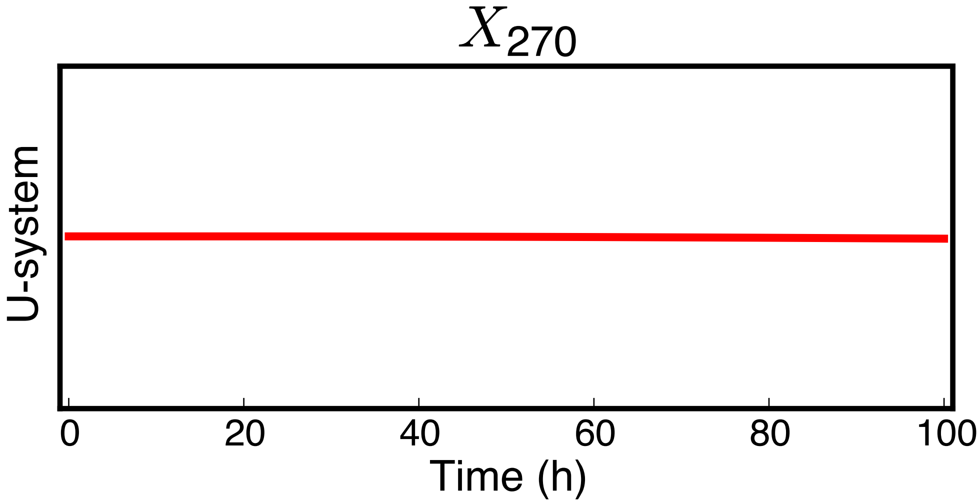

X_{270}
U-system
0
20
40
60
80
100
Time (h)

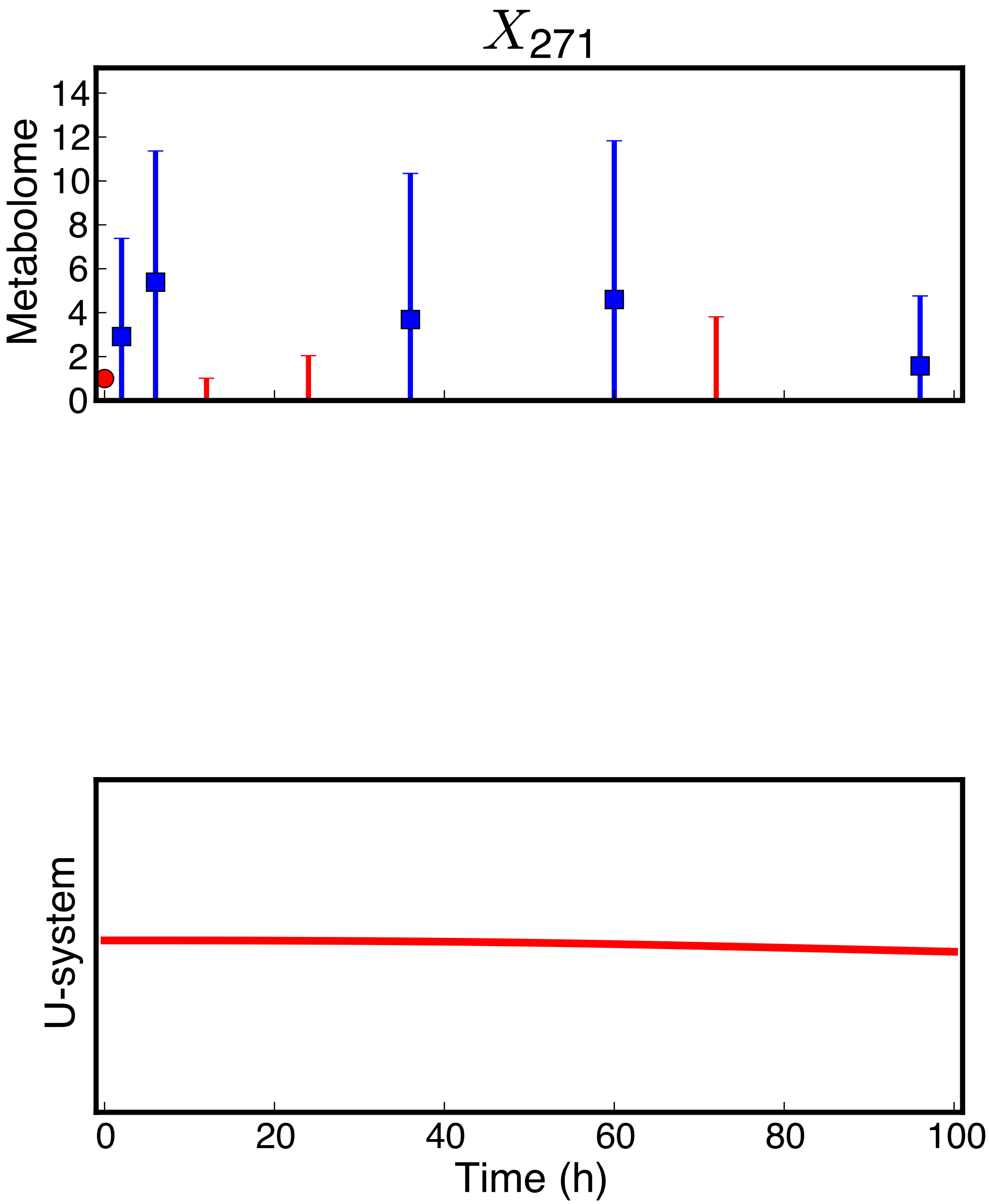

X_{271}
Metabolome
0
2
4
6
8
10
12
14
U-system
0
20
40
60
80
100
Time (h)

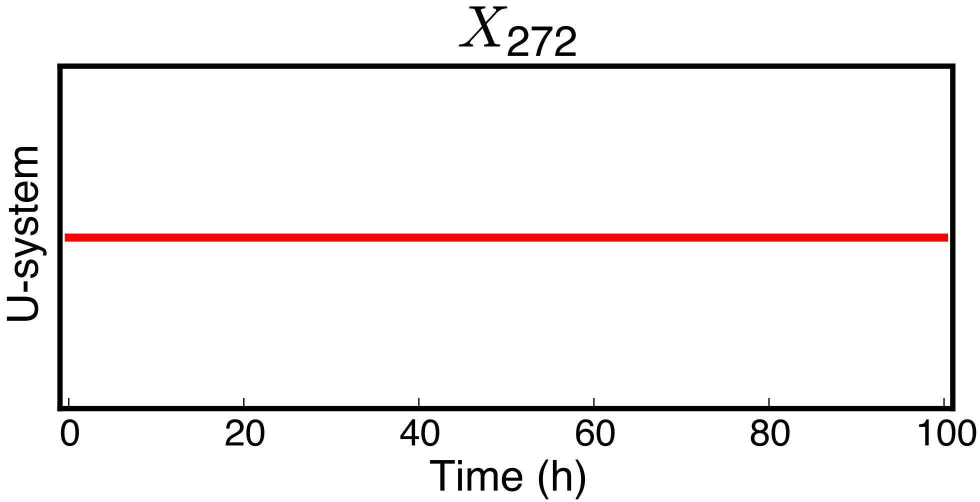
X_{272}
U-system
0
20
40
60
80
100
Time (h)

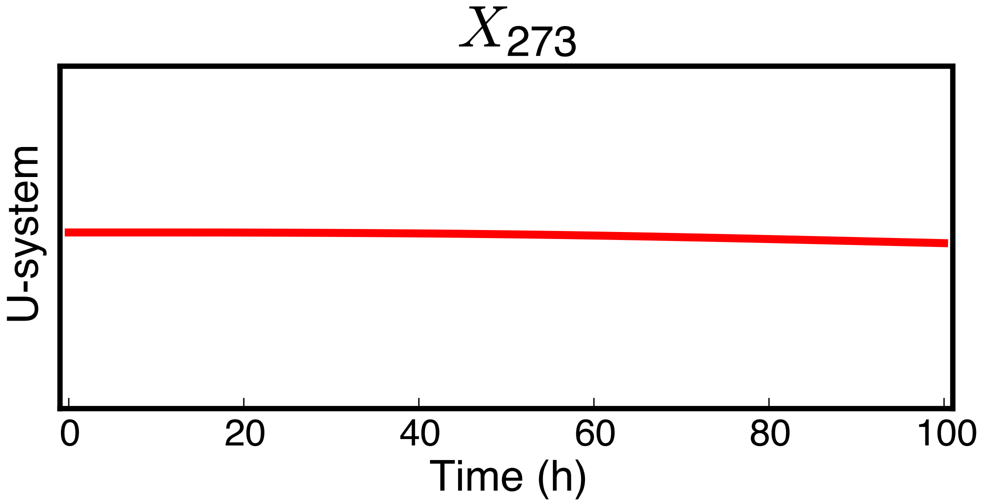

X_{273}
U-system
0
20
40
60
80
100
Time (h)

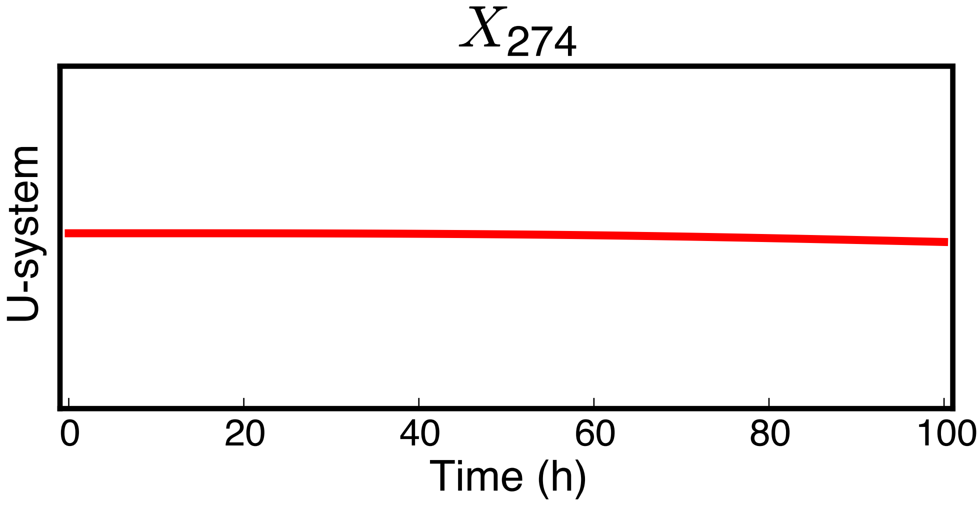

X_{274}
U-system
0
20
40
60
80
100
Time (h)

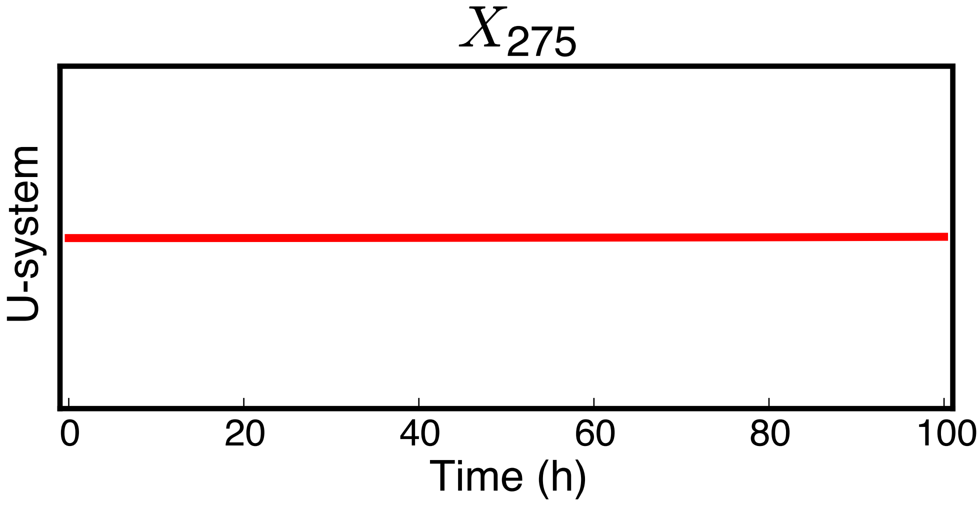
X_{275}
U-system
0
20
40
60
80
100
Time (h)

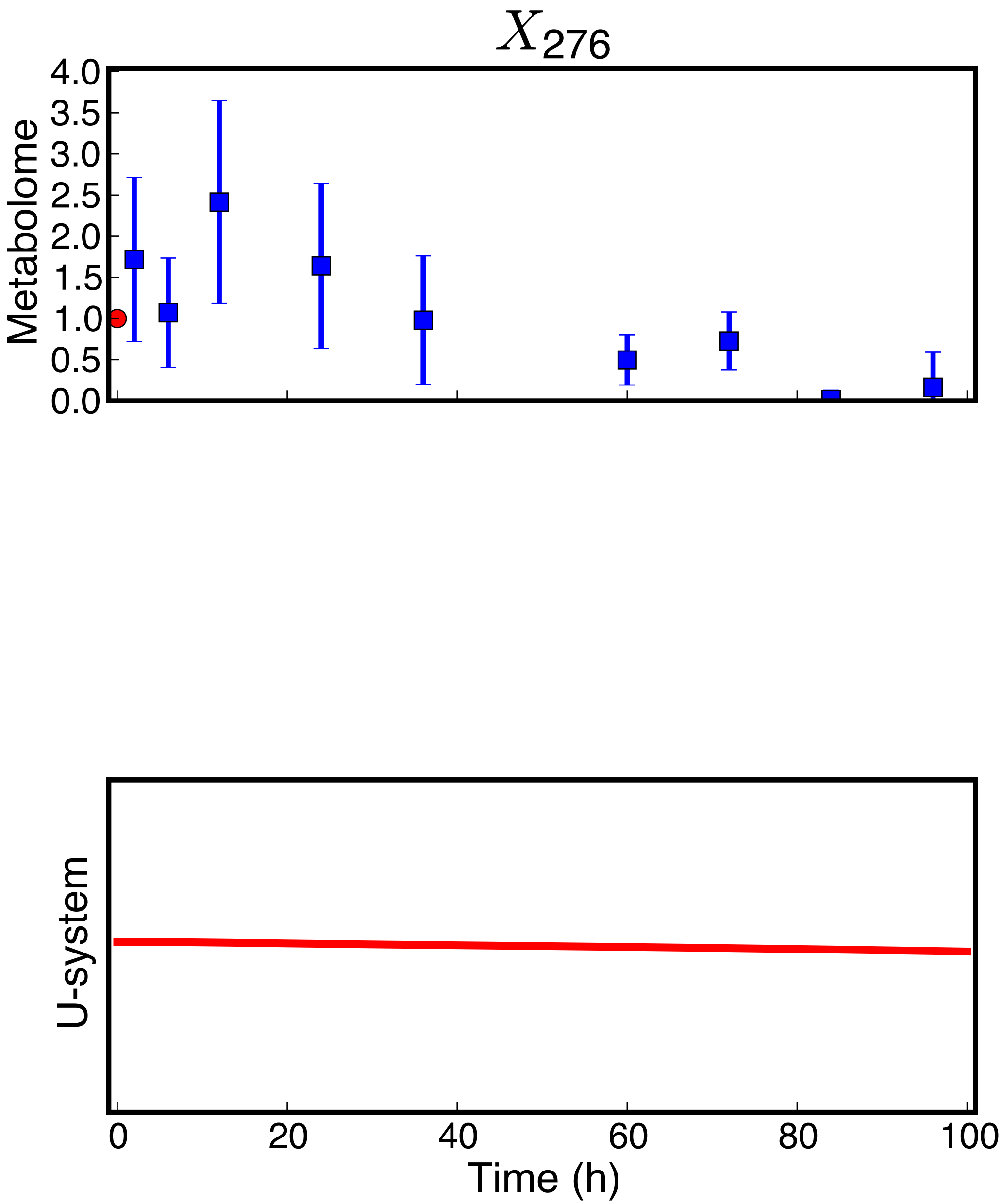

X_{276}
Metabolome
4.0
3.5
3.0
2.5
2.0
1.5
1.0
0.5
0.0
U-system
0
20
40
60
80
100
Time (h)

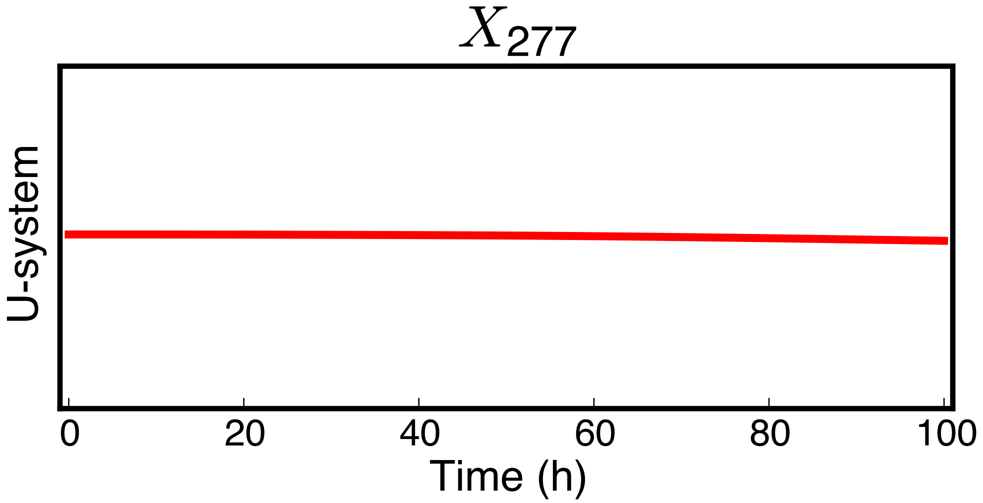

X_{277}
U-system
0
20
40
60
80
100
Time (h)

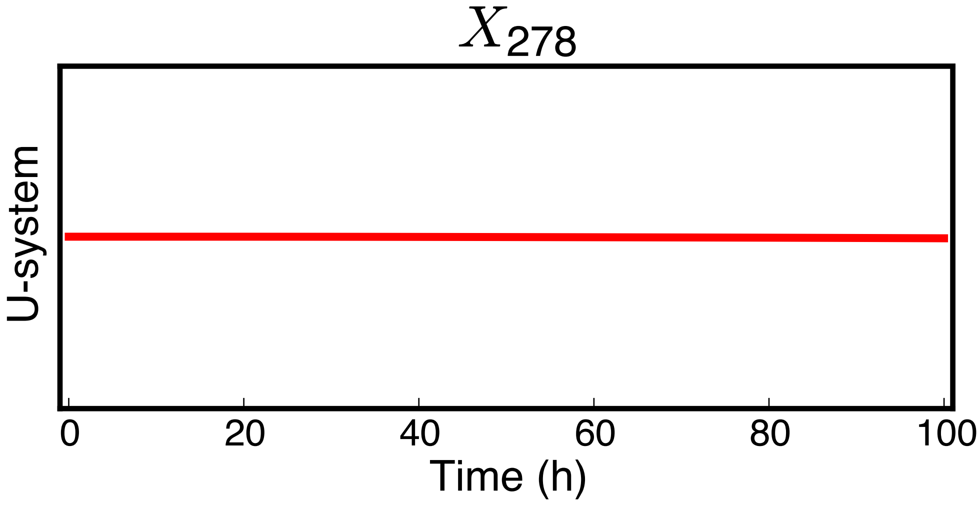
X_{278}
U-system
0
20
40
60
80
100
Time (h)

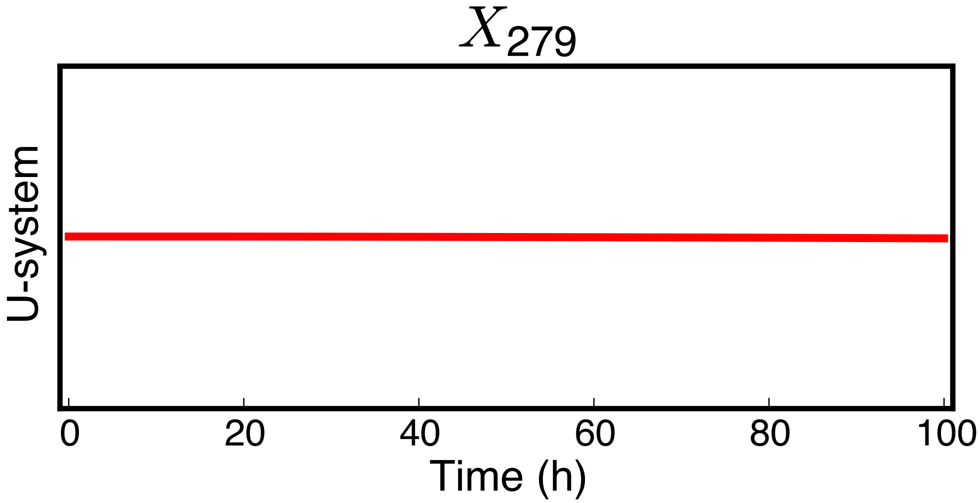

X_{279}
U-system
0
20
40
60
80
100
Time (h)

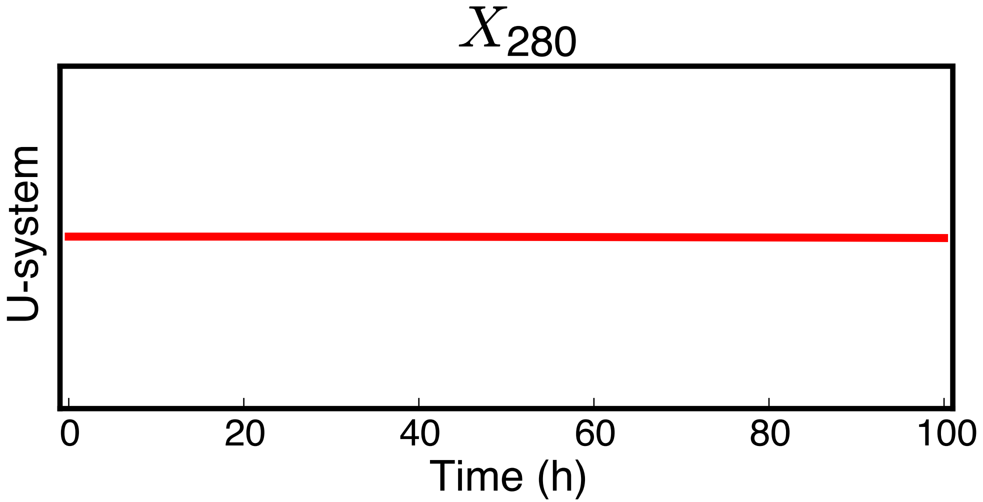
X_{280}
U-system
0
20
40
60
80
100
Time (h)

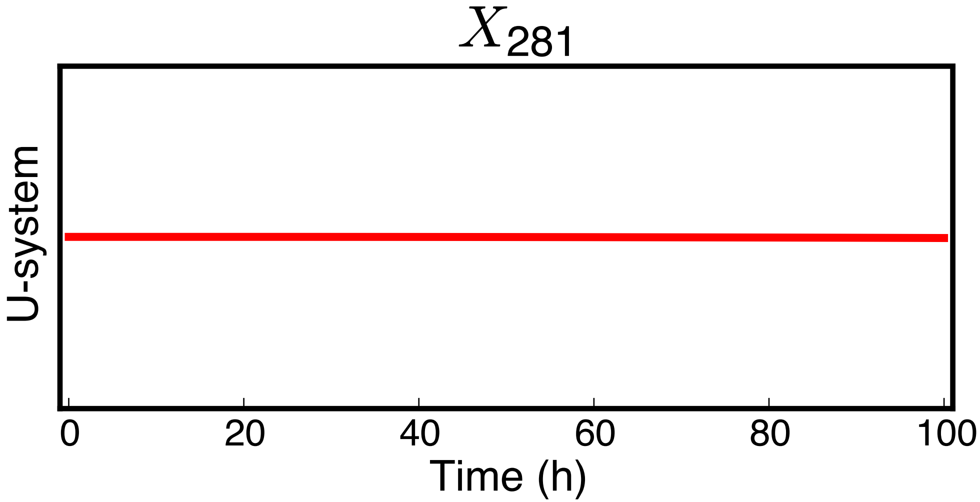

X_{281}
U-system
0
20
40
60
80
100
Time (h)

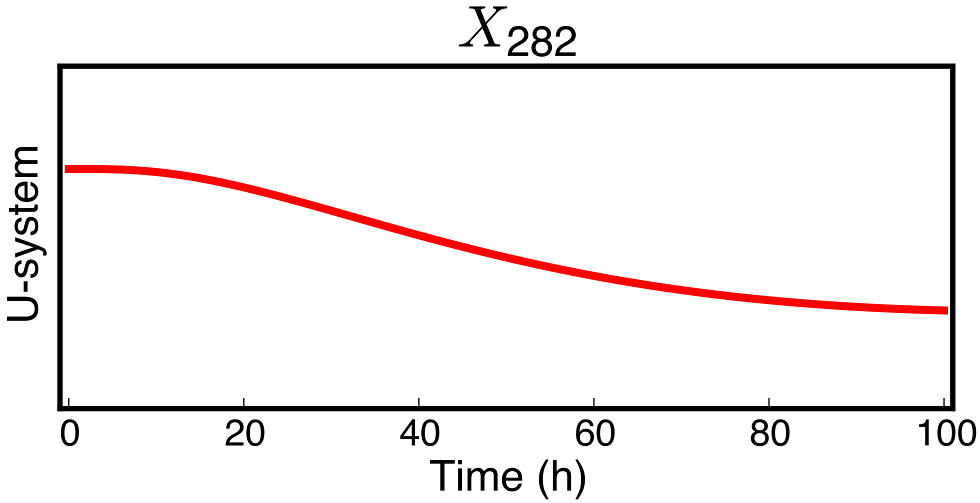

X_{282}
U-system
0
20
40
60
80
100
Time (h)

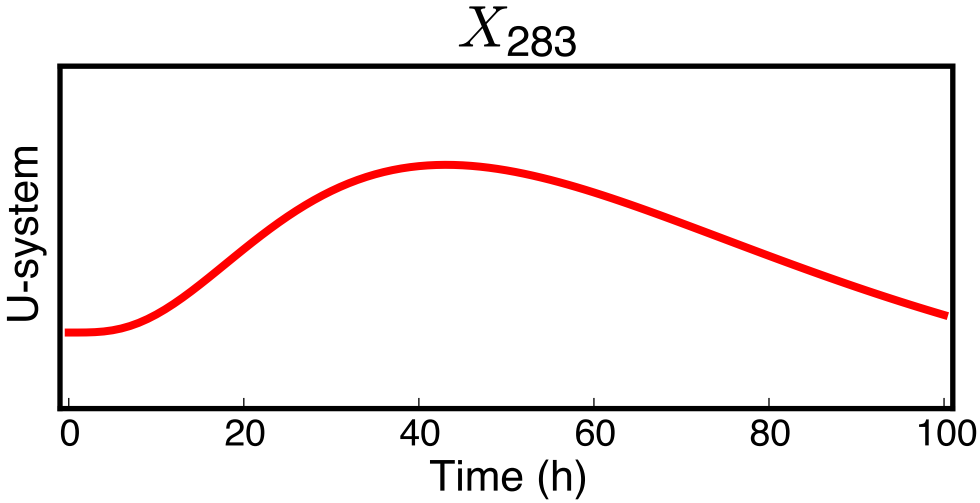

X_{283}
U-system
0
20
40
60
80
100
Time (h)

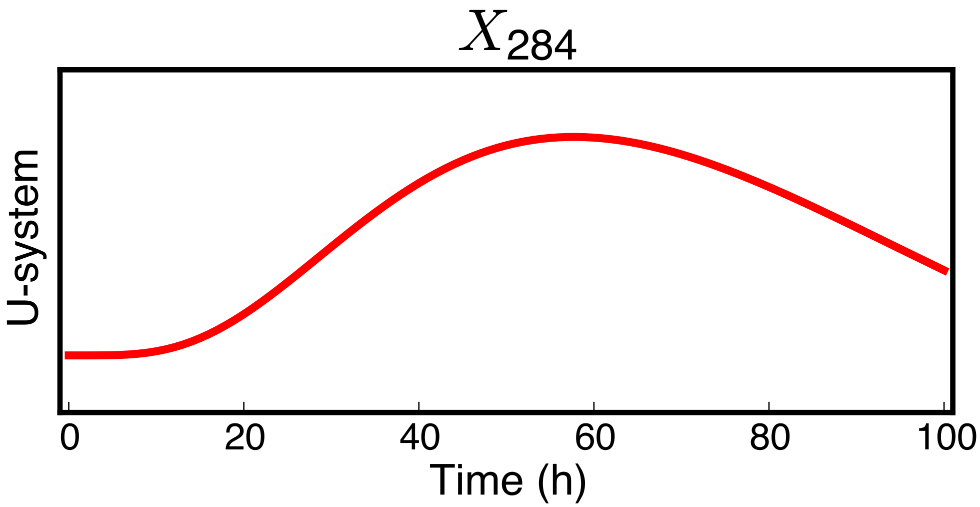
X_{284}
U-system
0
20
40
60
80
100
Time (h)

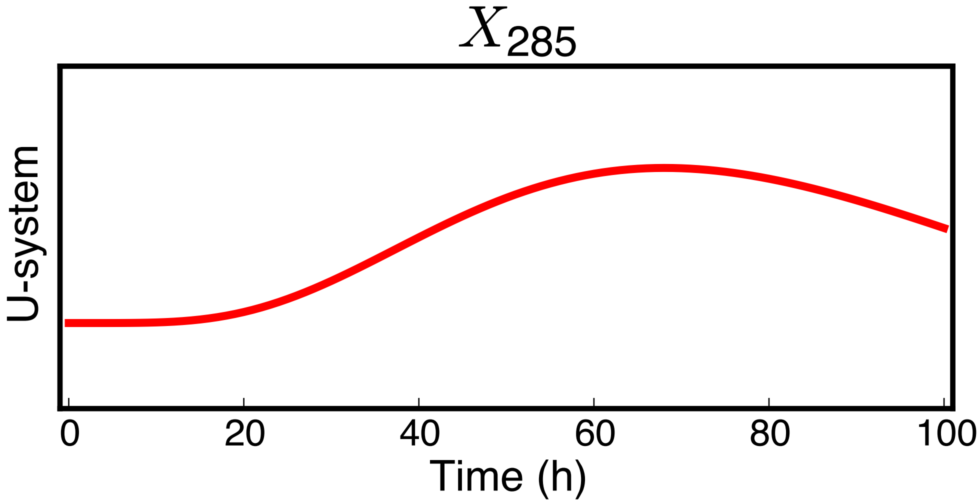

X_{285}
U-system
0
20
40
60
80
100
Time (h)

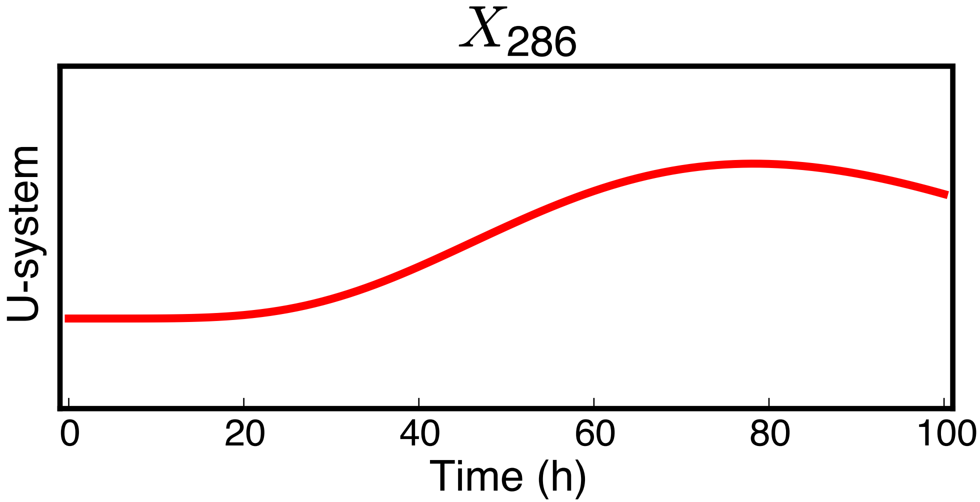

X_{286}
U-system
0
20
40
60
80
100
Time (h)

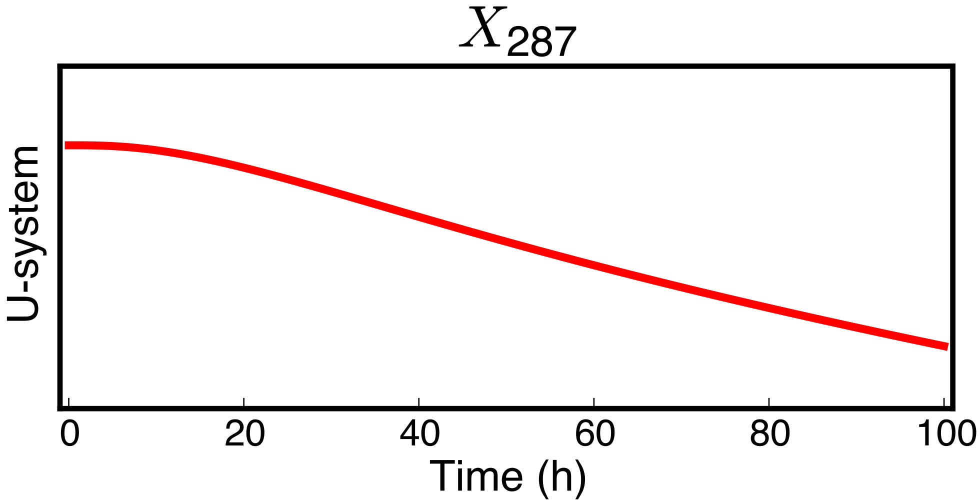

X_{287}
U-system
0
20
40
60
80
100
Time (h)

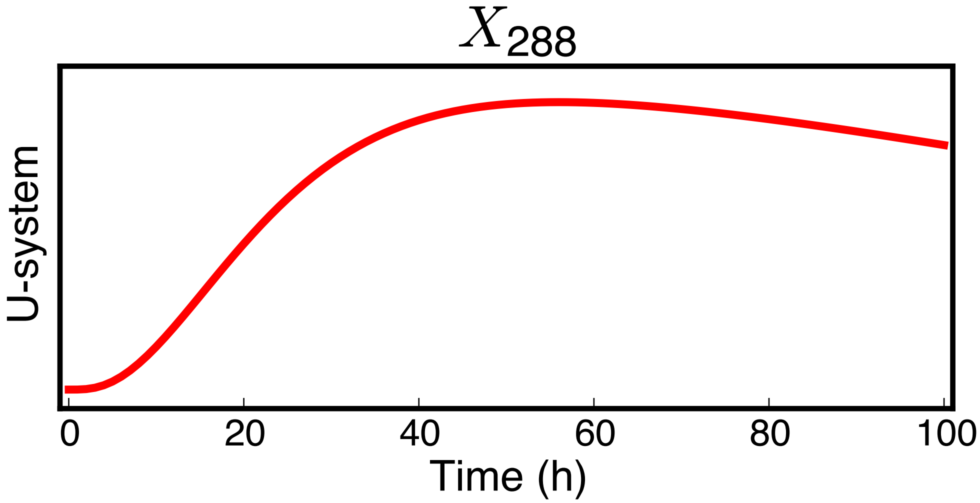

X_{288}
U-system
0
20
40
60
80
100
Time (h)

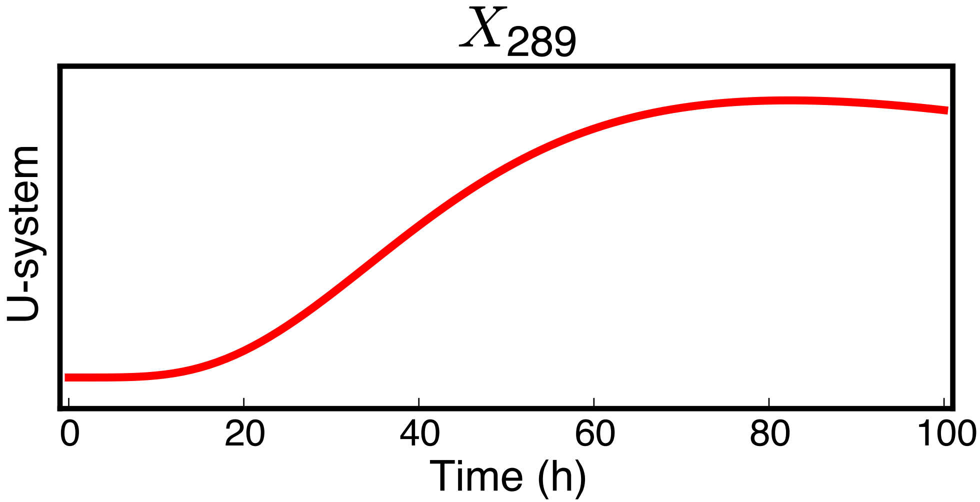

X_{289}
U-system
0
20
40
60
80
100
Time (h)

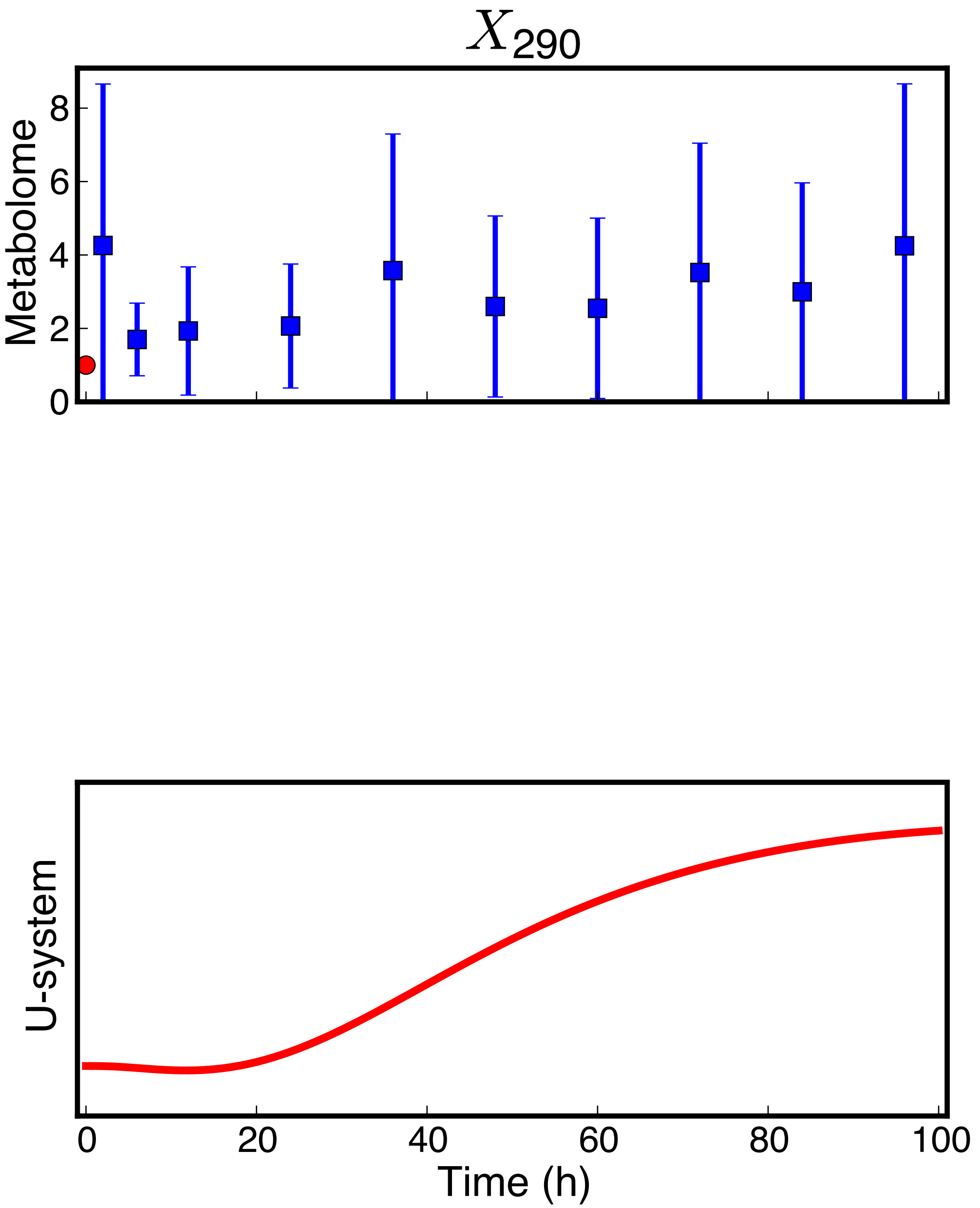

X_{290}
Metabolome
0
2
4
6
8
U-system
0
20
40
60
80
100
Time (h)

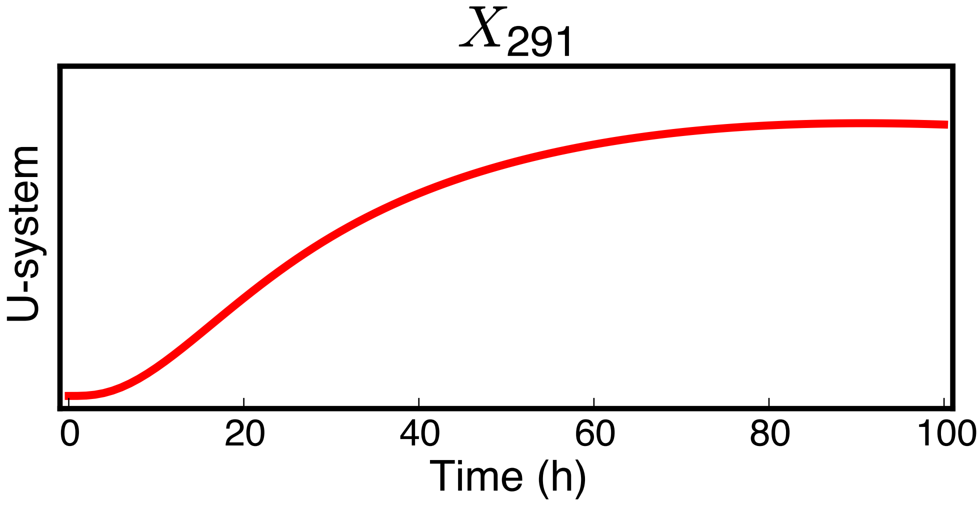

X_{291}
U-system
0
20
40
60
80
100
Time (h)

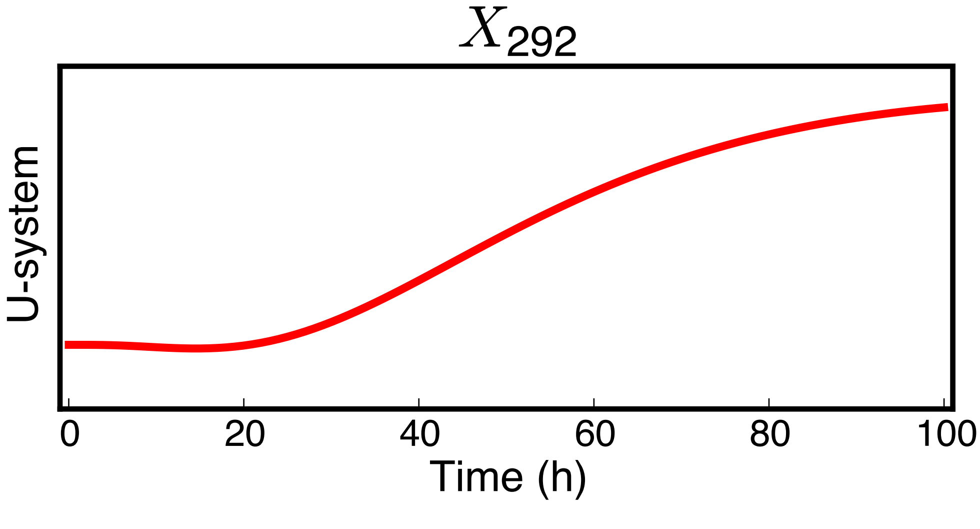

X_{292}
U-system
0
20
40
60
80
100
Time (h)

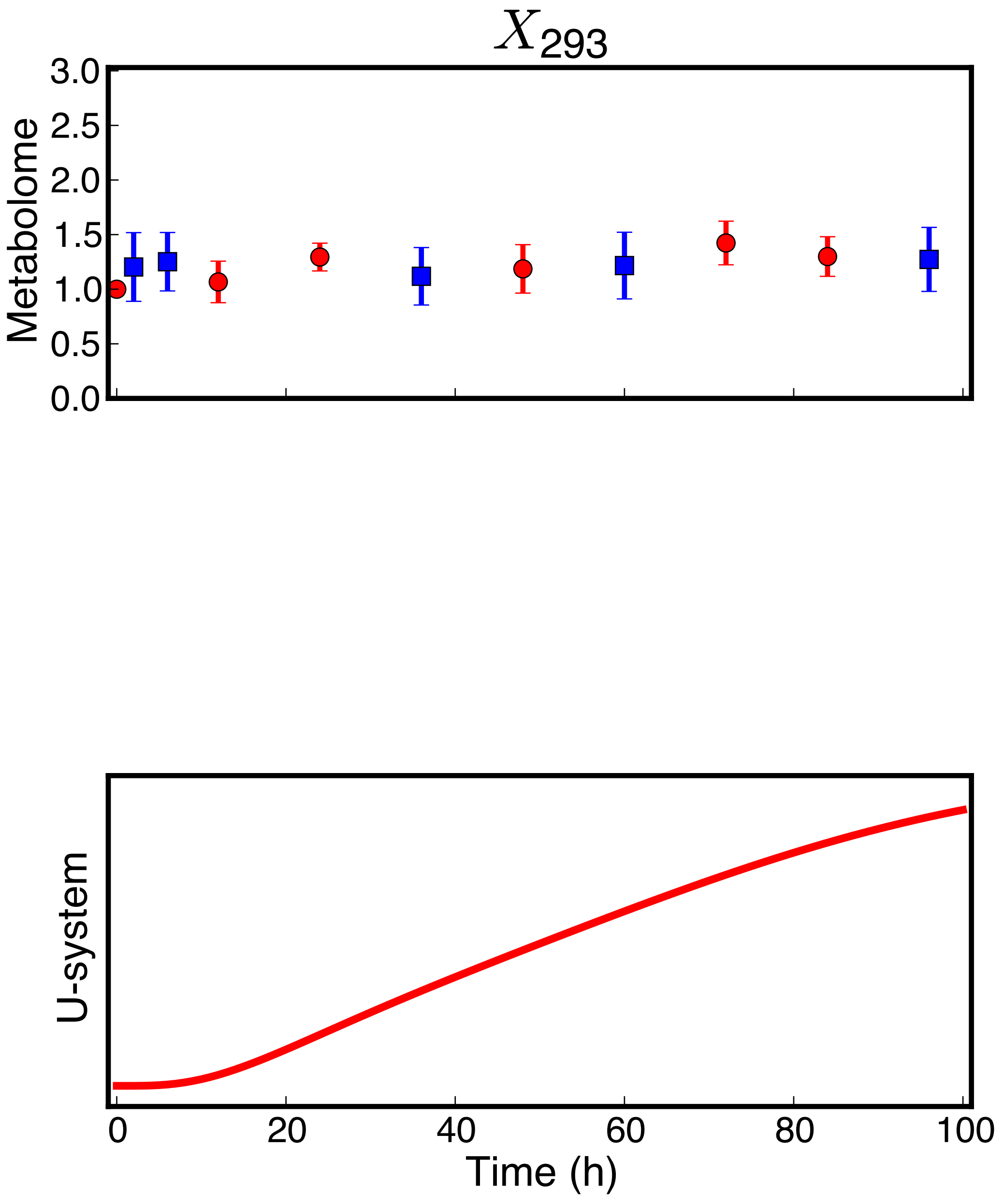
X_{293}
Metabolome
3.0
2.5
2.0
1.5
1.0
0.5
0.0
U-system
0
20
40
60
80
100
Time (h)

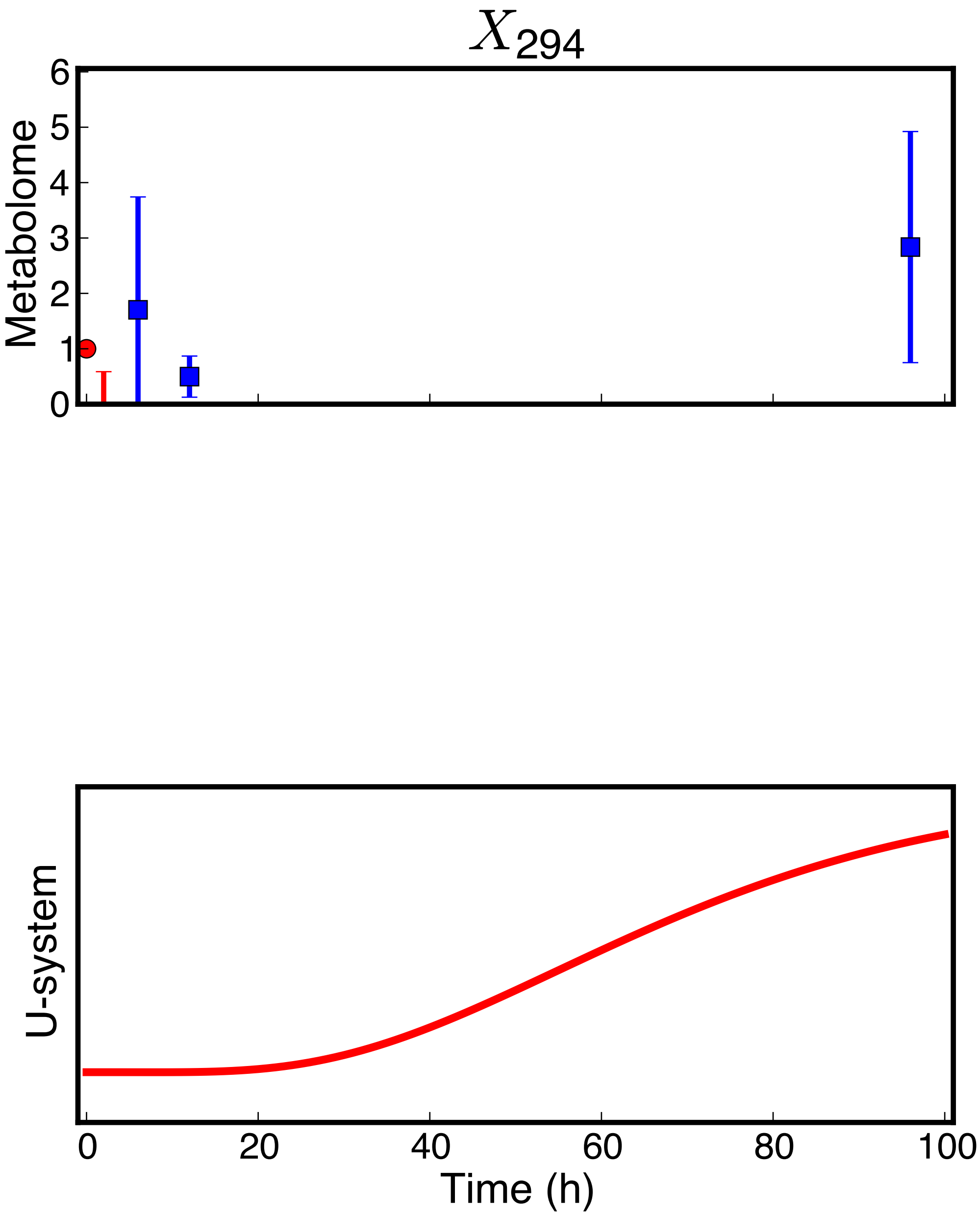

X_{294}
Metabolome
0
1
2
3
4
5
6
U-system
0
20
40
60
80
100
Time (h)

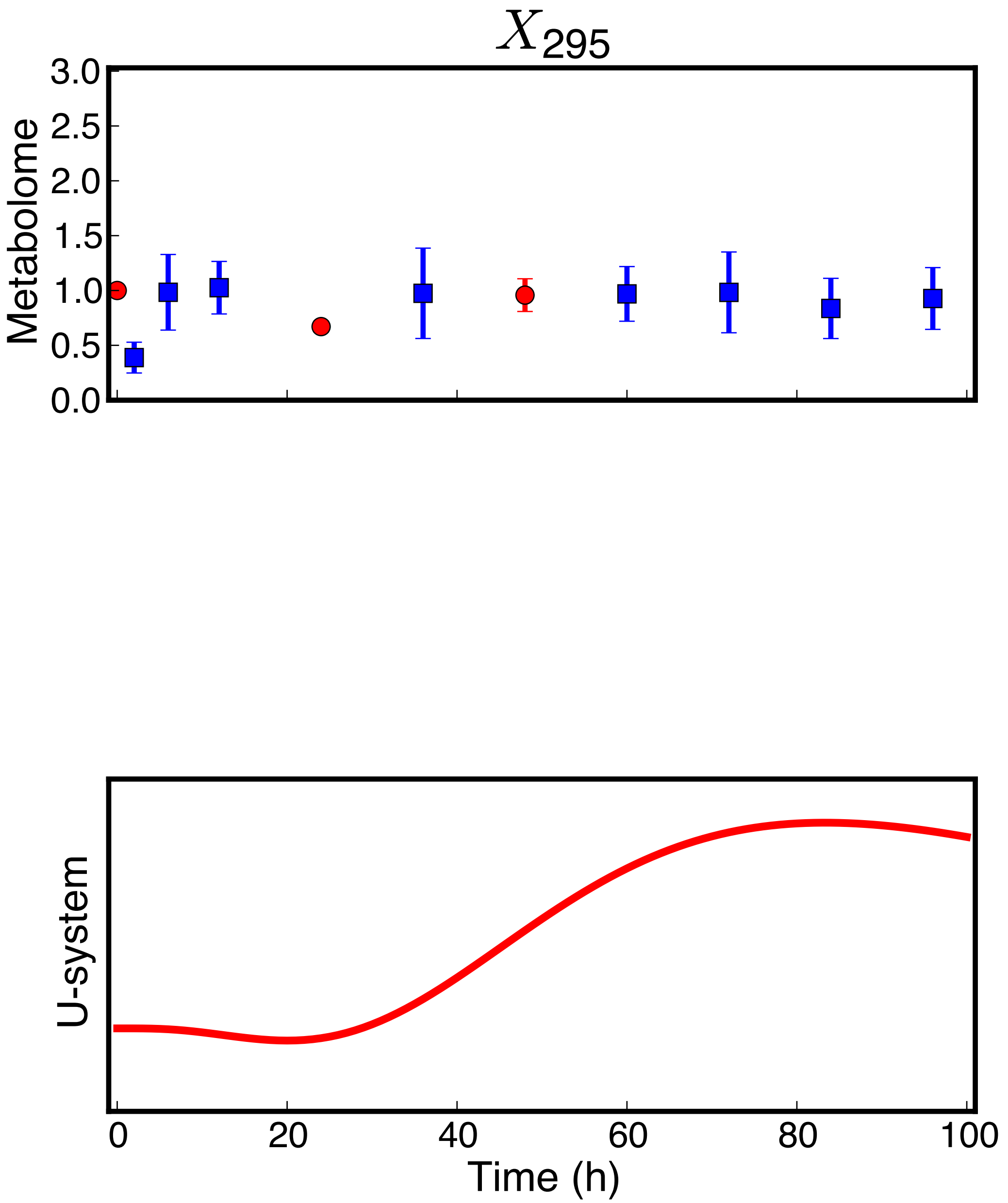
X_{295}
Metabolome
3.0
2.5
2.0
1.5
1.0
0.5
0.0
U-system
0
20
40
60
80
100
Time (h)

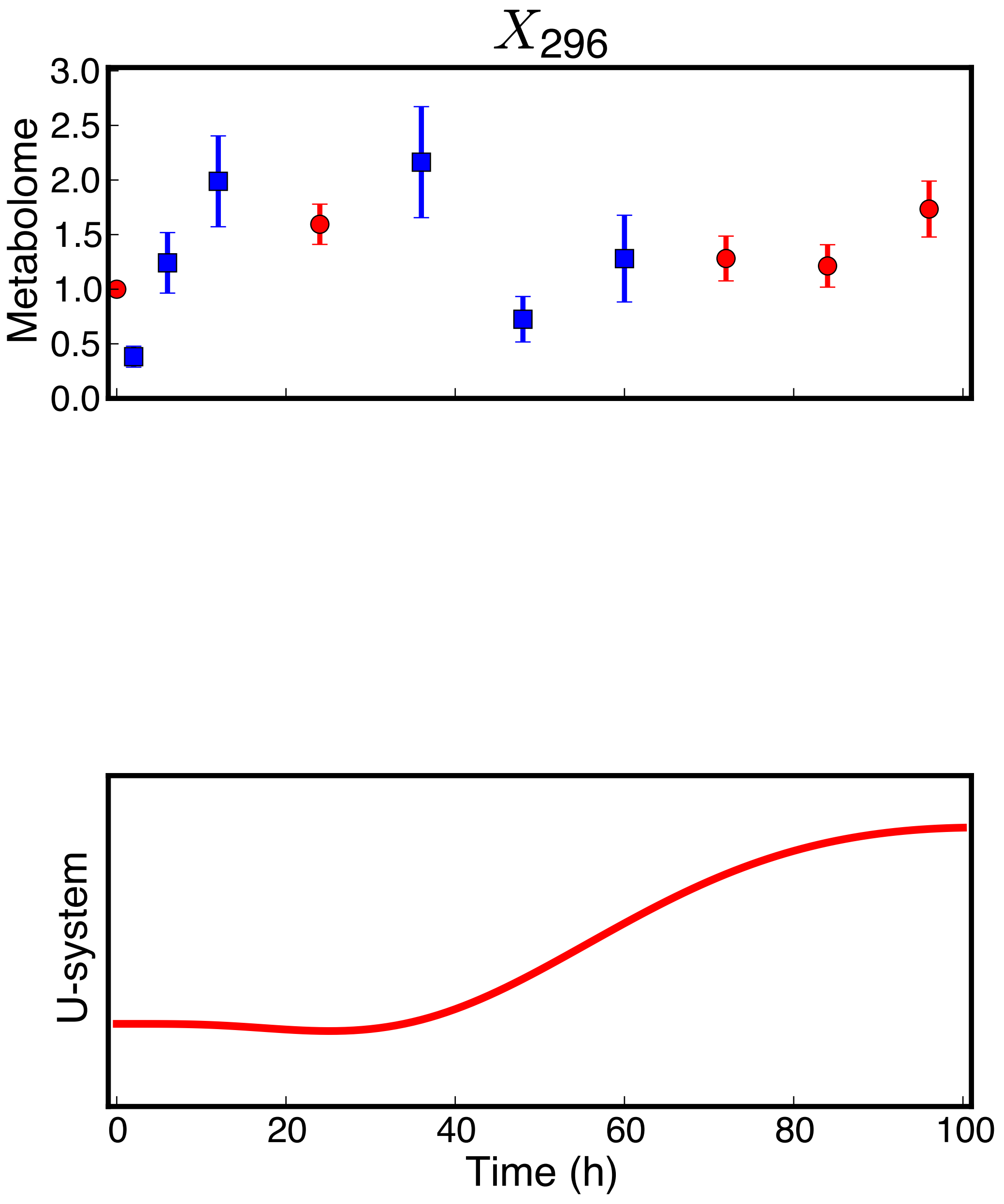

X_{296}
3.0
2.5
2.0
1.5
1.0
0.5
0.0
Metabolome
U-system
0
20
40
60
80
100
Time (h)

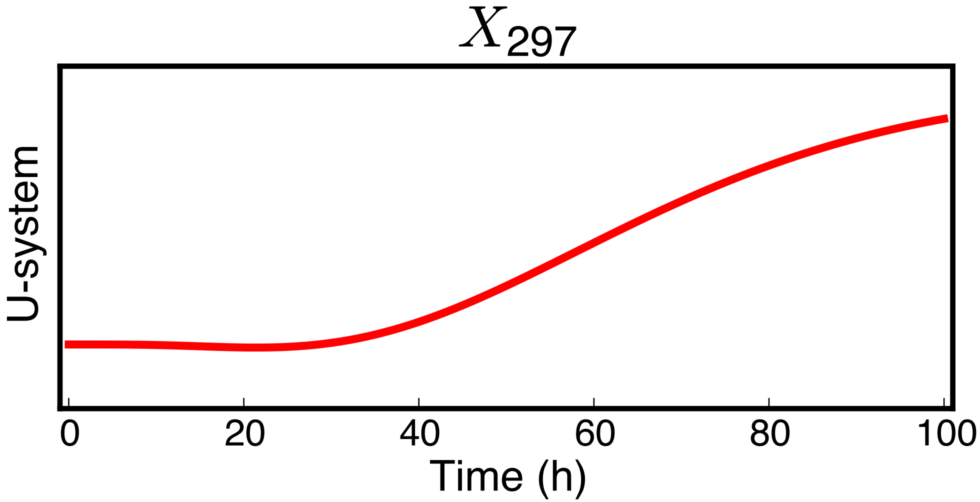

X_{297}
U-system
0
20
40
60
80
100
Time (h)

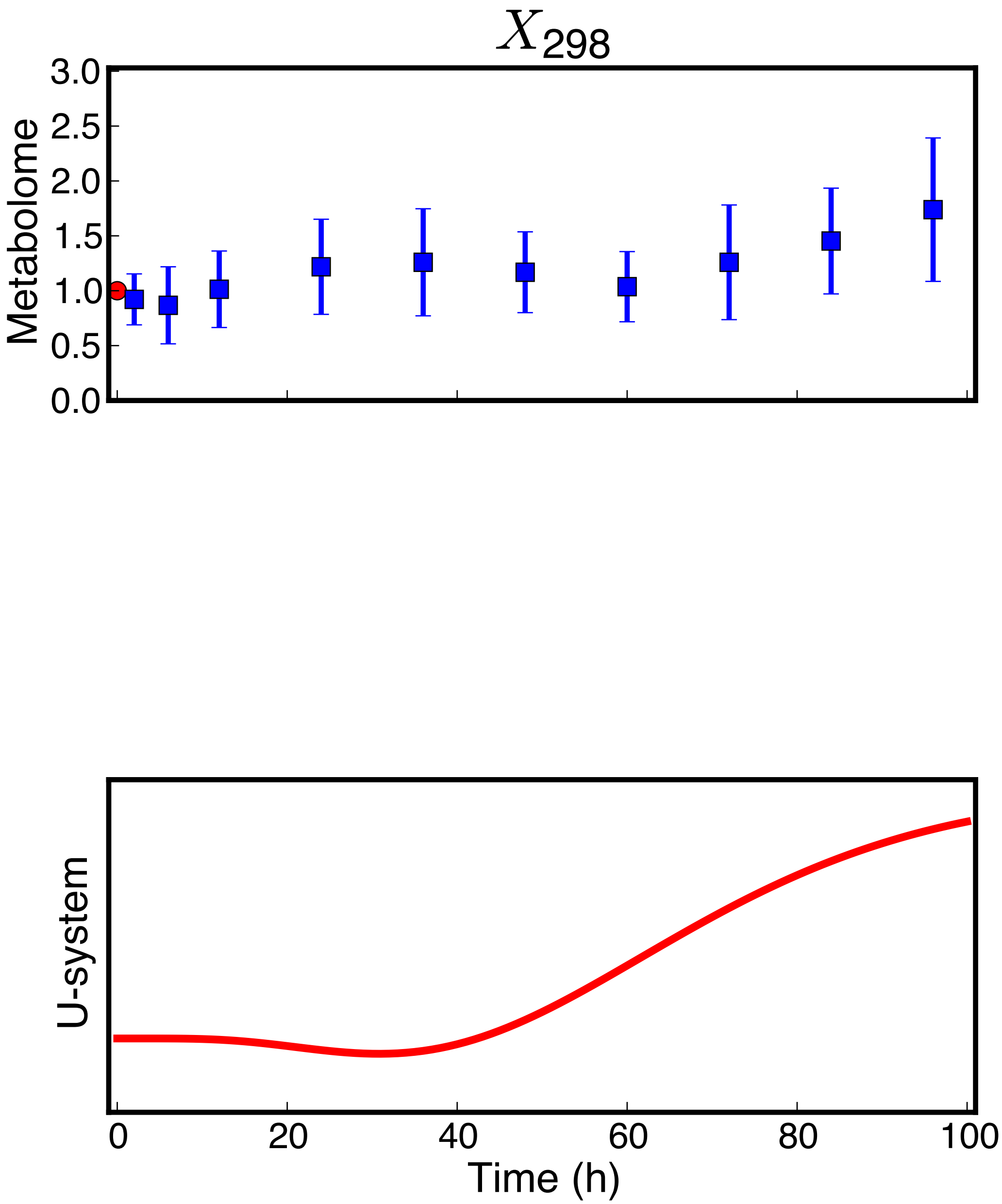
X_{298}
Metabolome
3.0
2.5
2.0
1.5
1.0
0.5
0.0
U-system
0
20
40
60
80
100
Time (h)

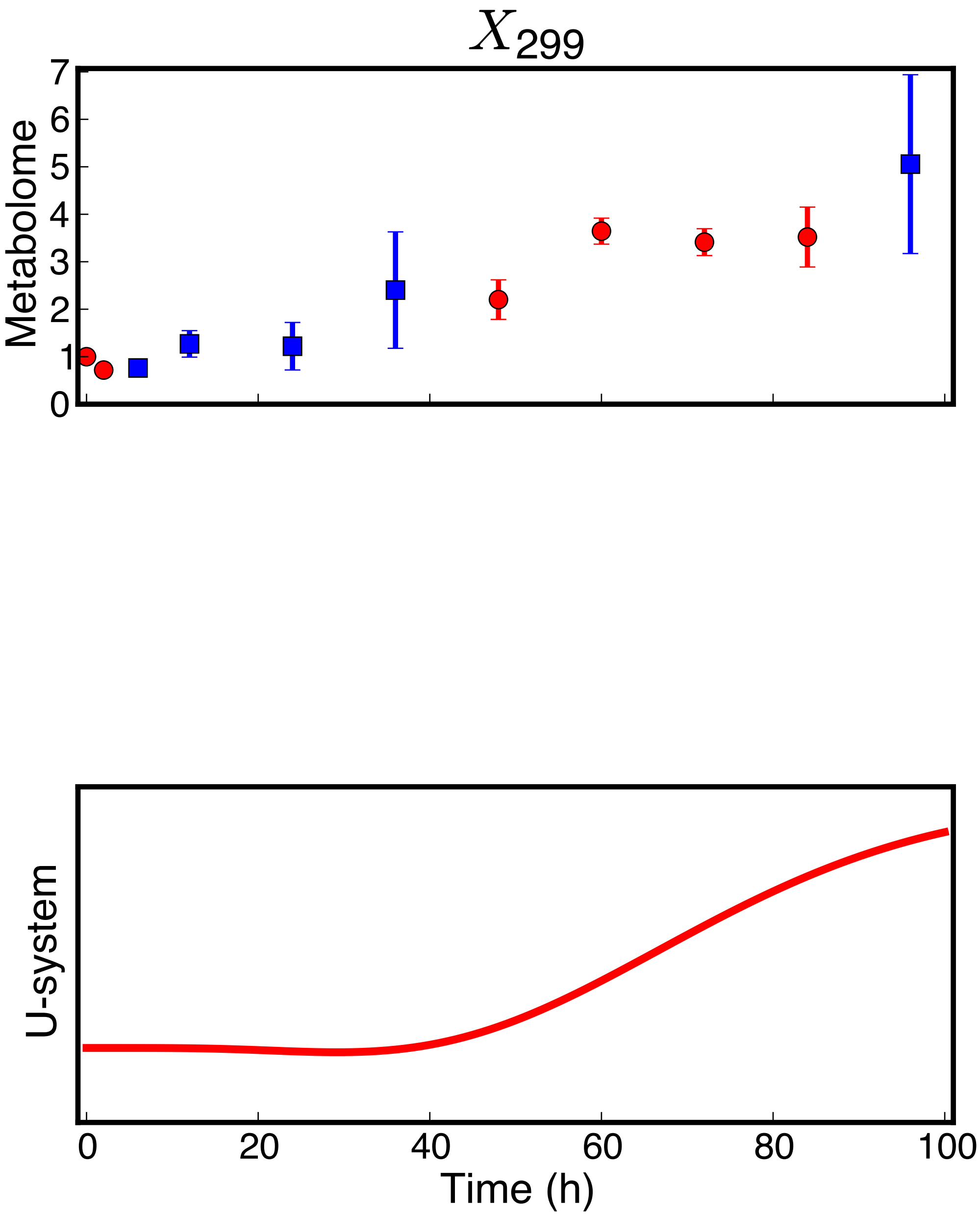

X_{299}
Metabolome
0
1
2
3
4
5
6
7
U-system
0
20
40
60
80
100
Time (h)

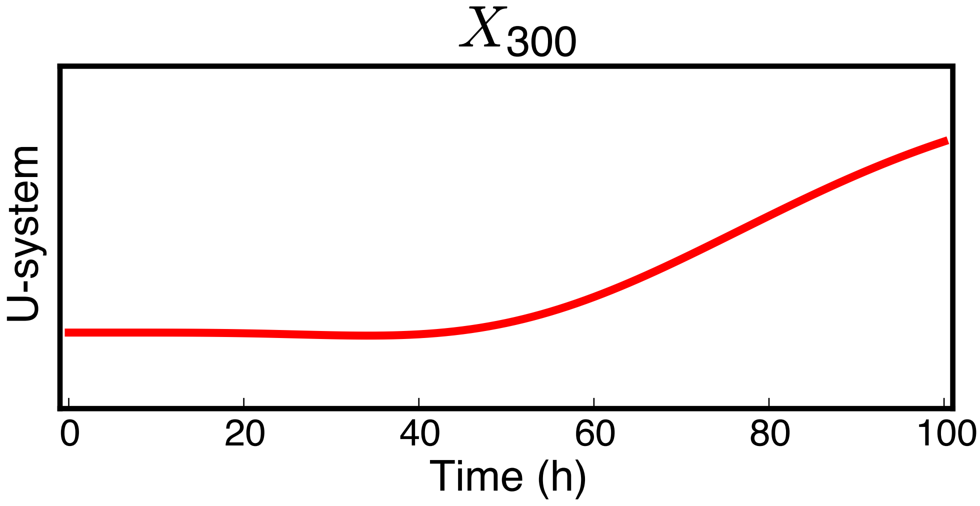

X_{300}
U-system
0
20
40
60
80
100
Time (h)

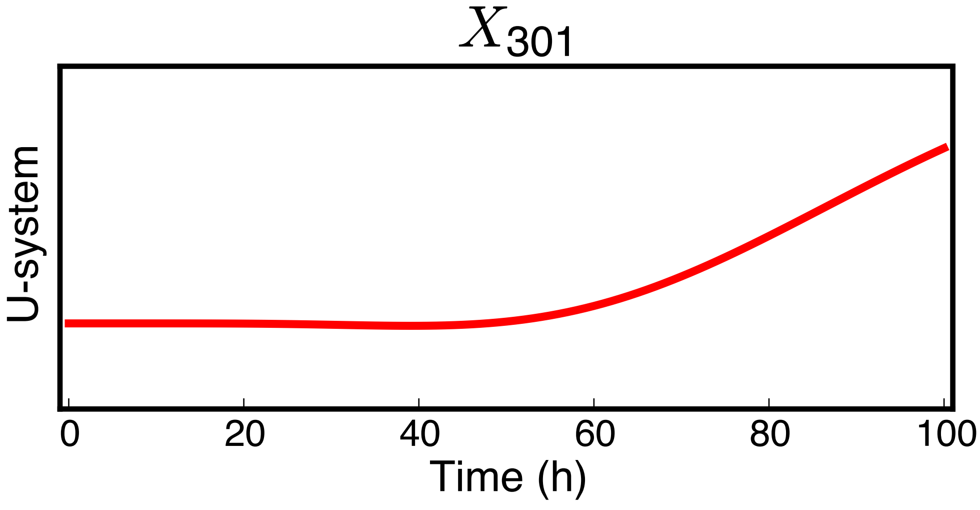
X_{301}
U-system
0
20
40
60
80
100
Time (h)

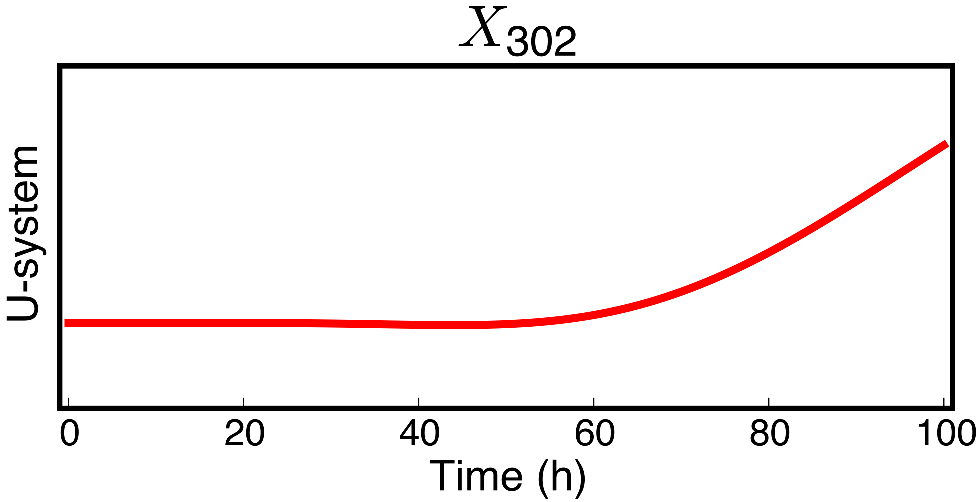

X_{302}
U-system
0
20
40
60
80
100
Time (h)

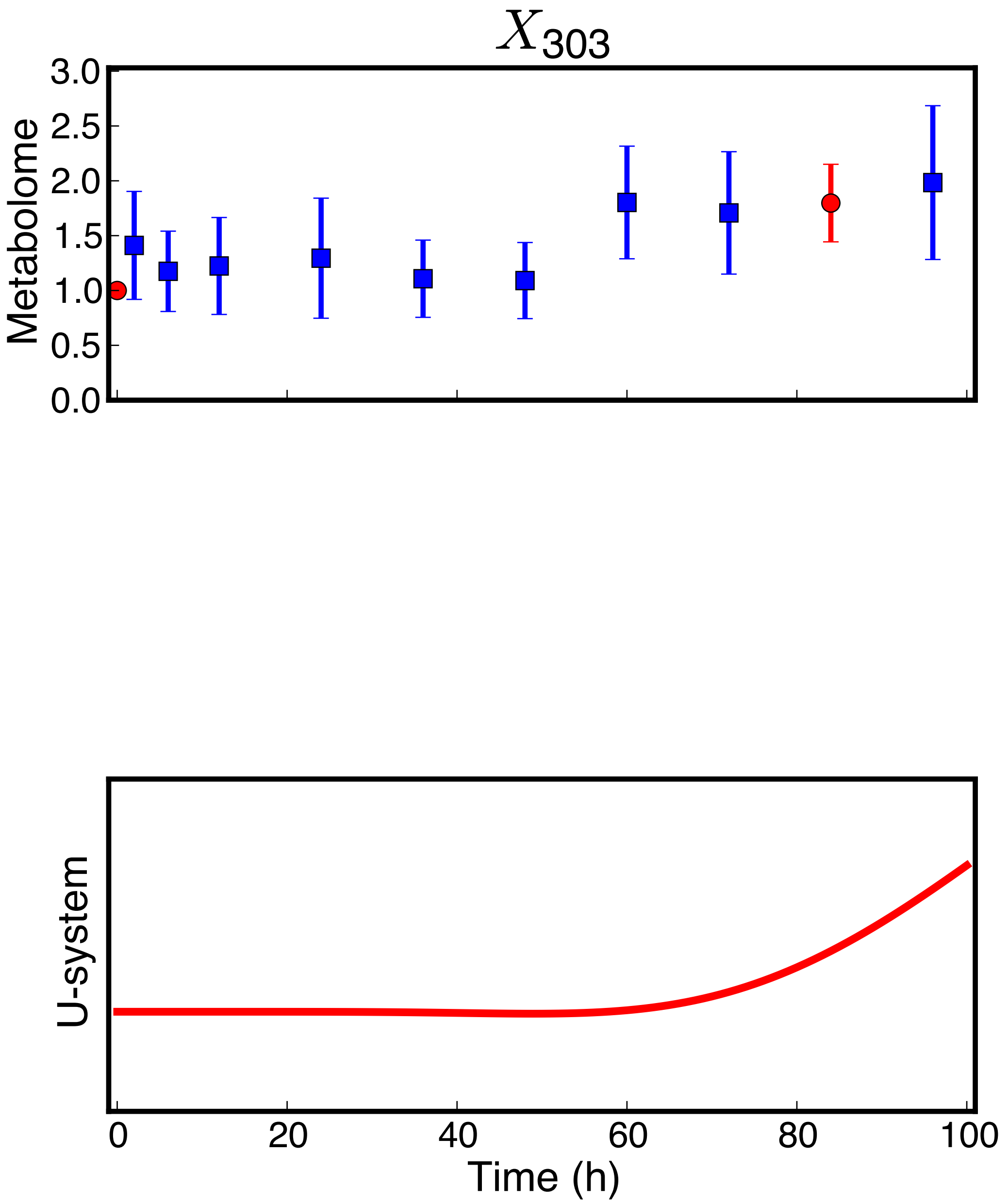

X_{303}
Metabolome
0.0
0.5
1.0
1.5
2.0
2.5
3.0
U-system
0
20
40
60
80
100
Time (h)

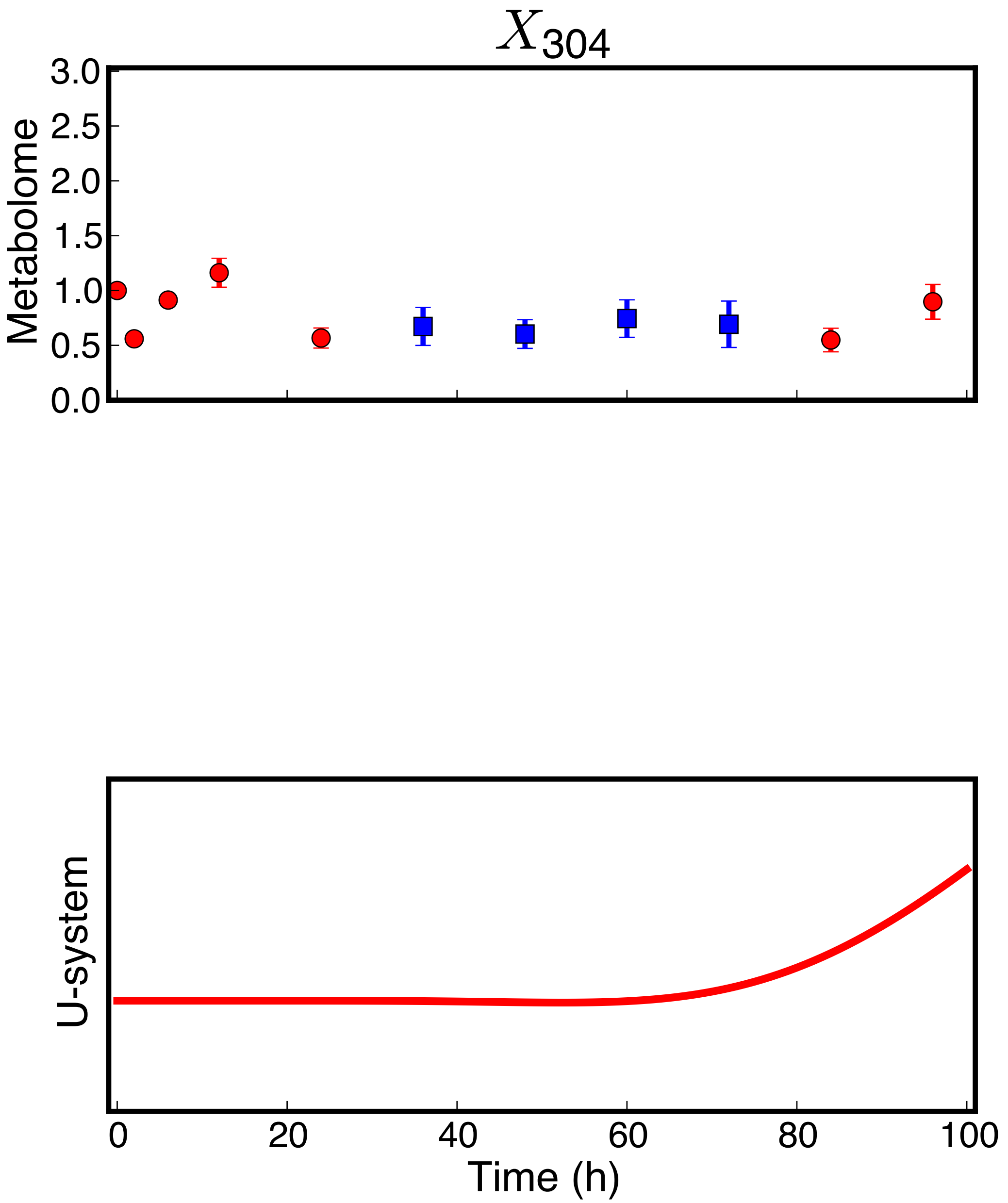
X_{304}
Metabolome
3.0
2.5
2.0
1.5
1.0
0.5
0.0
U-system
0
20
40
60
80
100
Time (h)

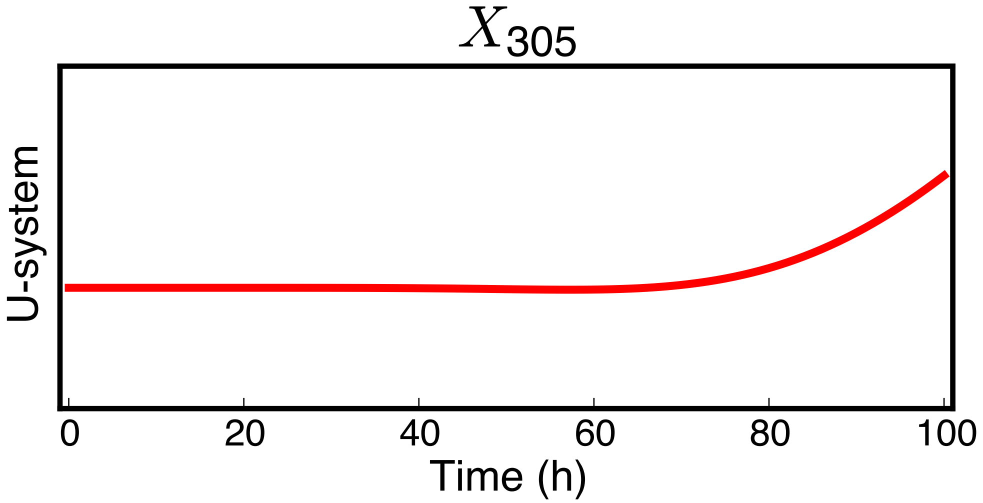

X_{305}
U-system
0
20
40
60
80
100
Time (h)

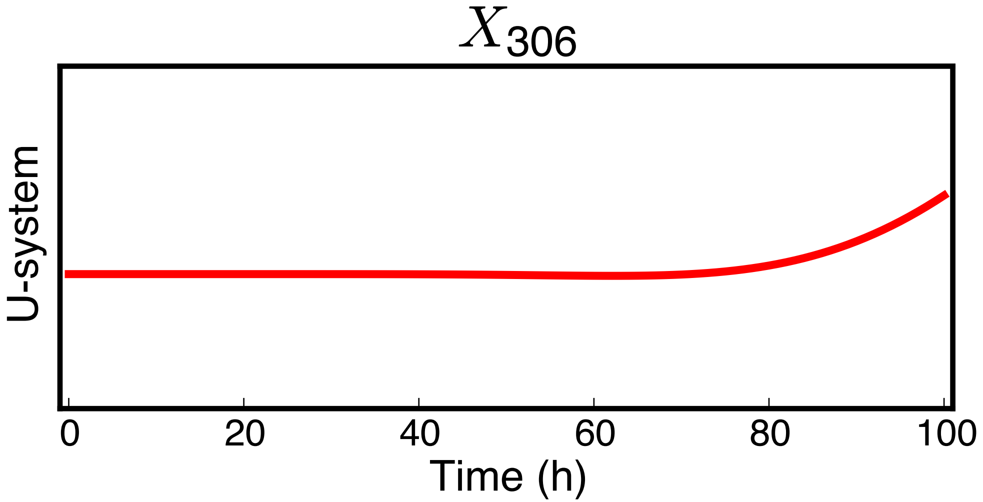

X_{306}
U-system
0
20
40
60
80
100
Time (h)

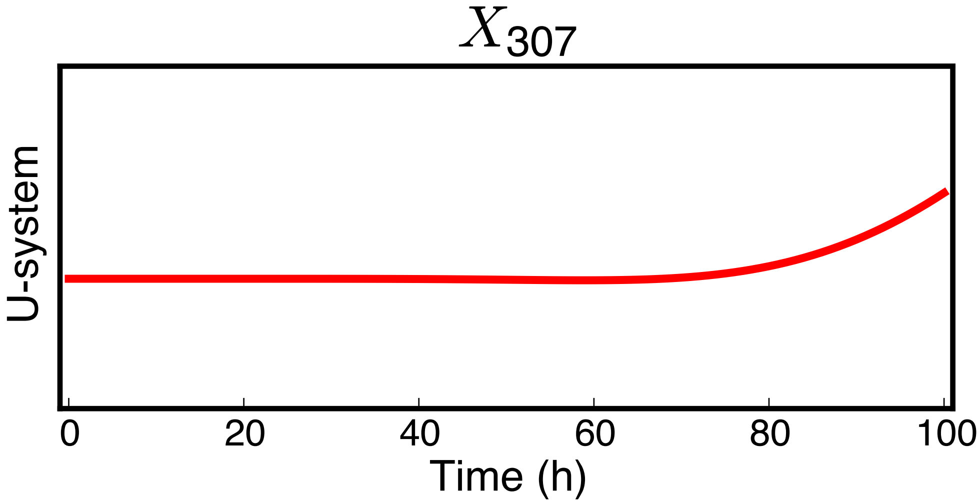

X_{307}
U-system
0
20
40
60
80
100
Time (h)

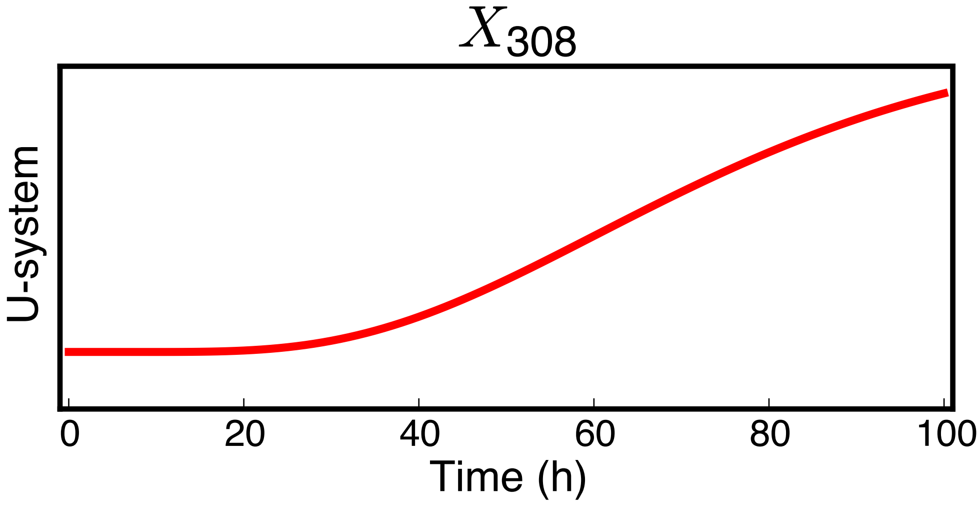
X_{308}
U-system
0
20
40
60
80
100
Time (h)

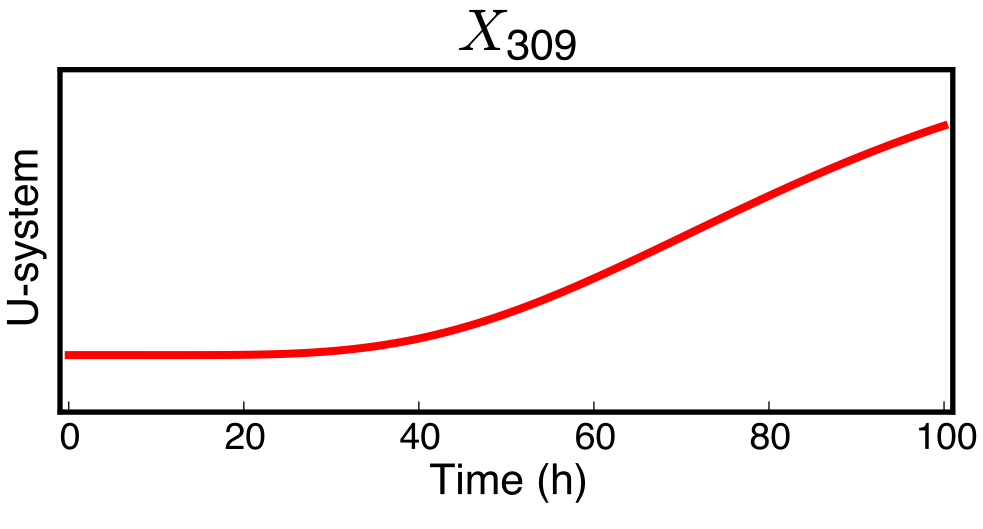
X_{309}
U-system
0
20
40
60
80
100
Time (h)

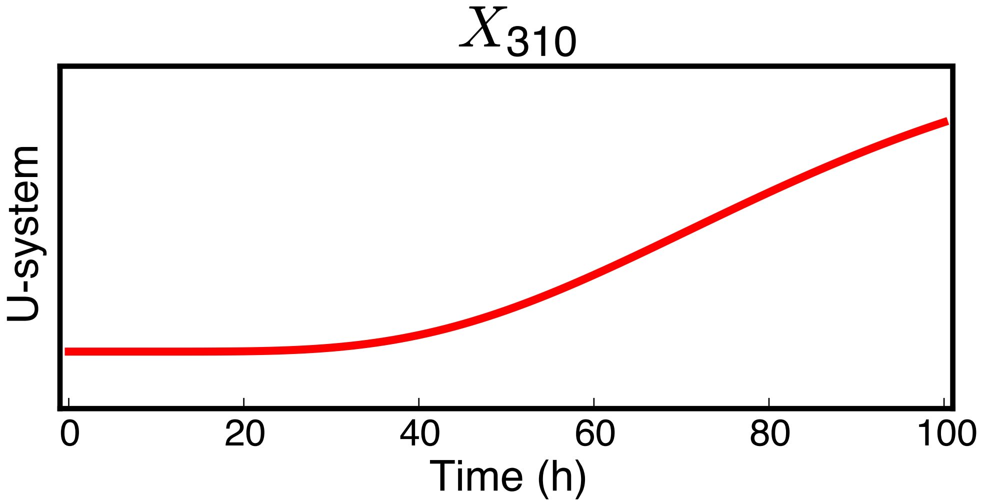
X_{310}
U-system
0
20
40
60
80
100
Time (h)

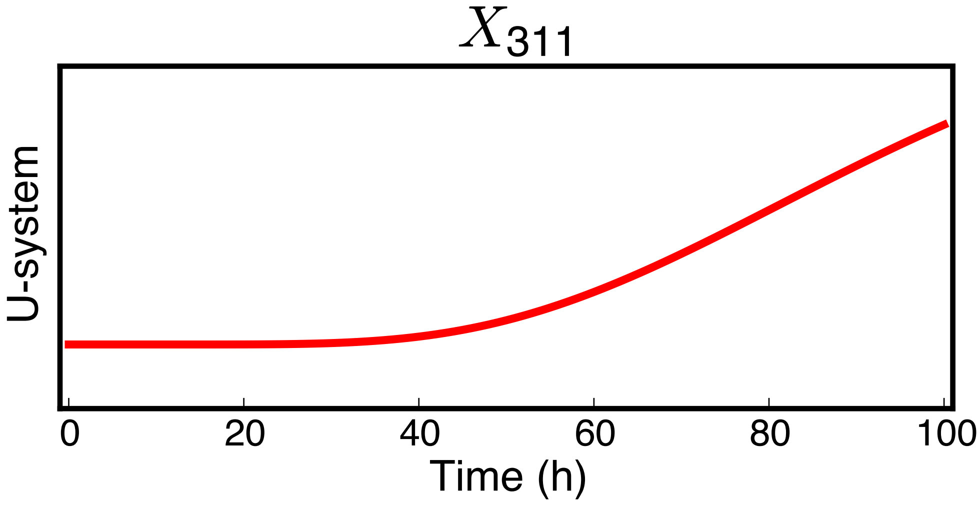

X_{311}
U-system
0
20
40
60
80
100
Time (h)

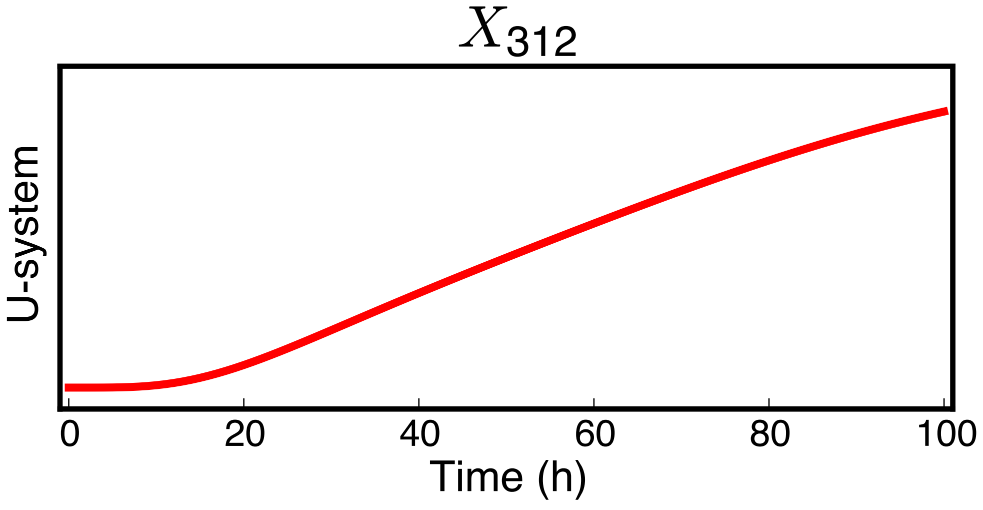
X_{312}
U-system
0
20
40
60
80
100
Time (h)

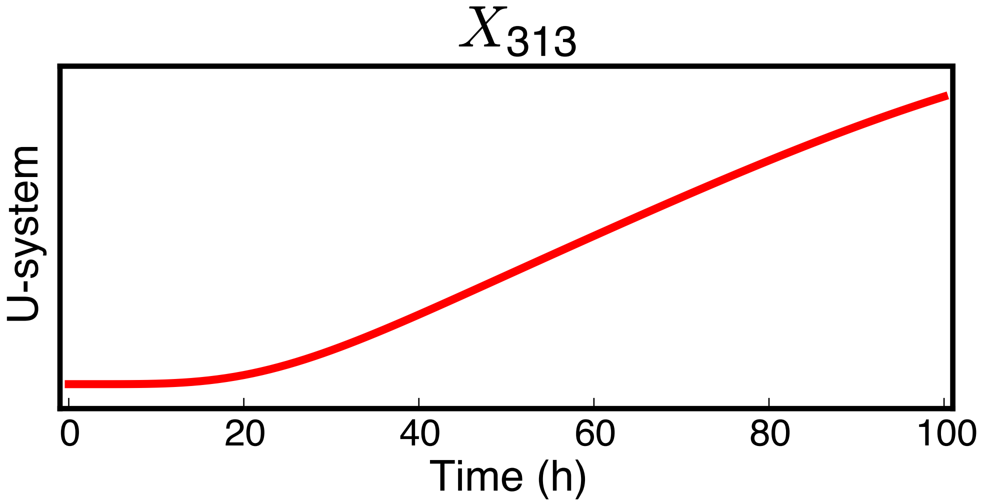
X_{313}
U-system
0
20
40
60
80
100
Time (h)

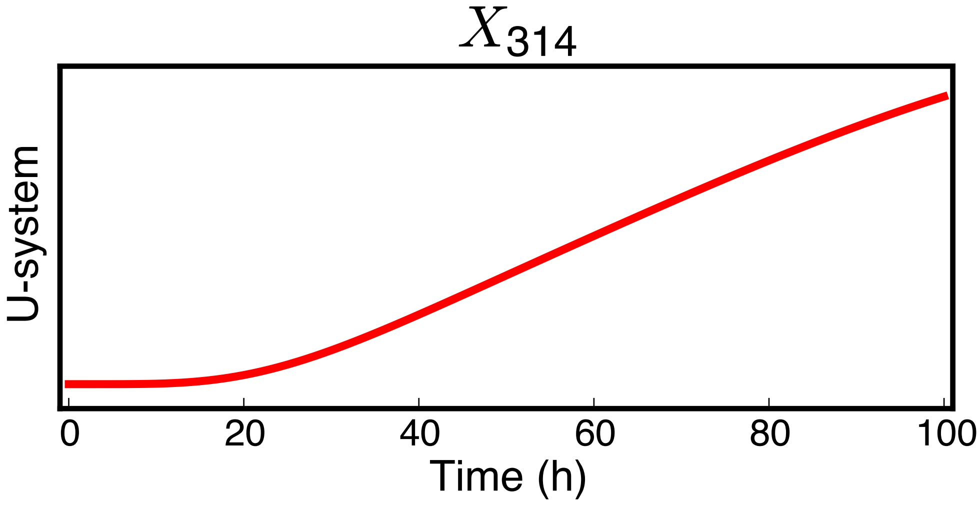

X_{314}
U-system
0
20
40
60
80
100
Time (h)

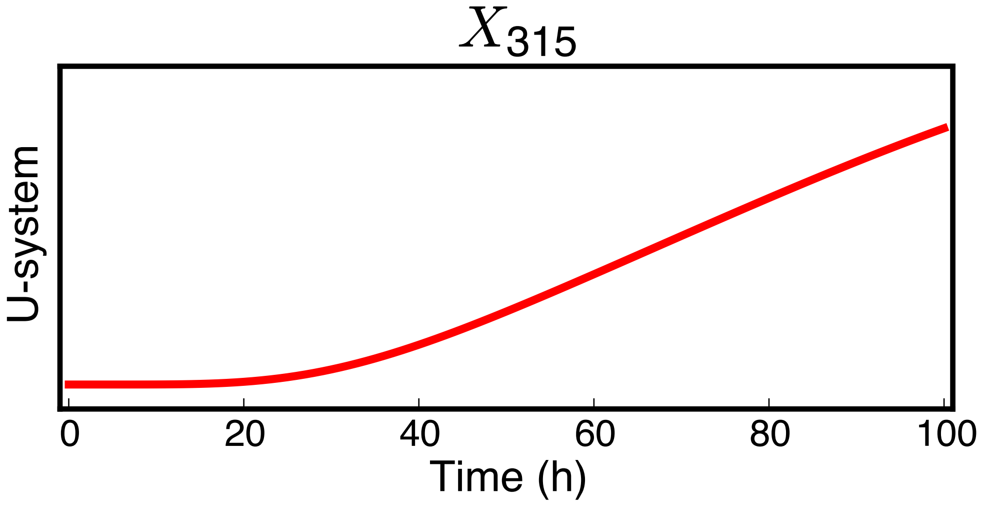

X_{315}
U-system
0
20
40
60
80
100
Time (h)

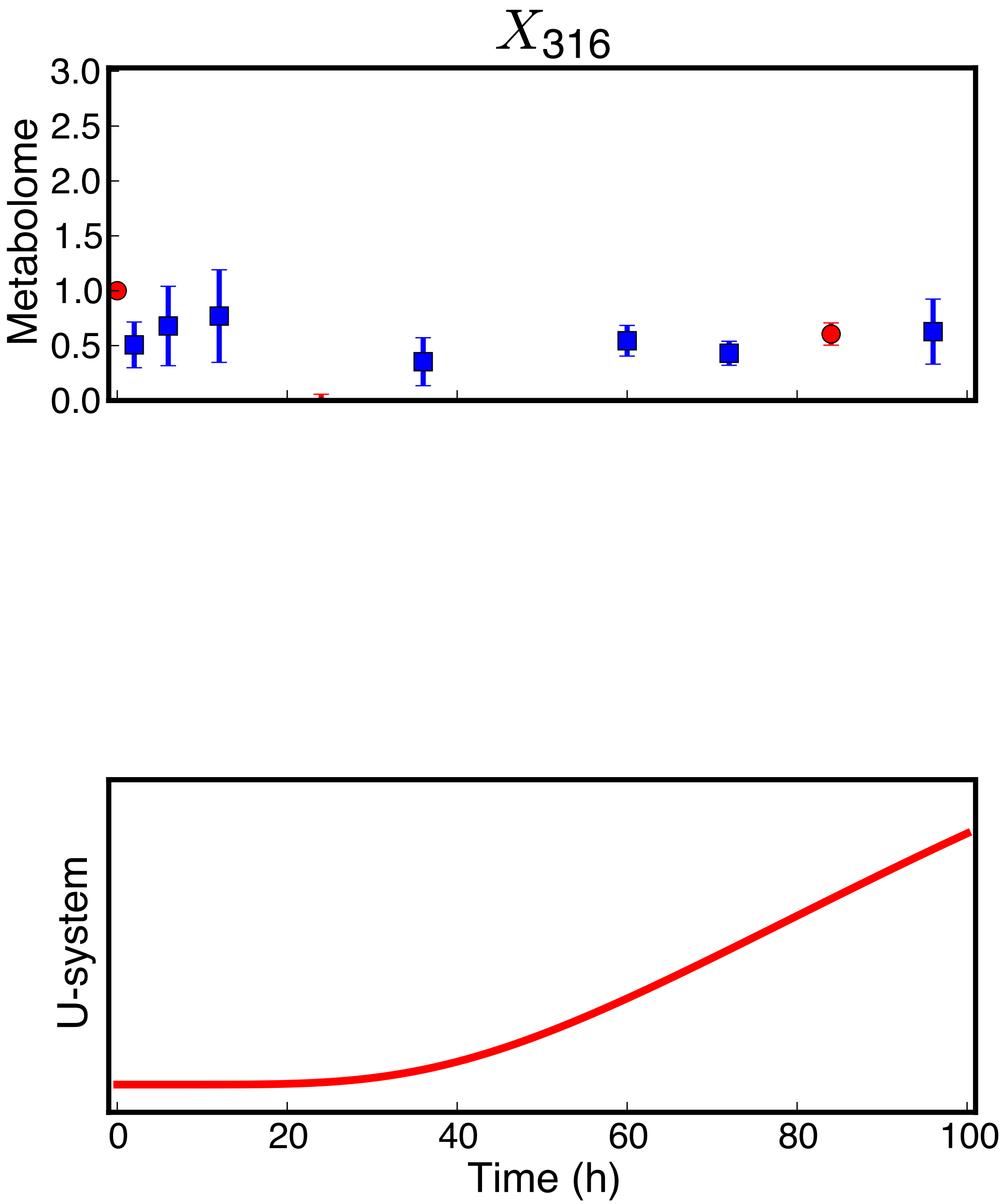

X_{316}
Metabolome
3.0
2.5
2.0
1.5
1.0
0.5
0.0
U-system
0
20
40
60
80
100
Time (h)

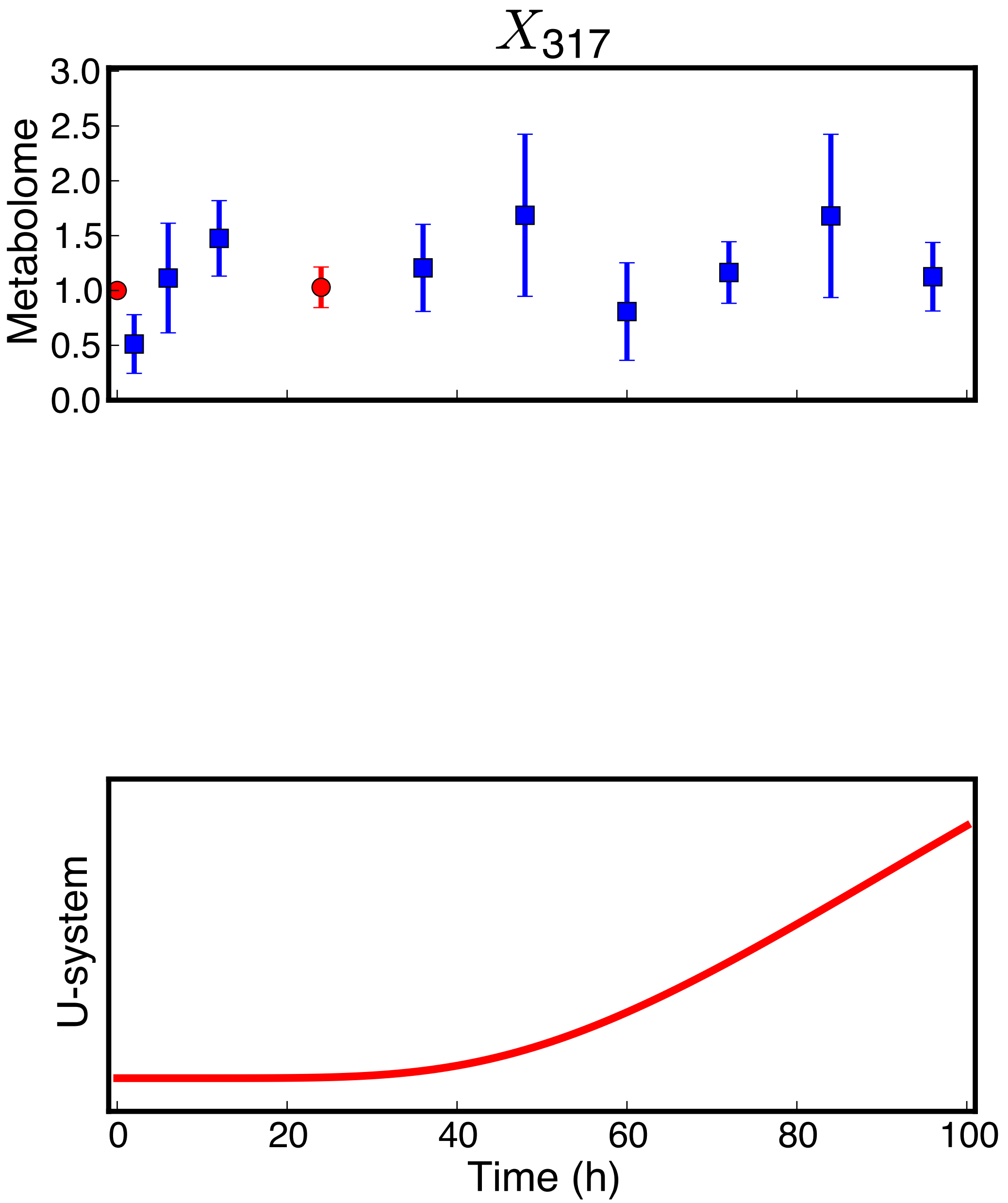

X_{317}
Metabolome
3.0
2.5
2.0
1.5
1.0
0.5
0.0
U-system
0
20
40
60
80
100
Time (h)

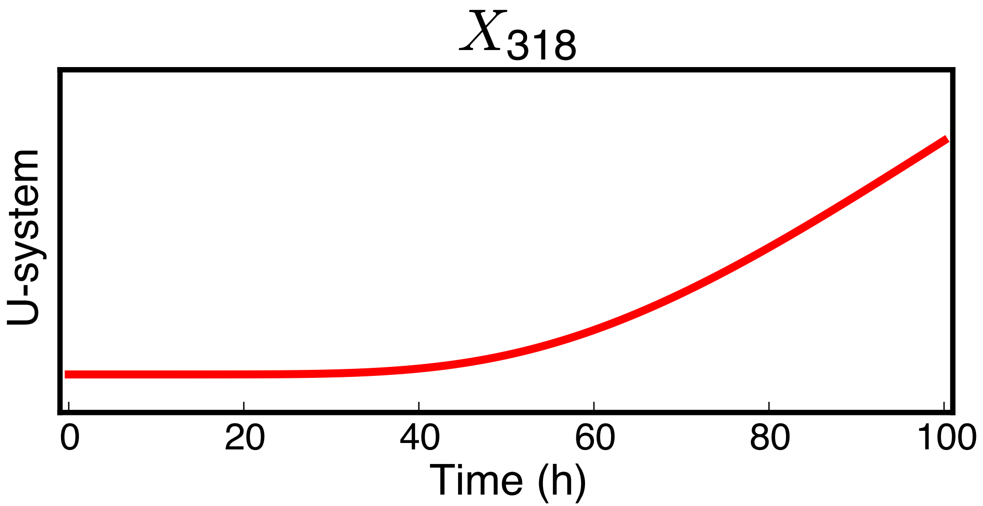
X_{318}
U-system
0
20
40
60
80
100
Time (h)

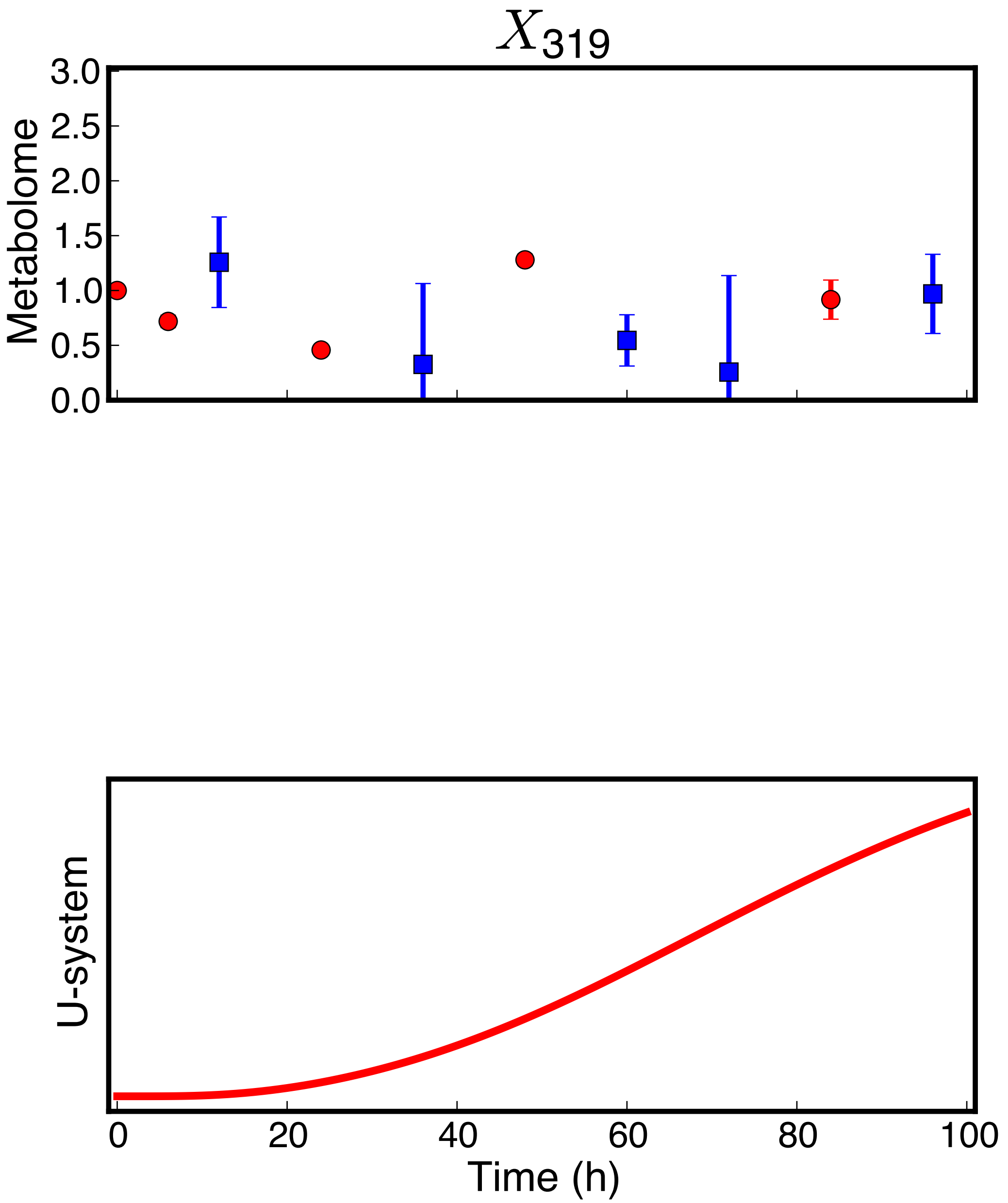

X_{319}
Metabolome
3.0
2.5
2.0
1.5
1.0
0.5
0.0
U-system
0
20
40
60
80
100
Time (h)

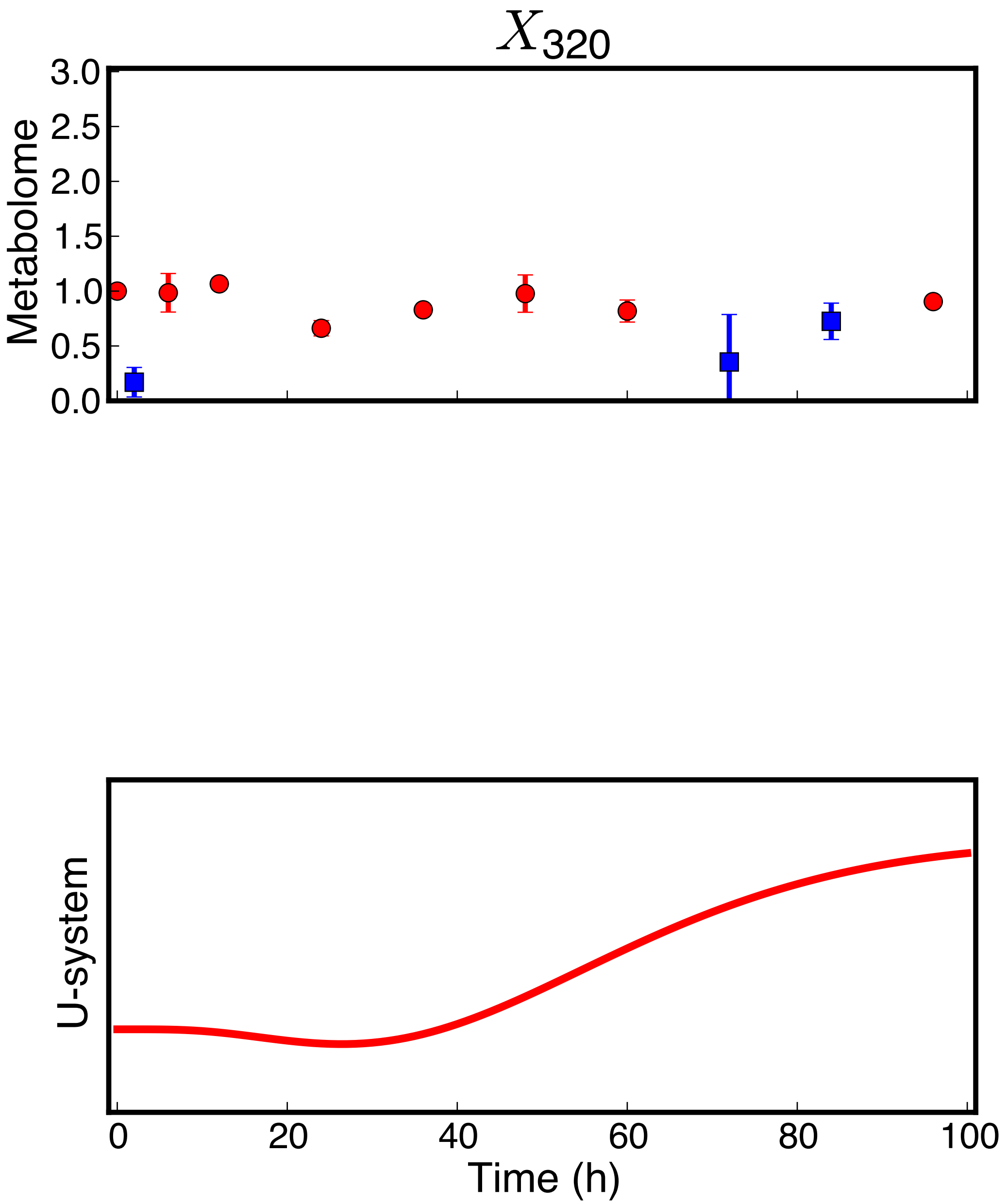

X_{320}
Metabolome
3.0
2.5
2.0
1.5
1.0
0.5
0.0
U-system
0
20
40
60
80
100
Time (h)

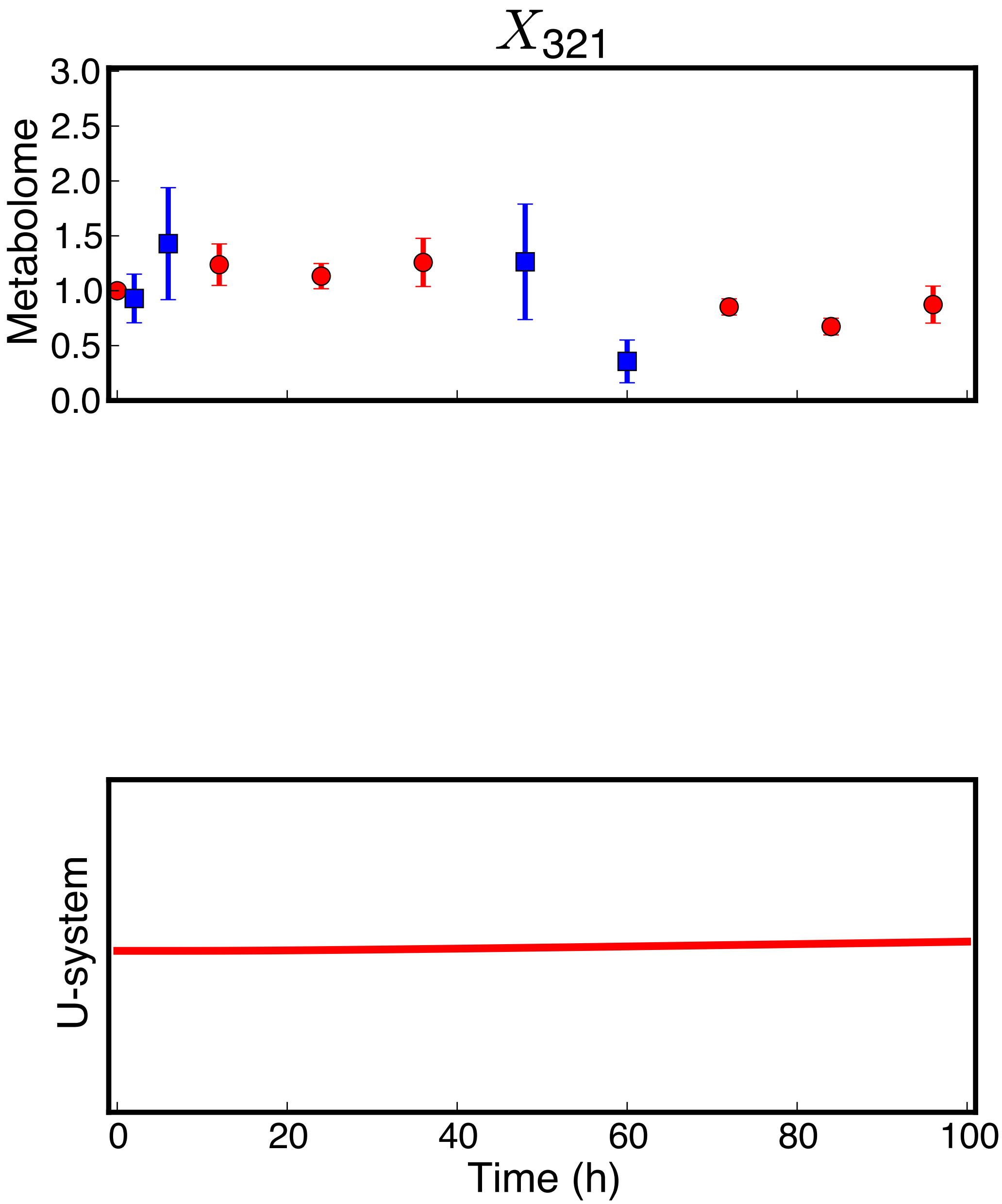
X_{321}
Metabolome
3.0
2.5
2.0
1.5
1.0
0.5
0.0
U-system
0
20
40
60
80
100
Time (h)

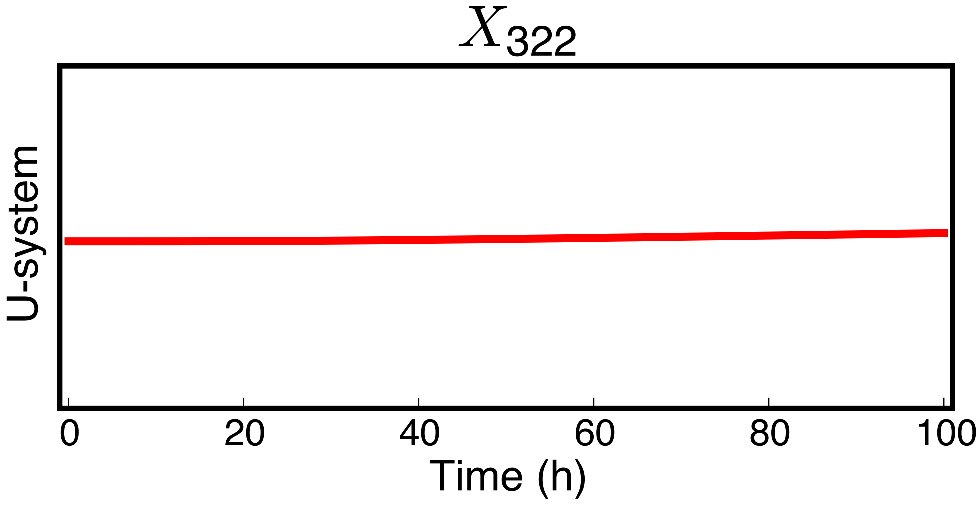

X_{322}
U-system
0
20
40
60
80
100
Time (h)

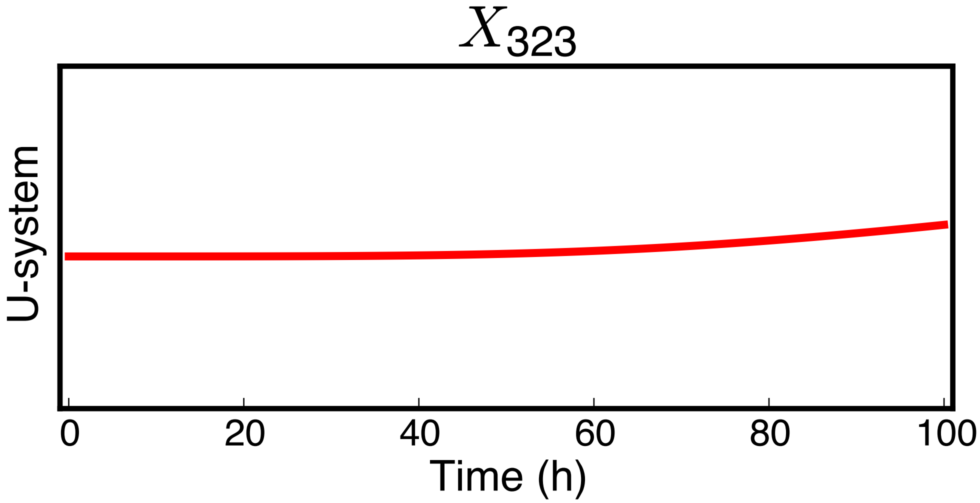
X_{323}
U-system
0
20
40
60
80
100
Time (h)

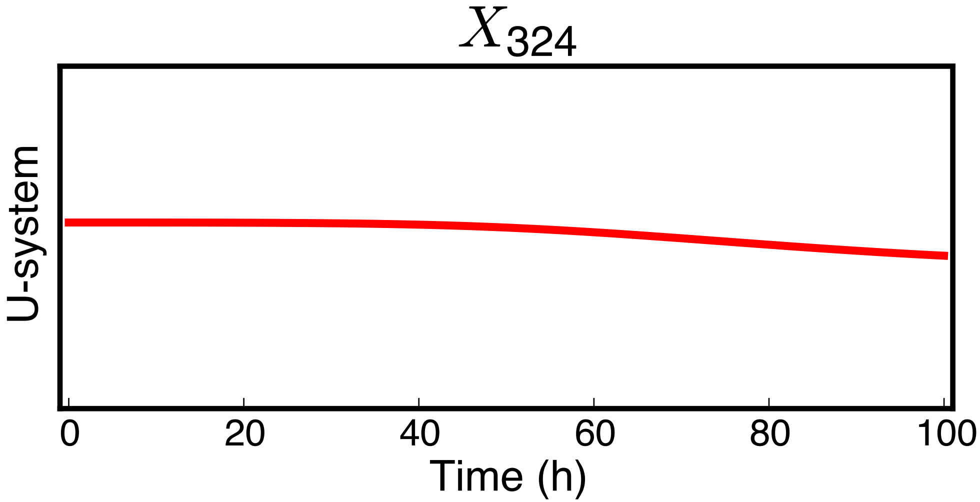
X_{324}
U-system
0
20
40
60
80
100
Time (h)

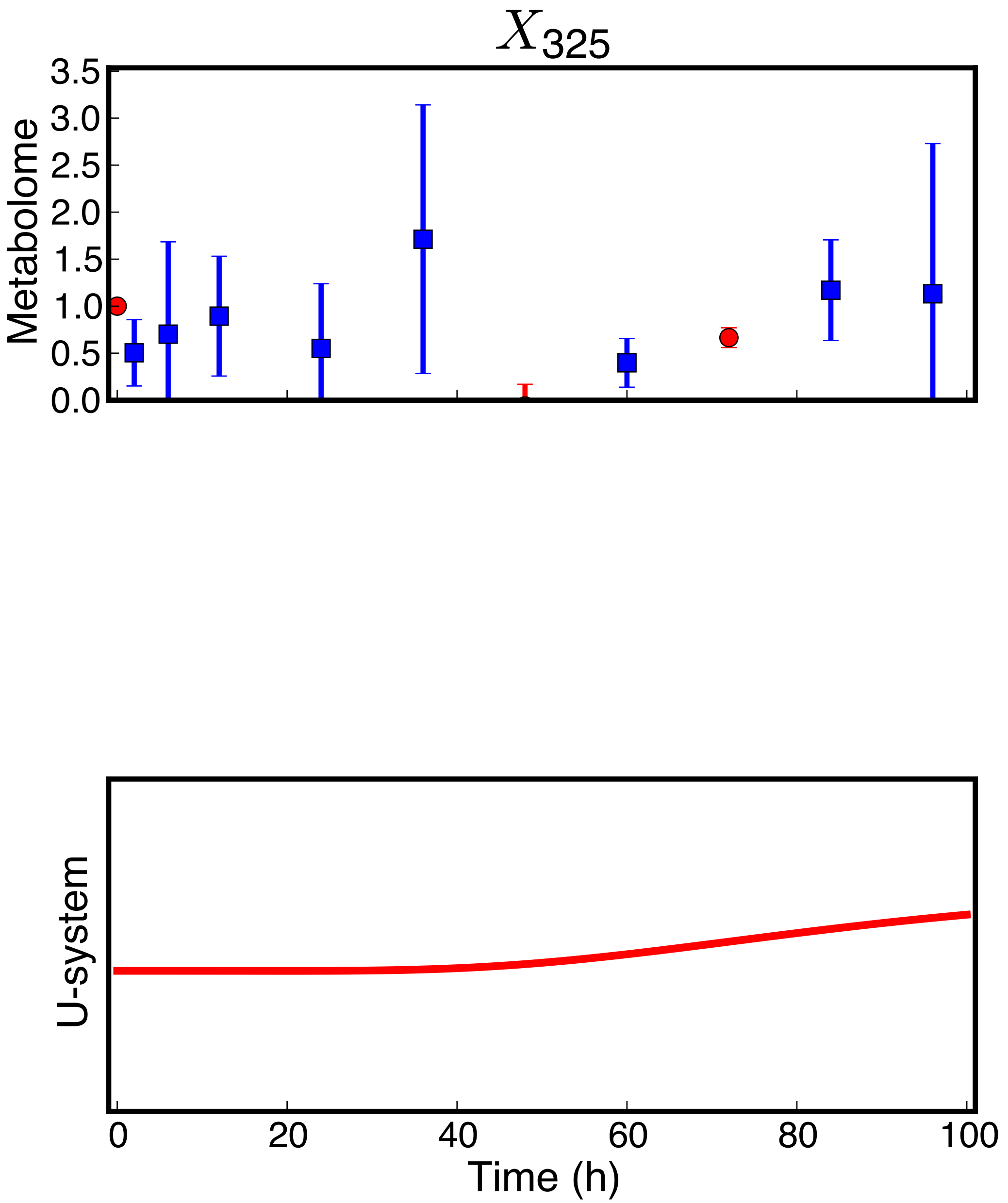

X_{325}
Metabolome
3.5
3.0
2.5
2.0
1.5
1.0
0.5
0.0
U-system
0
20
40
60
80
100
Time (h)

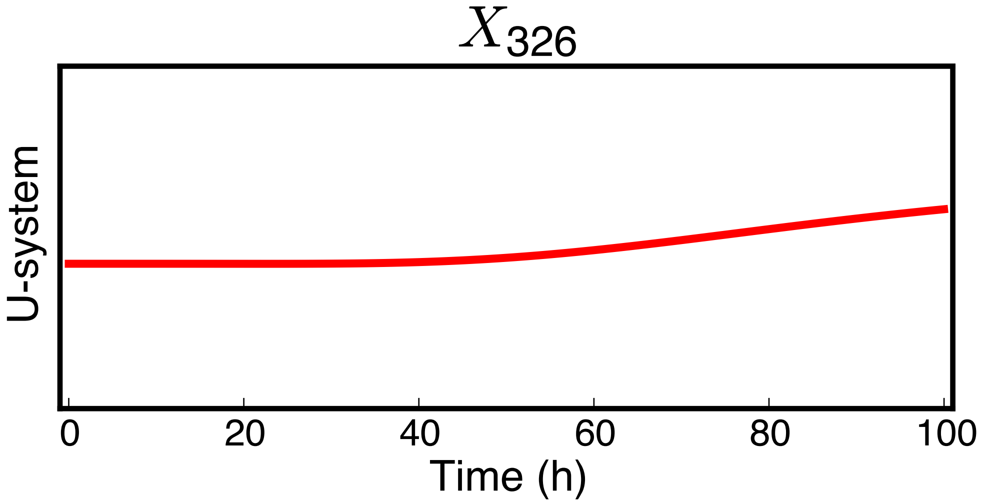
X_{326}
U-system
0
20
40
60
80
100
Time (h)

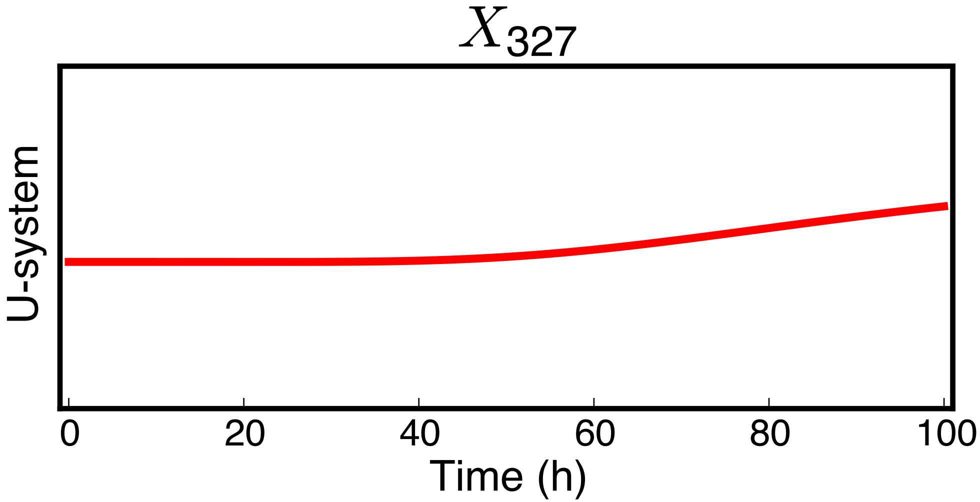

X_{327}
U-system
0
20
40
60
80
100
Time (h)

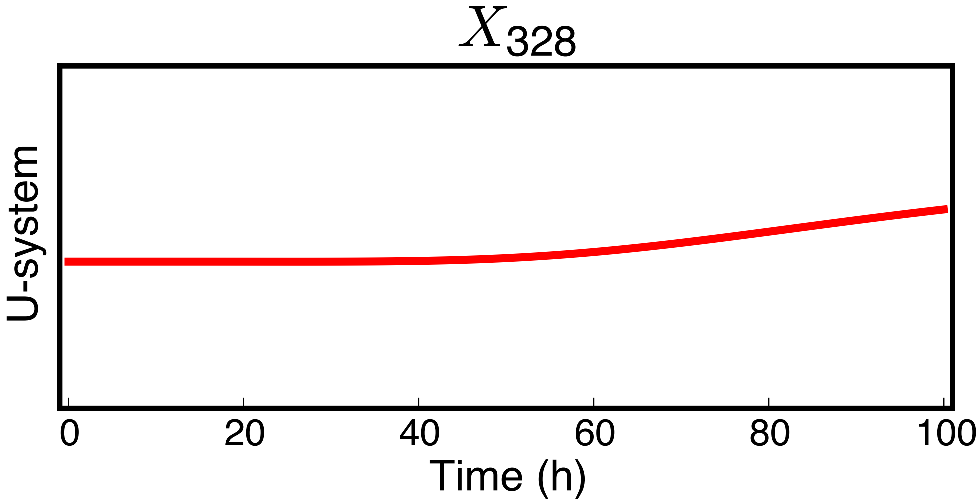

X_{328}
U-system
0
20
40
60
80
100
Time (h)

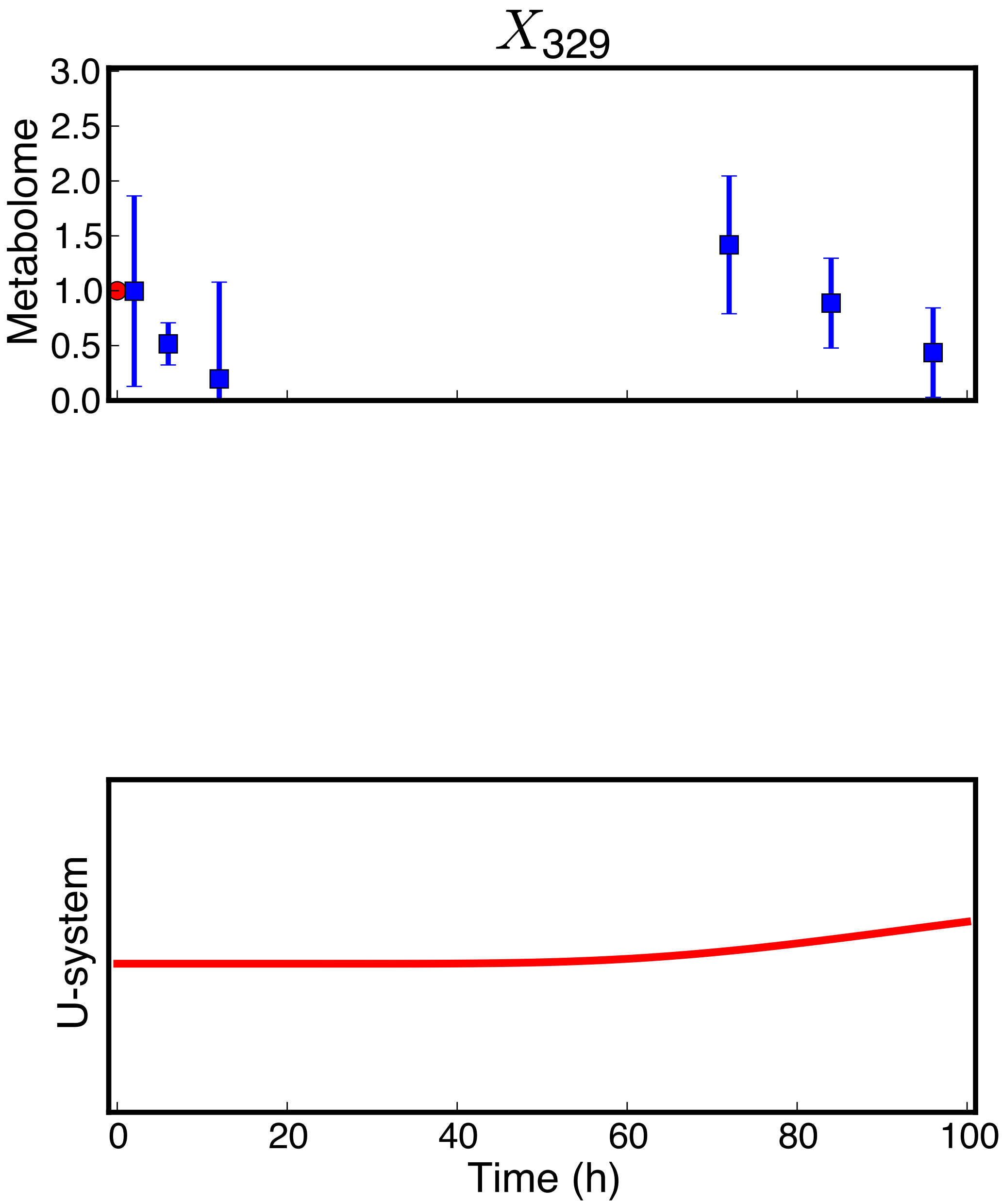

X_{329}
Metabolome
3.0
2.5
2.0
1.5
1.0
0.5
0.0
U-system
0
20
40
60
80
100
Time (h)

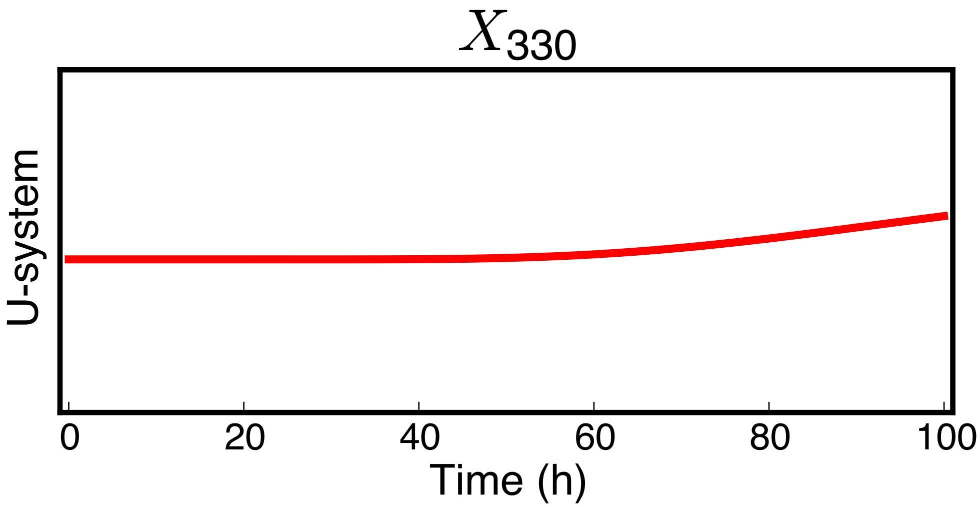

X_{330}
U-system
0
20
40
60
80
100
Time (h)

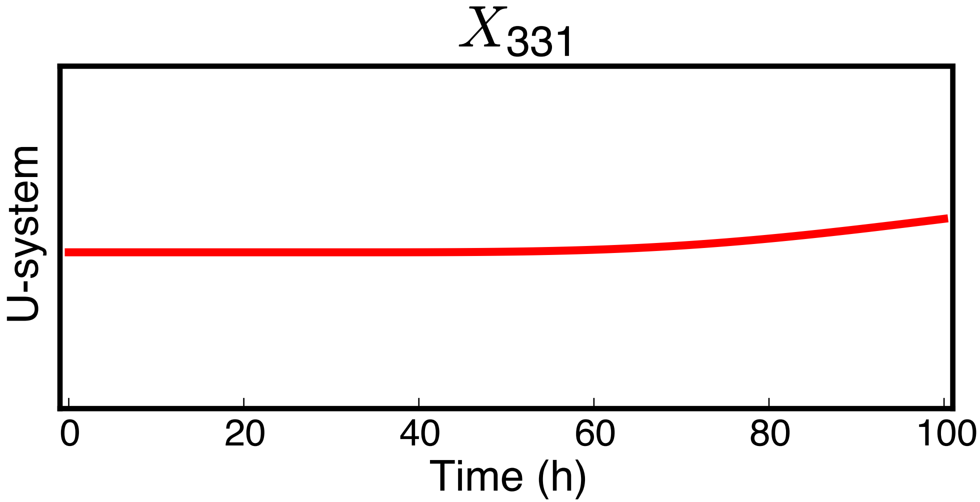

X_{331}
U-system
0
20
40
60
80
100
Time (h)

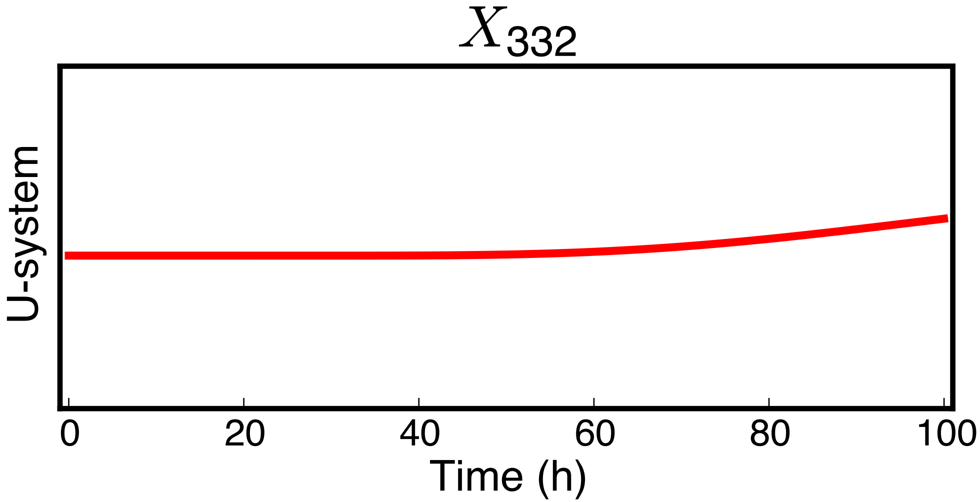

X332
U-system
0
20
40
60
80
100
Time (h)

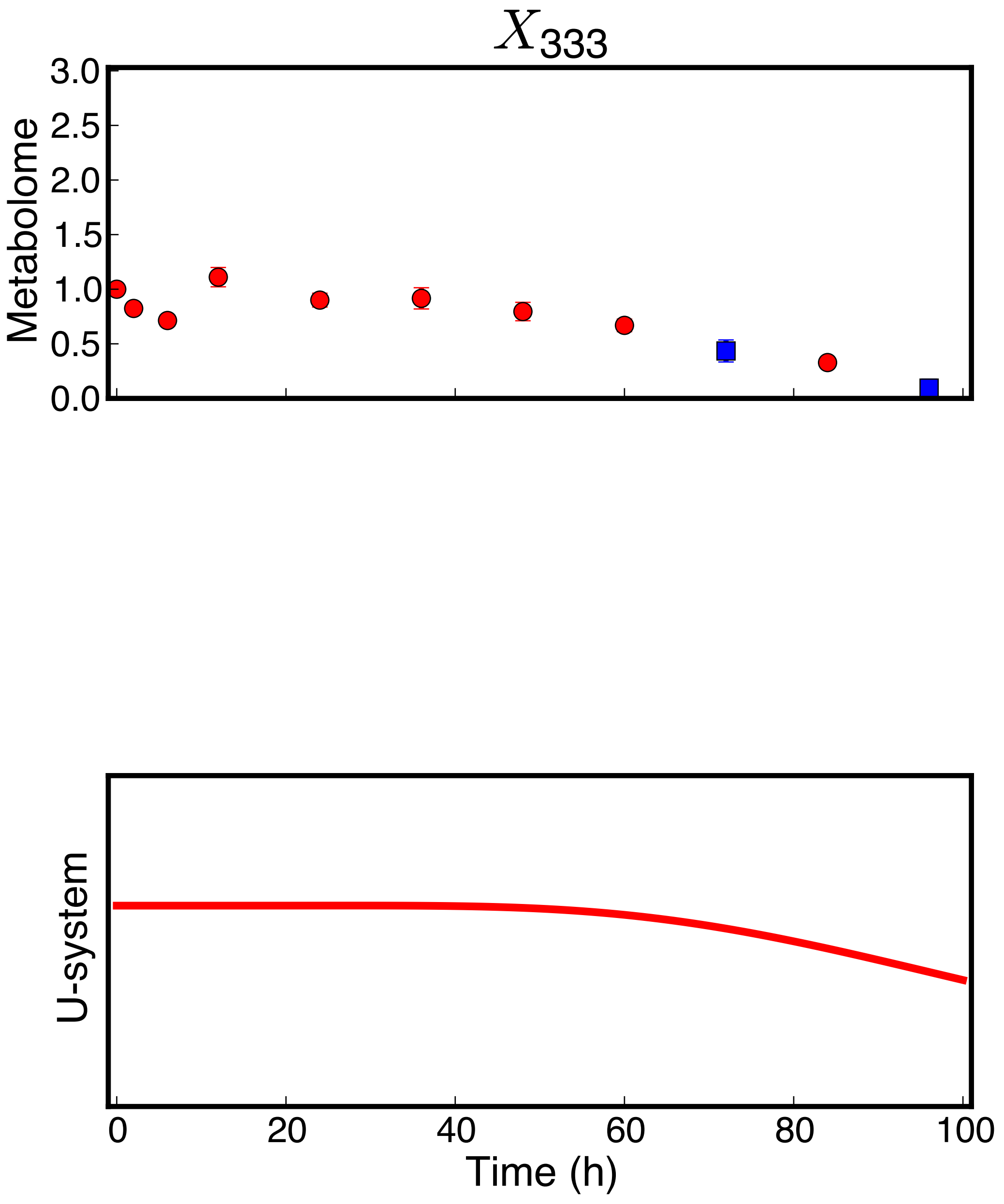

X_{333}
Metabolome
3.0
2.5
2.0
1.5
1.0
0.5
0.0
U-system
0
20
40
60
80
100
Time (h)

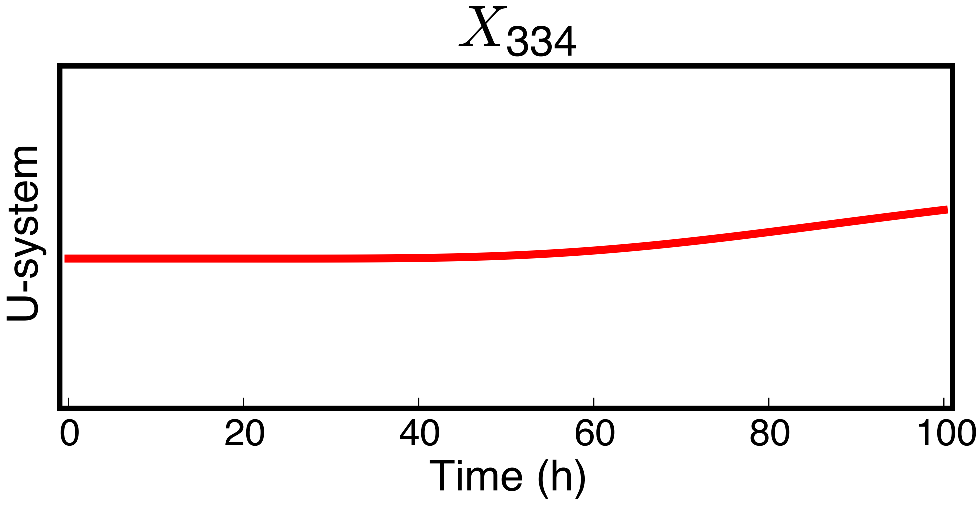

X_{334}
U-system
0
20
40
60
80
100
Time (h)

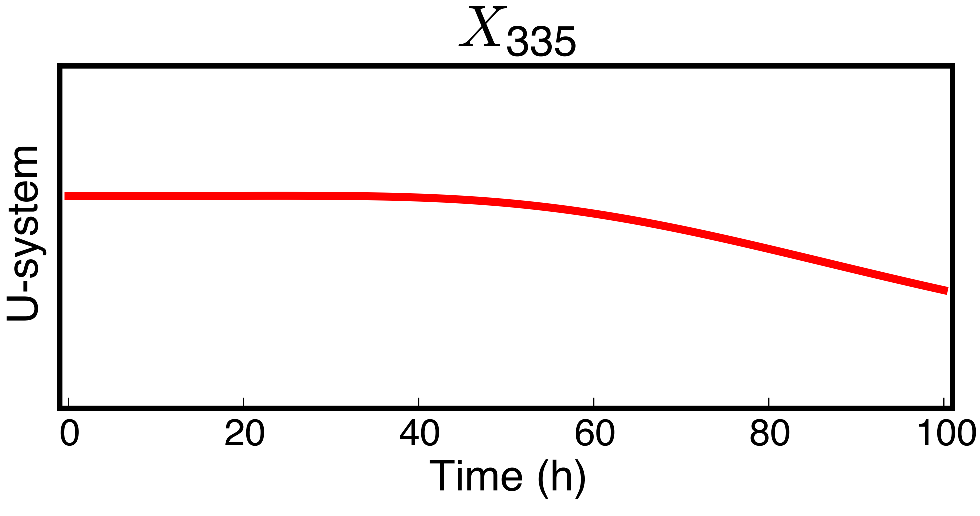

X_{335}
U-system
0
20
40
60
80
100
Time (h)

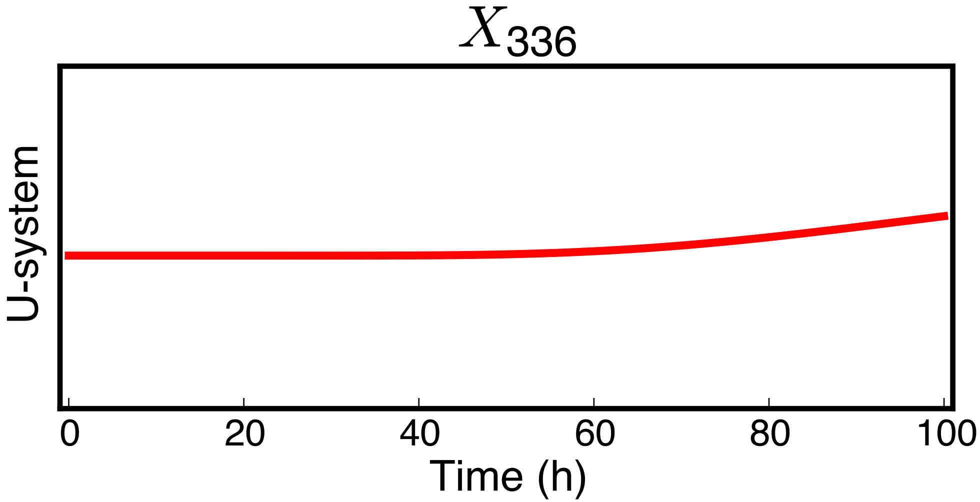

X_{336}
U-system
0
20
40
60
80
100
Time (h)

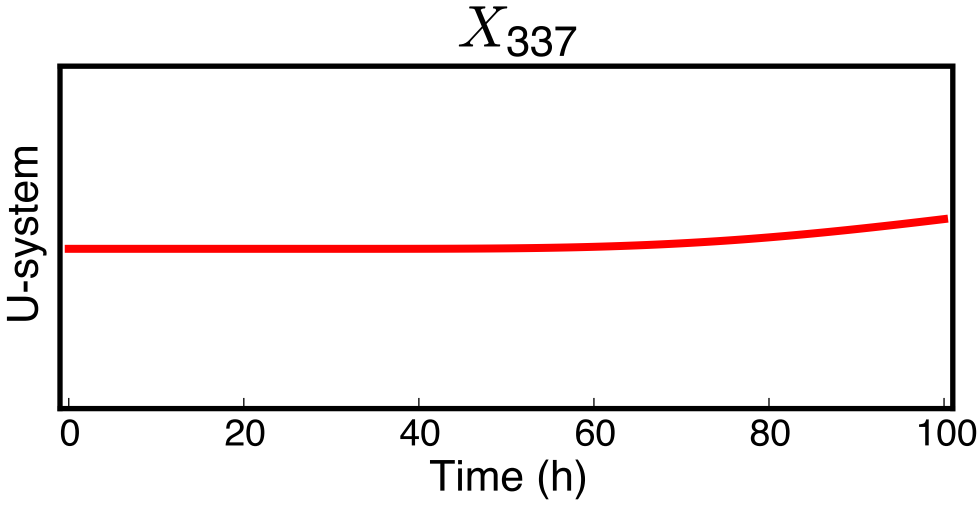

X_{337}
U-system
0
20
40
60
80
100
Time (h)

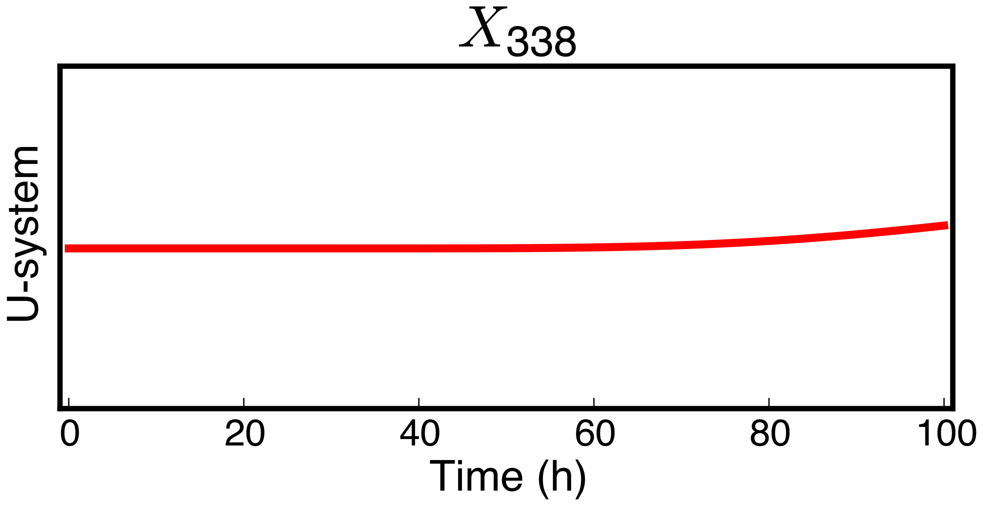

X_{338}
U-system
0
20
40
60
80
100
Time (h)

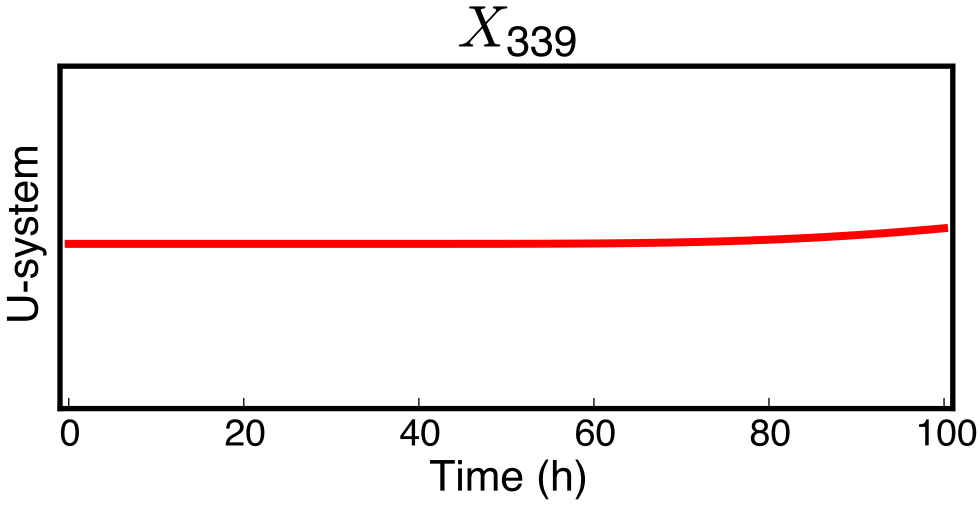

X_{339}
U-system
0
20
40
60
80
100
Time (h)

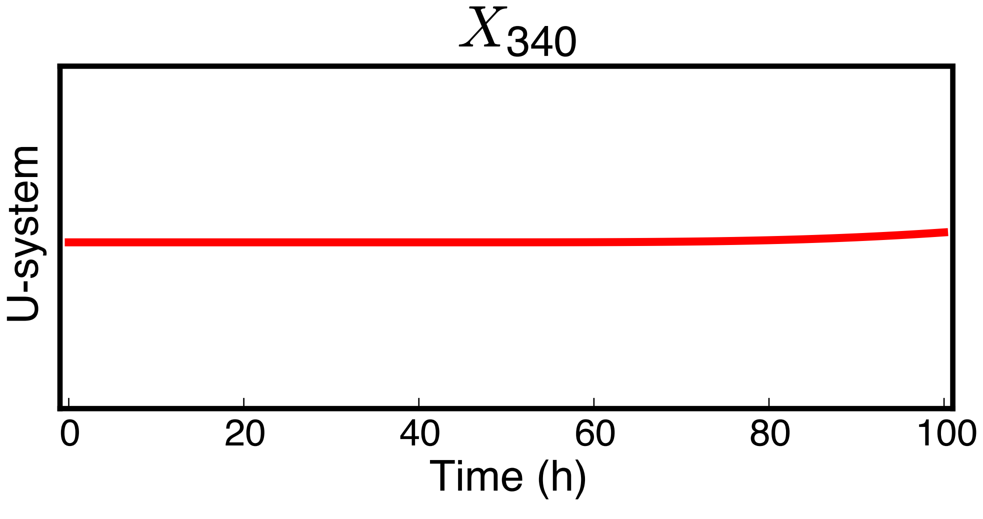

X_{340}
U-system
0
20
40
60
80
100
Time (h)

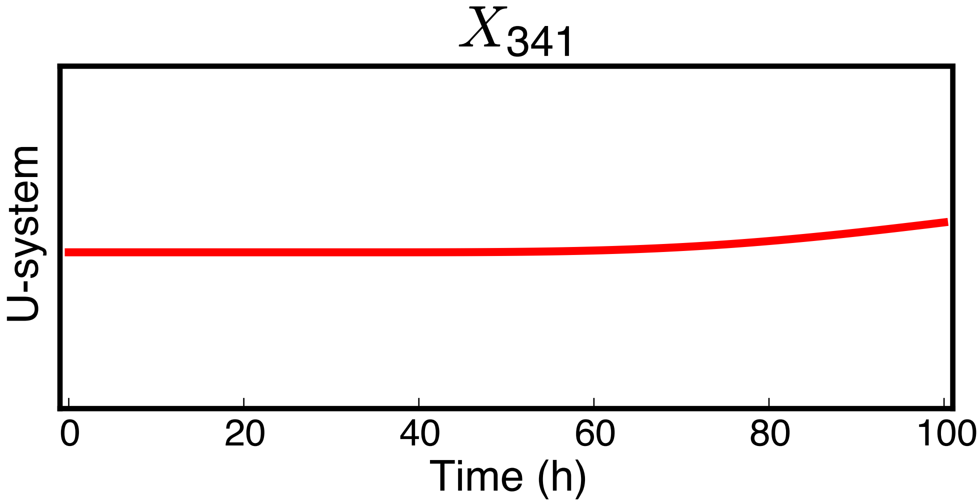

X_{341}
U-system
0
20
40
60
80
100
Time (h)

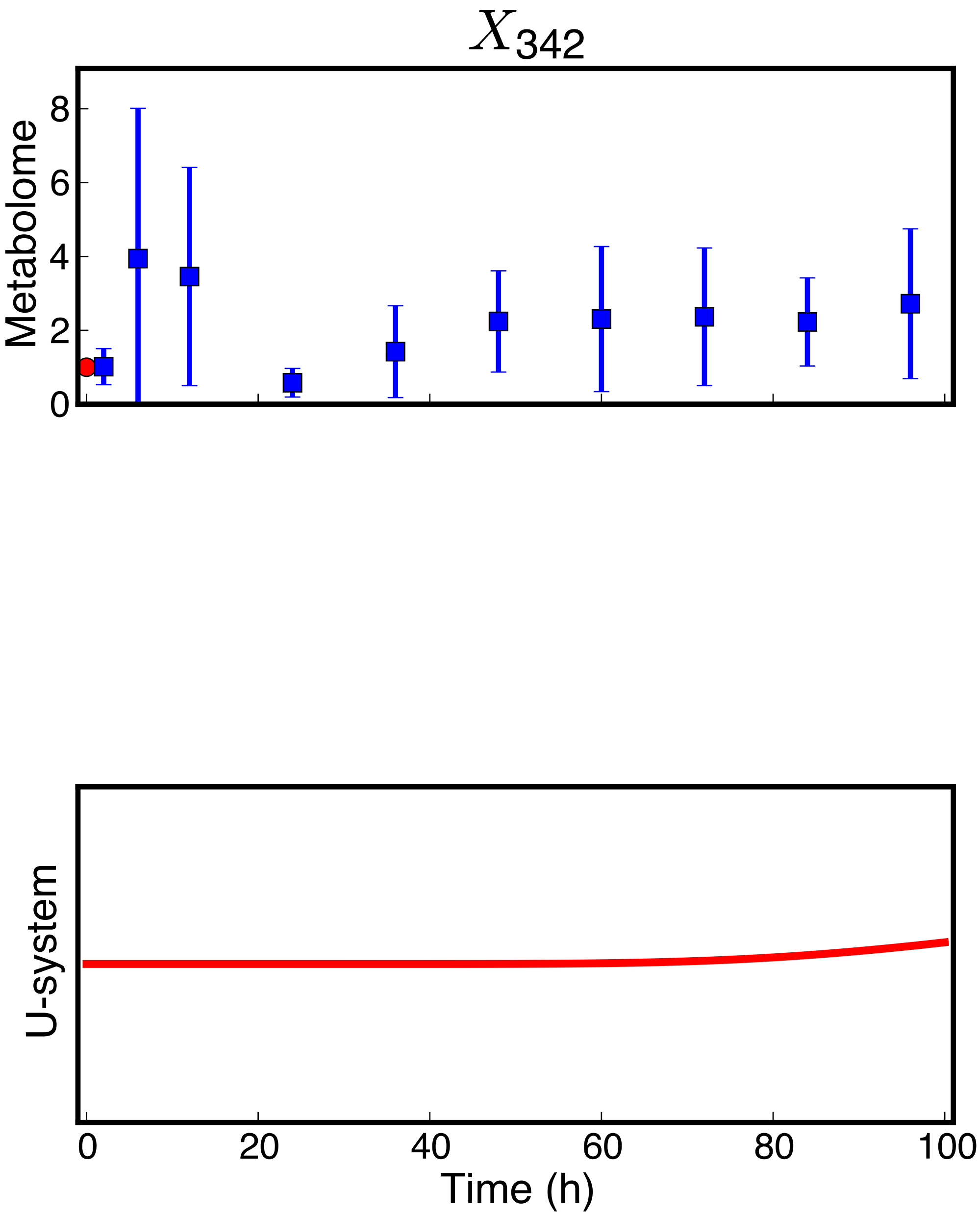

X_{342}
Metabolome
0
2
4
6
8
U-system
0
20
40
60
80
100
Time (h)

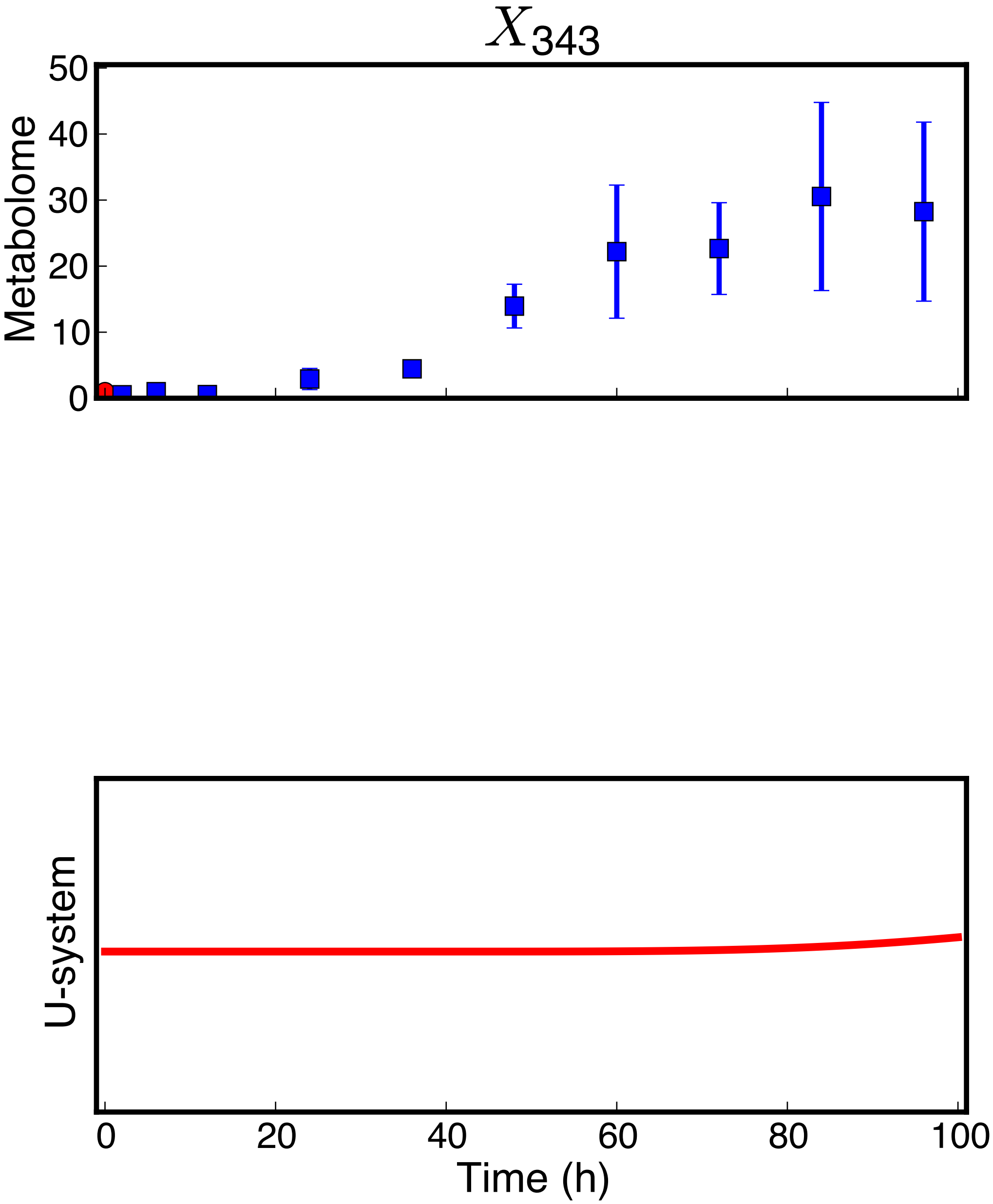

X_{343}
Metabolome
50
40
30
20
10
0
U-system
0
20
40
60
80
100
Time (h)

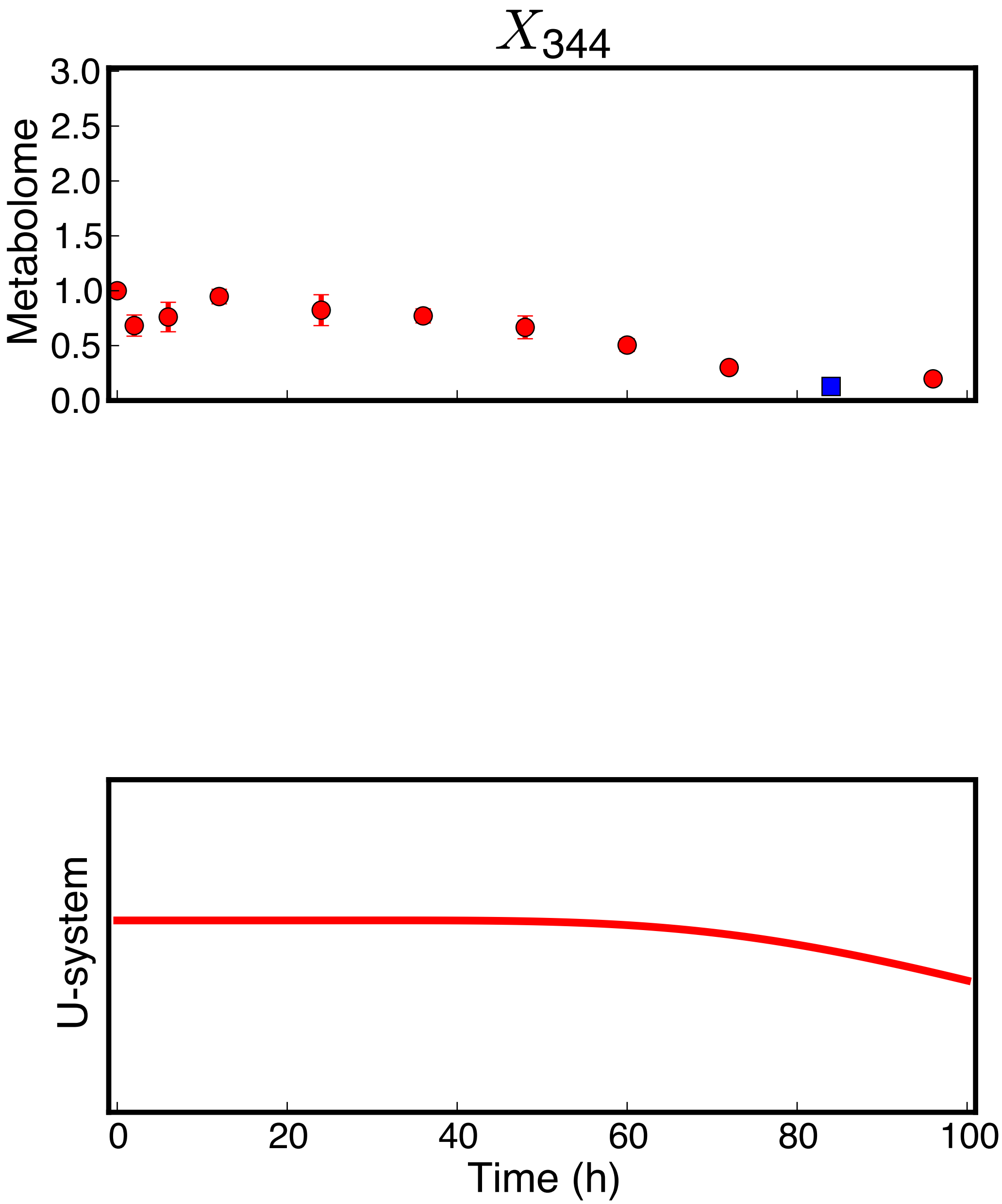

X_{344}
3.0
2.5
2.0
1.5
1.0
0.5
0.0
Metabolome
U-system
0
20
40
60
80
100
Time (h)

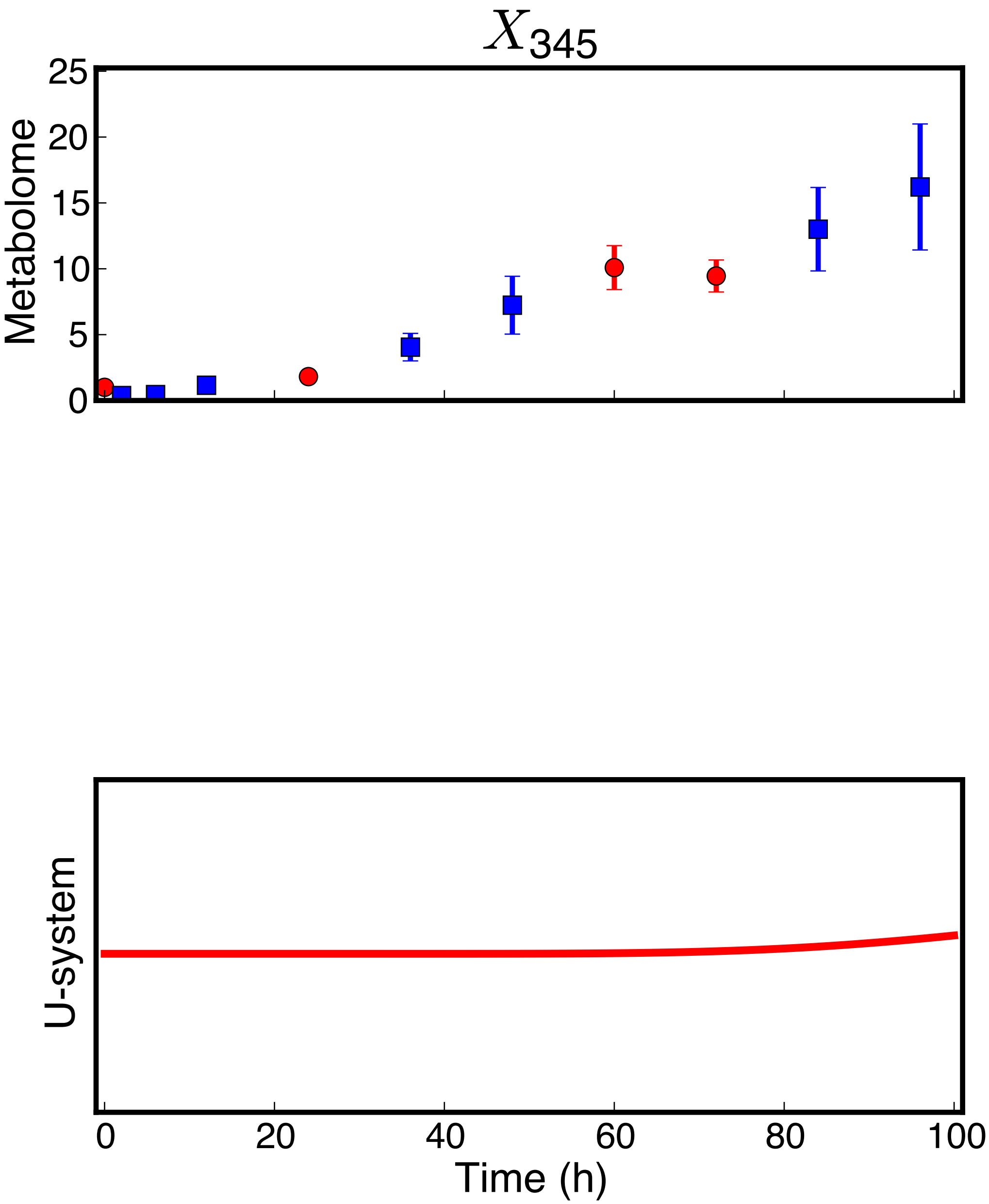
X_{345}
Metabolome
0
5
10
15
20
25
U-system
0
20
40
60
80
100
Time (h)

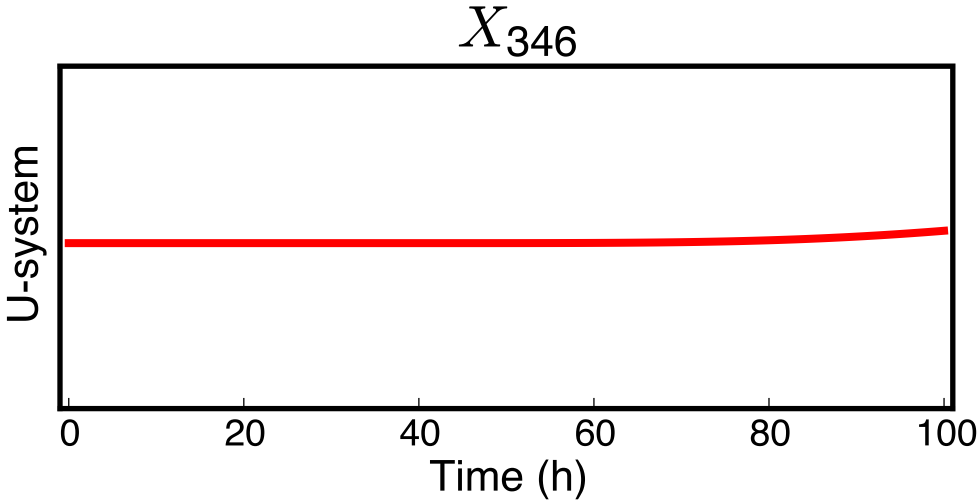
X_{346}
U-system
0
20
40
60
80
100
Time (h)

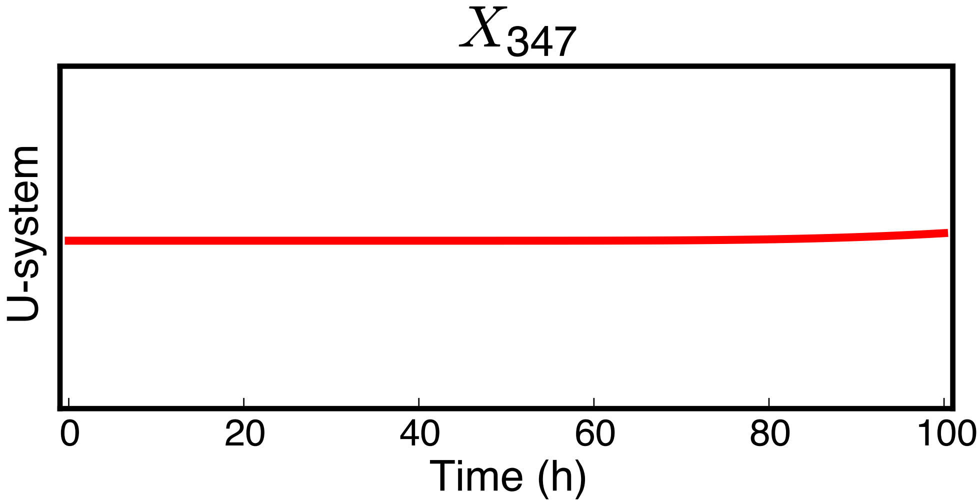

X_{347}
U-system
0
20
40
60
80
100
Time (h)

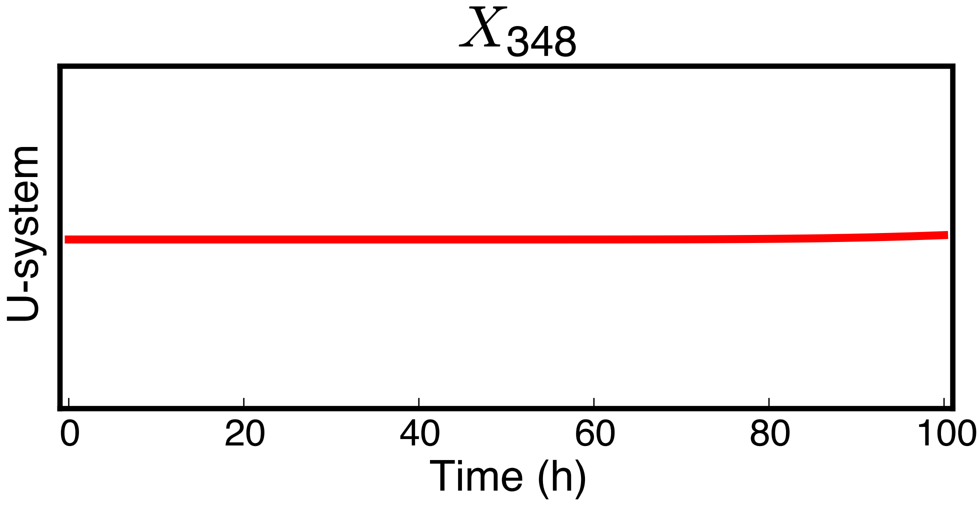

X_{348}
U-system
0
20
40
60
80
100
Time (h)

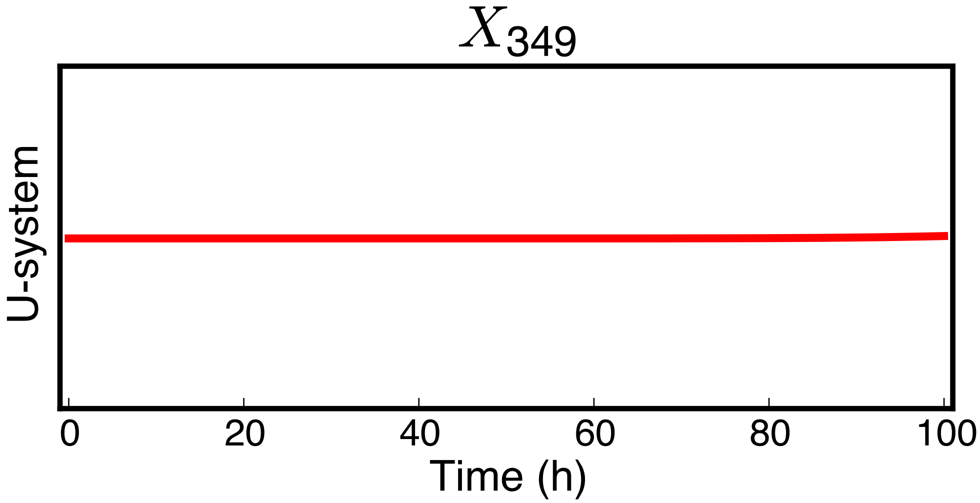
X_{349}
U-system
0
20
40
60
80
100
Time (h)

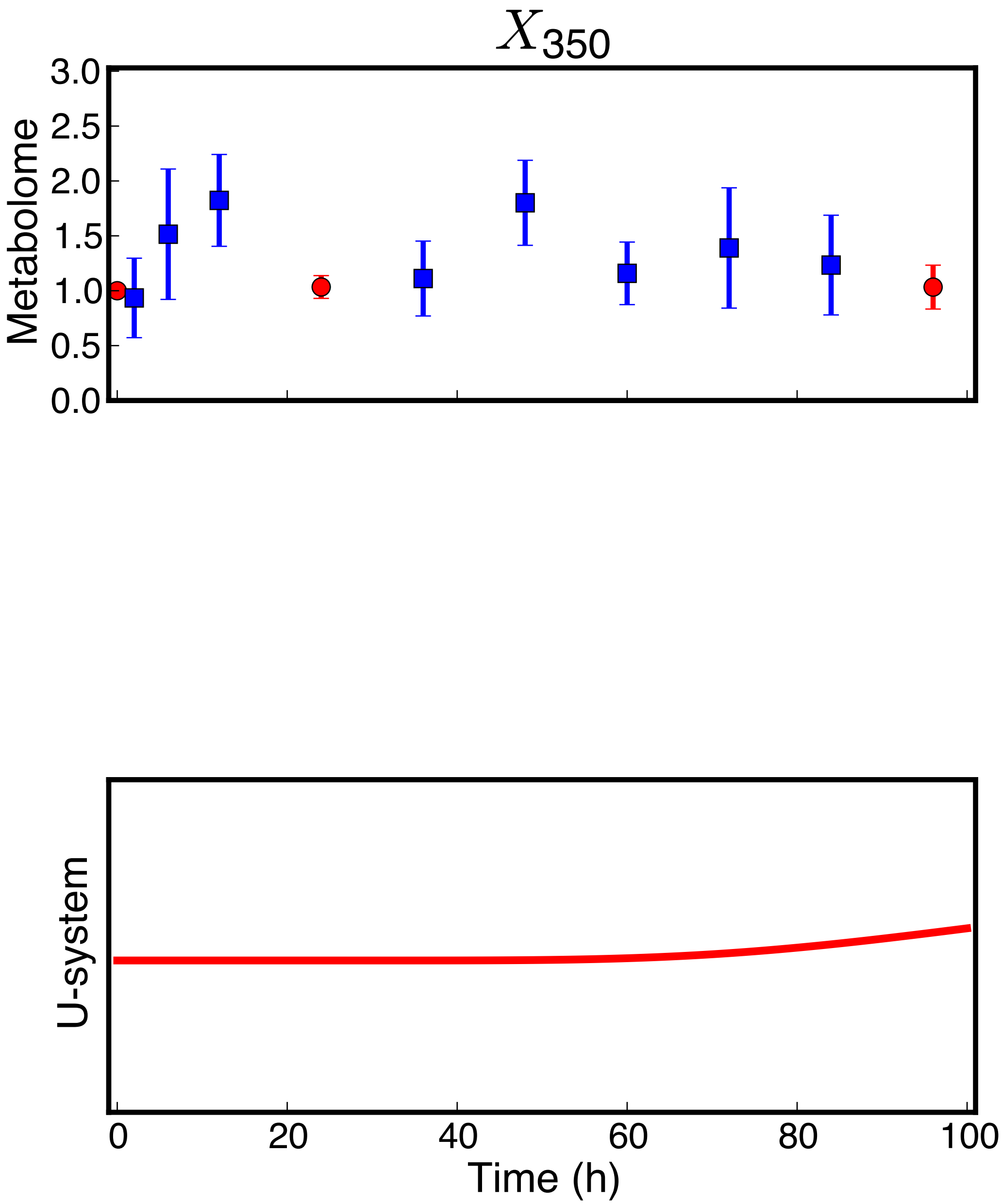

X_{350}
Metabolome
3.0
2.5
2.0
1.5
1.0
0.5
0.0
U-system
0
20
40
60
80
100
Time (h)

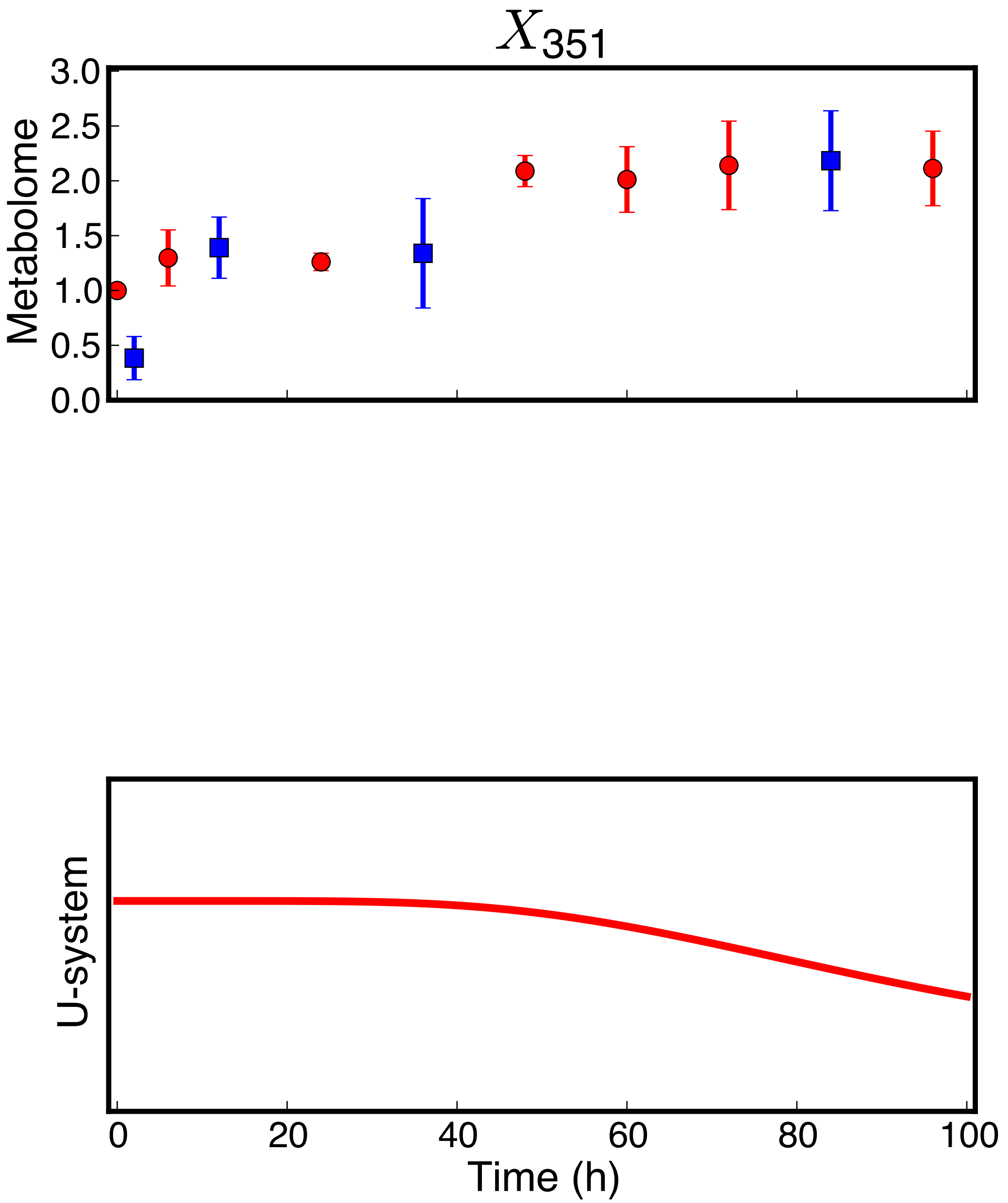

X_{351}
Metabolome
3.0
2.5
2.0
1.5
1.0
0.5
0.0
U-system
0
20
40
60
80
100
Time (h)

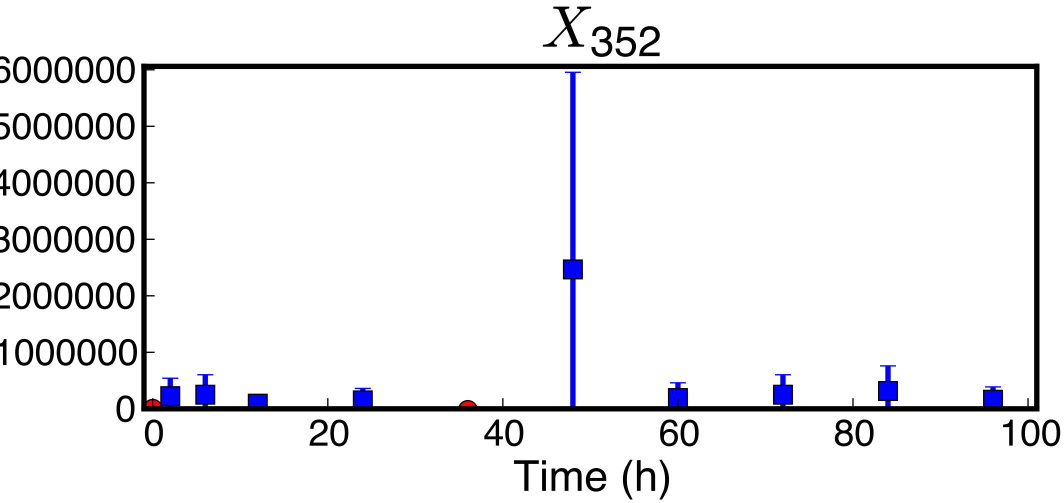

X_{352}
6000000
5000000
4000000
3000000
2000000
1000000
0
0
20
40
60
80
100
Time (h)

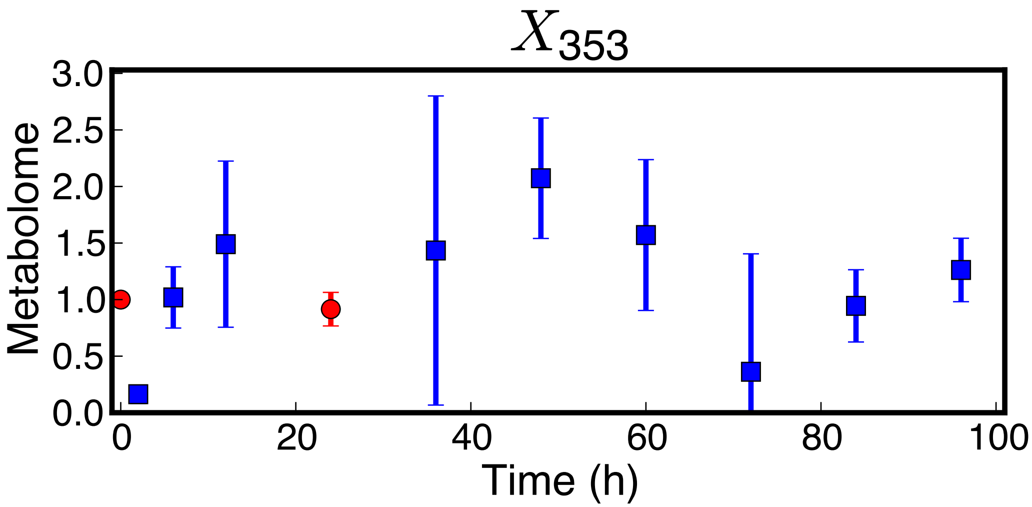

X_{353}
Metabolome
Time (h)
0.0
0.5
1.0
1.5
2.0
2.5
3.0
0
20
40
60
80
100

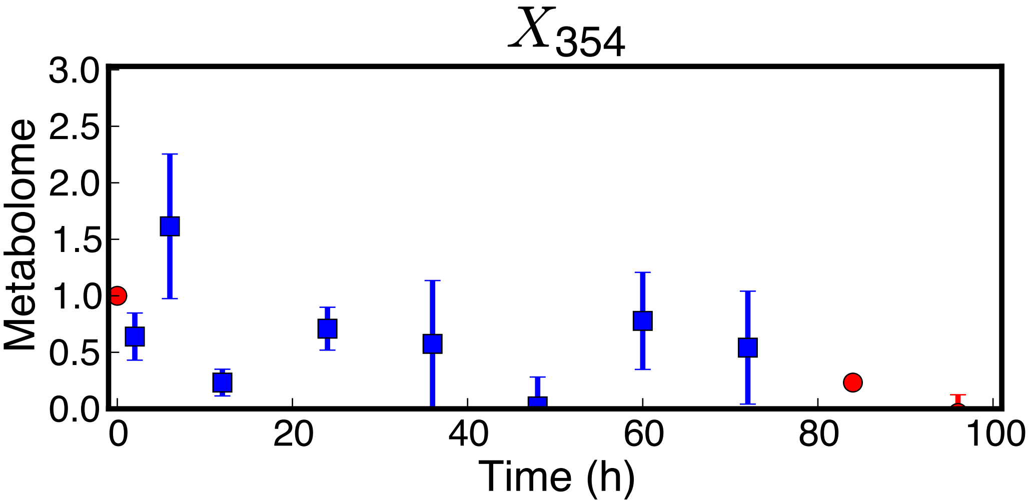

X_{354}
Metabolome
3.0
2.5
2.0
1.5
1.0
0.5
0.0
0
20
40
60
80
100
Time (h)

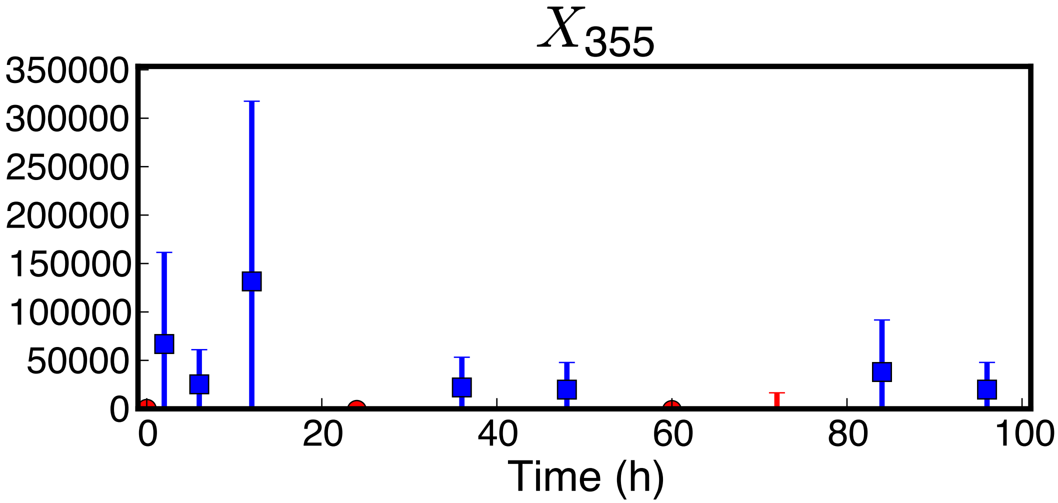
X_{355}
350000
300000
250000
200000
150000
100000
50000
0
0
20
40
60
80
100
Time (h)

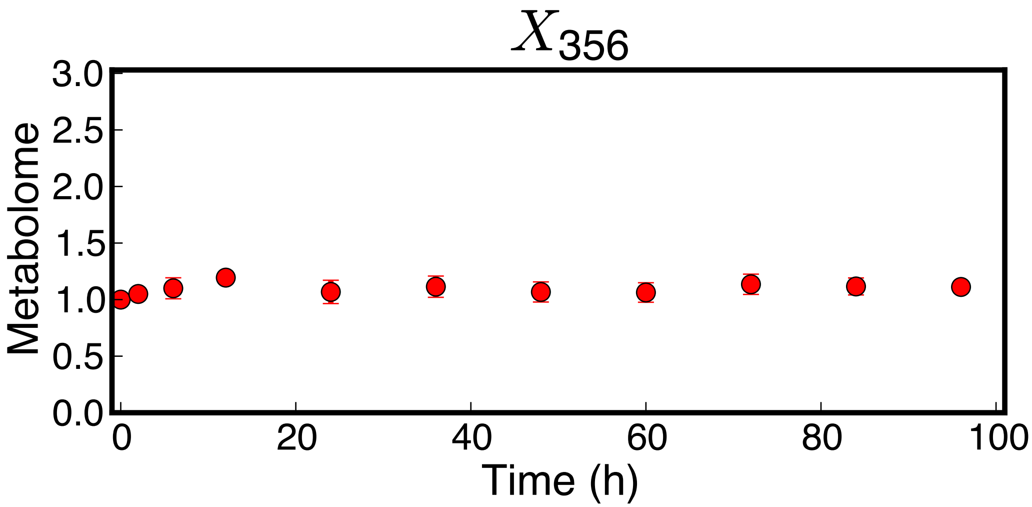

X_{356}
Metabolome
3.0
2.5
2.0
1.5
1.0
0.5
0.0
0
20
40
60
80
100
Time (h)

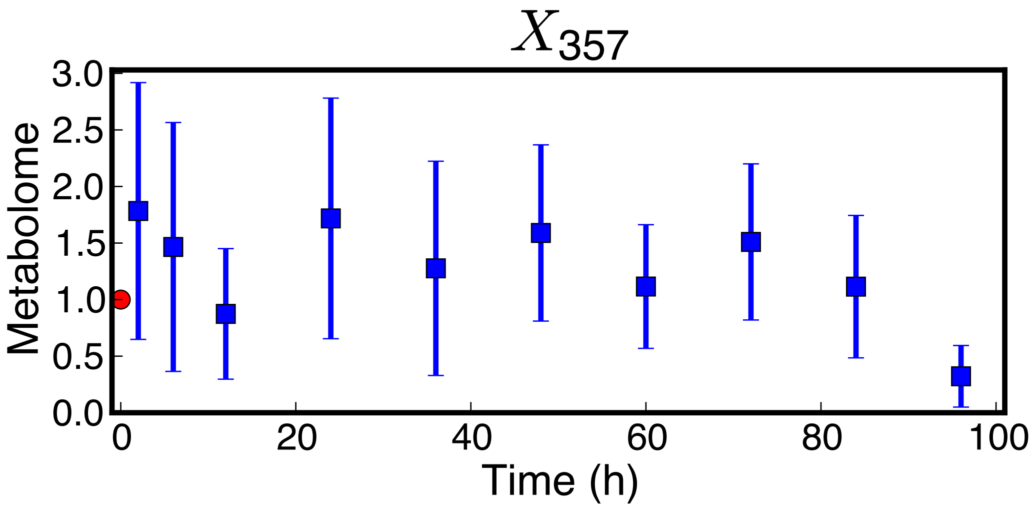
X_{357}
3.0
2.5
2.0
1.5
1.0
0.5
0.0
Metabolome
0
20
40
60
80
100
Time (h)

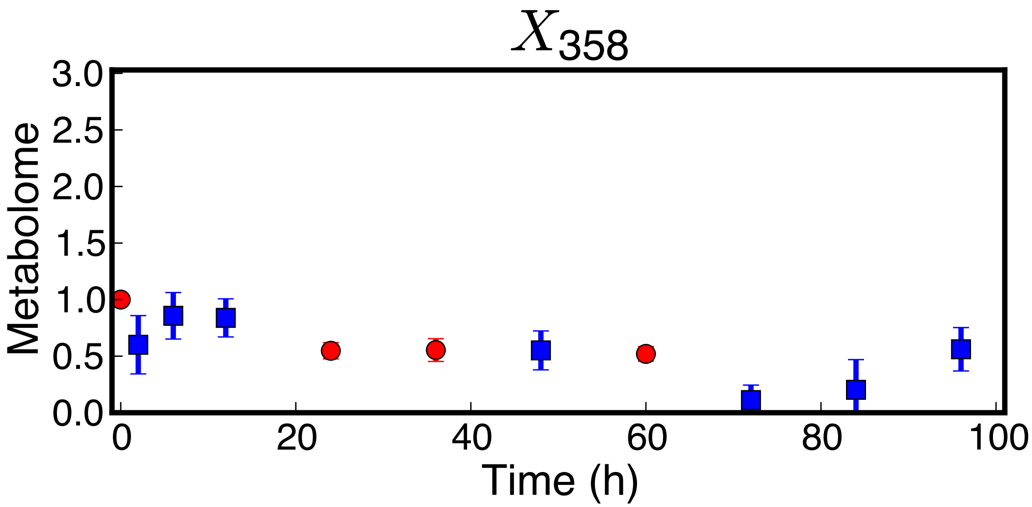

X_{358}
Metabolome
3.0
2.5
2.0
1.5
1.0
0.5
0.0
0
20
40
60
80
100
Time (h)

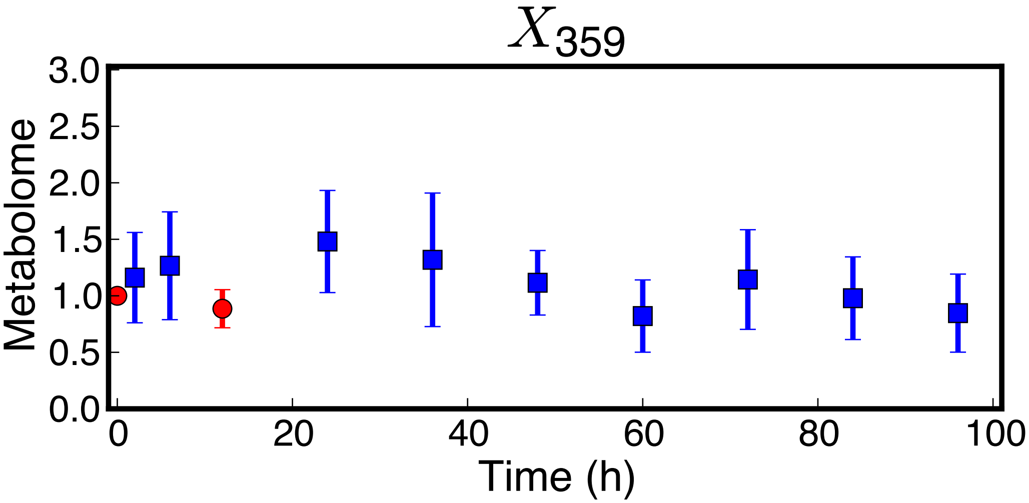
X_{359}
Metabolome
3.0
2.5
2.0
1.5
1.0
0.5
0.0
0
20
40
60
80
100
Time (h)

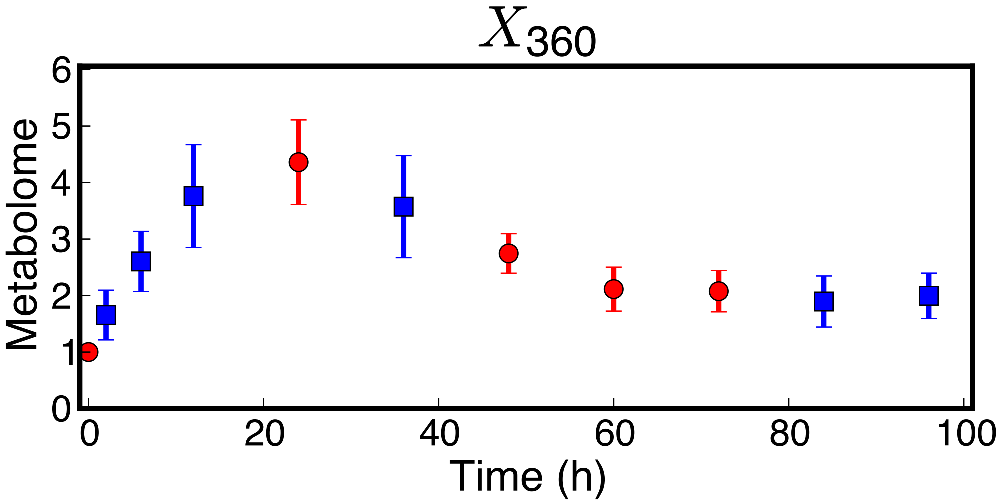

X_{360}
Metabolome
Time (h)
0
1
2
3
4
5
6
0
20
40
60
80
100

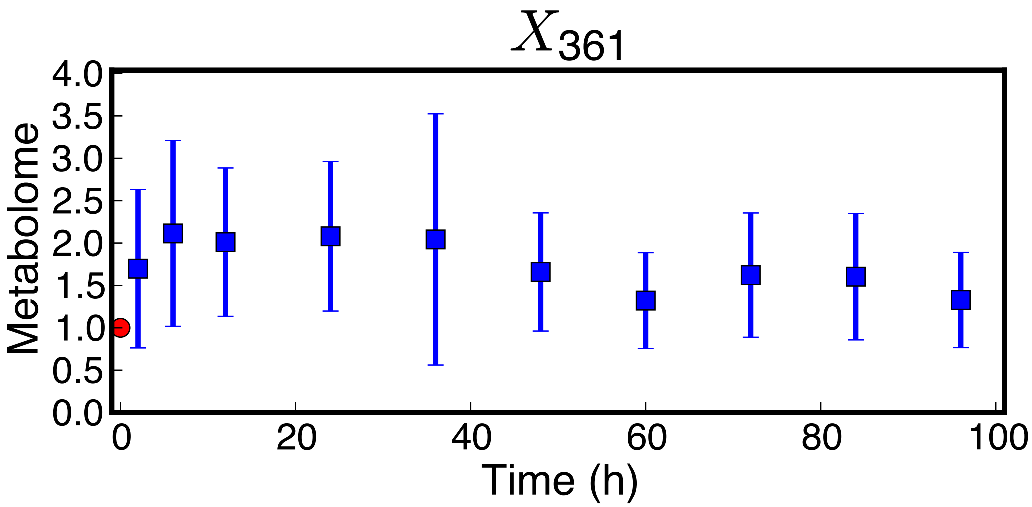

X_{361}
Metabolome
0.0
0.5
1.0
1.5
2.0
2.5
3.0
3.5
4.0
0
20
40
60
80
100
Time (h)

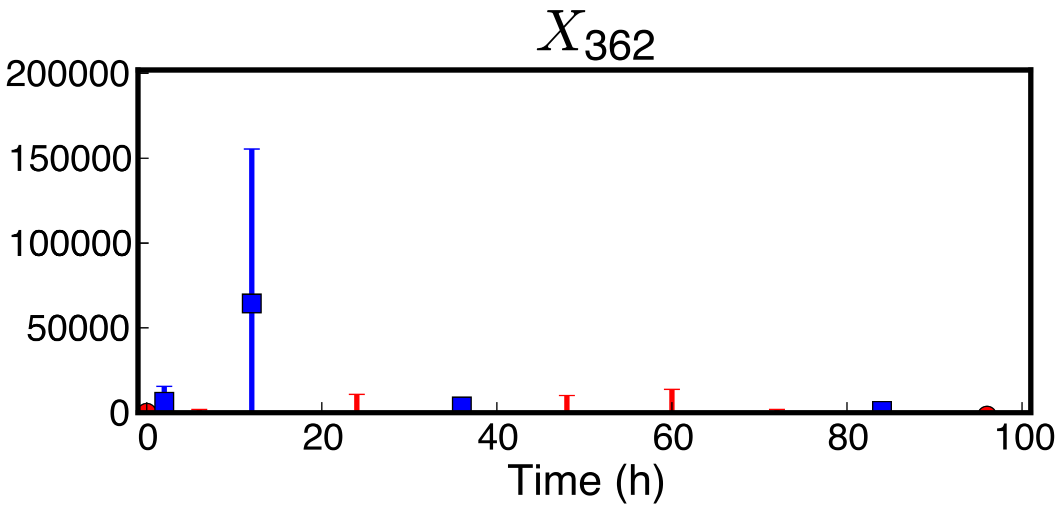
X_{362}
200000
150000
100000
50000
0
0
20
40
60
80
100
Time (h)

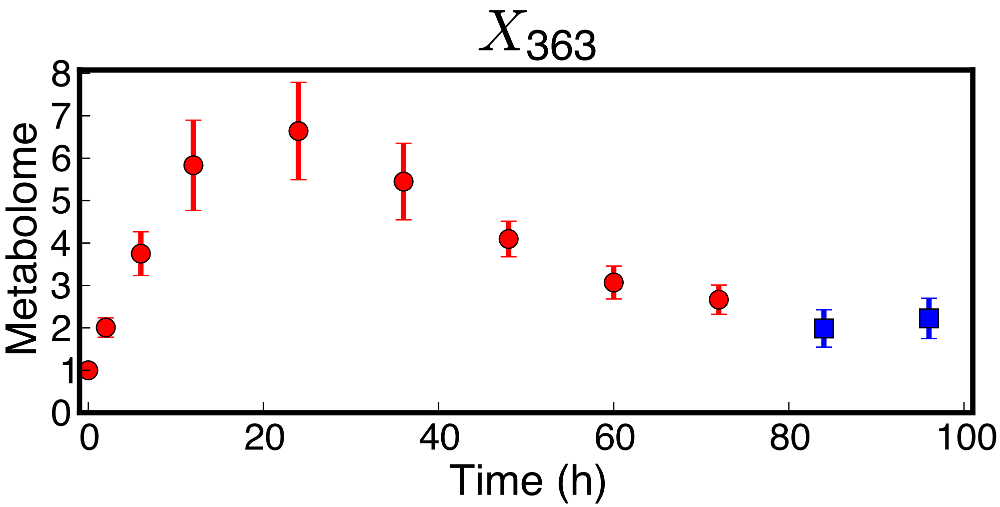
X_{363}
Metabolome
Time (h)
0
1
2
3
4
5
6
7
8
0
20
40
60
80
100

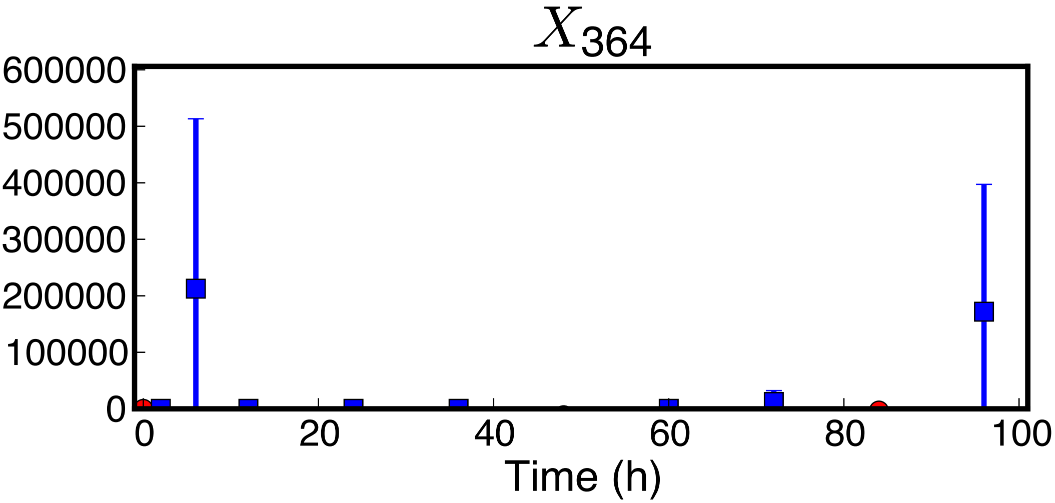

X_{364}
600000
500000
400000
300000
200000
100000
0
0
20
40
60
80
100
Time (h)

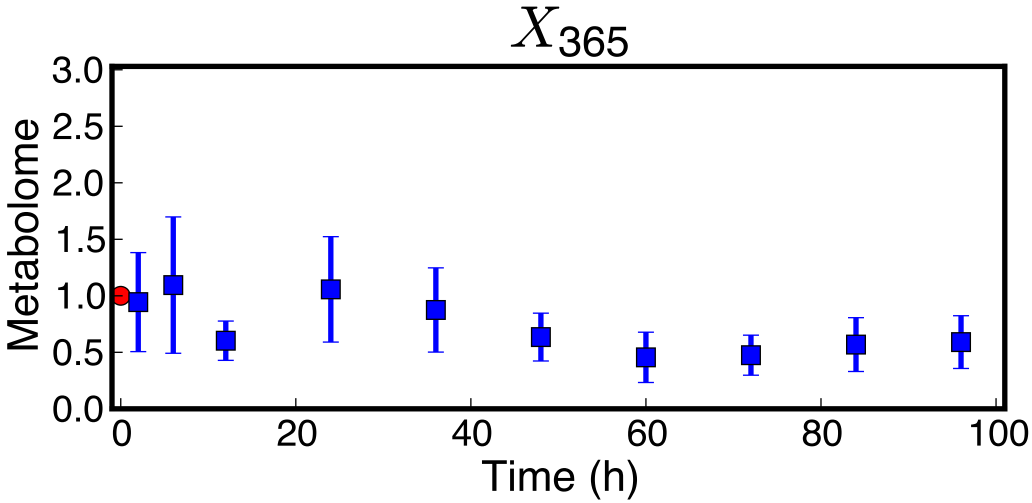

X_{365}
Metabolome
3.0
2.5
2.0
1.5
1.0
0.5
0.0
0
20
40
60
80
100
Time (h)

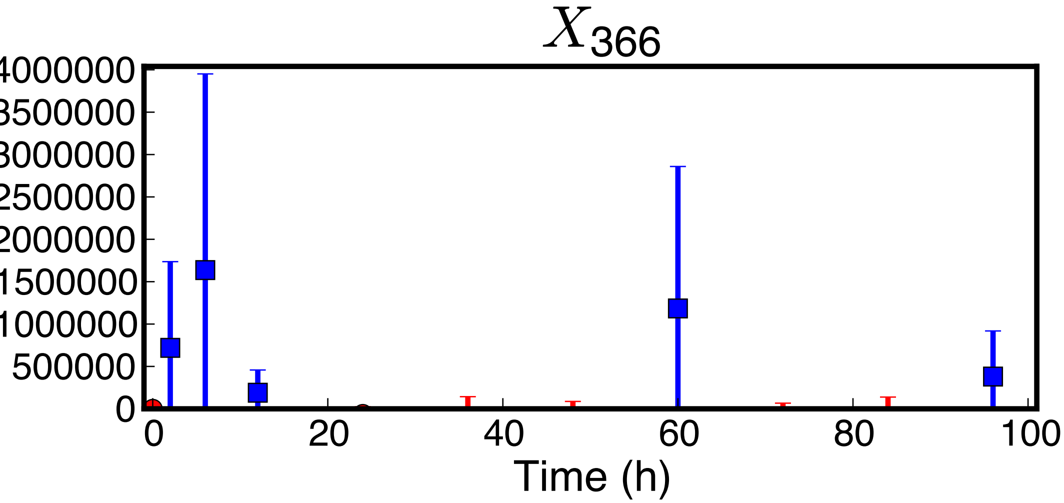

X_{366}
4000000
3500000
3000000
2500000
2000000
1500000
1000000
500000
0
0
20
40
60
80
100
Time (h)

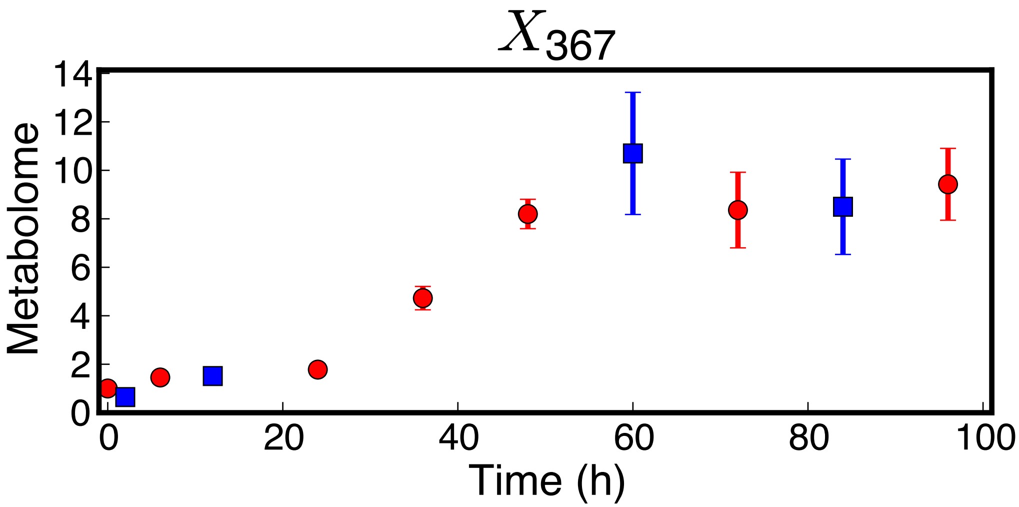

X_{367}
Metabolome
Time (h)
0
2
4
6
8
10
12
14
0
20
40
60
80
100

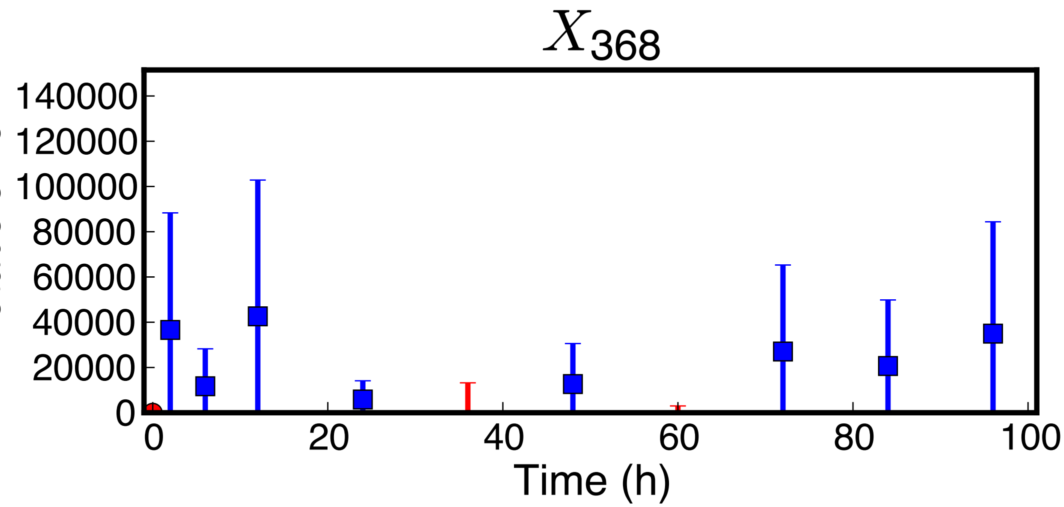

X_{368}
140000
120000
100000
80000
60000
40000
20000
0
0
20
40
60
80
100
Time (h)

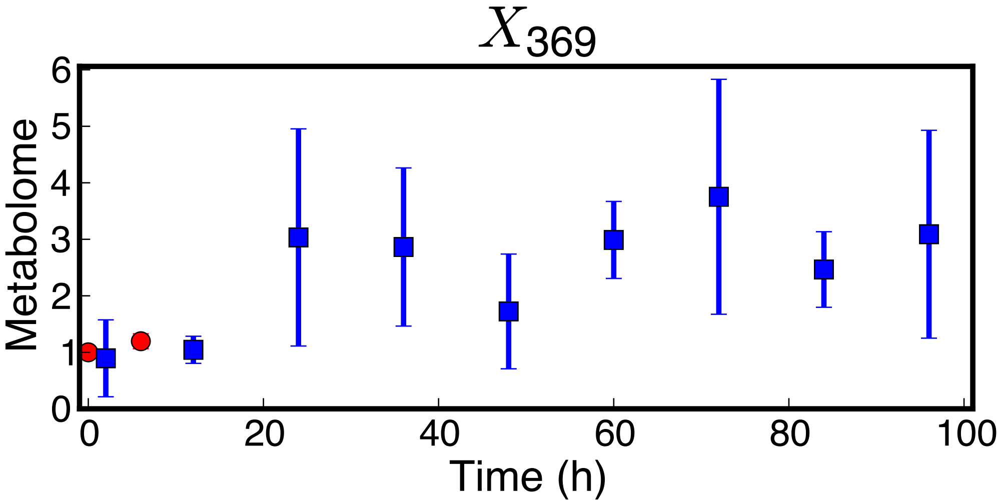
X_{369}
Metabolome
Time (h)
0
1
2
3
4
5
6
0
20
40
60
80
100

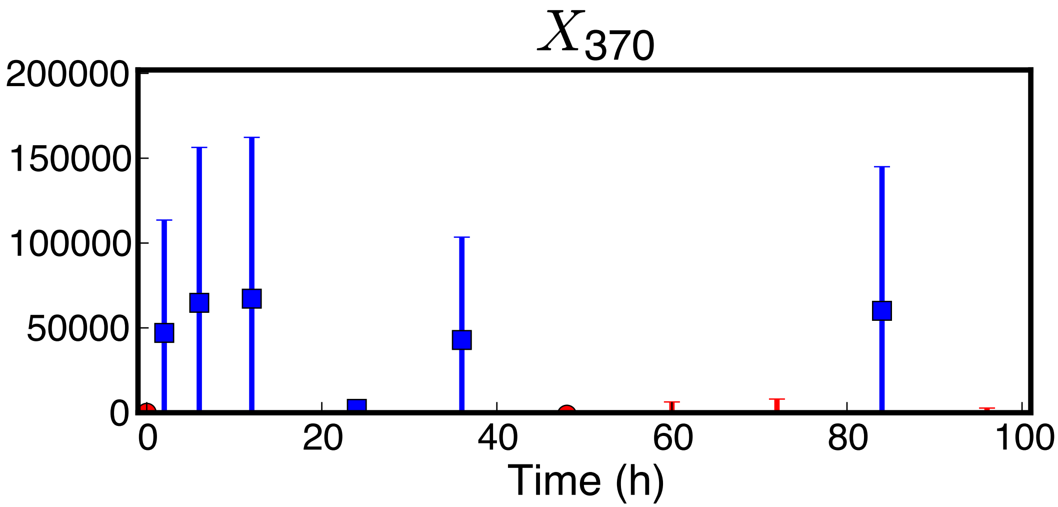

X_{370}
200000
150000
100000
50000
0
0
20
40
60
80
100
Time (h)

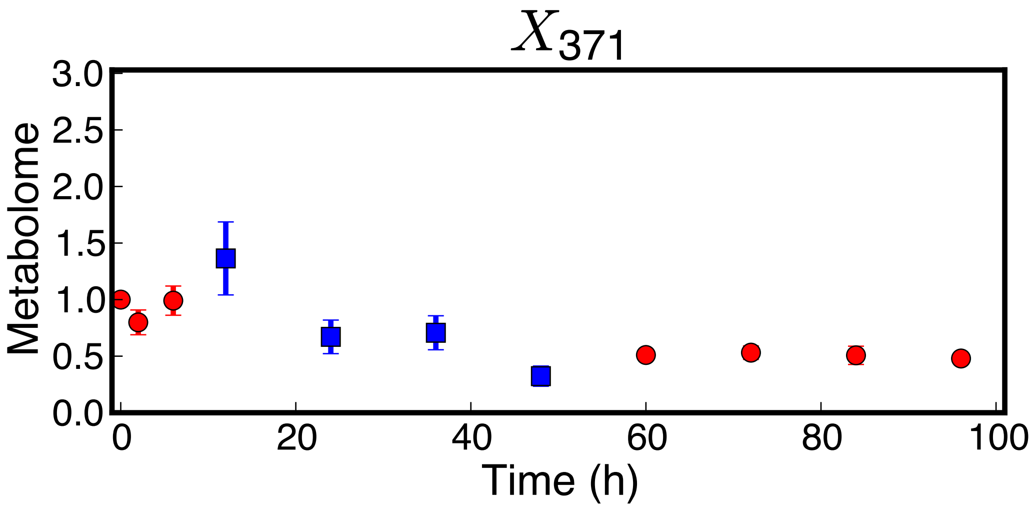

X_{371}
Metabolome
3.0
2.5
2.0
1.5
1.0
0.5
0.0
0
20
40
60
80
100
Time (h)

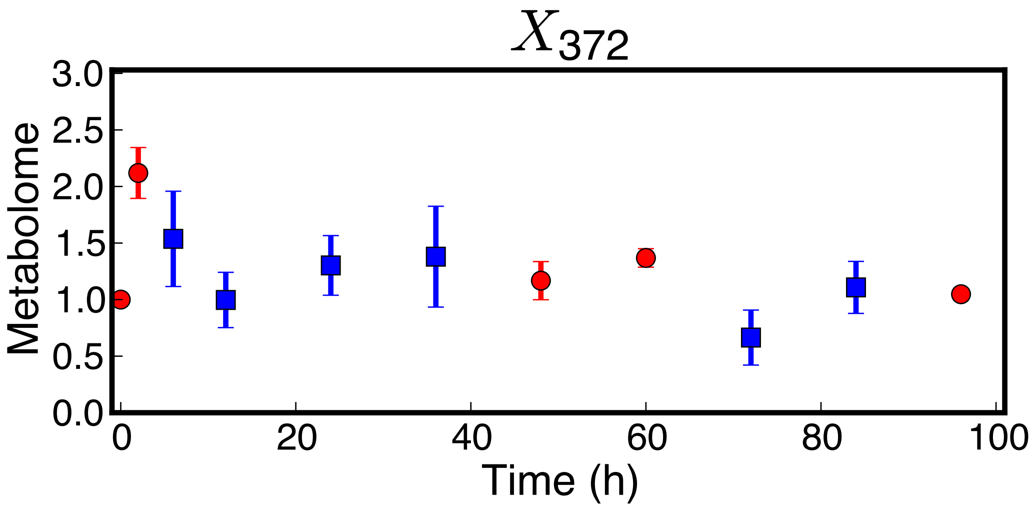

X_{372}
Metabolome
3.0
2.5
2.0
1.5
1.0
0.5
0.0
0
20
40
60
80
100
Time (h)

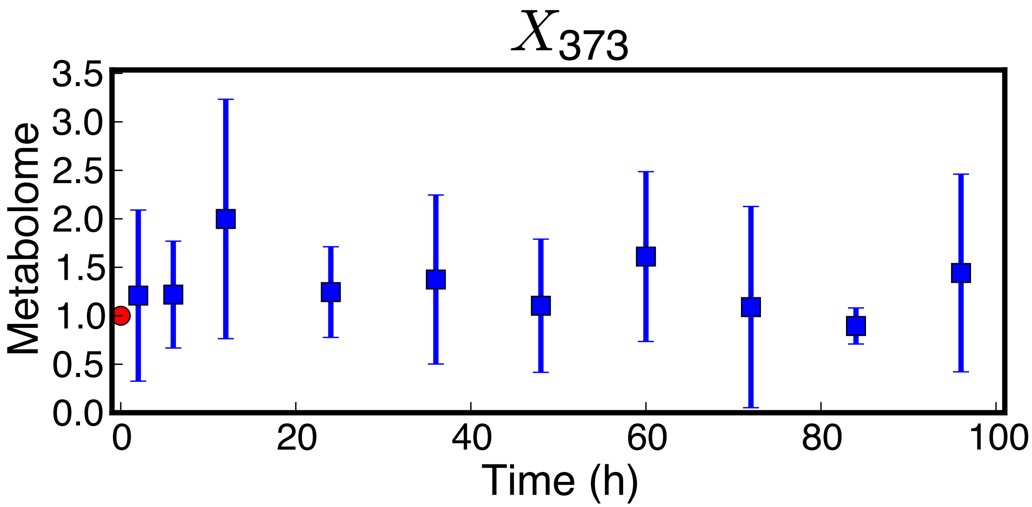

X_{373}
Metabolome
3.5
3.0
2.5
2.0
1.5
1.0
0.5
0.0
0
20
40
60
80
100
Time (h)

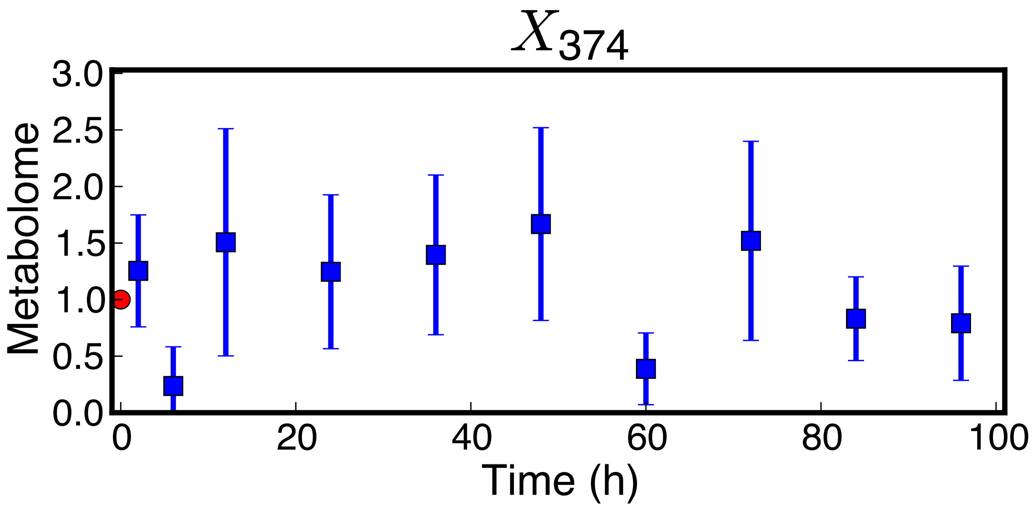

X_{374}
Metabolome
3.0
2.5
2.0
1.5
1.0
0.5
0.0
0
20
40
60
80
100
Time (h)

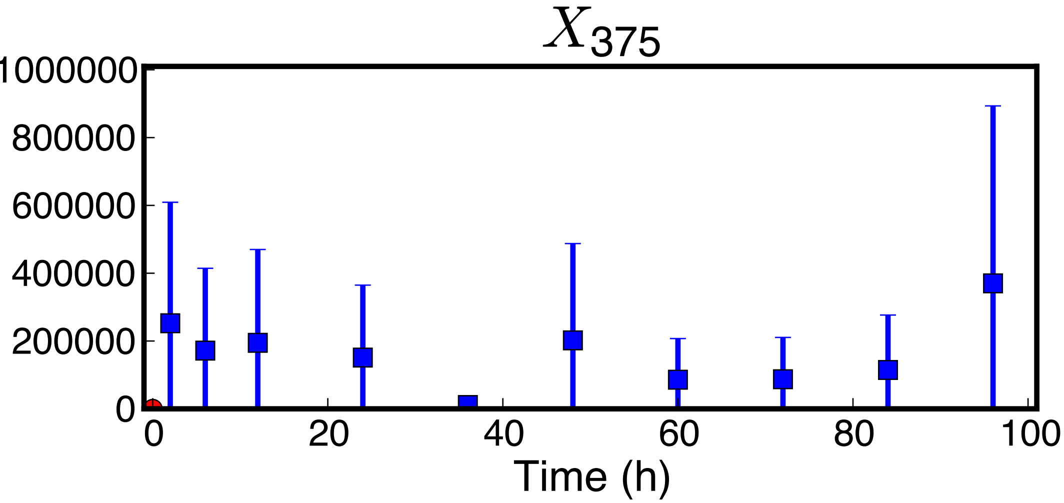

X_{375}
1000000
800000
600000
400000
200000
0
0
20
40
60
80
100
Time (h)

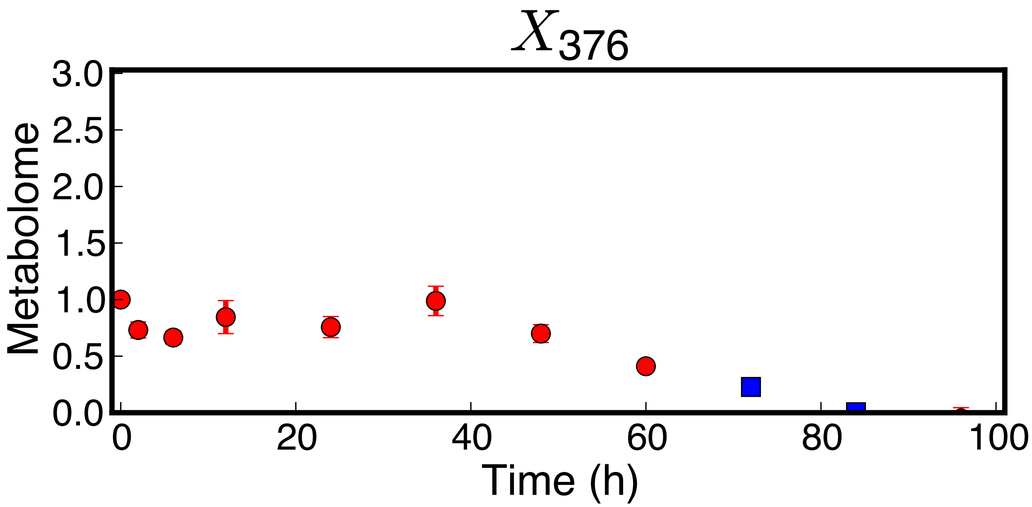

X_{376}
3.0
2.5
2.0
1.5
1.0
0.5
0.0
Metabolome
0
20
40
60
80
100
Time (h)

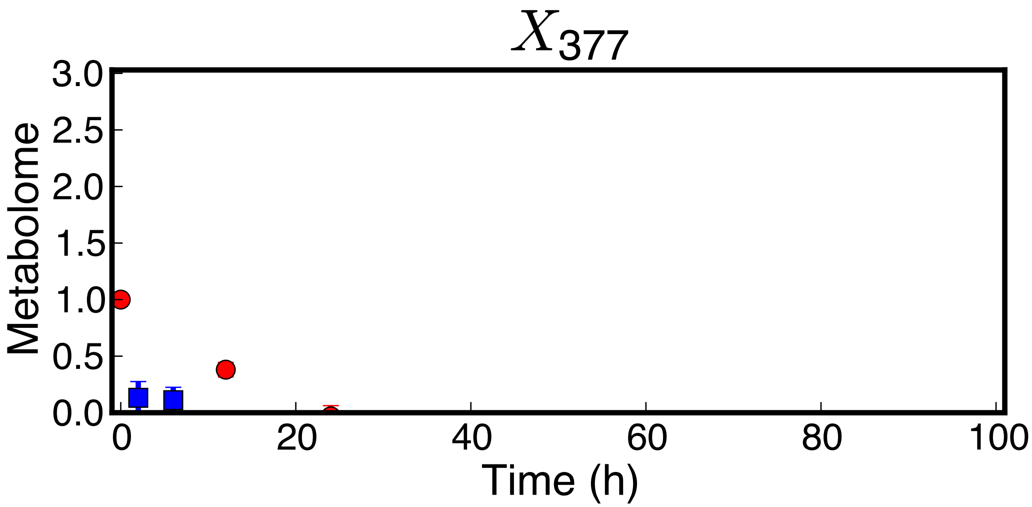

X_{377}
Metabolome
3.0
2.5
2.0
1.5
1.0
0.5
0.0
0
20
40
60
80
100
Time (h)

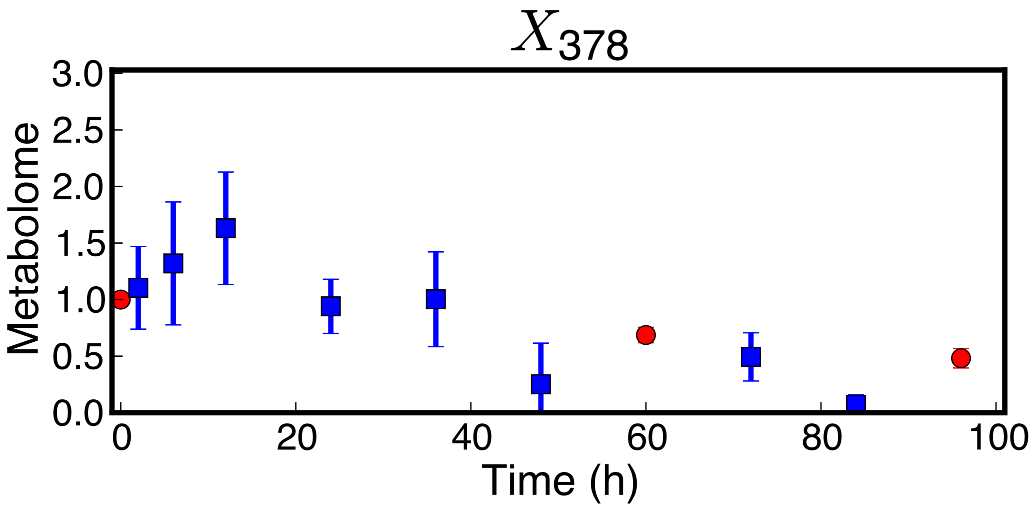

X_{378}
Metabolome
3.0
2.5
2.0
1.5
1.0
0.5
0.0
0
20
40
60
80
100
Time (h)

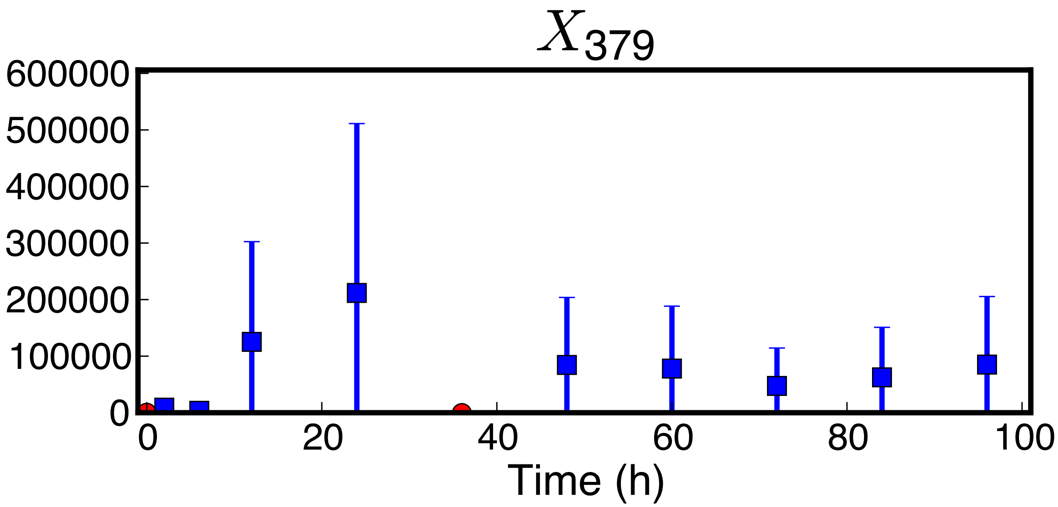

X_{379}
600000
500000
400000
300000
200000
100000
0
0
20
40
60
80
100
Time (h)

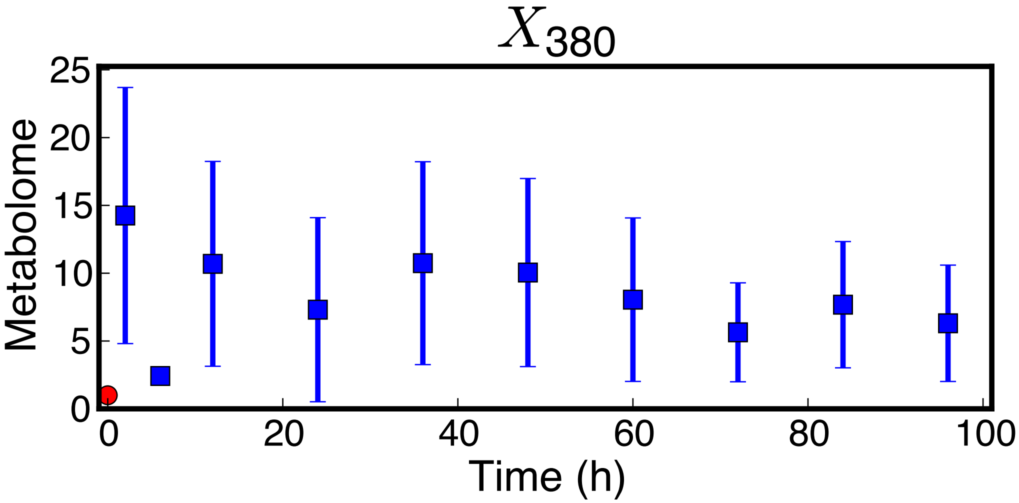

X_{380}
Metabolome
25
20
15
10
5
0
0
20
40
60
80
100
Time (h)

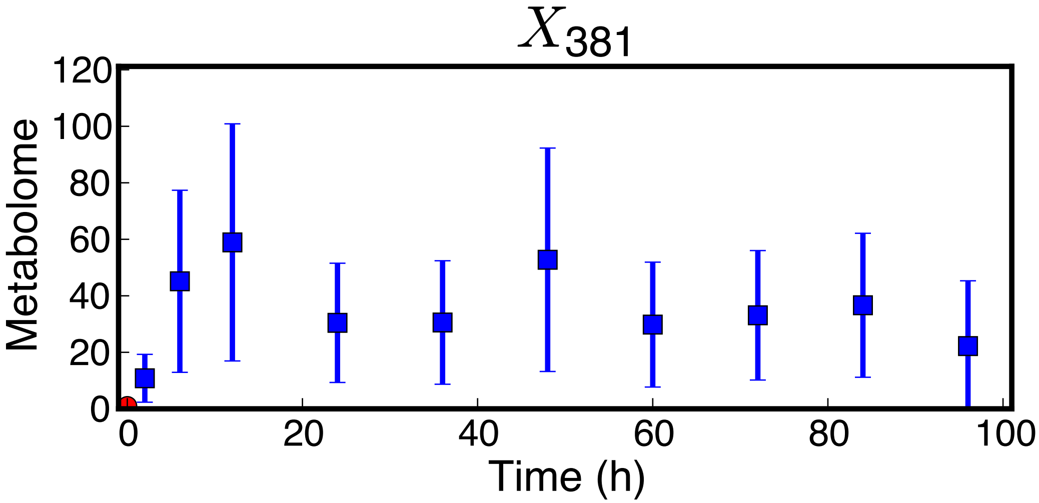

X_{381}
Metabolome
Time (h)
0
20
40
60
80
100
120

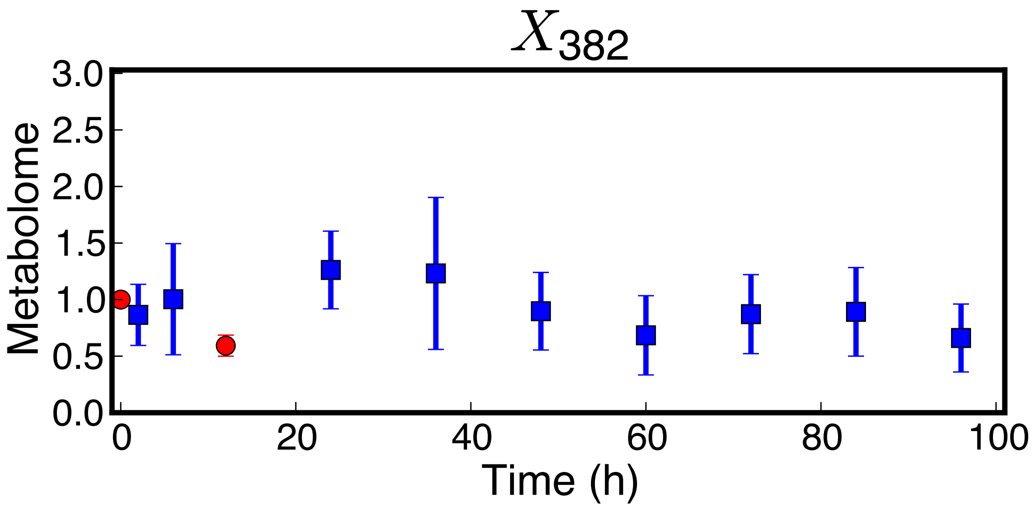

X_{382}
Metabolome
3.0
2.5
2.0
1.5
1.0
0.5
0.0
0
20
40
60
80
100
Time (h)

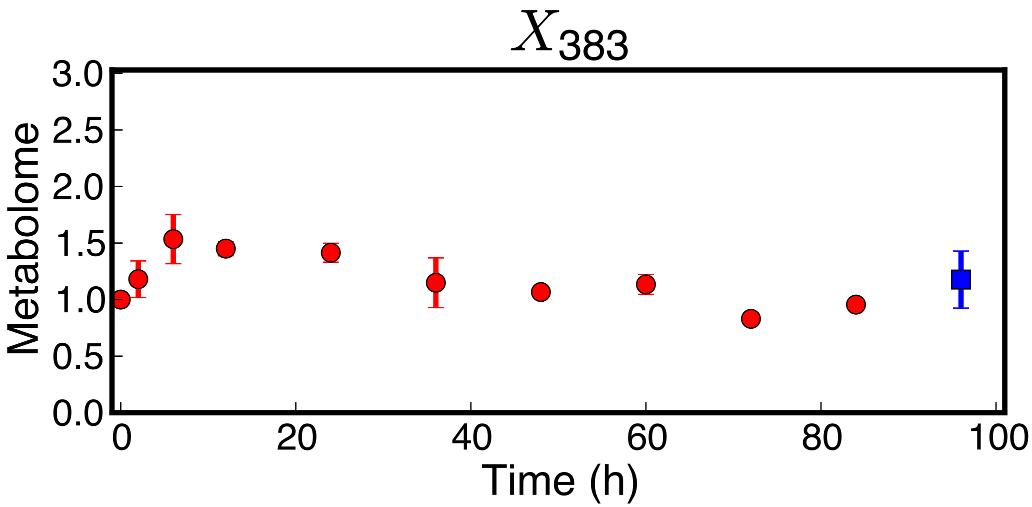

X_{383}
Metabolome
3.0
2.5
2.0
1.5
1.0
0.5
0.0
0
20
40
60
80
100
Time (h)

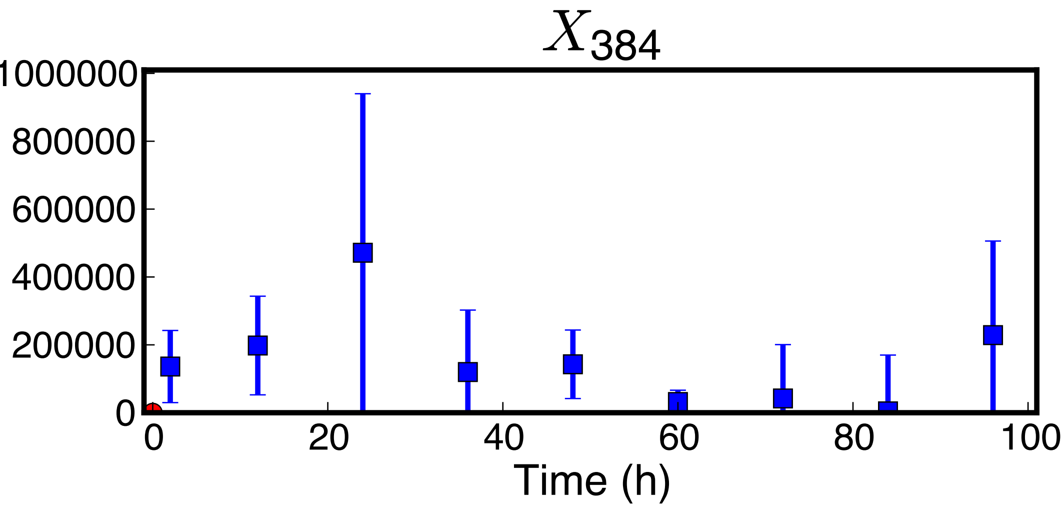

X_{384}
1000000
800000
600000
400000
200000
0
0
20
40
60
80
100
Time (h)

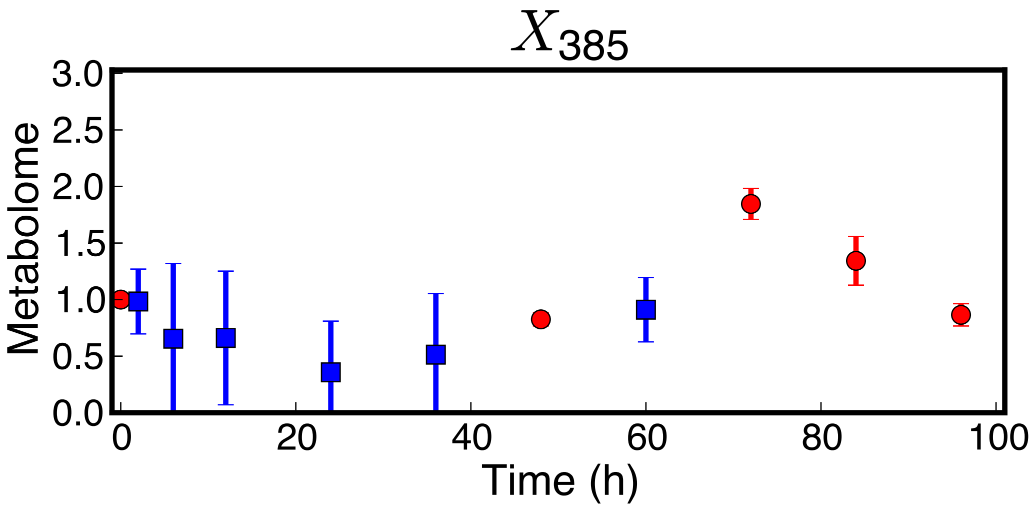

X_{385}
Metabolome
Time (h)
3.0
2.5
2.0
1.5
1.0
0.5
0.0
0
20
40
60
80
100

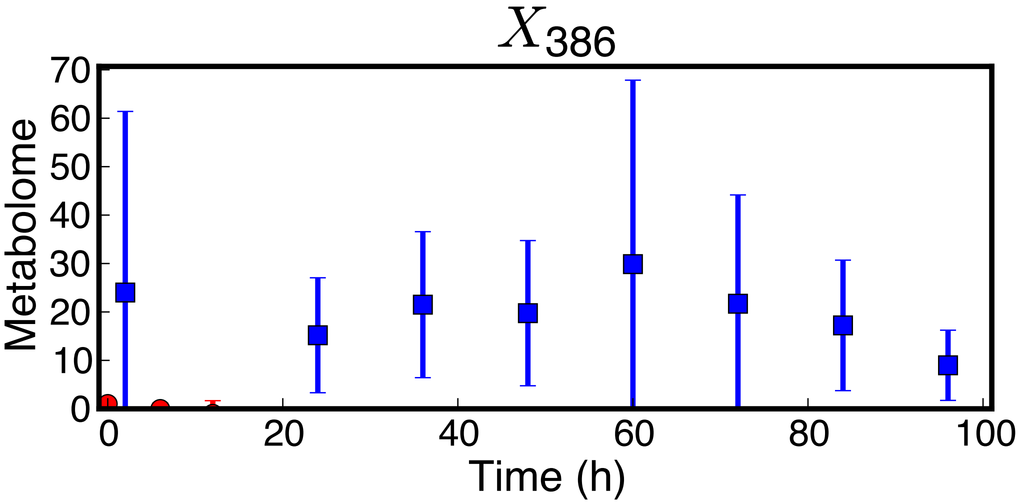
X_{386}
Metabolome
70
60
50
40
30
20
10
0
0
20
40
60
80
100
Time (h)

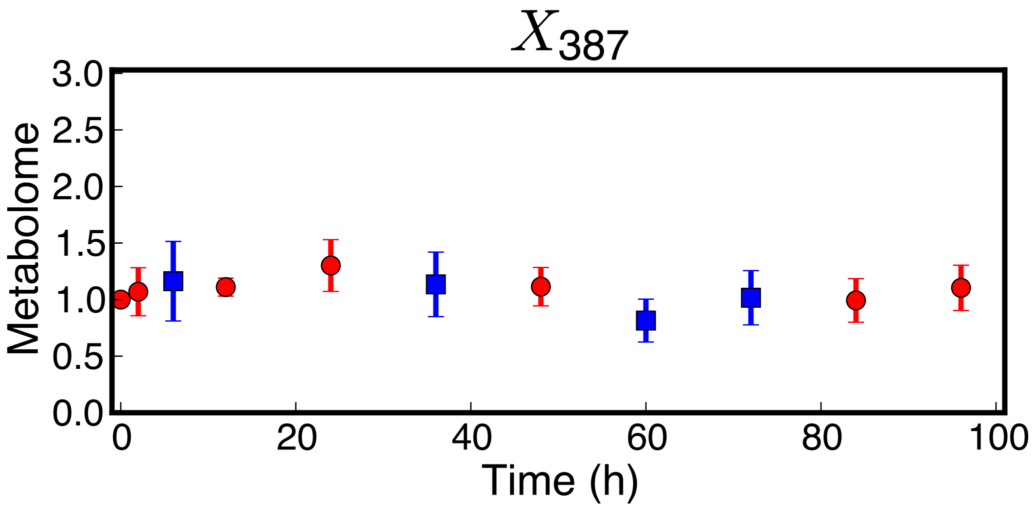

X_{387}
3.0
2.5
2.0
1.5
1.0
0.5
0.0
Metabolome
0
20
40
60
80
100
Time (h)

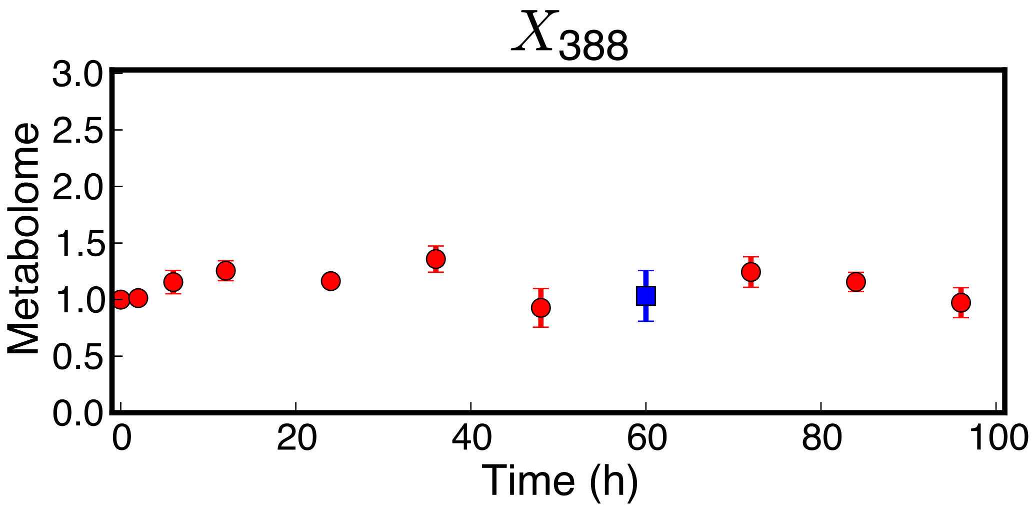

X_{388}
Metabolome
3.0
2.5
2.0
1.5
1.0
0.5
0.0
0
20
40
60
80
100
Time (h)

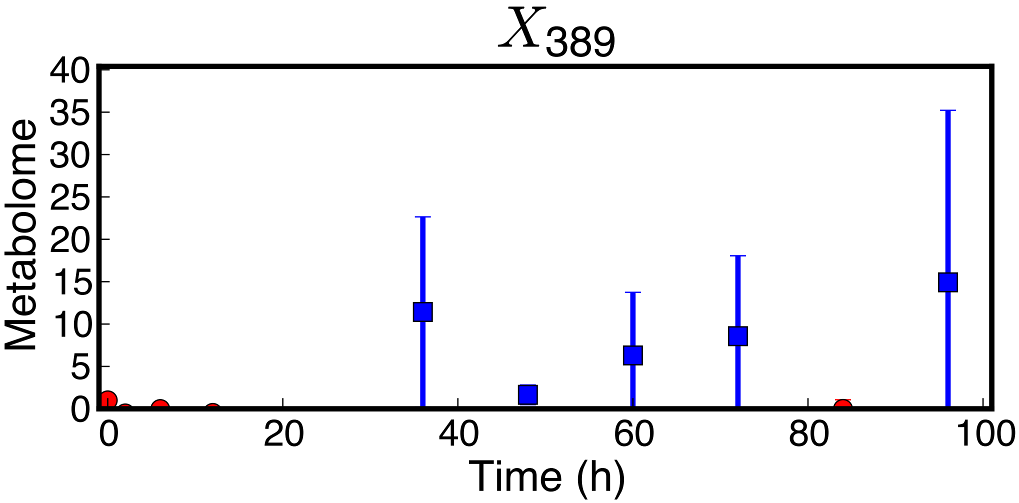

X_{389}
Metabolome
40
35
30
25
20
15
10
5
0
0
20
40
60
80
100
Time (h)

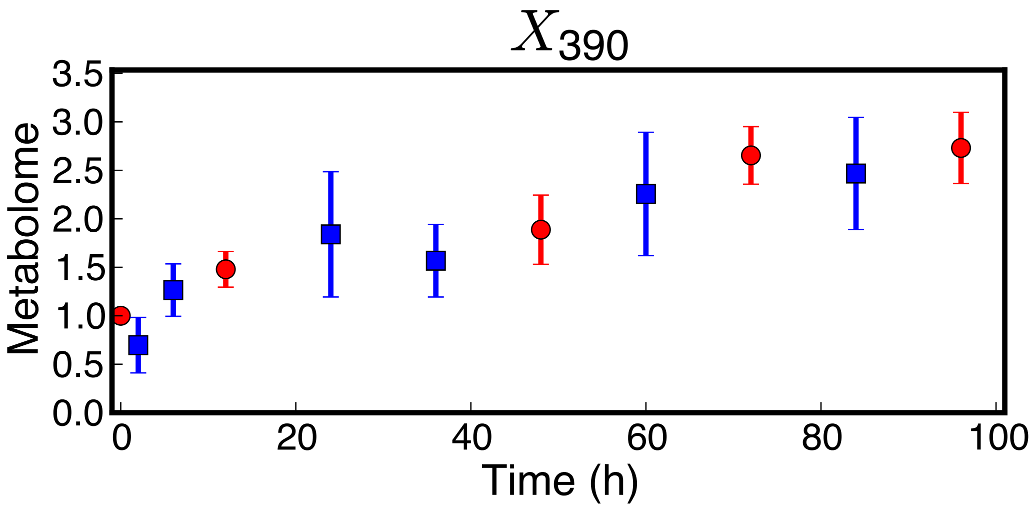

X_{390}
Metabolome
3.5
3.0
2.5
2.0
1.5
1.0
0.5
0.0
0
20
40
60
80
100
Time (h)

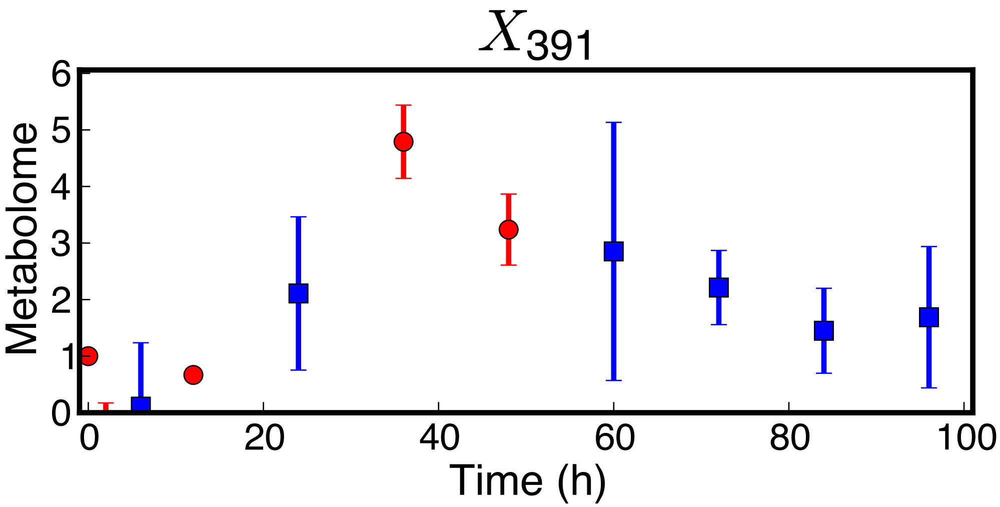

X_{391}
Metabolome
0
1
2
3
4
5
6
0
20
40
60
80
100
Time (h)

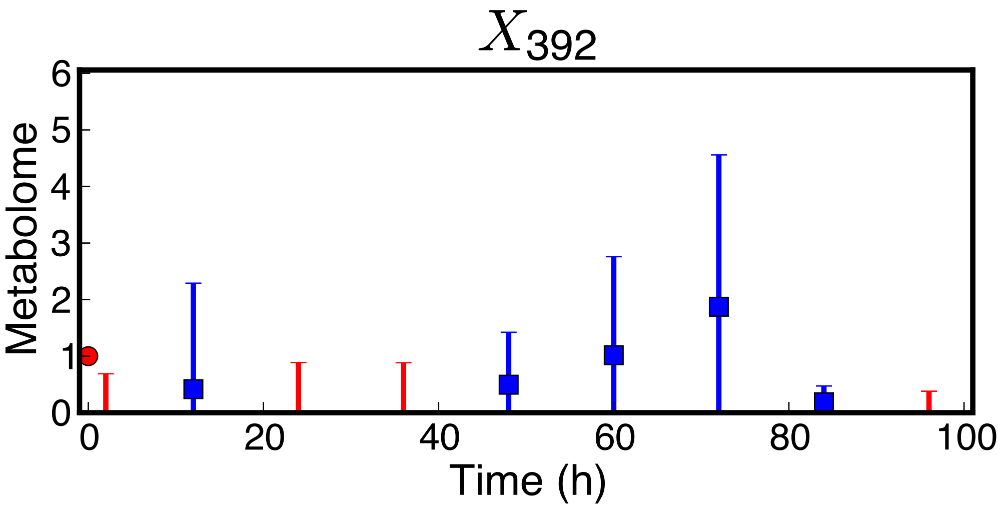

X_{392}
Metabolome
0
1
2
3
4
5
6
0
20
40
60
80
100
Time (h)

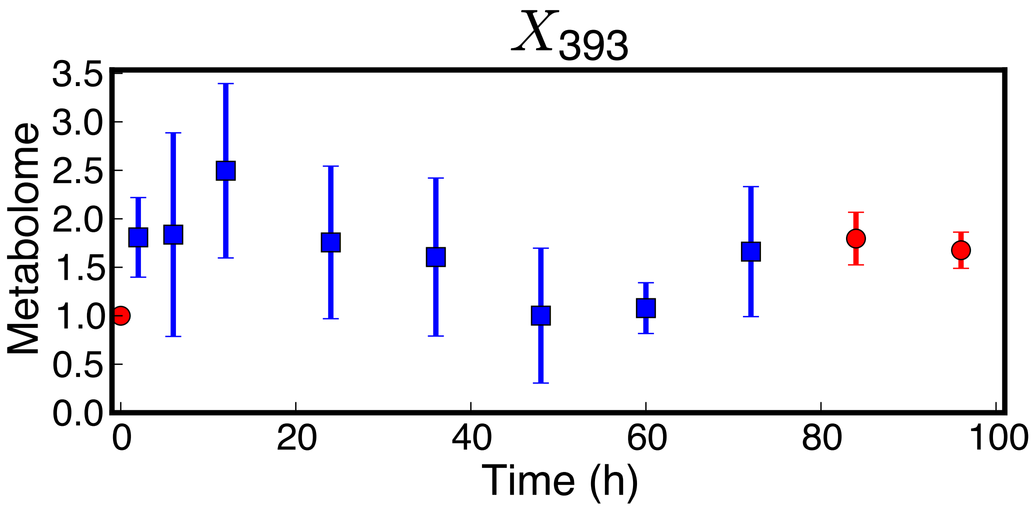
X_{393}
Metabolome
3.5
3.0
2.5
2.0
1.5
1.0
0.5
0.0
0
20
40
60
80
100
Time (h)

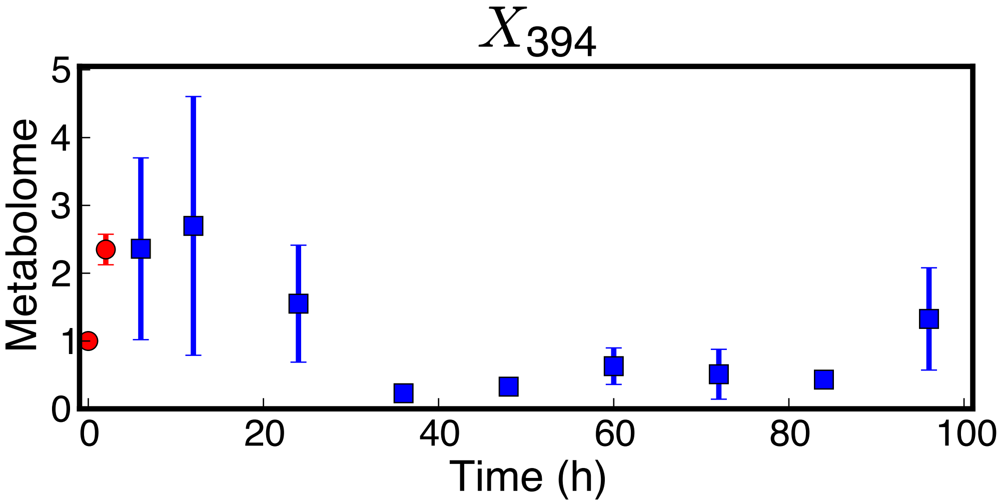

X_{394}
Metabolome
Time (h)
0
1
2
3
4
5
0
20
40
60
80
100

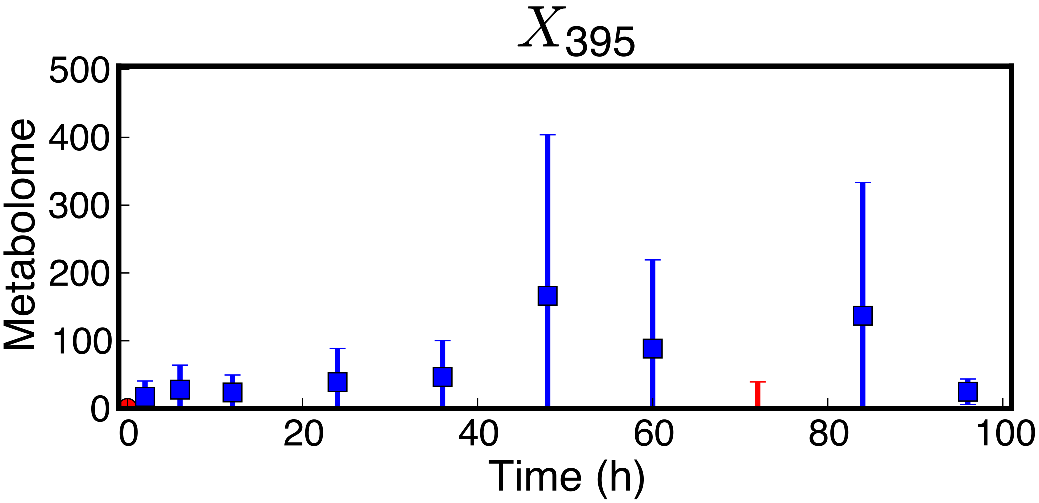

X_{395}
Metabolome
500
400
300
200
100
0
0
20
40
60
80
100
Time (h)

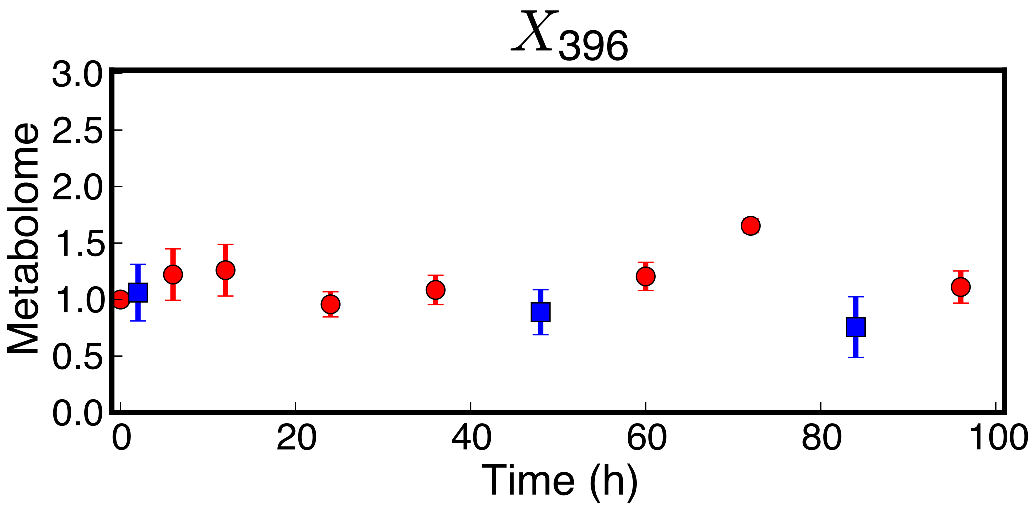
X_{396}
Metabolome
3.0
2.5
2.0
1.5
1.0
0.5
0.0
0
20
40
60
80
100
Time (h)

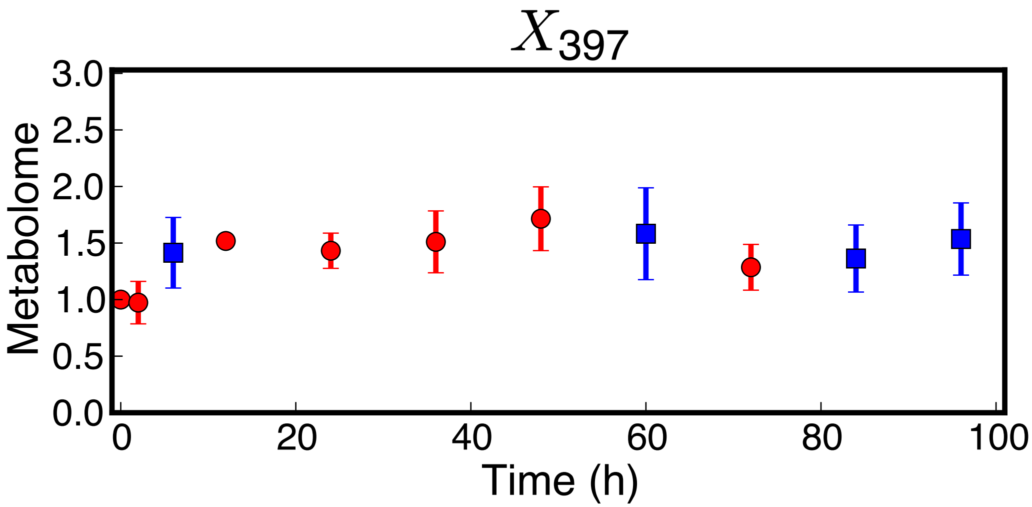
X_{397}
Metabolome
3.0
2.5
2.0
1.5
1.0
0.5
0.0
0
20
40
60
80
100
Time (h)

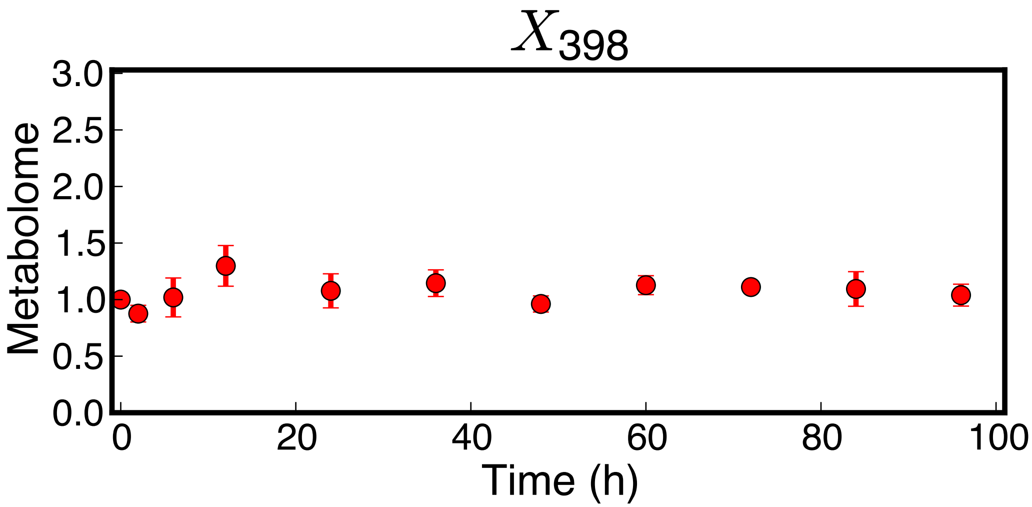
X_{398}
Metabolome
3.0
2.5
2.0
1.5
1.0
0.5
0.0
0
20
40
60
80
100
Time (h)

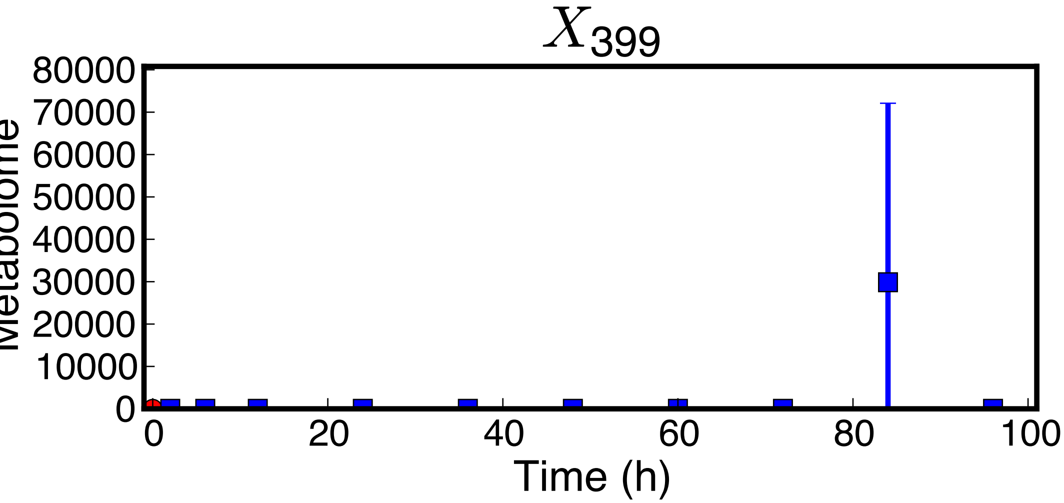

X_{399}
Metabolome
80000
70000
60000
50000
40000
30000
20000
10000
0
0
20
40
60
80
100
Time (h)

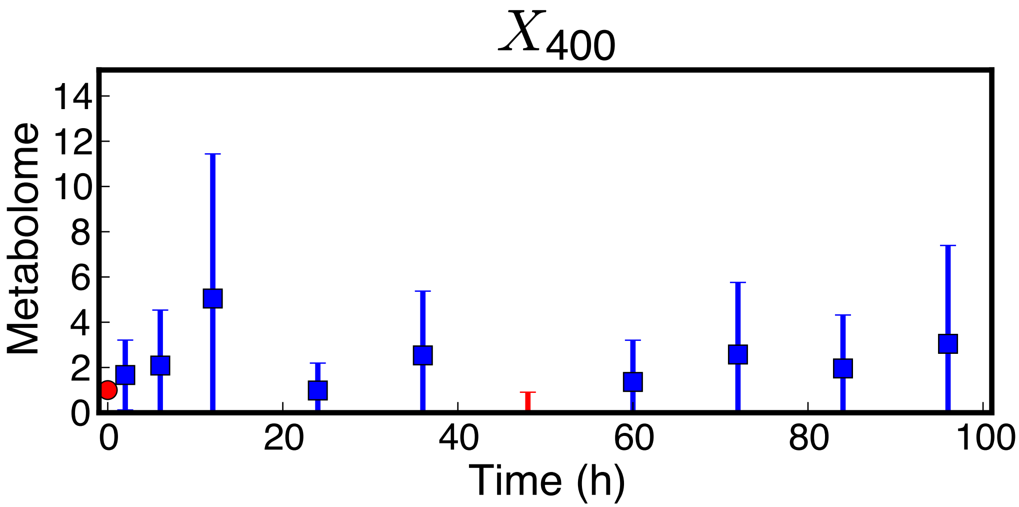
X_{400}
Metabolome
0
2
4
6
8
10
12
14
0
20
40
60
80
100
Time (h)

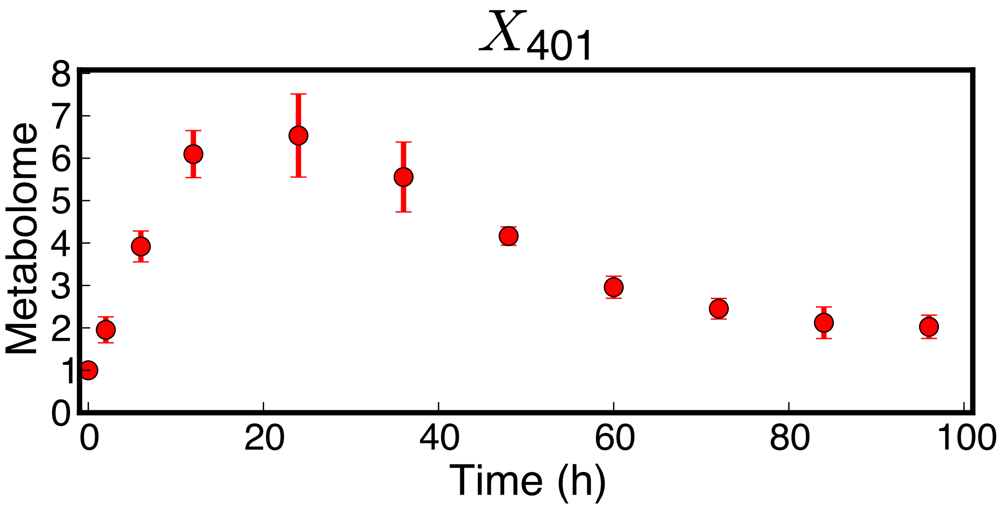

X_{401}
Metabolome
Time (h)
0
1
2
3
4
5
6
7
8
0
20
40
60
80
100

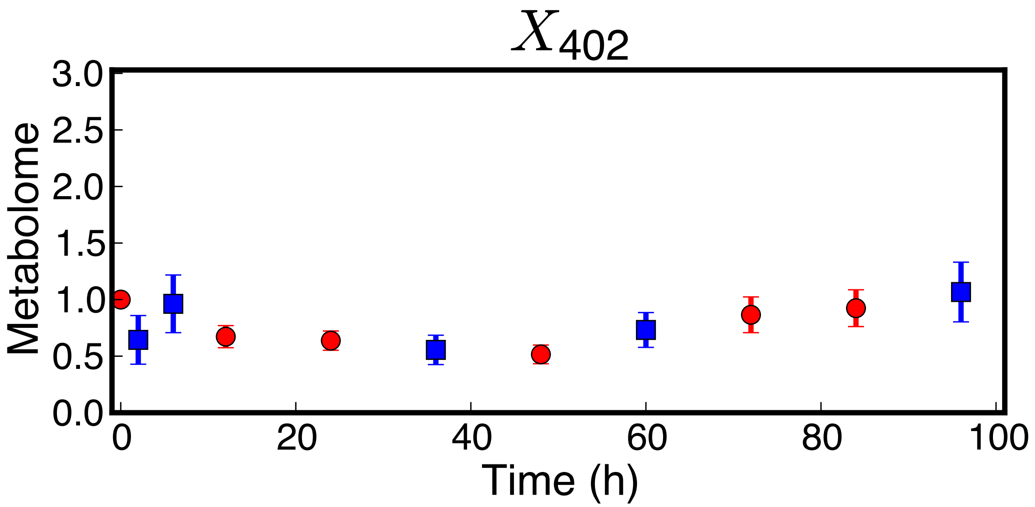

X_{402}
Metabolome
3.0
2.5
2.0
1.5
1.0
0.5
0.0
0
20
40
60
80
100
Time (h)

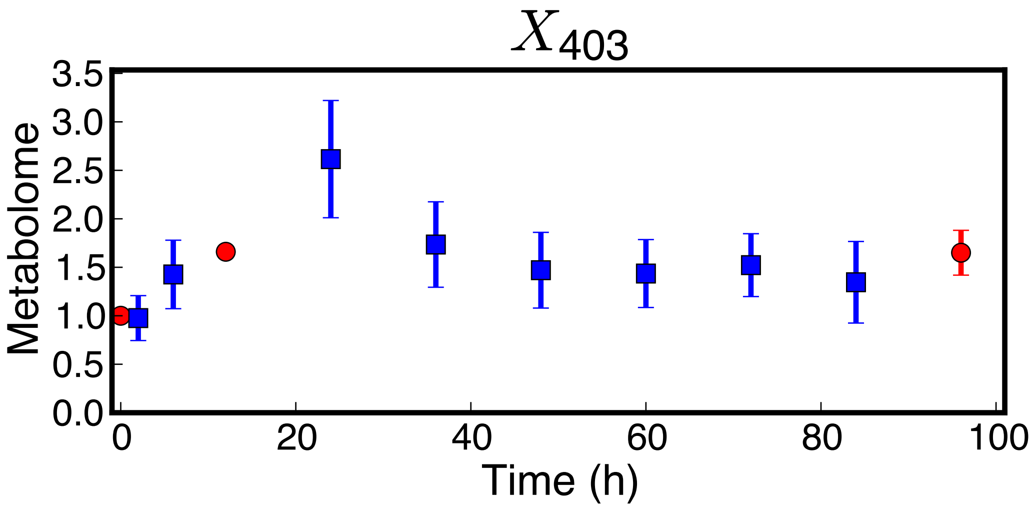

X_{403}
3.5
3.0
2.5
2.0
1.5
1.0
0.5
0.0
Metabolome
0
20
40
60
80
100
Time (h)

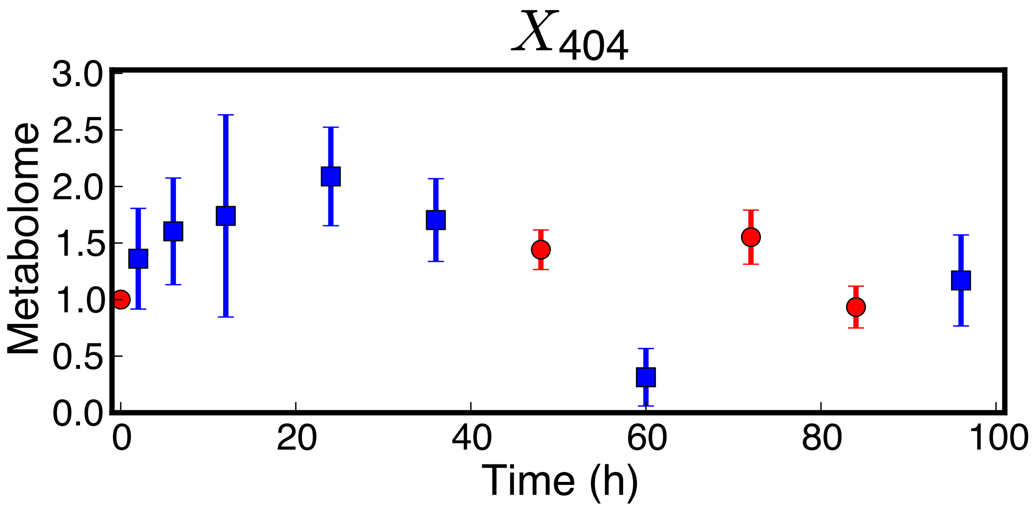

X_{404}
Metabolome
3.0
2.5
2.0
1.5
1.0
0.5
0.0
0
20
40
60
80
100
Time (h)

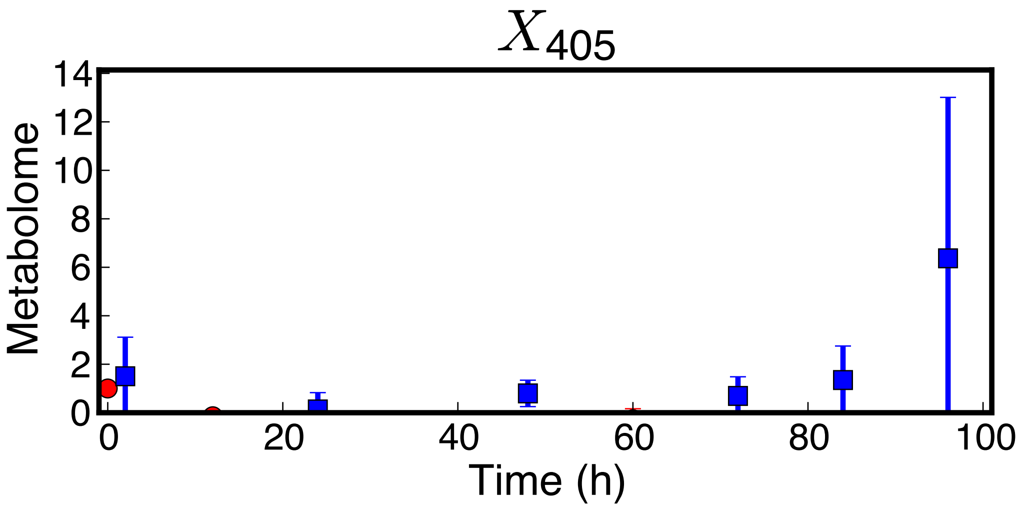

X_{405}
Metabolome
14
12
10
8
6
4
2
0
0
20
40
60
80
100
Time (h)

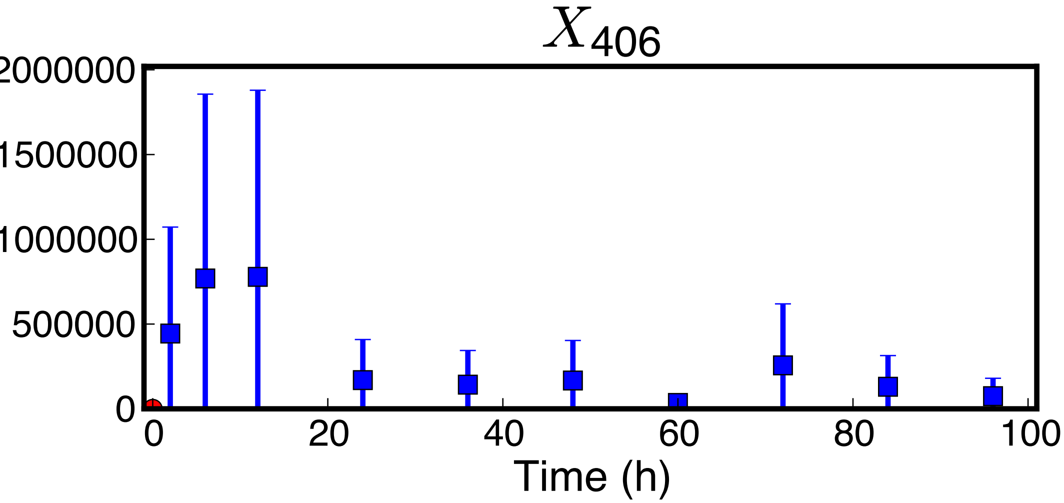

X_{406}
2000000
1500000
1000000
500000
0
0
20
40
60
80
100
Time (h)

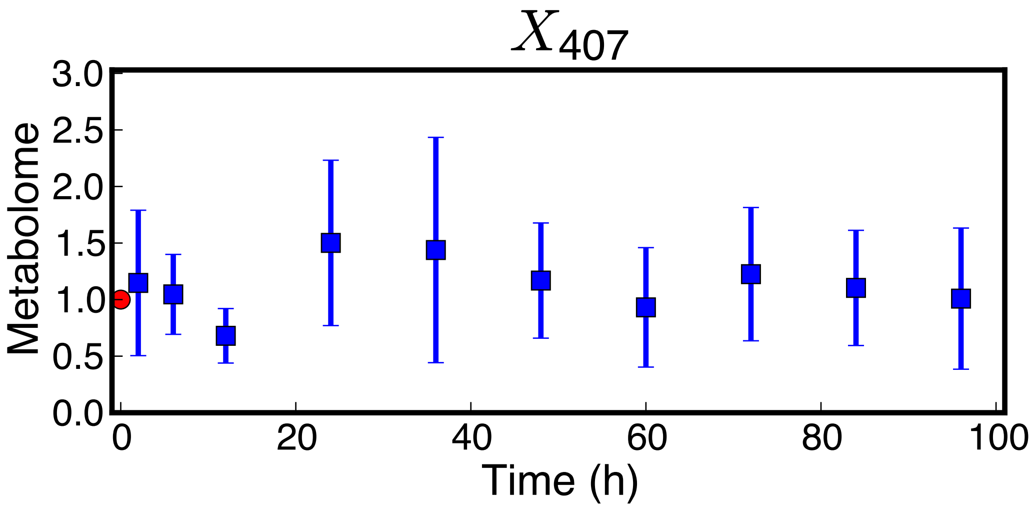

X_{407}
Metabolome
3.0
2.5
2.0
1.5
1.0
0.5
0.0
0
20
40
60
80
100
Time (h)

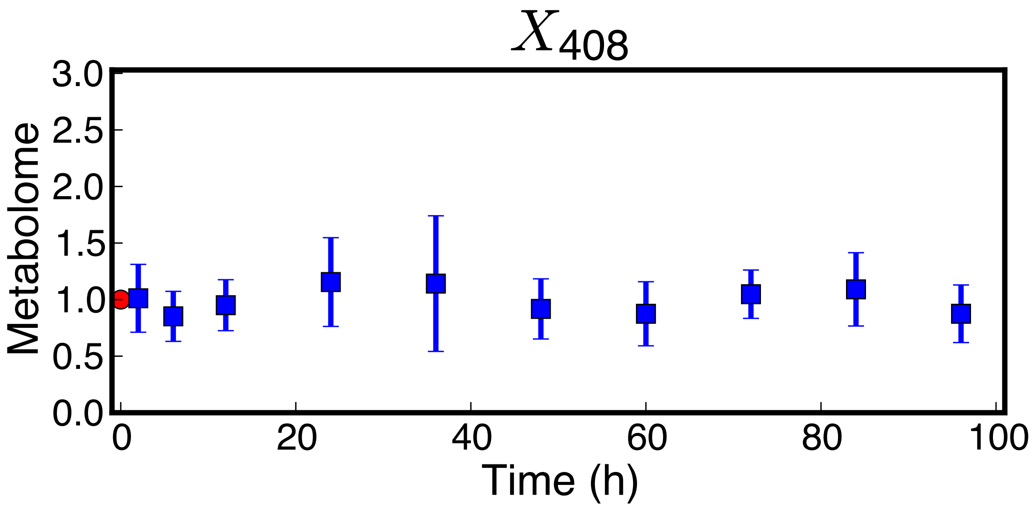

X_{408}
3.0
2.5
2.0
1.5
1.0
0.5
0.0
Metabolome
0
20
40
60
80
100
Time (h)

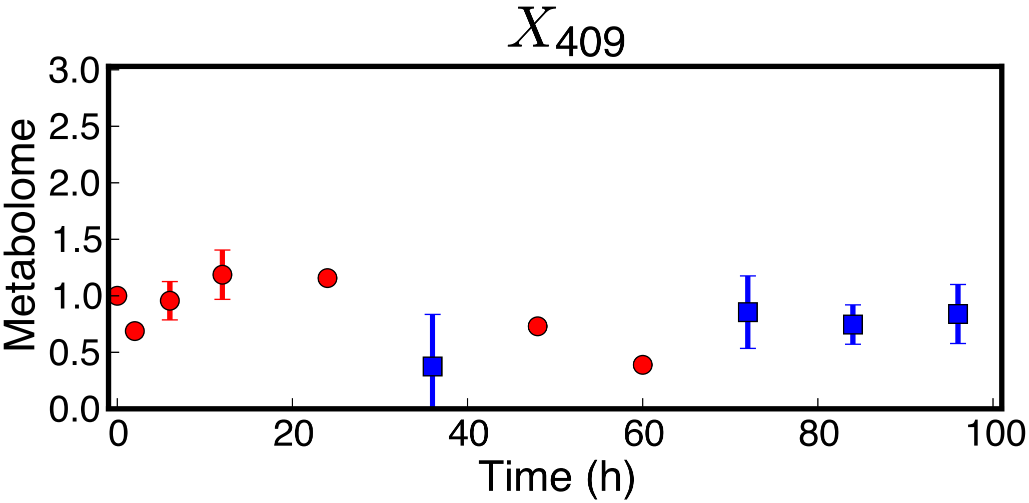

X_{409}
Metabolome
3.0
2.5
2.0
1.5
1.0
0.5
0.0
0
20
40
60
80
100
Time (h)

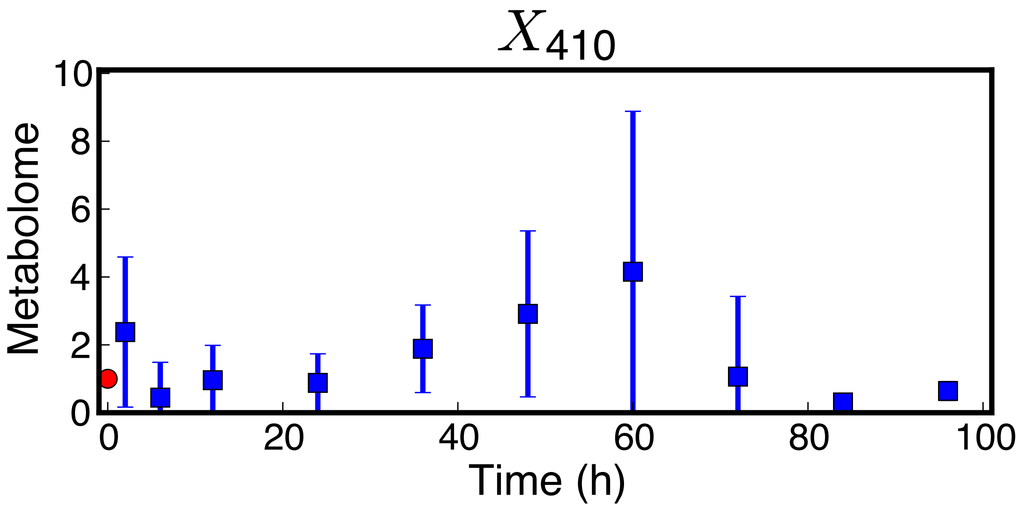

X_{410}
Metabolome
Time (h)
0
2
4
6
8
10
0
20
40
60
80
100

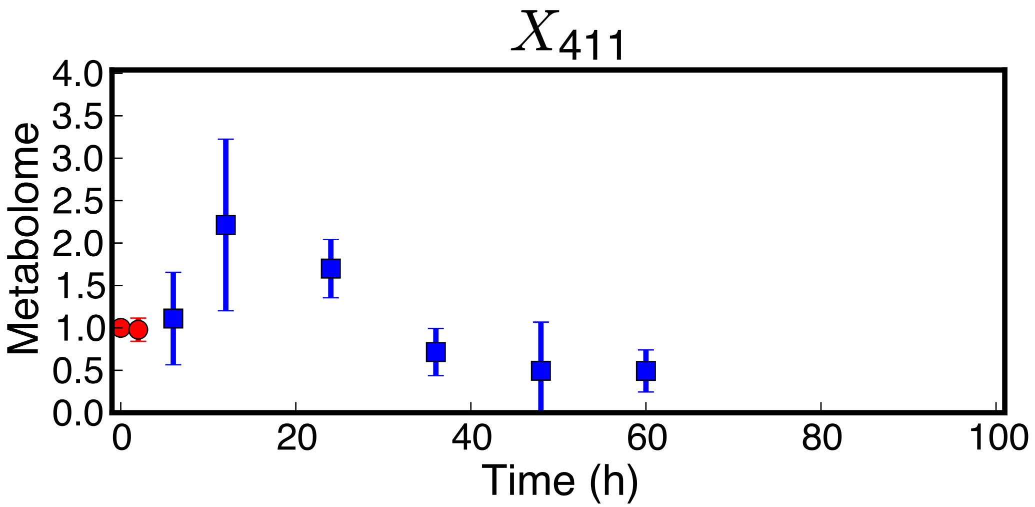

X_{411}
Metabolome
4.0
3.5
3.0
2.5
2.0
1.5
1.0
0.5
0.0
0
20
40
60
80
100
Time (h)

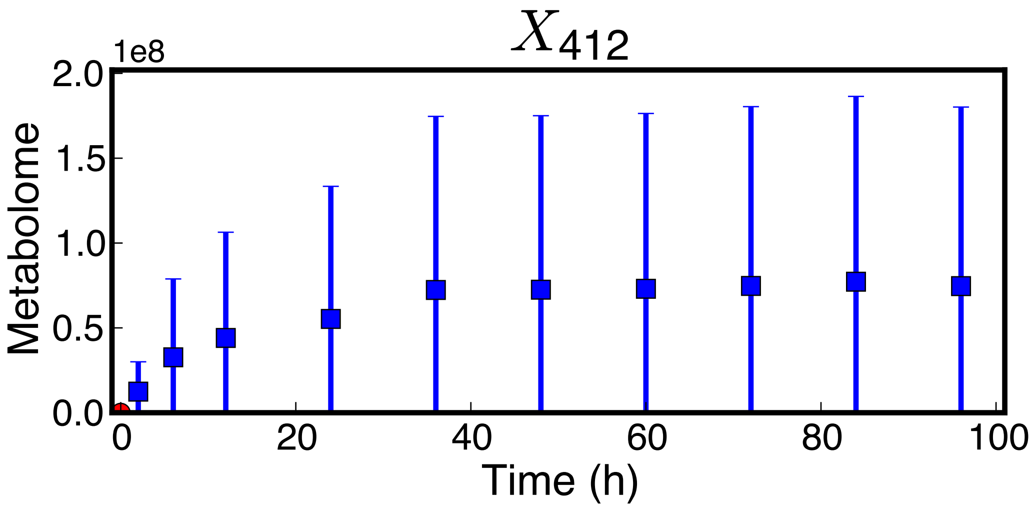

X_{412}
1e8
2.0
1.5
1.0
0.5
0.0
Metabolome
0
20
40
60
80
100
Time (h)

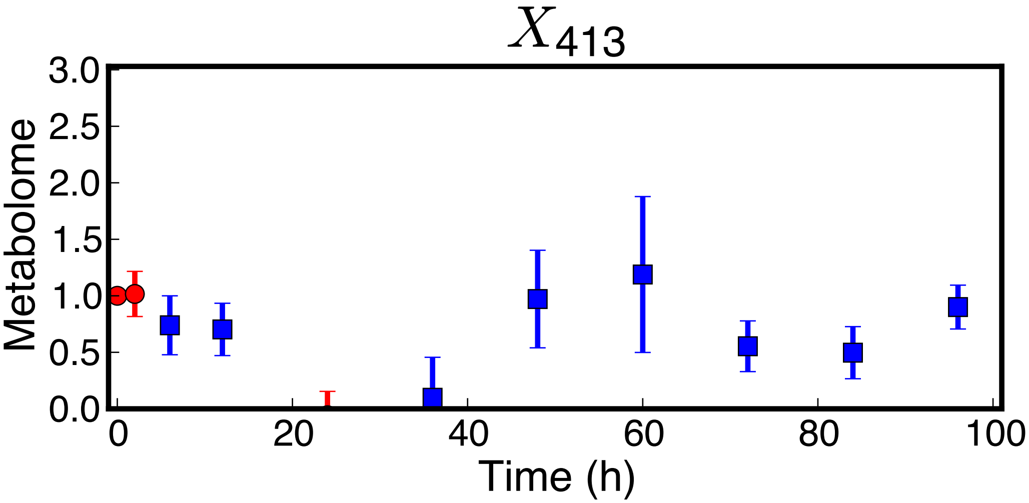

X_{413}
3.0
2.5
2.0
1.5
1.0
0.5
0.0
Metabolome
0
20
40
60
80
100
Time (h)

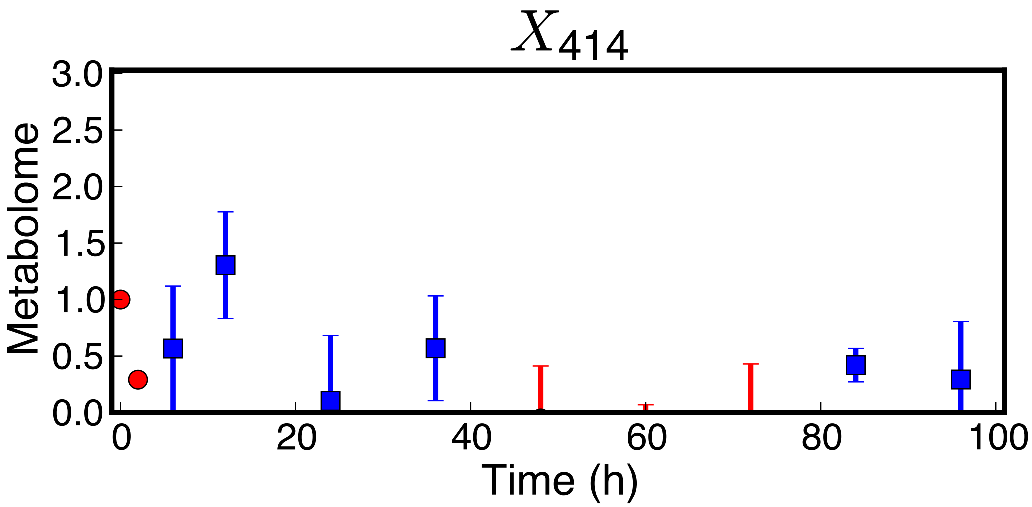

X_{414}
3.0
2.5
2.0
1.5
1.0
0.5
0.0
Metabolome
0
20
40
60
80
100
Time (h)

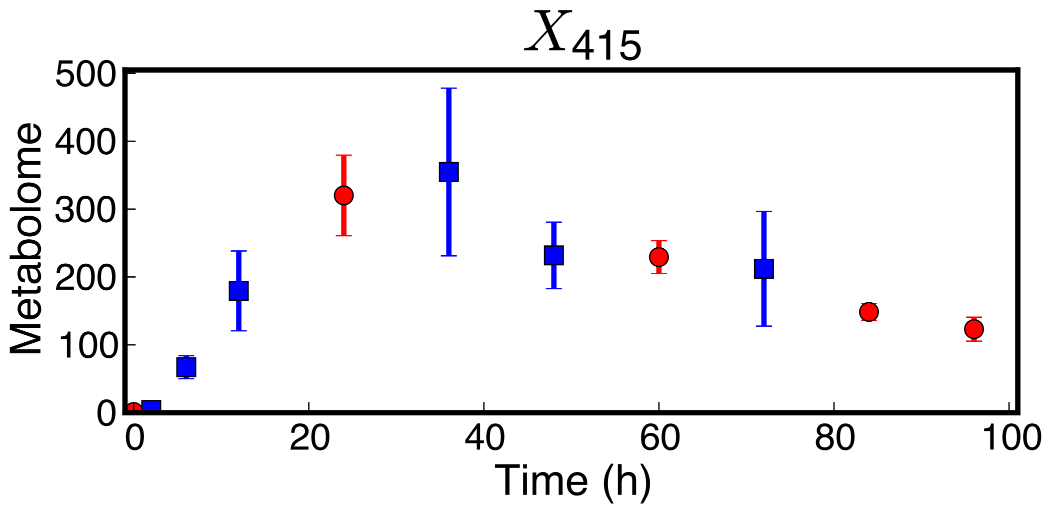
X_{415}
Metabolome
Time (h)
0
100
200
300
400
500
0
20
40
60
80
100

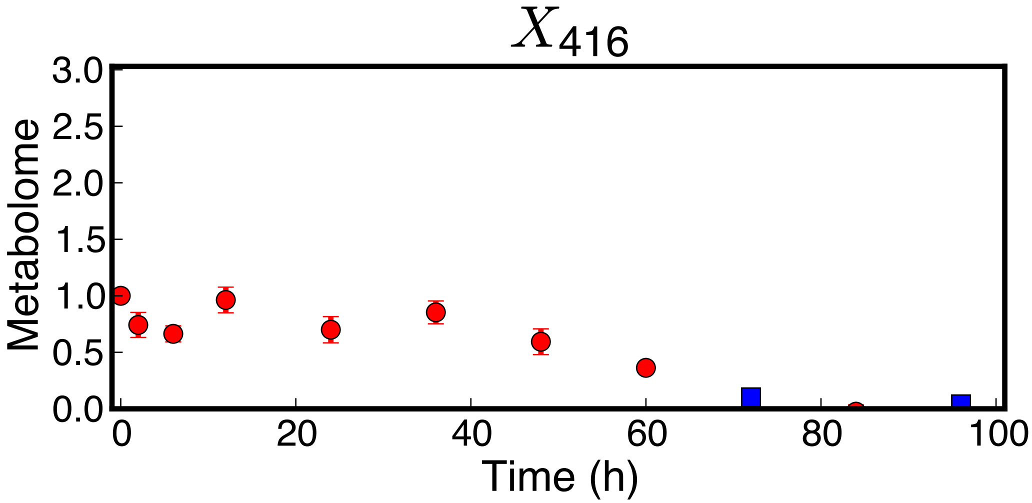

X_{416}
3.0
2.5
2.0
1.5
1.0
0.5
0.0
Metabolome
0
20
40
60
80
100
Time (h)

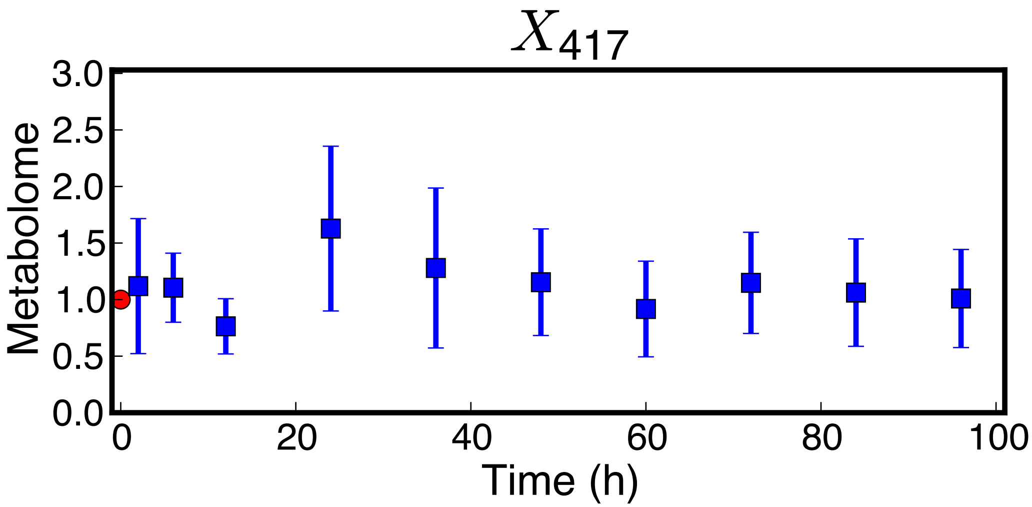
X_{417}
Metabolome
3.0
2.5
2.0
1.5
1.0
0.5
0.0
0
20
40
60
80
100
Time (h)

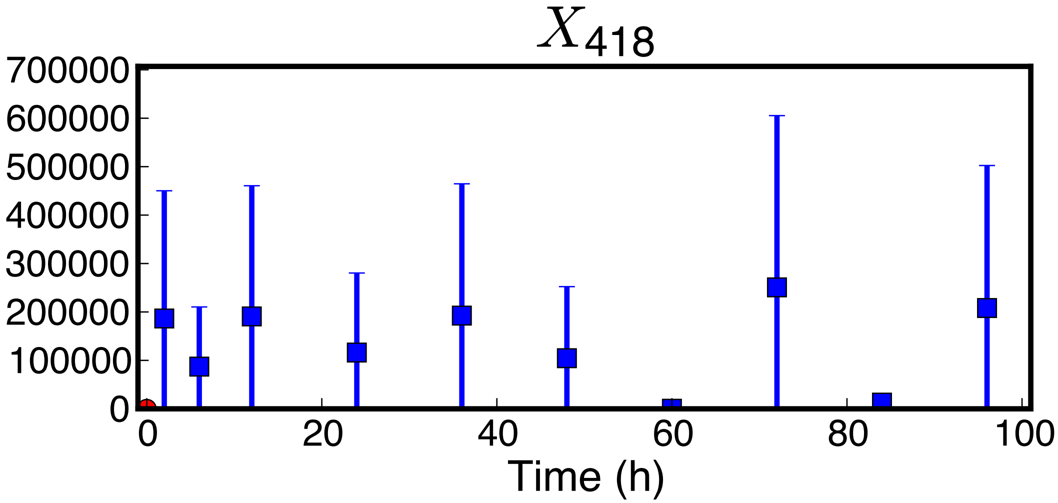
X_{418}
700000
600000
500000
400000
300000
200000
100000
0
0
20
40
60
80
100
Time (h)

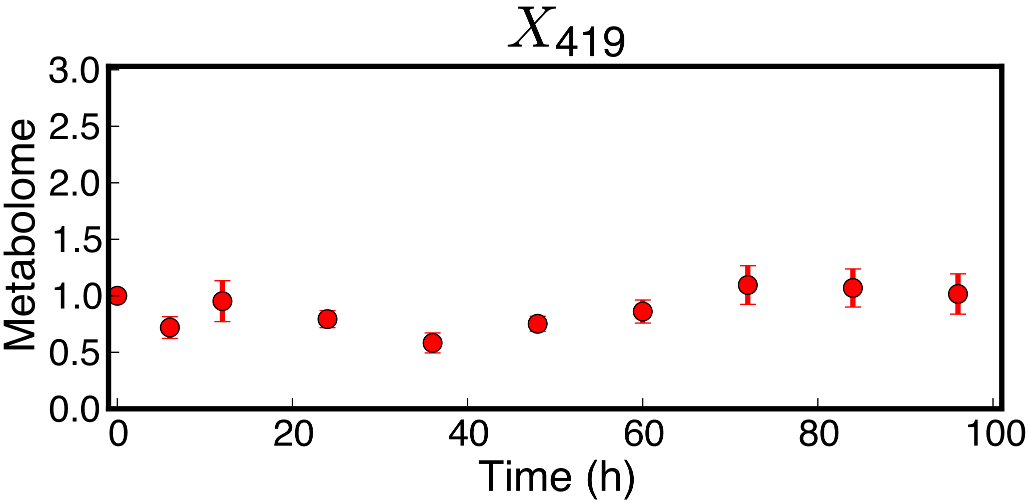

X_{419}
Metabolome
3.0
2.5
2.0
1.5
1.0
0.5
0.0
0
20
40
60
80
100
Time (h)

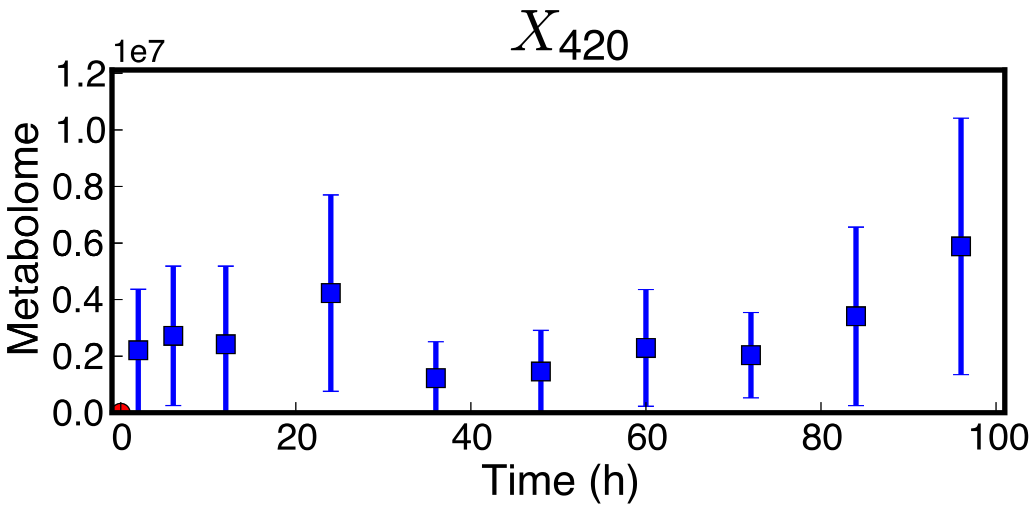
X_{420}
1e7
1.2
1.0
0.8
0.6
0.4
0.2
0.0
Metabolome
0
20
40
60
80
100
Time (h)

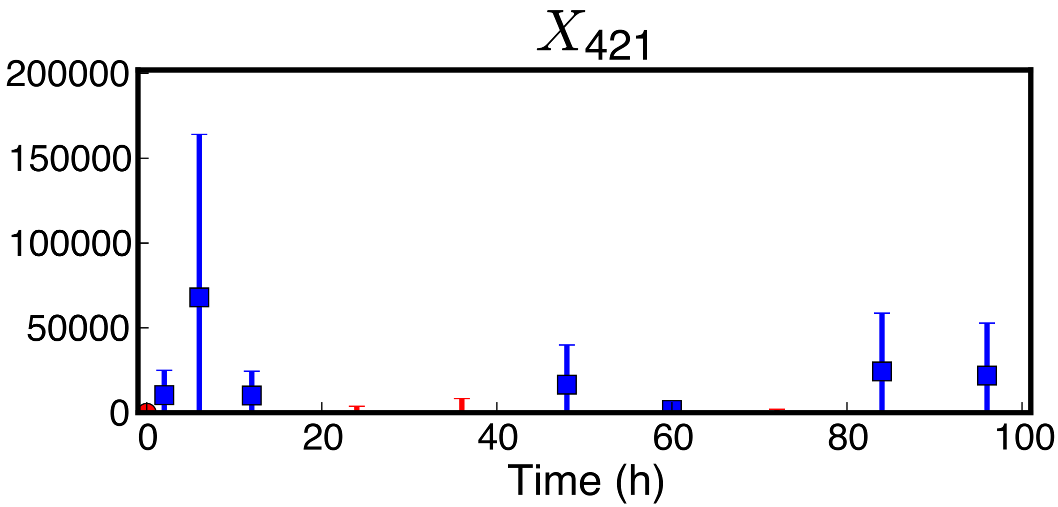
X_{421}
200000
150000
100000
50000
0
0
20
40
60
80
100
Time (h)

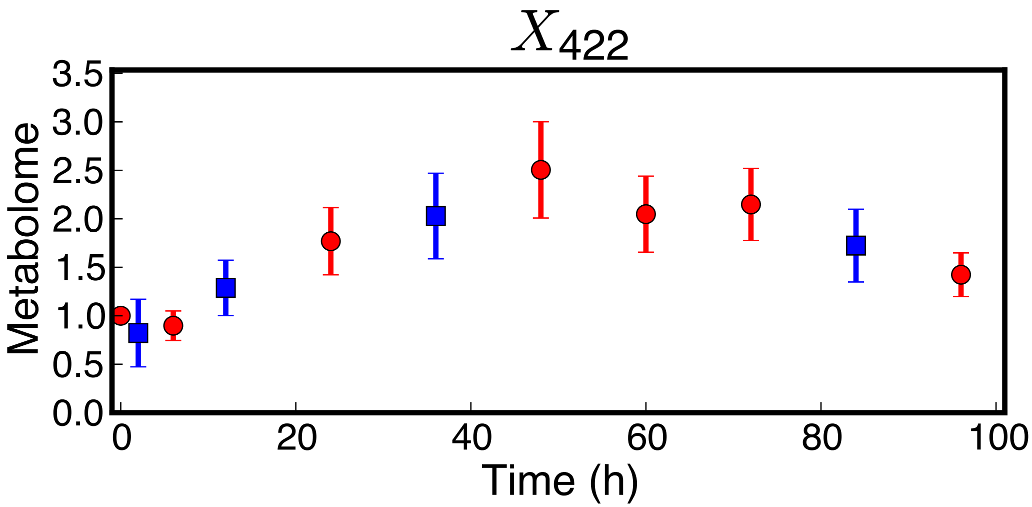
X_{422}
Metabolome
Time (h)
3.5
3.0
2.5
2.0
1.5
1.0
0.5
0.0
0
20
40
60
80
100

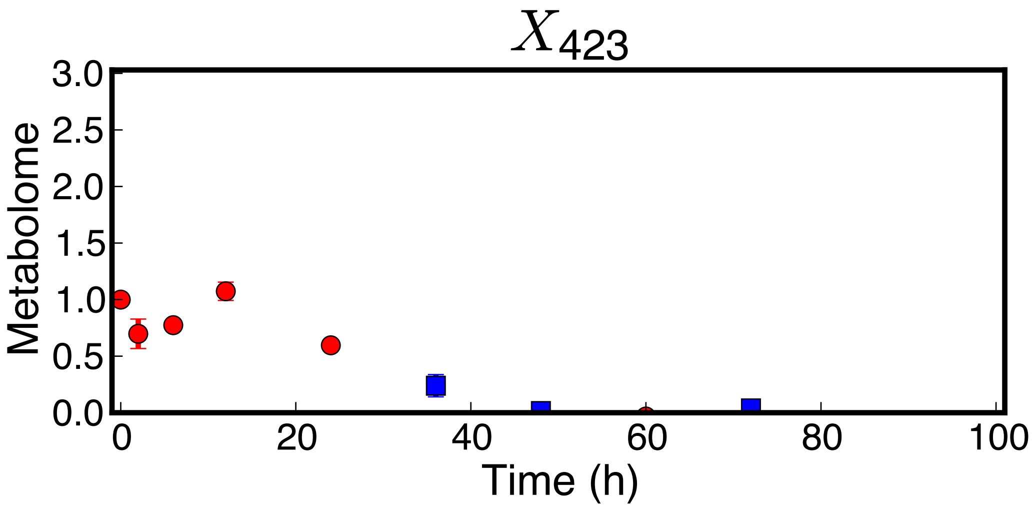

X_{423}
Metabolome
3.0
2.5
2.0
1.5
1.0
0.5
0.0
0
20
40
60
80
100
Time (h)

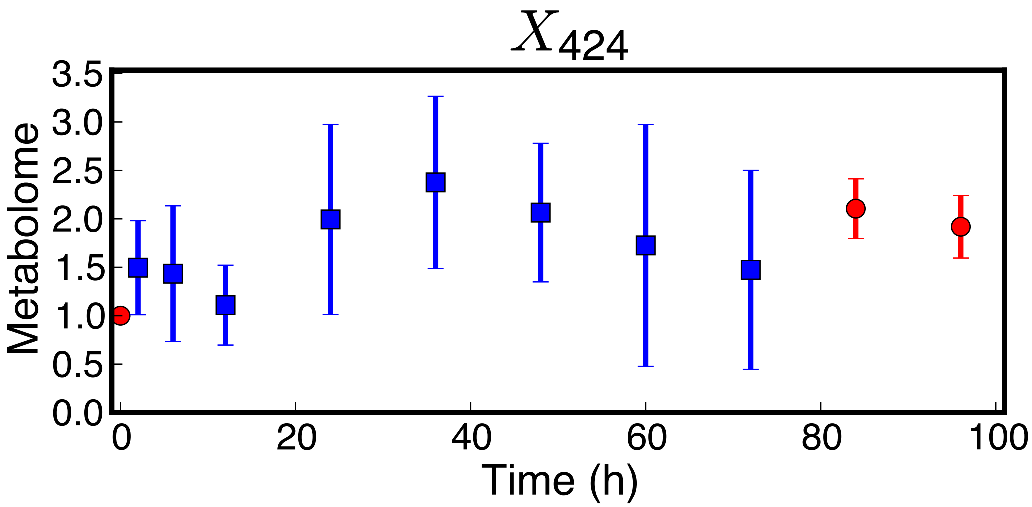

X_{424}
3.5
3.0
2.5
2.0
1.5
1.0
0.5
0.0
Metabolome
0
20
40
60
80
100
Time (h)

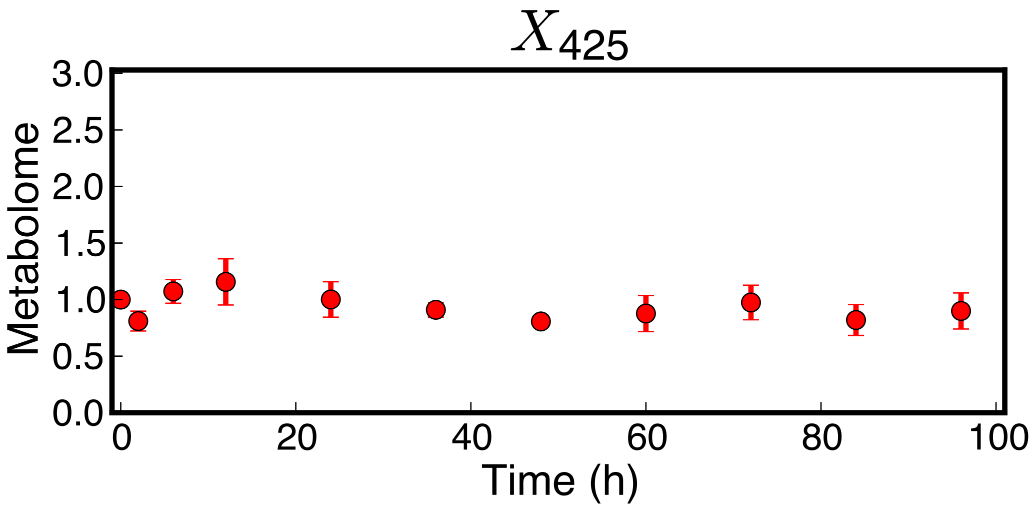

X_{425}
Metabolome
3.0
2.5
2.0
1.5
1.0
0.5
0.0
0
20
40
60
80
100
Time (h)

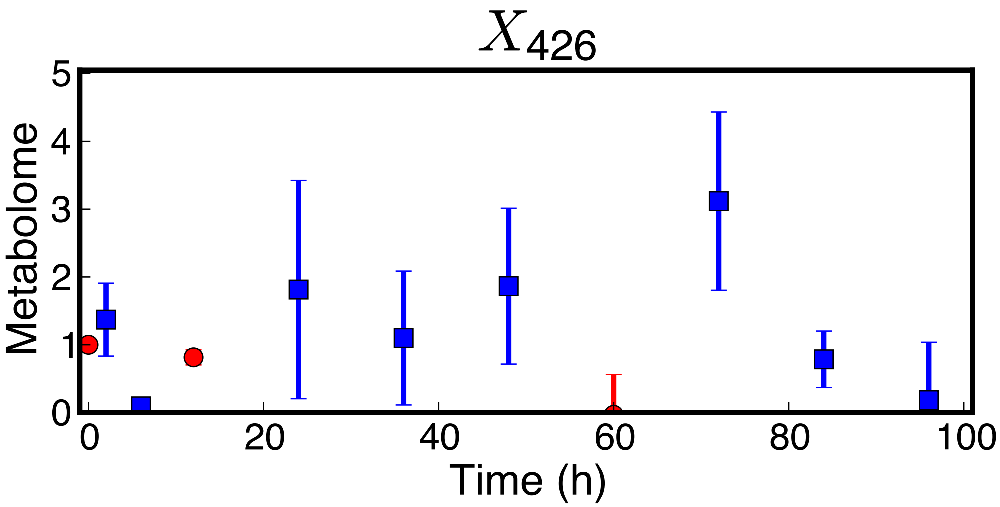

X_{426}
Metabolome
Time (h)
0
1
2
3
4
5
0
20
40
60
80
100

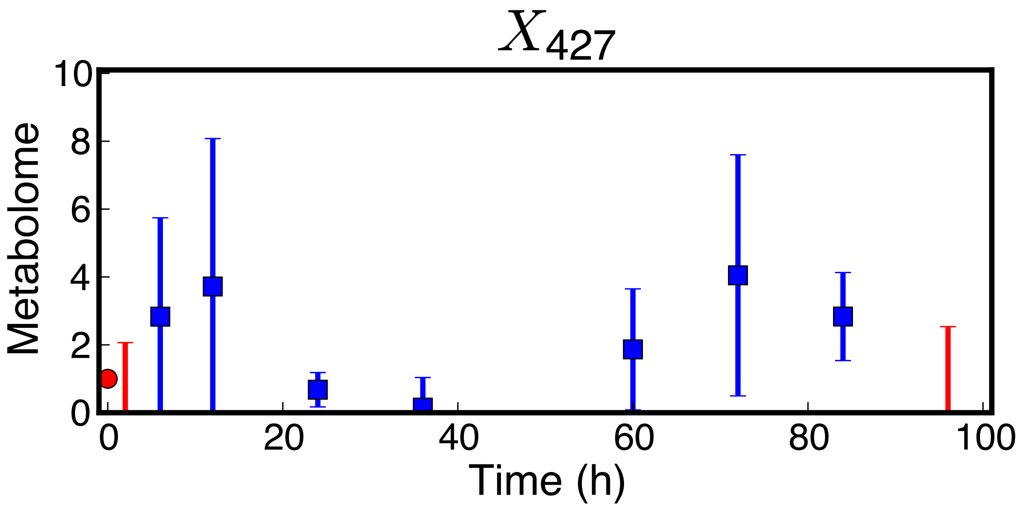

X_{427}
Metabolome
Time (h)
0
2
4
6
8
10
0
20
40
60
80
100

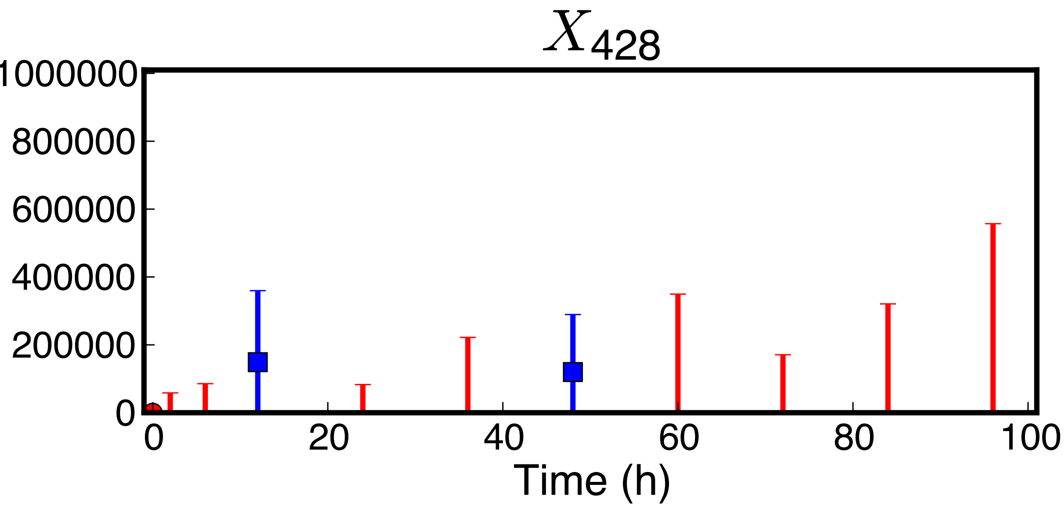

X_{428}
1000000
800000
600000
400000
200000
0
0
20
40
60
80
100
Time (h)

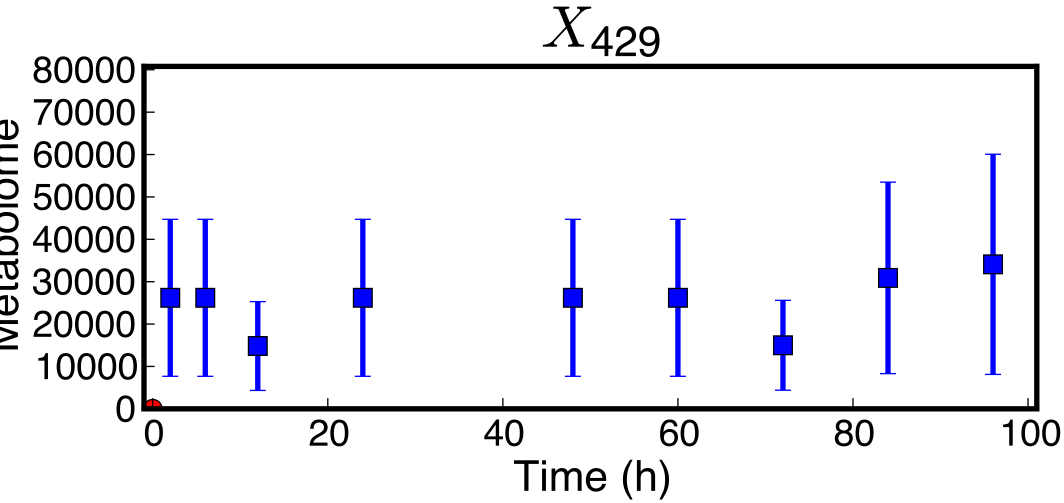

X_{429}
Metabolome
0
10000
20000
30000
40000
50000
60000
70000
80000
0
20
40
60
80
100
Time (h)

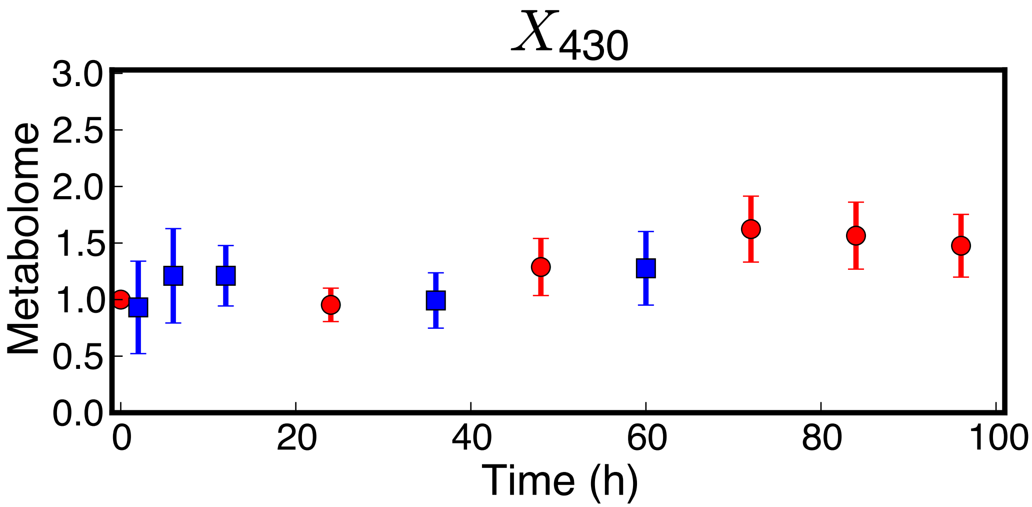

X_{430}
3.0
2.5
2.0
1.5
1.0
0.5
0.0
Metabolome
0
20
40
60
80
100
Time (h)

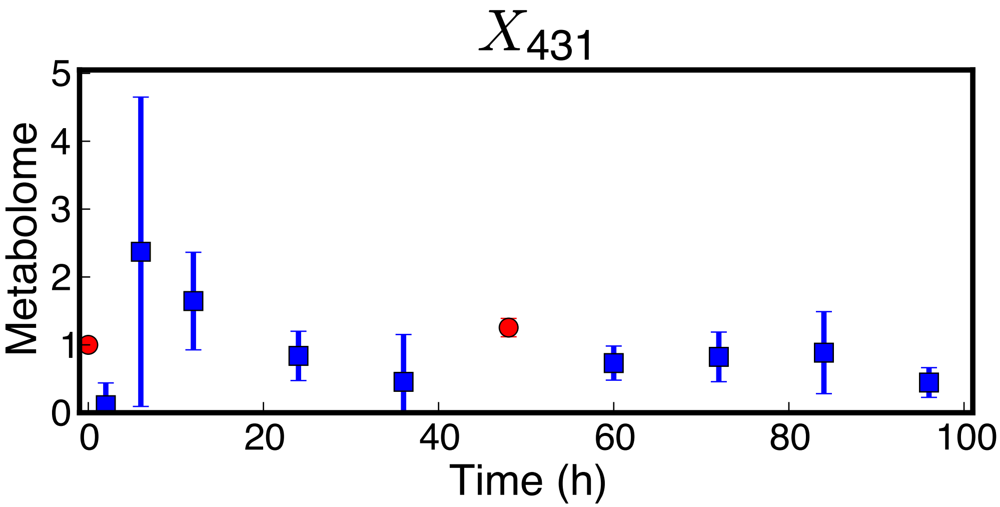

X_{431}
Metabolome
Time (h)
0
1
2
3
4
5
0
20
40
60
80
100

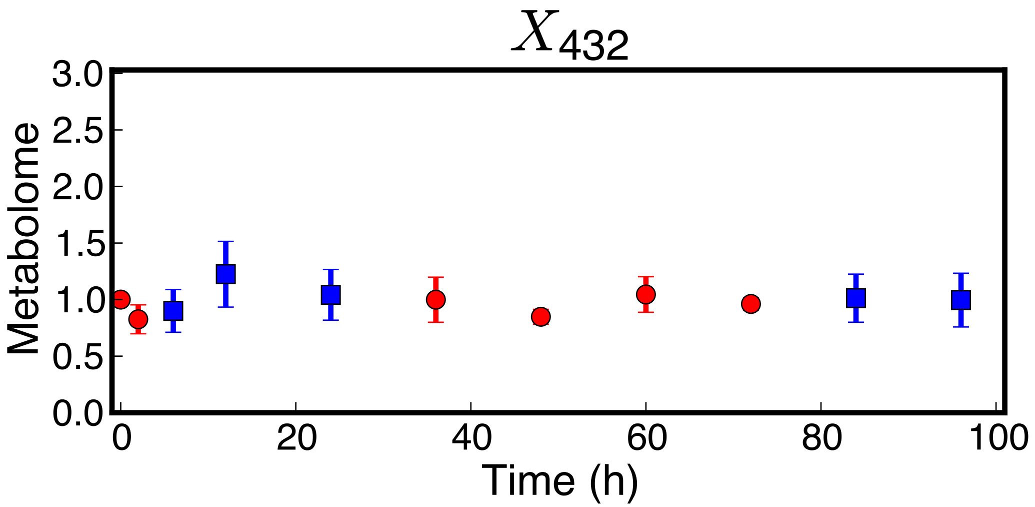

X_{432}
Metabolome
3.0
2.5
2.0
1.5
1.0
0.5
0.0
0
20
40
60
80
100
Time (h)

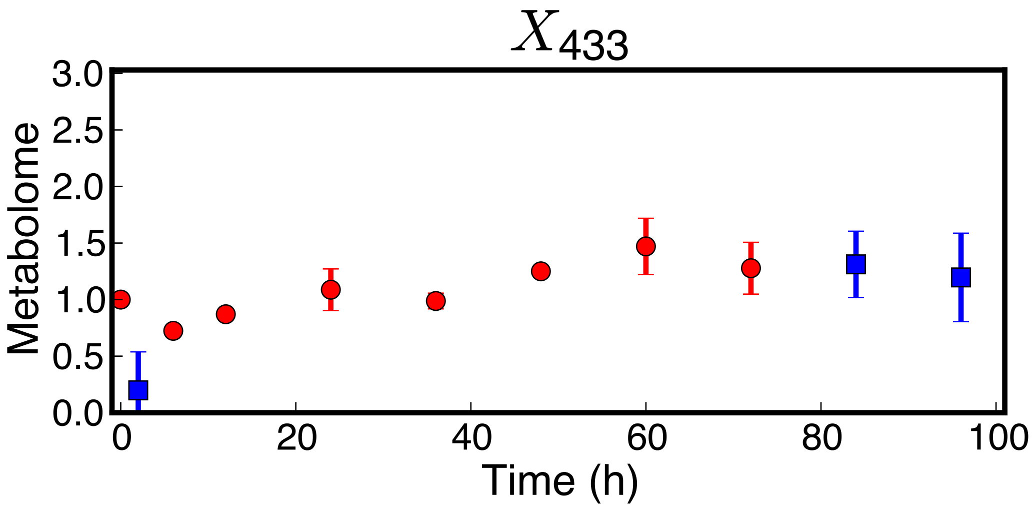
X_{433}
Metabolome
3.0
2.5
2.0
1.5
1.0
0.5
0.0
0
20
40
60
80
100
Time (h)

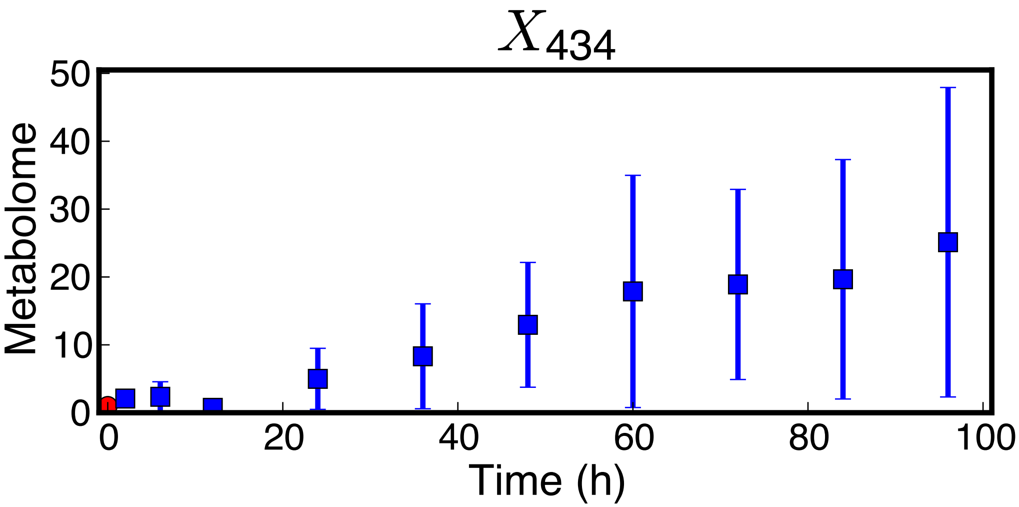

X_{434}
Metabolome
Time (h)
0
10
20
30
40
50
0
20
40
60
80
100

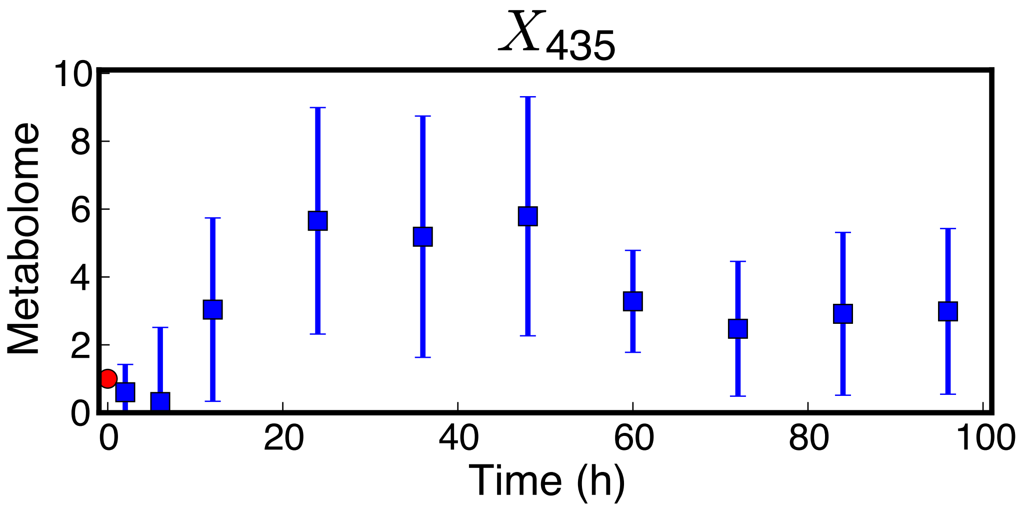

X_{435}
Metabolome
Time (h)
0
2
4
6
8
10
0
20
40
60
80
100

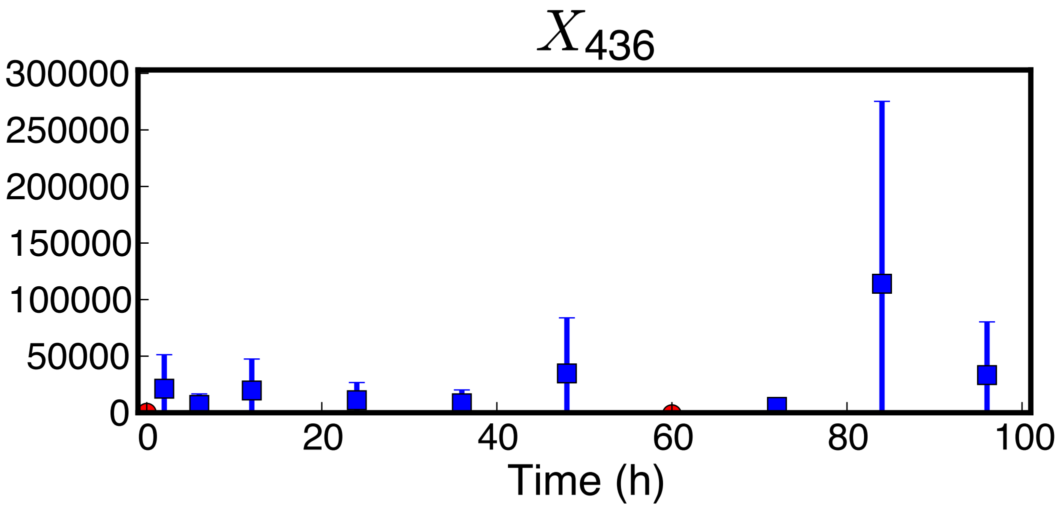

X_{436}
300000
250000
200000
150000
100000
50000
0
0
20
40
60
80
100
Time (h)

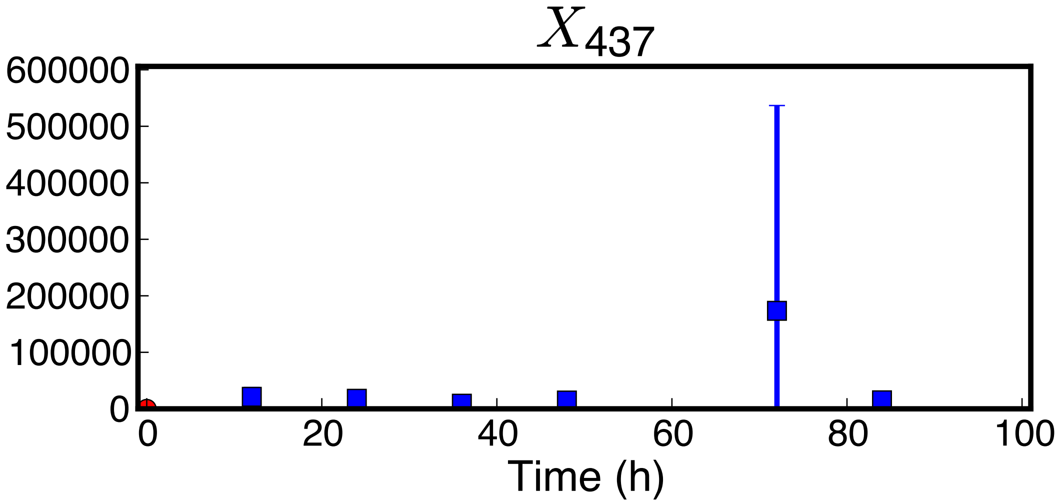

X_{437}
600000
500000
400000
300000
200000
100000
0
0
20
40
60
80
100
Time (h)

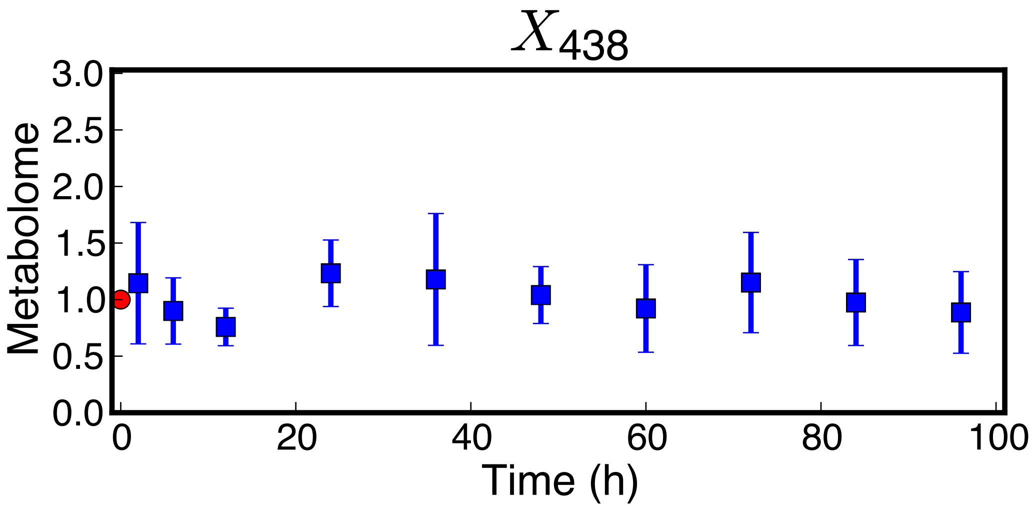

X_{438}
Metabolome
3.0
2.5
2.0
1.5
1.0
0.5
0.0
0
20
40
60
80
100
Time (h)

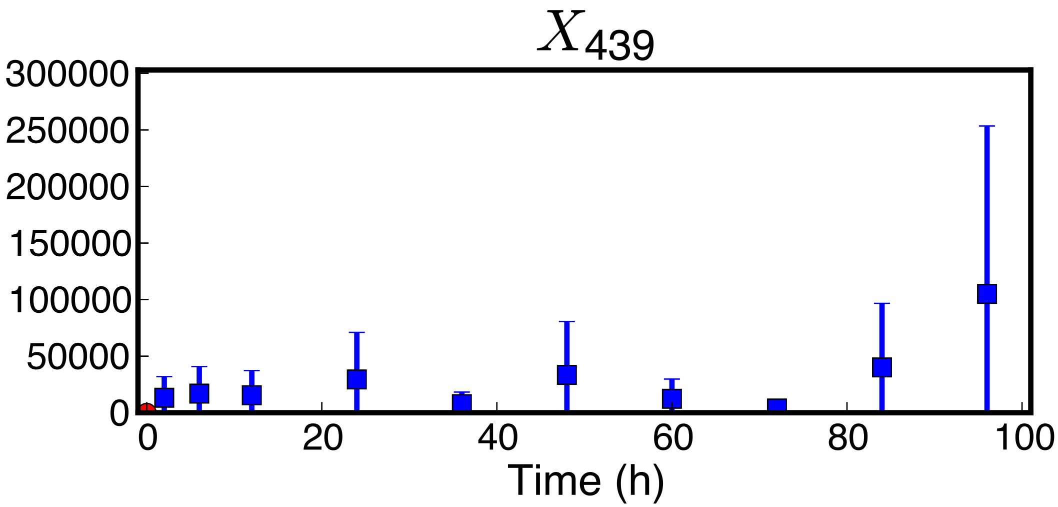

X439
300000
250000
200000
150000
100000
50000
0
0
20
40
60
80
100
Time (h)

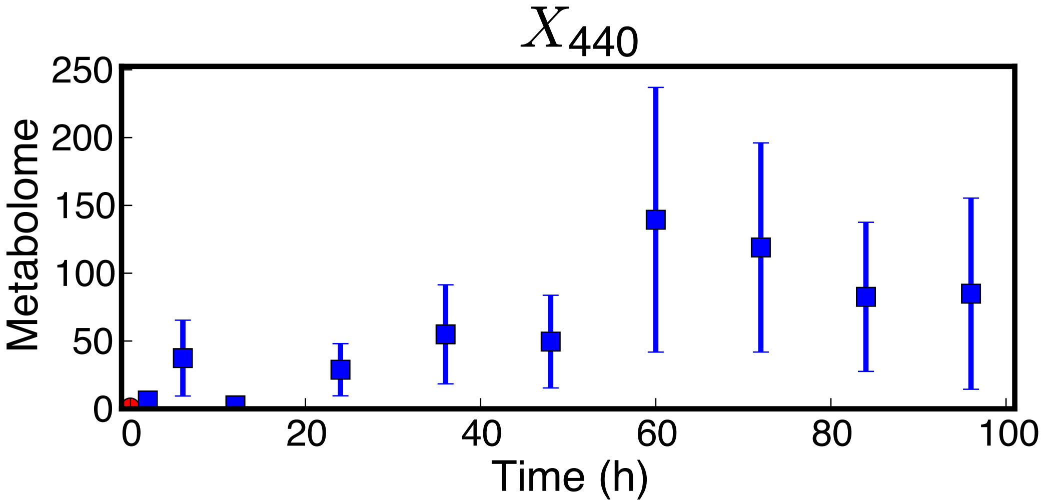

X_{440}
Metabolome
250
200
150
100
50
0
0
20
40
60
80
100
Time (h)

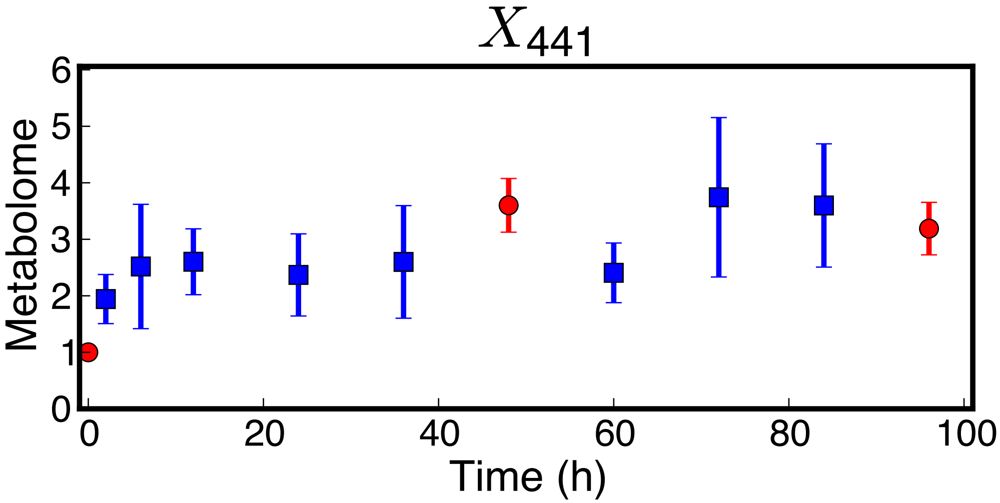

X_{441}
Metabolome
Time (h)
0
1
2
3
4
5
6
0
20
40
60
80
100

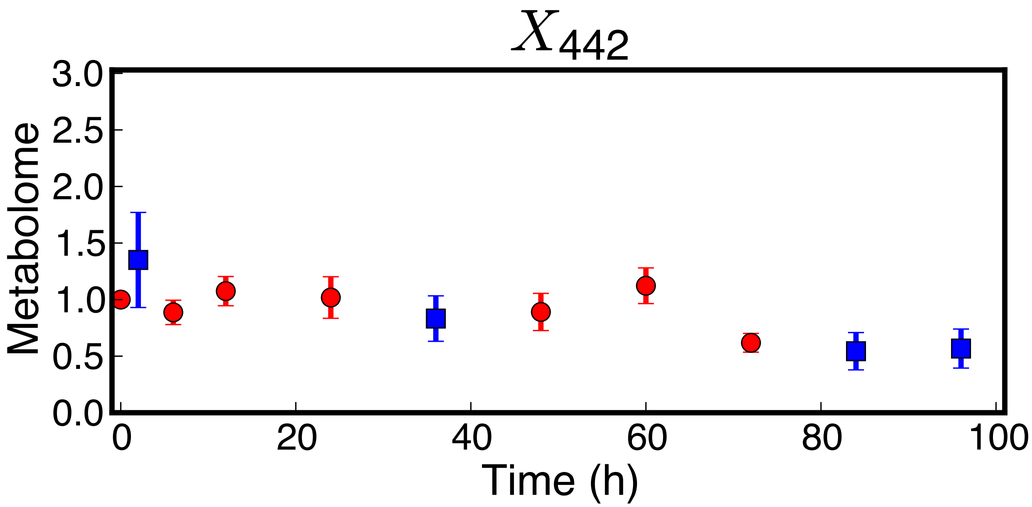

X_{442}
3.0
2.5
2.0
1.5
1.0
0.5
0.0
Metabolome
0
20
40
60
80
100
Time (h)

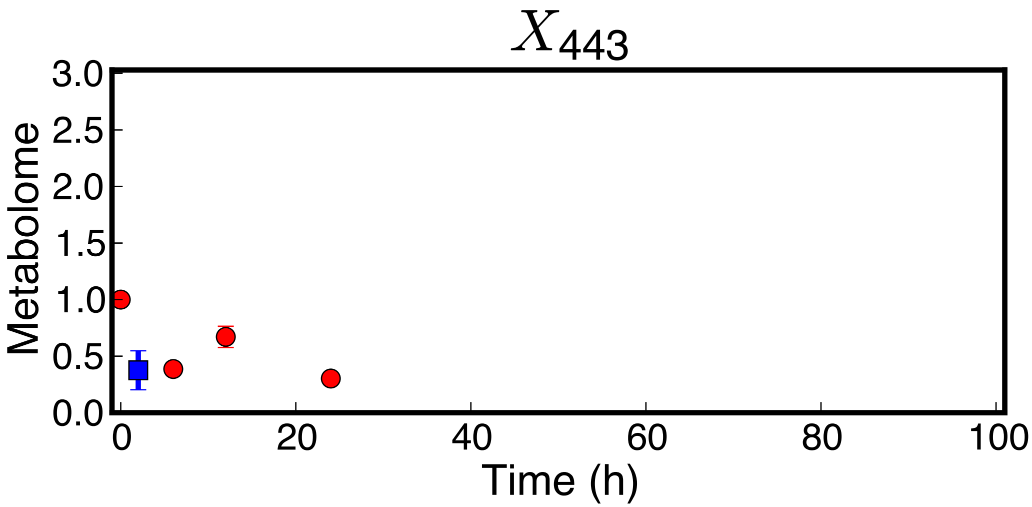

X_{443}
Metabolome
3.0
2.5
2.0
1.5
1.0
0.5
0.0
0
20
40
60
80
100
Time (h)

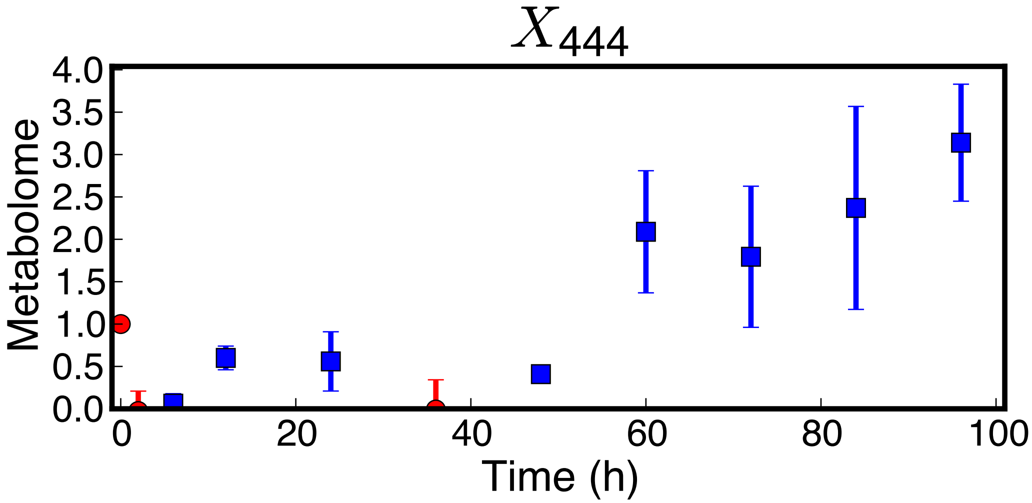

X_{444}
4.0
3.5
3.0
2.5
2.0
1.5
1.0
0.5
0.0
Metabolome
0
20
40
60
80
100
Time (h)

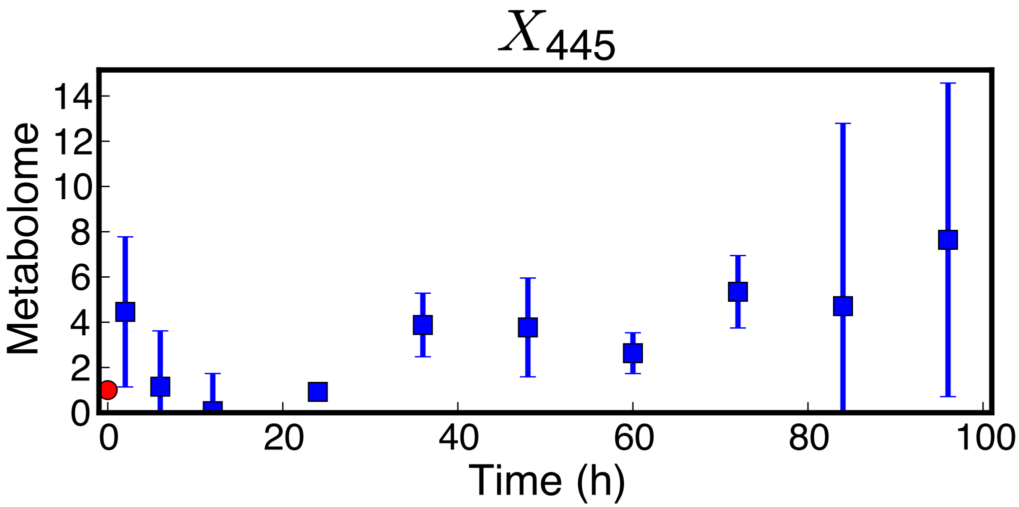

X445
Metabolome
0
2
4
6
8
10
12
14
0
20
40
60
80
100
Time (h)

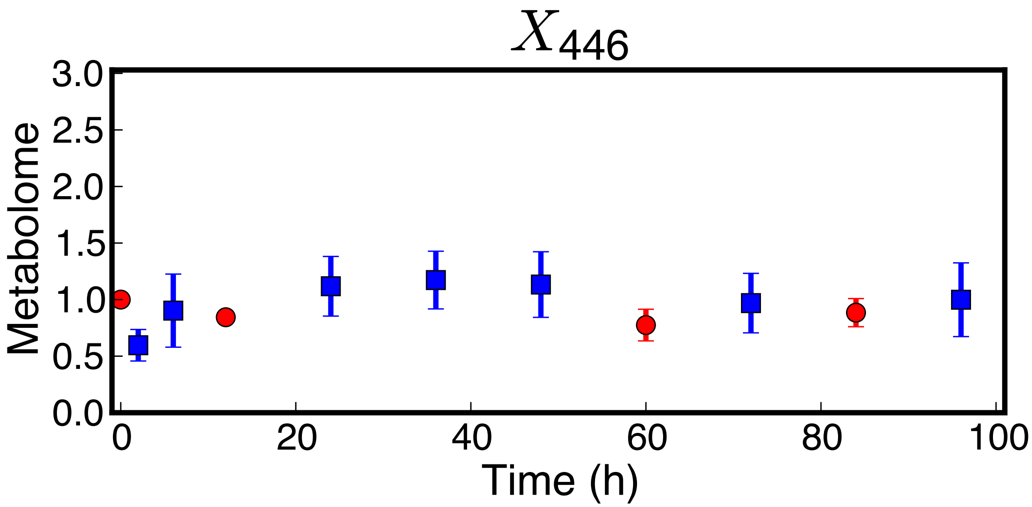

X_{446}
Metabolome
3.0
2.5
2.0
1.5
1.0
0.5
0.0
0
20
40
60
80
100
Time (h)

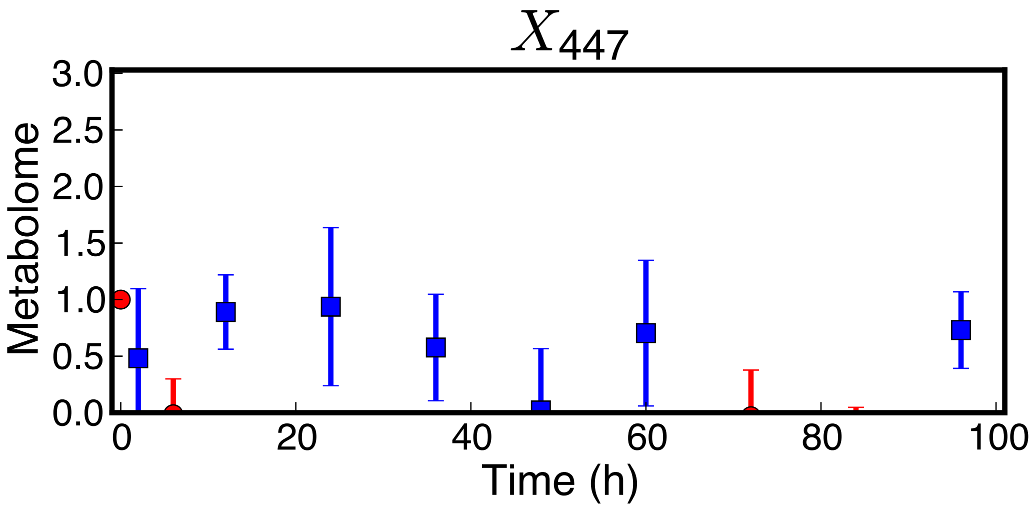

X_{447}
Metabolome
3.0
2.5
2.0
1.5
1.0
0.5
0.0
0
20
40
60
80
100
Time (h)

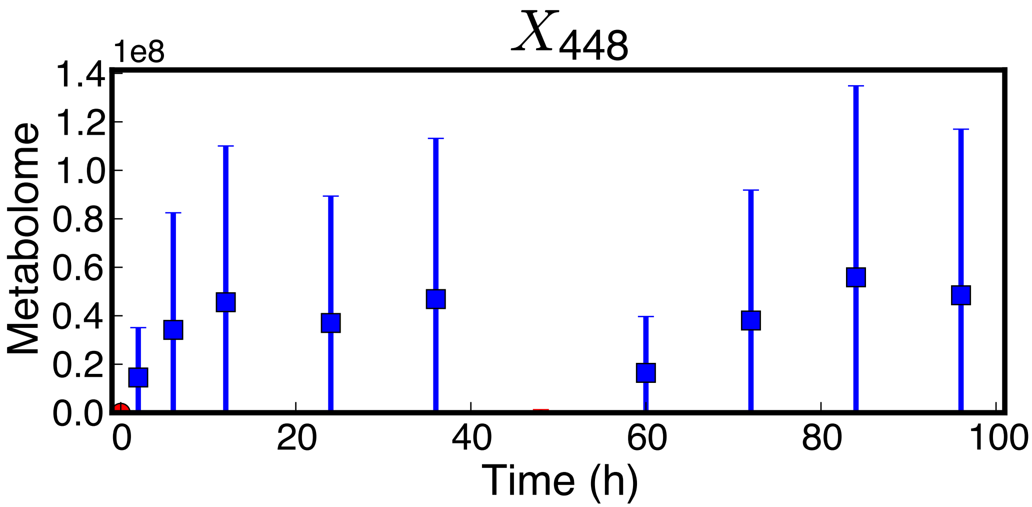

X_{448}
1e8
1.4
1.2
1.0
0.8
0.6
0.4
0.2
0.0
Metabolome
0
20
40
60
80
100
Time (h)

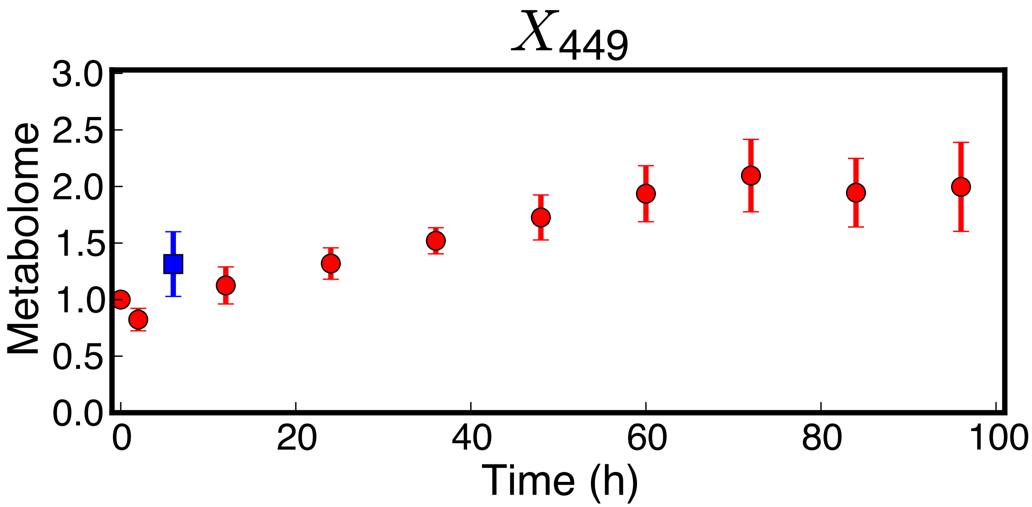

X_{449}
Metabolome
3.0
2.5
2.0
1.5
1.0
0.5
0.0
0
20
40
60
80
100
Time (h)

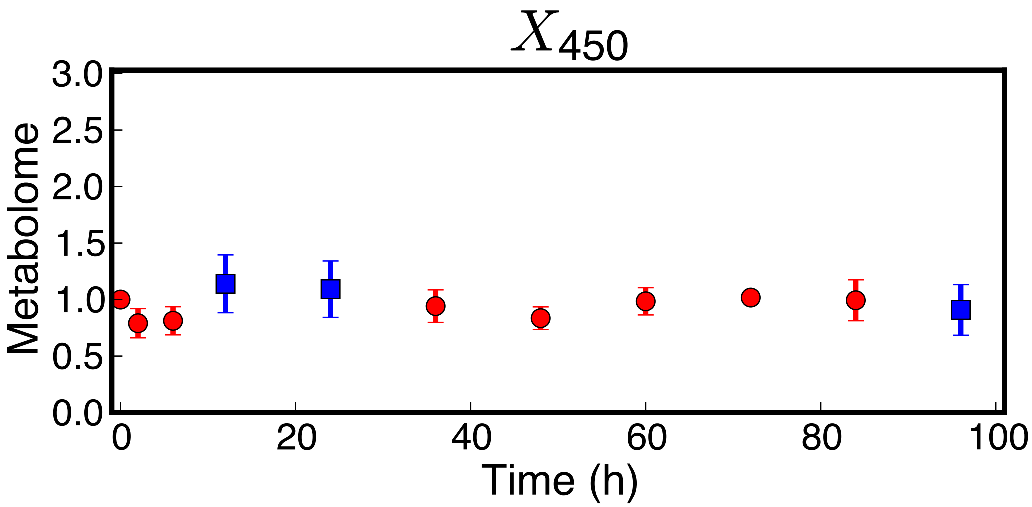
X_{450}
3.0
2.5
2.0
1.5
1.0
0.5
0.0
Metabolome
0
20
40
60
80
100
Time (h)

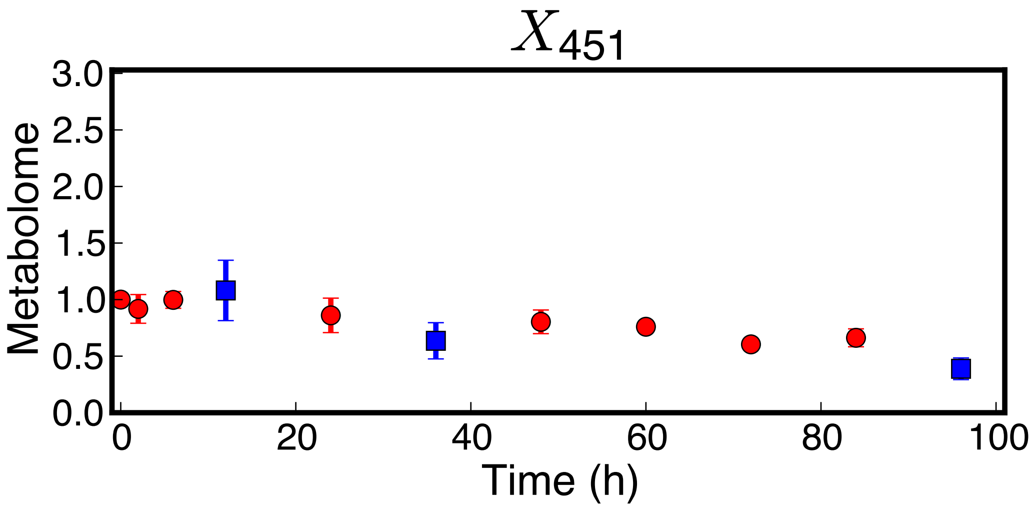
X_{451}
Metabolome
3.0
2.5
2.0
1.5
1.0
0.5
0.0
0
20
40
60
80
100
Time (h)

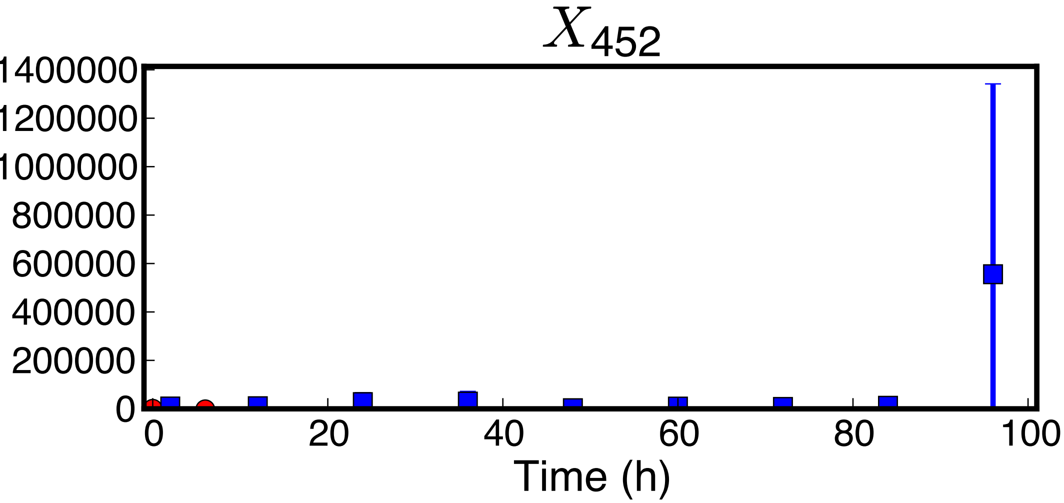

X_{452}
1400000
1200000
1000000
800000
600000
400000
200000
0
0
20
40
60
80
100
Time (h)

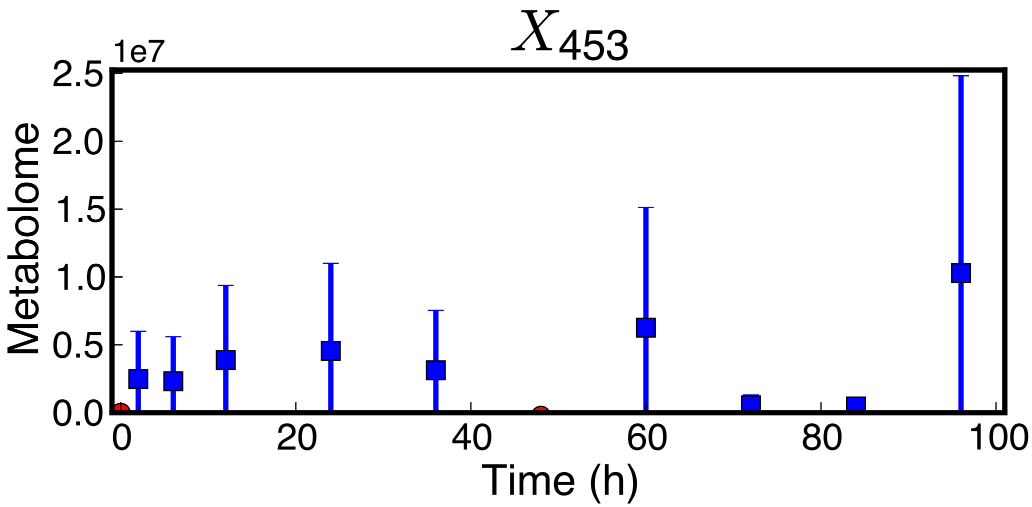

X_{453}
1e7
2.5
2.0
1.5
1.0
0.5
0.0
Metabolome
0
20
40
60
80
100
Time (h)

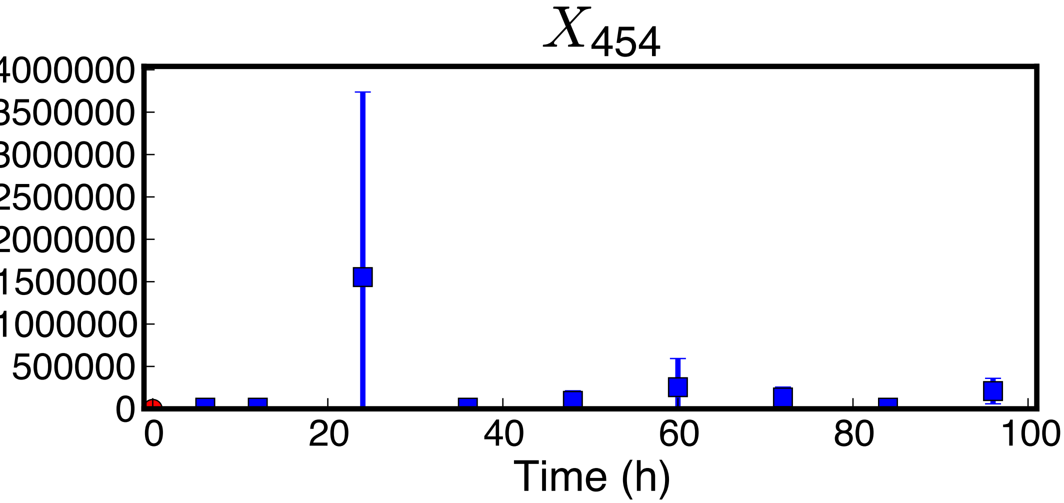

X_{454}
4000000
3500000
3000000
2500000
2000000
1500000
1000000
500000
0
0
20
40
60
80
100
Time (h)

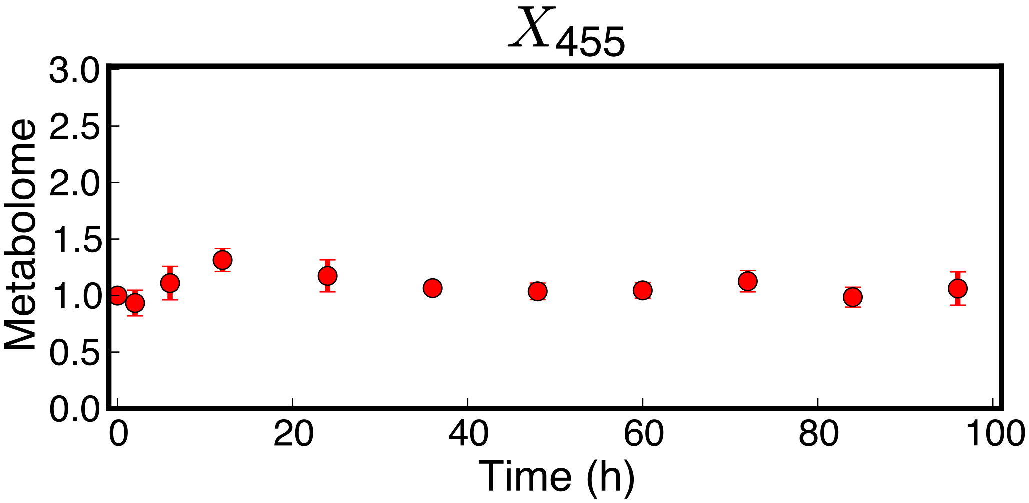

X_{455}
3.0
2.5
2.0
1.5
1.0
0.5
0.0
Metabolome
0
20
40
60
80
100
Time (h)

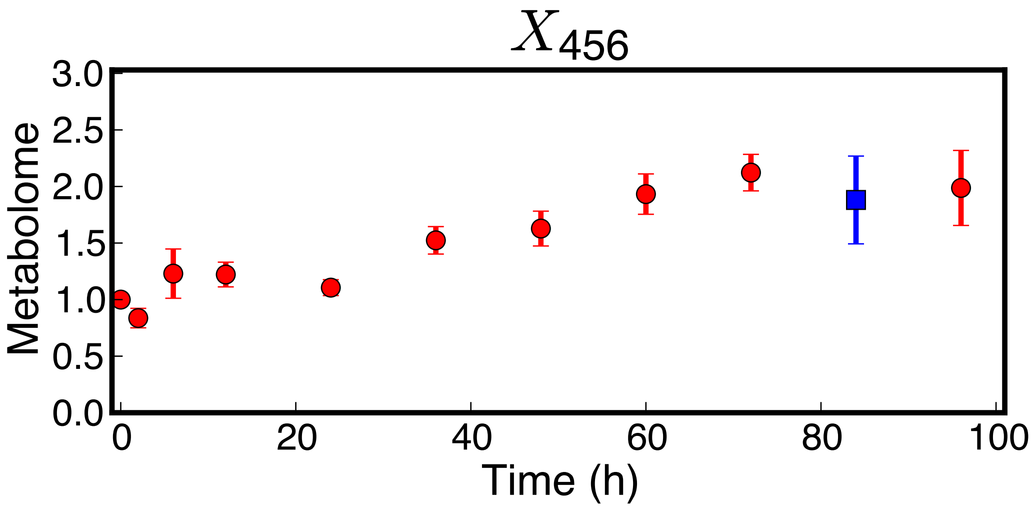

X_{456}
Metabolome
Time (h)
0.0
0.5
1.0
1.5
2.0
2.5
3.0
0
20
40
60
80
100

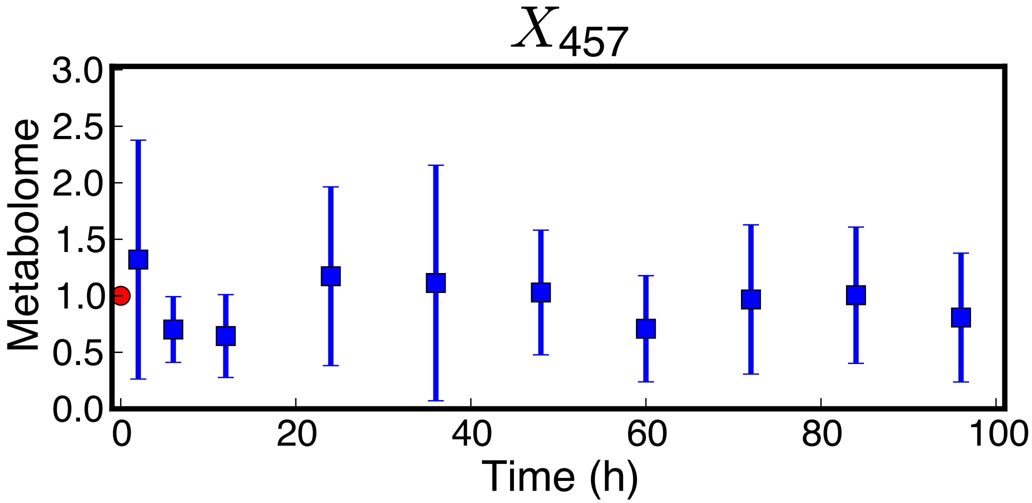

X_{457}
Metabolome
3.0
2.5
2.0
1.5
1.0
0.5
0.0
0
20
40
60
80
100
Time (h)

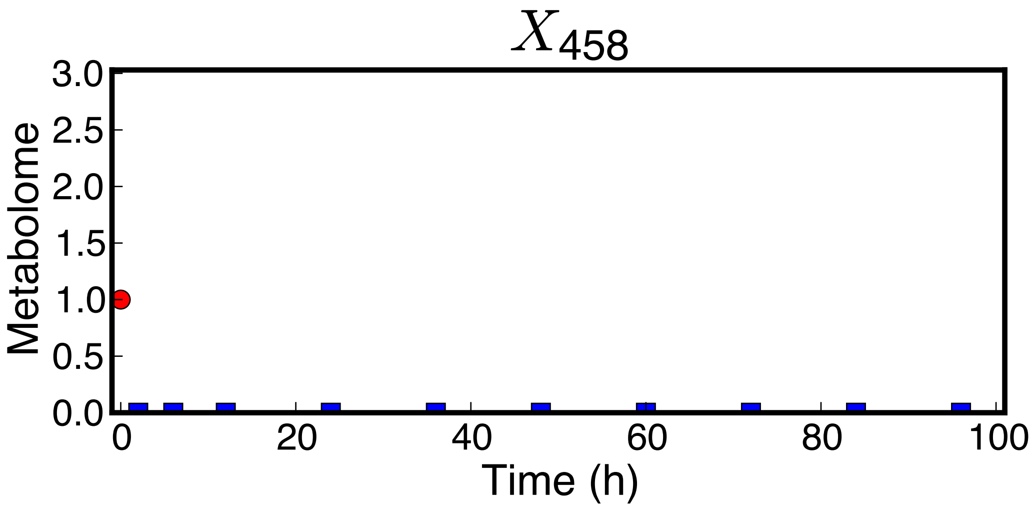
X_{458}
Metabolome
3.0
2.5
2.0
1.5
1.0
0.5
0.0
0
20
40
60
80
100
Time (h)

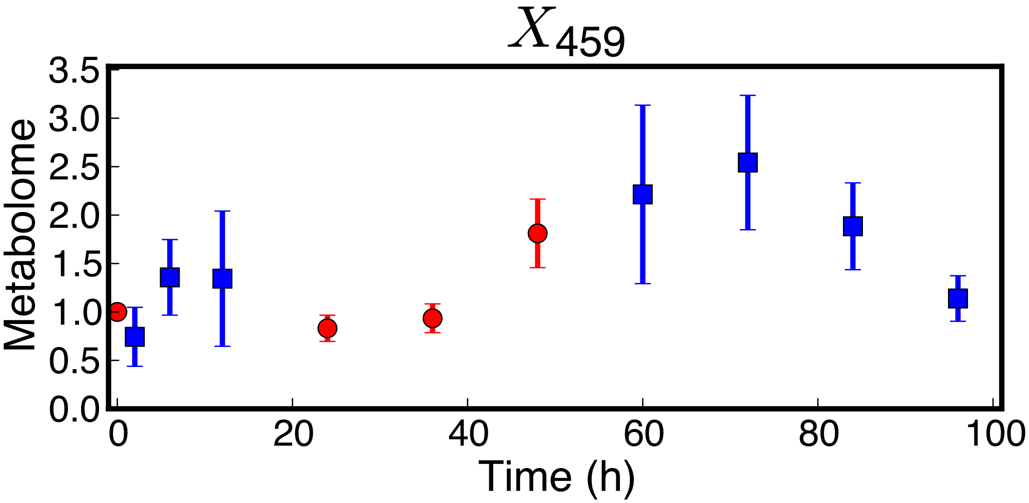
X_{459}
Metabolome
0.0
0.5
1.0
1.5
2.0
2.5
3.0
3.5
0
20
40
60
80
100
Time (h)

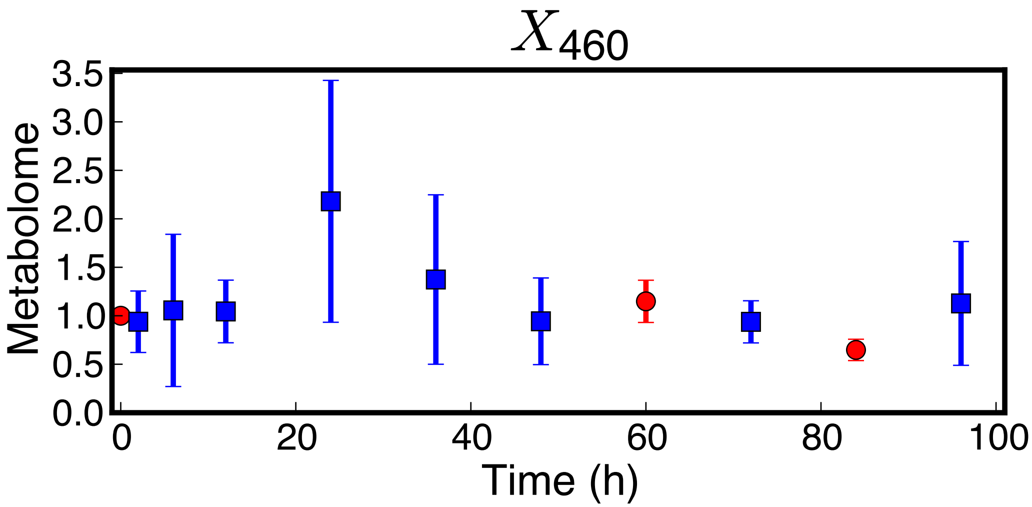

X_{460}
Metabolome
0.0
0.5
1.0
1.5
2.0
2.5
3.0
3.5
0
20
40
60
80
100
Time (h)

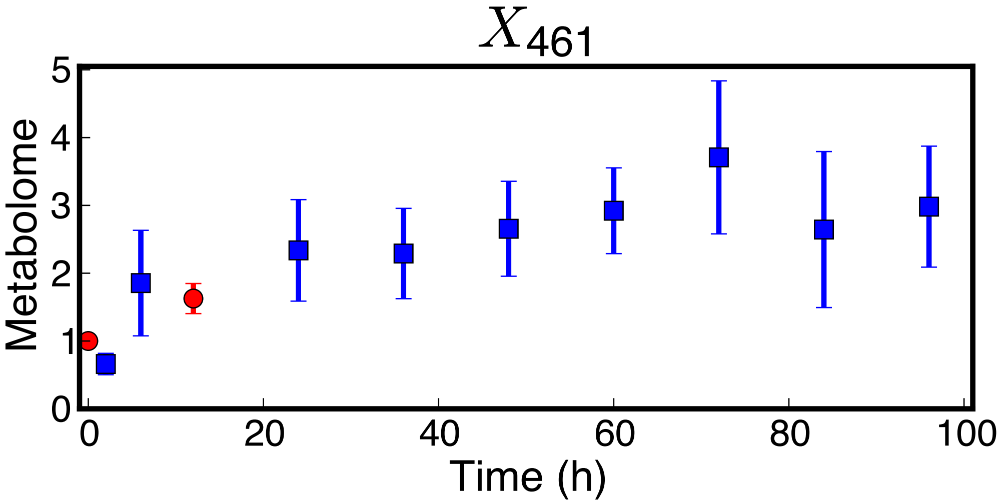
X_{461}
Metabolome
Time (h)
0
1
2
3
4
5
0
20
40
60
80
100

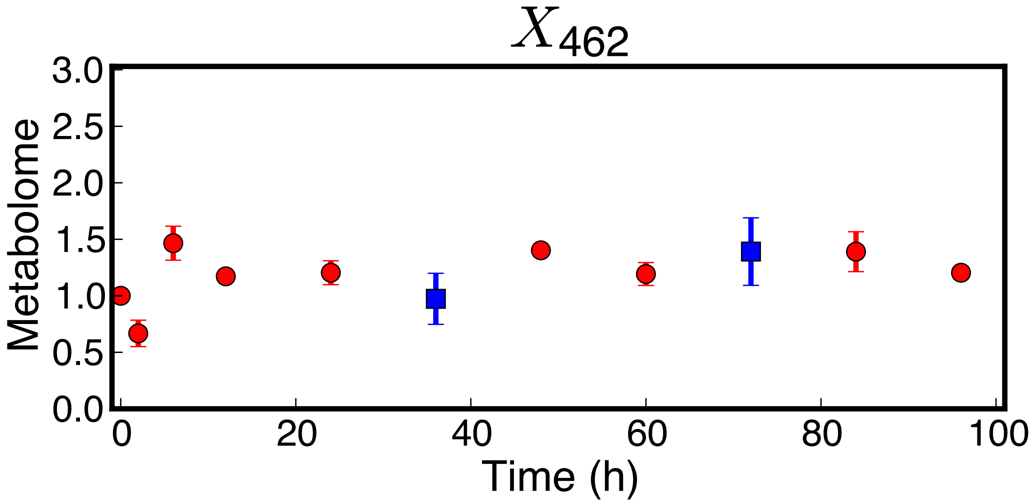

X_{462}
Metabolome
3.0
2.5
2.0
1.5
1.0
0.5
0.0
0
20
40
60
80
100
Time (h)

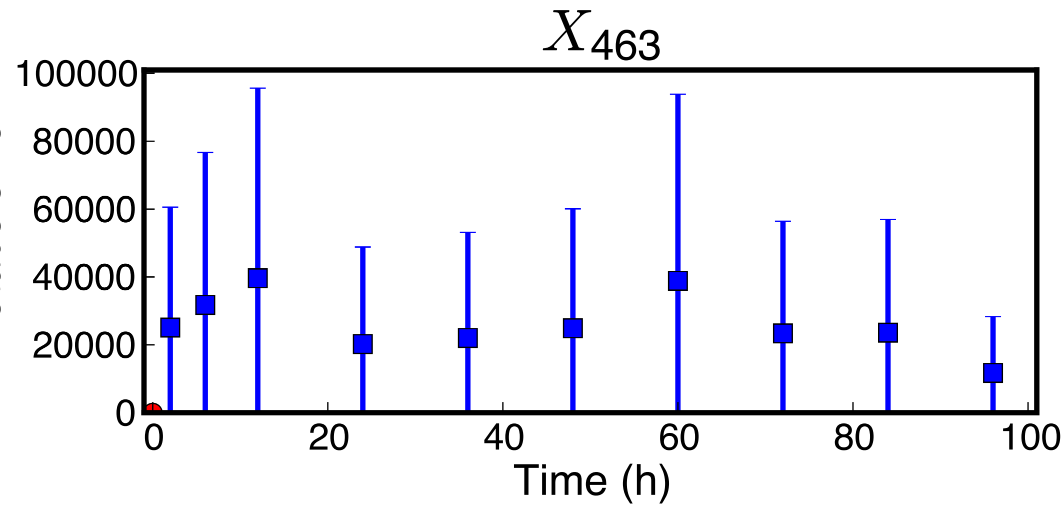
X_{463}
100000
80000
60000
40000
20000
0
0
20
40
60
80
100
Time (h)

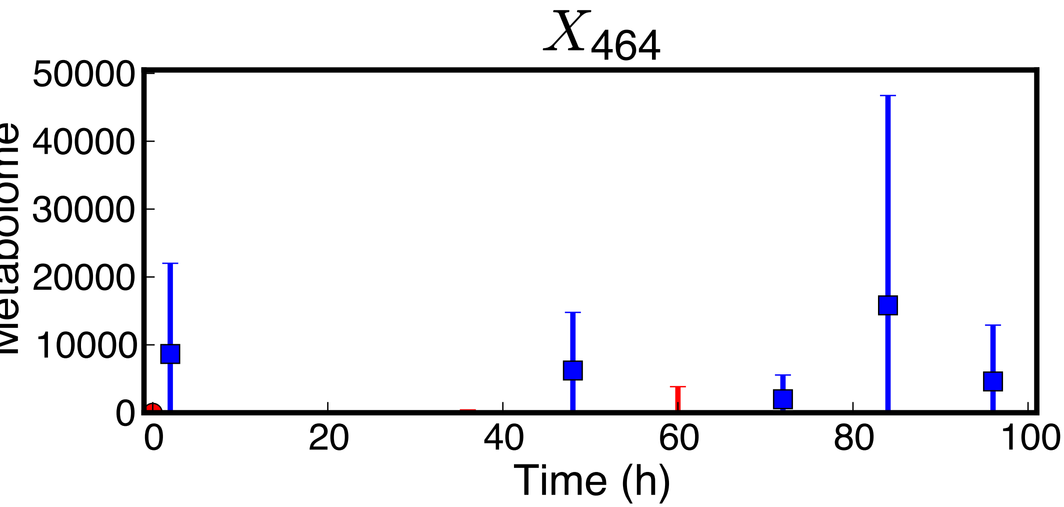

X_{464}
Metabolome
50000
40000
30000
20000
10000
0
0
20
40
60
80
100
Time (h)

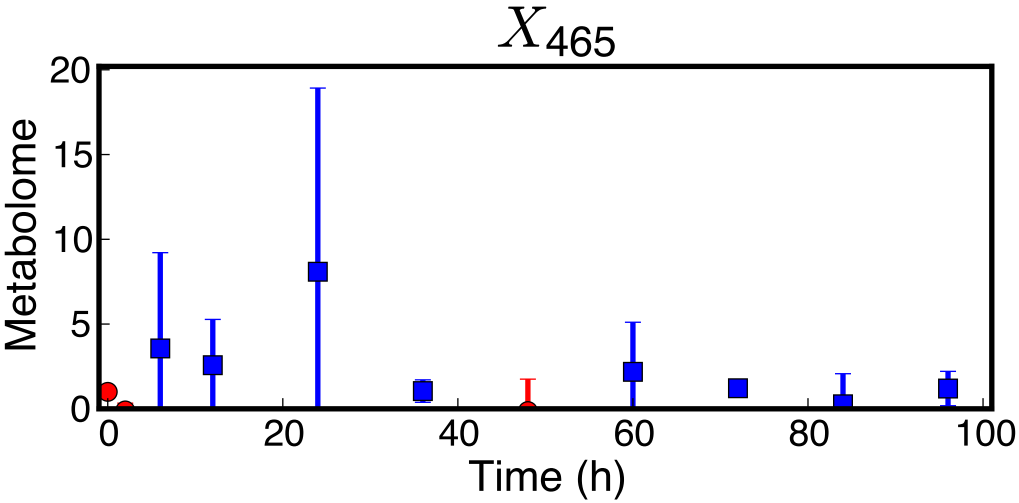

X_{465}
Metabolome
20
15
10
5
0
0
20
40
60
80
100
Time (h)

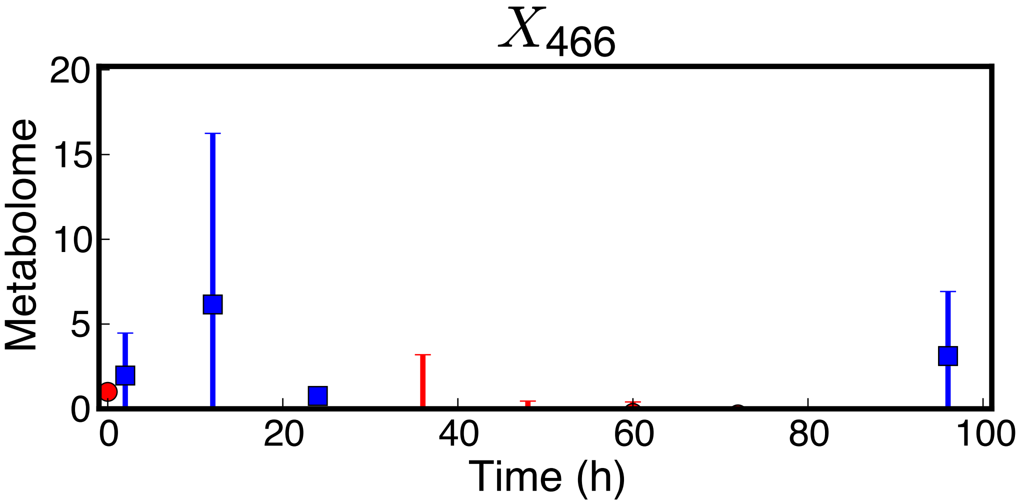

X_{466}
Metabolome
Time (h)
0
5
10
15
20
0
20
40
60
80
100

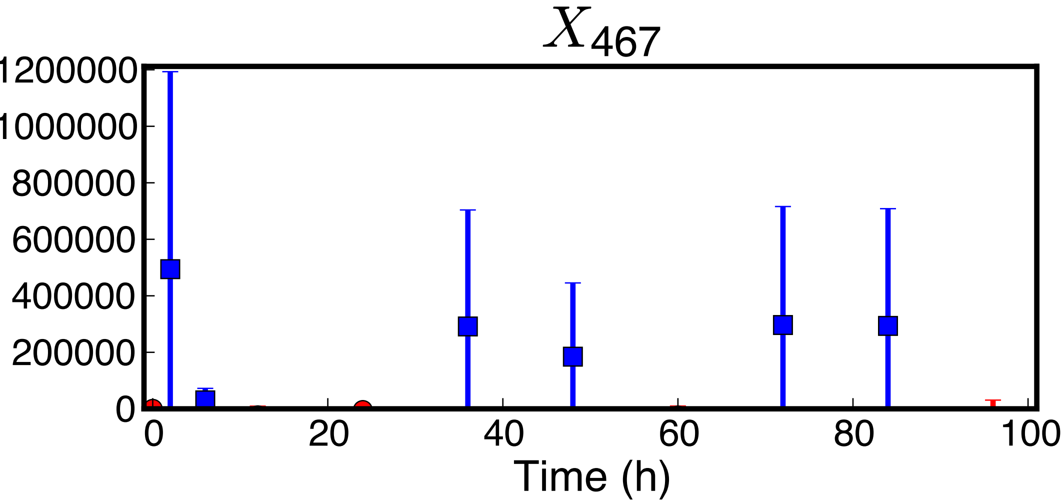

X_{467}
1200000
1000000
800000
600000
400000
200000
0
0
20
40
60
80
100
Time (h)

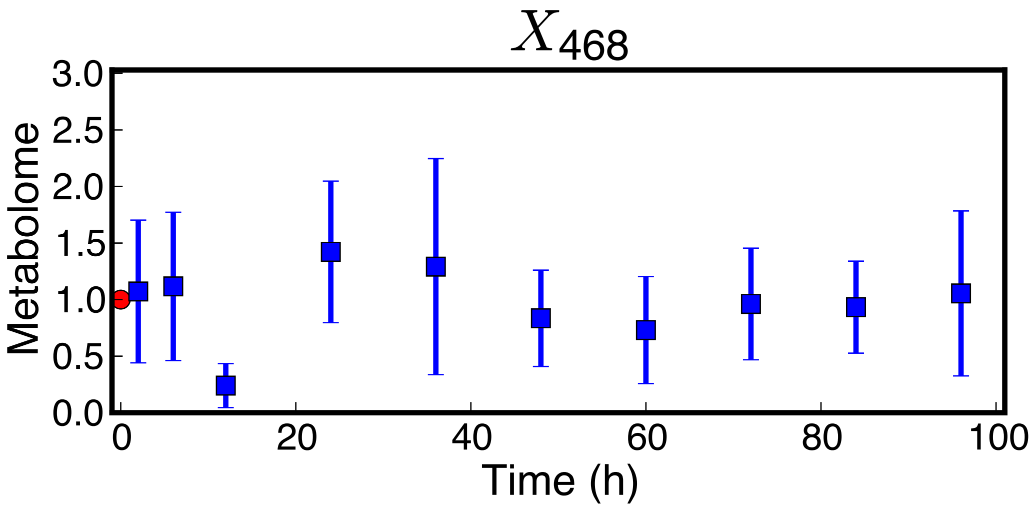
X_{468}
Metabolome
3.0
2.5
2.0
1.5
1.0
0.5
0.0
0
20
40
60
80
100
Time (h)

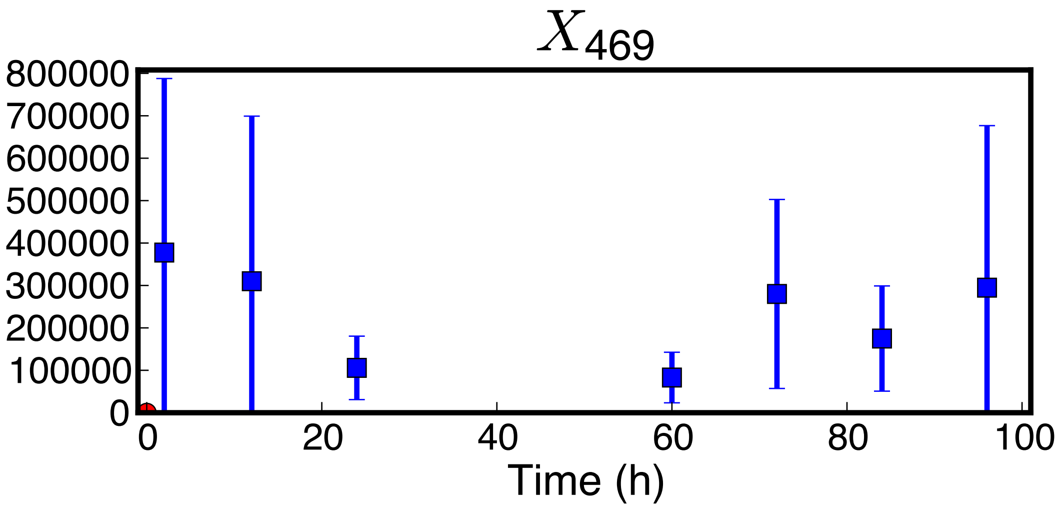

X_{469}
800000
700000
600000
500000
400000
300000
200000
100000
0
0
20
40
60
80
100
Time (h)

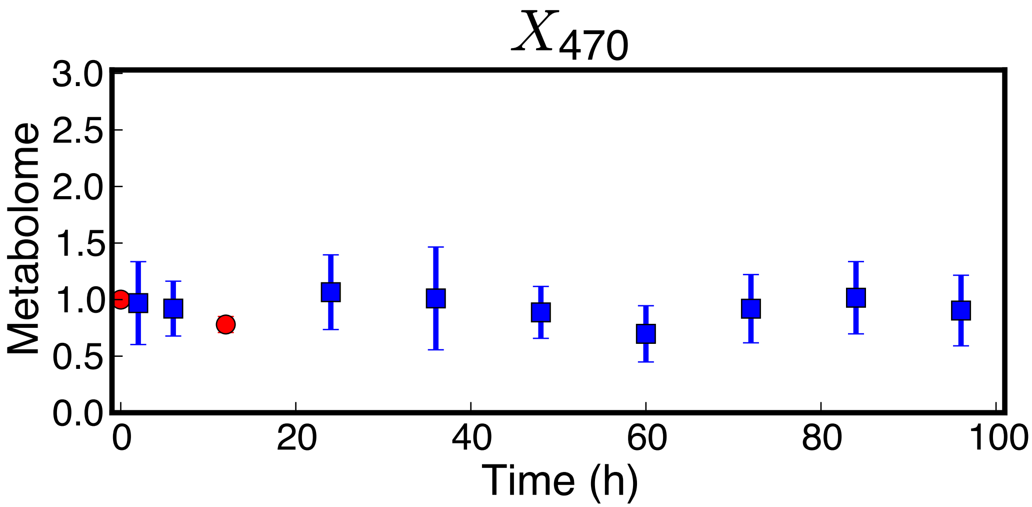

X_{470}
Metabolome
3.0
2.5
2.0
1.5
1.0
0.5
0.0
0
20
40
60
80
100
Time (h)

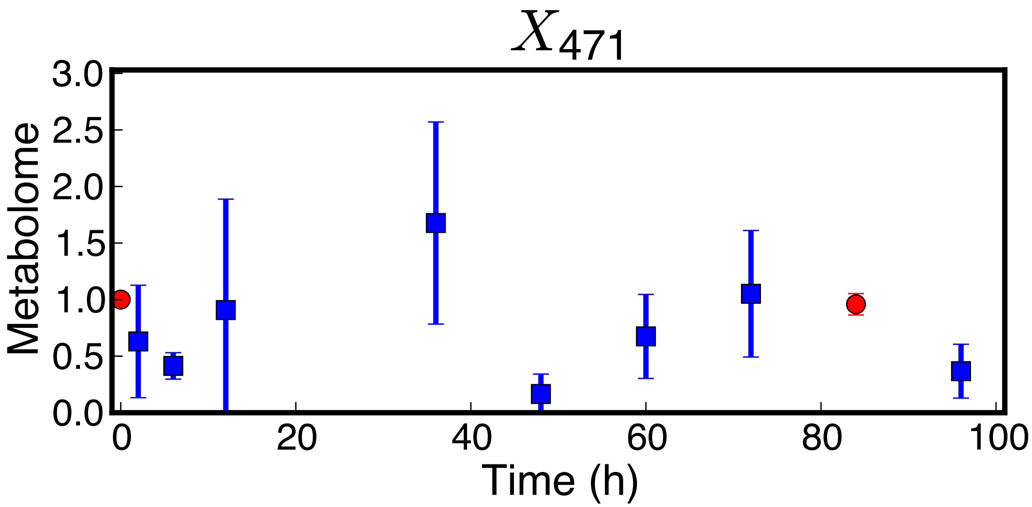

X_{471}
Metabolome
3.0
2.5
2.0
1.5
1.0
0.5
0.0
0
20
40
60
80
100
Time (h)

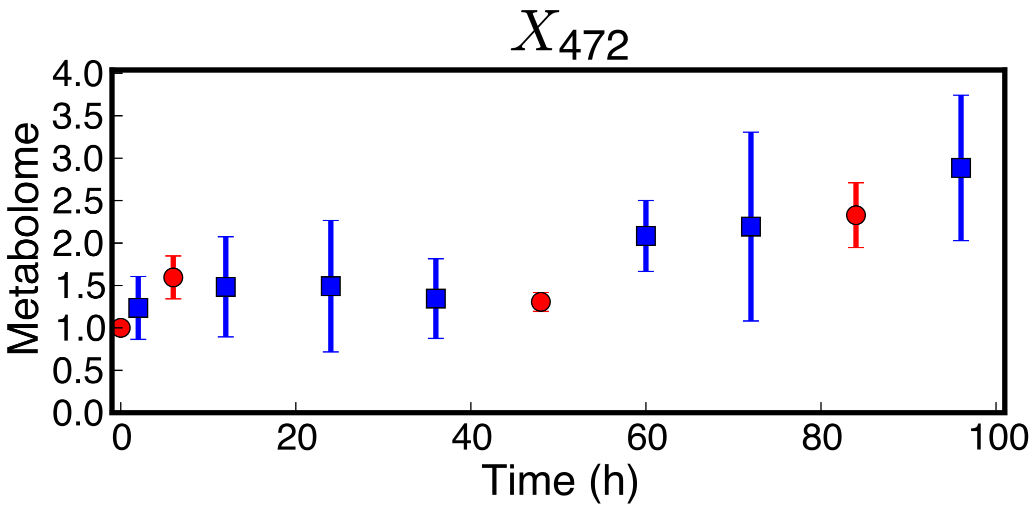

X_{472}
Metabolome
4.0
3.5
3.0
2.5
2.0
1.5
1.0
0.5
0.0
0
20
40
60
80
100
Time (h)

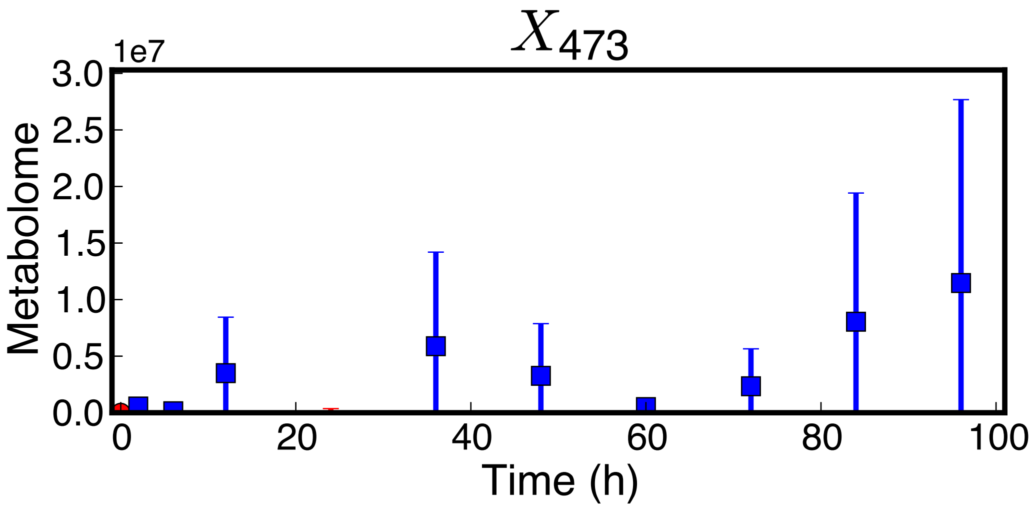

X_{473}
1e7
3.0
2.5
2.0
1.5
1.0
0.5
0.0
Metabolome
0
20
40
60
80
100
Time (h)

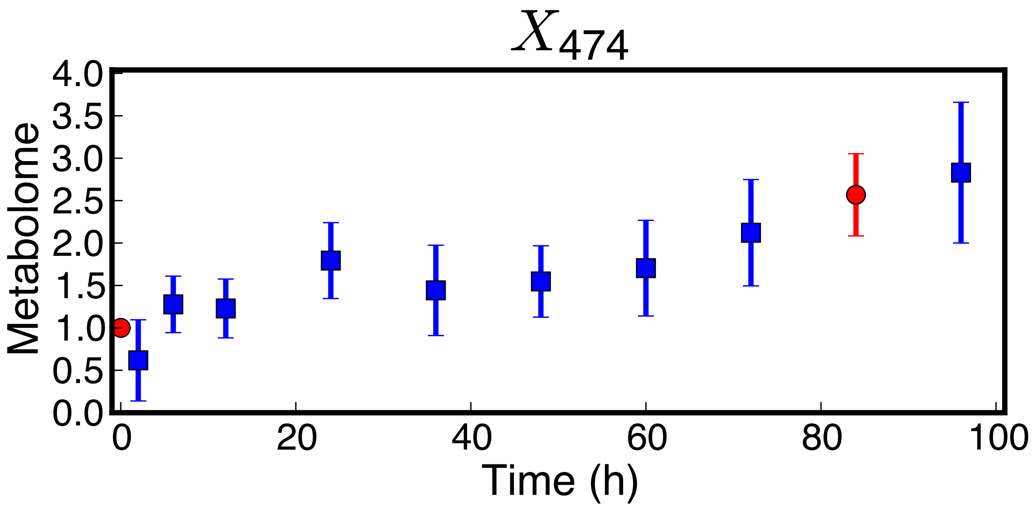

X_{474}
Metabolome
4.0
3.5
3.0
2.5
2.0
1.5
1.0
0.5
0.0
0
20
40
60
80
100
Time (h)

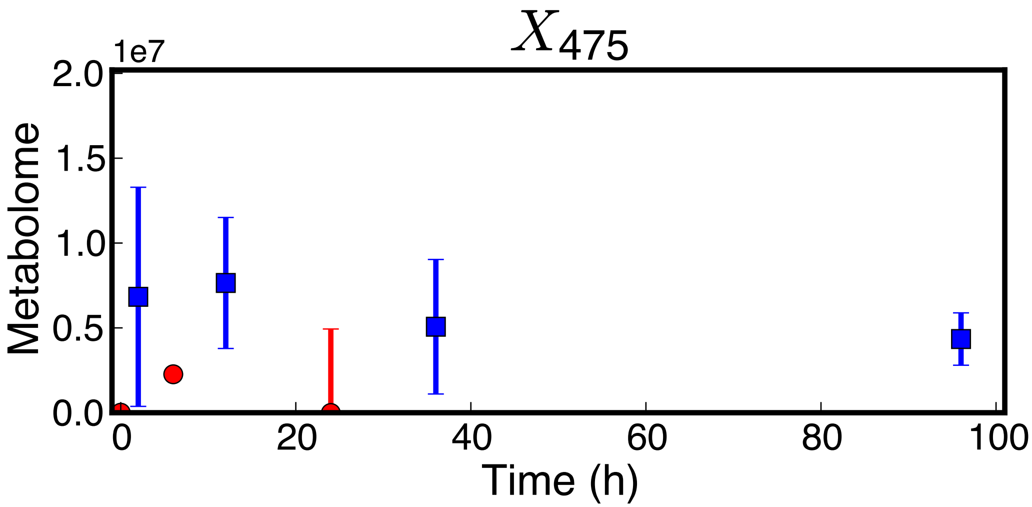

X_{475}
1e7
2.0
1.5
1.0
0.5
0.0
Metabolome
0
20
40
60
80
100
Time (h)

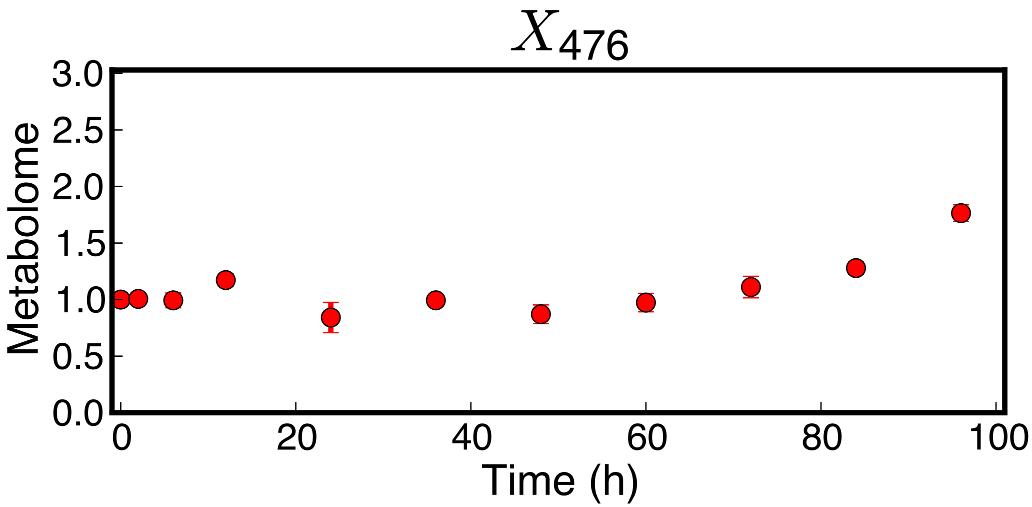

X476
Metabolome
Time (h)
3.0
2.5
2.0
1.5
1.0
0.5
0.0
0
20
40
60
80
100

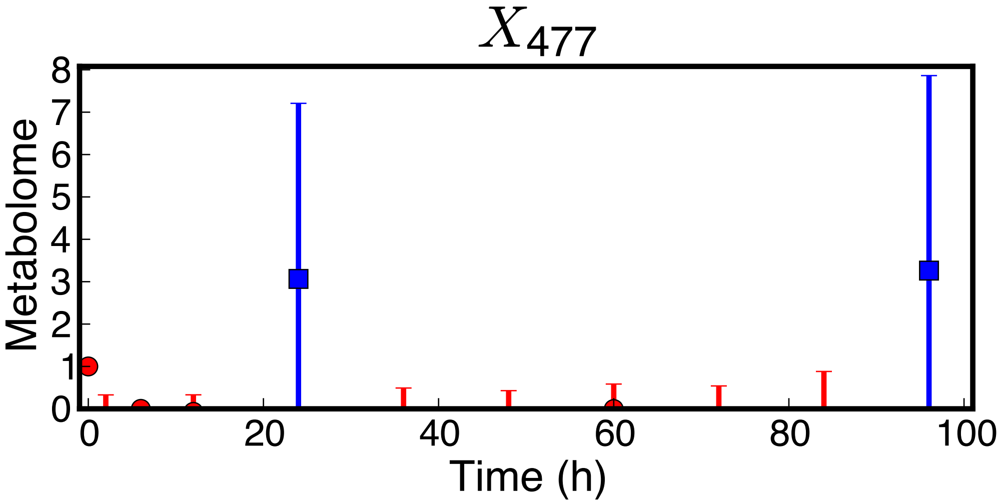

X_{477}
Metabolome
Time (h)
0
1
2
3
4
5
6
7
8
0
20
40
60
80
100

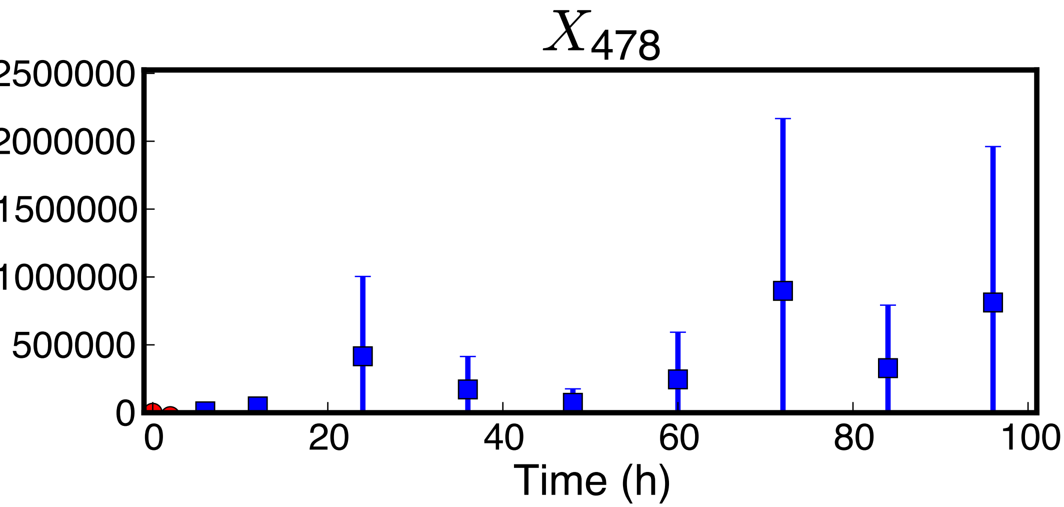

X_{478}
2500000
2000000
1500000
1000000
500000
0
0
20
40
60
80
100
Time (h)

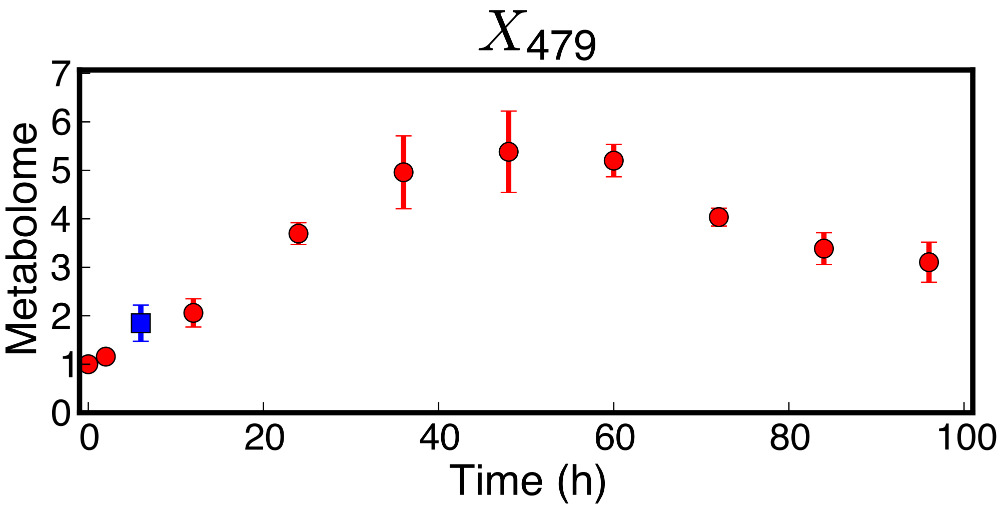

X_{479}
Metabolome
0
1
2
3
4
5
6
7
0
20
40
60
80
100
Time (h)

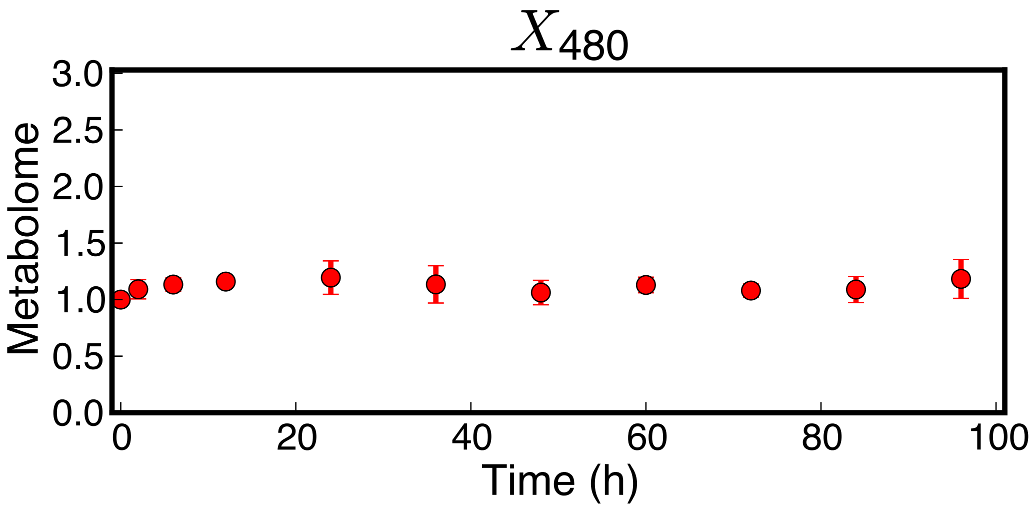
X_{480}
Metabolome
3.0
2.5
2.0
1.5
1.0
0.5
0.0
0
20
40
60
80
100
Time (h)

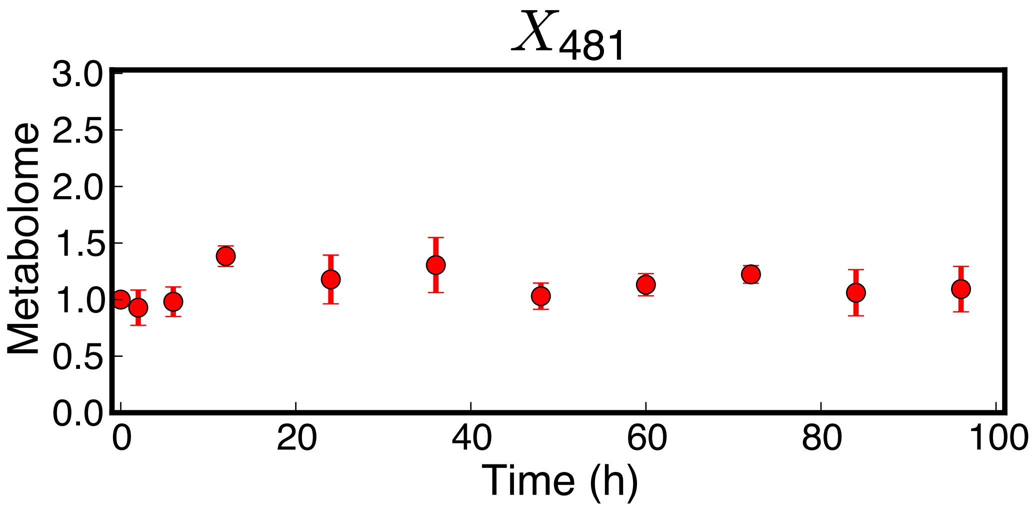
X_{481}
Metabolome
3.0
2.5
2.0
1.5
1.0
0.5
0.0
0
20
40
60
80
100
Time (h)

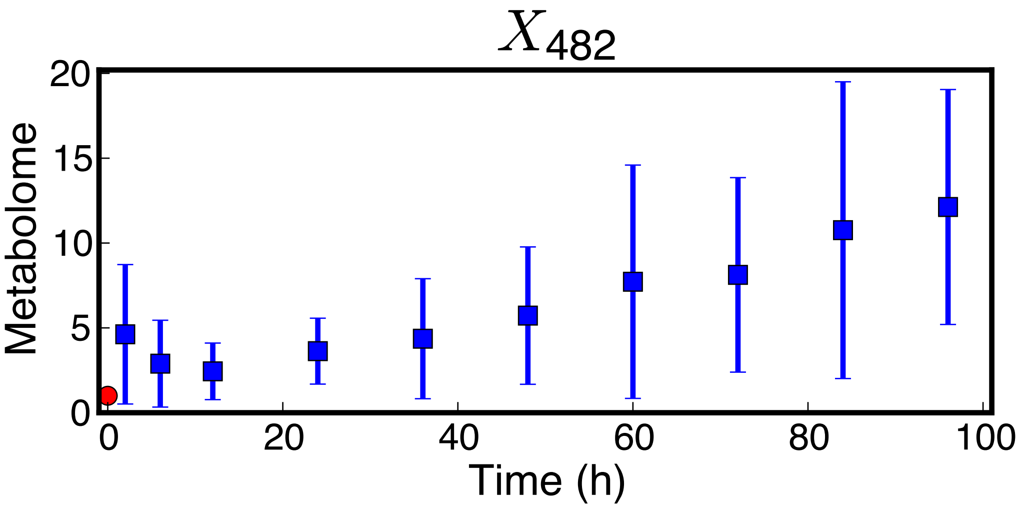

X_{482}
Metabolome
Time (h)
0
5
10
15
20
0
20
40
60
80
100

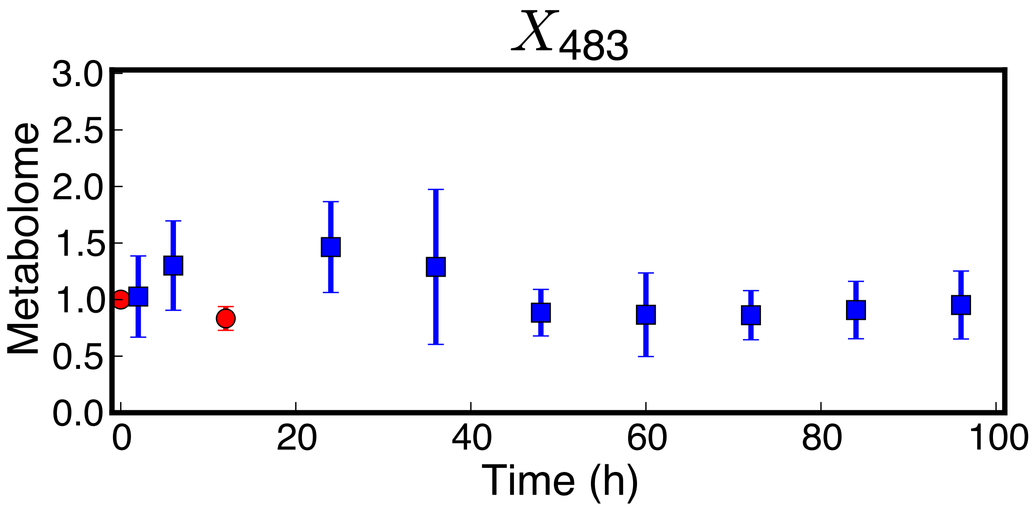
X_{483}
Metabolome
3.0
2.5
2.0
1.5
1.0
0.5
0.0
0
20
40
60
80
100
Time (h)

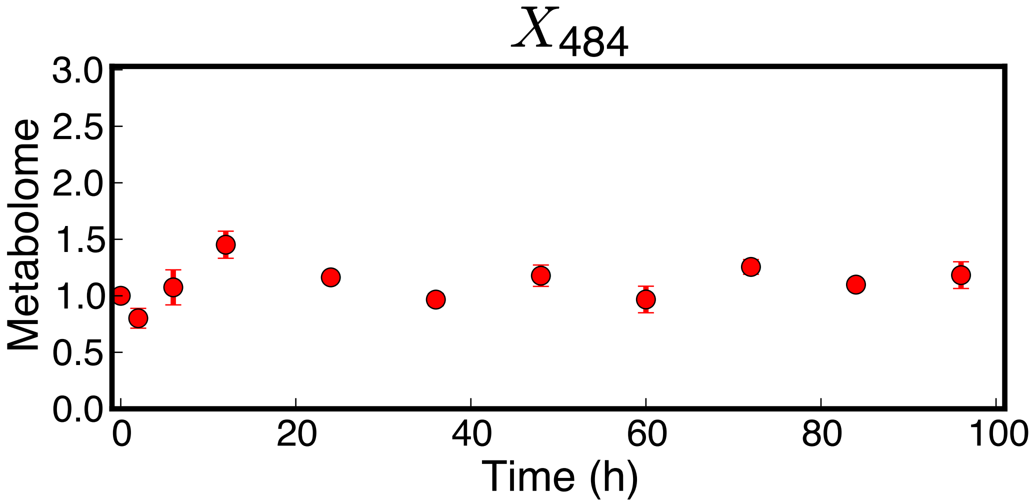

X_{484}
Metabolome
0.0
0.5
1.0
1.5
2.0
2.5
3.0
0
20
40
60
80
100
Time (h)

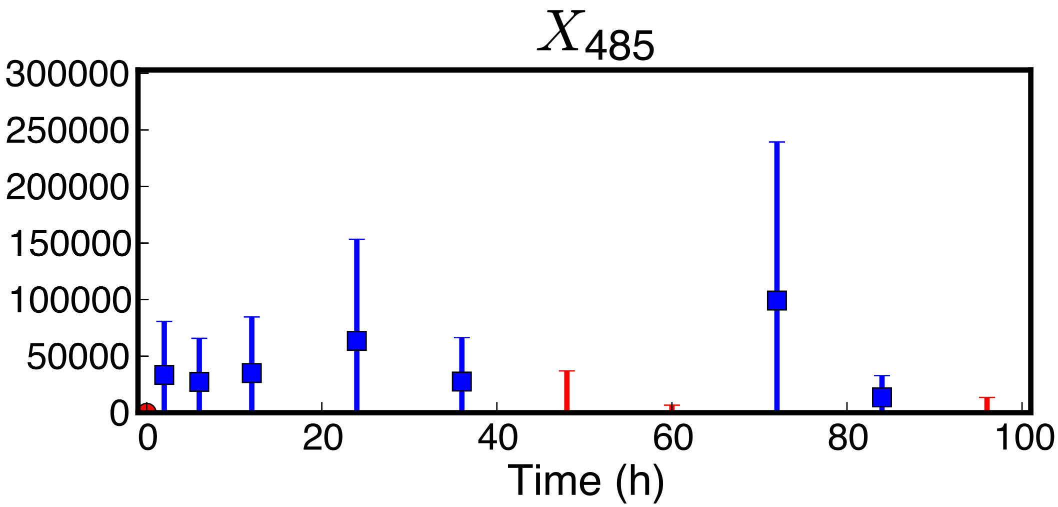

X_{485}
300000
250000
200000
150000
100000
50000
0
0
20
40
60
80
100
Time (h)

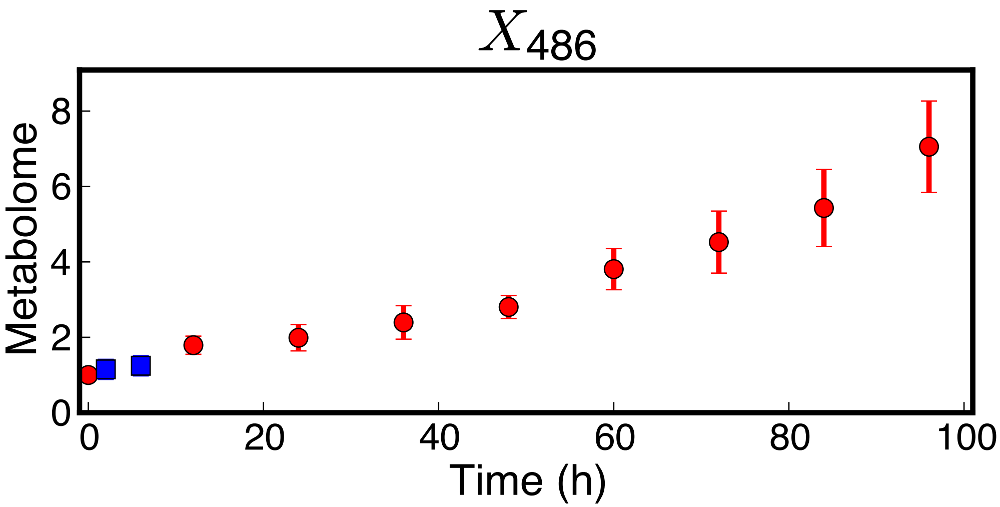

X_{486}
Metabolome
Time (h)
0
2
4
6
8
0
20
40
60
80
100

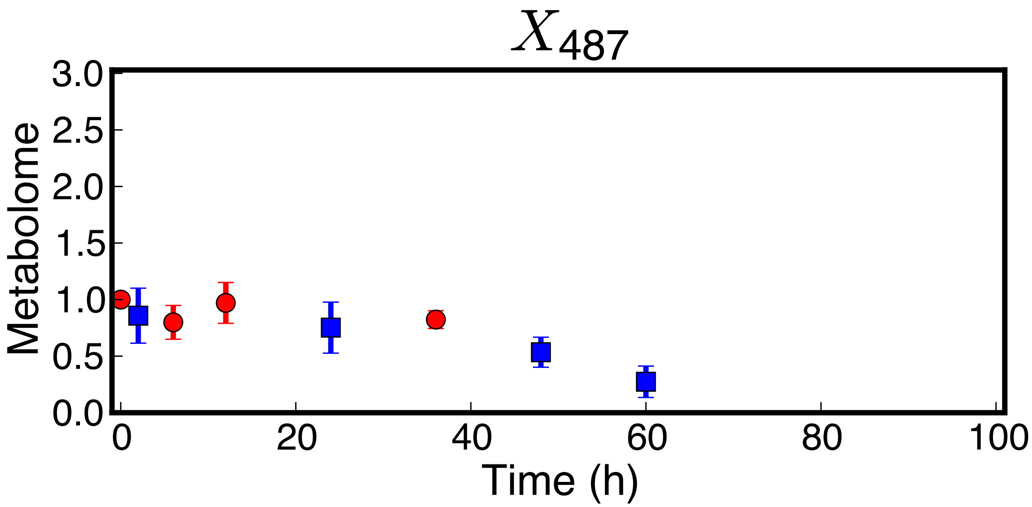
X_{487}
Metabolome
3.0
2.5
2.0
1.5
1.0
0.5
0.0
0
20
40
60
80
100
Time (h)

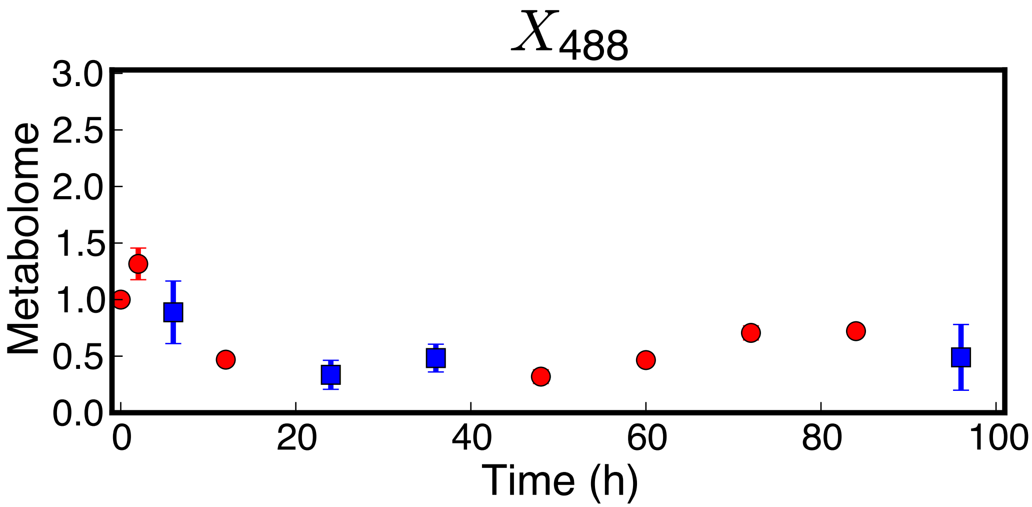

X488
Metabolome
Time (h)
0.0
0.5
1.0
1.5
2.0
2.5
3.0
0
20
40
60
80
100

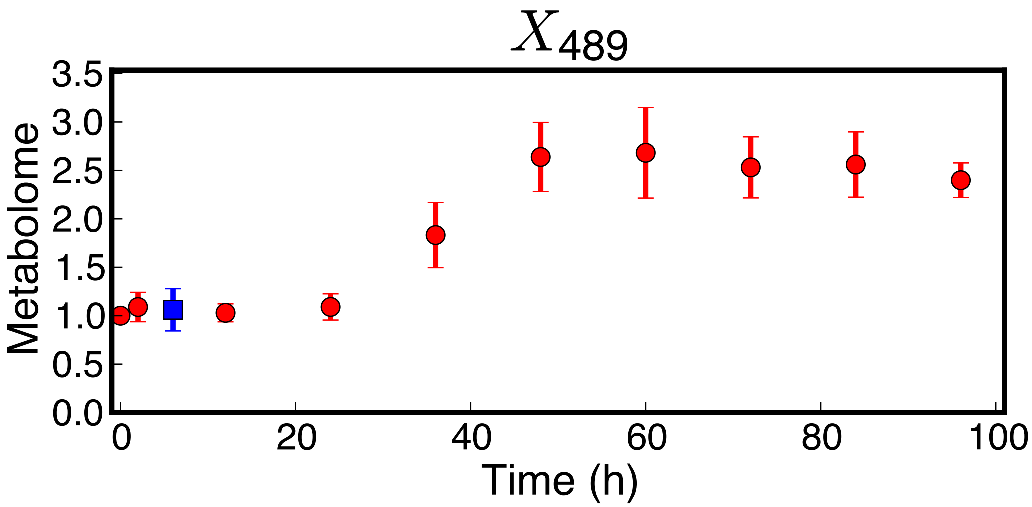

X_{489}
Metabolome
Time (h)
0.0
0.5
1.0
1.5
2.0
2.5
3.0
3.5
0
20
40
60
80
100

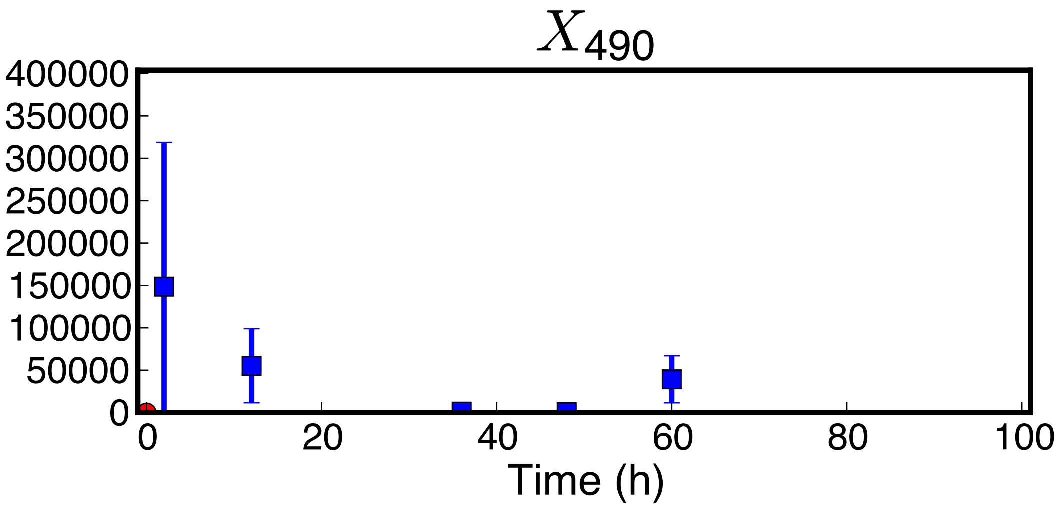

X_{490}
400000
350000
300000
250000
200000
150000
100000
50000
0
0
20
40
60
80
100
Time (h)

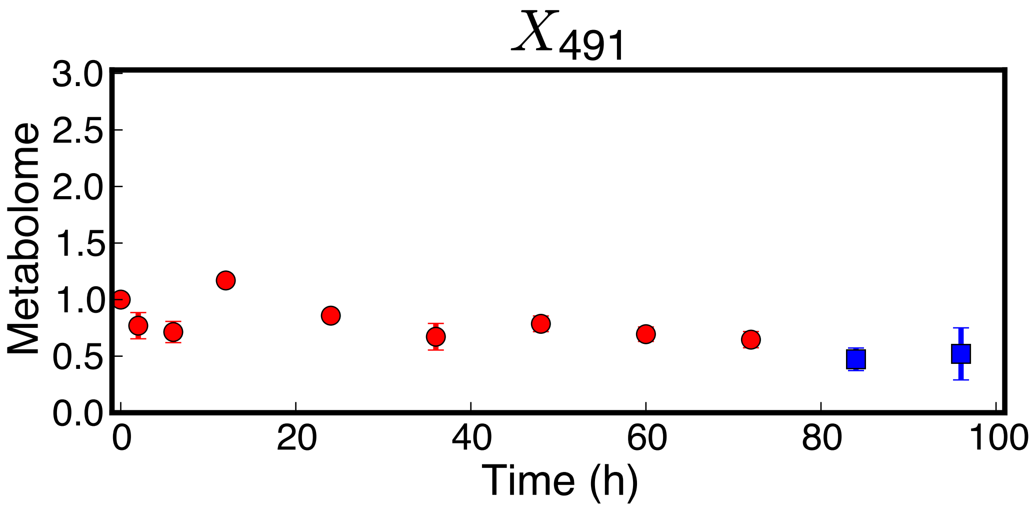

X_{491}
3.0
2.5
2.0
1.5
1.0
0.5
0.0
Metabolome
0
20
40
60
80
100
Time (h)

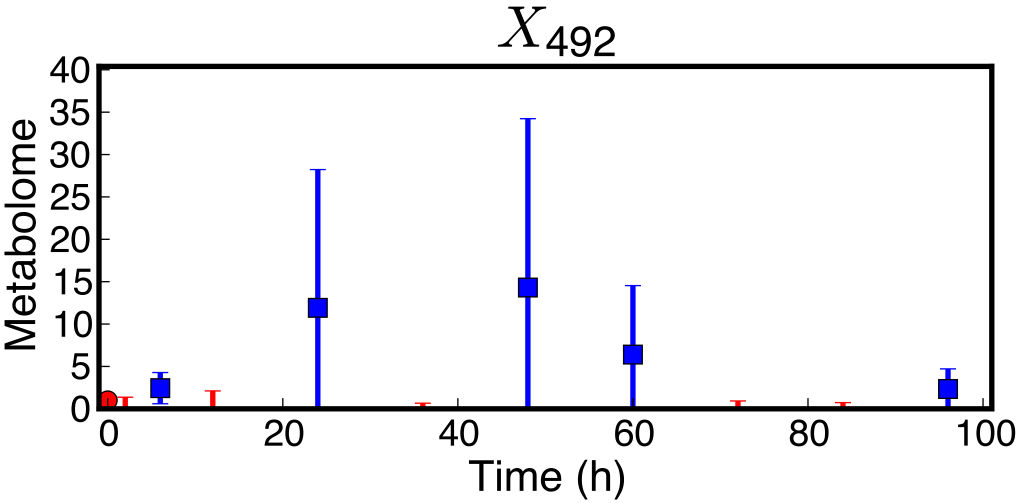

X_{492}
Metabolome
Time (h)
0
5
10
15
20
25
30
35
40
0
20
40
60
80
100

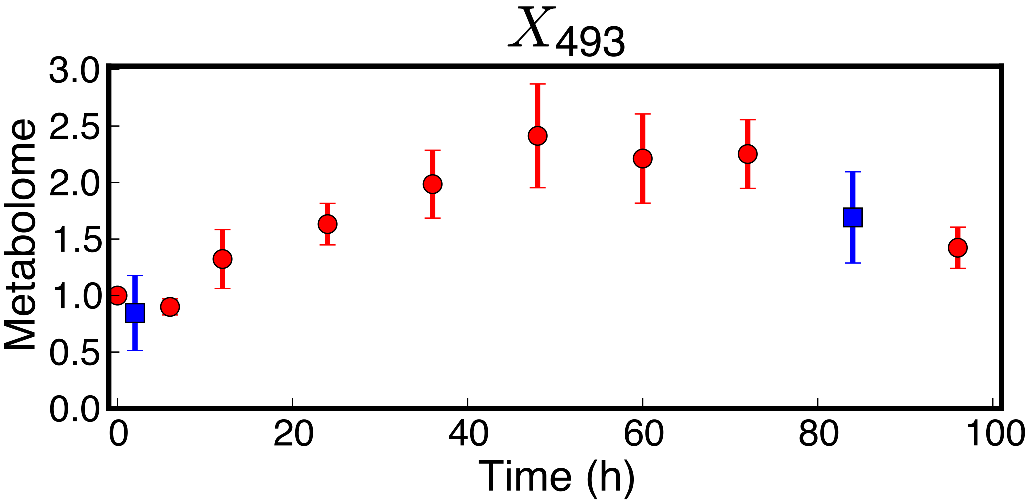

X_{493}
3.0
2.5
2.0
1.5
1.0
0.5
0.0
Metabolome
0
20
40
60
80
100
Time (h)

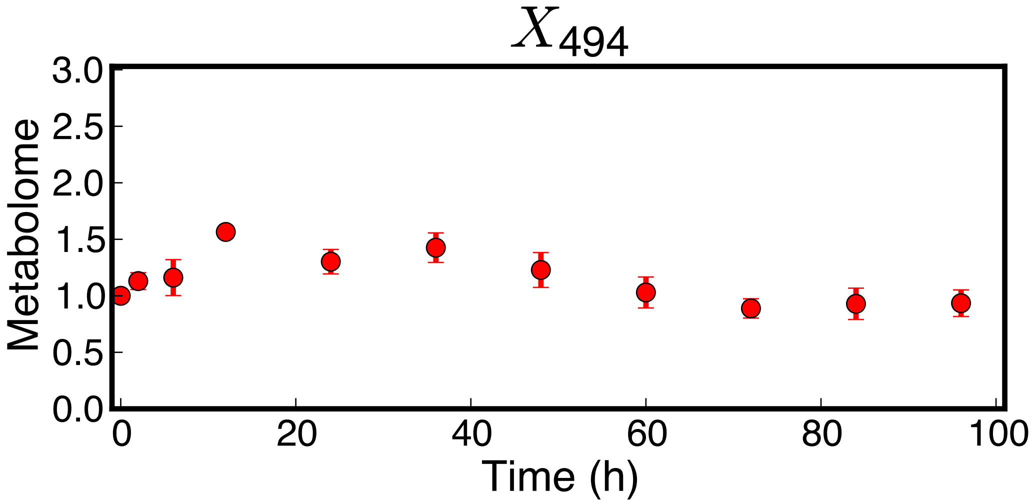

X_{494}
Metabolome
3.0
2.5
2.0
1.5
1.0
0.5
0.0
0
20
40
60
80
100
Time (h)

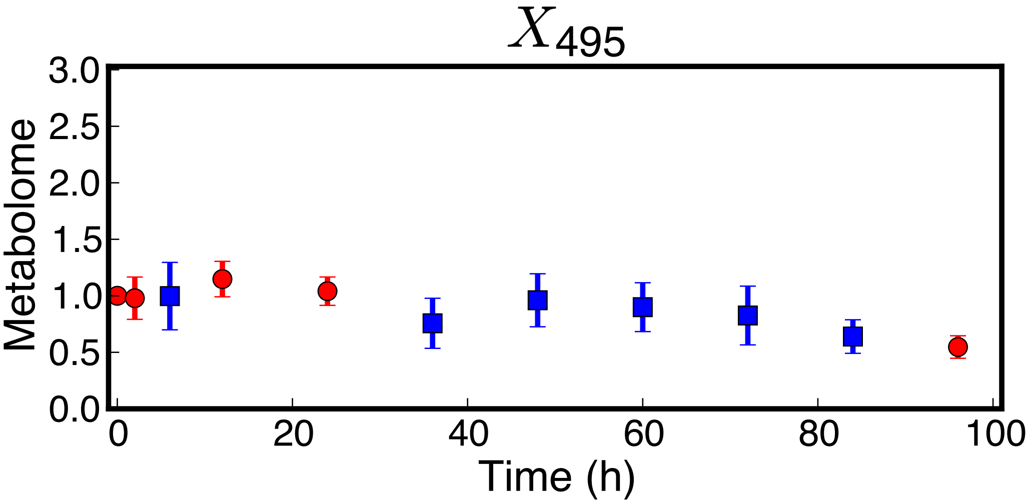

X_{495}
3.0
2.5
2.0
1.5
1.0
0.5
0.0
Metabolome
0
20
40
60
80
100
Time (h)

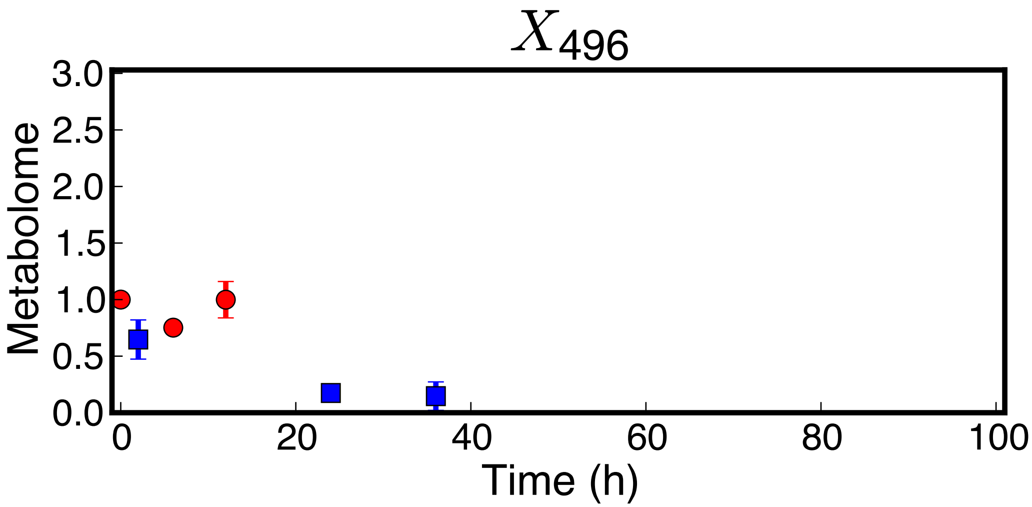

X_{496}
Metabolome
3.0
2.5
2.0
1.5
1.0
0.5
0.0
0
20
40
60
80
100
Time (h)

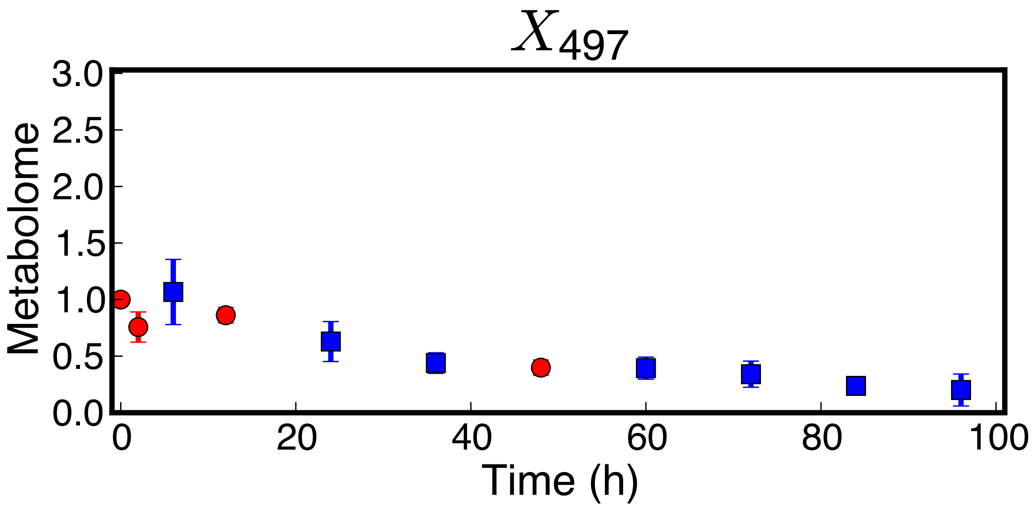

X_{497}
3.0
2.5
2.0
1.5
1.0
0.5
0.0
Metabolome
0
20
40
60
80
100
Time (h)

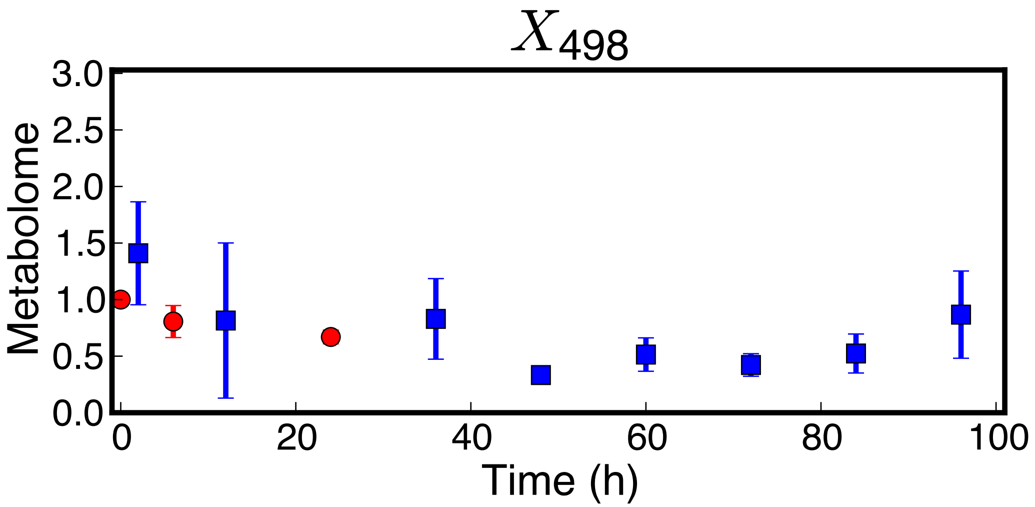
X_{498}
3.0
2.5
2.0
1.5
1.0
0.5
0.0
Metabolome
0
20
40
60
80
100
Time (h)

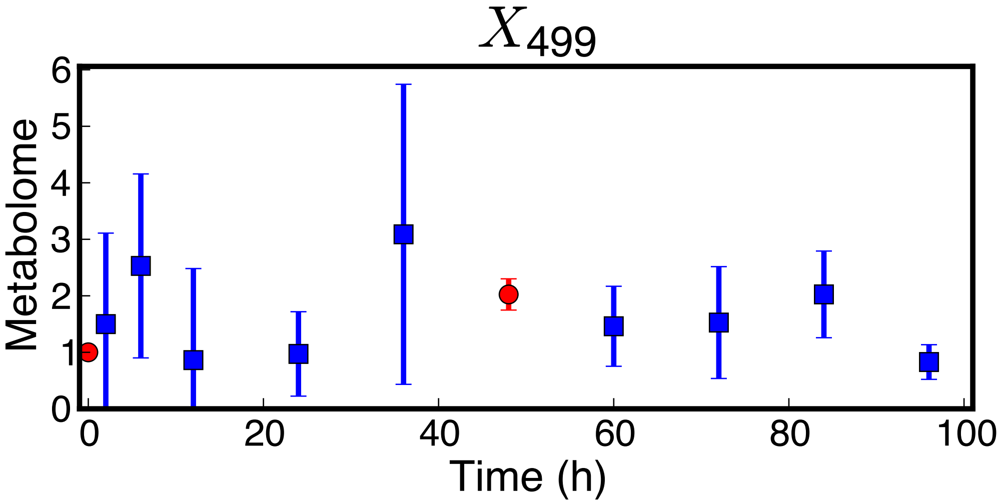

X_{499}
Metabolome
Time (h)
0
1
2
3
4
5
6
0
20
40
60
80
100

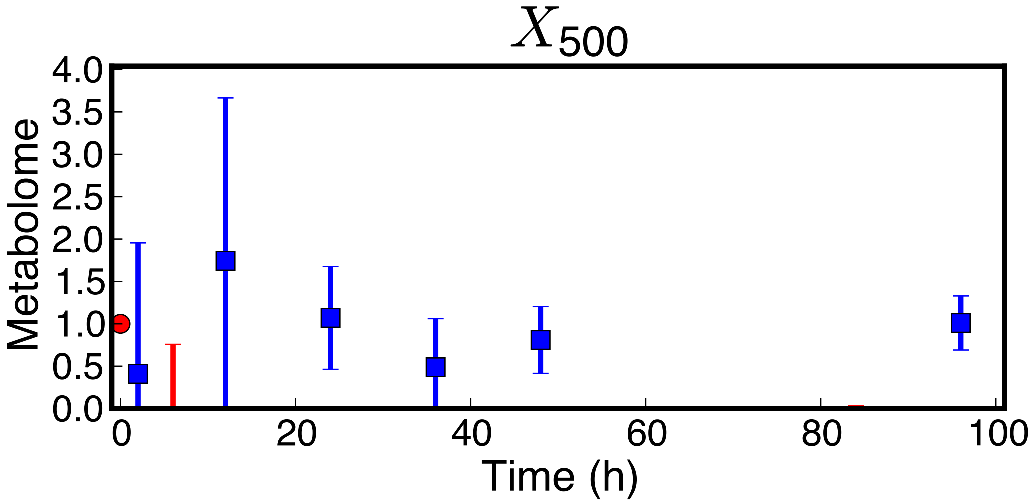

X_{500}
Metabolome
4.0
3.5
3.0
2.5
2.0
1.5
1.0
0.5
0.0
0
20
40
60
80
100
Time (h)

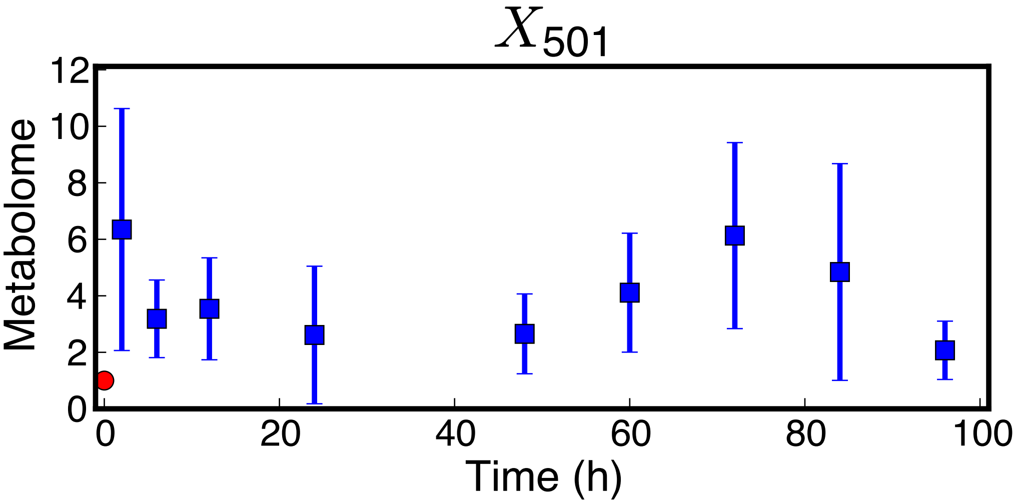

X_{501}
Metabolome
Time (h)
0
2
4
6
8
10
12
0
20
40
60
80
100

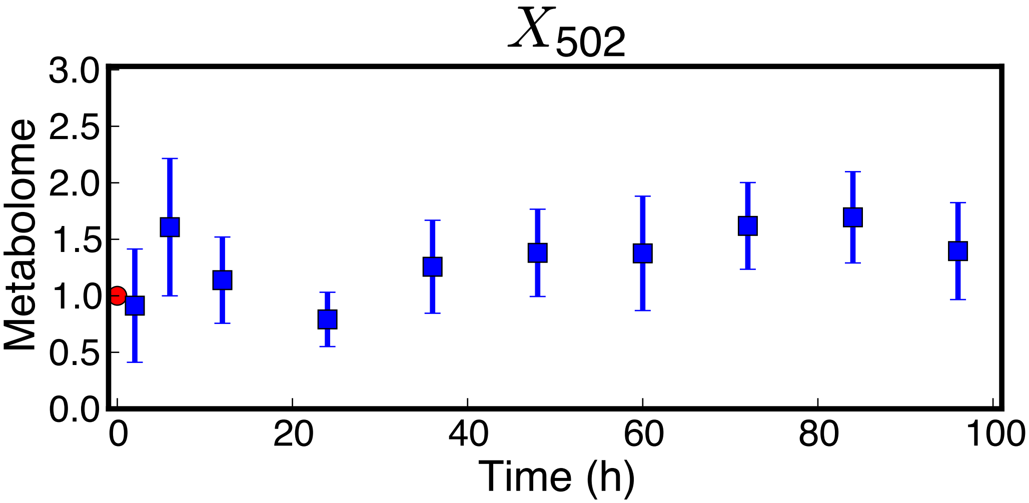

X_{502}
Metabolome
3.0
2.5
2.0
1.5
1.0
0.5
0.0
0
20
40
60
80
100
Time (h)

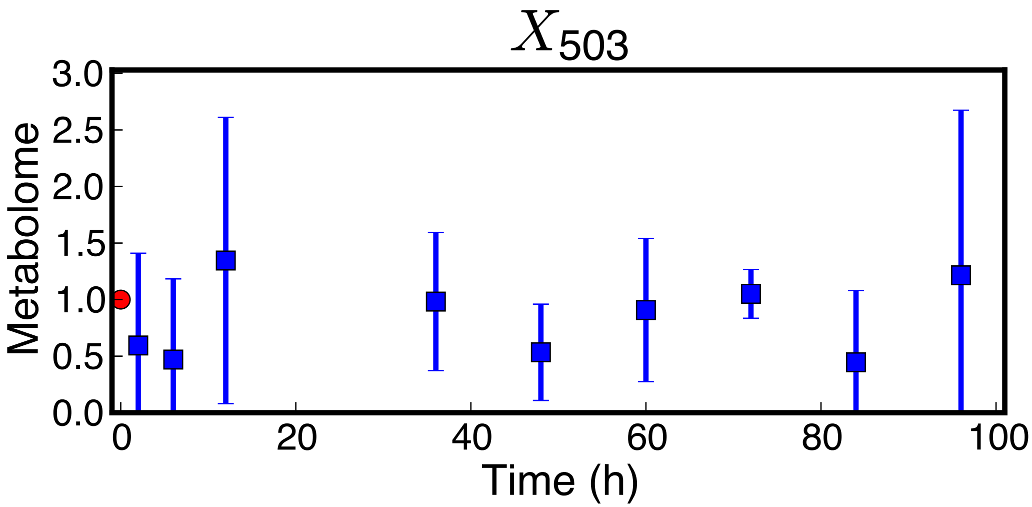

X_{503}
Metabolome
3.0
2.5
2.0
1.5
1.0
0.5
0.0
0
20
40
60
80
100
Time (h)

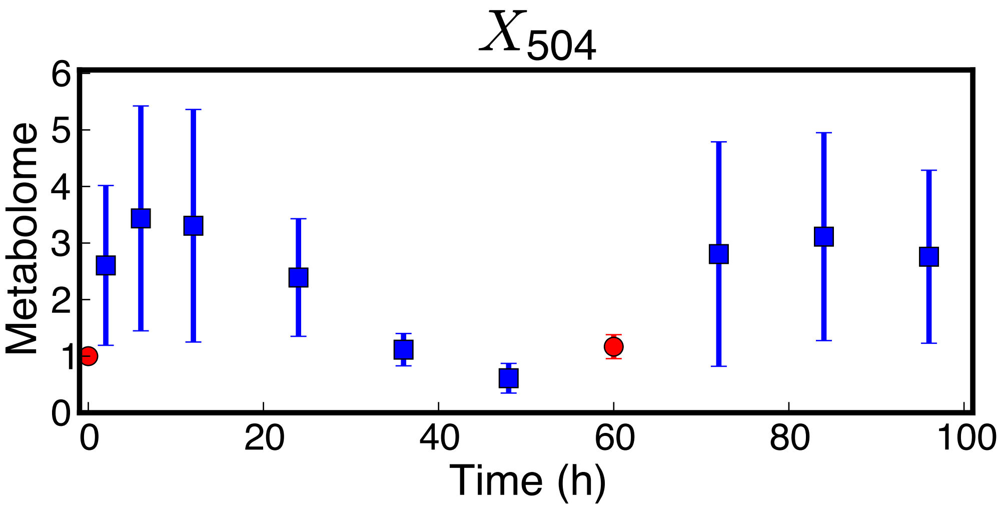
X_{504}
Metabolome
Time (h)
0
1
2
3
4
5
6
0
20
40
60
80
100

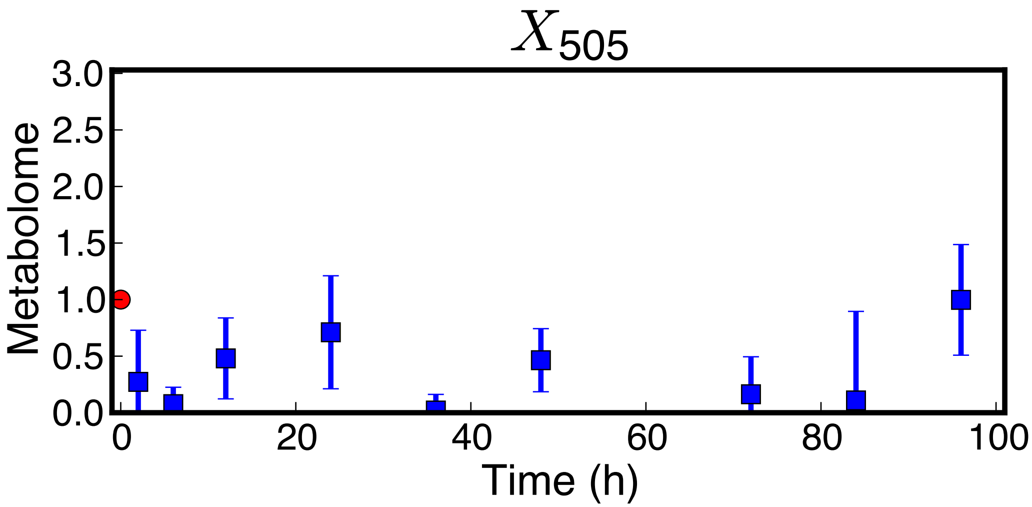

X_{505}
3.0
2.5
2.0
1.5
1.0
0.5
0.0
Metabolome
0
20
40
60
80
100
Time (h)

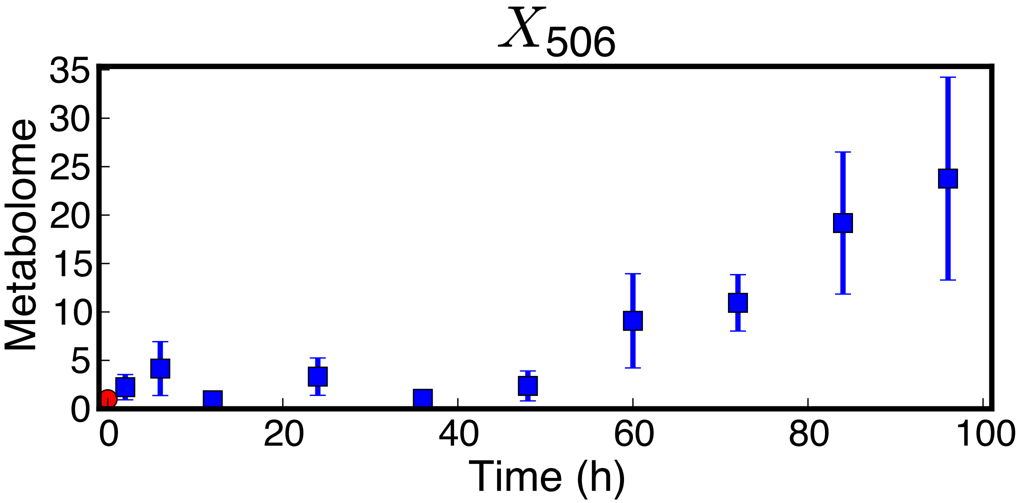

X_{506}
Metabolome
Time (h)
0
5
10
15
20
25
30
35
0
20
40
60
80
100

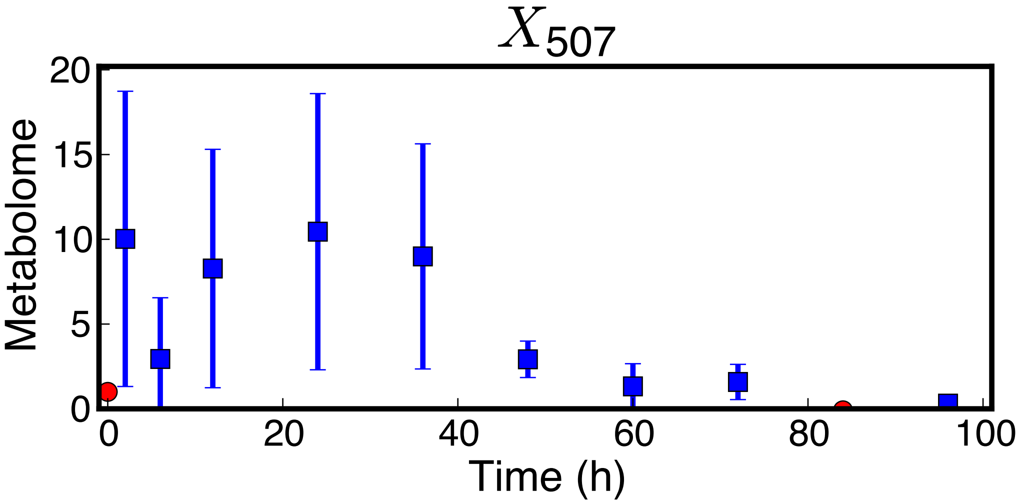

X_{507}
Metabolome
Time (h)
0
5
10
15
20
0
20
40
60
80
100

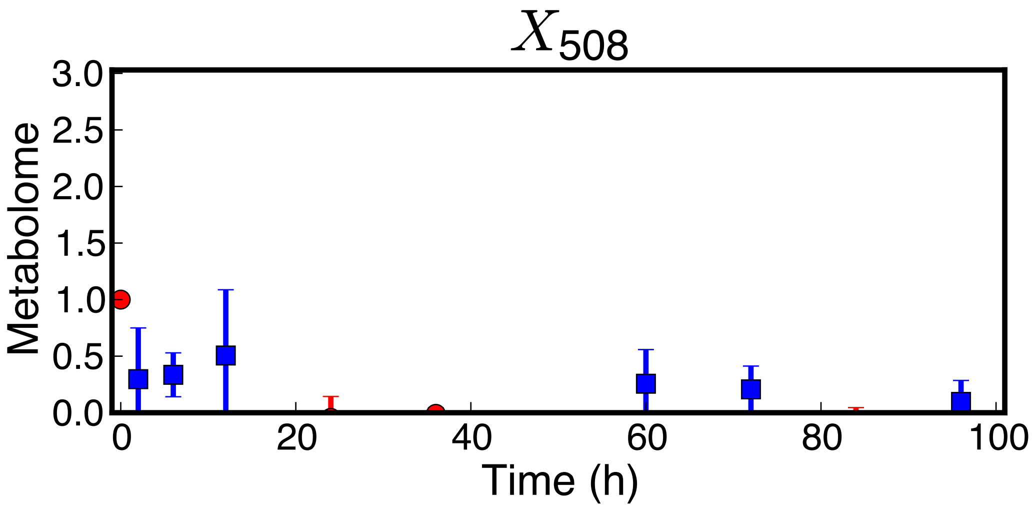

X_{508}
Metabolome
3.0
2.5
2.0
1.5
1.0
0.5
0.0
0
20
40
60
80
100
Time (h)

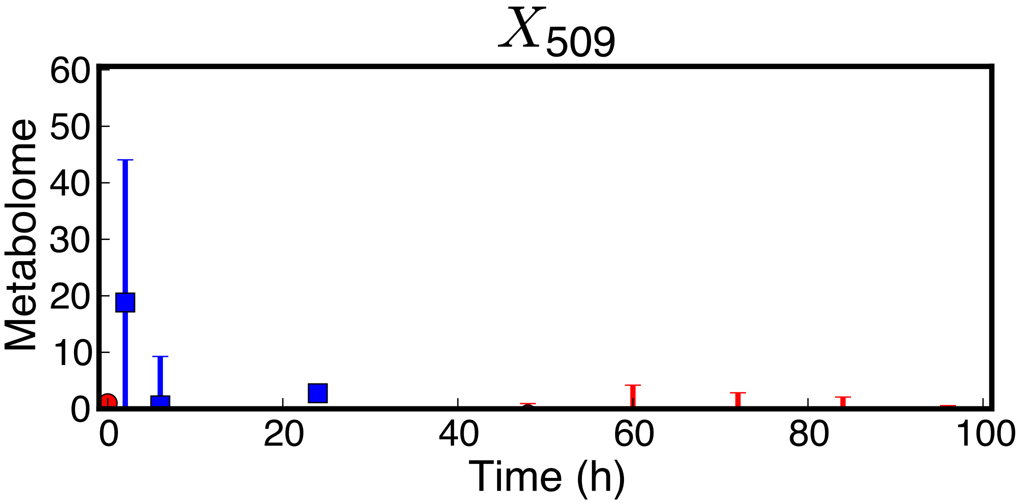

X_{509}
Metabolome
0
10
20
30
40
50
60
0
20
40
60
80
100
Time (h)

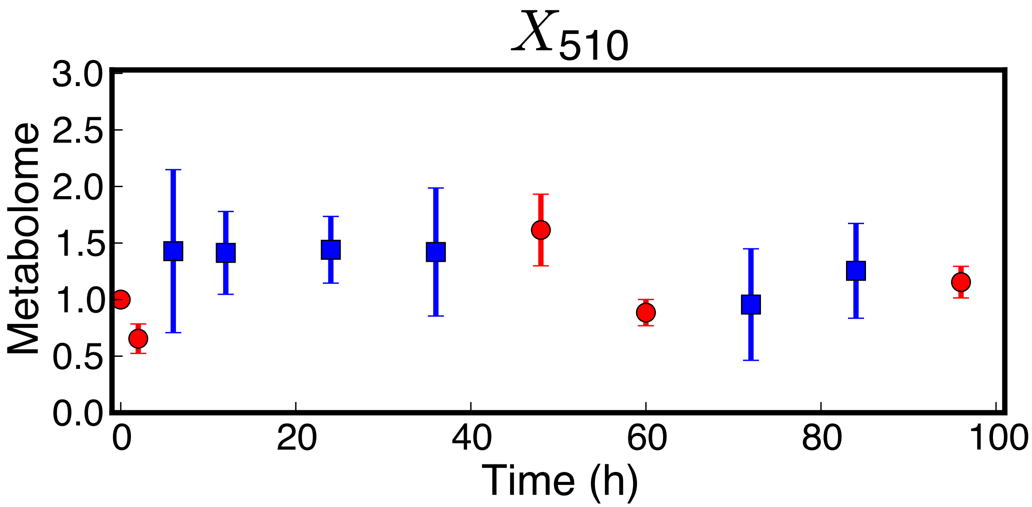
X_{510}
Metabolome
3.0
2.5
2.0
1.5
1.0
0.5
0.0
0
20
40
60
80
100
Time (h)

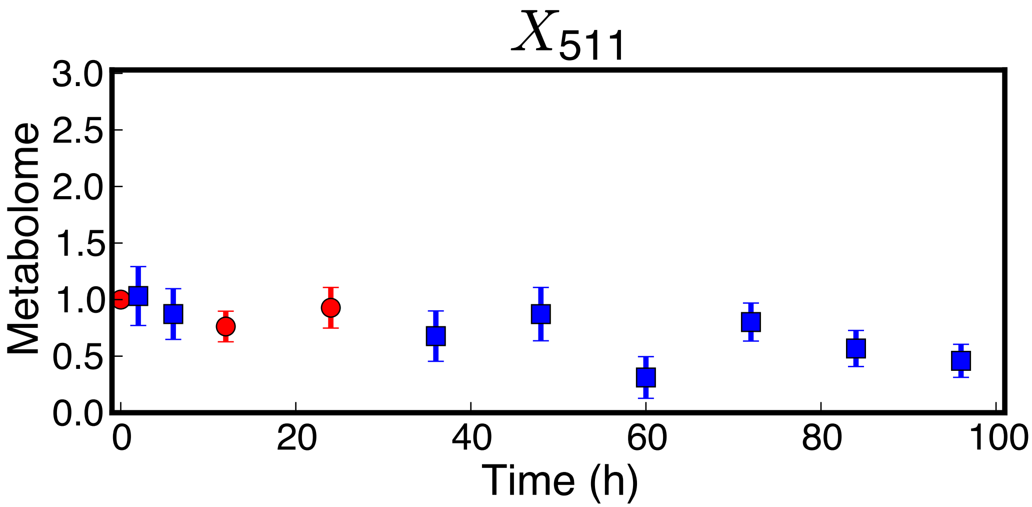
X_{511}
3.0
2.5
2.0
1.5
1.0
0.5
0.0
Metabolome
0
20
40
60
80
100
Time (h)

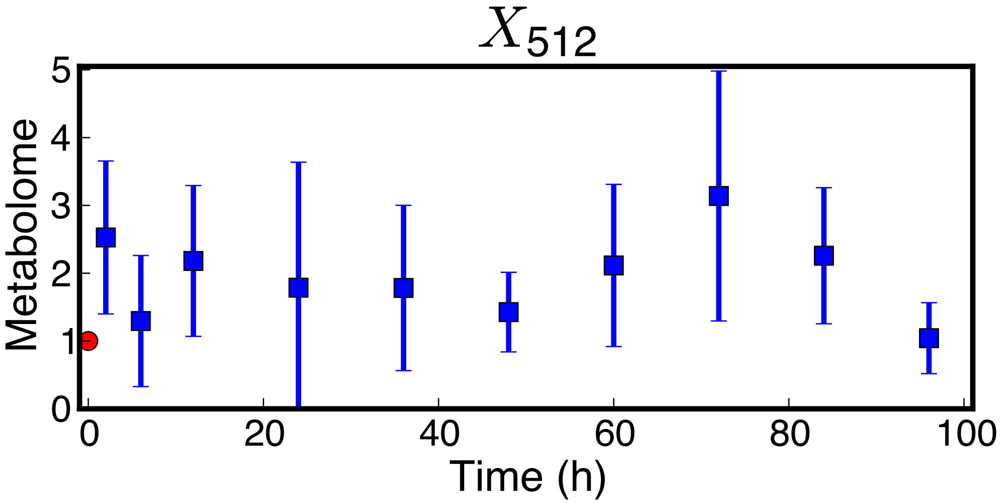

X_{512}
Metabolome
0
1
2
3
4
5
0
20
40
60
80
100
Time (h)

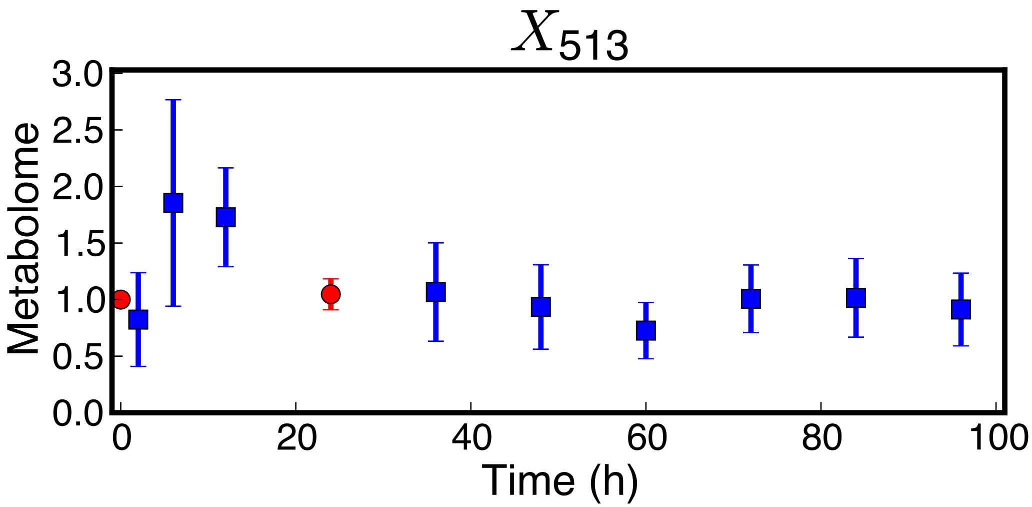

X_{513}
Metabolome
3.0
2.5
2.0
1.5
1.0
0.5
0.0
0
20
40
60
80
100
Time (h)

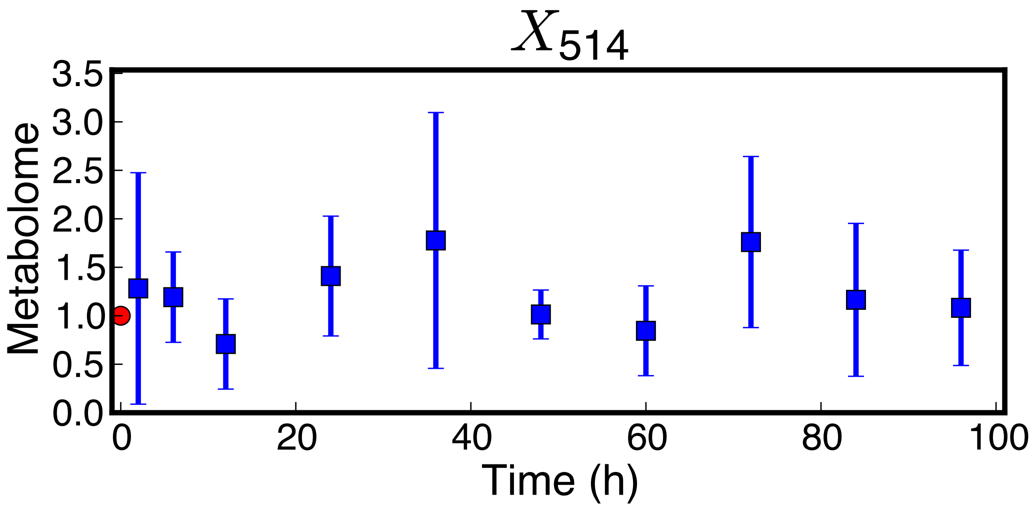

X_{514}
Metabolome
Time (h)
0.0
0.5
1.0
1.5
2.0
2.5
3.0
3.5
0
20
40
60
80
100

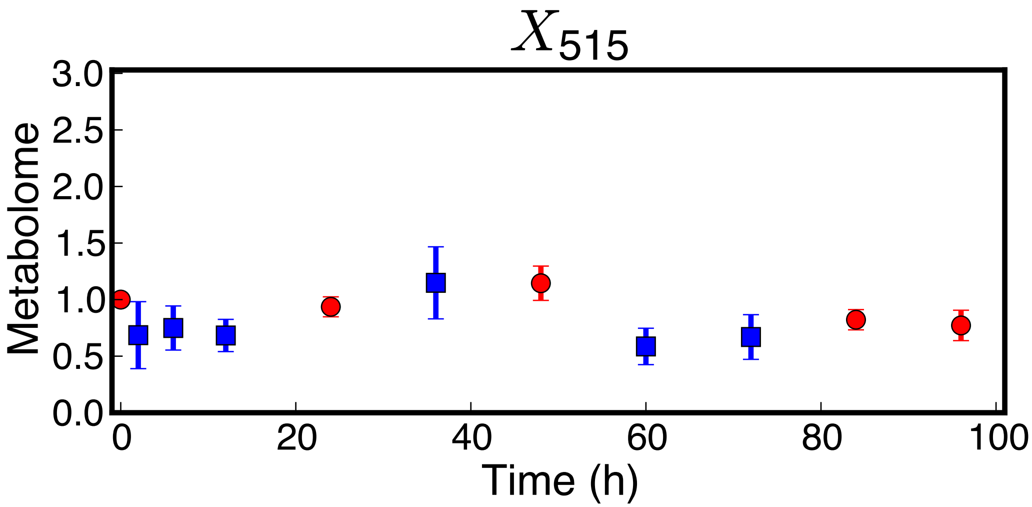

X_{515}
Metabolome
3.0
2.5
2.0
1.5
1.0
0.5
0.0
0
20
40
60
80
100
Time (h)

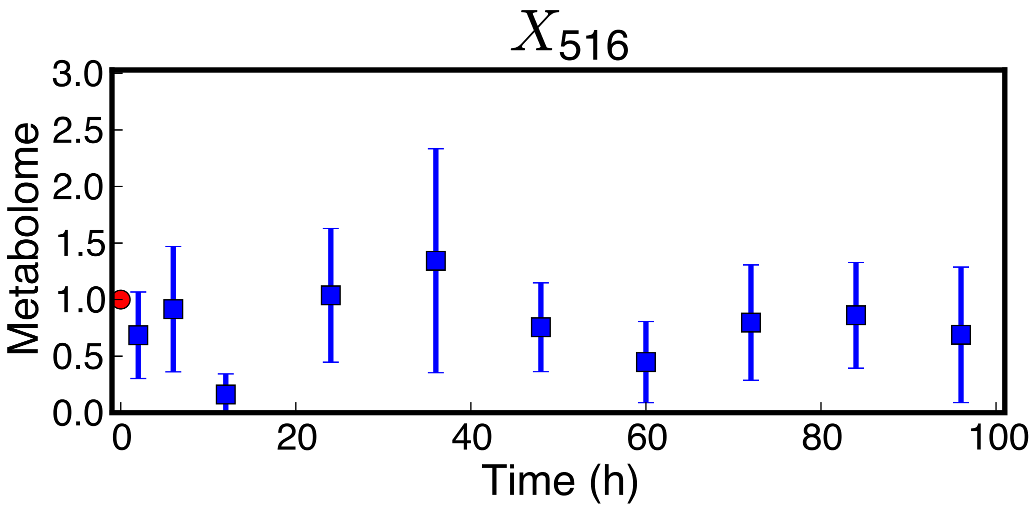

X_{516}
Metabolome
Time (h)
3.0
2.5
2.0
1.5
1.0
0.5
0.0
0
20
40
60
80
100

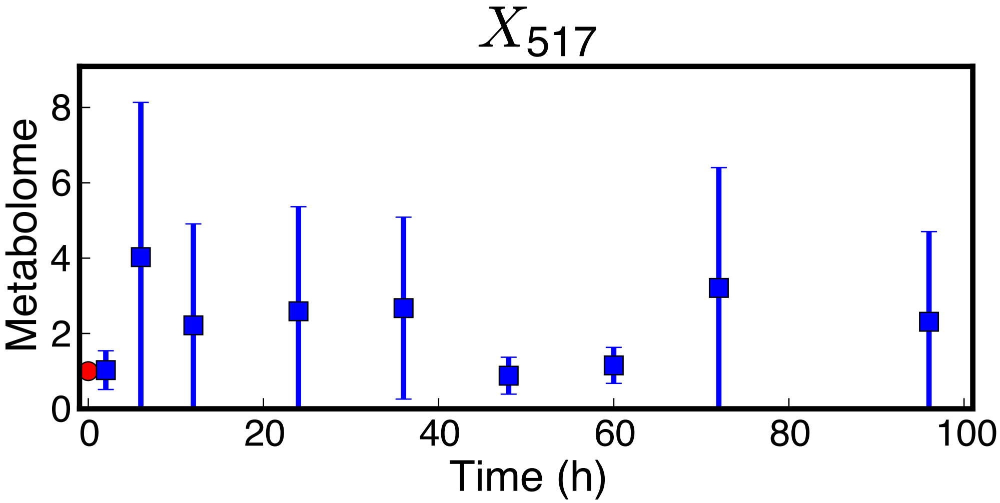

X_{517}
Metabolome
0
2
4
6
8
0
20
40
60
80
100
Time (h)

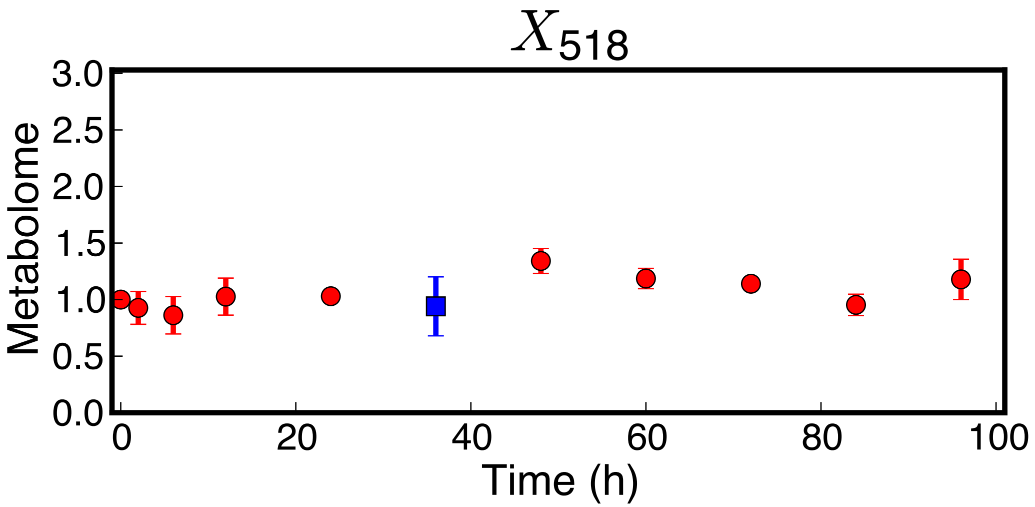
X_{518}
Metabolome
3.0
2.5
2.0
1.5
1.0
0.5
0.0
0
20
40
60
80
100
Time (h)

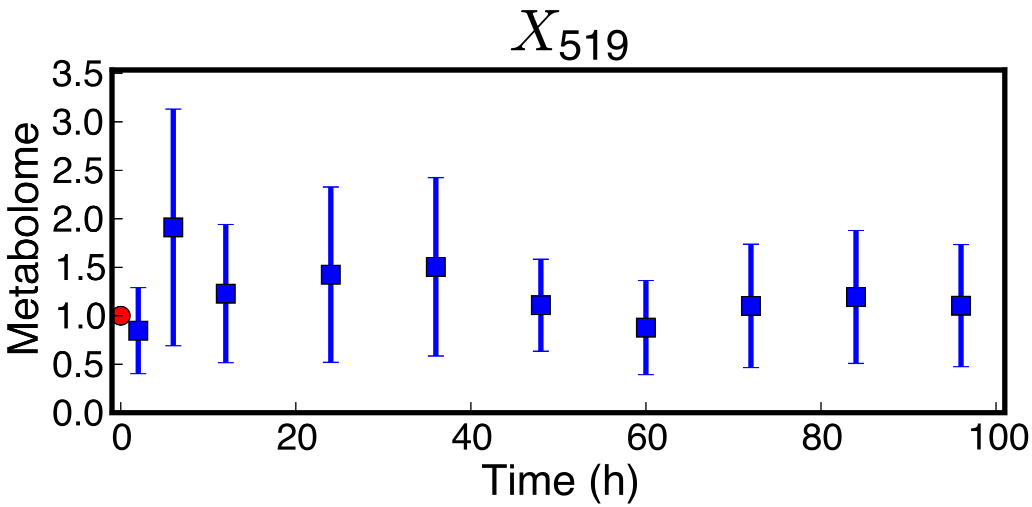
X_{519}
Metabolome
3.5
3.0
2.5
2.0
1.5
1.0
0.5
0.0
0
20
40
60
80
100
Time (h)

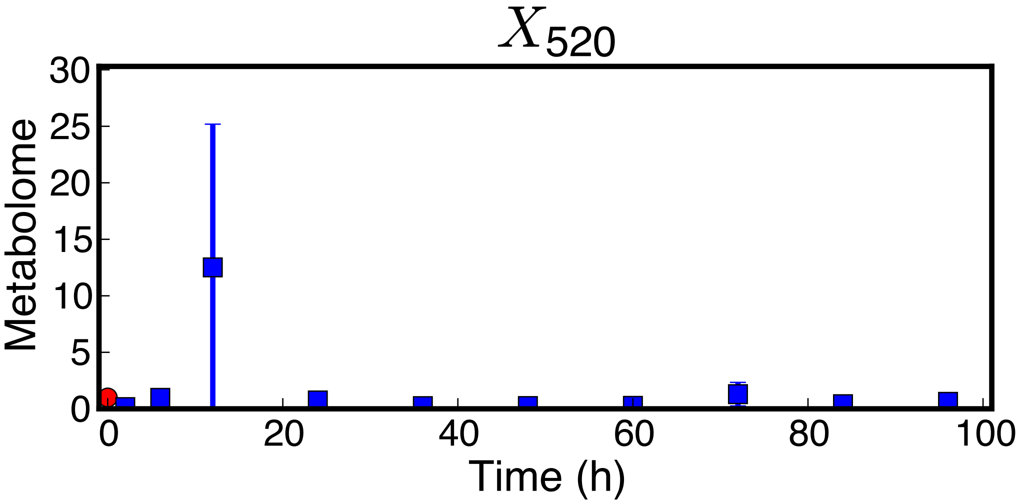

X_{520}
Metabolome
Time (h)
0
5
10
15
20
25
30
0
20
40
60
80
100

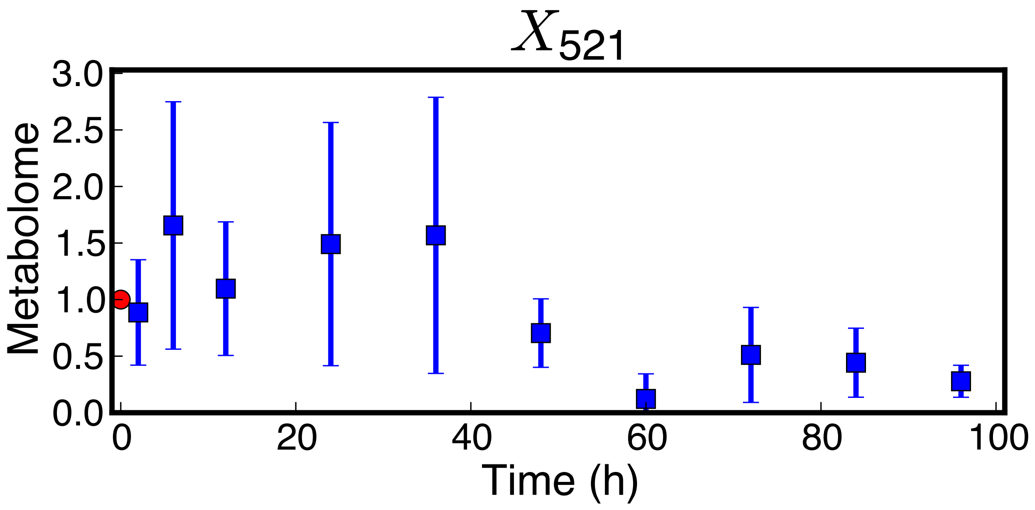
X_{521}
Metabolome
3.0
2.5
2.0
1.5
1.0
0.5
0.0
0
20
40
60
80
100
Time (h)

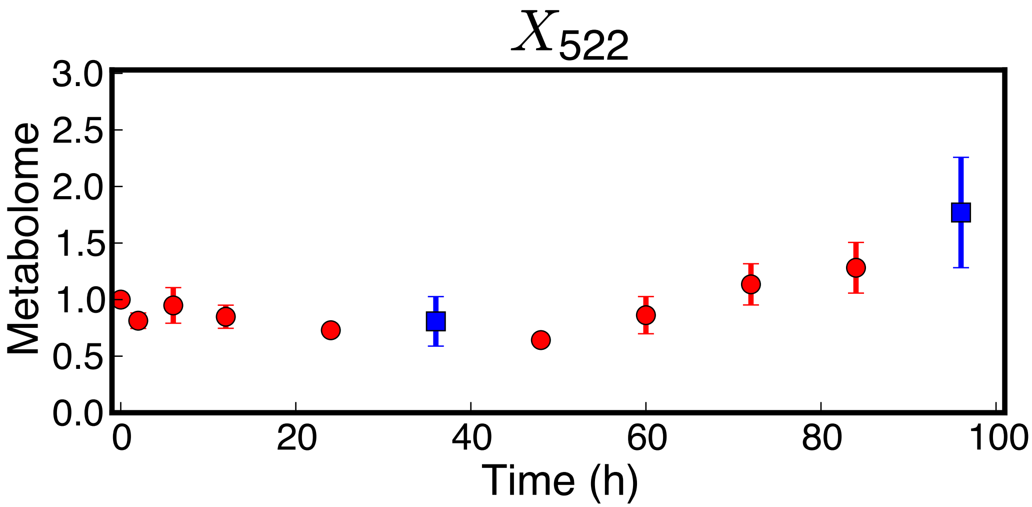
X_{522}
Metabolome
3.0
2.5
2.0
1.5
1.0
0.5
0.0
0
20
40
60
80
100
Time (h)

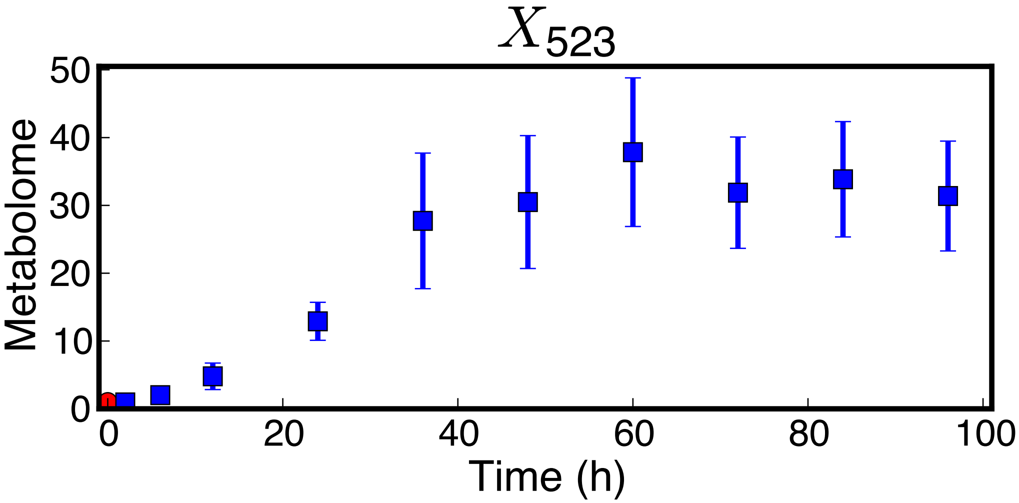

X_{523}
50
40
30
20
10
0
0
20
40
60
80
100
Metabolome
Time (h)

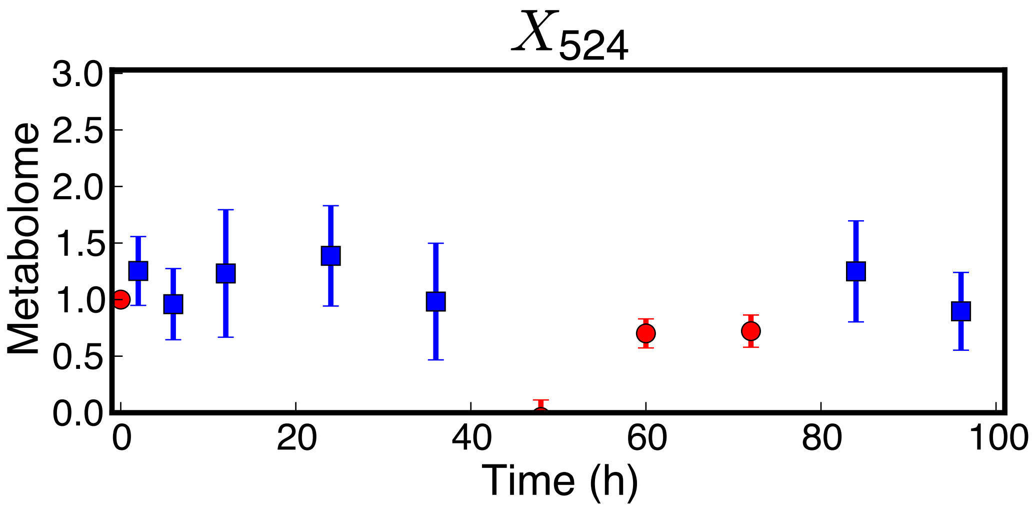

X_{524}
3.0
2.5
2.0
1.5
1.0
0.5
0.0
Metabolome
0
20
40
60
80
100
Time (h)

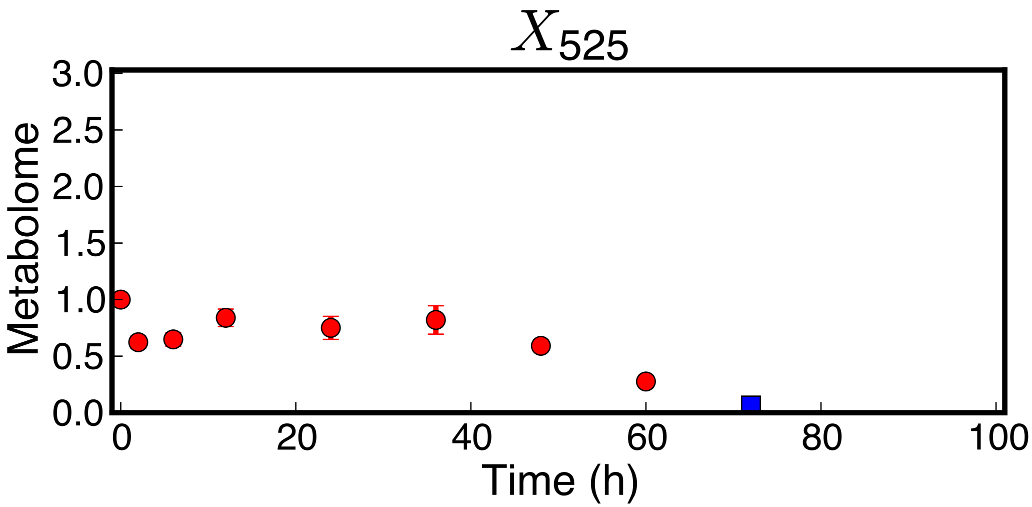

X525
3.0
2.5
2.0
1.5
1.0
0.5
0.0
Metabolome
0
20
40
60
80
100
Time (h)

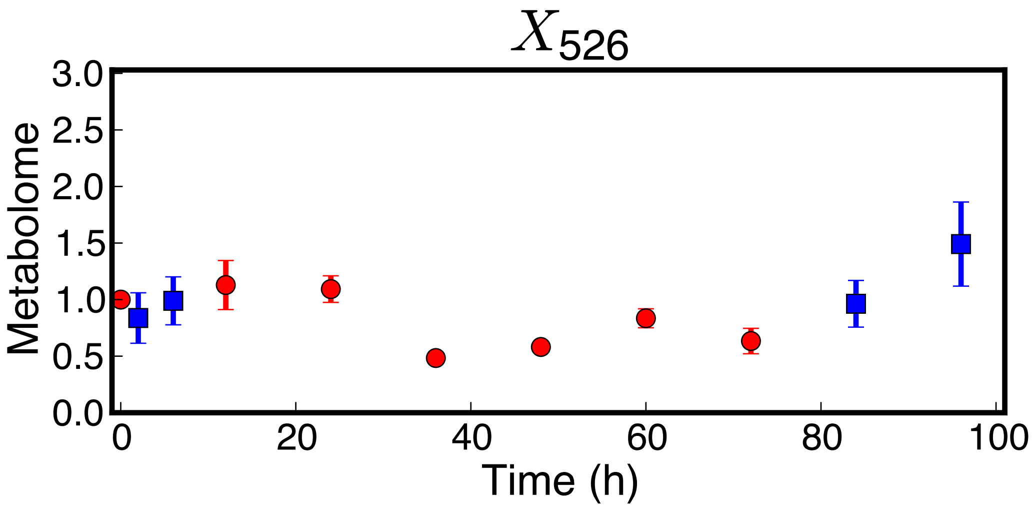

X_{526}
Metabolome
3.0
2.5
2.0
1.5
1.0
0.5
0.0
0
20
40
60
80
100
Time (h)

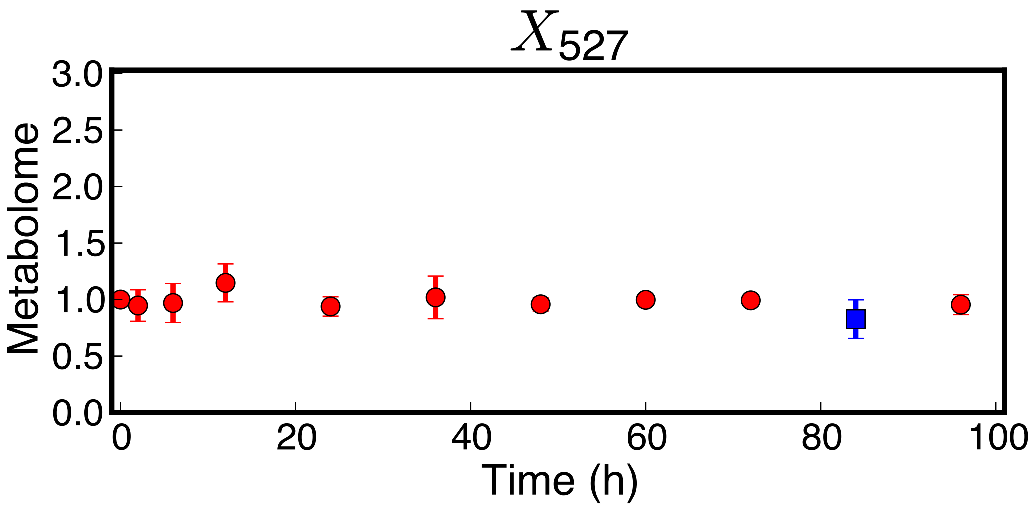

X_{527}
Metabolome
3.0
2.5
2.0
1.5
1.0
0.5
0.0
0
20
40
60
80
100
Time (h)

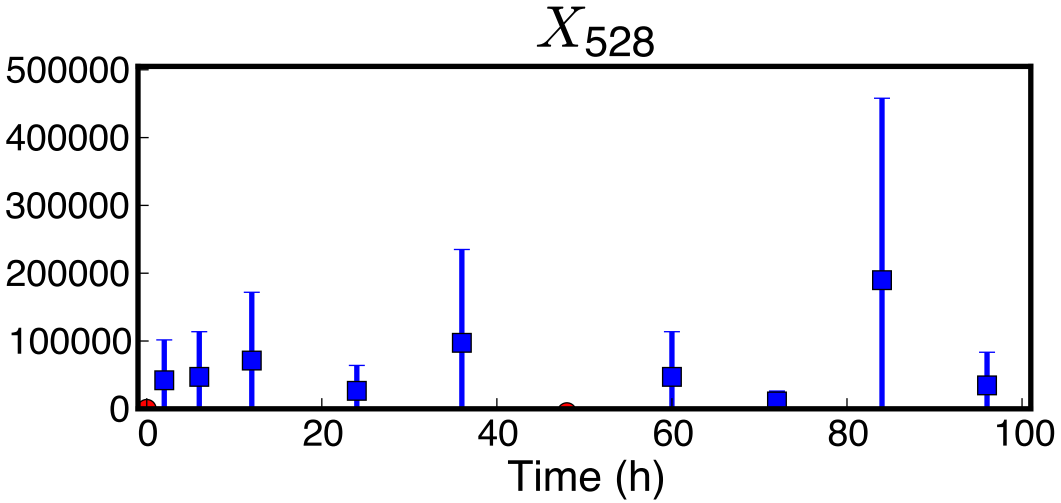

X_{528}
500000
400000
300000
200000
100000
0
0
20
40
60
80
100
Time (h)

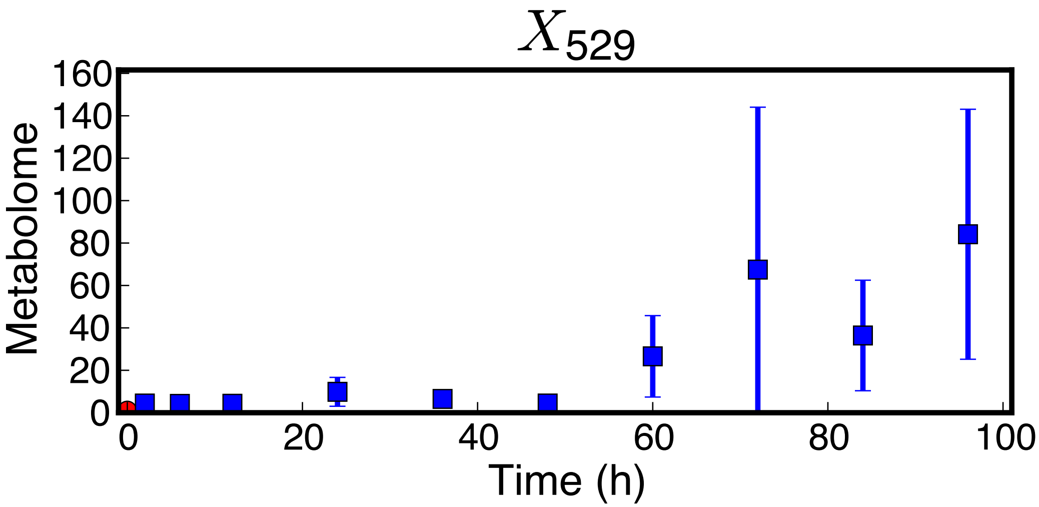

X_{529}
Metabolome
Time (h)
0
20
40
60
80
100
120
140
160
0
20
40
60
80
100

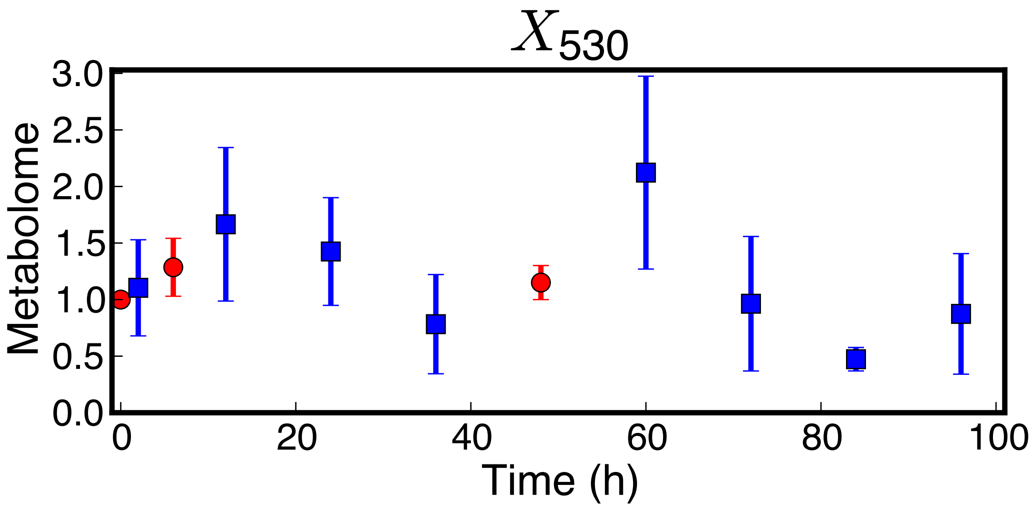

X_{530}
Metabolome
3.0
2.5
2.0
1.5
1.0
0.5
0.0
0
20
40
60
80
100
Time (h)

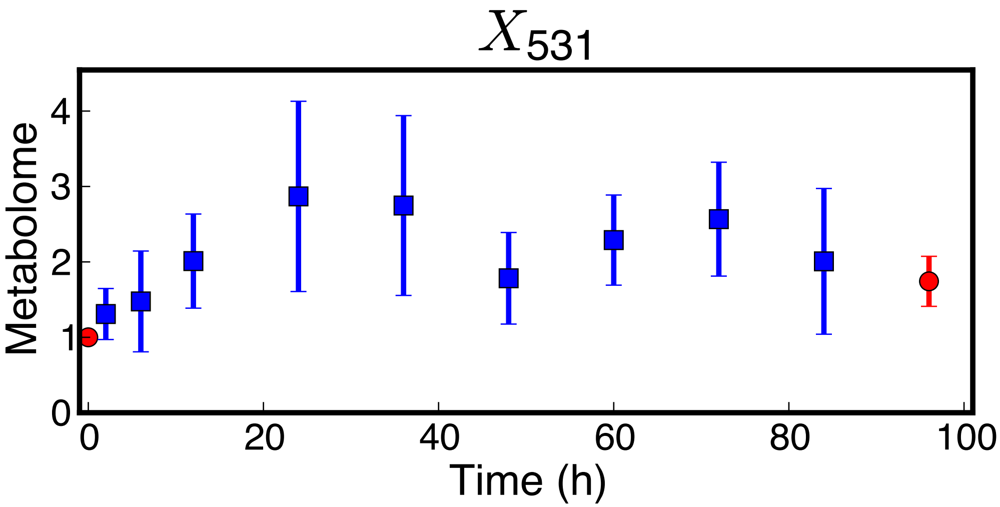

X_{531}
Metabolome
Time (h)
0
1
2
3
4
0
20
40
60
80
100

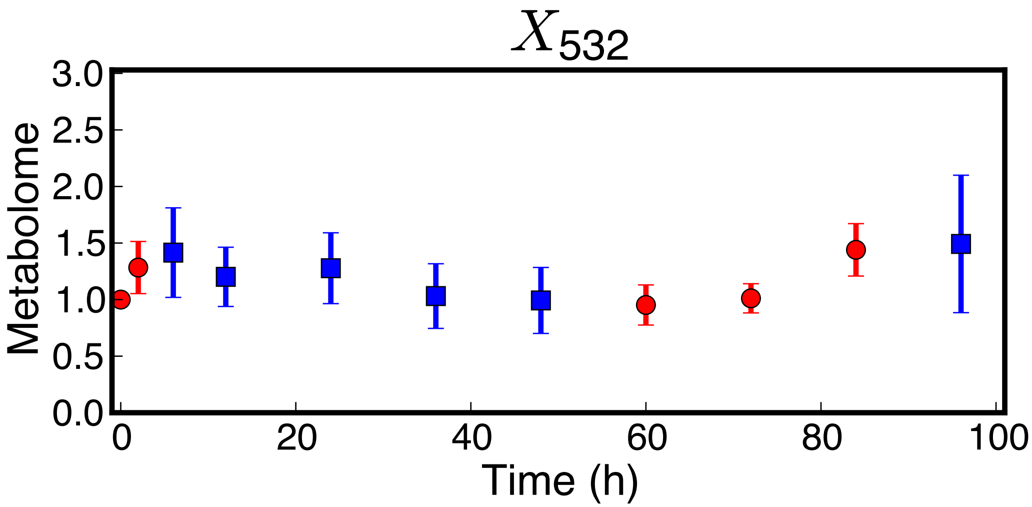

X_{532}
3.0
2.5
2.0
1.5
1.0
0.5
0.0
Metabolome
0
20
40
60
80
100
Time (h)

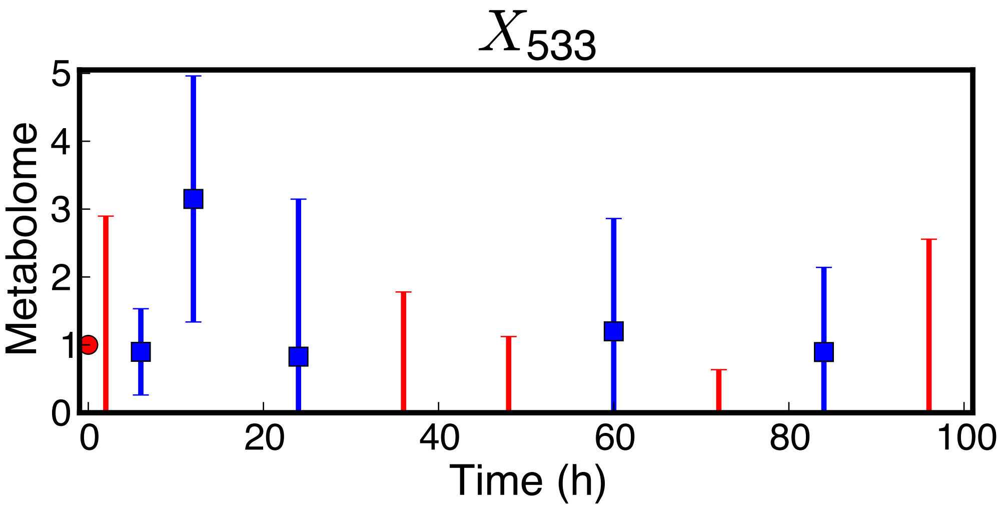

X_{533}
Metabolome
Time (h)
0
1
2
3
4
5
0
20
40
60
80
100
